# Formation of C–B, C–C, and C–X Bonds from Nonstabilized Aryl Radicals Generated from Diaryl Boryl Radicals

**DOI:** 10.1021/acscentsci.3c00993

**Published:** 2023-11-13

**Authors:** Fuyang Yue, Henan Ma, Pengxuan Ding, Hongjian Song, Yuxiu Liu, Qingmin Wang

**Affiliations:** State Key Laboratory of Elemento-Organic Chemistry, Research Institute of Elemento-Organic Chemistry, Frontiers Science Center for New Organic Matter, College of Chemistry, Nankai University, Tianjin 300071, People’s Republic of China

## Abstract

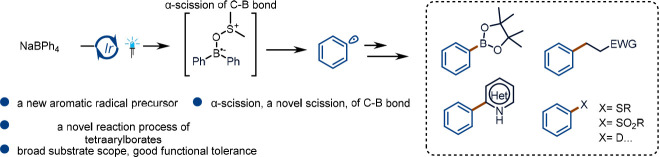

With the development of organoboron chemistry, boron-centered radicals have become increasingly attractive. However, their synthetic applications remain limited in that they have been used only as substrates for addition reactions or as initiators for catalytic reactions. We have achieved a new reaction pathway in which tetraarylborate salts are used as precursors for aryl radicals via boron radicals, by introducing a simple activation reagent. In addition, we carried out a diverse array of transformations involving these aryl radical precursors, which allowed the construction of new C–B, C–C, and C–X bonds in the presence of visible light.

## Abstract 2

A reaction pathway is achieved in which tetraarylborate salts are used as precursors for aryl radicals via boron radicals, and transformations are carried out involving these aryl radical precursors.

## Introduction

**Figure 1:**
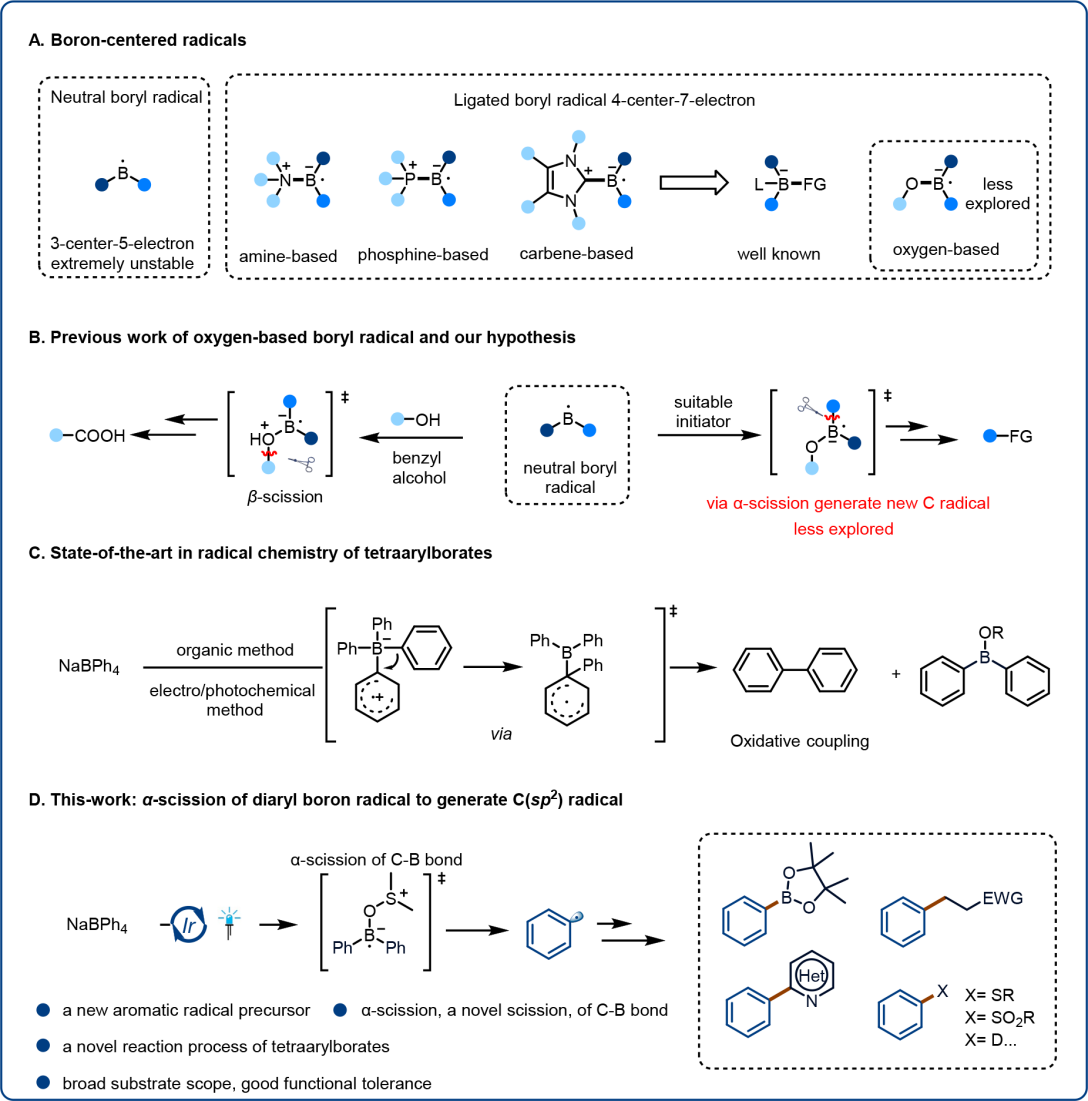
From inspiration to reaction design. (A) Typical boron-centered radicals. (B) Boryl radical activation by a C(sp^3^)–OH bond, β-scission, and our hypothesis. (C) State of the art in the radical chemistry of tetraarylborates. (D) α-Scission of a diaryl boryl radical to generate a C(sp^2^) radical.

Radicals, which can be generated from many feedstock chemicals, are among the most fundamental intermediates in synthetic chemistry and have become useful tools for developing novel methodologies.^1−12^ With the development of organoboron chemistry, boron-centered radicals have become more and more attractive, but their synthetic applications remain limited.^13−16^ For example, the applications of neutral boryl radicals, which are three-center–five-electron radicals, are limited because of their extreme electron deficiency (Figure 1A). In contrast, four-center–seven-electron boryl radicals ligated with a Lewis base (usually a carbene, a phosphine, or an amine) are relatively stable and have been extensively studied.^17−25^ These Lewis base-based boryl radicals are known to react with alkenes and heteroaromatic rings, and such reactions have been used to modify drug molecules.^26−35^ In recent years, the groups of Wang^4^ and Li^5^ have reported some elegant uses of amine-based boron free radicals as catalysts.^34,35^ In 2022, Xia’s group^7^ reported a method for alkyl radical generation by direct splitting of the C–O bonds of alcohol–boron radical intermediates; in these reactions, various alcohols were successfully used as alkyl radical precursors (Figure 1B). 

Despite the beautiful work that has been accomplished with boron radicals, they have been used only as substrates for addition reactions or as initiators for catalytic reactions, which is still limited. We envisioned that boron free radicals with unique electronic properties could have more special reaction types. For example, we hypothesized that when coordinated by a simple reagent, neutral boron-centered radicals would undergo α-scission to generate carbon radicals (Figure 1B). To test our hypothesis, we needed to consider several criteria. First, the precursor of the neutral boron radical should be inexpensive, stable, and readily available. Second, the conditions for producing the radical should be mild and operationally simple. Third, both the neutral boron radical and the reagent-coordinated boron radical should be weakly nucleophilic and should not readily participate in addition reactions. If we could satisfy these criteria, we would be able expand the application scope of boron radicals and provide new free-radical precursors for the development of new synthetic methodologies.

In chemistry pioneered by Xia^36^ and others^37−47^ sodium tetraphenylborate, which can be easily synthesized or purchased commercially, can be oxidized under electrochemical, thermal, or photochemical conditions to produce biphenyl compounds (Figure 1C). In these reactions, the most electron-rich aryl moiety undergoes one-electron oxidation; the resulting intermediate undergoes an intramolecular 1,2-aryl shift to afford a cyclohexadienyl radical, and, finally, departure of biphenyl generates a diaryl boron radical, which is captured by other chemical species in the reaction system. Inspired by this work, we thought that tetraarylborate salts would be an ideal source of diaryl boron radicals because boron radicals can be produced under mild conditions and the byproducts do not affect the reaction system. Furthermore, we envisioned that after the introduction of a suitable initiator, the diaryl boron radicals would undergo α-scission of the C–B bond to produce aryl radicals in situ. Indeed, we herein report a novel strategy for the generation of aryl radicals from sodium tetraarylborates, which have previously been used for aryl coupling reactions,^37−47^ enabled by introduction of a suitable initiator to induce α-scission of the C–B bond (Figure 1D).

## Results and Discussion

Given the importance of alkylboron compounds as synthetic precursors for a wide range of valuable functional groups,^48^ we first applied the above-described strategy to a boronization reaction with the goal of identifying the most suitable initiator and optimizing the reaction conditions. For our initial experiments, we chose sodium tetraphenylborate (**1a**) and the boronating reagent B_2_pin_2_ (**2**) as model substrates (Table 1). A solution of the substrates in *N,N*-dimethylacetamide (DMA, [**1a**] = 0.2 M) containing methanol (2.0 equiv) as an activation reagent, Co(dmgH)_2_pyCl (20 mol %) as a transition-metal catalyst, and Ir[dF(CF_3_)ppy]_2_(dtbbpy)PF_6_ (2 mol %) as a photocatalyst was irradiated with a 36 W blue LED at room temperature under an air atmosphere for 24 h. Unfortunately, no borylation products were detected under these conditions (entry 1).

**Table 1:**
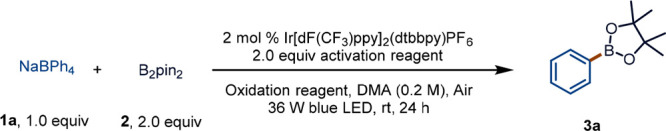
Optimization of Conditions for Borylation of Sodium Tetraphenylborate (**1a**) with Boronating Reagent B_2_pin_2_ (**2**)^a^

entry	activation reagent	catalyst or oxidation reagent	yield (%)^b^
1	methanol	Co(dmgH)_2_pyCl	NR
2	phenol	Co(dmgH)_2_pyCl	NR
3	*N*-methyl-2-pyrrolidone	Co(dmgH)_2_pyCl	NR
4	DMSO	Co(dmgH)_2_pyCl	19
5	DMSO	Co(dmgH)_2_Cl_2_	trace
6	DMSO	Co(dmgH)_2_(4-CO_2_Et)PyCl	trace
7^c^	DMSO	(NH_4_)_2_S_2_O_8_	32
8^d^	DMSO	(NH_4_)_2_S_2_O_8_	47
9^e^	DMSO	(NH_4_)_2_S_2_O_8_	75
10^f^	DMSO	(NH_4_)_2_S_2_O_8_	NR
11^c^	none	(NH_4_)_2_S_2_O_8_	NR

a Reaction conditions, unless otherwise noted: **1a** (0.4 mmol), **2** (0.8 mmol), Ir[dF(CF_3_)ppy]_2_(dtbbpy)PF_6_ (0.008 mmol), activation reagent (0.8 mmol, 2.0 equiv), catalyst (0.08 mmol, 0.2 equiv), *N,N*-dimethylacetamide (DMA, 2 mL), air, 36 W blue LED, rt, 24 h.

b Isolated yields are provided.

c (NH_4_)_2_S_2_O_8_ (0.8 mmol, 2.0 equiv).

d (NH_4_)_2_S_2_O_8_ (0.8 mmol, 2.0 equiv), DMSO (1.6 mmol, 4.0 equiv).

e (NH_4_)_2_S_2_O_8_ (0.8 mmol, 2.0 equiv), 5:1 (v/v) DMSO/DMA (2.0 mL).

f No light or no photocatalysis.

However, when we tested other activation reagents (entries 2–4 and the Supporting Information (SI)), we were delighted to find that when DMSO was present, borylated product **3a** was obtained in 19% yield (entry 4), which confirmed the feasibility of our strategy. In addition, we screened two different Co catalysts, but they afforded only a trace of the desired product (entries 5 and 6). Surprisingly, however, when we replaced the transition-metal catalyst with the inorganic oxidant (NH_4_)_2_S_2_O_8_ (2.0 equiv), the yield of **3a** increased from 19% to 32% (entry 7). Given this promising result, we carried out reactions with this oxidant and different amounts of the activation reagent (DMSO, entries 8 and 9). These experiments revealed that when DMSO was the solvent (2 mL of 5:1 [v/v] DMSO/DMA), the yield of **3a** increased from 32% to 75% (entry 9). Control experiments proved that the photocatalyst and light (entry 10) and the activation reagent (entry 11) were essential for the transformation.

**Figure 2:**
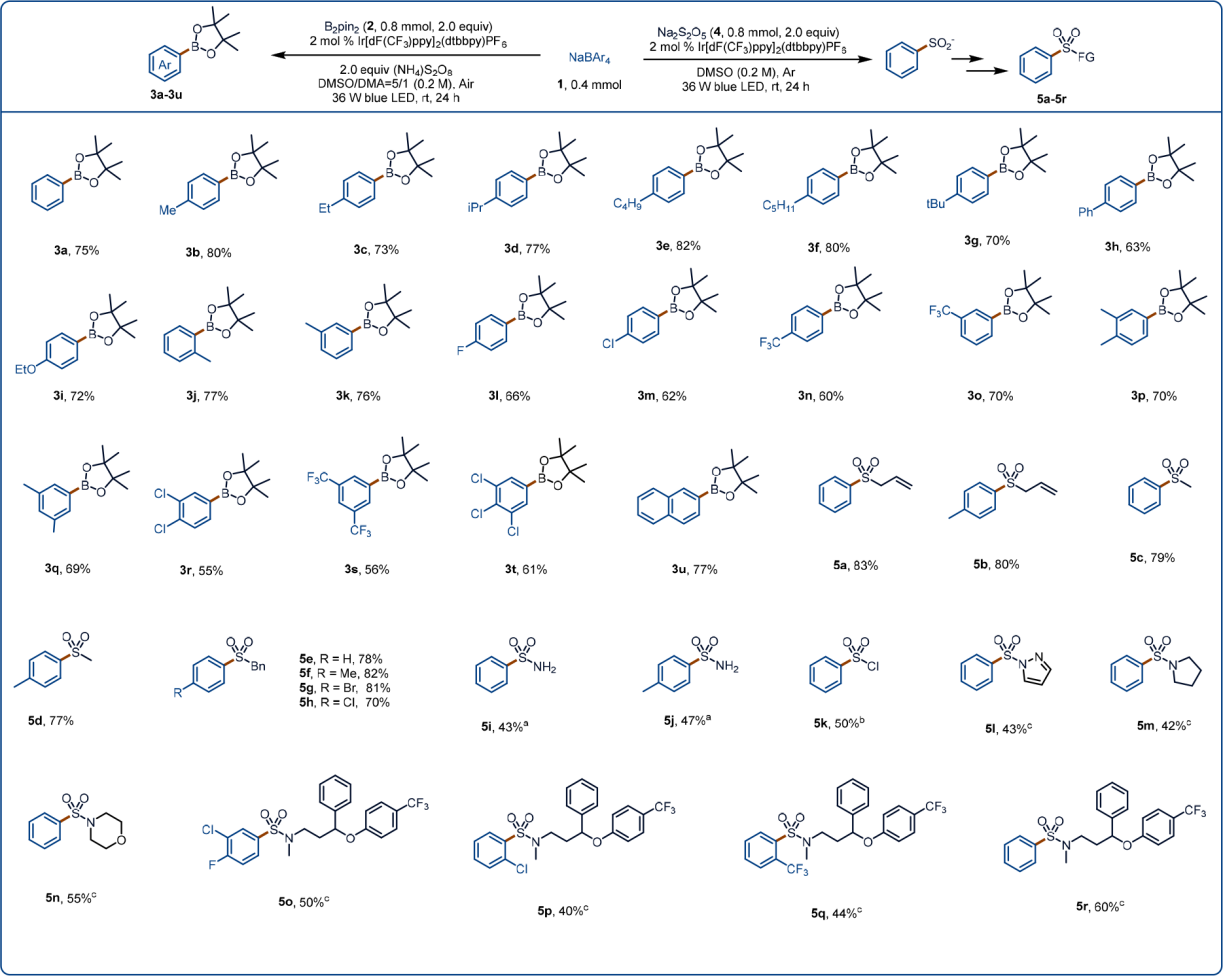
Substrate scope. Borylation conditions: **1** (0.4 mmol), B_2_pin_2_ (**2**, 0.8 mmol, 2.0 equiv), (NH_4_)_2_S_2_O_8_ (0.8 mmol, 2.0 equiv), Ir[dF(CF_3_)ppy]_2_(dtbbpy)PF_6_ (2 mol %), 5:1 (v/v) DMSO/DMA (2.0 mL), air, 36 W blue LED, rt, 24 h. Sulfinylation conditions: **1** (0.4 mmol), Na_2_S_2_O_5_ (**4**, 0.8 mmol, 2.0 equiv), Ir[dF(CF_3_)ppy]_2_(dtbbpy)PF_6_ (2 mol %), DMSO (2.0 mL), Ar, 36 W blue LED, 24 h; then addition of NaHCO_3_ (2.0 equiv), EtOH (1 mL), and R–X (1.5 equiv), stirring at rt for 16 h. See the SI for complete experimental details. DMA, *N,N*-dimethylacetamide. ^*a*^One-pot access to sulfonamides; see the SI for complete experimental details. ^*b*^One-pot access to a sulfonylhalide; see the SI for complete experimental details. *^c^*Two-step sulfonamide synthesis via a sulfonyl chloride; see the SI for complete experimental details.

Using the optimized conditions (Table 1, entry 9), we evaluated the substrate scope of the photocatalytic borylation reaction by testing a series of these new aryl radical precursors (Figure 2). A wide range of sodium tetraarylborates were viable, furnishing desired aryl boronic ester products **3**. Tetraarylborates with an alkyl substituent were suitable substrates, giving **3b**–**3g** in 70–82% yields. Substrates with other electron-donating groups (i.e., phenyl [**3h**] and alkoxy [**3i**]) were also compatible with the reaction conditions. The yields were approximately the same regardless of the position of the substituent (compare **3b**, **3j**, and **3k**). Tetraarylborates with electron-withdrawing substituents were converted to the corresponding boronic esters (**3l**–**3o**) in moderate yields, as were disubstituted tetraarylborates (**3p**–**3s**). A trisubstituted tetraarylborate was tolerated as well (**3t**, 61%). Even sodium tetranaphthylborate gave the corresponding product (**3u**, 77%).

We envisioned that these radical precursors could be used for the synthesis of sulfones, sulfonamides, which are widely used functional groups and are present in a variety of functional materials, agricultural chemicals, and pharmaceuticals.^49−51^ The abundance of these functional groups in biologically active molecules underscores their importance: in approved drugs, sulfur-containing functional groups are even more common than fluorine- or phosphorus-containing groups.^52^ Therefore, we used our radical precursors to develop a new method for the synthesis of sulfur-containing compounds, starting with sulfones (Figure 2). We were pleased to find that when we changed the reaction conditions, we were able to convert radical precursors **1** to sulfur-containing products by Ir-catalyzed reactions with Na_2_S_2_O_5_ (**4**) in DMSO under irradiation with a blue LED and subsequent reactions with alkyl halides. Although the isolation of crude aryl sulfonates was possible, to facilitate the characterization and isolation of the final products, we instead used a one-pot procedure to convert the intermediate sulfites to benzyl sulfones **5a**–**5h** by reactions with various alkyl halides (R–X). To further illustrate the wide range of synthetic uses for our method, we carried out one-pot reactions of **1** with **4** to afford aryl sulfonamides **5i** and **5j** and sulfonyl chloride **5k**. In addition, a two-step protocol was developed for the conversion of aryl sulfonates into sulfonamides **5l**–**5r** via the corresponding sulfonyl chloride intermediates.

**Figure 3:**
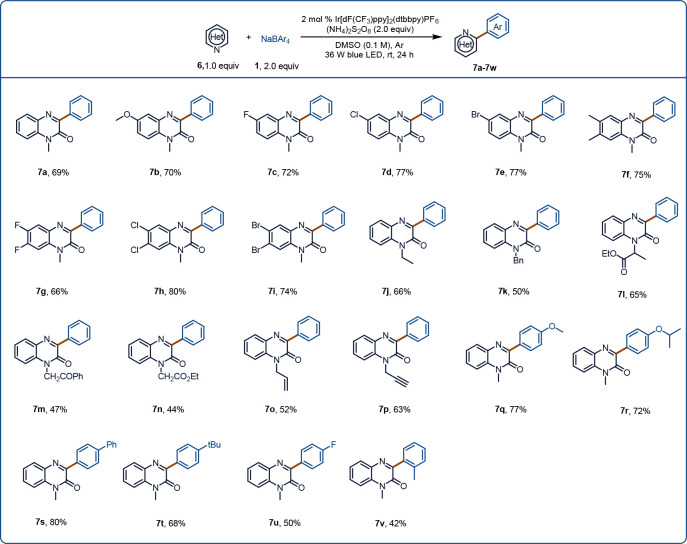
Substrate scope of the Minisci reaction. Reaction conditions: **1** (0.4 mmol, 2.0 equiv), **6** (0.2 mmol, 1.0 equiv), (NH_4_)_2_S_2_O_8_ (0.4 mmol, 2.0 equiv), Ir[dF(CF_3_)ppy]_2_(dtbbpy)PF_6_ (2 mol %), DMSO (2.0 mL), Ar, 36 W blue LED, rt, 24 h.

To demonstrate the universality of our approach, we planned to use it for other types of reactions, such as reactions of compounds with heteroaryl groups, which are widely found in natural products, organic materials, small-molecule drugs, and ligands for metal catalysts.^53,54^ Substituted heteroaryl groups can be obtained by Minisci reactions, which involve attack of a radical on a protonated heteroaromatic compound to generate a dearomatized intermediate, which then undergoes rearomatization. To realize our plan, we chose quinoxalin-2(1*H*)-ones as heteroaryl substrates and screened various reaction conditions, finally achieving efficient rearomatization of the intermediates under photocatalytic conditions. We then investigated the substrate scope by carrying out reactions of various quinoxalin-2(1*H*)-ones **6** with sodium tetraphenylborate (**1a**, Figure 3). *N*-Methyl quinoxalin-2(1*H*)-ones with methoxy, (di)fluoro, (di)chloro, (di)bromo, ester, or dimethyl substituents on the heteroaromatic ring reacted smoothly with **1a**, producing the corresponding products (**7a**–**7i**) in 66–80% yields. Moreover, quinoxalin-2(1*H*)-ones bearing substituents other than a methyl group on the nitrogen atom were also suitable substrates, providing **7j**–**7p**. Notably, the allyl group of **6o** and the alkyne of **6p** were retained under the reaction conditions. In addition, we were pleased to find that tetraarylborates with various substituents on the aryl ring showed good tolerance for the reaction conditions, affording desired products **7q**–**7v** in 42–80% yields.

**Figure 4:**
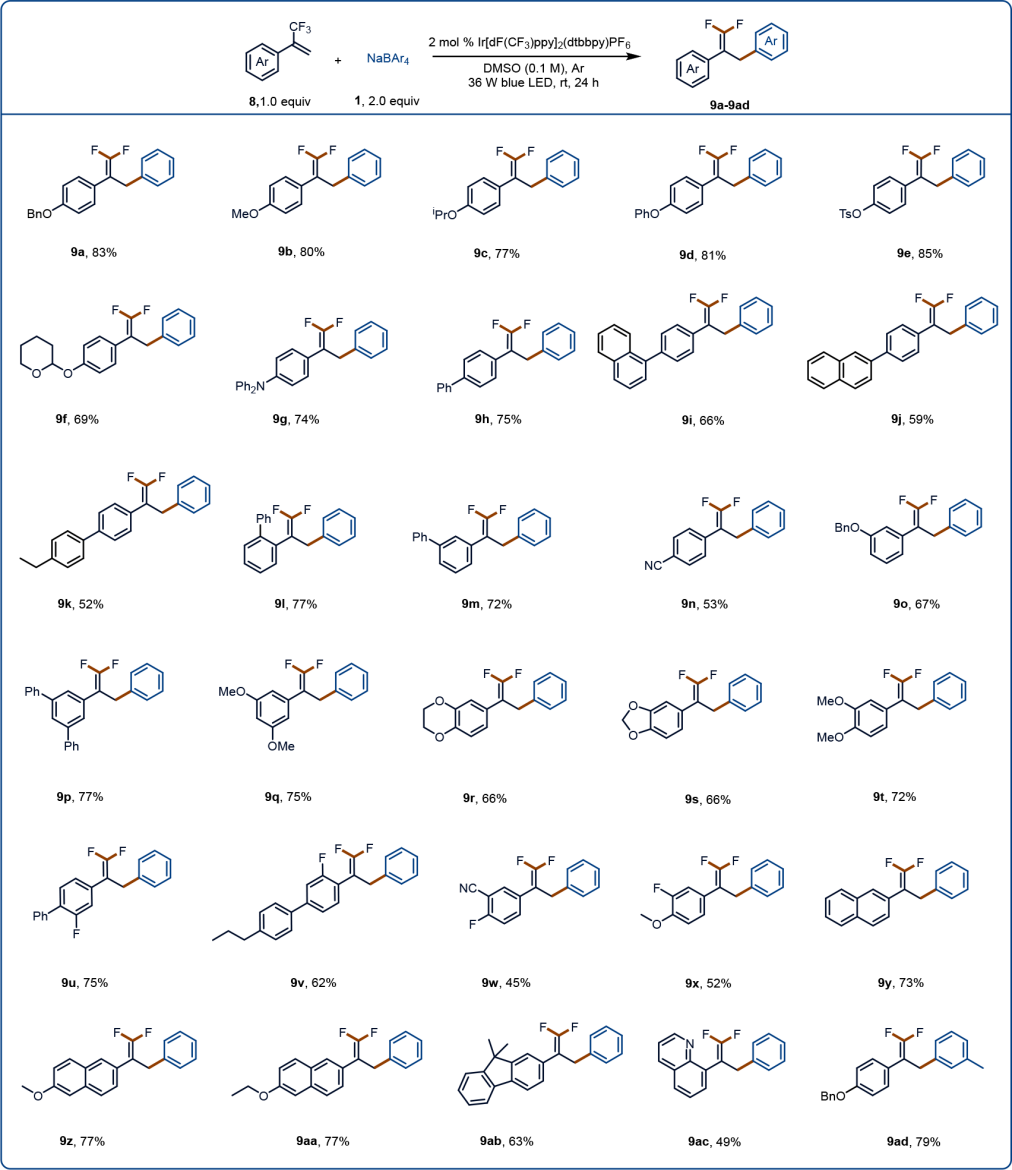
Substrate scope of the defluorinative arylation reaction. Reaction conditions: **1** (0.4 mmol, 2.0 equiv), **8** (0.2 mmol, 1.0 equiv), Ir[dF(CF_3_)ppy]_2_(dtbbpy)PF_6_ (2 mol %), DMSO (2.0 mL), Ar, 36 W blue LED, rt, 24 h.

Because fluorinated groups are frequently incorporated into organic molecules to impart desirable pharmacological properties such as increased metabolic stability, enhanced lipophilicity, and improved bioavailability, the development of methods for the synthesis of new and unusual fluorinated groups is of increasing interest to chemists. Therefore, in a further demonstration of the generality of our method, we used it to accomplish intramolecular radical polarity cross-elimination reactions, namely, defluorinative alkylation and allylation.^55−59^ We were pleased to find that when α-trifluoromethyl aryl alkenes **8** and sodium tetraphenylborate **1a** in DMSO containing Ir[dF(CF_3_)ppy]_2_(dtbbpy)PF_6_ were irradiated with a blue LED at rt, E1cb-type fluoride elimination prevailed over protonation and yielded desired *gem*-difluoroalkene products **9** (Figure 4). Specifically, α-trifluoromethyl aryl alkenes with an electron-donating group at the para position (alkoxy, arylamino, or phenyl) gave **9a**–**9h** in moderate to good yields. In addition, *para*-naphthyl-substituted compounds were suitable substrates (**9i** and **9j**), and a 4-(4-ethylphenyl)-substituted compound gave **9k**, albeit in a relatively low yield. The position of a phenyl group on the aromatic ring of the aryl alkene had little effect on the yield (compare **9h**, **9l**, and **9m**). The yield was relatively low for an aryl alkene with an electron-withdrawing cyano group (**9n**). Products with disubstituted aromatic rings (**9p**–**9x**) were obtained in 45–77% yields. Substrates containing naphthalene (**9y**–**9aa**), fluorene (**9ab**), and quinoline (**9ac**) rings, which are useful for further synthetic manipulations, were well tolerated. Sodium tetra(*p*-tolyl)borate was also tested and found to give desired product **9ad**.

**Figure 5:**
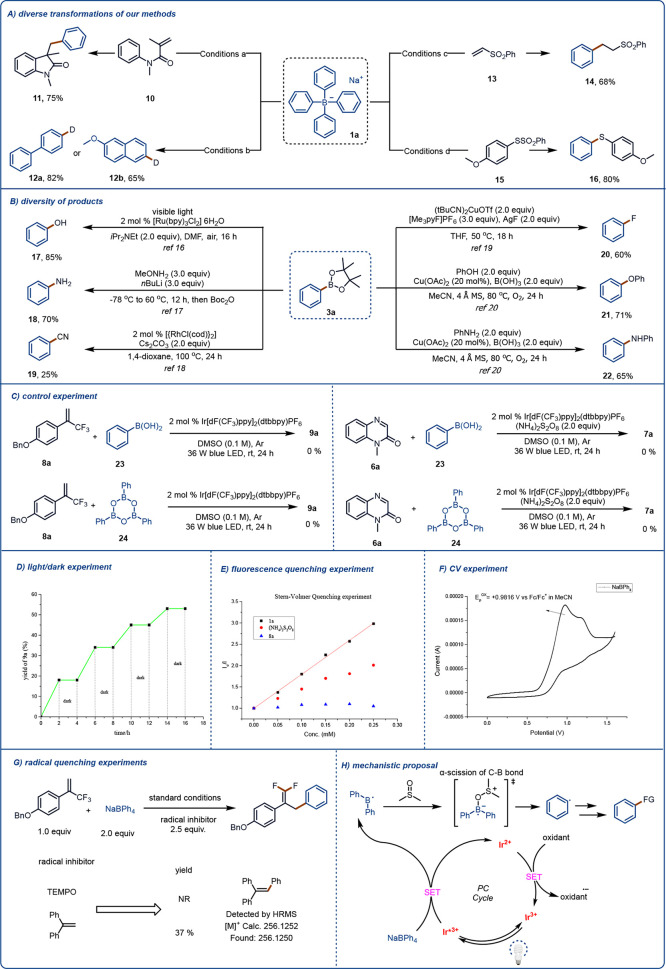
(A) Utility of sodium tetraarylborates for a diverse array of transformations. Conditions a: **1a** (0.4 mmol, 2.0 equiv), **10** (0.2 mmol, 1.0 equiv), (NH_4_)_2_S_2_O_8_ (0.4 mmol, 2.0 equiv), Ir[dF(CF_3_)ppy]_2_(dtbbpy)PF_6_ (2 mol %), DMSO (2.0 mL), Ar, 36 W blue LED, rt, 24 h. Conditions b: **1a** (0.4 mmol, 2.0 equiv), *tert*-butylthiol (20 mol %), Ir[dF(CF_3_)ppy]_2_(dtbbpy)PF_6_ (2 mol %), 5:1 (v/v) *d*_6_-DMSO/D_2_O (2.0 mL), Ar, 36 W blue LED, rt, 24 h. Conditions c: **1a** (0.4 mmol, 2.0 equiv), **13** (0.2 mmol, 1.0 equiv), Ir[dF(CF_3_)ppy]_2_(dtbbpy)PF_6_ (2 mol %), DMSO (2.0 mL), Ar, 36 W blue LED, rt, 24 h. Conditions d: **1a** (0.2 mmol, 2.0 equiv), **15** (0.4 mmol, 2.0 equiv), Ir[dF(CF_3_)ppy]_2_(dtbbpy)PF_6_ (2 mol %), DMSO (2.0 mL), Ar, 36 W blue LED, rt, 24 h. (B) Transformations of product **3a** (0.4 mmol). (C) Control experiments. (D) Light/dark experiment. (E) Fluorescence quenching experiment. (**F**) Cyclic voltammetry experiment. (**G**) Radical quenching experiments. (H) Proposed mechanism.

In addition to the reactions described above, photocatalytic reactions of sodium tetraarylborates with several other coupling partners could also be carried out at ambient temperature. For example, by using a catalytic amount of Ir[(dF(CF_3_)_2_ppy)_2_dtbpy]PF_6_ and irradiation with visible light, we could obtain tandem cyclization product **11** from the reaction of sodium tetraphenylborate (**1a**) and **10** in the presence of (NH_4_)_2_S_2_O_8_ as an oxidizing agent. By using the same photocatalyst, along with *tert*-butylthiol as a hydrogen-atom-transfer reagent, we achieved deuteration of this method. Alternatively, **1a** could be used for a Giese radical addition reaction with **13** to afford **14**, as well as for a direct deboronization sulfide reaction with **15** to afford **16** (Figure 5A). Moreover, phenylboronic ester **3a** could be efficiently synthesized on a gram scale from B_2_pin_2_ and **1a**, and the C–B bond of **3a** could be transformed into various C–O, C–N, C–F, and C–CN bonds by means of previously described methods (**17**–**22**, Figure 5B).^60−64^

We then performed several experiments to gain insight into the mechanism of reaction. According to Xia’s report,^7^**23** and **24** can be produced by oxidation of sodium tetraphenylborate (**1a**) under photocatalytic conditions (Figure 5C). Therefore, we used these two compounds instead of **1a** to react with radical receptor **8a** or **6a** under otherwise standard conditions. However, none of the desired product (**9a** or **7a**, respectively) was detected, so we concluded that neither **23** nor **24** was the source of the aryl radicals. Next, we carried out a light/dark experiment, which showed that the reaction of **8a** and **1a** stopped when there was no light (Figure 5D). This result suggests that any chain propagation process was transient and that light was essential for product formation. We then performed UV–vis spectroscopy and fluorescence quenching experiments and prepared Stern–Volmer diagrams (Figure 5E). The UV–vis spectra confirmed that the photocatalyst was quenched by **1a**. Electrochemical analysis of **1a** showed that **1a** had a low oxidation peak that completely quenched the photocatalyst (Figure 5F). Finally, we found that the reaction was stopped by free-radical scavengers, and we detected a free-radical-trapping product by means of high-resolution mass spectrometry (Figure 5G). This experiment clearly shows that the reaction proceeded via a free-radical pathway.

On the basis of literature reports and the results of our mechanistic experiments, we propose the reaction mechanism shown in Figure 5H. The excited-state photocatalyst is quenched by **1a** to produce a diarylboron radical, which then forms a complex with DMSO. The complex undergoes α-scission to produce an aryl radical, which is subsequently captured by a radical acceptor, resulting in aryl functionalization of the acceptor.

## Conclusion

In conclusion, we have described the use of tetraarylborate salts as new precursors for aryl radicals, which are generated upon irradiation of the salts with visible light in the presence of DMSO as an activation reagent. Our findings also offer new reaction pathways and applications for tetraarylborate salts. We used the radical precursors to accomplish various transformations. By addition of DMSO, the initially generated diarylboron radicals could be made to produce aryl radicals, which in turn enabled the formation of C–B, C–C, and C–X bonds. The extension of tetraarylborate salts to other challenging and useful transformations is currently being explored in our laboratory.

## Supporting Information Available

Experimental procedures and spectroscopic data for all new compounds (PDF)
