# Supplementary material for: Formation of C–B, C–C, and C–X Bonds from Nonstabilized Aryl Radicals Generated from Diaryl Boryl Radicals
Source: ACS Cent Sci. 2023 Nov 13;9(12):2268–76. doi: 10.1021/acscentsci.3c00993 (PMC10755731; doi:10.1021/acscentsci.3c00993)

Formation of C–B, C–C, and C–X bonds from nonstabilized aryl radicals generated from diaryl boryl radicals

Fuyang Yue, Henan Ma, Pengxuan Ding, Hongjian Song, Yuxiu Liu and Qingmin Wang*

State Key Laboratory of Elemento-Organic Chemistry, Research Institute of Elemento-Organic Chemistry, Frontiers Science Center for New Organic Matter, College of Chemistry, Nankai University, Tianjin 300071, People's Republic of China

*Correspondence to wangqm@nankai.edu.cn

Supporting Information

Table of Contents……………………….……………………………………………S1
General Information…………………………………………………………………….S2
Preparation of Photocatalyst ………………………………...……………………S3
General procedure for the preparation of sodium tetraarylborate……….…………S3
General procedure for the radical acceptor……………………………………S3–S4
Investigation of the Key Reaction Parameters………………………...………S4–S6
Investigation of the Mechamism……………………………………………..S6–S10
Experimental Procedures and Product Characterization………………………S11–S51
References………………………………..………………………….……...... S52
Copies of ^{1}H NMR ,^{13}C NMR and ^{19}F NMR spectra for new compounds….S51–S180

1. General Information

Reagents were purchased from commercial sources and were used as received. ^{1}H and ^{13}C ^{19}F ^{11}B Nuclear Magnetic Resonance (NMR) spectra were recorded on Bruker Avance 400 Ultrashield NMR spectrometers. Chemical shifts (δ) were given in parts per million (ppm) and were measured downfield from internal tetramethylsilane. High-resolution mass spectrometry (HRMS) data were obtained on an FTICR-MS instrument (Ionspec 7.0 T). The melting points were determined on an X-4 microscope melting point apparatus and are uncorrected. Conversion was monitored by thin layer chromatography (TLC). Flash column chromatography was performed over silica gel (100-200 mesh). Blue LED (36 W, λ_{max} = 470 nm) purchased from JIADENG (LS) was used for blue light irradiation. A fan attached to the apparatus was used to maintain the reaction temperature at room temperature.

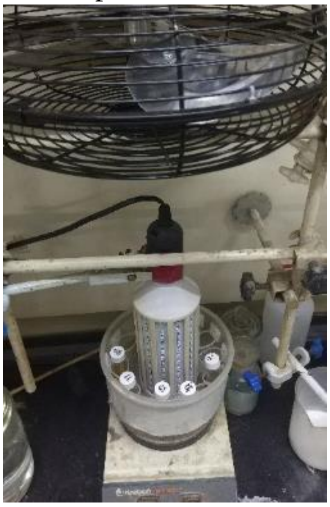

Figure S1 Photograph of the Photocatalytic reactor used for reactions conducted under blue LED irradiation.

2. Preparation of Photocatalyst

The photocatalyst was synthesized according to literature report.[1] The spectral data of the photocatalyst is consistent with the literature data. The other photocatalysts (Eosin Y, Fluorescein, 4CzIPN, $[Ru(bpy)_3]Cl_2{\cdot}6H_2O$, $Ru(bpy)_3(PF_6)_2$, $Ir(ppy)_3$) are commercially available.

3. General procedure for the preparation of sodium tetraarylborate.

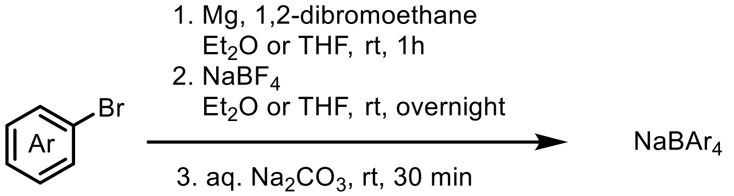

In analogy to a procedure of *Hayashi* et al.,[2] a few drops of 1,2-dibromoethane were added to a suspension of Mg turnings (6.6 or 5.5 equiv.) in THF or Et_2O without stirring. A solution of arylbromide (5.0 or 5.5 equiv.) in THF and/or Et_2O was added slowly. After the start of the reaction (in some cases heating the reaction mixture with a heatgut war necessary) stirring of the solution was started and the arylbromide solution was added dropwise over 60 minutes. The resulting mixture was further stirred for 1 h at room temperature. Sodium tetrafluoroborate (1.0 equiv.) was added and the mixture was stirred for 24 h before pouring it into a solution of Na_2CO_3 in water and stirring for 30 minutes. After filtration the filtrate was extracted with Et_2O, dried over Na_2SO_4 and concentrated in vacuo. The resulting solid was washed with pentane/DCM and dried under vacuum to afford the desired sodium tetraarylborate as a white solid.

4. General procedure for the radical acceptor

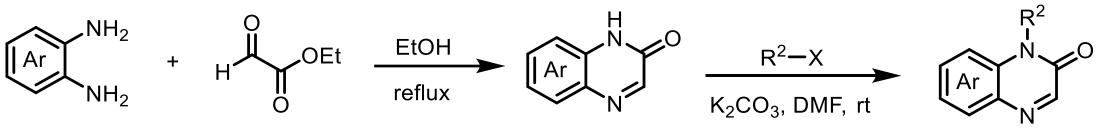

Quinoxalin-2(1H)-one was prepared from 1,2-phenylenediamines following the procedure of *Cui* and co-workers[3] on 5 mmol scale. To a solution of 1,2-phenylenediamines (5 mmol, 1.0 equiv.) in ethanol (40 mL) was added ethyl glyoxalate (6 mmol, 1.2 equiv.). The resultant reaction mixture was stirred at reflux until the raw material disappears. Then, the mixture was filtered and washed by ethanol. The solid was dried in vacuo. For alkylation, the corresponding halogenoalkane (1.6 equiv.) was added to a suspension of quinoxalinone (1.0 equiv.) and potassium carbonate (1.2 equiv.) in DMF (16 mL). The mixture was stirred at room temperature overnight. After complete reaction, brine was added, and then extracted three times with EtOAc. The combined organic layers were washed with a saturated solution of NH_4Cl then brine, dried over anhydrous Na_2SO_4, filtered and evaporated in vacuo. The residue was purified by column chromatography on silica gel to afford the desired product.

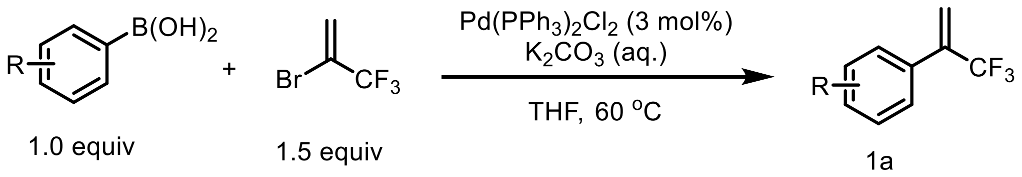

According to literature reports.[4] A Schlenk tube equipped with stir bar, arylboronic acid (1.0 equiv, 3 mmol) and $Pd(PPh_3)_2Cl_2$ (3 mol%, 0.09 mmol, 63.2 mg) were added. The vessel was evacuated and filled with argon (three times), and then aqueous K_2CO_3 (2.0 M, 6 mL) and THF (9 mL) were added. After addition of 2-bromo-3,3,3-trifluoro-1-propene (1.5 equiv, 4.5 mmol, 0.47 mL), the solution was stirred at 60 ^{o}C with heating mantle for 12 hours (TLC tracking detection). The solvent was removed under reduced pressure and the residue was purified by column chromatography to afford the corresponding trifluoromethyl alkene (PE/EA).

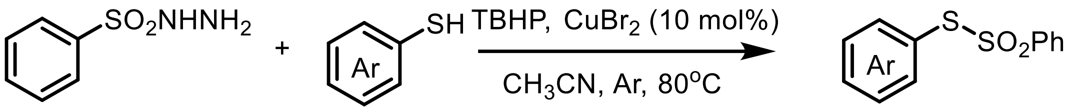

According to the reported procedure,[4] To a acetonitrile (25 mL) solution of sulfonylhydrazide (5 mmol), thiol (10 mmol) and CuBr2 (0.5 mmol) in Schlenk tube equipped a magnetic bar was added tert-butyl hydroperoxide (TBHP, 37.5 mmol) dropwise over a minute. The reaction mixture was stirred at 80 °C for 5 h in air. After the substrates were completely consumed, the solvent was evaporated off and the residue was purified by flash column chromatography (silica gel, petroleum ether/EtOAc = 40:1) to afford the thiosulfonates.

5. Investigation of the Key Reaction Parameters

Table S1: Screening of different activation reagents[a]

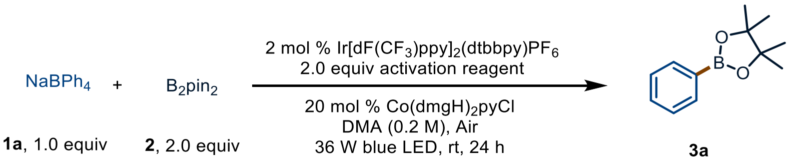

entry	activation reagent	yield (%)[b]
1	methanol	NR
2	phenol	NR
3	NMP	NR
4	dimethyl sulfoxide	19
5	ethanol	NR
6	cyclohexanol	NR
7	benzoic acid	NR
8	DMF	NR
12	benzhydrol	NR

aGeneral conditions: **1a** (0.4 mmol), **2** (0.8 mmol), PC (0.008 mmol), activation reagent (2.0 equiv), oxidation reagent (20 mol%), solvent (2.0 mL), rt, Air atmosphere, 24 h. bIsolated yields are provided, NR = no reaction.

Table S2: Screening of different oxidation reagent[a]

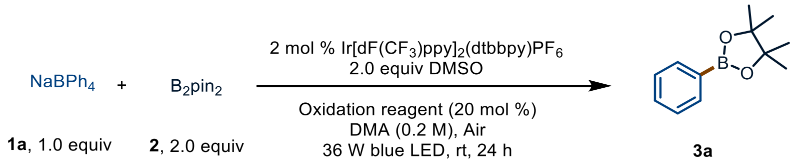

entry	oxidation reagent	yield (%)[b]
1	$Co(dmgH)_2pyCl$	19
2	$Co(dmgH)_2Cl_2$	trace
3	$Co(dmgH)_2(4\text{-}CO_2Et)PyCl$	16
4	$Co(dmgH)_2(4\text{-}tBu)PyCl$	trace
5	$Co(dmgH)_2(4\text{-}diMeN)PyCl$	NR
6	$Co(dmgH)_2(4\text{-}CN)PyCl$	NR
7	$Co(dmgH)_2(4\text{-}Br)pyCl$	17
8	$Co(dmgH)_2(3\text{-}Br)pyCl$	20
9	$Co(dmgH)_2(4\text{-}MeO)PyCl$	trace
10[c]	$(NH_4)_2S_2O_8$	32
11[c]	$Na_2S_2O_8$	17
12[c]	$K_2S_2O_8$	30
13	O_2	15

aGeneral conditions: **1a** (0.4 mmol), **2** (0.8 mmol), PC (0.008 mmol), activation reagent (2.0 equiv), oxidation reagent (20 mol%), solvent (2.0 mL), rt, Air atmosphere, 24 h. bIsolated yields are provided, NR = no reaction. cOxidation reagent (2.0 equiv)

Table S3: Screening of photocatalysts[a]

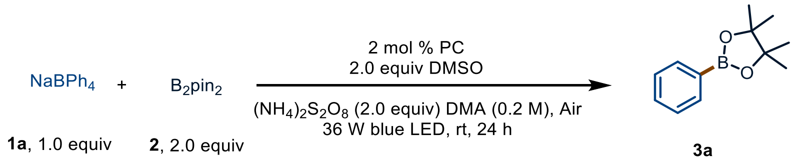

entry	photocatalyst	yield (%)[b]
1	$[Ir(dtbbpy)(ppy)_2][PF_6]$	trace
2	$Ir(ppy)_3$	NR
3	$[Ru(bpy)_3](PF_6)_2$	NR
4	$[Ru(bpy)_3]\ 6H_2O$	NR
5[c]	4CzIPN	25
6[c]	Eosin-Y	NR
7[c]	Mes-Acr	NR
8[c]	Fluorescein	NR

9	Ir[dF(CF_3)ppy]$_2$(dtbbpy)PF_6	32

[a]General conditions: **1a** (0.4 mmol), **2** (0.8 mmol), PC (0.008 mmol), activation reagent (2.0 equiv), oxidation reagent (2.0 equiv), solvent (2.0 mL), rt, Air atmosphere, 24 h. [b]Isolated yields are provided, NR = no reaction. [c]Photocatalyst (0.010 mmol).

Table S4: Screening of the amount of activation reagents[a]

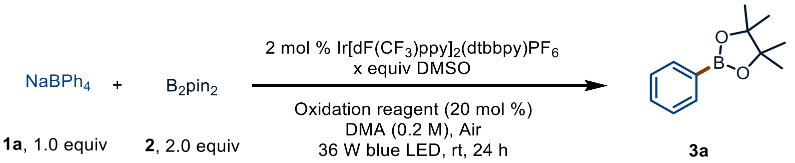

entry	x eq.	yield (%)[b]
1	0.0	NR
2	1.0	30
3	2.0	32
4	3.0	41
5	4.0	47
6[c]	as solvent	75

[a]General conditions: **1a** (0.4 mmol), **2** (0.8 mmol), PC (0.008 mmol), activation reagent (x equiv), oxidation reagent (2.0 equiv), solvent (2.0 mL), rt, Air atmosphere, 24 h. [b]Isolated yields are provided, NR = no reaction. [c]MSO/DMA = 5/1 (2 mL)

6. Investigation of the Mechamism

6.1 Control experiments

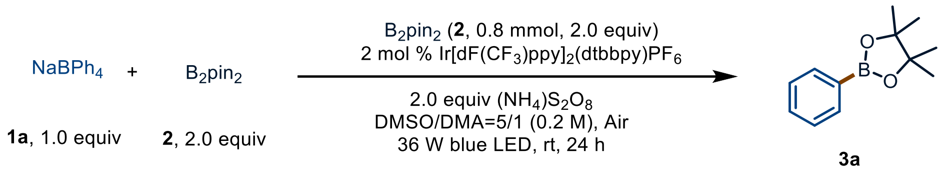

entry	control conditions	yield (%)
1	w/o photocatalyst	NR
2	w/o light	NR
3	w/o DMSO	NR
4	Ar	65
5	standard conditions, w/all	75

Yields were determined by ^{19}F NMR spectroscopy with fluorobenzene as an internal standard. NR = no reaction.

6.2 Emission Quenching Experiments (Stern–Volmer Studies)

Emission intensities were recorded using a CARY VARIAN luminescence spectrophotometer. All Ir[dF(CF_3)ppy]$_2$(dtbbpy)PF_6 solutions were excited at 350 nm and the emission intensity was collected at 470 nm. In a typical experiment, to a 3 ×

10^{-6} M solution of Ir[dF(CF_3)ppy]$_2$(dtbbpy)PF_6 in DMSO/DMA=5/1 was added the appropriate amount of a quencher in a screw-top quartz cuvette. After degassing the sample with a stream of argon for 10 minutes, the emission of the sample was collected.

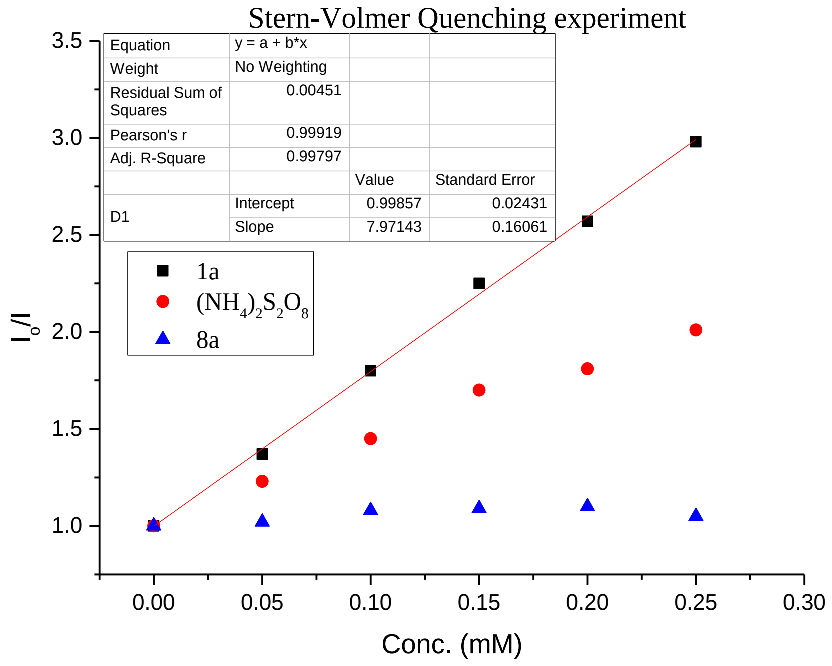

6.3 Light/dark experiment

Eight standard reaction mixtures in 10 mL glass vials were charged with **8a** (0.2 mmol), **1a** (0.4 mmol), PC (0.004 mmol), in DMSO (2.0 mL). The reaction mixtures were degassed by bubbling with Ar for 15 s with an outlet needle and the vials were sealed with PTFE caps. The mixtures were then stirred rapidly and irradiated with a 36 W Blue LED (approximately 2 cm away from the light source) at room temperature. After 2 h, the Blue LED was turned off, and one vial was removed from the irradiation setup for analysis. The remaining seven vials were stirred in the absence of light for an additional 2 h. Then, one vial was removed for analysis, and the Blue LED was turned back on to irradiate the remaining six reaction mixtures. After an additional 2 h of irradiation, the Blue LED was turned off, and one vial was removed for analysis. The remaining five vials were stirred in the absence of light for an additional 2 h. Then, a vial was removed for analysis, and the Blue LED was turned back on to irradiate the remaining four reaction mixtures. After 2 h, the Blue LED was turned off, and one vial was removed for analysis. The remaining three vials were stirred in the absence of light for an additional 2 h, then, a vial was removed for analysis and the Blue LED was turned back on to irradiate the remaining two reaction mixtures. After 2 h, the Blue LED was turned off, and one vial was removed for analysis. The last vial was stirred in the absence of light for an additional 2 h, and then it was analyzed. The yield was determined by ^{19}F NMR spectroscopy using Fluorobenzene as the internal standard.

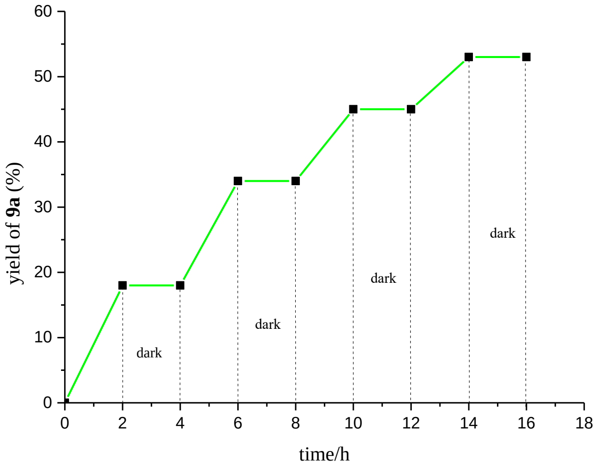

Light/dark experiment.

6.4 Cyclic voltammetry measurements

The experiments were conducted using a cyclic potentiometer with a glassy carbon working electrode, a Pt counter electrode and an Ag/AgCl reference electrode [referenced to SCE using ferrocene (Fc) as an internal standard (0.42 V vs. SCE)].20 In the standard procedure, 0.02 mmol of substrate were dissolved in 10 mL of a 0.1 M $[N(Bu)_4]PF_6$ electrolyte solution in degassed MeCN. The reactor was sealed with a rubber septum and purged with nitrogen. Each measurement was conducted at 100 mV/s at room temperature under nitrogen atmosphere without stirring.

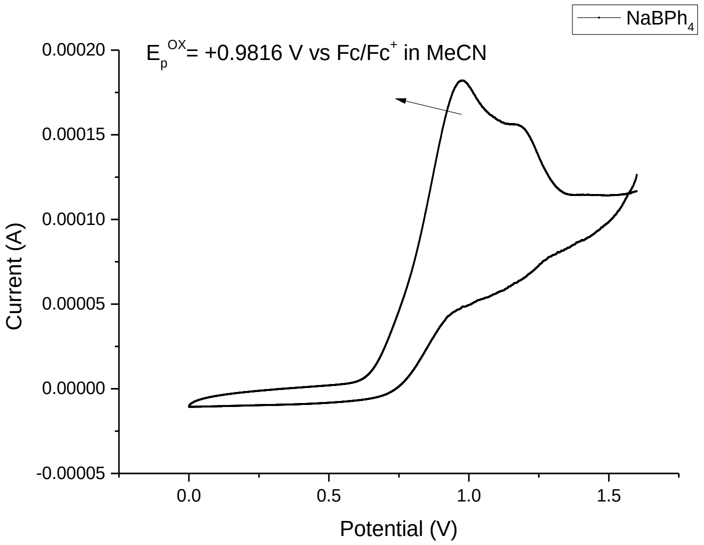

cyclic voltammetry measurements

6.5 TEMPO and 1,1-diphenylethylene were used as radical scavengers

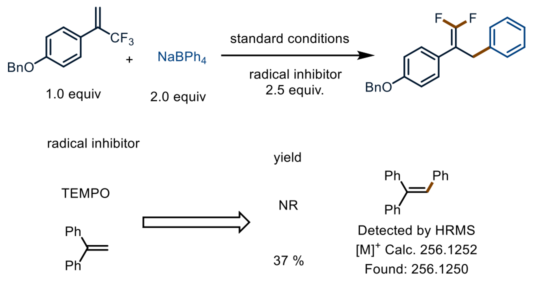

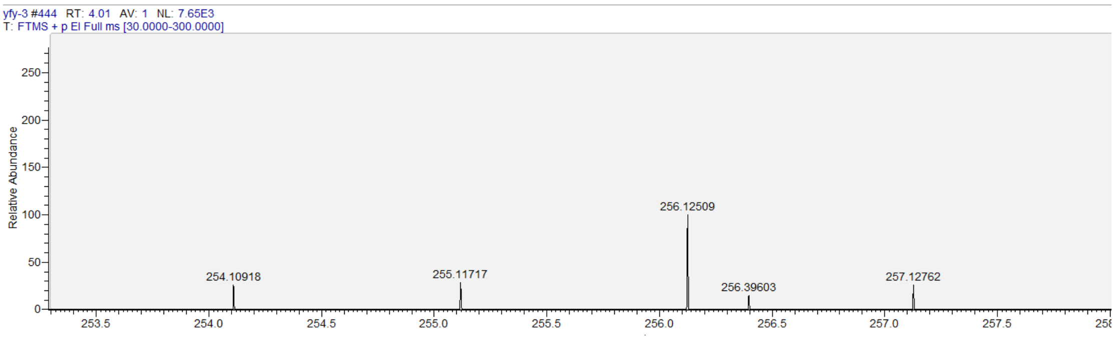

Scheme S1

To a 10 mL glass vial was added Ir[dF(CF_3)ppy]$_2$(dtbbpy)PF_6 (4.48 mg, 0.002 mmol, 2 mol %), 1-(benzyloxy)-4-(3,3,3-trifluoroprop-1-en-2-yl)benzene (0.2 mmol, 1.0 equiv), $NaBPh_4$ (0.4 mmol, 2.0 equiv), TEMPO (117 mg, 0.75 mmol, 2.5 equiv), or 1,1-diphenylethylene (135 mg, 0.75 mmol, 2.5 equiv), and 2.0 mL of DMSO. The reaction mixture was degassed by bubbling with Ar for 15 s with an outlet needle and the vial was sealed with PTFE cap. The mixture was then stirred rapidly and irradiated with a 36 W Blue LED (approximately 2 cm away from the light source) at room temperature for 24 h. The yields of corresponding alkylated product **3** were determined by ^{19}F NMR spectroscopy with fluorobenzene as an internal standard.

6.6 Generation of by-products

Through boron spectrum experiments and GCMS experiments, compound **24** was found in the reaction system. Therefore, we speculate that boron atoms will ultimately exist as compound **24** in the system. However, the control experiment Fig5C shows that the source of aromatic radicals does not come from compound 24. This further confirms the possibility of our mechanism.

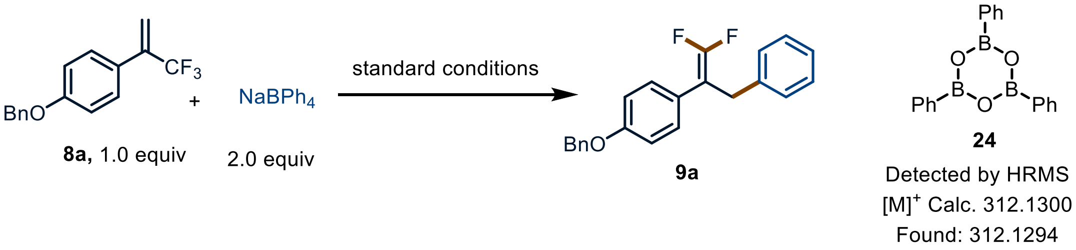

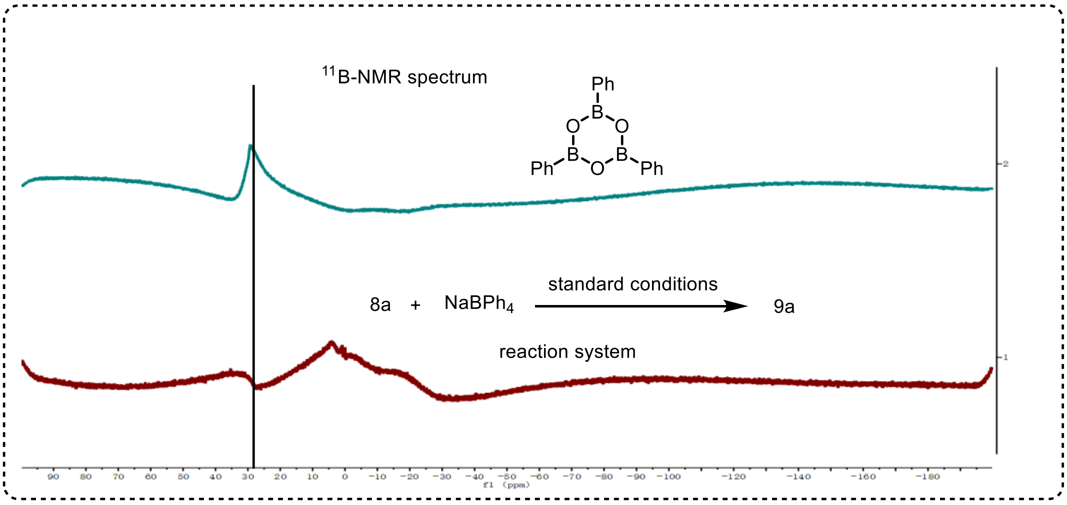

^{11}B-NMR spectrum
Ph
B
O
O
Ph
B
O
B
Ph
8a + NaBPh$_4$
standard conditions
9a
reaction system
f1 (ppm)

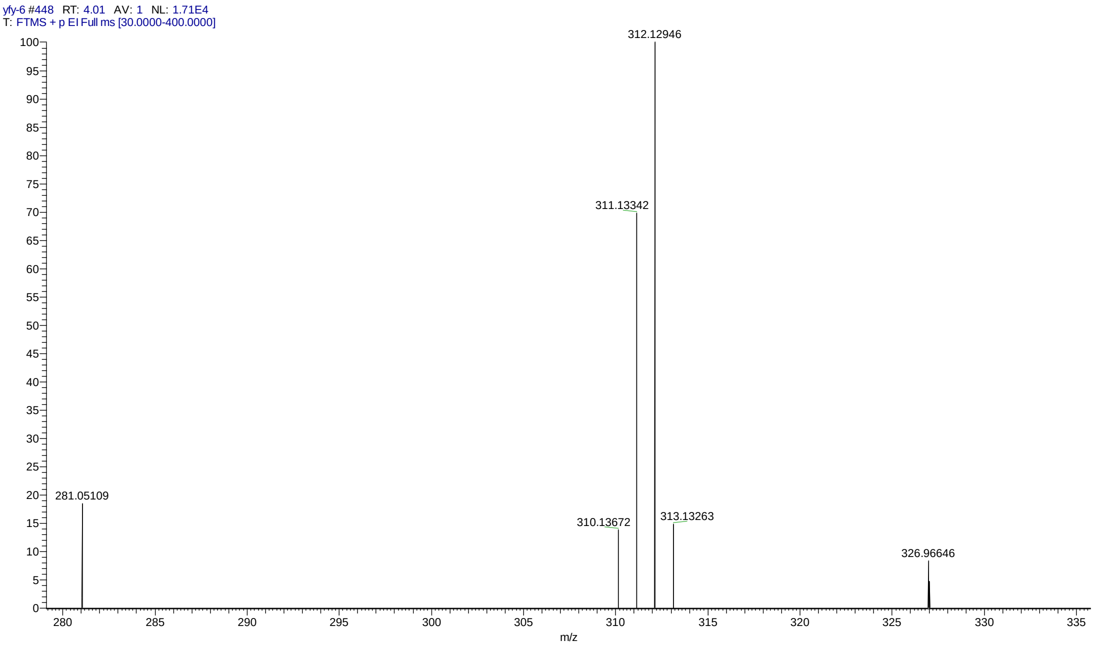

yfy-6 #448 RT: 4.01 AV: 1 NL: 1.71E4
T: FTMS + p EI Full ms [30.0000-400.0000]
312.12946
311.13342
281.05109
310.13672
313.13263
326.96646
m/z

7. Experimental Procedures and Product Characterization

7.1 General Procedure A:

To a 10 mL glass vial was added Ir[dF(CF_3)ppy)]$_2$(dtbbpy)PF_6 (8.96 mg, 0.008 mmol, 2 mol %), $NaBPh_4$ (0.4 mmol, 1.0 equiv), B_2Pin_2 (0.8 mmol, 2.0 equiv), $(NH_4)_2S_2O_8$ (0.8 mmol, 2.0 equiv) and 2.0 mL of solvent, and the vial was sealed with PTFE cap. The mixture was then stirred rapidly and irradiated with a 36 W Blue LED (approximately 2 cm away from the light source) at room temperature for 24 h. The reaction mixture was diluted with 10 mL of H_2O, and extracted with DCM (3 × 20 mL). The combined organic extracts were washed with brine (40 mL), dried over Na_2SO_4, and concentrated in vacuo. Purification of the crude product by flash chromatography on silica gel using the indicated solvent system afforded the desired product.

General Procedure B:

To a 10 mL glass vial was added Ir[dF(CF_3)ppy)]$_2$(dtbbpy)PF_6 (8.96 mg, 0.008 mmol, 2 mol %), $NaBPh_4$ (0.4 mmol, 1.0 equiv), $Na_2S_2O_5$ (0.8 mmol, 2.0 equiv), and 2.0 mL of solvent. The reaction mixture was degassed by bubbling with Ar for 15 s with an outlet needle and the vial was sealed with PTFE cap. The mixture was then stirred rapidly and irradiated with a 36 W Blue LED (approximately 2 cm away from the light source) at room temperature for 24 h. Then, sodium bicarbonate (1.5 equiv.) was added followed by EtOH (1 mL) and alkyl bromide (1.2 equiv.). The reaction mixture was stirred at room temperature for 16 hours. The reaction mixture was diluted with 10 mL of H_2O, and extracted with DCM (3 × 20 mL). The combined organic extracts were washed with brine (40 mL), dried over Na_2SO_4, and concentrated in vacuo. Purification of the crude product by flash chromatography on silica gel using the indicated solvent system afforded the desired product.

General Procedure C:

To a 10 mL glass vial was added Ir[dF(CF_3)ppy)]$_2$(dtbbpy)PF_6 (8.96 mg, 0.008 mmol, 2 mol %), $NaBPh_4$ (0.4 mmol, 1.0 equiv), $Na_2S_2O_5$ (0.8 mmol, 2.0 equiv), and 2.0 mL of solvent. The reaction mixture was degassed by bubbling with Ar for 15 s with an outlet needle and the vial was sealed with PTFE cap. The mixture was then stirred rapidly and irradiated with a 36 W Blue LED (approximately 2 cm away from the light source) at room temperature for 24 h. Then, sodium acetate (2 equiv.) was added followed by hydroxylamine-O-sulfonic acid (2 equiv.), and the reaction mixture was stirred at room temperature for 24 hours. The reaction mixture was diluted with 10 mL of H_2O, and extracted with DCM (3 × 20 mL). The combined organic extracts were washed with brine (40 mL), dried over Na_2SO_4, and concentrated in vacuo. Purification of the crude product by flash chromatography on silica gel using the indicated solvent system afforded the desired product.

General Procedure D:

To a 10 mL glass vial was added Ir[dF(CF_3)ppy)]$_2$(dtbbpy)PF_6 (8.96 mg, 0.008 mmol, 2 mol %), $NaBPh_4$ (0.4 mmol, 1.0 equiv), $Na_2S_2O_5$ (0.8 mmol, 2.0 equiv), and 2.0 mL of solvent. The reaction mixture was degassed by bubbling with Ar for 15 s with an

outlet needle and the vial was sealed with PTFE cap. The mixture was then stirred rapidly and irradiated with a 36 W Blue LED (approximately 2 cm away from the light source) at room temperature for 24 h. Then, sodium bicarbonate (2.0 equiv.) was added followed by N-chlorosuccinimide (1.0 equiv.) at room temperature. The reaction mixture was stirred at room temperature for 30 minutes. The reaction mixture was diluted with 10 mL of H2O, and extracted with DCM (3 × 20 mL). The combined organic extracts were washed with brine (40 mL), dried over Na_2SO_4, and concentrated in vacuo. Purification of the crude product by flash chromatography on silica gel using the indicated solvent system afforded the desired product.

General Procedure E:

To a 10 mL glass vial was added $Ir[dF(CF_3)ppy)]_2(dtbbpy)PF_6$ (8.96 mg, 0.008 mmol, 2 mol %), $NaBPh_4$ (0.4 mmol, 1.0 equiv), $Na_2S_2O_5$ (0.8 mmol, 2.0 equiv), and 2.0 mL of solvent. The reaction mixture was degassed by bubbling with Ar for 15 s with an outlet needle and the vial was sealed with PTFE cap. The mixture was then stirred rapidly and irradiated with a 36 W Blue LED (approximately 2 cm away from the light source) at room temperature for 24 h. Then, the reaction mixture was cooled to 0 ^{o}C in an ice bath, and sodium bicarbonate (1.5 equiv.) was added followed by N-chlorosuccinimide (1.2 equiv.). The reaction mixture was stirred at 0 ºC for 15 minutes, then transferred to a separatory funnel, diluted with brine (20 mL) and extracted with dichloromethane (3 x 20 mL). The combined organic extracts were dried over Na2SO4, filtered, and concentrated in vacuo. The residue was dissolved in THF (1 mL) and nucleophilic reagent (2 equiv.) was added in a single portion at room temperature. The reaction mixture was stirred at room temperature for 15 minutes. The reaction mixture was diluted with 10 mL of H2O, and extracted with DCM (3 × 20 mL). The combined organic extracts were washed with brine (40 mL), dried over Na_2SO_4, and concentrated in vacuo. Purification of the crude product by flash chromatography on silica gel using the indicated solvent system afforded the desired product.

General Procedure F:

To a 10 mL glass vial was added $Ir[dF(CF_3)ppy)]_2(dtbbpy)PF_6$ (4.48 mg, 0.004 mmol, 2 mol %), $NaBPh_4$ (0.4 mmol, 2.0 equiv), **6** (0.2 mmol, 1.0 equiv), $(NH_4)_2S_2O_8$ (0.4 mmol, 2.0 equiv) and 2.0 mL of solvent. The reaction mixture was degassed by bubbling with Ar for 15 s with an outlet needle and the vial was sealed with PTFE cap.. The mixture was then stirred rapidly and irradiated with a 36 W Blue LED (approximately 2 cm away from the light source) at room temperature for 24 h. The reaction mixture was diluted with 10 mL of H_2O, and extracted with DCM (3 × 20 mL). The combined organic extracts were washed with brine (40 mL), dried over Na_2SO_4, and concentrated in vacuo. Purification of the crude product by flash chromatography on silica gel using the indicated solvent system afforded the desired product.

General Procedure G:

To a 10 mL glass vial was added Ir[dF(CF_3)ppy)]$_2$(dtbbpy)PF_6 (4.48 mg, 0.004 mmol, 2 mol %), $NaBPh_4$ (0.4 mmol, 2.0 equiv), **8** (0.2 mmol, 1.0 equiv) and 2.0 mL of solvent. The reaction mixture was degassed by bubbling with Ar for 15 s with an outlet needle and the vial was sealed with PTFE cap. The mixture was then stirred rapidly and irradiated with a 36 W Blue LED (approximately 2 cm away from the light source) at room temperature for 24 h. The reaction mixture was diluted with 10 mL of H_2O, and extracted with DCM (3 × 20 mL). The combined organic extracts were washed with brine (40 mL), dried over Na_2SO_4, and concentrated in vacuo. Purification of the crude product by flash chromatography on silica gel using the indicated solvent system afforded the desired product.

7.2. Product Characterization

4,4,5,5-tetramethyl-2-phenyl-1,3,2-dioxaborolane (3a).

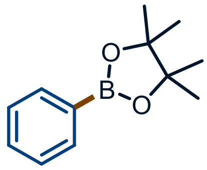

According to the *general procedure A*. The spectral data is consistent with the literature data.[5]

Colorless oil (61.2 mg, 75%).

R_f 0.70 (Petroleum ether/EtOAc, 40/1).

^{1}H NMR (400 MHz, $CDCl_3$) δ 7.69 (d, J = 7.2 Hz, 2H), 7.32 (d, J = 7.4 Hz, 1H), 7.24 (t, J = 7.4 Hz, 2H), 1.22 (s, 12H). **^{13}C NMR** (100 MHz, $CDCl_3$) δ 134.79, 131.28, 127.74, 83.78, 24.91.

^{11}B NMR (128 MHz, $CDCl_3$) δ 31.14.

4,4,5,5-tetramethyl-2-(p-tolyl)-1,3,2-dioxaborolane (3b).

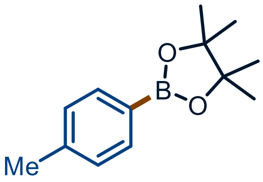

According to the *general procedure A*. The spectral data is consistent with the literature data.[5]

Colorless oil (69.7 mg, 80%).

R_f 0.70 (Petroleum ether/EtOAc, 40/1).

^{1}H NMR (400 MHz, $CDCl_3$) δ ^{1}H NMR (400 MHz, $CDCl_3$) δ 7.75 (d, J = 6.2 Hz, 2H), 7.23 (d, J = 6.4 Hz, 2H), 2.41 (s, 3H), 1.38 (s, 12H). **^{13}C NMR** (100 MHz, $CDCl_3$) δ 141.40, 134.83, 128.53, 83.62, 24.87, 21.74.

^{11}B NMR (128 MHz, $CDCl_3$) δ 31.47.

2-(4-ethylphenyl)-4,4,5,5-tetramethyl-1,3,2-dioxaborolane (3c).

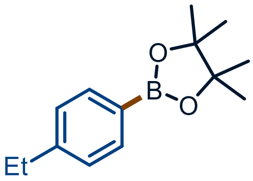

According to the *general procedure A*. The spectral data is consistent with the literature data.[5]

Colorless oil (67.7 mg, 73%).

*R*f 0.70 (Petroleum ether/EtOAc, 40/1).

^{1}H NMR (400 MHz, $CDCl_3$) *δ* 7.78 (d, *J* = 7.7 Hz, 2H), 7.23 (d, *J* = 7.7 Hz, 2H), 2.67 (d, *J* = 7.6 Hz, 2H), 1.35 (s, 12H), 1.26 (t, *J* = 7.6 Hz, 3H). **^{13}C NMR** (100 MHz, $CDCl_3$) *δ* 147.73, 135.03, 127.40, 83.62, 29.20, 24.93, 15.57.

^{11}B NMR (128 MHz, $CDCl_3$) *δ* 31.40.

2-(4-isopropylphenyl)-4,4,5,5-tetramethyl-1,3,2-dioxaborolane (3d).

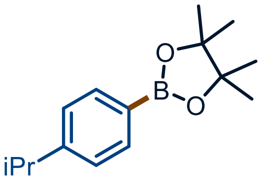

According to the *general procedure A*. The spectral data is consistent with the literature data.[5]

Colorless oil (75.7 mg, 77%).

*R*f 0.70 (Petroleum ether/EtOAc, 40/1).

^{1}H NMR (400 MHz, $CDCl_3$) *δ* 7.76 (d, *J* = 7.9 Hz, 2H), 7.24 (d, *J* = 7.9 Hz, 2H), 2.90 (dt, *J* = 13.8, 6.9 Hz, 1H), 1.32 (s, 12H), 1.24 (d, *J* = 6.9 Hz, 6H). **^{13}C NMR** (100 MHz, $CDCl_3$) *δ* 152.35, 135.02, 125.97, 83.63, 34.41, 24.91, 23.91.

^{11}B NMR (128 MHz, $CDCl_3$) *δ* 31.18.

2-(4-butylphenyl)-4,4,5,5-tetramethyl-1,3,2-dioxaborolane (3e).

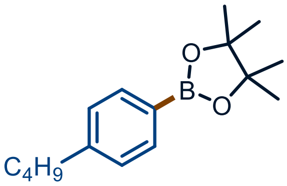

According to the *general procedure A*. The spectral data is consistent with the literature data.[5]

Colorless oil (85.2 mg, 82%).

*R*f 0.60 (Petroleum ether/EtOAc, 40/1).

^{1}H NMR (400 MHz, $CDCl_3$) *δ* 7.73 (d, *J* = 7.6 Hz, 2H), 7.19 (d, *J* = 7.7 Hz, 2H), 2.61 (t, *J* = 7.7 Hz, 2H), 1.65 – 1.53 (m, 2H), 1.33 (t, *J* = 5.0 Hz, 14H), 0.91 (dd, *J* = 8.3, 6.4 Hz, 3H). **^{13}C NMR** (100 MHz, $CDCl_3$) *δ* 146.42, 134.88, 127.96, 83.62, 35.94, 33.56, 24.91, 22.41, 14.00.

^{11}B NMR (128 MHz, $CDCl_3$) δ 31.74.

4,4,5,5-tetramethyl-2-(4-pentylphenyl)-1,3,2-dioxaborolane (3f).

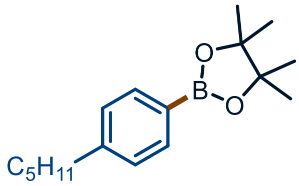

According to the *general procedure A*. The spectral data is consistent with the literature data.[5]
Colorless oil (87.6 mg, 80%).
R_f 0.70 (Petroleum ether/EtOAc, 40/1).
^{1}H NMR (400 MHz, $CDCl_3$) δ ^{1}H NMR (400 MHz, $CDCl_3$) δ 7.79 (d, J = 7.9 Hz, 2H), 7.25 (d, J = 7.9 Hz, 2H), 2.71 – 2.62 (m, 2H), 1.72 – 1.61 (m, 2H), 1.42 – 1.34 (m, 16H), 0.94 (t, J = 6.9 Hz, 3H). **^{13}C NMR** (100 MHz, $CDCl_3$) δ 146.46, 134.86, 127.95, 83.63, 36.20, 31.53, 31.09, 24.90, 22.59, 14.08.
^{11}B NMR (128 MHz, $CDCl_3$) δ 31.90.

2-(4-(tert-butyl)phenyl)-4,4,5,5-tetramethyl-1,3,2-dioxaborolane (3g).

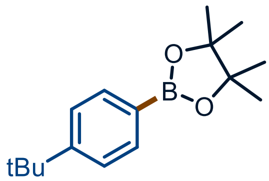

According to the *general procedure A*. The spectral data is consistent with the literature data.[5]
Colorless oil (72.8 mg, 70%).
R_f 0.70 (Petroleum ether/EtOAc, 40/1).
^{1}H NMR (400 MHz, $CDCl_3$) δ ^{1}H NMR (400 MHz, $CDCl_3$) δ 7.60 (d, 2H), 7.24 (d, 2H), 1.16 (s, 21H). **^{13}C NMR** (100 MHz, $CDCl_3$) δ 157.9, 153.5 (t, J = 287.8 Hz), 141.5, 137.3, 136.9, 130.5, 129.4 (t, J = 3.2 Hz), 128.6, 128.0, 127.5, 125.8, 91.5 (dd, J = 18.0, 17.1 Hz), 90.8, 70.0, 34.6, 29.1, 27.2.
^{11}B NMR (128 MHz, $CDCl_3$) δ 31.79.

2-([1,1'-biphenyl]-4-yl)-4,4,5,5-tetramethyl-1,3,2-dioxaborolane (3h).

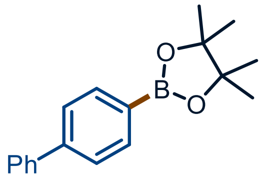

According to the *general procedure A*. The spectral data is consistent with the literature data.[5]
Colorless oil (70.5 mg, 63%).

R_f 0.70 (Petroleum ether/EtOAc, 40/1).
^{1}H NMR (400 MHz, $CDCl_3$) δ 7.88 (t, J = 7.4 Hz, 2H), 7.60 (t, J = 6.4 Hz, 4H), 7.46 – 7.38 (m, 2H), 7.37 – 7.29 (m, 1H), 1.34 (dd, J = 4.4, 1.3 Hz, 12H). **^{13}C NMR** (100 MHz, $CDCl_3$) δ 143.95, 141.07, 135.32, 128.83, 127.61, 127.29, 126.52, 83.87, 24.94.
^{11}B NMR (128 MHz, $CDCl_3$) δ 31.45.

2-(4-ethoxyphenyl)-4,4,5,5-tetramethyl-1,3,2-dioxaborolane (3i).

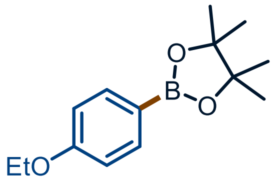

According to the *general procedure A*. The spectral data is consistent with the literature data.[5]
Colorless oil (71.4 mg, 72%).
R_f 0.70 (Petroleum ether/EtOAc, 40/1).
^{1}H NMR (400 MHz, $CDCl_3$) δ 7.73 (d, J = 8.5 Hz, 2H), 6.87 (d, J = 8.5 Hz, 2H), 4.04 (q, J = 7.0 Hz, 2H), 1.40 (t, J = 7.0 Hz, 3H), 1.32 (s, 12H).**^{13}C NMR** (100 MHz, $CDCl_3$) δ 161.56, 136.52, 113.84, 83.51, 63.22, 24.88, 14.78.
^{11}B NMR (128 MHz, $CDCl_3$) δ 31.50.

4,4,5,5-tetramethyl-2-(o-tolyl)-1,3,2-dioxaborolane (3j).

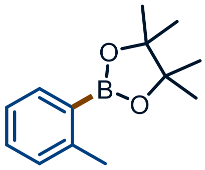

According to the *general procedure A*. The spectral data is consistent with the literature data.[5]
Colorless oil (67.1 mg, 77%).
R_f 0.70 (Petroleum ether/EtOAc, 40/1).
^{1}H NMR (400 MHz, $CDCl_3$) δ ^{1}H NMR (400 MHz, $CDCl_3$) δ 7.76 (d, J = 7.3 Hz, 1H), 7.31 (t, J = 7.4 Hz, 1H), 7.16 (d, J = 6.0 Hz, 2H), 2.54 (s, 3H), 1.34 (s, 12H). **^{13}C NMR** (100 MHz, $CDCl_3$) δ 147.76, 134.99, 127.40, 83.64, 29.17, 24.91, 15.56.
^{11}B NMR (128 MHz, $CDCl_3$) δ 30.68.

4,4,5,5-tetramethyl-2-(m-tolyl)-1,3,2-dioxaborolane (3k).

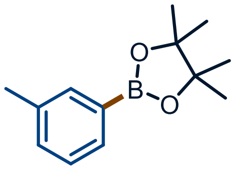

According to the *general procedure A*. The spectral data is consistent with the literature data.[5]
Colorless oil (66.1 mg, 76%).
R_f 0.70 (Petroleum ether/EtOAc, 40/1).
^{1}H NMR (400 MHz, $CDCl_3$) δ 7.71 (d, J = 7.5 Hz, 1H), 7.26 (t, J = 7.4 Hz, 1H), 7.11 (d, J = 6.1 Hz, 2H), 2.49 (s, 3H), 1.29 (s, 12H). **^{13}C NMR** (100 MHz, $CDCl_3$) δ 147.64, 134.87, 127.28, 83.52, 29.06, 24.79, 15.44.
^{11}B NMR (128 MHz, $CDCl_3$) δ 31.16.

2-(4-fluorophenyl)-4,4,5,5-tetramethyl-1,3,2-dioxaborolane (3l).

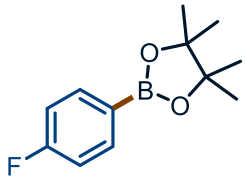

According to the *general procedure A*. The spectral data is consistent with the literature data.[5]
Colorless oil (58.6 mg, 66%).
R_f 0.50 (Petroleum ether/EtOAc, 40/1).
^{1}H NMR (400 MHz, $CDCl_3$) δ 8.02 (dd, J = 8.5, 6.3 Hz, 2H), 7.32 – 7.15 (m, 2H), 1.52 (s, 12H). **^{13}C NMR** (100 MHz, $CDCl_3$) δ166.38, 163.89, 137.10, 137.02, 114.90, 114.70, 83.82, 24.86, 24.82, 24.77.
^{11}B NMR (128 MHz, $CDCl_3$) δ 30.72.

2-(4-chlorophenyl)-4,4,5,5-tetramethyl-1,3,2-dioxaborolane (3m).

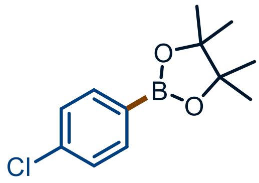

According to the *general procedure A*. The spectral data is consistent with the literature data.[5]
Clear oil (59.0 mg, 62%).
R_f 0.60 (Petroleum ether/EtOAc, 40/1).
^{1}H NMR (400 MHz, $CDCl_3$) δ 7.62 (d, J = 7.2 Hz, 2H), 7.23 (d, J = 7.5 Hz, 2H), 1.23 (s, 12H) **^{13}C NMR** (100 MHz, $CDCl_3$) δ 137.54, 136.13, 128.01, 84.01, 24.87.
^{11}B NMR (128 MHz, $CDCl_3$) δ 31.04.

4,4,5,5-tetramethyl-2-(4-(trifluoromethyl)phenyl)-1,3,2-dioxaborolane (3n).

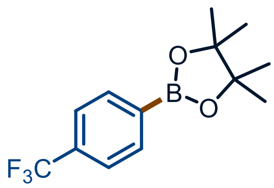

According to the *general procedure A*. The spectral data is consistent with the literature data.[5]

Yellow oil (65.2 mg, 60%).

R_f 0.60 (Petroleum ether/EtOAc, 40/1).

^{1}H NMR (400 MHz, $CDCl_3$) δ ^{1}H NMR (400 MHz, $CDCl_3$) δ 7.92 (d, J = 7.7 Hz, 2H), 7.60 (d, J = 7.8 Hz, 2H), 1.34 (s, 12H). **^{13}C NMR** (100 MHz, $CDCl_3$) δ 135.03, 133.31, 132.99, 132.67, 132.36, 128.23, 124.32, 124.28, 124.25, 124.21, 122.82, 120.12, 84.23, 24.76.

^{11}B NMR (128 MHz, $CDCl_3$) δ 30.88.

4,4,5,5-tetramethyl-2-(3-(trifluoromethyl)phenyl)-1,3,2-dioxaborolane (3o).

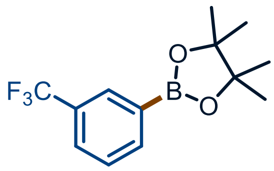

According to the *general procedure A*. The spectral data is consistent with the literature data.[5]

Yellow oil (76.1 mg, 70%).

R_f 0.50 (Petroleum ether/EtOAc, 40/1).

^{1}H NMR (400 MHz, $CDCl_3$) δ 8.11 (s, 1H), 8.02 (d, J = 7.4 Hz, 1H), 7.73 (d, J = 7.9 Hz, 1H), 7.51 (t, J = 7.6 Hz, 1H), 1.39 (s, 12H). **^{13}C NMR** (100 MHz, $CDCl_3$) δ 138.02, 131.41, 131.37, 131.34, 131.30, 128.04, 127.83, 127.79, 127.76, 127.72, 84.28, 24.84.

^{11}B NMR (128 MHz, $CDCl_3$) δ 30.70.

2-(3,4-dimethylphenyl)-4,4,5,5-tetramethyl-1,3,2-dioxaborolane (3p).

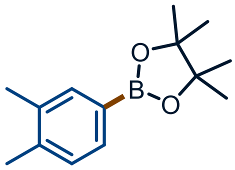

According to the *general procedure A*. The spectral data is consistent with the literature data.[5]

Colorless oil (64.9 mg, 70%).

R_f 0.45 (Petroleum ether/EtOAc, 40/1).

^{1}**H NMR** (400 MHz, $CDCl_3$) δ 7.66 (d, J = 15.7 Hz, 2H), 7.24 (s, 1H), 2.37 (s, 6H), 1.44 (s, 12H). ^{13}**C NMR** (100 MHz, $CDCl_3$) δ 140.14, 135.96, 132.44, 129.18, 83.59, 24.87, 20.02, 19.48.
^{11}**B NMR** (128 MHz, $CDCl_3$) δ 31.35.

2-(3,5-dimethylphenyl)-4,4,5,5-tetramethyl-1,3,2-dioxaborolane (3q).

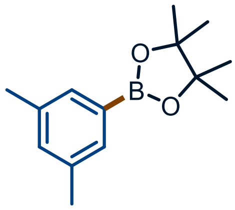

According to the *general procedure A*. The spectral data is consistent with the literature data.[5]
Colorless oil (63.0 mg, 69%).
R_f 0.7 (Petroleum ether/EtOAc, 40/1).
^{1}**H NMR** (400 MHz, $CDCl_3$) δ7.44 (s, 2H), 7.10 (s, 1H), 2.32 (s, 6H), 1.34 (s, 12H).
^{13}**C NMR** (100 MHz, $CDCl_3$) δ 137.19, 133.03, 132.44, 83.70, 24.89, 21.18.
^{11}**B NMR** (128 MHz, $CDCl_3$) δ 31.16.

2-(3,4-dichlorophenyl)-4,4,5,5-tetramethyl-1,3,2-dioxaborolane (3r).

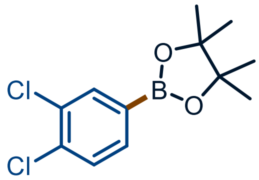

According to the *general procedure A*. The spectral data is consistent with the literature data.[5]
Colorless oil (59.8 mg, 55%).
R_f 0.70 (Petroleum ether/EtOAc, 40/1).
^{1}**H NMR** (400 MHz, $CDCl_3$) δ 7.67 (s, 1H), 7.40 (d, J = 7.9 Hz, 1H), 7.23 (d, J = 7.9 Hz, 1H), 1.14 (s, 12H). ^{13}**C NMR** (100 MHz, $CDCl_3$) δ 136.57, 135.49, 133.77, 132.26, 130.01, 84.33, 24.85.
^{11}**B NMR** (128 MHz, $CDCl_3$) δ 30.67.

2-(3,5-bis(trifluoromethyl)phenyl)-4,4,5,5-tetramethyl-1,3,2-dioxaborolane (3s).

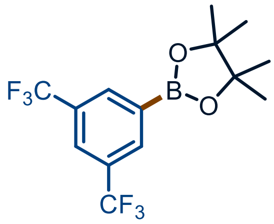

According to the *general procedure A*. The spectral data is consistent with the literature data.[5]

Colorless oil (76.1 mg, 56%).

R_f 0.60 (Petroleum ether/EtOAc, 40/1).

^{1}H NMR (400 MHz, $CDCl_3$) δ 8.22 (s, 2H), 7.92 (s, 1H), 1.35 (s, 12H). **^{13}C NMR** (100 MHz, $CDCl_3$) δ 134.65, 131.06, 130.73, 124.84, 124.68, 122.13, 84.83, 24.81.

^{11}B NMR (128 MHz, $CDCl_3$) δ 30.71.

4,4,5,5-tetramethyl-2-(3,4,5-trichlorophenyl)-1,3,2-dioxaborolane (3t).

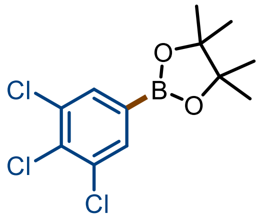

According to the *general procedure*. The spectral data is consistent with the literature data.[5]

White solid (74.9 mg, 61%). M.p. = 108 – 109 °C.

R_f 0.70 (Petroleum ether/EtOAc, 40/1).

^{1}H NMR (400 MHz, $CDCl_3$) δ 7.76 (s, 2H), 1.34 (s, 12H). **^{13}C NMR** (100 MHz, $CDCl_3$) δ 134.44, 134.10, 133.96, 84.67, 24.85.

^{11}B NMR (128 MHz, $CDCl_3$) δ 30.31.

4,4,5,5-tetramethyl-2-(naphthalen-2-yl)-1,3,2-dioxaborolane (3u).

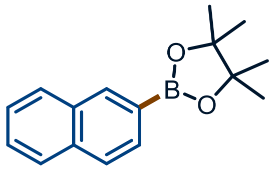

According to the *general procedure A*. The spectral data is consistent with the literature data.[5]

White solid (78.2 mg, 77%). M.p. = 107 – 108 °C.

R_f 0.60 (Petroleum ether/EtOAc, 40/1).

^{1}**H NMR** (400 MHz, $CDCl_3$) δ 8.39 (s, 1H), 7.86 (dd, *J* = 23.2, 8.6 Hz, 4H), 7.49 (t, *J* = 7.7 Hz, 2H), 1.39 (s, 12H). ^{13}**C NMR** (100 MHz, $CDCl_3$) δ 136.29, 135.08, 132.86, 130.45, 128.69, 127.75, 127.01, 125.83, 83.95, 24.96.
^{11}**B NMR** (128 MHz, $CDCl_3$) δ 31.83.

(allylsulfonyl)benzene (5a).

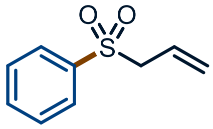

According to the *general procedure B*. The spectral data is consistent with the literature data.[6]
Yellow oil (60.4 mg, 83%).
R_f 0.60 (Petroleum ether/EtOAc, 40/1).
^{1}**H NMR** (400 MHz, $CDCl_3$) δ 7.85 (d, *J* = 7.9 Hz, 2H), 7.62 (t, *J* = 7.4 Hz, 1H), 7.53 (t, *J* = 7.6 Hz, 2H), 5.85 – 5.66 (m, 1H), 5.30 (d, *J* = 10.1 Hz, 1H), 5.12 (d, *J* = 17.1 Hz, 1H), 3.78 (d, *J* = 7.4 Hz, 2H).^{13}**C NMR** (100 MHz, $CDCl_3$) δ 138.30, 133.78, 129.07, 128.50, 124.74, 124.64, 60.88.

1-(allylsulfonyl)-4-methylbenzene (5b).

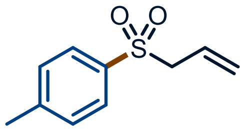

According to the *general procedure B*. The spectral data is consistent with the literature data.[6]
Yellow oil (62.7 mg, 80%).
R_f 0.60 (Petroleum ether/EtOAc, 40/1).
^{1}**H NMR** (400 MHz, $CDCl_3$) δ 7.75 (d, *J* = 8.1 Hz, 2H), 7.35 (d, *J* = 8.0 Hz, 2H), 5.79 (dq, *J* = 10.0, 7.4 Hz, 1H), 5.33 (d, *J* = 10.1 Hz, 1H), 5.15 (d, *J* = 17.1 Hz, 1H), 3.79 (d, *J* = 7.3 Hz, 2H), 2.45 (s, 3H). ^{13}**C NMR** (100 MHz, $CDCl_3$) δ 144.74, 135.41, 129.68, 128.50, 124.82, 124.56, 60.96, 21.64.

(methylsulfonyl)benzene (5c).

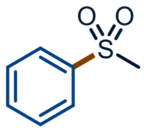

According to the *general procedure B*. The spectral data is consistent with the literature data.[6]
Yellow oil (49.2 mg, 79%).

R_f 0.60 (Petroleum ether/EtOAc, 40/1).
^{1}H NMR (400 MHz, $CDCl_3$) δ ^{1}H NMR (400 MHz, $CDCl_3$) δ 7.92 (d, *J* = 8.0 Hz, 2H), 7.64 (t, *J* = 7.0 Hz, 1H), 7.55 (t, *J* = 7.7 Hz, 2H), 3.03 (s, 3H).**^{13}C NMR** (100 MHz, $CDCl_3$) δ 140.60, 133.72, 129.39, 127.36, 44.51.

1-methyl-4-(methylsulfonyl)benzene (5d).

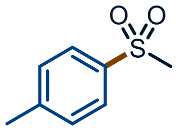

According to the *general procedure B*. The spectral data is consistent with the literature data.[6]
Yellow oil (52.3 mg, 77%).
R_f 0.60 (Petroleum ether/EtOAc, 40/1).
^{1}H NMR (400 MHz, $CDCl_3$) δ ^{1}H NMR (400 MHz, $CDCl_3$) δ 7.83 (d, *J* = 8.2 Hz, 2H), 7.37 (d, *J* = 8.0 Hz, 2H), 3.04 (s, 3H), 2.46 (s, 3H). **^{13}C NMR** (100 MHz, $CDCl_3$) δ ^{13}C NMR (101 MHz, $CDCl_3$) δ 144.68, 137.75, 129.96, 127.39, 44.62, 21.62.

(benzylsulfonyl)benzene (5e).

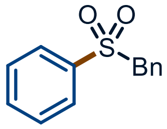

According to the *general procedure B*. The spectral data is consistent with the literature data.[6]
Yellow oil (72.3 mg, 78%).
R_f 0.40 (Petroleum ether/EtOAc, 20/1).
^{1}H NMR (400 MHz, $CDCl_3$) δ 7.76 (dd, *J* = 15.4, 7.4 Hz, 3H), 7.60 (t, *J* = 7.1 Hz, 2H), 7.51 – 7.37 (m, 3H), 7.23 (d, *J* = 6.7 Hz, 2H), 4.46 (s, 2H). **^{13}C NMR** (100 MHz, $CDCl_3$) δ 137.87, 133.72, 130.83, 128.89, 128.78, 128.64, 128.59, 128.13, 62.90.

1-(benzylsulfonyl)-4-methylbenzene (5f).

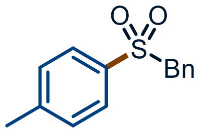

According to the *general procedure*. The spectral data is consistent with the literature data.[6]
Yellow oil (80.6 mg, 82%).
R_f 0.40 (Petroleum ether/EtOAc, 20/1).
^{1}H NMR (400 MHz, $CDCl_3$) δ 7.50 (d, *J* = 7.8 Hz, 2H), 7.37 – 7.17 (m, 5H), 7.09 (d, *J* = 7.1 Hz, 2H), 4.29 (s, 2H), 2.41 (s, 3H). **^{13}C NMR** (100 MHz, $CDCl_3$) δ 144.71, 134.97, 130.85, 129.53, 128.71, 128.66, 128.57, 128.31, 62.93, 21.67.

1-(benzylsulfonyl)-4-bromobenzene (5g).

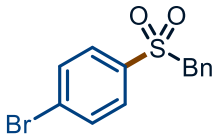

According to the *general procedure B*. The spectral data is consistent with the literature data.[6]

Yellow oil (100.1 mg, 81%).

R_f 0.40 (Petroleum ether/EtOAc, 20/1).

^{1}H NMR (400 MHz, $CDCl_3$) δ 7.35 (d, J = 8.5 Hz, 2H), 7.23 (d, J = 8.5 Hz, 2H), 7.13 – 7.03 (m, 3H), 6.86 (d, J = 7.2 Hz, 2H), 4.08 (s, 2H). **^{13}C NMR** (100 MHz, $CDCl_3$) δ 136.83, 132.20, 130.82, 130.21, 129.13, 128.98, 128.74, 127.84, 62.90.

^{19}F NMR (376 MHz, $CDCl_3$) δ-86.97 (d, J = 39.0 Hz), -92.36 (d, J = 39.0 Hz).

1-(benzylsulfonyl)-4-chlorobenzene (5h).

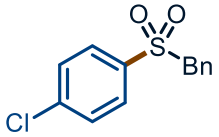

According to the *general procedure B*. The spectral data is consistent with the literature data.[6]

Yellow oil (74.4 mg, 70%).

R_f 0.40 (Petroleum ether/EtOAc, 20/1).

^{1}H NMR (400 MHz, $CDCl_3$) δ 7.35 (d, J = 8.5 Hz, 2H), 7.23 (d, J = 8.5 Hz, 2H), 7.19 – 7.07 (m, 3H), 6.91 (d, J = 7.2 Hz, 2H), 4.13 (s, 2H). **^{13}C NMR** (100 MHz, $CDCl_3$) δ 140.52, 136.28, 130.81, 130.15, 129.21, 128.97, 128.73, 127.88, 62.93.

benzenesulfonamide (5i).

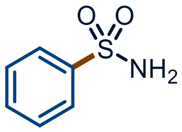

According to the *general procedure C*. The spectral data is consistent with the literature data.[6]

Yellow oil (27.0 mg, 43%).

R_f 0.40 (Petroleum ether/EtOAc, 1/1).

^{1}H NMR (400 MHz, DMSO) δ 7.84 (d, J = 6.6 Hz, 2H), 7.58 (t, J = 6.3 Hz, 3H), 7.37 (s, 2H). **^{13}C NMR** (100 MHz, DMSO) δ144.60, 132.27, 129.41, 126.05.

4-methylbenzenesulfonamide (5j).

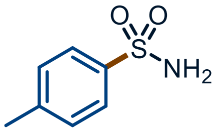

According to the *general procedure C*. The spectral data is consistent with the literature data.[6]

Yellow oil (29.4 mg, 47%).

R_f 0.40 (Petroleum ether/EtOAc, 1/1).

^{1}H NMR (400 MHz, DMSO) δ7.71 (d, J = 8.2 Hz, 2H), 7.36 (d, J = 8.0 Hz, 2H), 7.28 (s, 2H), 2.37 (s, 3H). **^{13}C NMR** (100 MHz, DMSO) δ142.33, 141.89, 129.77, 126.09, 21.38.

benzenesulfonyl chloride (5k).

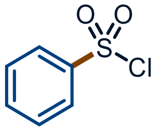

According to the *general procedure D*. The spectral data is consistent with the literature data.[6]

Yellow oil (35.2 mg, 50%).

R_f 0.60 (Petroleum ether/EtOAc, 40/1).

^{1}H NMR (400 MHz, $CDCl_3$) δ 8.05 (dt, J = 8.7, 1.6 Hz, 2H), 7.81 – 7.72 (m, 1H), 7.68 – 7.59 (m, 2H). **^{13}C NMR** (100 MHz, $CDCl_3$) δ 144.36, 135.34, 129.76, 126.99.

1-(phenylsulfonyl)-1H-pyrazole (5l).

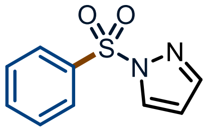

According to the *general procedure E*. The spectral data is consistent with the literature data.[6]

Yellow oil (35.7 mg, 43%).

R_f 0.40 (Petroleum ether/EtOAc, 20/1).

^{1}H NMR (400 MHz, $CDCl_3$) δ 8.09 (s, 1H), 7.98 (d, J = 7.9 Hz, 2H), 7.70 (s, 1H), 7.61 (t, J = 7.4 Hz, 1H), 7.50 (t, J = 7.5 Hz, 2H), 6.37 (s, 1H). **^{13}C NMR** (100 MHz, $CDCl_3$) δ 145.40, 137.08, 134.59, 131.28, 129.42, 128.06, 108.95.

1-(phenylsulfonyl)pyrrolidine (5m).

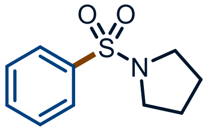

According to the *general procedure E*. The spectral data is consistent with the literature data.[6]

Yellow oil (36.2 mg, 42%).

R_f 0.40 (Petroleum ether/EtOAc, 60/1).

^{1}H NMR (400 MHz, $CDCl_3$) δ 7.78 (d, J = 7.7 Hz, 2H), 7.58 – 7.52 (m, 1H), 7.48 (t, J = 7.5 Hz, 2H), 3.20 (t, J = 6.3 Hz, 4H), 1.70 (t, J = 6.5 Hz, 4H). **^{13}C NMR** (100 MHz, $CDCl_3$) δ 136.89, 132.60, 129.02, 127.45, 47.94, 25.21.

4-(phenylsulfonyl)morpholine (5n).

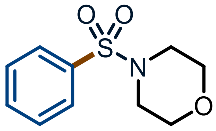

According to the *general procedure E*. The spectral data is consistent with the literature data.[6]

Yellow oil (57.9 mg, 55%).

R_f 0.70 (Petroleum ether/EtOAc, 15/1).

^{1}H NMR (400 MHz, $CDCl_3$) δ ^{1}H NMR (400 MHz, $CDCl_3$) δ 7.73 (d, J = 7.6 Hz, 2H), 7.60 (t, J = 7.3 Hz, 1H), 7.53 (t, J = 7.5 Hz, 2H), 3.75 – 3.69 (m, 4H), 3.01 – 2.94 (m, 4H). **^{13}C NMR** (100 MHz, $CDCl_3$) δ 135.13, 133.09, 129.15, 127.85, 66.11, 46.00.

3-chloro-4-fluoro-N-methyl-N-(3-phenyl-3-(4-(trifluoromethyl)phenoxy)propyl)benzenesulfonamide (5o).

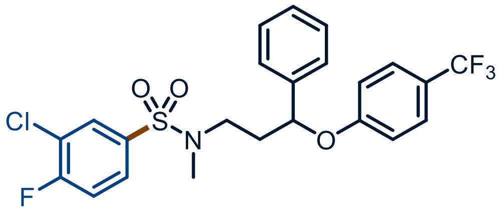

According to the *general procedure E*. The spectral data is consistent with the literature data.[6]

Yellow oil (100.2 mg, 50%).

R_f 0.50 (Petroleum ether/EtOAc, 1/1).

^{1}H NMR (400 MHz, $CDCl_3$) δ 7.74 (d, *J* = 5.6 Hz, 1H), 7.54 (s, 1H), 7.33 (d, *J* = 8.0 Hz, 2H), 7.24 (s, 4H), 7.15 (t, *J* = 9.2 Hz, 2H), 6.80 (d, *J* = 8.0 Hz, 2H), 5.26 – 5.13 (m, 1H), 3.23 (dd, *J* = 13.1, 6.5 Hz, 1H), 3.12 – 2.98 (m, 1H), 2.68 (s, 3H), 2.22 – 1.91 (m, 2H). **^{13}C NMR** (100 MHz, $CDCl_3$) δ 161.89, 160.16, 159.33, 140.23, 134.59, 130.21, 128.98, 128.19, 127.82, 127.74, 126.85, 126.81, 125.77, 122.96, 122.64, 122.46, 117.54, 117.32, 115.84, 47.10, 37.18, 35.40.

2-chloro-N-methyl-N-(3-phenyl-3-(4-(trifluoromethyl)phenoxy)propyl)benzenesulfonamide (5p).

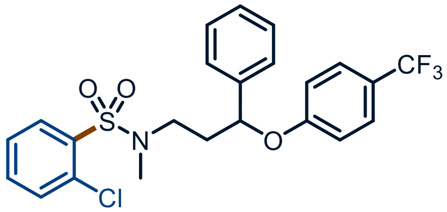

According to the *general procedure E*. The spectral data is consistent with the literature data.[6]
Black oil (77.2 mg, 40%).
R_f 0.60 (Petroleum ether/EtOAc, 1/1).
^{1}H NMR (400 MHz, $CDCl_3$) δ 7.96 (d, *J* = 7.5 Hz, 1H), 7.42 – 7.14 (m, 11H), 6.76 (d, *J* = 8.0 Hz, 2H), 5.22 – 5.08 (m, 1H), 3.55 – 3.38 (m, 1H), 3.36 – 3.22 (m, 1H), 2.87 (s, 3H), 2.11 (d, *J* = 33.9 Hz, 2H). **^{13}C NMR** (100 MHz, $CDCl_3$) δ 160.14, 140.41, 136.78, 133.54, 132.11, 132.06, 132.02, 128.94, 128.07, 126.92, 126.76, 126.72, 125.68, 115.76, 99.99, 46.77, 37.20, 34.74.

N-methyl-N-(3-phenyl-3-(4-(trifluoromethyl)phenoxy)propyl)-2-(trifluoromethyl) benzenesulfonamide (5q).

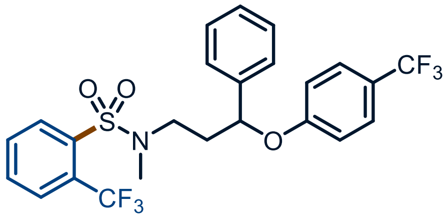

According to the *general procedure E*. The spectral data is consistent with the literature data.[6]
White solid (91.0 mg, 44%). M.p. = 144 – 145 °C.
R_f 0.60 (Petroleum ether/EtOAc, 1/1).
^{1}H NMR (400 MHz, $CDCl_3$) δ 7.95 (d, *J* = 4.9 Hz, 1H), 7.74 (s, 1H), 7.54 (s, 2H), 7.33 (d, *J* = 8.0 Hz, 2H), 7.29 – 7.14 (m, 5H), 6.76 (d, *J* = 8.0 Hz, 2H), 5.33 – 4.99 (m, 1H), 3.57 – 3.22 (m, 2H), 2.83 (s, 3H), 2.16 (dd, *J* = 22.1, 19.1 Hz, 2H). **^{13}C NMR**

(100 MHz, $CDCl_3$) *δ* 160.13, 140.29, 138.19, 132.55, 132.13, 131.44, 128.95, 128.63, 128.57, 128.11, 126.78, 126.75, 125.68, 123.93, 123.15, 122.82, 121.21, 115.75, 46.84, 37.05, 34.58.

N-methyl-N-(3-phenyl-3-(4-(trifluoromethyl)phenoxy)propyl)benzenesulfonamide (5r).

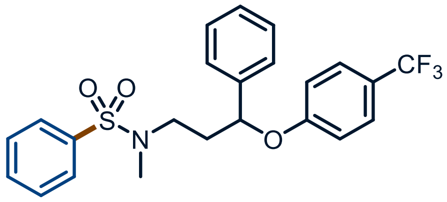

According to the *general procedure E*. The spectral data is consistent with the literature data.[6]

White solid (107.7 mg, 60%). M.p. = 155 – 156 °C.

*R*f 0.60 (Petroleum ether/EtOAc, 1/1).

^{1}H NMR (400 MHz, $CDCl_3$) *δ* 7.76 (d, *J* = 7.3 Hz, 2H), 7.66 – 7.20 (m, 10H), 6.90 (d, *J* = 8.6 Hz, 2H), 5.31 (dd, *J* = 8.6, 4.1 Hz, 1H), 3.20 (tdd, *J* = 13.3, 10.6, 6.3 Hz, 2H), 2.75 (s, 3H), 2.37 – 1.97 (m, 2H). **^{13}C NMR** (100 MHz, $CDCl_3$) *δ* ^{13}C NMR (101 MHz, $CDCl_3$) δ 159.77, 140.04, 136.41, 133.17, 131.74, 131.69, 131.65, 128.57, 127.70, 126.55, 126.39, 126.35, 125.31, 122.40, 115.39, 76.88, 46.40, 36.83, 34.37.

1-methyl-3-phenylquinoxalin-2(1H)-one (7a).

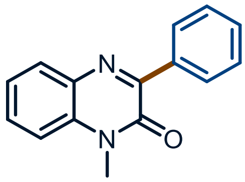

According to the *general procedure F*. The spectral data is consistent with the literature data.[7]

Black oil (32.5 mg, 69%).

*R*f 0.60 (Petroleum ether/EtOAc, 7/1).

^{1}H NMR (400 MHz, $CDCl_3$) *δ* ^{1}H NMR (400 MHz, $CDCl_3$) δ 8.35 – 8.25 (m, 2H), 7.94 (dd, *J* = 8.0, 1.1 Hz, 1H), 7.56 – 7.32 (m, 6H), 3.76 (s, 3H). **^{13}C NMR** (100 MHz, $CDCl_3$) *δ* ^{13}C NMR (101 MHz, $CDCl_3$) δ 154.73, 154.19, 136.10, 133.38, 133.12, 130.47, 130.33, 129.59, 129.58, 129.56, 128.13, 128.12, 128.11, 128.09, 128.08, 123.74, 113.59, 29.31.

6-methoxy-1-methyl-3-phenylquinoxalin-2(1H)-one (7b).

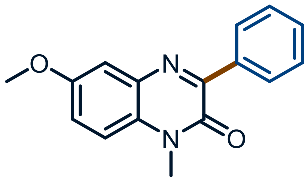

According to the *general procedure F*. The spectral data is consistent with the literature data.[7]

Brown solid (37.2 mg, 70%). M.p. = 110– 111 °C.

R_f 0.30 (Petroleum ether/EtOAc, 20/1).

^{1}H NMR (400 MHz, $CDCl_3$) δ 8.29 (d, *J* = 2.9 Hz, 2H), 7.51 – 7.44 (m, 3H), 7.41 (s, 1H), 7.22 (dd, *J* = 23.6, 7.9 Hz, 2H), 3.90 (s, 3H), 3.75 (s, 3H). **^{13}C NMR** (100 MHz, $CDCl_3$) δ156.06, 154.63, 154.40, 136.21, 133.85, 130.30, 129.55, 128.09, 127.71, 119.74, 114.51, 111.60, 55.80, 29.45.

6-fluoro-1-methyl-3-phenylquinoxalin-2(1H)-one (7c)

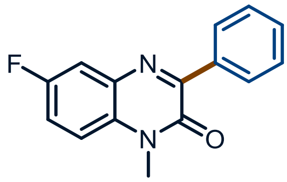

According to the *general procedure F*. The spectral data is consistent with the literature data.[7]

Yellow oil (36.5 mg, 72%).

R_f 0.40 (Petroleum ether/EtOAc, 20/1).

^{1}H NMR (400 MHz, $CDCl_3$) δ8.35 – 8.25 (m, 2H), 7.62 (dd, *J* = 8.7, 2.6 Hz, 1H), 7.48 (dd, *J* = 5.2, 1.8 Hz, 3H), 7.36 – 7.23 (m, 2H), 3.75 (s, 3H). **^{13}C NMR** (100 MHz, $CDCl_3$) δ 159.98, 157.56, 155.35, 154.34, 135.74, 133.68, 133.57, 130.70, 130.06, 129.66, 128.13, 118.18, 117.94, 115.75, 115.53, 114.73, 114.65, 29.58.

6-chloro-1-methyl-3-phenylquinoxalin-2(1H)-one (7d).

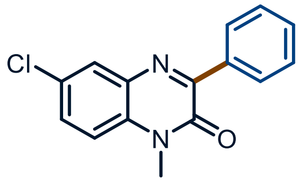

According to the *general procedure F*. The spectral data is consistent with the literature data.[7]

Yellow oil (41.5 mg, 77%).

R_f 0.50 (Petroleum ether/EtOAc, 20/1).

^{1}H NMR (400 MHz, $CDCl_3$) δ ^{1}H NMR (400 MHz, $CDCl_3$) δ 8.30 (d, *J* = 7.4 Hz, 2H), 7.90 (s, 1H), 7.49 (d, *J* = 6.2 Hz, 4H), 7.23 (d, *J* = 9.0 Hz, 1H), 3.72 (s, 3H). **^{13}C**

NMR (100 MHz, $CDCl_3$) δ 155.16, 154.36, 135.66, 133.59, 132.05, 130.73, 130.21, 129.66, 129.00, 128.13, 114.72, 29.49.

6-bromo-1-methyl-3-phenylquinoxalin-2(1H)-one (7e).

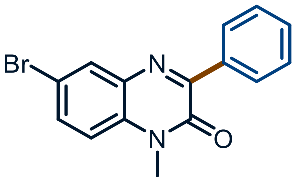

According to the *general procedure F*. The spectral data is consistent with the literature data.[7]

Yellow oil (48.3 mg, 77%).

R_f 0.70 (Petroleum ether/EtOAc, 20/1).

^{1}H NMR (400 MHz, $CDCl_3$) δ 8.36 (s, 2H), 8.13 (d, J = 4.3 Hz, 1H), 7.69 (t, J = 6.6 Hz, 1H), 7.54 (d, J = 5.5 Hz, 3H), 7.27 – 7.18 (m, 1H), 3.78 (d, J = 6.5 Hz, 3H). **^{13}C NMR** (100 MHz, $CDCl_3$) δ 155.09, 154.35, 135.63, 133.89, 132.95, 132.71, 132.47, 130.75, 129.66, 128.14, 116.25, 115.02, 29.47.

1,6,7-trimethyl-3-phenylquinoxalin-2(1H)-one (7f)

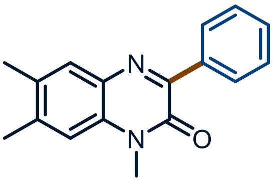

According to the *general procedure F*. The spectral data is consistent with the literature data.[7]

Yellow oil (39.6 mg, 75%).

R_f 0.70 (Petroleum ether/EtOAc, 20/1).

^{1}H NMR (400 MHz, $CDCl_3$) δ 8.28 (dd, J = 6.6, 3.1 Hz, 2H), 7.68 (s, 1H), 7.52 – 7.42 (m, 3H), 7.07 (s, 1H), 3.72 (s, 3H), 2.42 (s, 3H), 2.35 (s, 3H). **^{13}C NMR** (100 MHz, $CDCl_3$) δ ^{13}C NMR (101 MHz, $CDCl_3$) δ 154.79, 152.90, 140.29, 136.40, 132.65, 131.58, 131.43, 130.49, 129.97, 129.45, 128.02, 114.14, 29.19, 20.65, 19.21.

6,7-difluoro-1-methyl-3-phenylquinoxalin-2(1H)-one (7g)

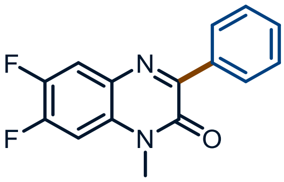

According to the *general procedure F*. The spectral data is consistent with the literature data.[7]

Yellow oil (35.9 mg, 66%).

R_f 0.70 (Petroleum ether/EtOAc, 20/1).
^{1}H NMR (400 MHz, $CDCl_3$) δ 8.32 – 8.24 (m, 2H), 7.74 (dd, J = 10.2, 8.2 Hz, 1H), 7.51 – 7.44 (m, 3H), 7.13 (dd, J = 11.3, 7.1 Hz, 1H), 3.72 (s, 3H). **^{13}C NMR** (100 MHz, $CDCl_3$) δ 154.42, 154.38, 154.27, 152.91, 152.77, 150.39, 150.25, 148.12, 147.98, 145.66, 145.53, 135.51, 130.73, 129.65, 129.54, 128.17, 117.94, 117.78, 102.33, 102.10, 29.85.

6,7-dichloro-1-methyl-3-phenylquinoxalin-2(1H)-one (7h)

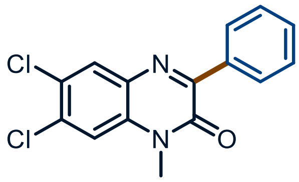

According to the *general procedure F*. The spectral data is consistent with the literature data.[7]
Yellow oil (48.6 mg, 80%).
R_f 0.70 (Petroleum ether/EtOAc, 20/1).
^{1}H NMR (400 MHz, $CDCl_3$) δ 8.29 (dd, J = 7.7, 1.7 Hz, 2H), 8.00 (s, 1H), 7.49 (t, J = 6.5 Hz, 3H), 7.40 (s, 1H), 3.70 (s, 3H).
^{13}C NMR (100 MHz, $CDCl_3$) δ 155.06, 154.09, 135.36, 134.28, 132.69, 132.13, 131.06, 130.93, 129.65, 128.17, 127.49, 115.06, 29.56.

6,7-dibromo-1-methyl-3-phenylquinoxalin-2(1H)-one (7i)

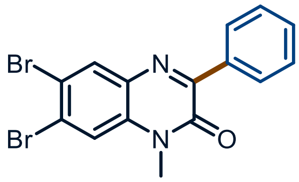

According to the *general procedure F*. The spectral data is consistent with the literature data.[7]
Yellow oil (57.8 mg, 74%).
R_f 0.70 (Petroleum ether/EtOAc, 20/1).
^{1}H NMR (400 MHz, $CDCl_3$) δ ^{1}H NMR (400 MHz, $CDCl_3$) δ 8.30 (dd, J = 7.9, 1.7 Hz, 2H), 8.18 (s, 1H), 7.60 (s, 1H), 7.52 – 7.49 (m, 2H), 7.26 (s, 1H), 3.72 (s, 3H).
^{13}C NMR (100 MHz, $CDCl_3$) δ 154.94, 153.98, 135.24, 134.16, 132.57, 132.01, 130.94, 130.81, 129.53, 128.05, 127.38, 114.95, 29.44.

1-ethyl-3-phenylquinoxalin-2(1H)-one (7j)

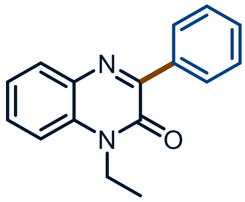

According to the *general procedure F*. The spectral data is consistent with the literature data.[7]
Yellow oil (33.0 mg, 66%).
R_f 0.70 (Petroleum ether/EtOAc, 20/1).
^{1}H NMR (400 MHz, $CDCl_3$) δ 8.28 – 8.19 (m, 2H), 7.85 (d, J = 8.0 Hz, 1H), 7.44 (t, J = 7.8 Hz, 1H), 7.38 (d, J = 3.0 Hz, 3H), 7.24 (t, J = 7.8 Hz, 2H), 4.27 (q, J = 7.1 Hz, 2H), 1.31 (t, J = 7.1 Hz, 3H). **^{13}C NMR** (100 MHz, $CDCl_3$) δ 154.18, 154.06, 136.10, 133.42, 132.31, 130.73, 130.31, 129.63, 128.08, 123.53, 113.44, 37.60, 12.44.

1-benzyl-3-phenylquinoxalin-2(1H)-one (7k)

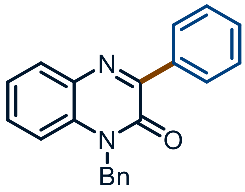

According to the *general procedure F*. The spectral data is consistent with the literature data.[7]
Yellow oil (31.2 mg, 50%).
R_f 0.70 (Petroleum ether/EtOAc, 20/1).
^{1}H NMR (400 MHz, $CDCl_3$) δ 8.41 – 8.30 (m, 2H), 7.95 (dd, J = 7.9, 1.1 Hz, 1H), 7.53 – 7.38 (m, 4H), 7.37 – 7.21 (m, 7H), 5.57 (s, 2H). **^{13}C NMR** (100 MHz, $CDCl_3$) δ154.82, 154.25, 136.04, 135.39, 133.40, 132.76, 130.62, 130.47, 130.36, 129.68, 128.97, 128.15, 127.73, 127.01, 123.84, 114.39, 46.16.

ethyl 2-(2-oxo-3-phenylquinoxalin-1(2H)-yl)propanoate (7l)

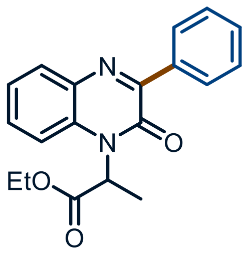

According to the *general procedure F*. The spectral data is consistent with the literature data.[7]
Yellow oil (41.8 mg, 65%).
R_f 0.70 (Petroleum ether/EtOAc, 20/1).

¹H NMR (400 MHz, $CDCl_3$) δ ¹H NMR (400 MHz, $CDCl_3$) δ 8.22 – 8.14 (m, 2H), 7.85 (d, *J* = 8.0 Hz, 1H), 7.43 – 7.30 (m, 4H), 7.24 (t, *J* = 7.6 Hz, 1H), 7.08 (d, *J* = 8.4 Hz, 1H), 5.56 (s, 1H), 4.18 – 3.92 (m, 2H), 1.62 (d, *J* = 7.0 Hz, 3H), 1.03 (t, *J* = 7.1 Hz, 3H). **¹³C NMR** (100 MHz, $CDCl_3$) *δ* 169.79, 153.97, 153.92, 135.73, 133.68, 131.76, 131.11, 130.50, 130.24, 129.59, 128.13, 123.90, 113.36, 61.86, 51.64, 14.09, 13.93.

1-(2-oxo-2-phenylethyl)-3-phenylquinoxalin-2(1H)-one (7m)

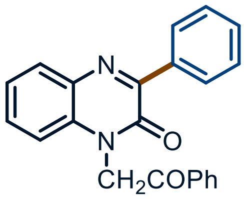

According to the *general procedure F*. The spectral data is consistent with the literature data.[7]

Yellow oil (31.96 mg, 47%).

R_f 0.70 (Petroleum ether/EtOAc, 20/1).

¹H NMR (400 MHz, $CDCl_3$) *δ* 8.32 (dd, *J* = 6.7, 3.0 Hz, 2H), 8.09 (d, *J* = 7.6 Hz, 2H), 7.98 (dd, *J* = 7.9, 1.0 Hz, 1H), 7.67 (t, *J* = 7.4 Hz, 1H), 7.55 (t, *J* = 7.7 Hz, 2H), 7.51 – 7.42 (m, 4H), 7.35 (t, *J* = 7.6 Hz, 1H), 6.98 (d, *J* = 8.3 Hz, 1H), 5.79 (s, 2H). **¹³C NMR** (100 MHz, $CDCl_3$) *δ* 191.25, 154.45, 153.81, 135.88, 134.62, 134.34, 133.31, 132.82, 130.75, 130.42, 129.62, 129.09, 128.21, 128.11, 123.92, 113.47, 48.66.

ethyl 2-(2-oxo-3-phenylquinoxalin-1(2H)-yl)acetate (7n)

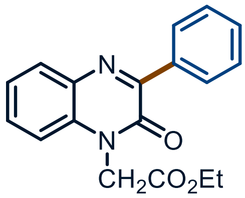

According to the *general procedure F*. The spectral data is consistent with the literature data.[7]

Yellow oil (27.1 mg, 44%).

R_f 0.60 (Petroleum ether/EtOAc, 20/1).

¹H NMR (400 MHz, $CDCl_3$) *δ* 8.50 – 8.40 (m, 2H), 8.09 (d, *J* = 8.0 Hz, 1H), 7.68 – 7.57 (m, 4H), 7.50 (t, *J* = 7.6 Hz, 1H), 7.23 (d, *J* = 8.4 Hz, 1H), 5.21 (s, 2H), 4.39 (q, *J* = 7.1 Hz, 2H), 1.41 (t, *J* = 7.1 Hz, 3H). **¹³C NMR** (100 MHz, $CDCl_3$) *δ* 167.20, 154.32, 153.91, 135.75, 133.20, 132.55, 130.81, 130.50, 129.59, 128.11, 124.05, 113.04, 62.11, 43.79, 14.15.

1-allyl-3-phenylquinoxalin-2(1H)-one (7o)

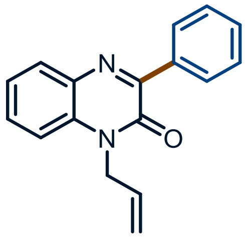

According to the *general procedure F*. The spectral data is consistent with the literature data.[7]

Yellow oil (27.2 mg, 52%).

R_f 0.70 (Petroleum ether/EtOAc, 20/1).

^{1}H NMR (400 MHz, $CDCl_3$) δ 8.26 (dd, J = 5.4, 2.4 Hz, 2H), 7.88 (d, J = 8.0 Hz, 1H), 7.49 – 7.43 (m, 1H), 7.42 – 7.38 (m, 3H), 7.28 (t, J = 7.6 Hz, 1H), 7.23 (d, J = 8.4 Hz, 1H), 5.91 (ddd, J = 15.9, 10.3, 5.1 Hz, 1H), 5.22 (d, J = 10.4 Hz, 1H), 5.15 (d, J = 17.3 Hz, 1H), 4.90 (d, J = 4.8 Hz, 2H). **^{13}C NMR** (100 MHz, $CDCl_3$) δ 154.27, 154.10, 136.00, 133.29, 132.61, 130.71, 130.57, 130.39, 130.26, 129.63, 128.09, 123.74, 118.19, 114.13, 44.76.

3-phenyl-1-(prop-2-yn-1-yl)quinoxalin-2(1H)-one (7p)

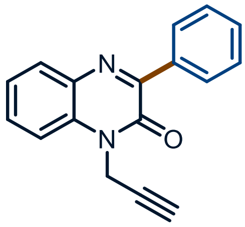

According to the *general procedure F*. The spectral data is consistent with the literature data.[7]

Yellow oil (32.7 mg, 63%).

R_f 0.70 (Petroleum ether/EtOAc, 20/1).

^{1}H NMR (400 MHz, $CDCl_3$) δ 8.32 (dd, J = 6.7, 3.0 Hz, 2H), 7.95 (dd, J = 8.0, 1.2 Hz, 1H), 7.62 – 7.56 (m, 1H), 7.47 (dd, J = 7.2, 3.3 Hz, 4H), 7.41 – 7.35 (m, 1H), 5.11 (d, J = 2.5 Hz, 2H), 2.31 (t, J = 2.5 Hz, 1H). **^{13}C NMR** (100 MHz, $CDCl_3$) δ 153.98, 153.72, 135.78, 133.31, 131.87, 130.63, 130.53, 130.45, 129.61, 128.14, 124.15, 114.07, 73.19, 31.69.

3-(4-methoxyphenyl)-1-methylquinoxalin-2(1H)-one (7q)

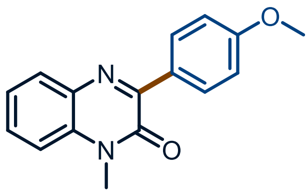

According to the *general procedure F*. The spectral data is consistent with the literature data.[7]

Yellow oil (40.9 mg, 77%).
R_f 0.70 (Petroleum ether/EtOAc, 20/1).
^{1}H NMR (400 MHz, $CDCl_3$) δ 8.48 – 8.35 (m, 2H), 7.90 (dd, J = 8.0, 1.3 Hz, 1H), 7.59 – 7.44 (m, 1H), 7.45 – 7.26 (m, 2H), 7.05 – 6.91 (m, 2H), 3.87 (s, 3H), 3.75 (s, 3H). **^{13}C NMR** (100 MHz, $CDCl_3$) δ 161.48, 154.85, 153.20, 133.18, 133.13, 131.39, 130.14, 129.77, 128.77, 123.67, 113.52, 113.49, 55.40, 29.28.

3-(4-isopropoxyphenyl)-1-methylquinoxalin-2(1H)-one (7r)

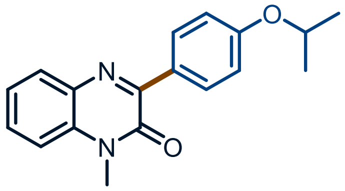

According to the *general procedure F*. The spectral data is consistent with the literature data.[7]
Yellow oil (42.3 mg, 72%).
R_f 0.50 (Petroleum ether/EtOAc, 10/1).
^{1}H NMR (400 MHz, $CDCl_3$) δ 8.37 (d, J = 8.8 Hz, 2H), 7.91 (d, J = 7.9 Hz, 1H), 7.53 (t, J = 7.4 Hz, 1H), 7.42 – 7.28 (m, 2H), 6.97 (d, J = 8.8 Hz, 2H), 4.66 (dt, J = 12.0, 6.0 Hz, 1H), 3.76 (s, 3H), 1.37 (d, J = 6.0 Hz, 6H). **^{13}C NMR** (100 MHz, $CDCl_3$) δ 159.94, 154.87, 153.28, 133.22, 133.13, 131.38, 130.12, 129.68, 128.42, 123.64, 115.17, 113.49, 69.88, 29.26, 22.04.

3-([1,1'-biphenyl]-4-yl)-1-methylquinoxalin-2(1H)-one (7s)

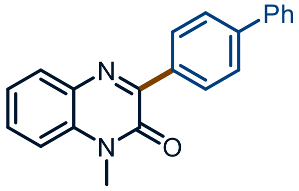

According to the *general procedure F*. The spectral data is consistent with the literature data.[7]
Yellow oil (49.9 mg, 80%).
R_f 0.70 (Petroleum ether/EtOAc, 20/1).
^{1}H NMR (400 MHz, $CDCl_3$) δ 8.47 – 8.39 (m, 2H), 7.97 (dd, J = 8.0, 1.4 Hz, 1H), 7.75 – 7.70 (m, 2H), 7.69 – 7.65 (m, 2H), 7.62 – 7.54 (m, 1H), 7.47 (dd, J = 10.3, 4.8 Hz, 2H), 7.41 – 7.33 (m, 3H), 3.79 (s, 3H). **^{13}C NMR** (100 MHz, $CDCl_3$) δ 154.82, 153.69, 143.01, 140.66, 135.05, 133.38, 133.21, 130.49, 130.33, 130.06, 128.85, 127.69, 127.24, 126.83, 123.80, 113.62, 29.35.

3-(4-(tert-butyl)phenyl)-1-methylquinoxalin-2(1H)-one (7t)

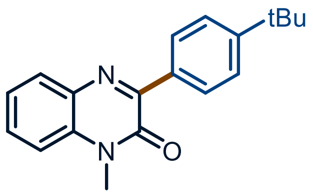

According to the *general procedure F*. The spectral data is consistent with the literature data.[7]

Yellow oil (39.7 mg, 68%).

R_f 0.70 (Petroleum ether/EtOAc, 20/1).

^{1}H NMR (400 MHz, $CDCl_3$) δ 8.24 (d, J = 8.4 Hz, 2H), 7.92 (d, J = 7.4 Hz, 1H), 7.52 (dd, J = 16.8, 7.9 Hz, 3H), 7.41 – 7.27 (m, 2H), 3.75 (s, 3H), 1.36 (s, 9H). **^{13}C NMR** (100 MHz, $CDCl_3$) δ 154.79, 154.19, 153.63, 133.36, 133.31, 133.21, 130.37, 130.05, 129.31, 125.13, 123.65, 113.54, 34.87, 31.24, 29.28.

3-(4-fluorophenyl)-1-methylquinoxalin-2(1H)-one (7u)

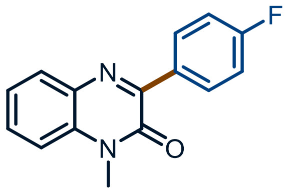

According to the *general procedure F*. The spectral data is consistent with the literature data.[7]

Yellow oil (25.4 mg, 50%).

R_f 0.70 (Petroleum ether/EtOAc, 20/1).

^{1}H NMR (400 MHz, $CDCl_3$) δ 8.45 – 8.34 (m, 2H), 7.92 (dd, J = 8.0, 1.1 Hz, 1H), 7.61 – 7.50 (m, 1H), 7.41 – 7.29 (m, 2H), 7.15 (t, J = 8.7 Hz, 2H), 3.76 (s, 3H). **^{13}C NMR** (100 MHz, $CDCl_3$) δ 165.45, 162.96, 154.66, 152.74, 140.82, 133.31, 133.00, 132.21, 131.89, 131.81, 130.40, 123.84, 115.18, 114.97, 113.63, 29.34.

1-methyl-3-(o-tolyl)quinoxalin-2(1H)-one (7v)

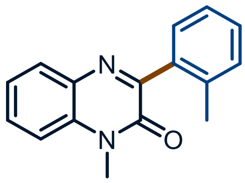

According to the *general procedure F*. The spectral data is consistent with the literature data.[7]

Yellow oil (21.0 mg, 42%).

R_f 0.70 (Petroleum ether/EtOAc, 20/1).

^{1}H NMR (400 MHz, $CDCl_3$) δ 7.93 (d, J = 7.3 Hz, 1H), 7.61 (t, J = 7.3 Hz, 1H), 7.46 (d, J = 7.1 Hz, 1H), 7.41 – 7.27 (m, 5H), 3.78 (s, 3H), 2.35 (s, 3H). **^{13}C NMR** (100

MHz, $CDCl_3$) δ 158.44, 154.57, 136.85, 136.14, 133.60, 132.90, 130.58, 130.55, 130.47, 129.36, 129.19, 125.63, 123.76, 113.71, 29.40, 19.95.

1-(benzyloxy)-4-(1,1-difluoro-3-phenylprop-1-en-2-yl)benzene (9a)

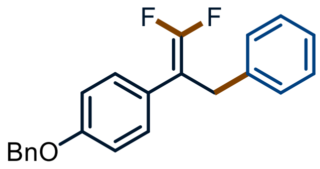

According to the *general procedure G*. The spectral data is consistent with the literature data.[8]
Yellow oil (55.7 mg, 83%).
R_f 0.70 (Petroleum ether/EtOAc, 20/1).
^{1}H NMR (400 MHz, $CDCl_3$) δ 7.32 – 7.02 (m, 11H), 6.84 – 6.71 (m, 3H), 4.87 (s, 2H), 3.61 (s, 2H). **^{13}C NMR** (100 MHz, $CDCl_3$) δ 158.48, 157.15, 154.29, 154.25, 151.39, 138.26, 136.67, 134.74, 129.19, 128.41, 128.32, 128.11, 127.81, 127.34, 126.24, 120.85, 114.86, 113.59, 91.65, 91.52, 91.45, 91.31, 69.76, 33.71.
^{19}F NMR (376 MHz, $CDCl_3$) δ -89.99 (s), -90.07 (s).

1-(1,1-difluoro-3-phenylprop-1-en-2-yl)-4-methoxybenzene (9b)

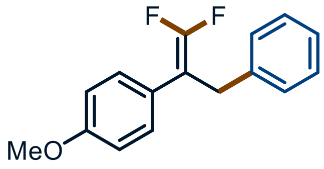

According to the *general procedure G*. The spectral data is consistent with the literature data.[8]
Yellow oil (41.6 mg, 80%).
R_f 0.70 (Petroleum ether/EtOAc, 20/1).
^{1}H NMR (400 MHz, $CDCl_3$) δ ^{1}H NMR (400 MHz, $CDCl_3$) δ 7.19 (dt, J = 18.4, 8.1 Hz, 7H), 6.80 (d, J = 8.8 Hz, 2H), 3.74 (s, 3H), 3.69 (s, 2H). **^{13}C NMR** (100 MHz, $CDCl_3$) δ ^{13}C NMR (101 MHz, $CDCl_3$) δ 158.69, 154.30 (dd, J = 290.5, 286.8 Hz), 138.63, 129.48, 129.45, 129.41, 128.50, 128.47, 128.33, 126.39, 125.74, 125.71, 113.85, 91.19 (dd, J = 21.3, 14.0 Hz), 55.19, 34.03.
^{19}F NMR (376 MHz, $CDCl_3$) δ -91.47 (d, J = 42.9 Hz), -91.88 (d, J = 42.8 Hz).

1-(1,1-difluoro-3-phenylprop-1-en-2-yl)-4-isopropoxybenzene (9c)

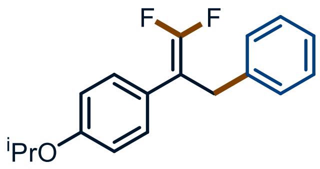

According to the *general procedure G*. The spectral data is consistent with the literature data.[8]
Colorless oil (44.3 mg, 77%).
R_f 0.80 (Petroleum ether/EtOAc, 20/1).
^{1}H NMR (400 MHz, $CDCl_3$) δ7.38 – 7.15 (m, 7H), 6.86 (d, J = 8.6 Hz, 2H), 4.63 – 4.48 (m, 1H), 3.76 (s, 2H), 1.37 (d, J = 6.0 Hz, 5H). **^{13}C NMR** (100 MHz, $CDCl_3$) δ157.20, 157.05, 154.35, 154.31, 151.46, 138.70, 129.46, 129.42, 129.39, 128.50, 128.32, 126.37, 125.43, 115.61, 91.36, 91.23, 91.15, 91.02, 69.81, 34.01, 22.07.
^{19}F NMR (376 MHz, $CDCl_3$) δ -91.41 (d, J = 42.9 Hz), -91.82 (d, J = 42.9 Hz).

1-(1,1-difluoro-3-phenylprop-1-en-2-yl)-4-phenoxybenzene (9d)

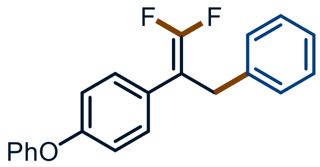

According to the *general procedure G*. The spectral data is consistent with the literature data.[8]
Colorless oil (52.1 mg, 81%).
R_f 0.70 (Petroleum ether/EtOAc, 20/1).
^{1}H NMR (400 MHz, $CDCl_3$) δ 7.34 – 7.28 (m, 2H), 7.27 – 7.13 (m, 7H), 7.08 (t, J = 7.3 Hz, 1H), 6.98 (d, J = 7.3 Hz, 2H), 6.89 (dd, J = 8.7, 2.2 Hz, 2H), 3.70 (s, 2H). **^{13}C NMR** (100 MHz, $CDCl_3$) δ 157.31, 156.79, 156.58, 154.45, 154.41, 151.56, 138.47, 129.82, 129.71, 129.68, 129.64, 128.57, 128.32, 126.49, 123.58, 119.26, 118.43, 91.32, 91.18, 91.11, 90.97, 34.01.
^{19}F NMR (376 MHz, $CDCl_3$) δ -90.64 (d, J = 41.3 Hz), -91.10 (d, J = 41.2 Hz).

4-(1,1-difluoro-3-phenylprop-1-en-2-yl)phenyl 4-methylbenzenesulfonate (9e)

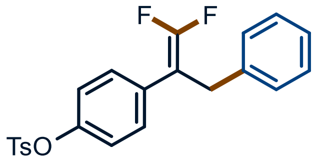

According to the *general procedure G*. The spectral data is consistent with the literature data.[8]
Yellow oil (68.0 mg, 85%).
R_f 0.30 (Petroleum ether/EtOAc, 20/1).
^{1}H NMR (400 MHz, $CDCl_3$) δ 7.64 (d, J = 7.2 Hz, 2H), 7.27 – 7.20 (m, 4H), 7.20 – 7.12 (m, 3H), 7.09 (d, J = 6.8 Hz, 2H), 6.88 (d, J = 7.2 Hz, 2H), 3.67 (s, 2H), 2.42 (s, 3H). **^{13}C NMR** (100 MHz, $CDCl_3$) δ 157.34, 154.48, 154.44, 151.57, 148.53, 145.42, 138.01, 132.55, 132.31, 129.76, 129.55, 129.52, 129.48, 128.57, 128.49, 128.24, 126.58, 122.34, 91.07, 90.94, 90.85, 90.72, 33.81, 21.72.

^{19}**F NMR** (376 MHz, $CDCl_3$) δ -89.60 (d, J = 39.0 Hz), -90.16 (d, J = 39.0 Hz).

2-(4-(1,1-difluoro-3-phenylprop-1-en-2-yl)phenoxy)tetrahydro-2H-pyran (9f)

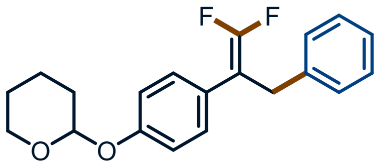

According to the *general procedure G*. The spectral data is consistent with the literature data.[8]
Colorless oil (45.5 mg, 69%).
R_f 0.70 (Petroleum ether/EtOAc, 10/1).
^{1}**H NMR** (400 MHz, $CDCl_3$) δ 7.24 (dd, J = 8.7, 5.7 Hz, 2H), 7.16 (t, J = 7.7 Hz, 5H), 6.96 (d, J = 8.8 Hz, 2H), 5.37 (t, J = 3.1 Hz, 1H), 3.93 – 3.81 (m, 1H), 3.69 (s, 2H), 3.58 (dt, J = 9.9, 3.3 Hz, 1H), 2.06 – 1.93 (m, 1H), 1.83 (dt, J = 8.7, 4.9 Hz, 2H), 1.62 (ddd, J = 21.7, 8.1, 3.2 Hz, 3H). ^{13}**C NMR** (100 MHz, $CDCl_3$) δ 157.19, 156.18, 154.34, 154.30, 151.44, 138.62, 129.62, 129.37, 129.33, 129.30, 128.47, 128.31, 126.60, 126.36, 116.29, 115.32, 96.35, 91.36, 91.22, 91.14, 91.01, 62.13, 33.98, 30.35, 25.19, 18.80.
^{19}**F NMR** (376 MHz, $CDCl_3$) δ -91.27 (d, J = 42.0 Hz), -91.68 (d, J = 42.3 Hz).

4-(1,1-difluoro-3-phenylprop-1-en-2-yl)-N,N-diphenylaniline (9g)

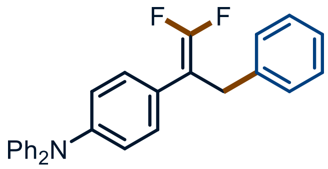

According to the *general procedure G*. The spectral data is consistent with the literature data.[8]
Yellow oil (58.7 mg, 74%).
R_f 0.50 (Petroleum ether/EtOAc, 20/1).
^{1}**H NMR** (400 MHz, $CDCl_3$) δ 7.33 – 7.21 (m, 9H), 7.18 (d, J = 8.5 Hz, 2H), 7.15 – 6.95 (m, 8H), 3.76 (s, 2H). ^{13}**C NMR** (100 MHz, $CDCl_3$) δ 157.40, 154.55, 154.50, 151.64, 147.53, 146.82, 138.72, 129.30, 128.92, 128.88, 128.85, 128.53, 128.26, 127.13, 126.40, 124.61, 123.10, 122.93, 91.31, 91.17, 91.09, 90.96, 33.79.
^{19}**F NMR** (376 MHz, $CDCl_3$) δ -90.33 (d, J = 40.7 Hz), -90.76 (d, J = 41.1 Hz).

4-(1,1-difluoro-3-phenylprop-1-en-2-yl)-1,1'-biphenyl (9h)

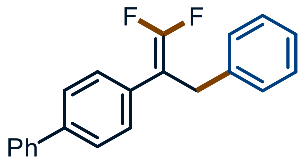

According to the *general procedure G*. The spectral data is consistent with the literature data.[8]

Colorless oil (45.9 mg, 75%).

R_f 0.50 (Petroleum ether/EtOAc, 40/1).

^{1}H NMR (400 MHz, $CDCl_3$) δ 7.65 – 7.54 (m, 4H), 7.48 (dd, J = 11.2, 3.9 Hz, 2H), 7.40 (dd, J = 10.5, 4.7 Hz, 3H), 7.32 (dd, J = 10.6, 4.3 Hz, 2H), 7.27 (t, J = 6.6 Hz, 3H), 3.83 (s, 2H). **^{13}C NMR** (100 MHz, $CDCl_3$) δ 157.49, 154.62, 154.58, 151.72, 140.52, 140.05, 138.51, 132.53, 128.82, 128.66, 128.62, 128.59, 128.31, 127.55, 127.43, 127.09, 127.01, 126.50, 91.59, 91.46, 91.38, 91.25, 33.80.

^{19}F NMR (376 MHz, $CDCl_3$) δ -89.60 (d, J = 39.0 Hz), -90.16 (d, J = 39.0 Hz).

1-(4-(1,1-difluoro-3-phenylprop-1-en-2-yl)phenyl)naphthalene (9i)

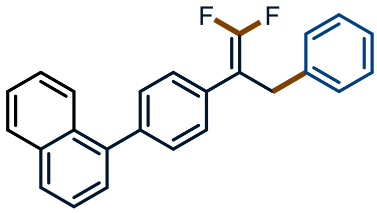

According to the *general procedure G*. The spectral data is consistent with the literature data.[8]

Yellow oil (46.9 mg, 66%).

R_f 0.70 (Petroleum ether/EtOAc, 30/1).

^{1}H NMR (400 MHz, $CDCl_3$) δ 7.98 (s, 1H), 7.91 – 7.79 (m, 3H), 7.74 – 7.58 (m, 3H), 7.52 – 7.36 (m, 4H), 7.31 – 7.14 (m, 5H), 3.77 (s, 2H). **^{13}C NMR** (100 MHz, $CDCl_3$) δ 157.53, 154.67, 154.63, 151.76, 139.94, 138.53, 137.85, 133.71, 132.73, 132.63, 128.77, 128.74, 128.70, 128.63, 128.52, 128.35, 128.26, 127.70, 127.36, 126.54, 126.39, 126.06, 125.74, 125.36, 91.66, 91.52, 91.44, 91.31, 33.82.

^{19}F NMR (376 MHz, $CDCl_3$) δ -89.51 (d, J = 38.8 Hz), -90.03 (d, J = 38.9 Hz).

2-(4-(1,1-difluoro-3-phenylprop-1-en-2-yl)phenyl)naphthalene (9j)

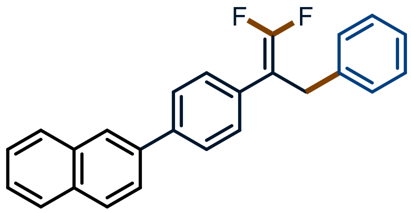

According to the *general procedure G*. The spectral data is consistent with the literature data.[8]
Black oil (42.0 mg, 59%).
R_f 0.50 (Petroleum ether/EtOAc, 10/1).
^{1}H NMR (400 MHz, $CDCl_3$) δ 7.87 (d, *J* = 8.2 Hz, 2H), 7.83 (d, *J* = 8.2 Hz, 1H), 7.52 – 7.44 (m, 2H), 7.39 (q, *J* = 7.4 Hz, 6H), 7.32 – 7.17 (m, 5H), 3.81 (s, 2H). **^{13}C NMR** (100 MHz, $CDCl_3$) δ 157.61, 154.75, 154.70, 151.84, 139.73, 139.68, 138.58, 133.85, 132.58, 131.54, 130.12, 128.64, 128.34, 128.13, 128.10, 128.06, 127.79, 126.97, 126.53, 126.12, 125.96, 125.85, 125.41, 91.64, 91.51, 91.42, 91.29, 33.88.
^{19}F NMR (376 MHz, $CDCl_3$) δ -89.37 (d, *J* = 38.9 Hz), -90.06 (d, *J* = 38.8 Hz).

4-(1,1-difluoro-3-phenylprop-1-en-2-yl)-4'-ethyl-1,1'-biphenyl (9k)

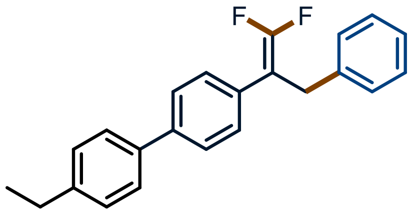

According to the *general procedure G*. The spectral data is consistent with the literature data.[8]
Colorless oil (34.7 mg, 52%).
R_f 0.80 (Petroleum ether/EtOAc, 20/1).
^{1}H NMR (400 MHz, $CDCl_3$) δ 7.62 – 7.52 (m, 4H), 7.41 (d, *J* = 7.8 Hz, 2H), 7.37 – 7.17 (m, 7H), 3.84 (s, 2H), 2.76 (q, *J* = 7.5 Hz, 2H), 1.34 (t, *J* = 7.6 Hz, 3H). **^{13}C NMR** (100 MHz, $CDCl_3$) δ 157.49, 154.63, 154.58, 151.72, 143.60, 140.02, 138.56, 137.89, 132.22, 128.88, 128.61, 128.59, 128.54, 128.36, 128.32, 127.56, 127.11, 126.93, 126.92, 126.49, 91.61, 91.48, 91.40, 91.27, 33.82, 28.57, 15.59.
^{19}F NMR (376 MHz, $CDCl_3$) δ -89.67 (d, *J* = 39.3 Hz), -90.22 (d, *J* = 39.2 Hz).

2-(1,1-difluoro-3-phenylprop-1-en-2-yl)-1,1'-biphenyl (9l)

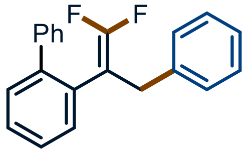

According to the *general procedure G*. The spectral data is consistent with the literature data.[8]
Yellow oil (47.1 mg, 77%).
R_f 0.50 (Petroleum ether/EtOAc, 30/1).
^{1}H NMR (400 MHz, $CDCl_3$) δ 7.41 – 7.27 (m, 5H), 7.19 (dddd, *J* = 20.2, 14.2, 5.2, 3.1 Hz, 6H), 7.02 (d, *J* = 7.6 Hz, 1H), 6.93 – 6.85 (m, 2H), 3.15 (s, 2H). **^{3}C NMR**

(100 MHz, $CDCl_3$) δ 156.45, 153.60, 150.74, 141.74, 141.22, 138.25, 131.16, 130.26, 128.77, 128.74, 128.28, 128.23, 128.05, 127.19, 127.12, 126.34, 92.66, 92.49, 92.44, 92.28, 34.50.
^{19}F NMR (376 MHz, $CDCl_3$) δ -90.58 (d, J = 42.4 Hz), -93.54 (d, J = 42.2 Hz).

3-(1,1-difluoro-3-phenylprop-1-en-2-yl)-1,1'-biphenyl (9m)

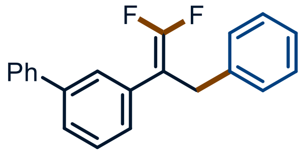

According to the *general procedure G*. The spectral data is consistent with the literature data.[8]
Yellow oil (44.0 mg, 72%).
R_f 0.70 (Petroleum ether/EtOAc, 20/1).
^{1}H NMR (400 MHz, $CDCl_3$) δ 7.51 – 7.45 (m, 3H), 7.45 – 7.37 (m, 3H), 7.33 (t, J = 7.2 Hz, 2H), 7.24 (dd, J = 8.8, 5.8 Hz, 3H), 7.18 (d, J = 7.2 Hz, 3H), 3.76 (s, 2H). **^{13}C NMR** (100 MHz, $CDCl_3$) δ 157.48, 154.62, 154.58, 151.72, 141.39, 140.92, 138.52, 134.10, 128.88, 128.84, 128.62, 128.43, 127.49, 127.29, 127.26, 127.22, 126.55, 126.25, 92.05, 91.92, 91.84, 91.71, 34.09.
^{19}F NMR (376 MHz, $CDCl_3$) δ -90.07 (d, J = 39.8 Hz), -90.43 (d, J = 39.6 Hz).

4-(1,1-difluoro-3-phenylprop-1-en-2-yl)benzonitrile (9n)

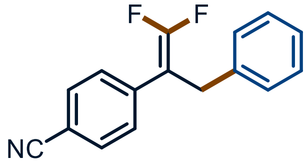

According to the *general procedure G*. The spectral data is consistent with the literature data.[8]
Colorless oil (27.0 mg, 53%).
R_f 0.80 (Petroleum ether/EtOAc, 50/1).
^{1}H NMR (400 MHz, $CDCl_3$) δ 7.51 – 7.44 (m, 2H), 7.35 (s, 1H), 7.28 (d, J = 7.8 Hz, 2H), 7.16 (d, J = 7.0 Hz, 2H), 7.05 (d, J = 6.8 Hz, 2H), 3.67 (s, 2H). **^{13}C NMR** (100 MHz, $CDCl_3$) δ 157.73, 154.85, 154.80, 151.92, 138.48, 137.51, 132.18, 128.92, 128.89, 128.85, 128.76, 128.15, 126.83, 118.60, 111.03, 91.37, 91.24, 91.14, 91.03, 33.38.
^{19}F NMR (376 MHz, $CDCl_3$) δ -86.87 (d, J = 32.6 Hz), -87.56 (d, J = 32.6 Hz).

1-(benzyloxy)-3-(1,1-difluoro-3-phenylprop-1-en-2-yl)benzene (9o)

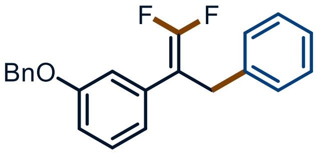

According to the *general procedure G*. The spectral data is consistent with the literature data.[8]
Yellow oil (45.0 mg, 67%).
R_f 0.70 (Petroleum ether/EtOAc, 20/1).
^{1}H NMR (400 MHz, $CDCl_3$) δ 7.40 – 7.28 (m, 5H), 7.18 (ddd, J = 22.3, 14.6, 7.1 Hz, 6H), 6.90 – 6.80 (m, 3H), 4.95 (s, 2H), 3.69 (s, 2H). **^{13}C NMR** (100 MHz, $CDCl_3$) δ 158.72, 157.39, 154.53, 154.49, 151.63, 138.50, 136.91, 134.98, 129.43, 128.65, 128.56, 128.35, 128.05, 127.58, 126.48, 121.09, 115.10, 113.83, 91.89, 91.76, 91.69, 91.55, 70.00, 33.95.
^{19}F NMR (376 MHz, $CDCl_3$) δ -89.94 (d, J = 39.0 Hz), -90.12 (d, J = 39.2 Hz).

5'-(1,1-difluoro-3-phenylprop-1-en-2-yl)-1,1':3',1''-terphenyl (9p)

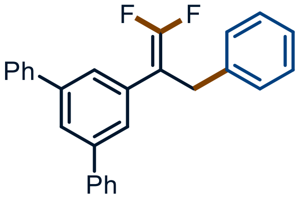

According to the *general procedure G*. The spectral data is consistent with the literature data.[8]
Yellow oil (58.8 mg, 77%).
R_f 0.30 (Petroleum ether/EtOAc, 20/1).
^{1}H NMR (400 MHz, $CDCl_3$) δ 7.58 (s, 1H), 7.50 – 7.45 (m, 4H), 7.41 – 7.32 (m, 6H), 7.28 (t, J = 7.3 Hz, 2H), 7.22 – 7.11 (m, 5H), 3.74 (s, 2H). **^{13}C NMR** (100 MHz, $CDCl_3$) δ 157.51, 154.65, 154.61, 151.75, 141.89, 140.86, 138.45, 128.83, 128.61, 128.46, 127.57, 127.27, 126.56, 126.21, 125.25, 92.08, 91.94, 91.87, 91.73, 34.14.
^{19}F NMR (376 MHz, $CDCl_3$) δ -89.96 (d, J = 39.0 Hz), -90.15 (d, J = 39.2 Hz).

1-(1,1-difluoro-3-phenylprop-1-en-2-yl)-3,5-dimethoxybenzene (9q)

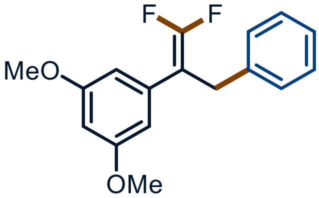

According to the *general procedure G*. The spectral data is consistent with the literature data.[8]
Colorless oil (43.5 mg, 75%).
R_f 0.30 (Petroleum ether/EtOAc, 20/1).
^{1}H NMR (400 MHz, $CDCl_3$) δ7.29 – 7.21 (m, 2H), 7.16 (d, J = 7.0 Hz, 3H), 6.44 – 6.37 (m, 2H), 6.33 (d, J = 1.9 Hz, 1H), 3.69 (d, J = 1.9 Hz, 8H). **^{13}C NMR** (100 MHz,

$CDCl_3$) δ 160.59, 157.33, 154.48, 154.43, 151.58, 138.53, 135.50, 128.52, 128.35, 126.47, 106.74, 106.71, 106.67, 99.40, 92.08, 91.95, 91.87, 91.74, 55.26, 34.02.
^{19}F NMR (376 MHz, $CDCl_3$) δ -89.65 (d, J = 39.2 Hz), -90.06 (d, J = 39.5 Hz).

6-(1,1-difluoro-3-phenylprop-1-en-2-yl)-2,3-dihydrobenzo[b][1,4]dioxine (9r)

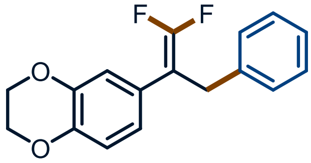

According to the *general procedure G*. The spectral data is consistent with the literature data.[8]
Yellow oil (38.0 mg, 66%).
R_f 0.70 (Petroleum ether/EtOAc, 10/1).
^{1}H NMR (400 MHz, $CDCl_3$) δ 7.35 – 7.18 (m, 5H), 6.85 (d, J = 17.6 Hz, 3H), 4.25 (s, 4H), 3.75 (s, 2H). **^{13}C NMR** (100 MHz, $CDCl_3$) δ 157.29, 154.43, 154.39, 151.54, 143.29, 142.84, 138.56, 128.53, 128.30, 126.69, 126.42, 121.53, 121.49, 121.46, 117.26, 117.23, 117.18, 91.30, 91.17, 91.09, 90.95, 64.37, 64.31, 33.89.
^{19}F NMR (376 MHz, $CDCl_3$) δ -90.90 (d, J = 41.5 Hz), -91.15 (d, J = 41.5 Hz).

5-(1,1-difluoro-3-phenylprop-1-en-2-yl)benzo[d][1,3]dioxole (9s)

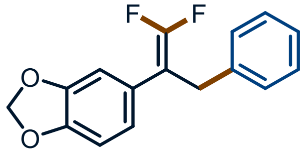

According to the *general procedure G*. The spectral data is consistent with the literature data.[8]
Yellow oil (36.1 mg, 66%).
R_f 0.70 (Petroleum ether/EtOAc, 5/1).
^{1}H NMR (400 MHz, $CDCl_3$) δ ^{1}H NMR (400 MHz, $CDCl_3$) δ 7.33 (t, J = 7.2 Hz, 2H), 7.25 (dd, J = 15.0, 7.3 Hz, 3H), 6.81 (d, J = 8.5 Hz, 3H), 5.99 (s, 2H), 3.75 (s, 2H).
^{13}C NMR (100 MHz, $CDCl_3$) δ 157.19, 154.33, 154.31, 151.44, 147.61, 146.73, 138.40, 128.51, 128.31, 127.16, 126.45, 122.00, 121.97, 121.94, 108.90, 108.87, 108.83, 108.24, 101.09, 91.71, 91.55, 91.50, 91.35, 34.21.
^{19}F NMR (376 MHz, $CDCl_3$) δ -91.15 (d, J = 41.7 Hz), -91.30 (d, J = 41.7 Hz).

4-(1,1-difluoro-3-phenylprop-1-en-2-yl)-1,2-dimethoxybenzene (9t)

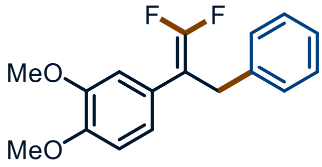

According to the *general procedure G*. The spectral data is consistent with the literature data.[8]
Yellow oil (41.7 mg, 72%).
R_f 0.70 (Petroleum ether/EtOAc, 5/1).
^{1}H NMR (400 MHz, $CDCl_3$) δ 7.29 – 7.10 (m, 5H), 6.42 – 6.35 (m, 2H), 6.33 (d, J = 1.9 Hz, 1H), 3.69 (d, J = 1.9 Hz, 8H). **^{13}C NMR** (100 MHz, $CDCl_3$) δ 160.59, 157.33, 154.48, 154.43, 151.58, 138.53, 135.50, 128.52, 128.35, 126.47, 106.74, 106.71, 106.67, 99.40, 92.08, 91.95, 91.87, 91.74, 55.26, 34.02.
^{19}F NMR (376 MHz, $CDCl_3$) δ -89.65 (d, J = 39.2 Hz), -90.06 (d, J = 39.5 Hz).

4-(1,1-difluoro-3-phenylprop-1-en-2-yl)-2-fluoro-1,1'-biphenyl (9u)

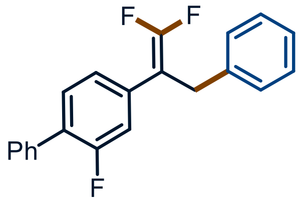

According to the *general procedure G*. The spectral data is consistent with the literature data.[8]
Yellow oil (48.6 mg, 75%).
R_f 0.70 (Petroleum ether/EtOAc, 20/1).
^{1}H NMR (400 MHz, $CDCl_3$) δ 7.55 (d, J = 6.4 Hz, 2H), 7.45 (t, J = 6.3 Hz, 2H), 7.39 (d, J = 7.1 Hz, 2H), 7.30 (d, J = 5.3 Hz, 2H), 7.24 (s, 3H), 7.16 (t, J = 10.5 Hz, 2H), 3.79 (s, 2H). **^{13}C NMR** (100 MHz, $CDCl_3$) δ 160.78, 158.32, 157.72, 154.85, 154.80, 151.93, 138.10, 135.33, 134.63, 130.61, 130.57, 128.97, 128.94, 128.70, 128.51, 128.25, 127.98, 127.82, 127.56, 127.10, 126.67, 124.20, 124.16, 116.07, 115.83, 91.12, 90.98, 90.91, 90.76, 33.55.

^{19}F NMR (376 MHz, $CDCl_3$) δ -89.24 (d, J = 40.5 Hz), -89.73 (d, J = 40.3 Hz), -117.50 – -118.39 (m).

4-(1,1-difluoro-3-phenylprop-1-en-2-yl)-3-fluoro-4'-propyl-1,1'-biphenyl (9v)

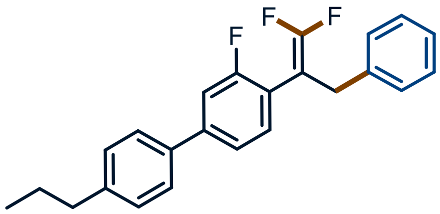

According to the *general procedure G*. The spectral data is consistent with the literature data.[8]
Yellow oil (45.3 mg, 62%).
R_f 0.50 (Petroleum ether/EtOAc, 20/1).

^{1}H NMR (400 MHz, CDCl$_3$) δ 7.50 (d, J = 8.1 Hz, 2H), 7.35 – 7.13 (m, 10H), 3.78 (s, 2H), 2.75 – 2.57 (m, 2H), 1.72 (dd, J = 15.0, 7.5 Hz, 2H), 1.01 (t, J = 7.3 Hz, 3H). **^{13}C NMR** (100 MHz, CDCl$_3$) δ 161.64, 159.18, 156.80, 153.93, 142.96, 142.88, 142.71, 138.14, 136.70, 131.18, 129.05, 128.48, 126.76, 126.52, 122.36, 114.16, 113.92, 86.96, 86.80, 86.72, 86.55, 37.70, 34.07, 24.51, 13.86.

^{19}F NMR (376 MHz, CDCl$_3$) δ -88.17 (dd, J = 36.4, 13.1 Hz), -91.11 (d, J = 36.4 Hz), -113.81 (dd, J = 20.0, 12.3 Hz).

5-(1,1-difluoro-3-phenylprop-1-en-2-yl)-2-fluorobenzonitrile (9w)

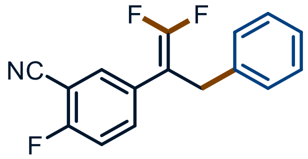

According to the *general procedure G*. The spectral data is consistent with the literature data.[8]

Yellow oil (24.5 mg, 45%).

R_f 0.70 (Petroleum ether/EtOAc, 5/1).

^{1}H NMR (400 MHz, CDCl$_3$) δ 7.52 – 7.38 (m, 2H), 7.34 – 6.97 (m, 7H), 3.71 (s, 2H).

^{13}C NMR (100 MHz, CDCl$_3$) δ 163.30, 160.72, 157.53, 154.65, 154.62, 151.74, 137.12, 135.03, 134.95, 133.23, 130.79, 128.85, 128.21, 126.99, 116.75, 116.56, 113.60, 101.87, 101.71, 90.19, 90.05, 89.95, 89.81, 33.71.

^{19}F NMR (376 MHz, CDCl$_3$) δ -88.45 (d, J = 36.1 Hz), -89.05 (d, J = 36.1 Hz), -107.45 – -108.41 (m).

4-(1,1-difluoro-3-phenylprop-1-en-2-yl)-2-fluoro-1-methoxybenzene (9x)

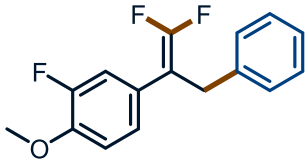

According to the *general procedure G*. The spectral data is consistent with the literature data.[8]

Yellow solid (31.4 mg, 52%).

R_f 0.70 (Petroleum ether/EtOAc, 10/1).

^{1}H NMR (400 MHz, CDCl$_3$) δ 7.33 (t, J = 7.1 Hz, 2H), 7.25 (dd, J = 16.2, 7.0 Hz, 3H), 7.08 (dd, J = 20.2, 10.7 Hz, 2H), 6.93 (t, J = 8.6 Hz, 1H), 3.92 (s, 3H), 3.77 (s, 2H). **^{13}C NMR** (100 MHz, CDCl$_3$) δ 157.33, 154.47, 154.43, 153.24, 151.57, 150.80, 146.80, 146.69, 138.19, 128.59, 128.51, 128.45, 128.25, 126.55, 126.26, 124.23, 124.19, 124.16, 116.18, 116.14, 116.02, 115.99, 113.17, 113.15, 90.89, 90.74, 90.66, 90.53, 56.15, 33.75.

^{19}F NMR (376 MHz, CDCl$_3$) δ -90.31 (d, J = 40.2 Hz), -90.61 (d, J = 40.3 Hz), -135.06 (d, J = 9.0 Hz).

2-(1,1-difluoro-3-phenylprop-1-en-2-yl)naphthalene (9y)

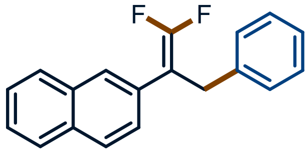

According to the *general procedure G*. The spectral data is consistent with the literature data.[8]

White solid (40.8 mg, 73%). M.p. = 133– 134 °C.

R_f 0.70 (Petroleum ether/EtOAc, 20/1).

^{1}H NMR (400 MHz, $CDCl_3$) δ 7.86 – 7.73 (m, 4H), 7.54 – 7.40 (m, 3H), 7.34 – 7.14 (m, 5H), 3.88 (s, 2H). **^{13}C NMR** (100 MHz, $CDCl_3$) δ 157.54, 154.68, 154.64, 151.78, 138.50, 133.22, 132.46, 130.99, 128.56, 128.36, 128.02, 127.58, 127.55, 126.48, 126.24, 126.17, 92.13, 92.00, 91.92, 91.79, 34.03.

^{19}F NMR (376 MHz, $CDCl_3$) δ -90.44 (d, J = 42.8 Hz), -91.07 (d, J = 42.8 Hz).

2-(1,1-difluoro-3-phenylprop-1-en-2-yl)-6-methoxynaphthalene (9z)

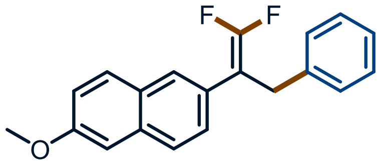

According to the *general procedure G*. The spectral data is consistent with the literature data.[8]

Yellow oil (47.7 mg, 77%).

R_f 0.70 (Petroleum ether/EtOAc, 5/1).

^{1}H NMR (400 MHz, $CDCl_3$) δ 7.69 (t, J = 7.3 Hz, 3H), 7.41 (d, J = 8.6 Hz, 1H), 7.31 – 7.08 (m, 7H), 3.93 (s, 3H), 3.86 (s, 2H). **^{13}C NMR** (100 MHz, $CDCl_3$) δ 157.94, 157.44, 154.58, 154.54, 151.68, 138.60, 133.61, 129.51, 128.70, 128.52, 128.35, 127.33, 127.30, 127.26, 126.84, 126.63, 126.42, 119.09, 105.53, 92.02, 91.88, 91.80, 91.67, 55.33, 34.03.

^{19}F NMR (376 MHz, $CDCl_3$) δ -90.31 (d, J = 40.4 Hz), -91.08 (d, J = 40.5 Hz).

2-(1,1-difluoro-3-phenylprop-1-en-2-yl)-6-ethoxynaphthalene (9aa)

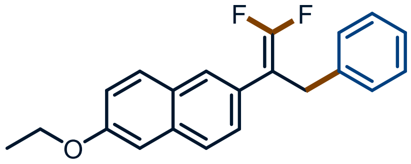

According to the *general procedure G*. The spectral data is consistent with the literature data.[8]
White solid (49.9 mg, 77%). M.p. =134 – 135 °C.
R_f 0.40 (Petroleum ether/EtOAc, 10/1).
^{1}H NMR (400 MHz, $CDCl_3$) δ 7.62 (d, J = 8.5 Hz, 3H), 7.34 (d, J = 8.6 Hz, 1H), 7.27 – 6.95 (m, 7H), 4.10 (q, J = 7.0 Hz, 2H), 3.80 (s, 2H), 1.45 (t, J = 7.0 Hz, 3H). **^{13}C NMR** (100 MHz, $CDCl_3$) δ 157.45, 157.27, 154.59, 154.55, 151.69, 138.63, 133.67, 129.48, 128.64, 128.52, 128.36, 127.32, 127.29, 127.26, 126.81, 126.57, 126.42, 119.38, 106.31, 92.04, 91.91, 91.83, 91.69, 63.52, 34.04, 14.81.
^{19}F NMR (376 MHz, $CDCl_3$) δ -90.34 (d, J = 40.9 Hz), -91.10 (d, J = 40.6 Hz).

2-(1,1-difluoro-3-phenylprop-1-en-2-yl)-9,9-dimethyl-9H-fluorene (9ab)

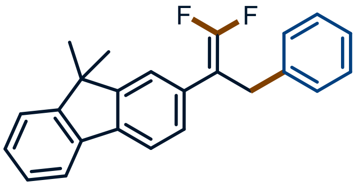

According to the *general procedure G*. The spectral data is consistent with the literature data.[8]
Yellow solid (43.6 mg, 63%). M.p. =120 – 121 °C.
R_f 0.40 (Petroleum ether/EtOAc, 10/1).
^{1}H NMR (400 MHz, $CDCl_3$) δ 7.69 – 7.55 (m, 2H), 7.41 – 7.36 (m, 1H), 7.32 – 7.11 (m, 9H), 3.76 (s, 2H), 1.41 (s, 6H). **^{13}C NMR** (100 MHz, $CDCl_3$) δ 157.34, 154.48, 154.44, 153.82, 153.67, 151.58, 138.76, 138.69, 138.44, 132.49, 128.53, 128.47, 127.39, 127.26, 127.05, 126.47, 122.64, 120.08, 119.90, 92.44, 92.30, 92.24, 92.09, 46.84, 34.29, 27.09.
^{19}F NMR (376 MHz, $CDCl_3$) δ -90.37 (d, J = 40.3 Hz), -90.58 (d, J = 40.5 Hz).

8-(1,1-difluoro-3-phenylprop-1-en-2-yl)quinoline (9ac)

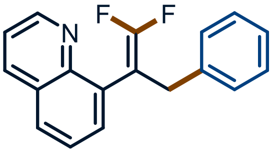

According to the *general procedure G*. The spectral data is consistent with the literature data.[8]
Yellow oil (27.5 mg, 49%).
R_f 0.50 (Petroleum ether/EtOAc, 10/1).
^{1}H NMR (400 MHz, $CDCl_3$) δ 8.99 (d, J = 1.3 Hz, 1H), 8.15 (dd, J = 8.2, 1.1 Hz, 1H), 7.74 (dd, J = 8.1, 1.5 Hz, 1H), 7.47 – 6.99 (m, 8H), 4.01 (s, 2H). **^{13}C NMR** (100 MHz, $CDCl_3$) δ 153.97, 150.05, 146.55, 146.52, 139.00, 136.44, 131.68, 128.76, 128.60, 128.19, 126.16, 126.00, 121.18, 91.50, 91.43, 91.37, 91.27, 34.97.
^{19}F NMR (376 MHz, $CDCl_3$) δ -90.83 (d, J = 42.6 Hz), -92.68 (d, J = 42.7 Hz).

1-(2-(4-(benzyloxy)phenyl)-3,3-difluoroallyl)-3-methylbenzene (9ad)

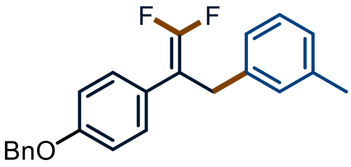

According to the *general procedure G*. The spectral data is consistent with the literature data.[8]

Yellow oil (55.3 mg, 79%).

R_f 0.50 (Petroleum ether/EtOAc, 20/1).

^{1}H NMR (400 MHz, $CDCl_3$) δ 7.36 (dt, J = 15.3, 7.1 Hz, 5H), 7.22 – 7.08 (m, 3H), 6.96 (dd, J = 15.3, 7.5 Hz, 3H), 6.88 (d, J = 8.8 Hz, 2H), 5.01 (s, 2H), 3.65 (s, 2H), 2.28 (s, 3H). **^{13}C NMR** (100 MHz, $CDCl_3$) δ 157.90, 157.21, 154.35, 154.32, 151.46, 138.52, 138.10, 136.91, 129.47, 129.44, 129.40, 129.06, 128.62, 128.37, 128.03, 127.50, 127.16, 126.12, 125.31, 114.74, 91.29, 91.16, 91.08, 90.95, 70.00, 33.91, 21.43.

^{19}F NMR (376 MHz, $CDCl_3$) δ -91.26 (d, J = 42.3 Hz), -91.70 (d, J = 42.6 Hz).

3-benzyl-1,3-dimethylindolin-2-one (11)

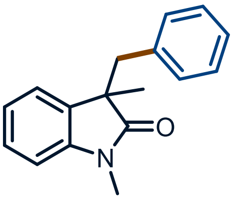

Yellow oil (37.6 mg, 75%).

R_f 0.50 (Petroleum ether/EtOAc, 10/1).

^{1}H NMR (400 MHz, $CDCl_3$) δ 7.35 (ddd, J = 18.6, 12.5, 3.9 Hz, 2H), 7.27 – 7.18 (m, 4H), 7.03 (dd, J = 7.3, 2.1 Hz, 2H), 6.79 (d, J = 7.7 Hz, 1H), 3.32 – 3.15 (m, 5H), 1.66 (s, 3H). **^{13}C NMR** (100 MHz, $CDCl_3$) δ 179.98, 143.15, 136.19, 133.03, 129.84, 127.75, 127.48, 126.42, 123.32, 122.06, 107.75, 49.93, 44.56, 25.89, 22.74.

1,1'-biphenyl-4-*d* (12a)

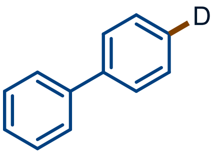

Colorless oil (25.4 mg, 82%).

R_f 0.50 (Petroleum ether/EtOAc, 40/1).

^{1}H NMR (400 MHz, $CDCl_3$) δ 7.62 – 7.57 (m, 4H), 7.47 – 7.41 (m, 4H), 7.35 (d, J = 7.4 Hz, 1H). **^{13}C NMR** (100 MHz, $CDCl_3$) δ 141.27, 128.77, 128.66, 127.26, 127.19.

2-methoxynaphthalene-6-*d* (12b)

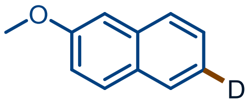

Colorless oil (20.6 mg, 65%).
R_f 0.50 (Petroleum ether/EtOAc, 20/1).
^{1}H NMR (400 MHz, $CDCl_3$) δ 7.89 – 7.81 (m, 3H), 7.54 (d, J = 8.2 Hz, 1H), 7.28 – 7.22 (m, 2H), 4.02 (s, 3H). **^{13}C NMR** (100 MHz, $CDCl_3$) δ 157.63, 134.61, 129.42, 128.99, 127.57, 126.77, 126.29, 118.74, 105.79, 55.31.
.

(phenethylsulfonyl)benzene (14)

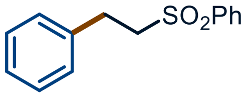

White solid (33.4 mg, 68%). M.p. =120 – 121 °C.
R_f 0.50 (Petroleum ether/EtOAc, 20/1).
^{1}H NMR (400 MHz, $CDCl_3$) δ 7.95 – 7.89 (m, 2H), 7.65 (t, J = 7.4 Hz, 1H), 7.56 (t, J = 7.6 Hz, 2H), 7.24 (dd, J = 7.8, 6.6 Hz, 2H), 7.18 (dd, J = 8.6, 5.9 Hz, 1H), 7.09 (d, J = 7.1 Hz, 2H), 3.38 – 3.30 (m, 2H), 3.07 – 2.99 (m, 2H). **^{13}C NMR** (100 MHz, $CDCl_3$) δ 139.05, 137.46, 133.82, 129.38, 128.83, 128.30, 128.10, 126.95, 57.55, 28.75.

(4-methoxyphenyl)(phenyl)sulfane (16)

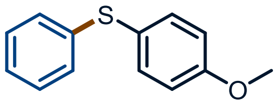

Yellow oil (34.5 mg, 80%).
R_f 0.50 (Petroleum ether/EtOAc, 20/1).
^{1}H NMR (400 MHz, $CDCl_3$) δ 7.49 – 7.43 (m, 2H), 7.28 (dd, J = 10.7, 4.0 Hz, 2H), 7.19 (d, J = 8.5 Hz, 2H), 6.98 – 6.91 (m, 2H), 3.86 (s, 3H). **^{13}C NMR** (100 MHz, $CDCl_3$) δ 159.86, 138.64, 135.42, 128.96, 128.21, 125.78, 124.31, 115.01, 55.40.

phenol (17)

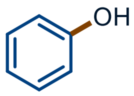

Colorless oil (31.9 mg, 85%).
R_f 0.50 (Petroleum ether/EtOAc, 10/1).
^{1}H NMR (400 MHz, $CDCl_3$) δ 7.20 (td, J = 7.5, 2.0 Hz, 2H), 6.91 (t, J = 7.4 Hz, 1H), 6.88 – 6.79 (m, 2H), 6.30 – 6.05 (m, 1H). **^{13}C NMR** (100 MHz, $CDCl_3$) δ 155.19, 129.91, 121.15, 115.55.

aniline (18)

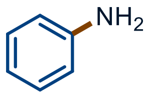

Yellow oil (26.0 mg, 70%).
R_f 0.50 (Petroleum ether/EtOAc, 1/1).
^{1}H NMR (400 MHz, $CDCl_3$) δ 7.19 – 7.04 (m, 2H), 6.76 – 6.67 (m, 1H), 6.58 (d, J = 8.1 Hz, 2H), 3.51 (s, 2H). **^{13}C NMR** (100 MHz, $CDCl_3$) δ 146.75, 129.50, 118.61, 115.31.

benzonitrile (19)

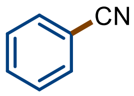

Colorless oil (10.3 mg, 25%).
R_f 0.50 (Petroleum ether/EtOAc, 10/1).
^{1}H NMR (400 MHz, $CDCl_3$) δ 7.61 (ddd, J = 12.5, 7.9, 1.2 Hz, 3H), 7.49 – 7.43 (m, 2H). **^{13}C NMR** (100 MHz, $CDCl_3$) δ 132.83, 132.08, 129.16, 118.84, 112.31.

fluorobenzene (20)

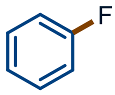

Colorless oil (23.0 mg, 60%).
R_f 0.50 (Petroleum ether).
^{1}H NMR (400 MHz, $CDCl_3$) δ 7.51 (dd, J = 13.9, 7.7 Hz, 2H), 7.31 (t, J = 7.4 Hz, 1H), 7.24 (t, J = 8.8 Hz, 2H). **^{13}C NMR** (100 MHz, $CDCl_3$) δ 164.20, 161.76, 130.06, 130.03, 129.98, 124.06, 124.03, 115.48, 115.27.

oxydibenzene (21)

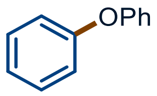

Colorless oil (48.2 mg, 71%).
R_f 0.50 (Petroleum ether/EtOAc, 20/1).
^{1}H NMR (400 MHz, $CDCl_3$) δ 7.28 (t, J = 7.9 Hz, 4H), 7.05 (t, J = 7.4 Hz, 2H), 7.02 – 6.95 (m, 4H). **^{13}C NMR** (100 MHz, $CDCl_3$) δ 157.42, 129.91, 123.37, 119.05.

diphenylamine (22)

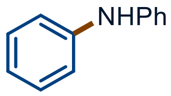

Colorless oil (43.3 mg, 65%).
R_f 0.50 (Petroleum ether/EtOAc, 20/1).
^{1}H NMR (400 MHz, $CDCl_3$) δ 7.54 – 7.39 (m, 4H), 7.34 – 7.18 (m, 4H), 7.18 – 7.03 (m, 2H), 5.82 (s, 1H). **^{13}C NMR** (100 MHz, $CDCl_3$) δ 143.25, 129.47, 129.42, 121.11, 121.07, 117.95, 117.90.

References

[1] M. S. Lowry, J. I. Goldsmith, J. D. Slinker, R. Rohl, R. A. Pascal, G. G. Malliaras, S. Bernhard, *Chem. Mater.* 2005, **17**, 5712.

[2] (a) R. Shintani, M. Takeda, T. Tsuji, T. Hayashi, J. Am. Chem. Soc. 2010, **132**, 13168-13169; (b) C. Gerleve, A. Studer *Angew. Chem. Int. Ed.* 2020, **59**, 15468–15473.

[3] Gao, M.; Li, Y.; Xie, L.; Chauvin, R.; Cui, X., Chem. Commun., 2016, 52, 2846-2849.

[4] F. Yue, H. Ma, H. Song, Y. Liu, J. Dong and Q. Wang. *Chem. Sci.,* 2022, **13**, 13466-13474.

[5] Q. Wei, Y. Lee, W. Liang, X. Chen, B. Mu, X. Cui, W. Wu, S. Bai and Z. Liu, *Nature Communications*, 2022, **13**, 7112

[6] (a)Shi, J.; Guo, L.; Hu, Q.; Liu, Y.; Li, Q. and Pan, F. Photoredox-Catalyzed Difunctionalization of Unactivated Olefins for Synthesizing Lactam-Substituted gem-Difluoroalkenes. *Org. Lett.* **23**, 8822−8827 (2021); (b) Noble, A. and MacMillan, D. W. C. Photoredox α-Vinylation of α-Amino Acids and N-Aryl Amines. *J. Am. Chem. Soc.*, **136**, 11602−11605 (2014).

[7] Zhang, G.; Lv, S.; Shoberu, A. and Zou, J. Copper-Catalyzed TBHP-Mediated Radical Cross-Coupling Reaction of Sulfonylhydrazides with Thiols Leading to Thiosulfonates. *J. Org. Chem.* **82**, 9801−9807 (2017).

[8] (a) Lan, Y.; Yang, F. and Wang, C. Synthesis of gem-Difluoroalkenes via Nickel-Catalyzed Allylic Defluorinative Reductive Cross-Coupling. *ACS Catal.* **8**, 9245−9251 (2018); (b) Yan, S.; Yu, W.; Zhang, J.; Fan, H.; Lu, Z.; Zhang, Z. and Wang, T. Access to gem-Difluoroalkenes via Organic Photoredox-Catalyzed gem-Difluoroallylation of Alkyl Iodides. *J. Org. Chem.* **87**, 1574–1584 (2022); (c) Gladkov, A. A.; Chernov, G. N.; Levin, V. V.; Kokorekin, V. A. and Dilman A. D. Photoredox Activation of Organozinc Reagents: Barbier-Type Reaction of Alkyl Halides with α-(Trifluoromethyl)styrenes. *Org. Lett.* **23**, 9645–9648 (2021); (d) Wang, Q.; Yue, L.; Bao, Y.; Wang, Y.; Kang, D.; Gao, Y. and Yuan, Z. Oxalates as

Activating Groups for Tertiary Alcohols in Photoredox-Catalyzed gem-Difluoroallylation To Construct All-Carbon Quaternary Centers. *J. Org. Chem.* **87**, 8237–8247 (2022); (e) Guo, Y.; Cao, Y.; Song, H.; Liu, Y. and Wang, Q. Photoredox relay-catalyzed gem-difluoroallylation of alkyl iodides Chem. Commun., **57**, 9768-9771 (202); (f) Lin, Z.; Lan, Y. and Wang, C. Reductive Allylic Defluorinative Cross-Coupling Enabled by Ni/Ti Cooperative Catalysis. *Org. Lett.* **21**, 8316–8322 (2019); (g) Jin, Y.; Wu, J.; Lin, Z.; Lan, Y. and Wang C. Merger of C–F and C–N Bond Cleavage in Cross-Electrophile Coupling for the Synthesis of gem-Difluoroalkenes. *Org. Lett.* **22**, 5347–5352 (2020); (h) Liu, Y.; Tao, X.; Mao, Y.; Yuan, X.; Qiu, J.; Kong, L.; Ni, S.; Guo, K.; Wang Y. and Pan Y. Electrochemical C–N bond activation for deaminative reductive coupling of Katritzky salts. *Nat Commun* **12**, 6745 (2021).

NMR Spectra

^{1}H NMR (400 MHz, $CDCl_3$) spectrum of compound **3a**

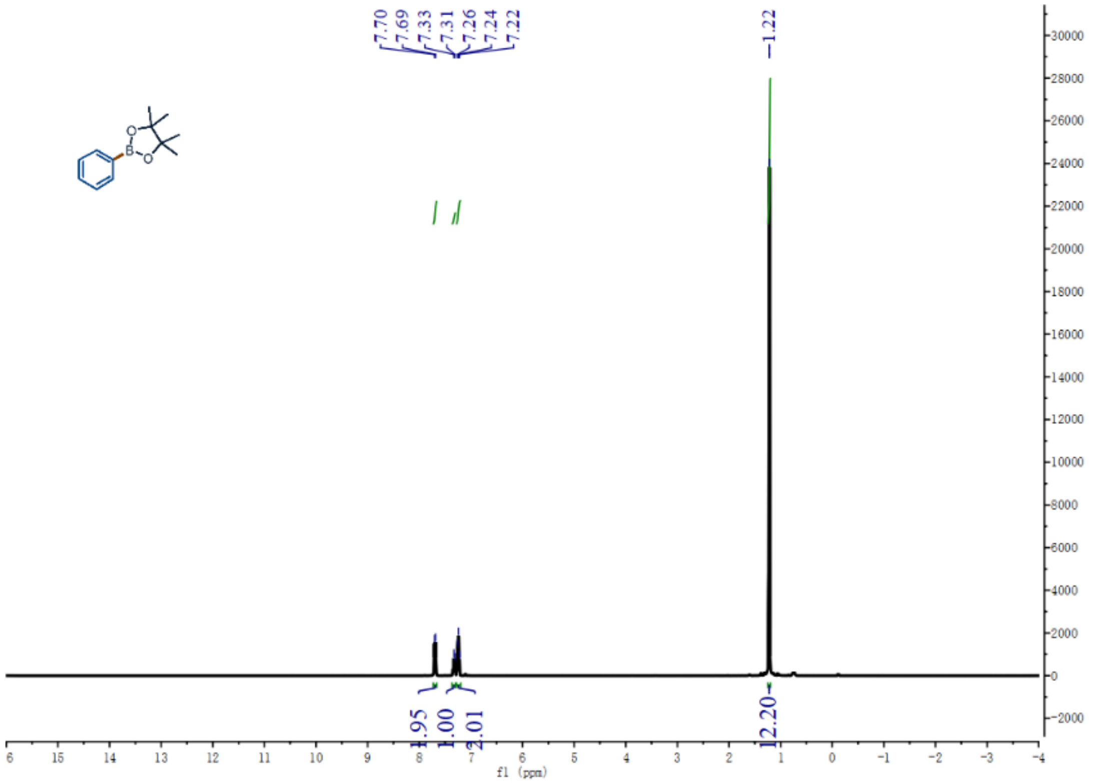

^{13}C NMR (100 MHz, $CDCl_3$) spectrum of compound **3a**

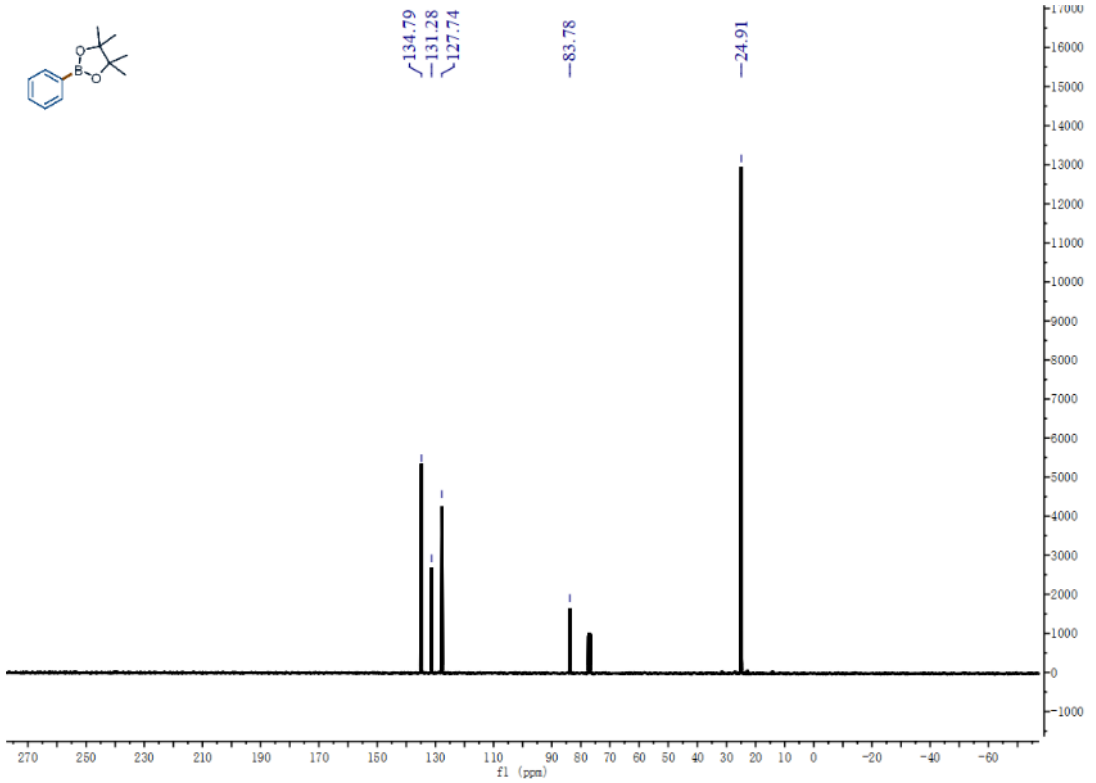

^{11}B NMR (376 MHz, $CDCl_3$) spectrum of compound **3a**

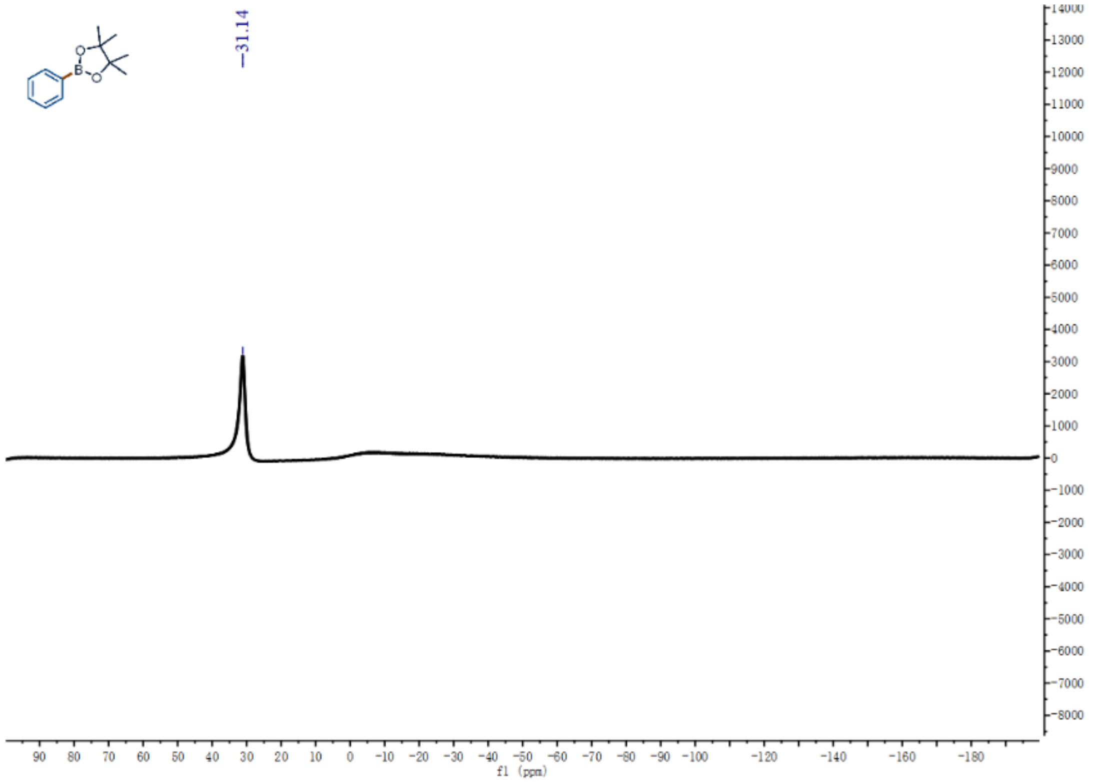

^{1}H NMR (400 MHz, $CDCl_3$) spectrum of compound **3b**

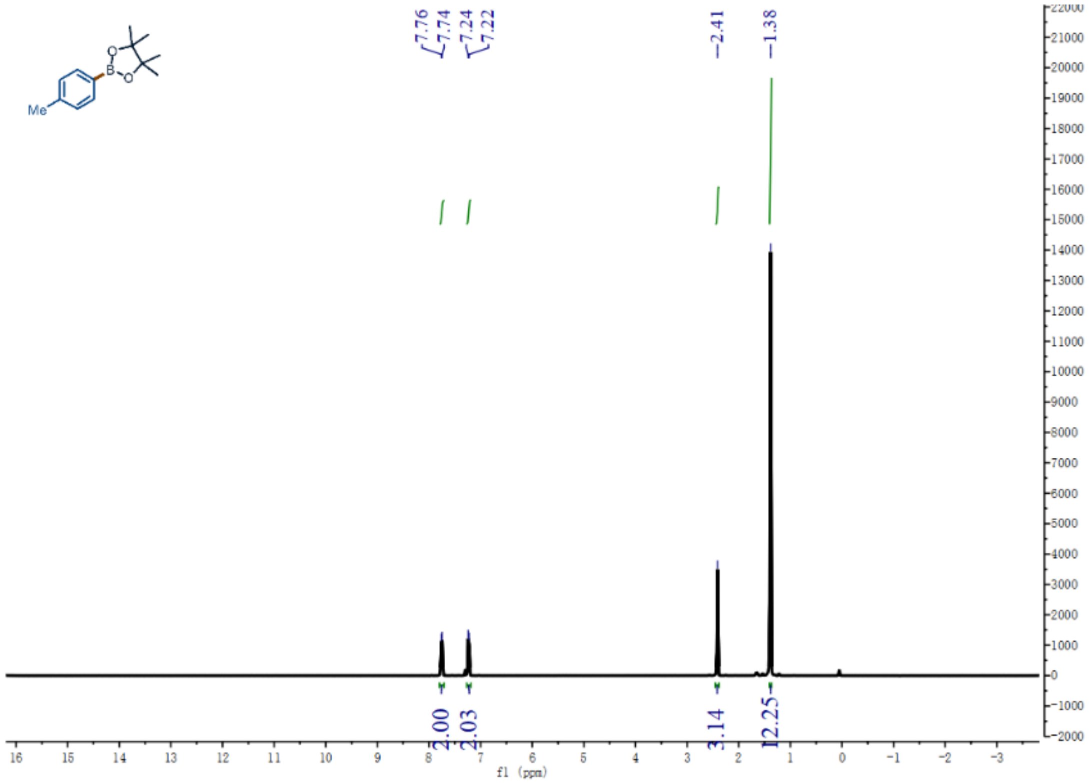

^{13}C NMR (100 MHz, $CDCl_3$) spectrum of compound **3b**

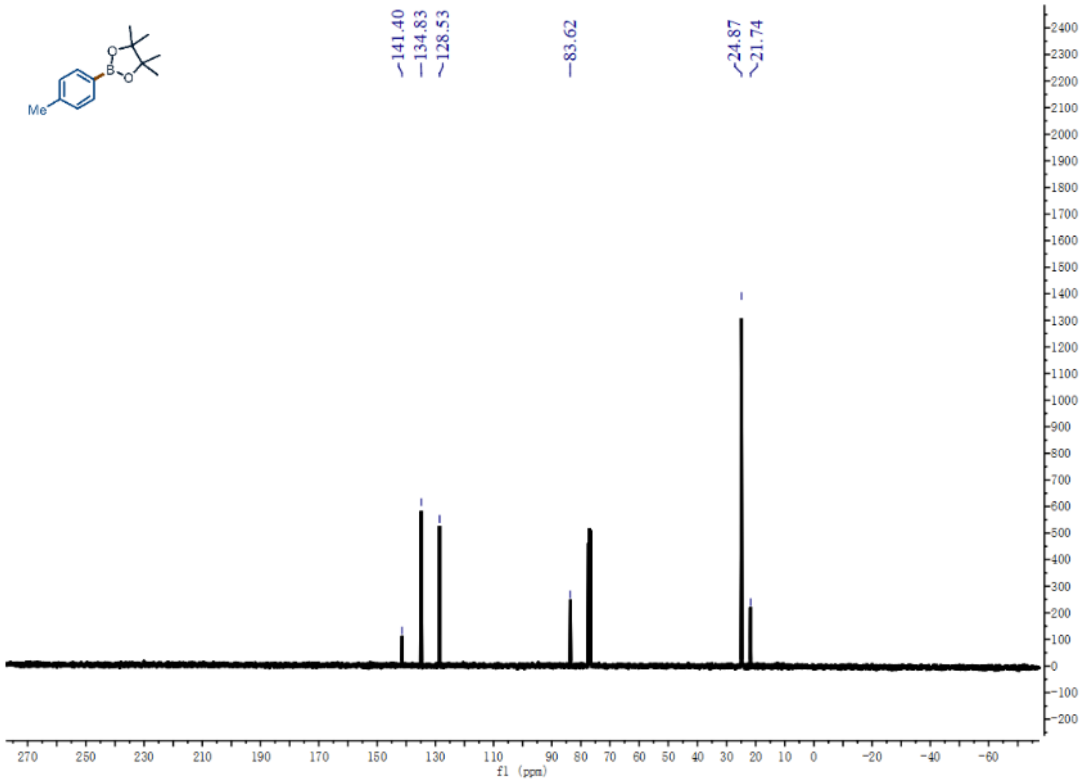

^{11}B NMR (376 MHz, $CDCl_3$) spectrum of compound **3b**

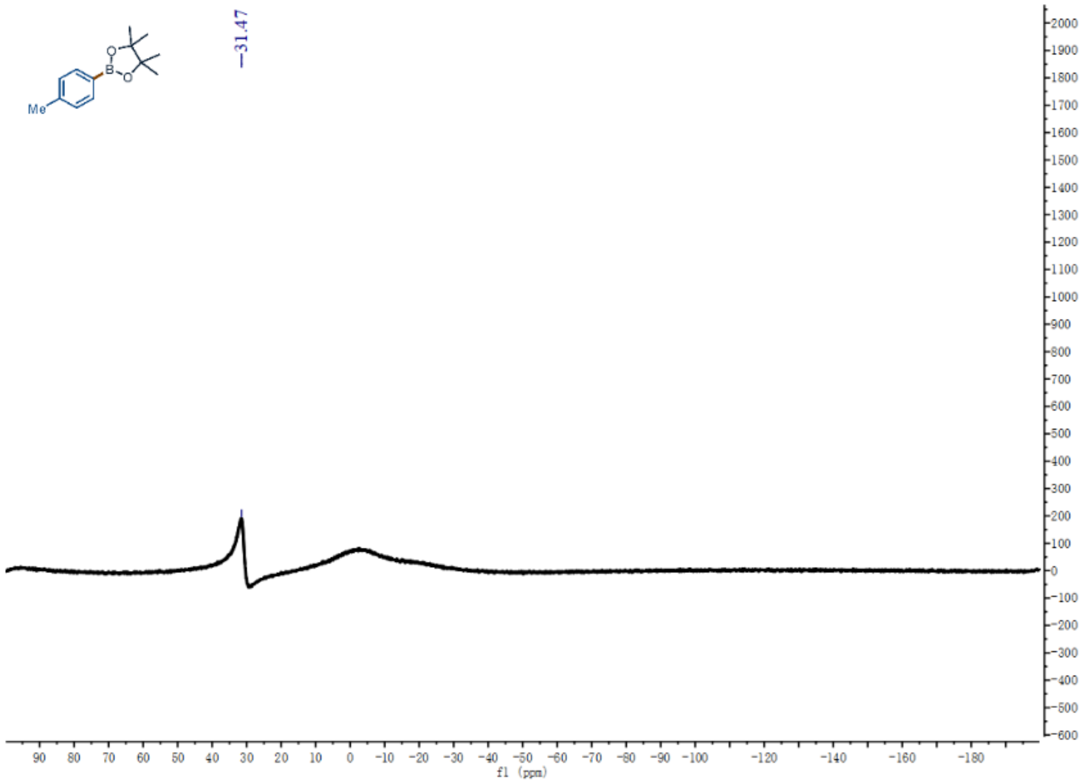

^{1}H NMR (400 MHz, $CDCl_3$) spectrum of compound **3c**

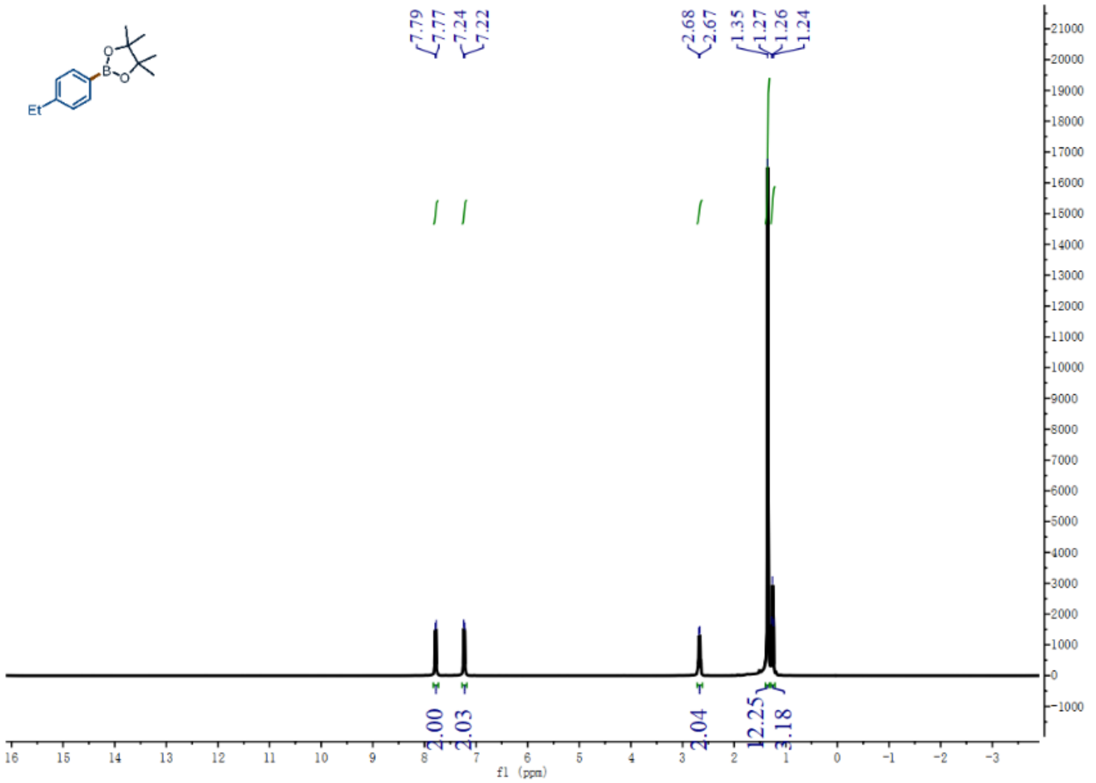

^{13}C NMR (100 MHz, $CDCl_3$) spectrum of compound **3c**

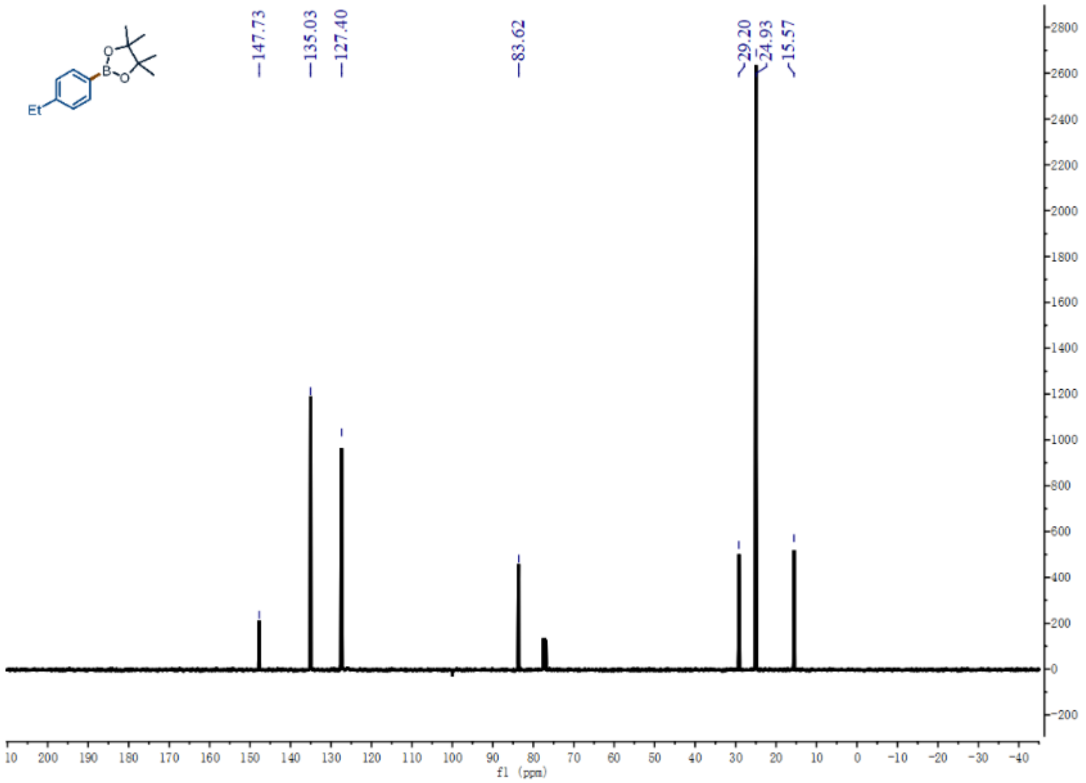

^{11}B NMR (376 MHz, $CDCl_3$) spectrum of compound **3c**

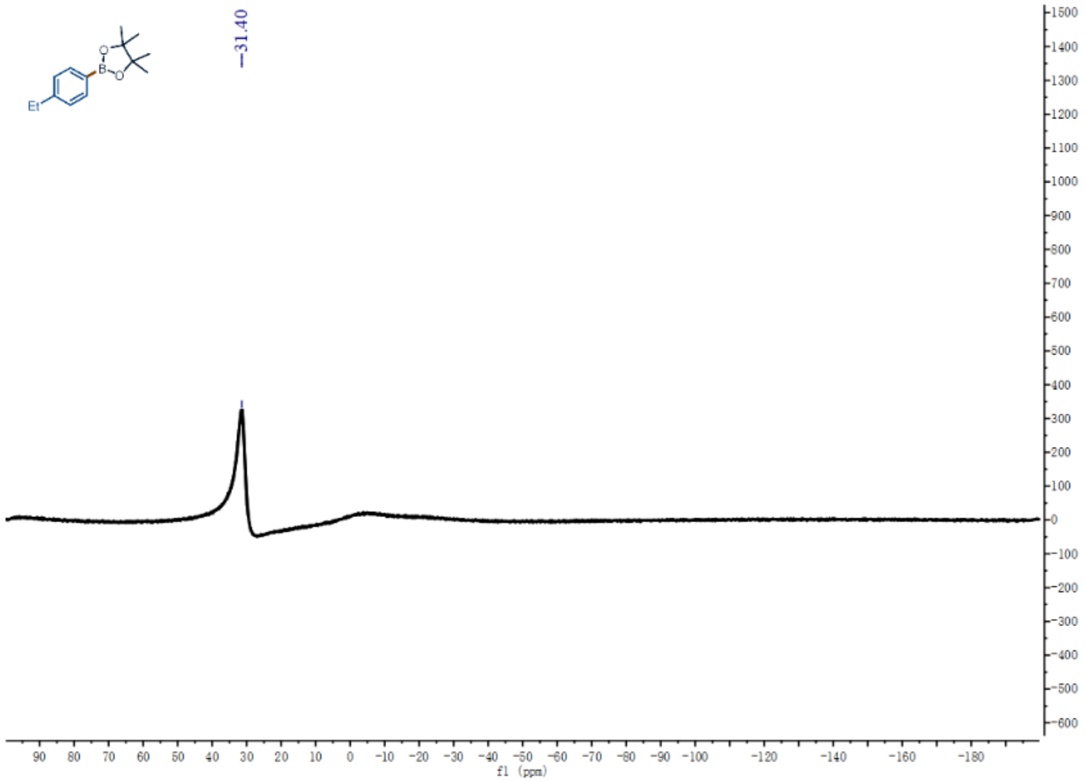

^{1}H NMR (400 MHz, $CDCl_3$) spectrum of compound **3d**

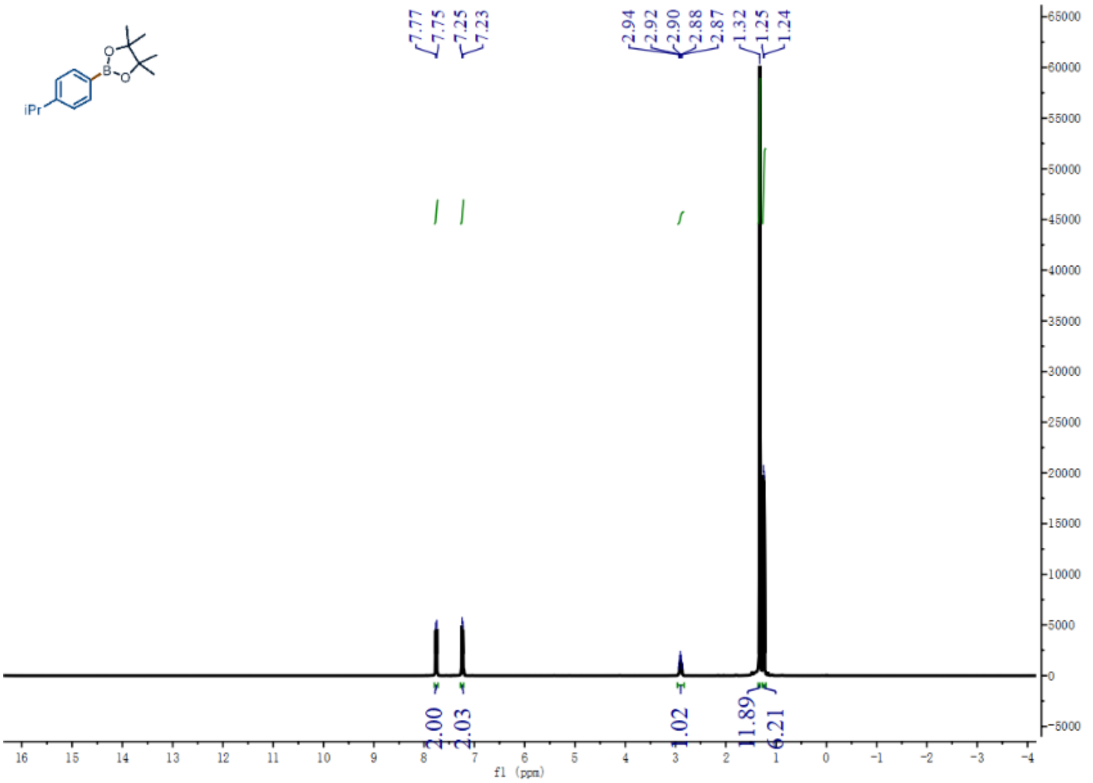

^{13}C NMR (100 MHz, $CDCl_3$) spectrum of compound **3d**

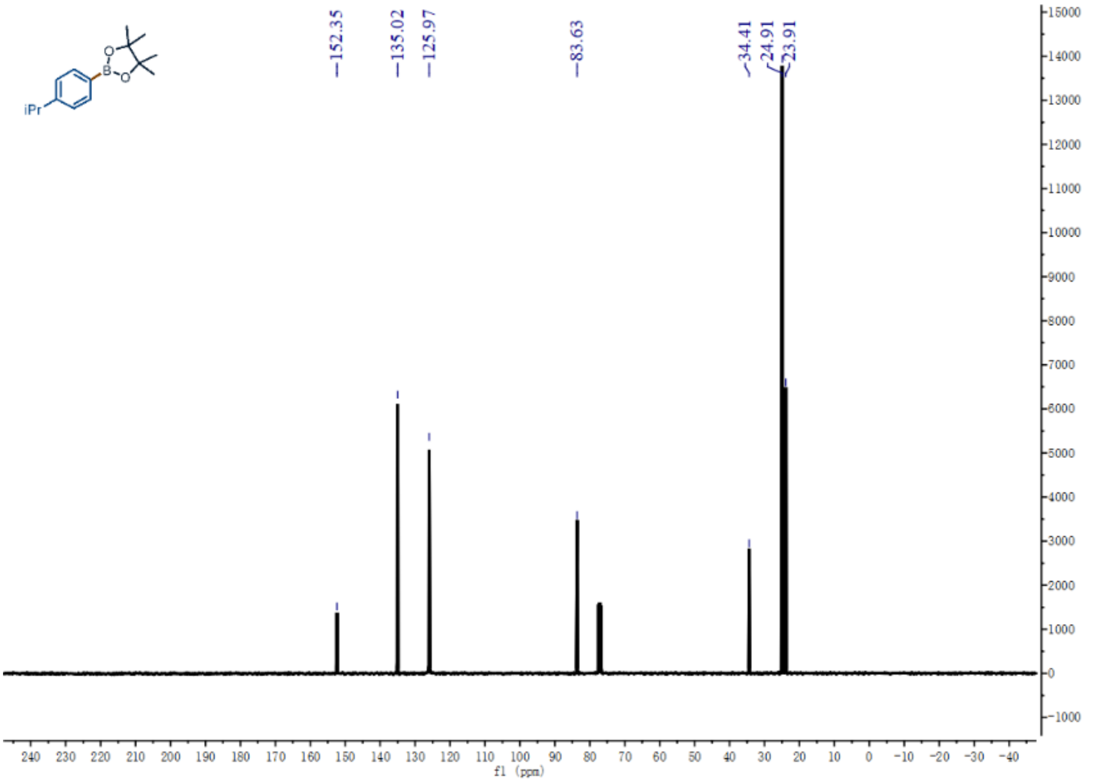

^{11}B NMR (376 MHz, $CDCl_3$) spectrum of compound **3d**

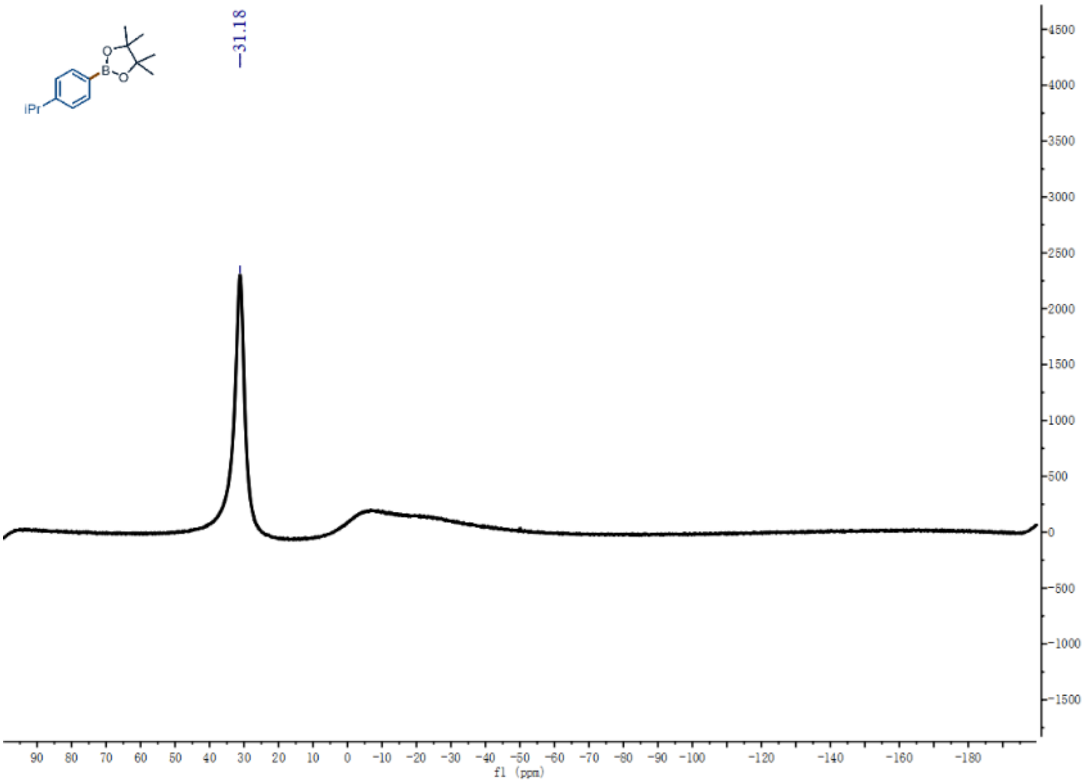

^{1}H NMR (400 MHz, $CDCl_3$) spectrum of compound **3e**

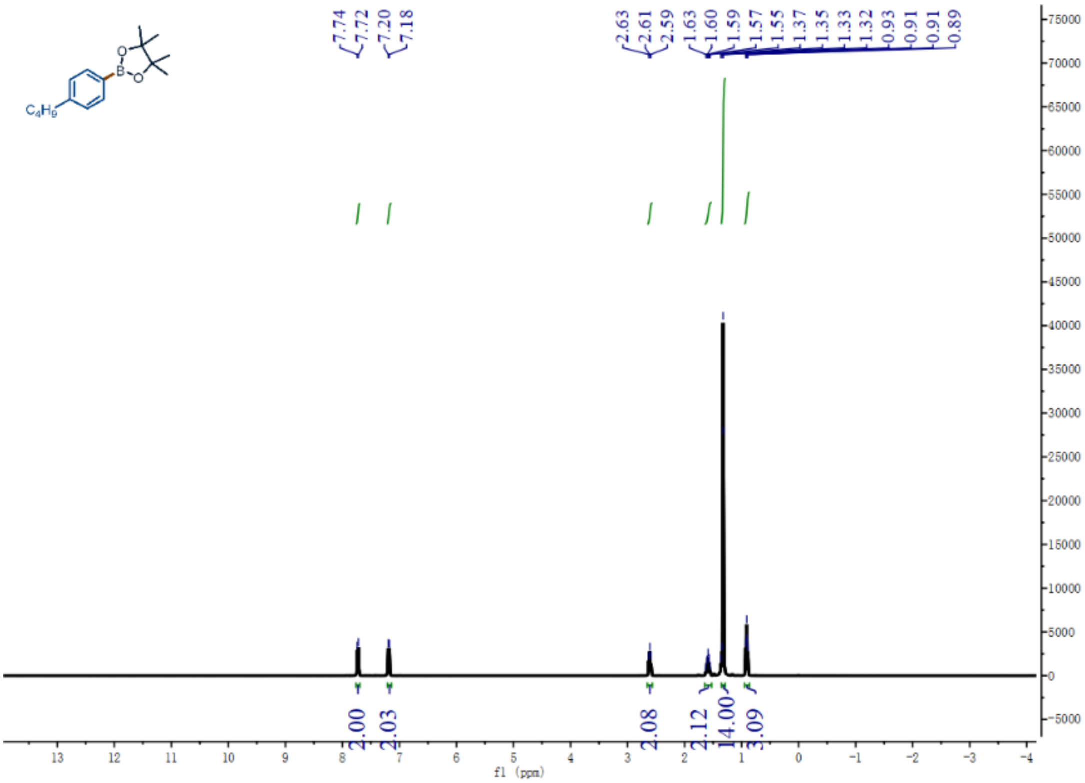

^{13}C NMR (100 MHz, $CDCl_3$) spectrum of compound **3e**

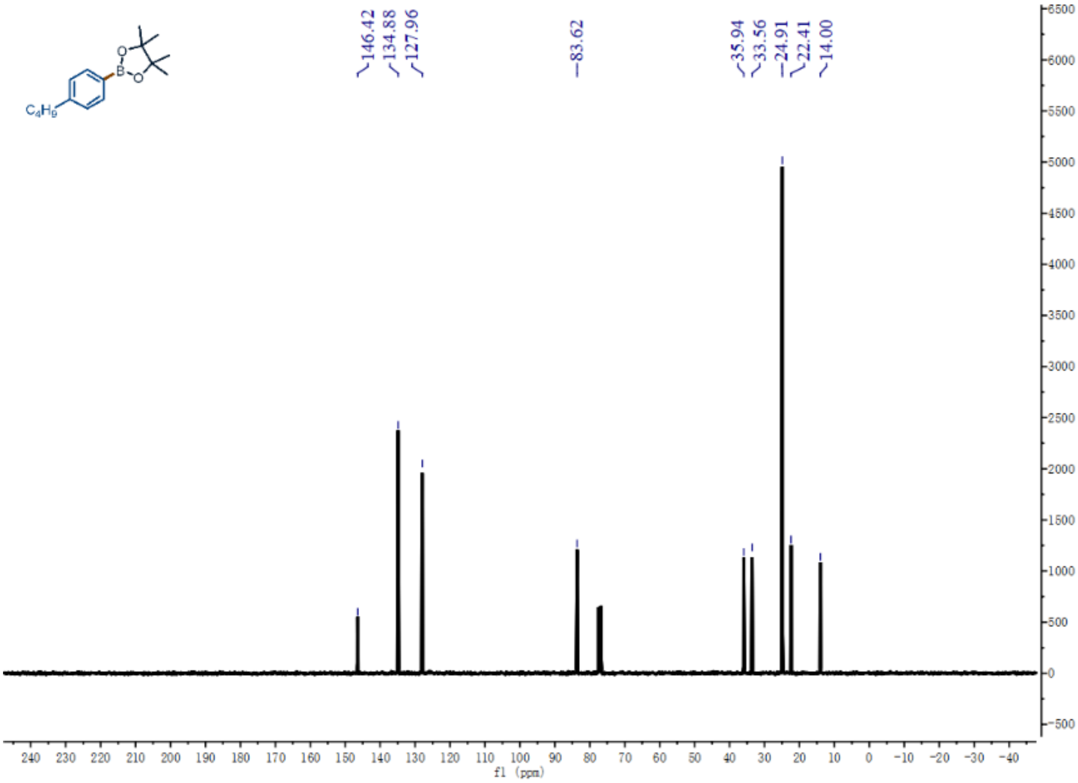

^{11}B NMR (376 MHz, $CDCl_3$) spectrum of compound **3e**

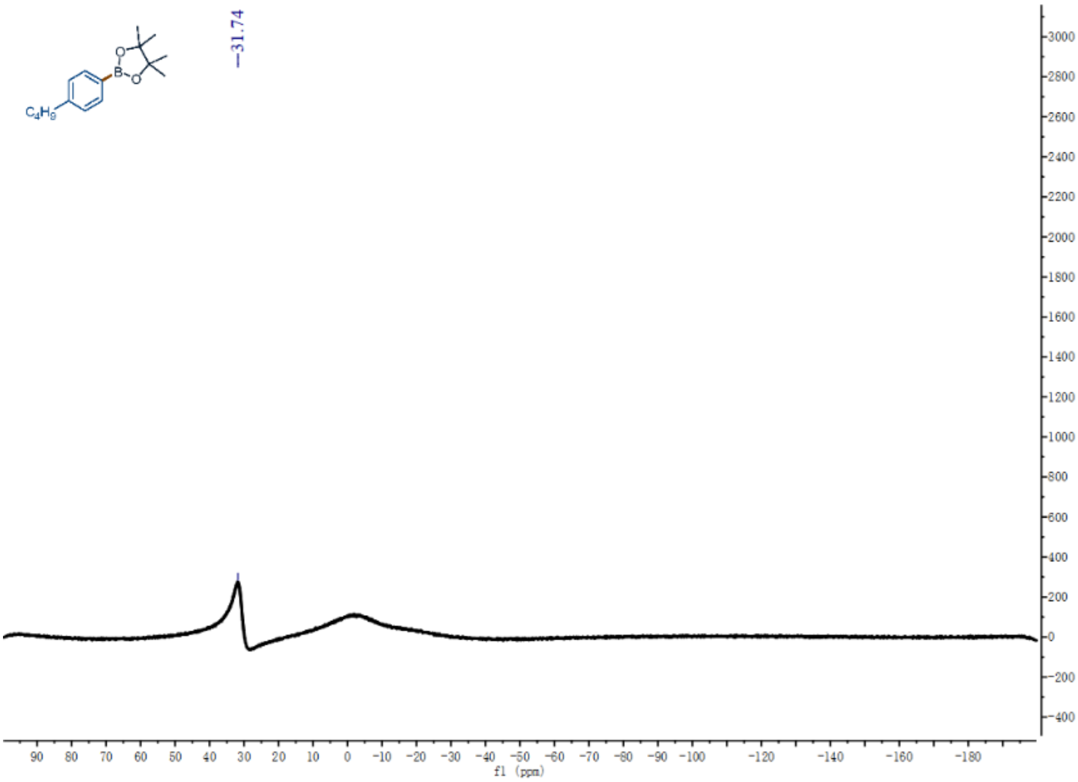

^{1}H NMR (400 MHz, $CDCl_3$) spectrum of compound **3f**

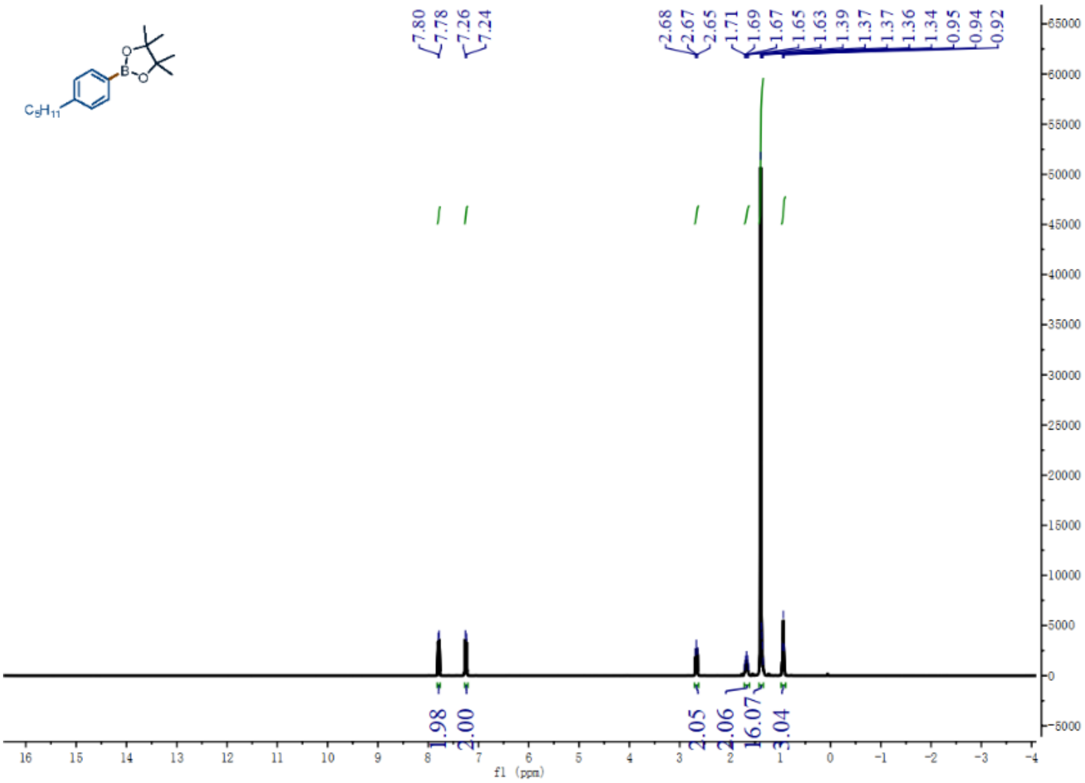

^{13}C NMR (100 MHz, $CDCl_3$) spectrum of compound **3f**

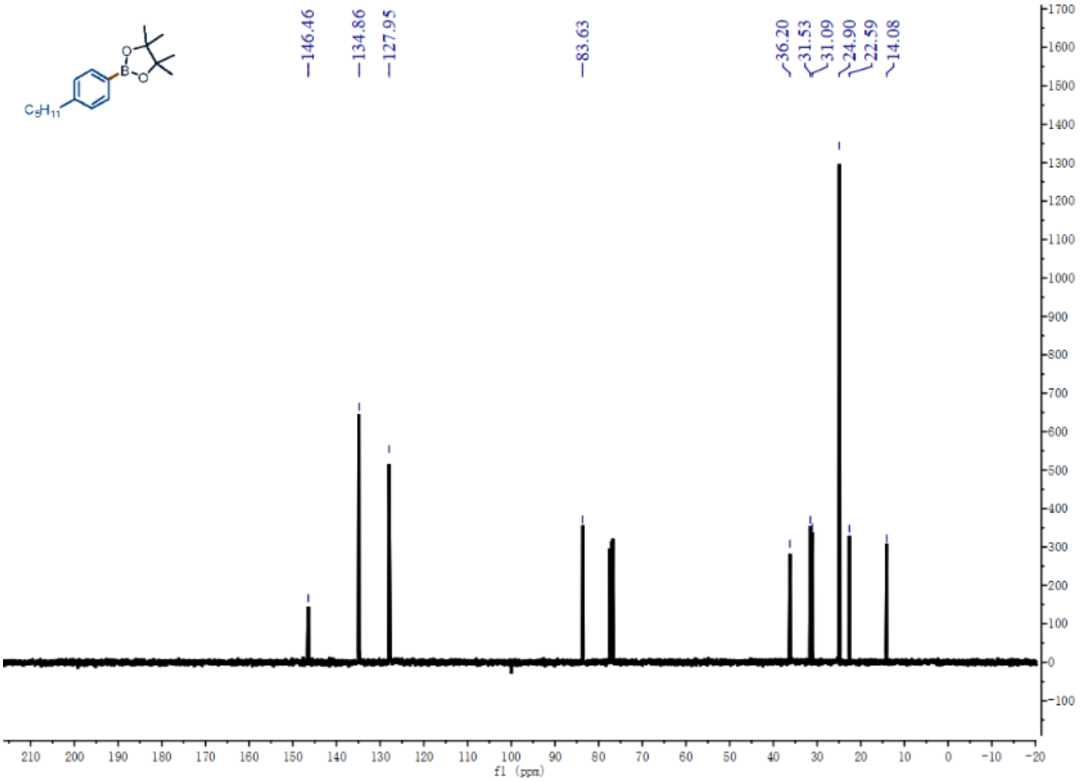

^{11}B NMR (376 MHz, $CDCl_3$) spectrum of compound **3f**

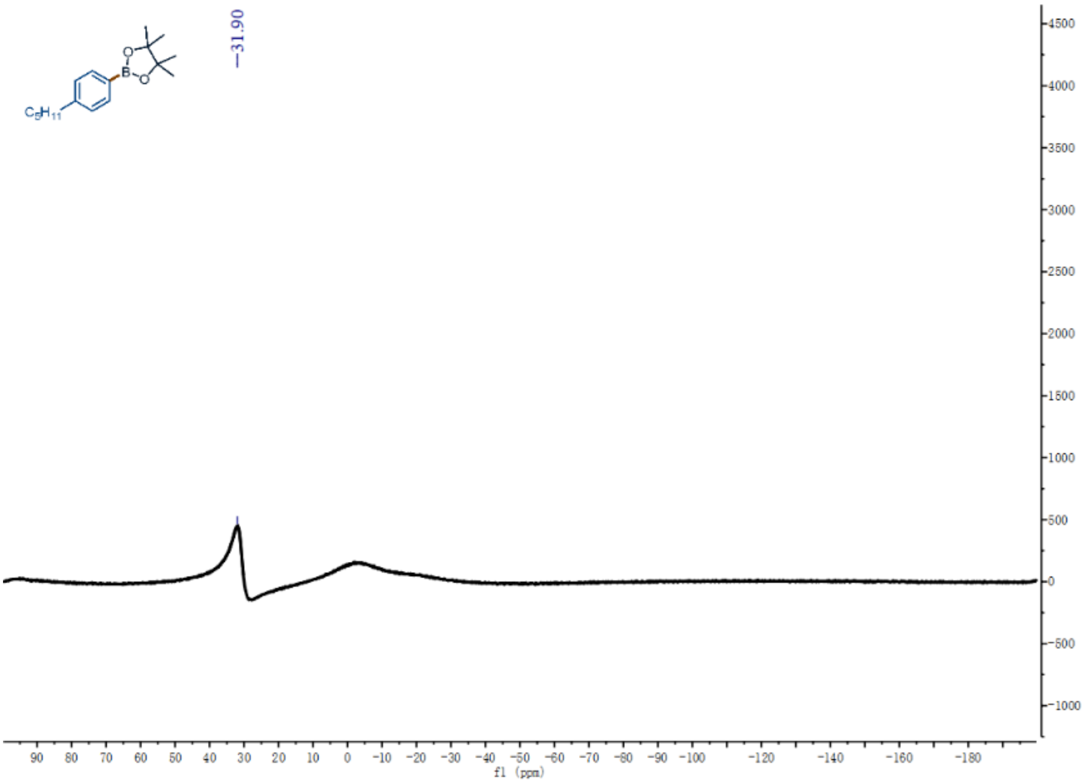

^{1}H NMR (400 MHz, $CDCl_3$) spectrum of compound **3g**

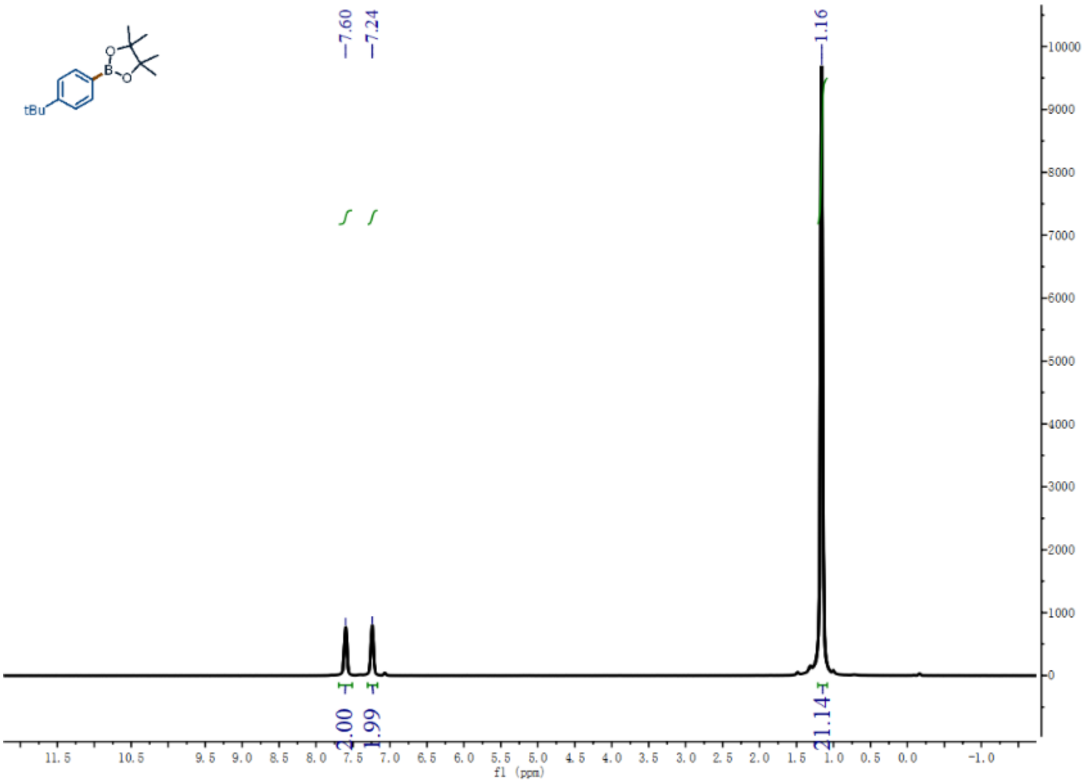

^{13}C NMR (100 MHz, $CDCl_3$) spectrum of compound **3g**

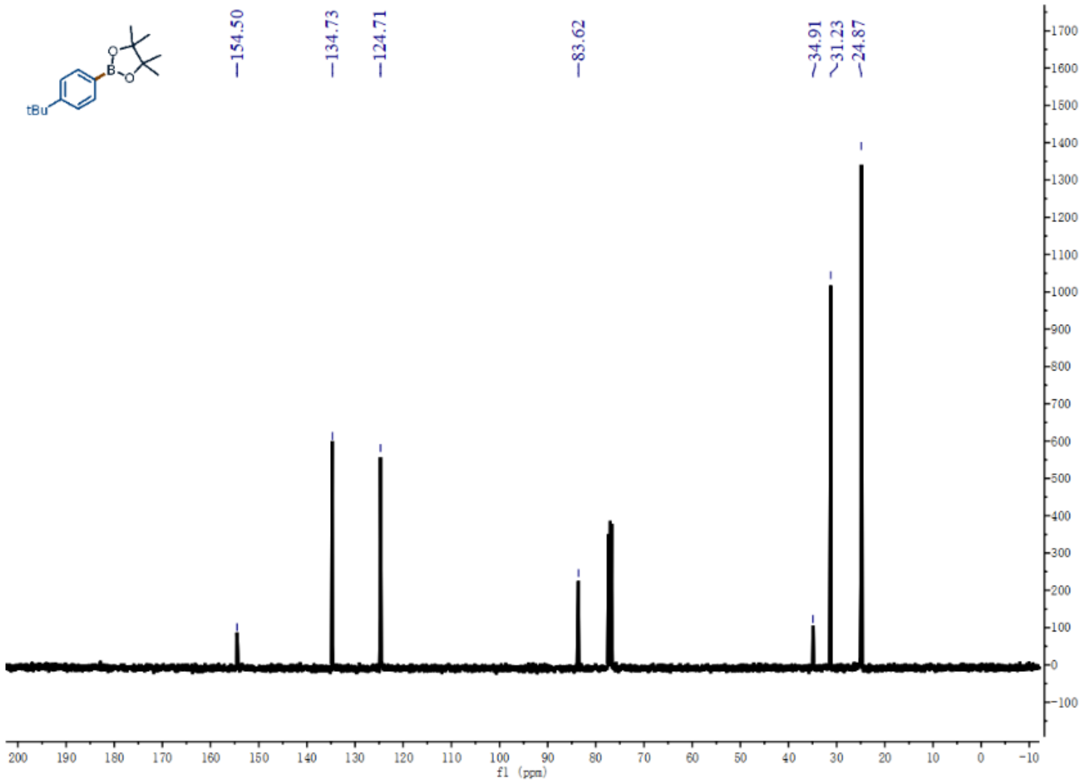

^{11}B NMR (376 MHz, $CDCl_3$) spectrum of compound **3g**

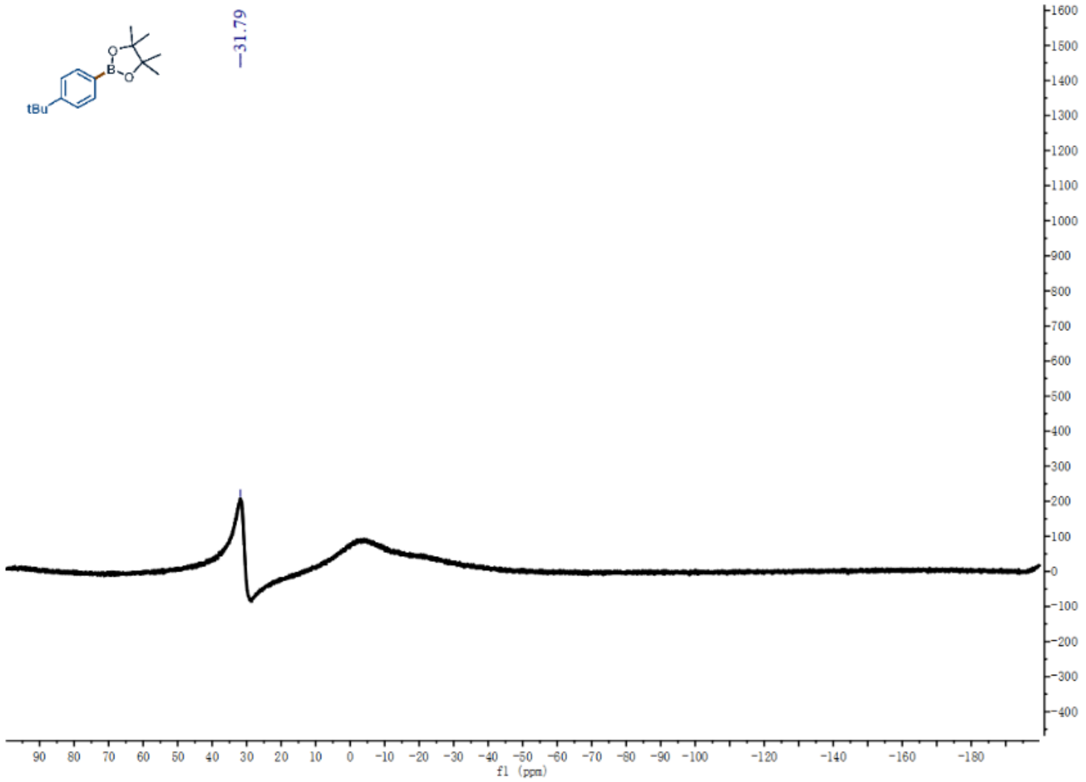

^{1}H NMR (400 MHz, $CDCl_3$) spectrum of compound **3h**

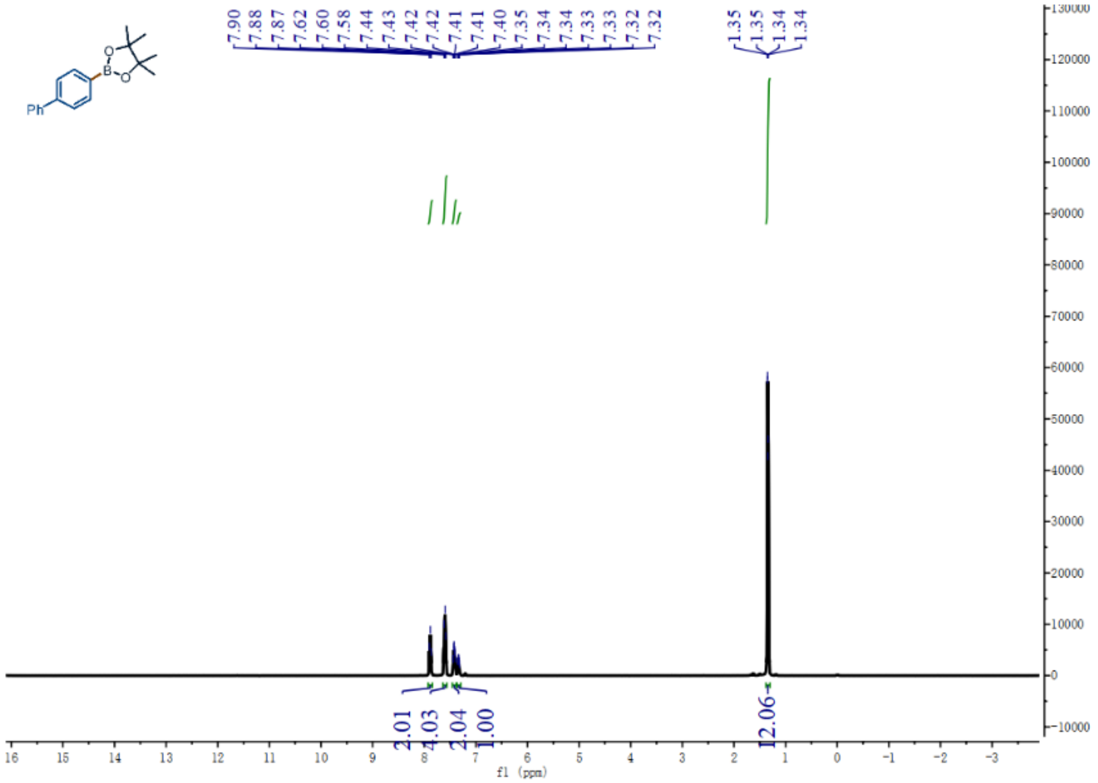

^{13}C NMR (100 MHz, $CDCl_3$) spectrum of compound **3h**

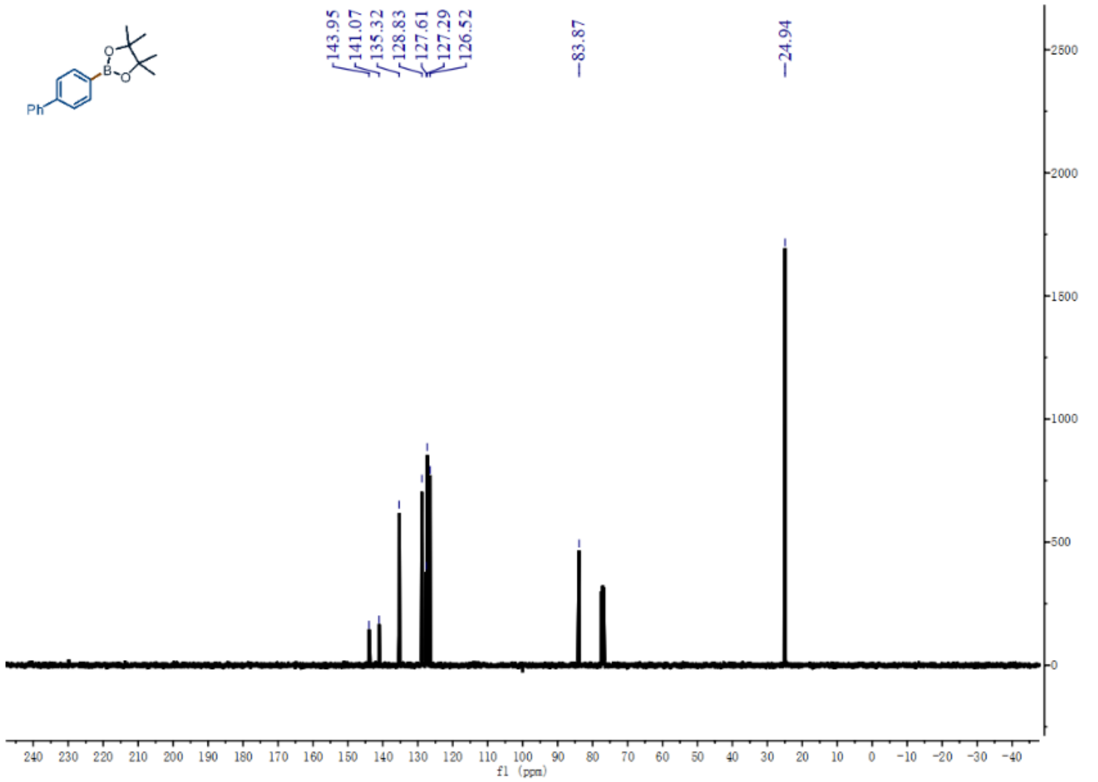

^{11}B NMR (376 MHz, $CDCl_3$) spectrum of compound **3h**

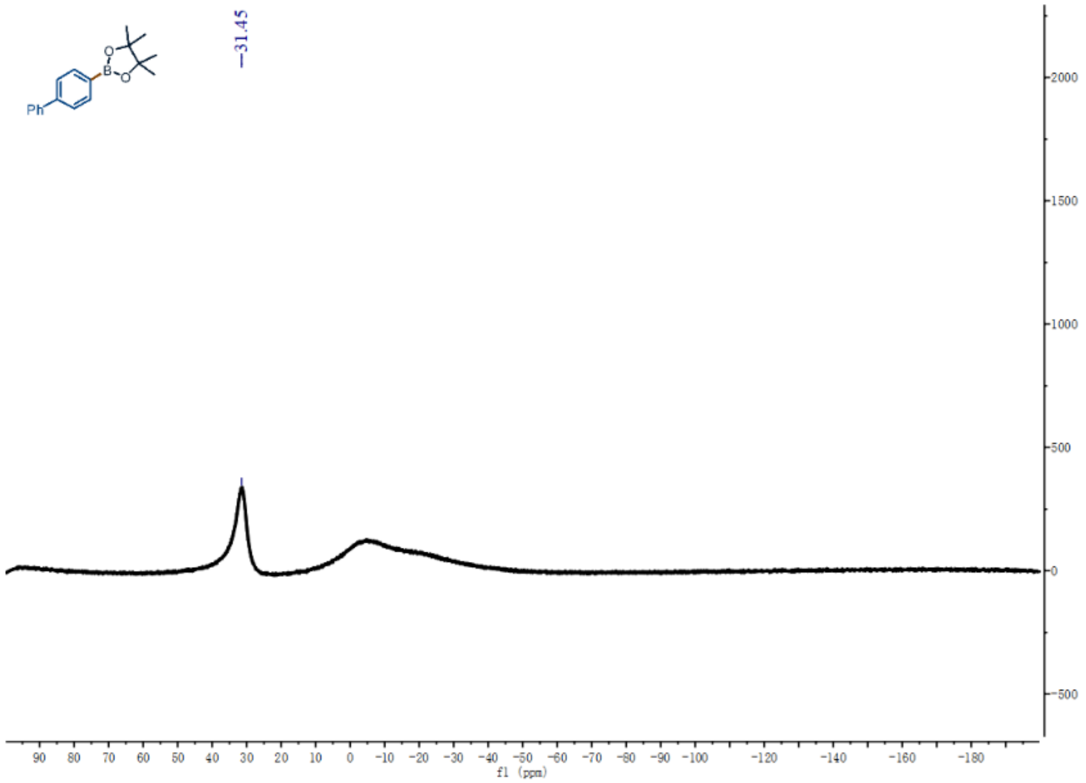

^{1}H NMR (400 MHz, $CDCl_3$) spectrum of compound **3i**

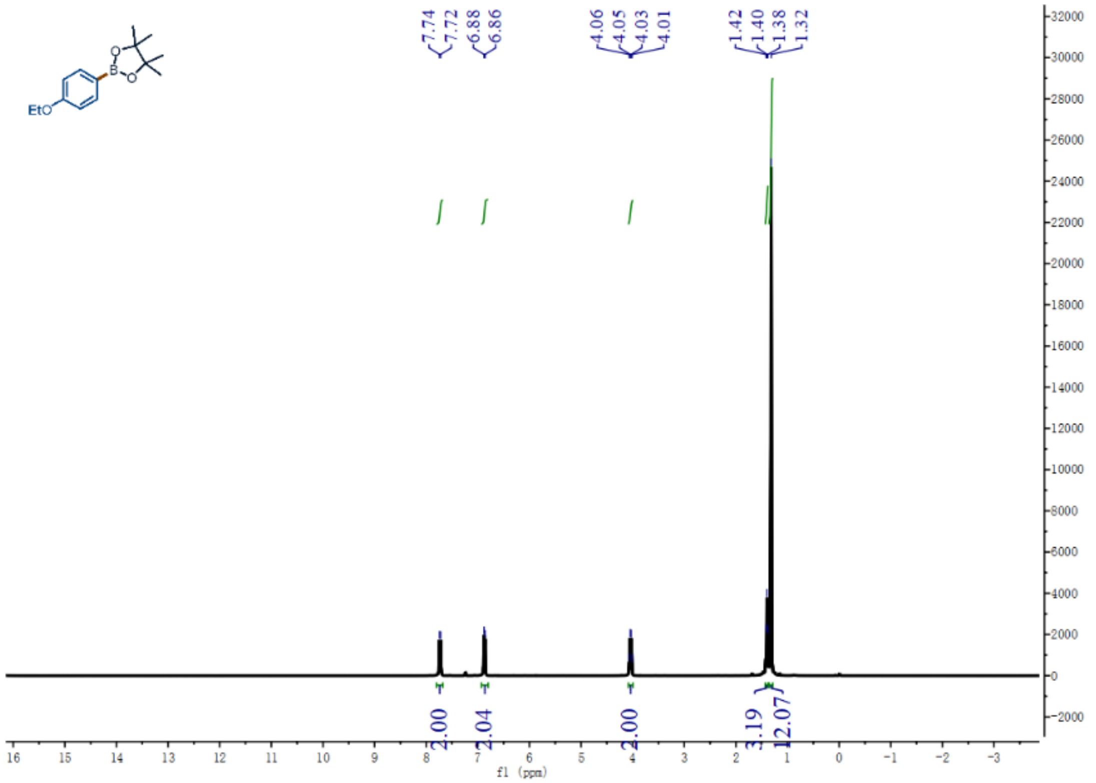

^{13}C NMR (100 MHz, $CDCl_3$) spectrum of compound **3i**

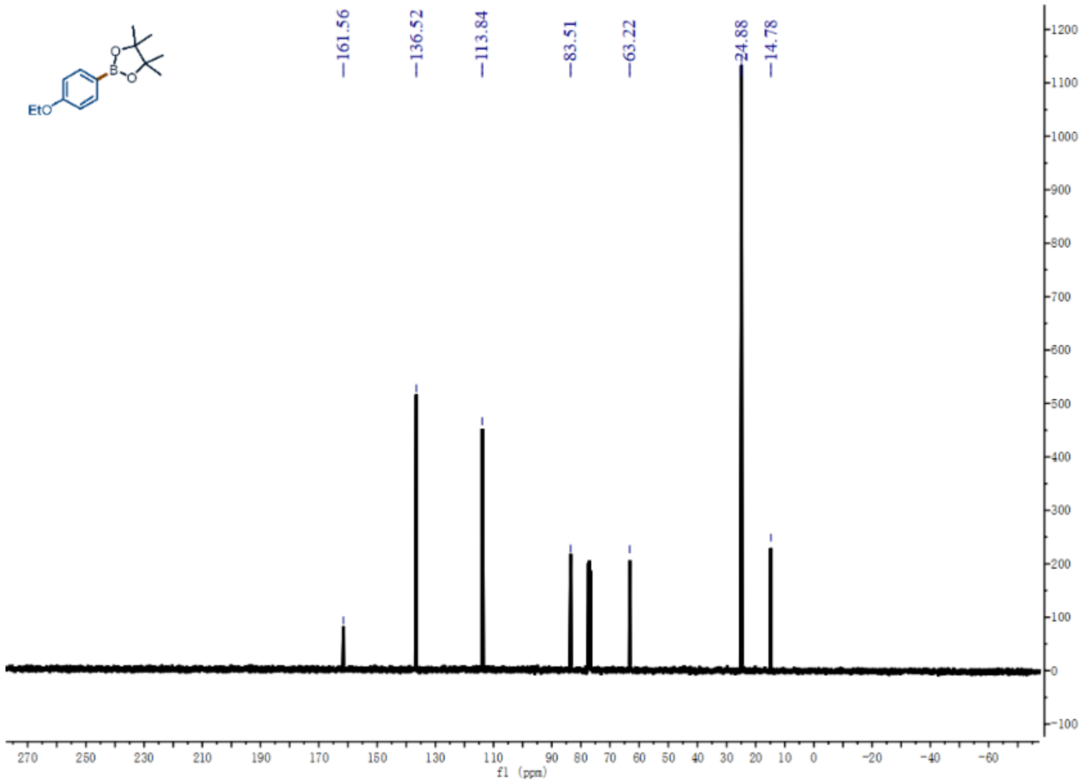

^{11}B NMR (376 MHz, $CDCl_3$) spectrum of compound **3i**

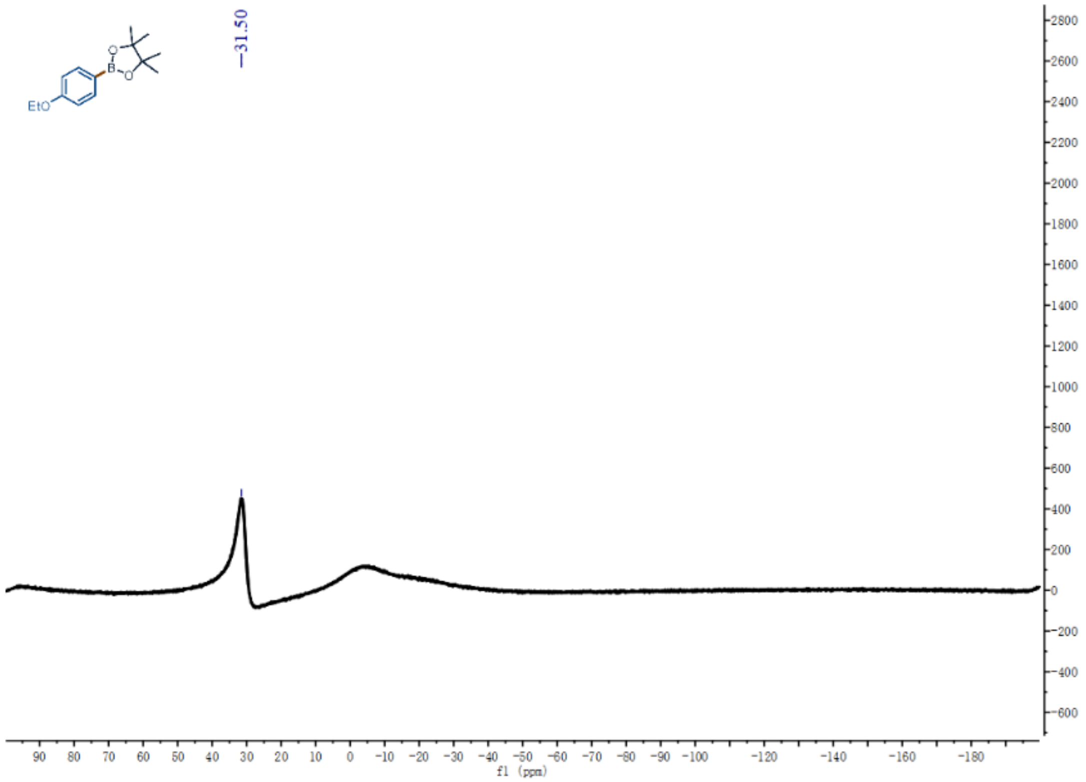

^{1}H NMR (400 MHz, $CDCl_3$) spectrum of compound **3j**

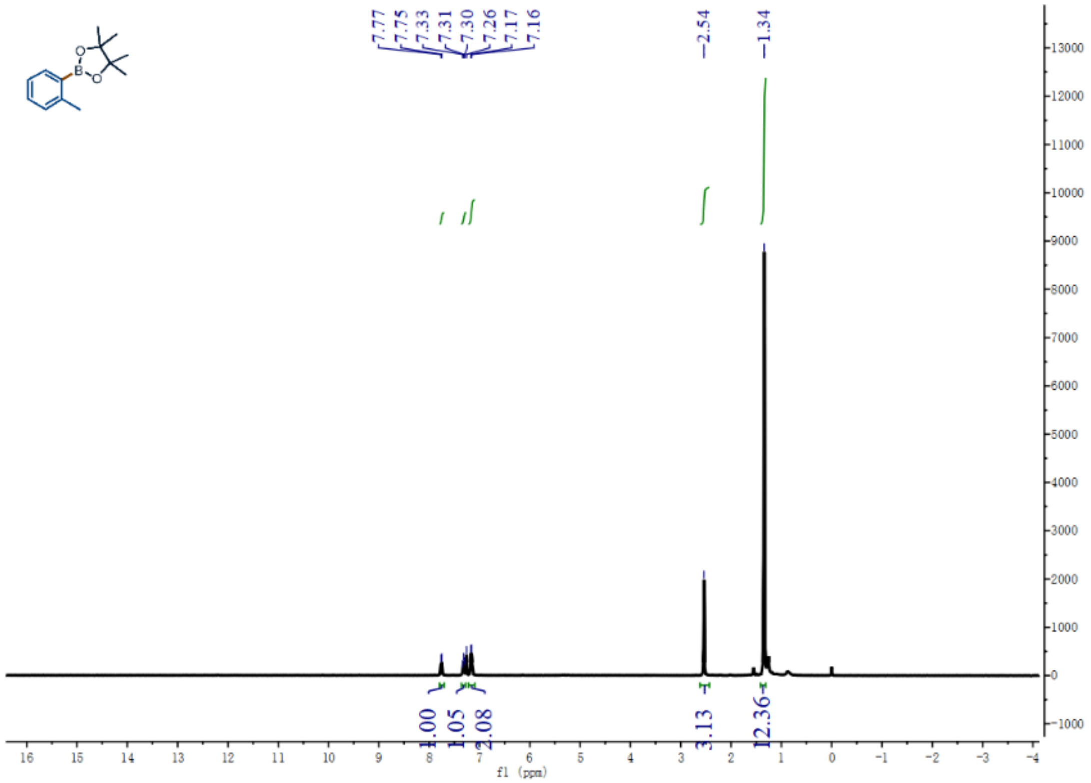

^{13}C NMR (100 MHz, $CDCl_3$) spectrum of compound **3j**

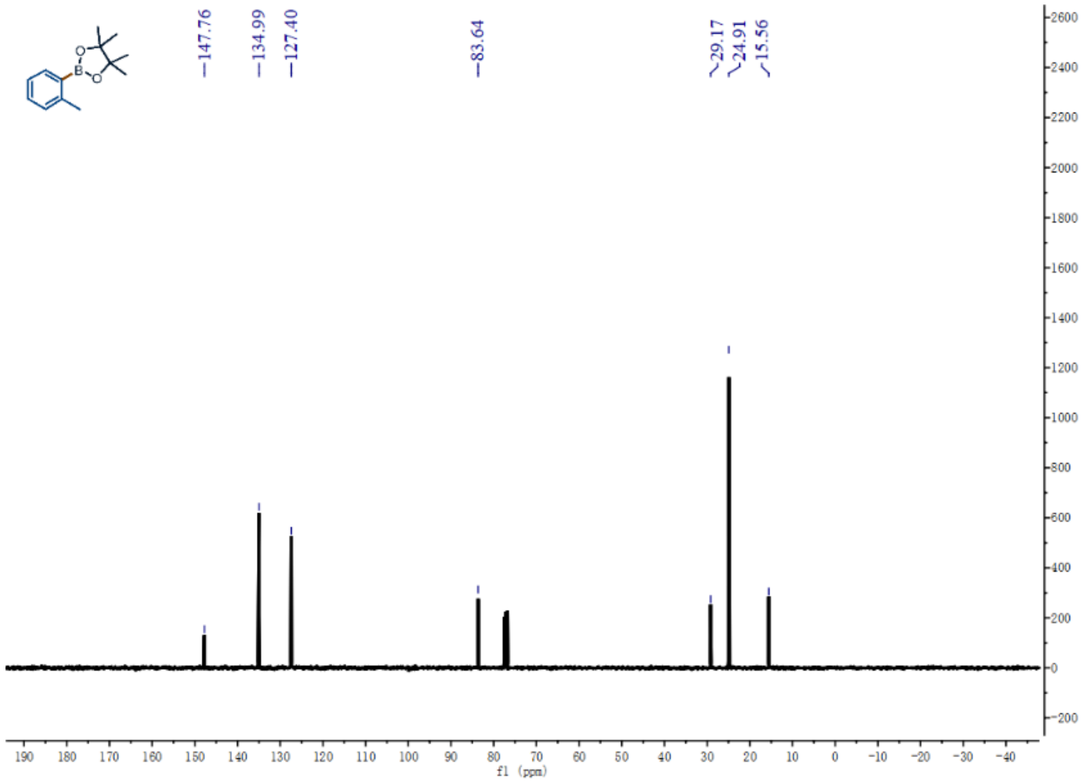

^{11}B NMR (376 MHz, $CDCl_3$) spectrum of compound **3j**

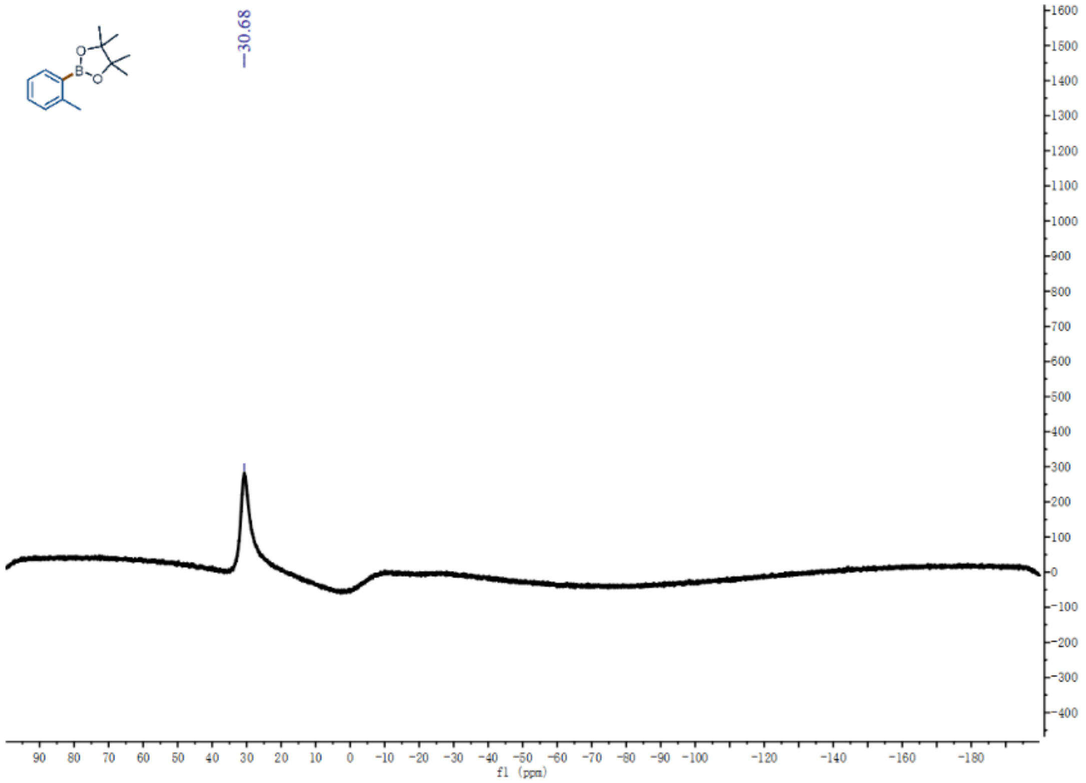

^{1}H NMR (400 MHz, $CDCl_3$) spectrum of compound **3k**

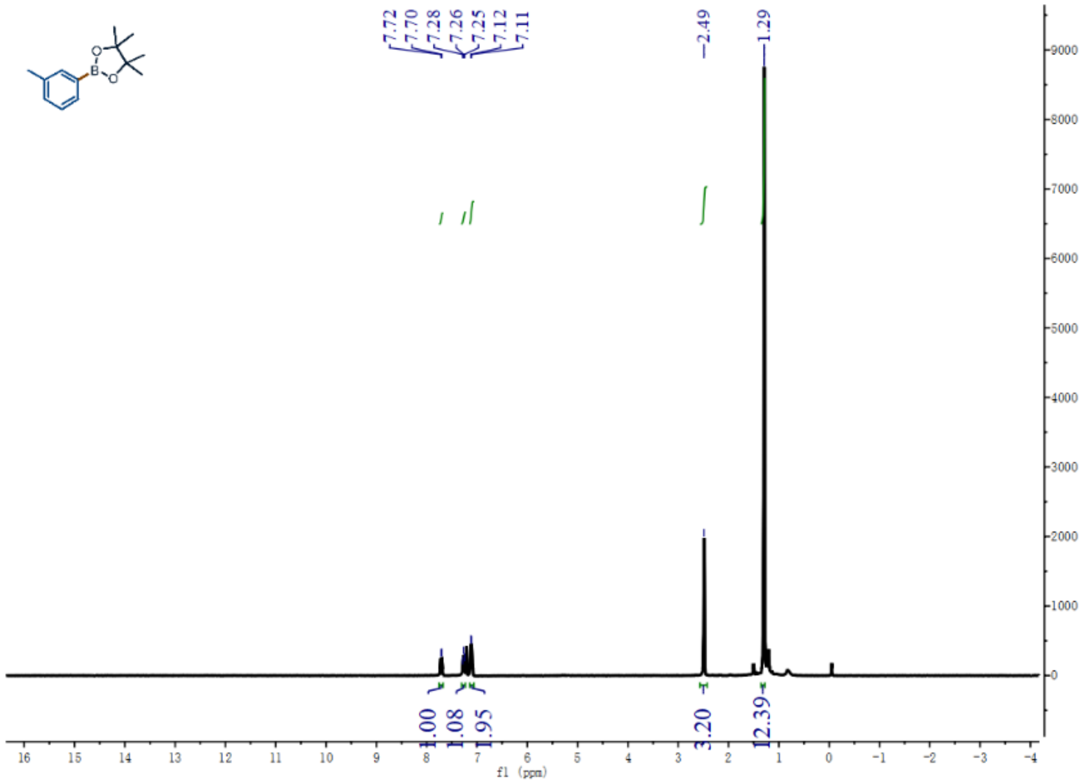

^{13}C NMR (100 MHz, $CDCl_3$) spectrum of compound **3k**

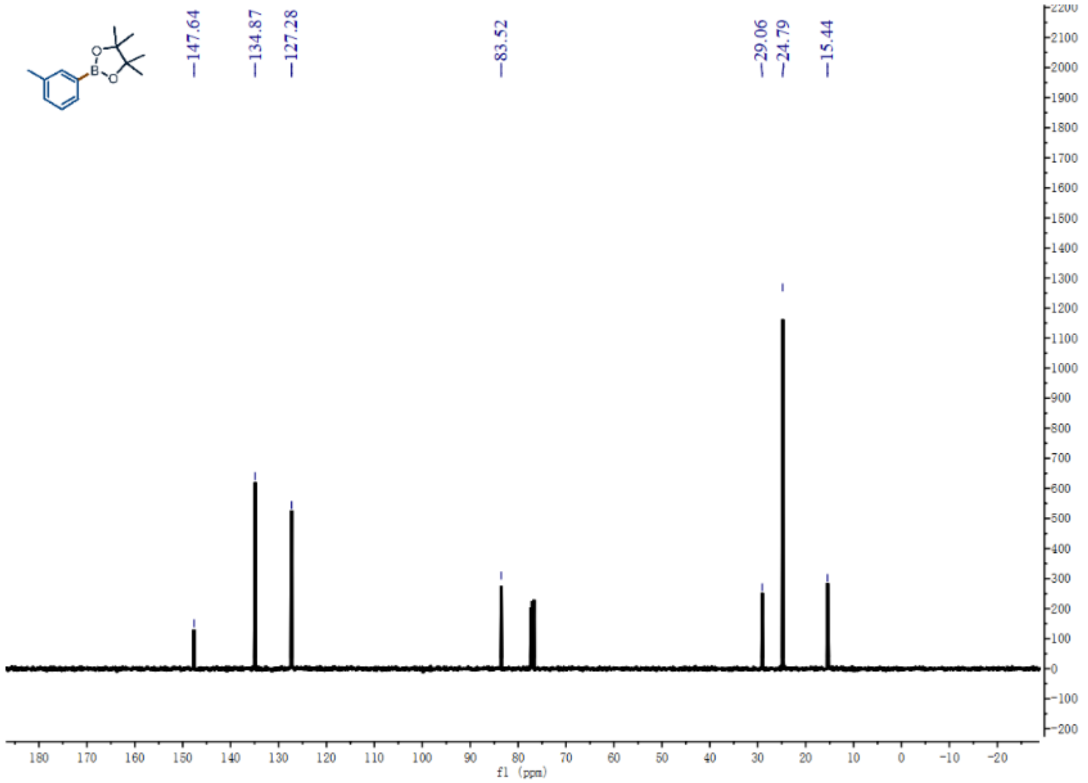

^{11}B NMR (376 MHz, $CDCl_3$) spectrum of compound **3k**

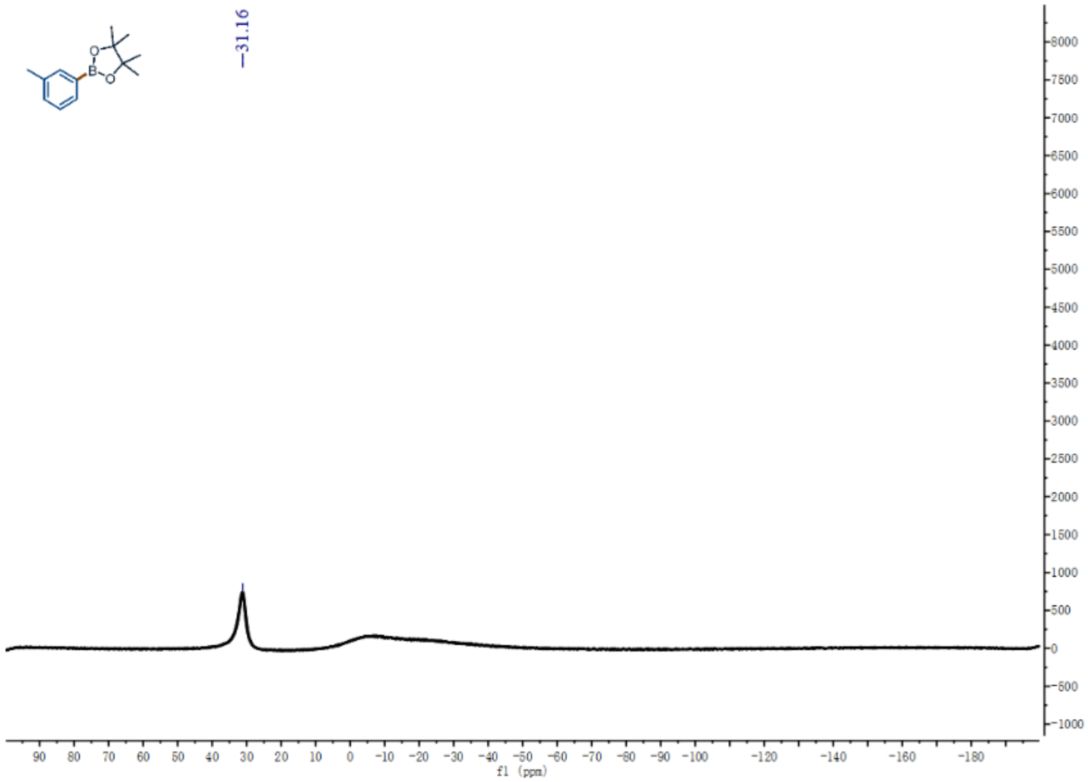

^{1}H NMR (400 MHz, $CDCl_3$) spectrum of compound **3l**

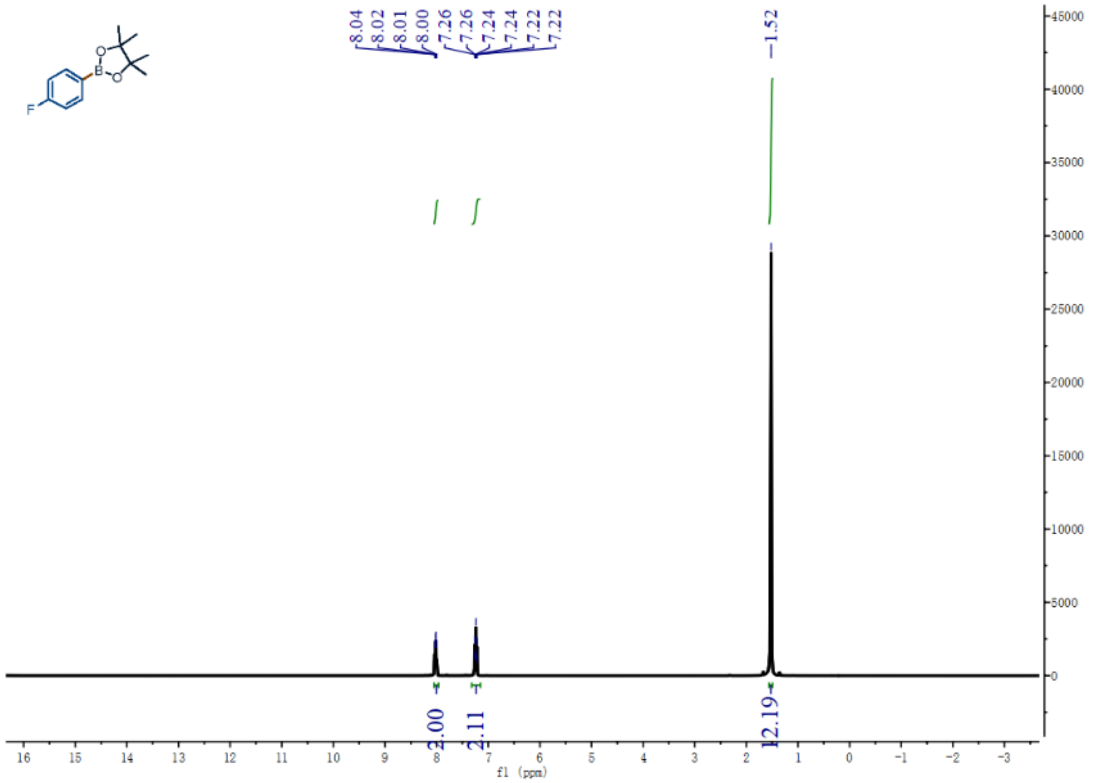

^{13}C NMR (100 MHz, $CDCl_3$) spectrum of compound **3l**

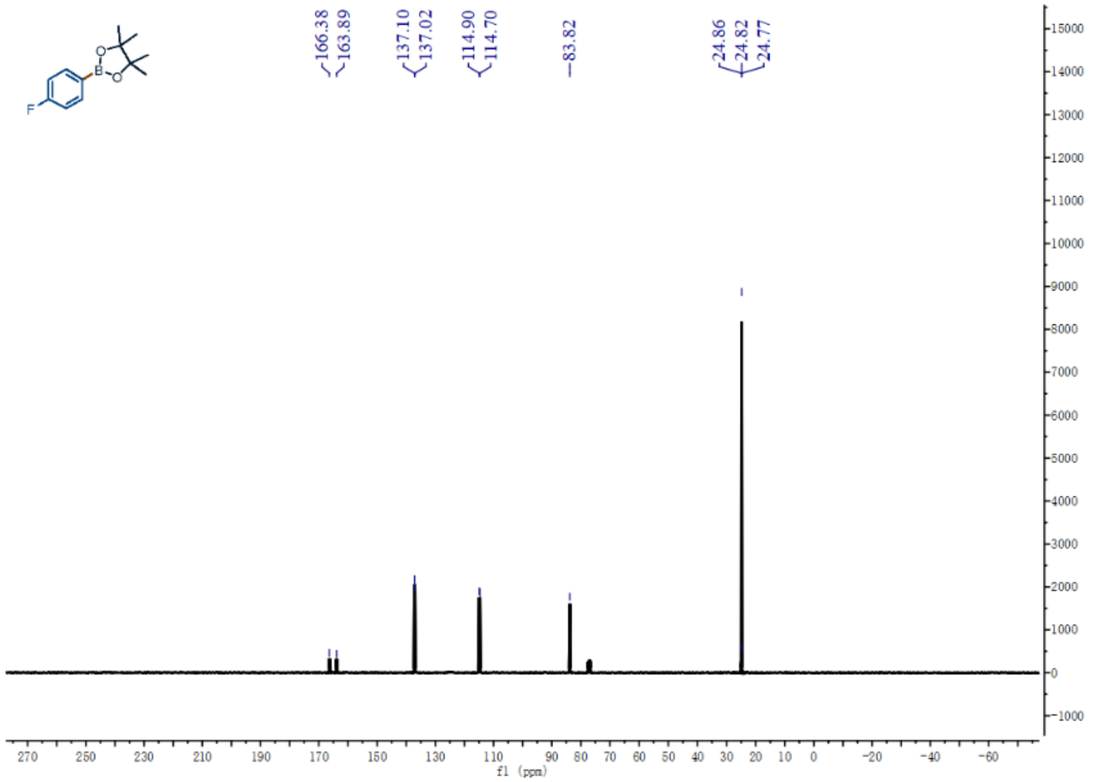

^{11}B NMR (376 MHz, $CDCl_3$) spectrum of compound **3l**

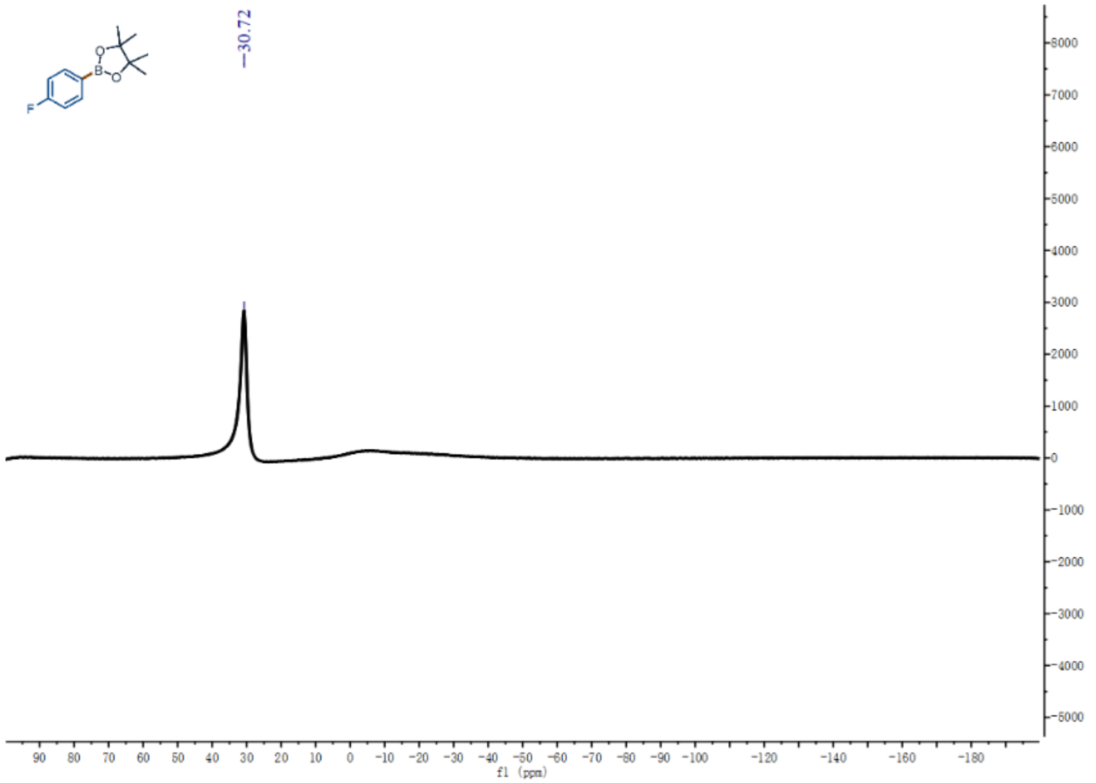

^{1}H NMR (400 MHz, $CDCl_3$) spectrum of compound **3m**

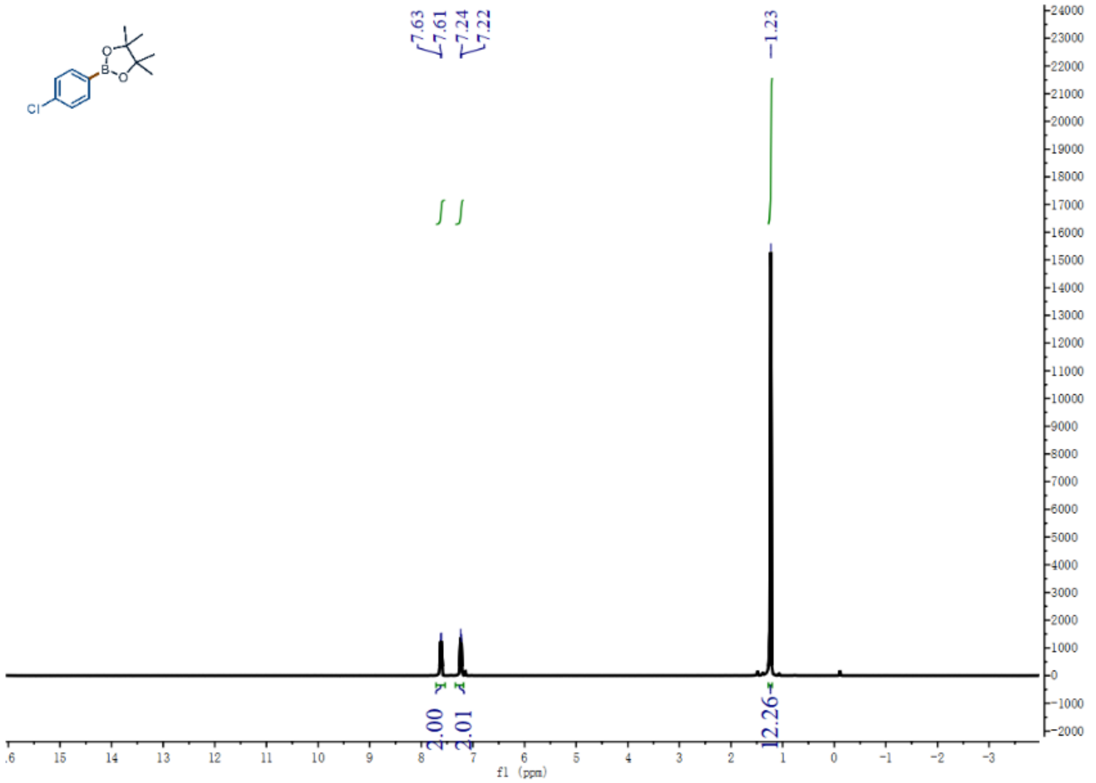

^{13}C NMR (100 MHz, $CDCl_3$) spectrum of compound **3m**

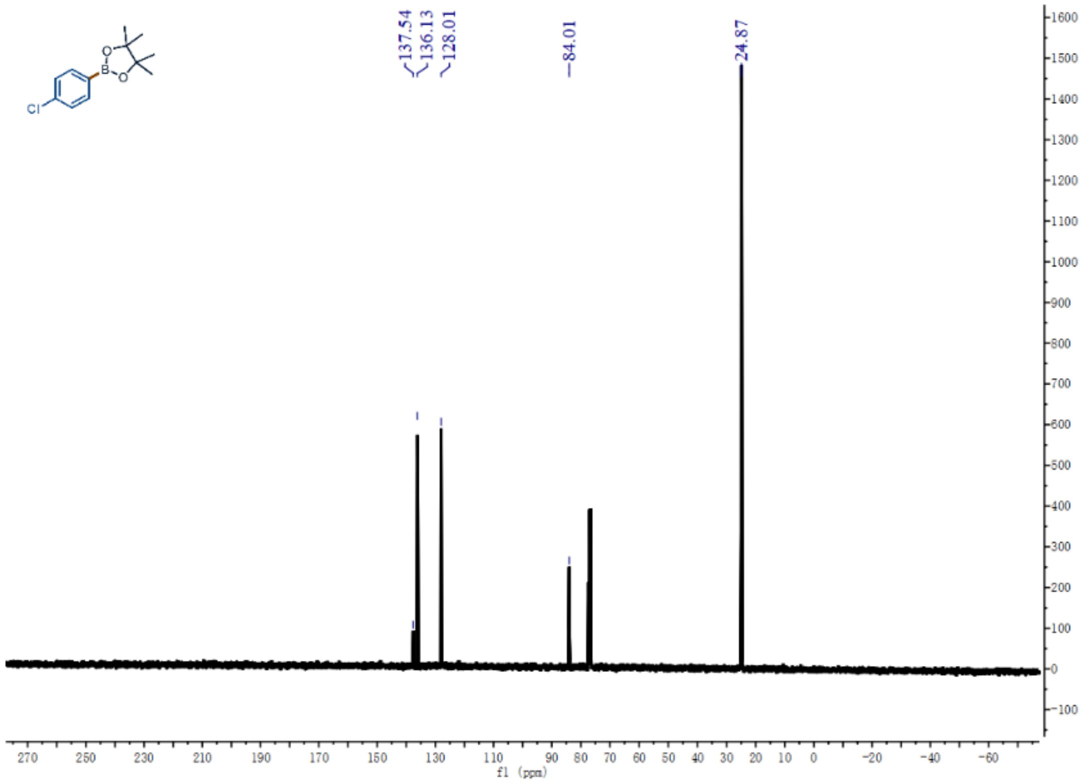

^{11}B NMR (376 MHz, $CDCl_3$) spectrum of compound **3m**

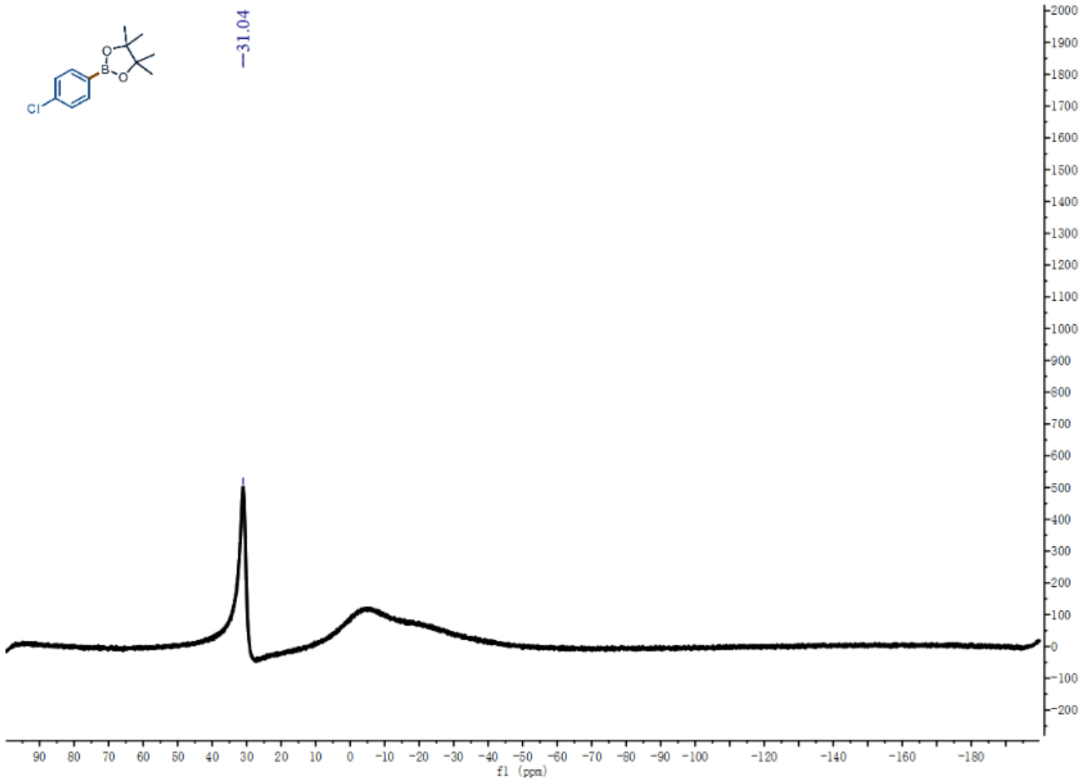

^{1}H NMR (400 MHz, $CDCl_3$) spectrum of compound **3n**

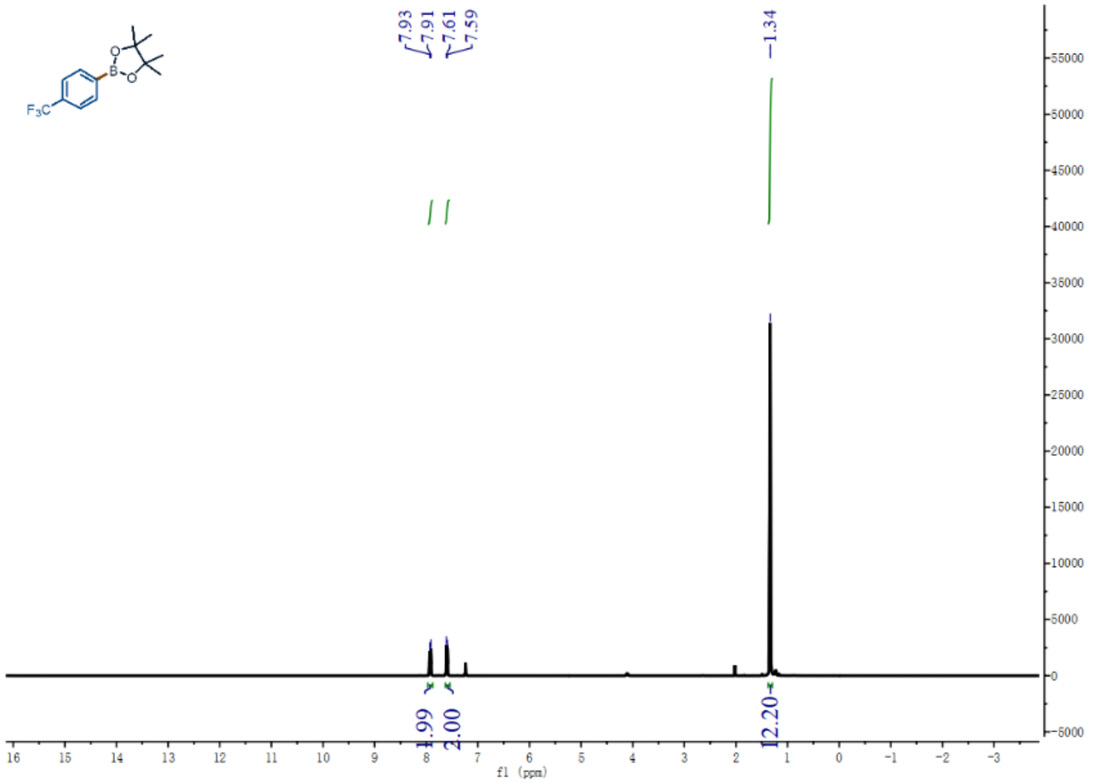

^{13}C NMR (100 MHz, $CDCl_3$) spectrum of compound **3n**

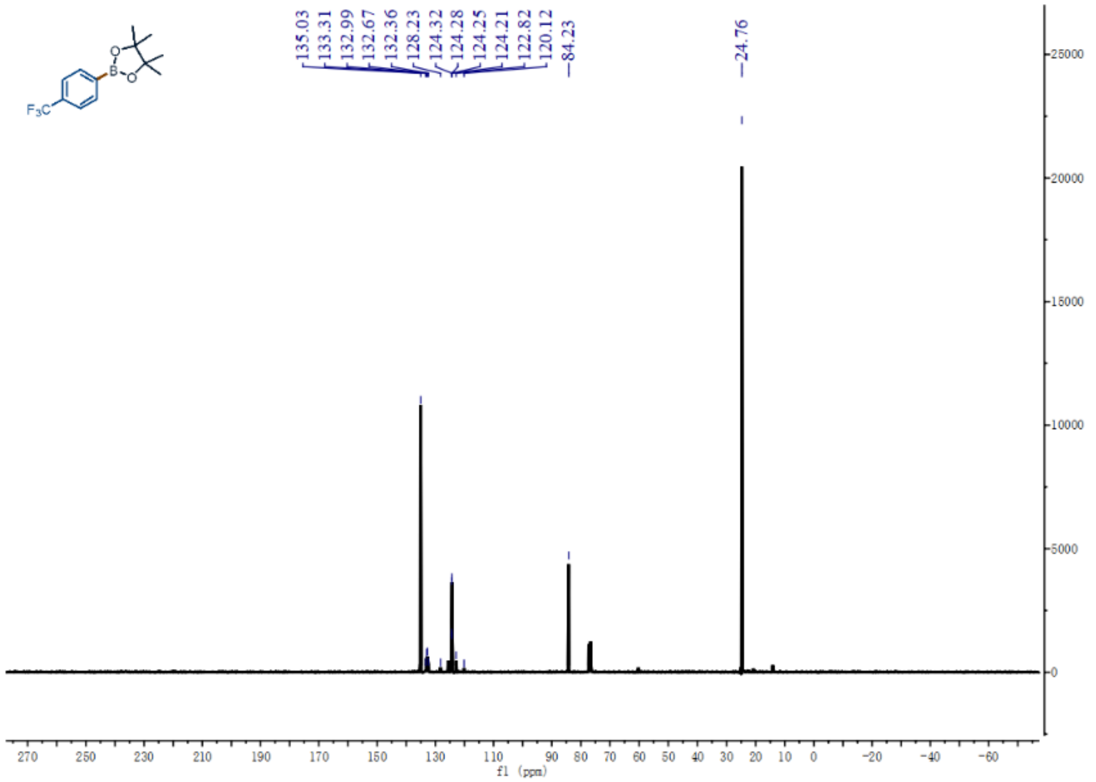

^{11}B NMR (376 MHz, $CDCl_3$) spectrum of compound **3n**

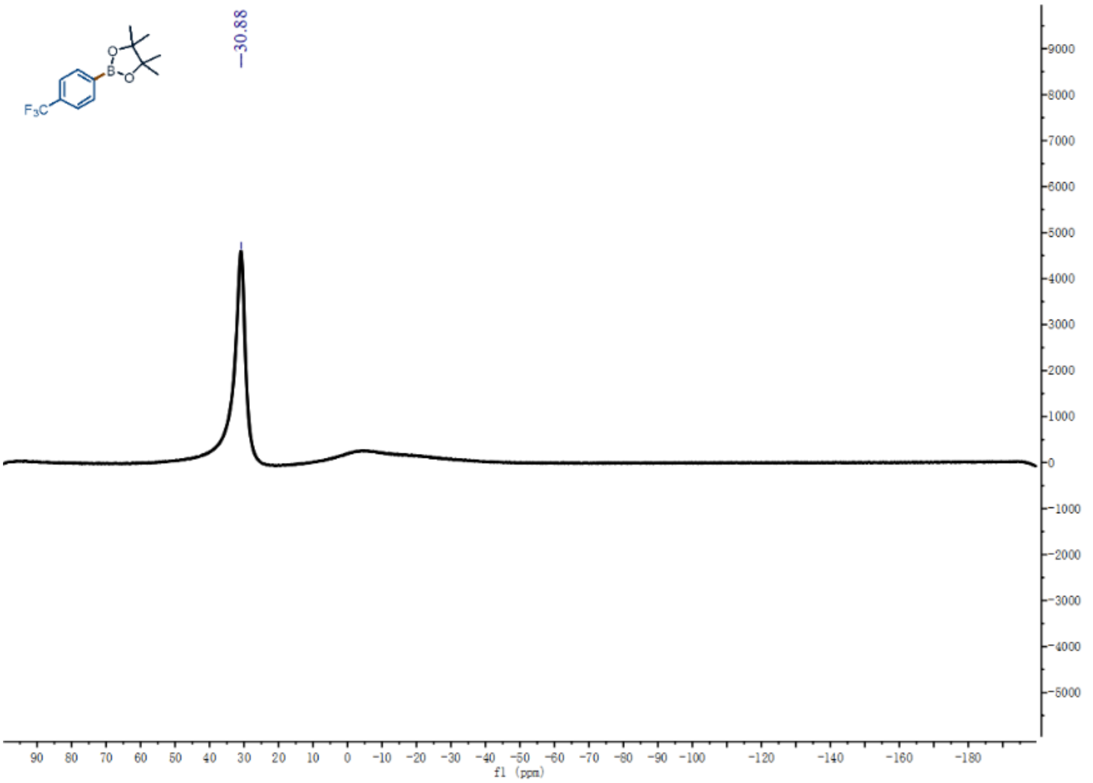

^{1}H NMR (400 MHz, $CDCl_3$) spectrum of compound **3o**

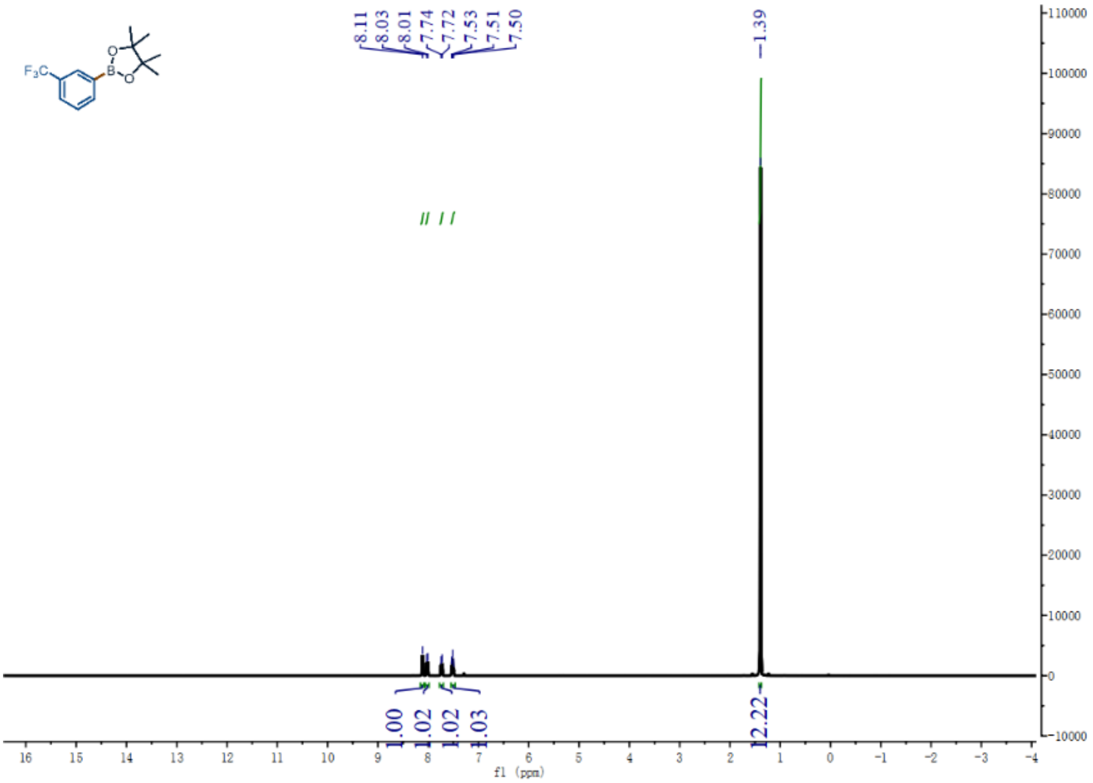

^{13}C NMR (100 MHz, $CDCl_3$) spectrum of compound **3o**

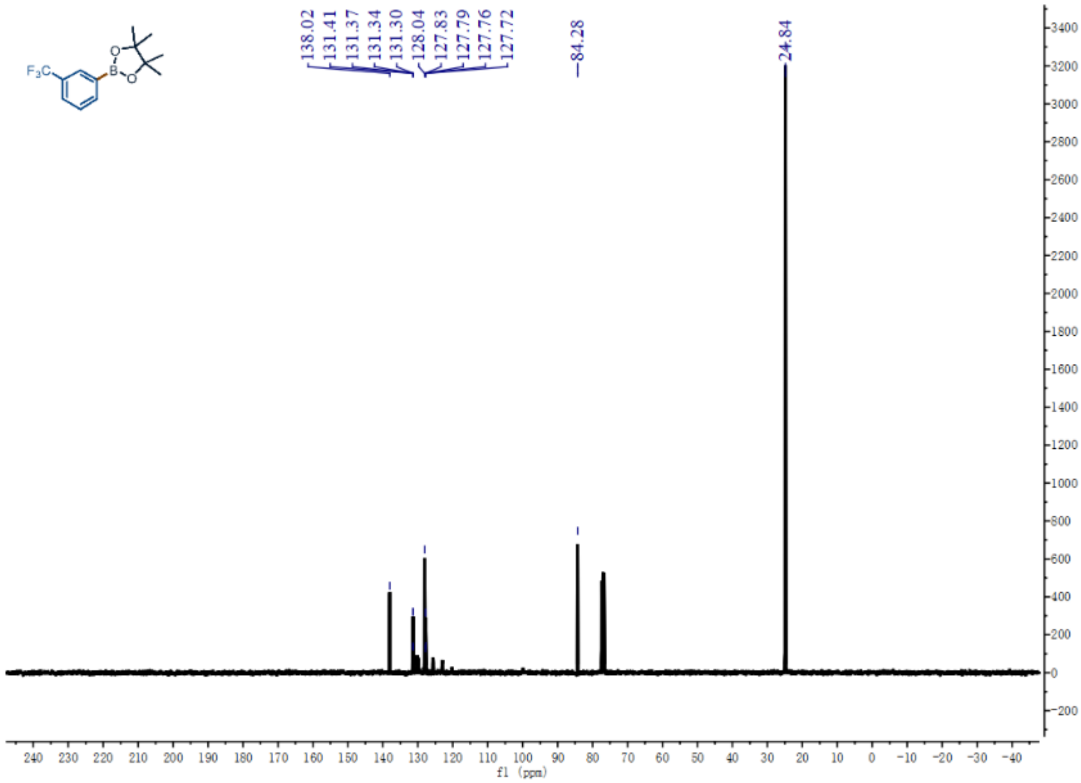

^{11}B NMR (376 MHz, $CDCl_3$) spectrum of compound **3o**

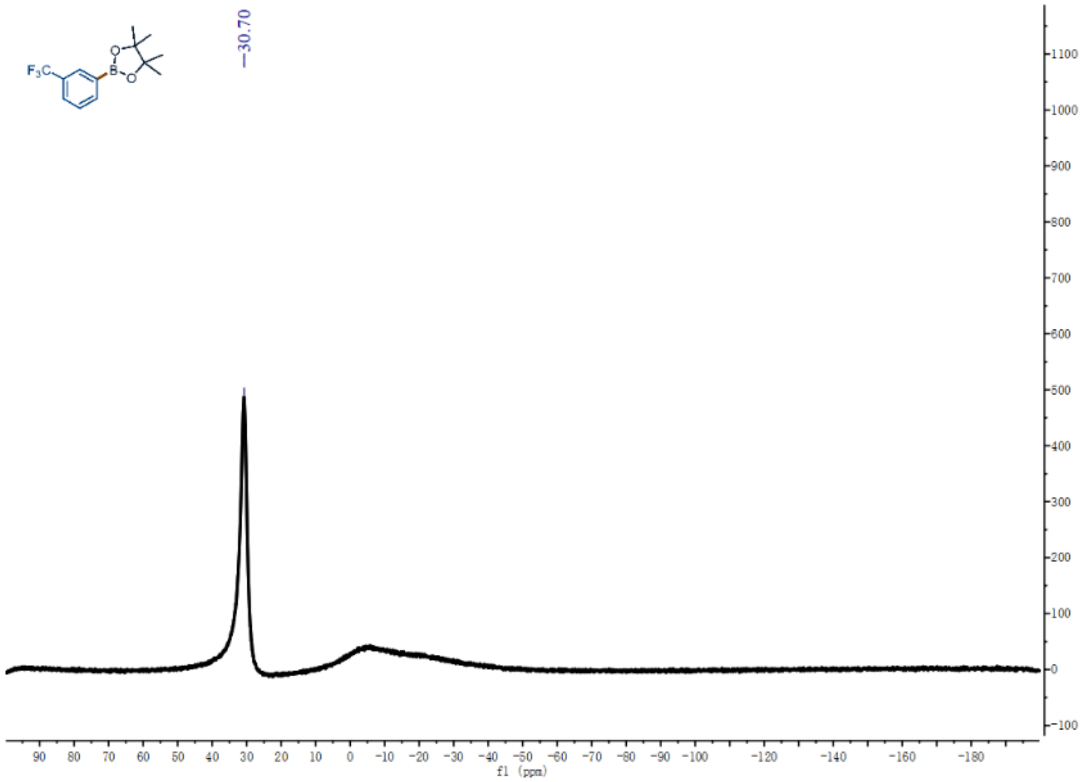

^{1}H NMR (400 MHz, $CDCl_3$) spectrum of compound **3p**

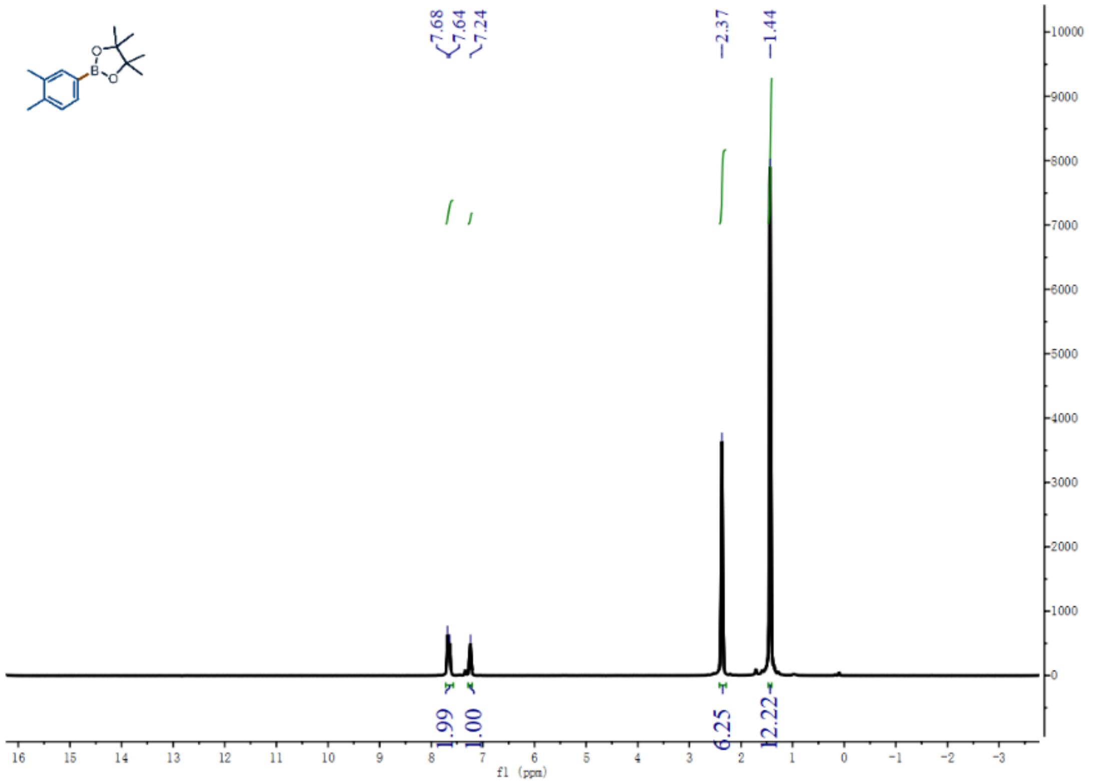

^{13}C NMR (100 MHz, $CDCl_3$) spectrum of compound **3p**

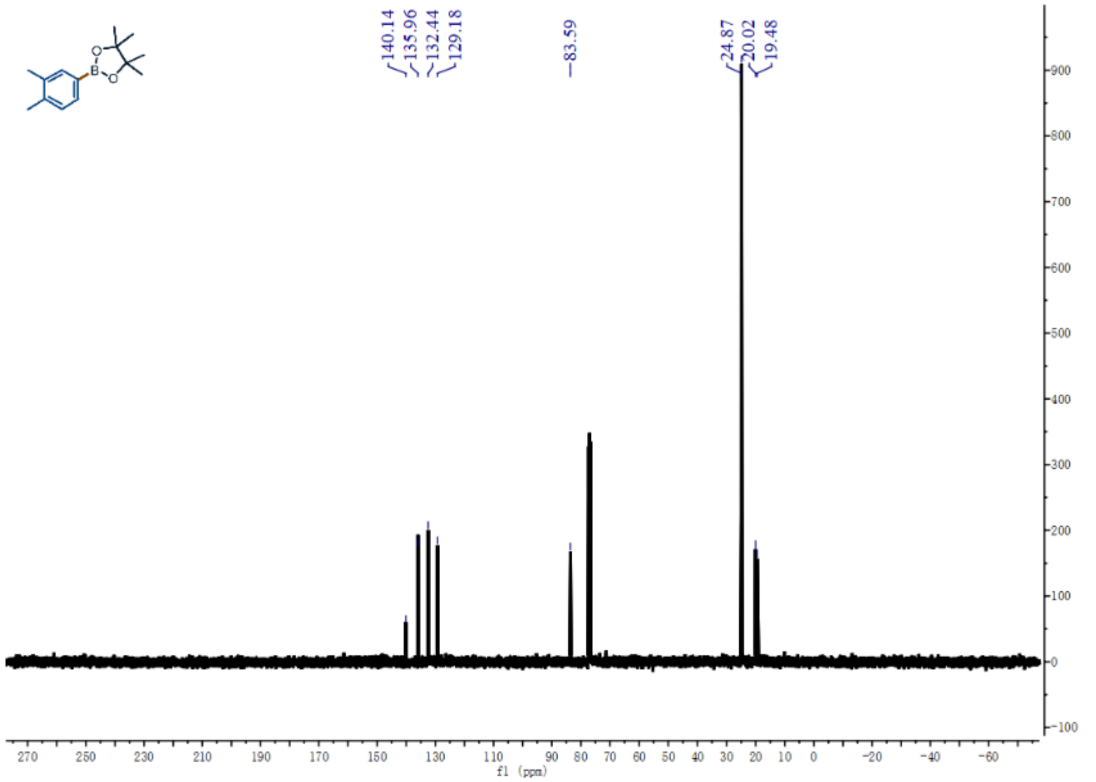

^{11}B NMR (376 MHz, $CDCl_3$) spectrum of compound **3p**

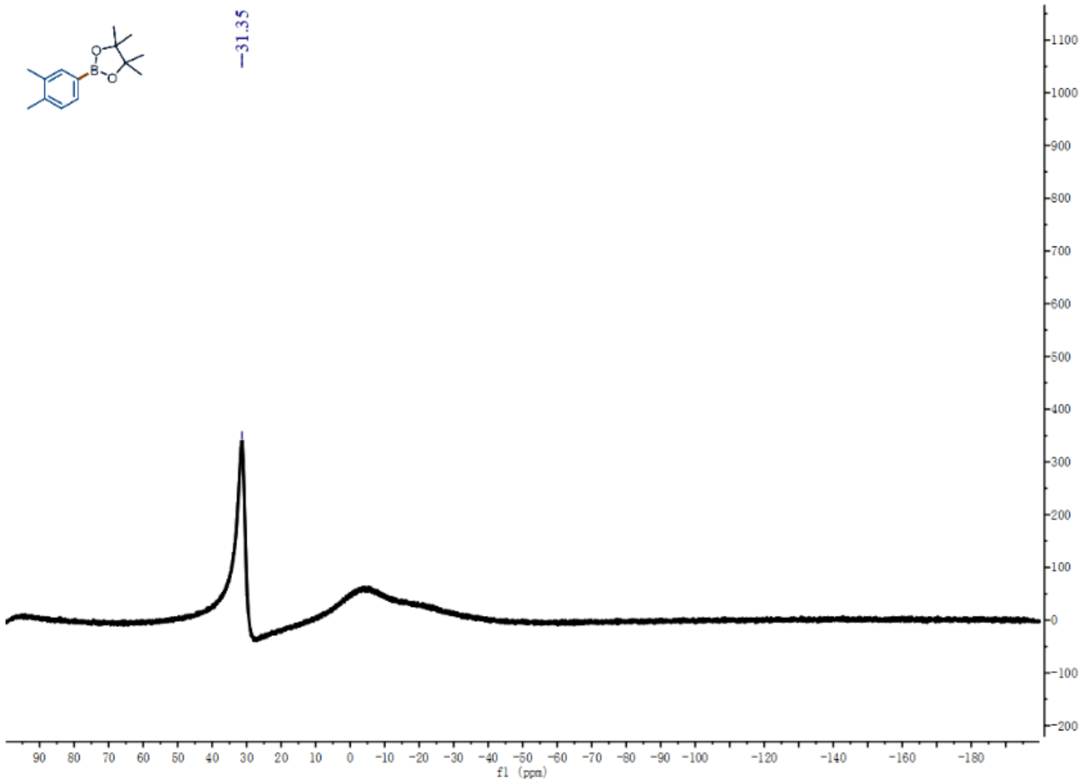

^{1}H NMR (400 MHz, $CDCl_3$) spectrum of compound **3q**

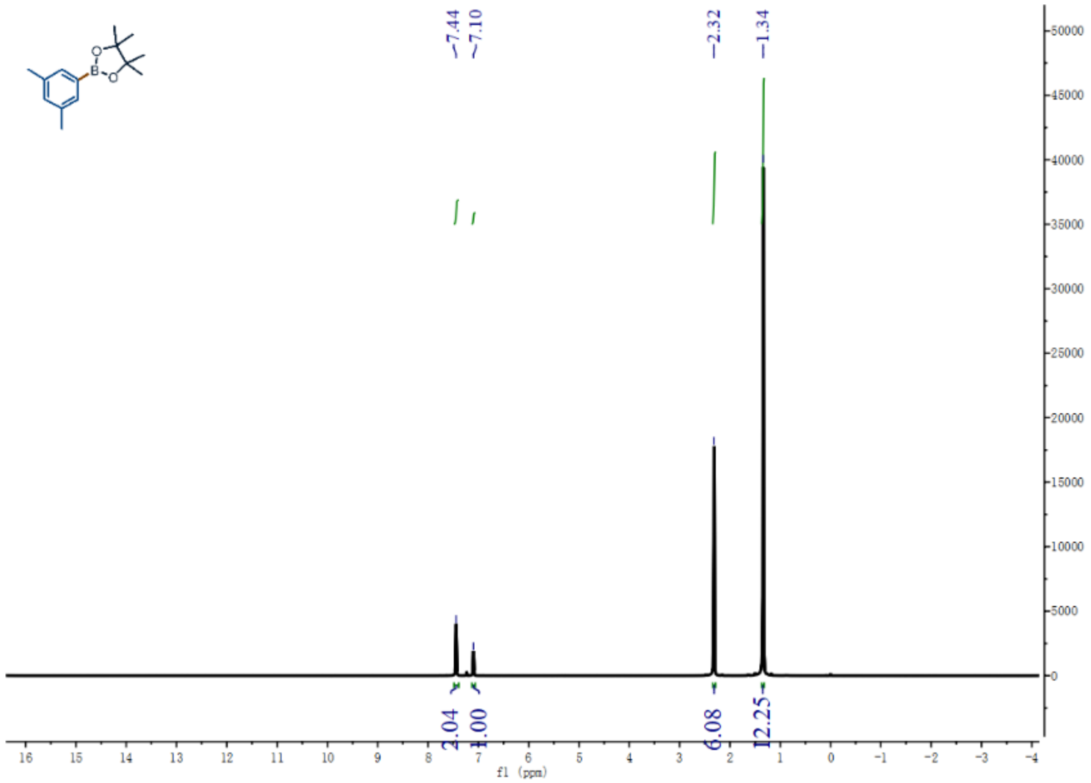

^{13}C NMR (100 MHz, $CDCl_3$) spectrum of compound **3q**

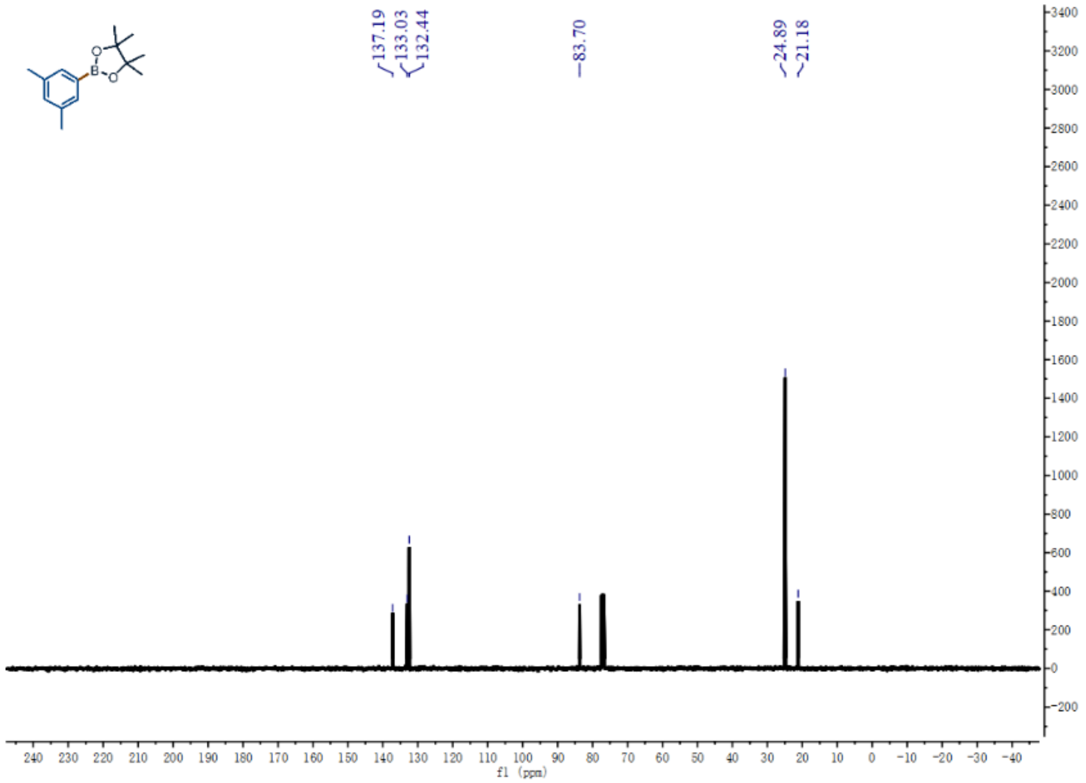

^{11}B NMR (376 MHz, $CDCl_3$) spectrum of compound **3q**

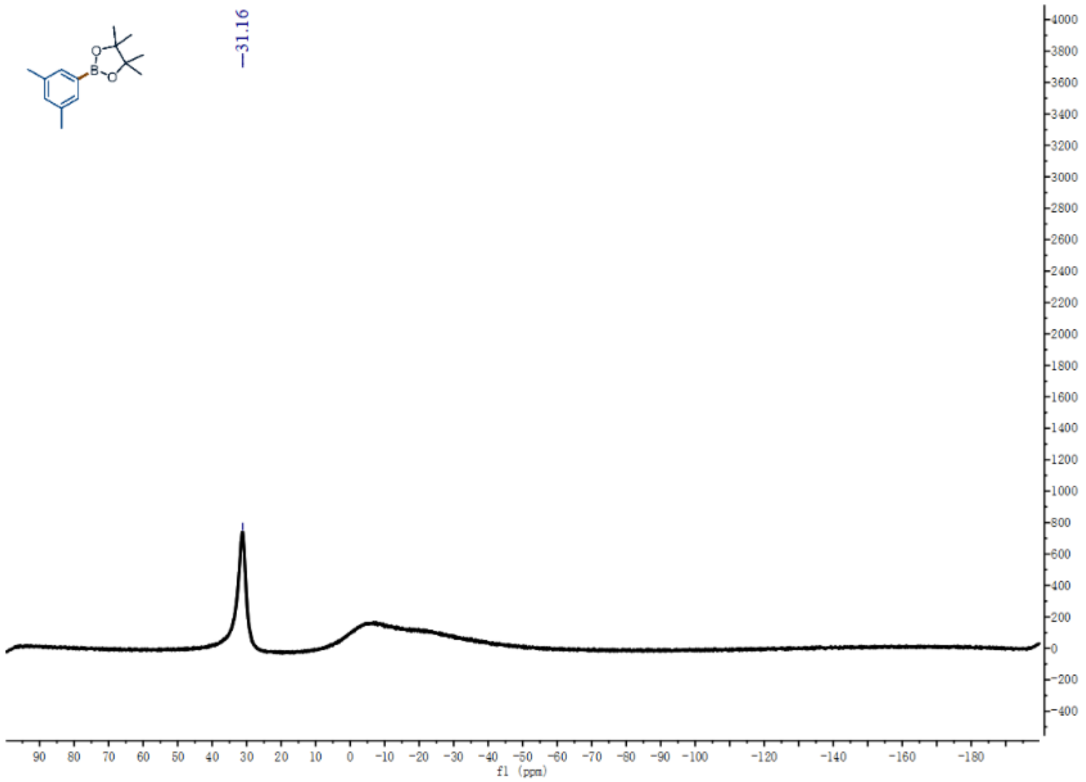

^{1}H NMR (400 MHz, $CDCl_3$) spectrum of compound **3r**

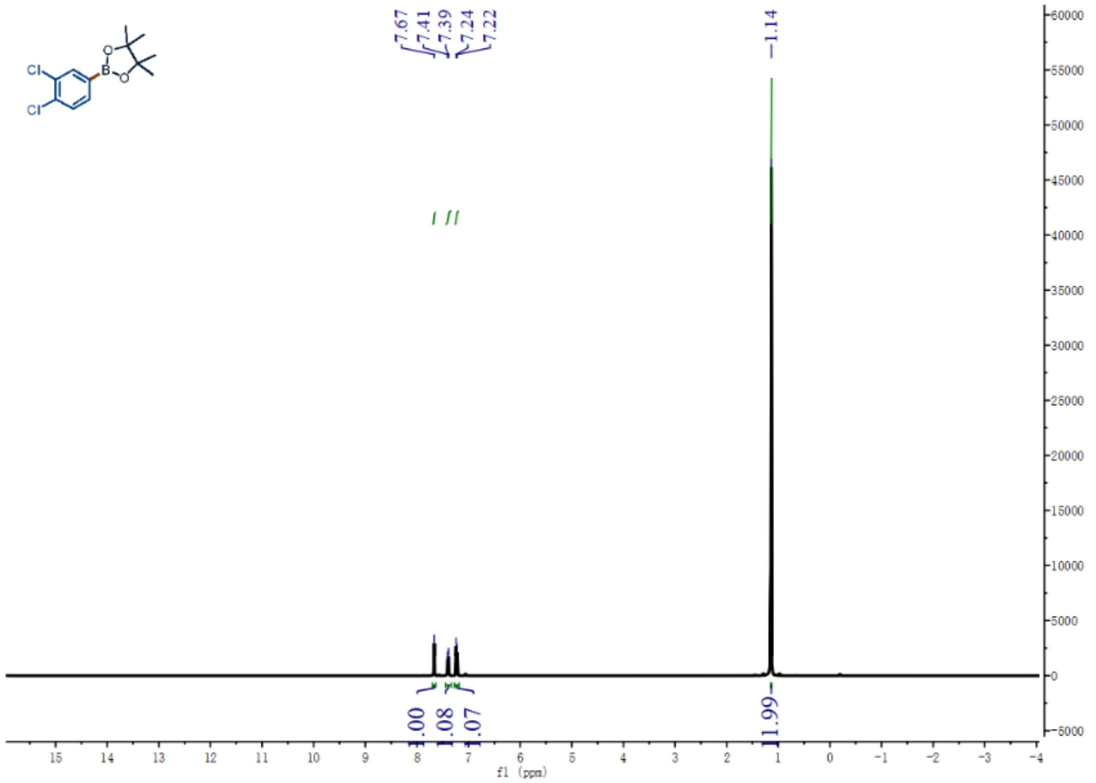

^{13}C NMR (100 MHz, $CDCl_3$) spectrum of compound **3r**

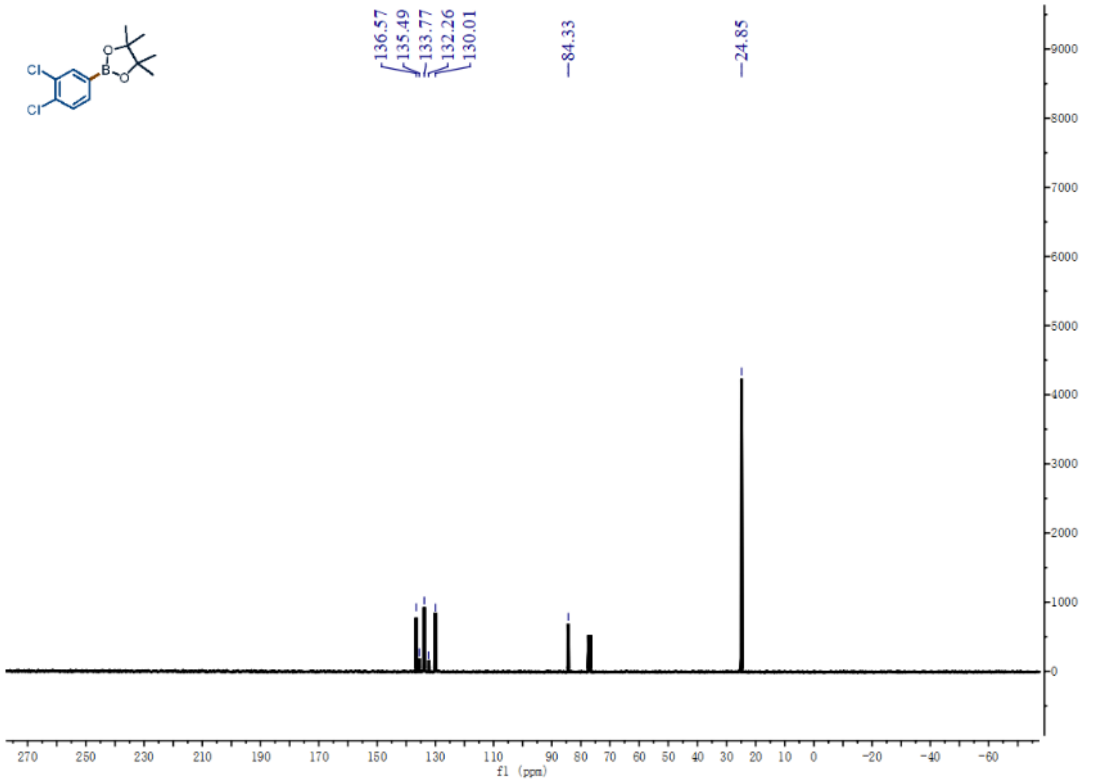

^{11}B NMR (376 MHz, $CDCl_3$) spectrum of compound **3r**

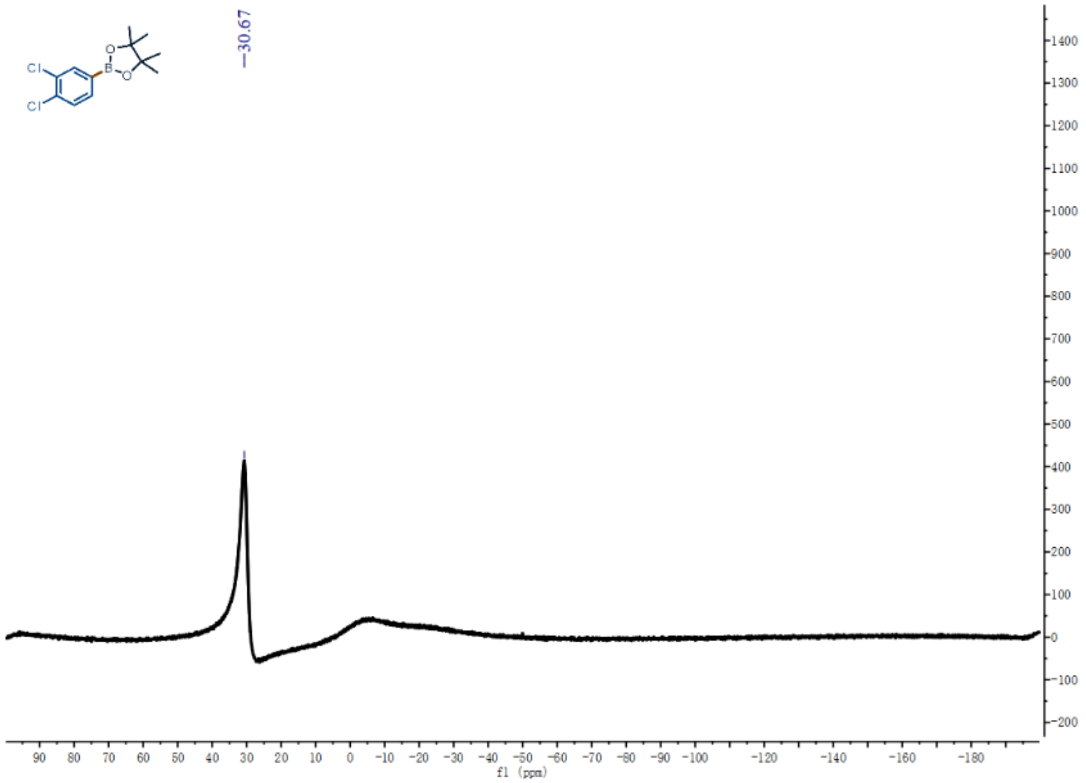

^{1}H NMR (400 MHz, $CDCl_3$) spectrum of compound **3s**

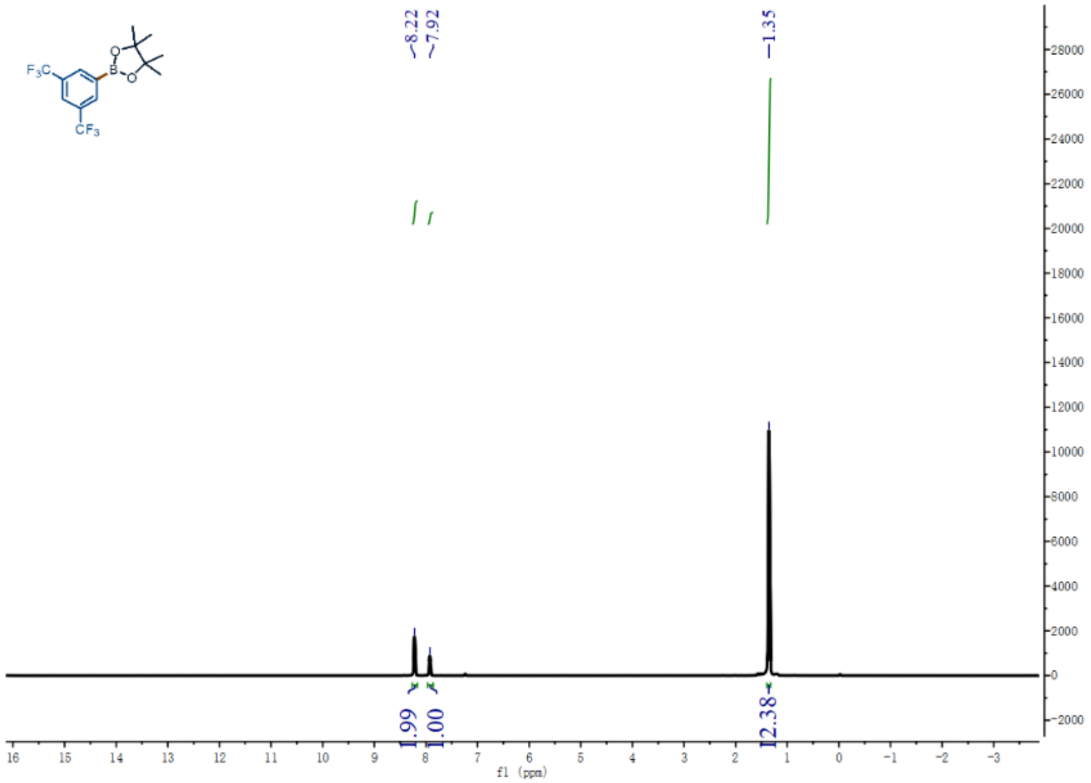

^{13}C NMR (100 MHz, $CDCl_3$) spectrum of compound **3s**

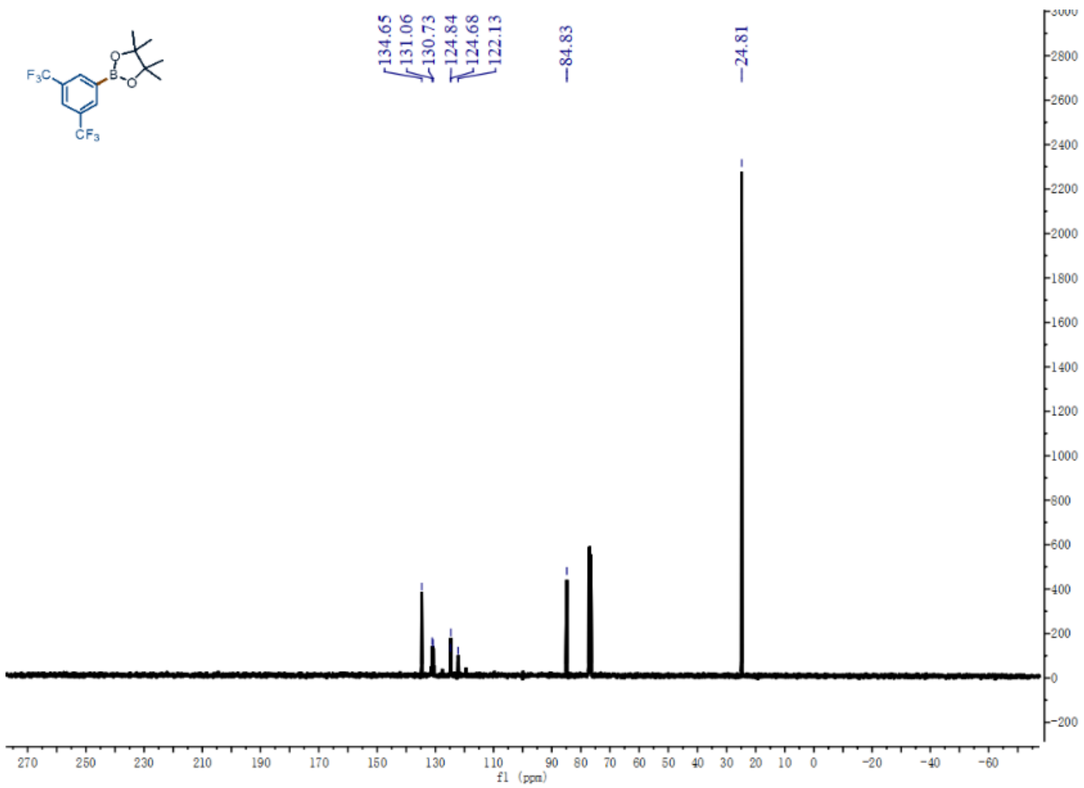

^{11}B NMR (376 MHz, $CDCl_3$) spectrum of compound **3s**

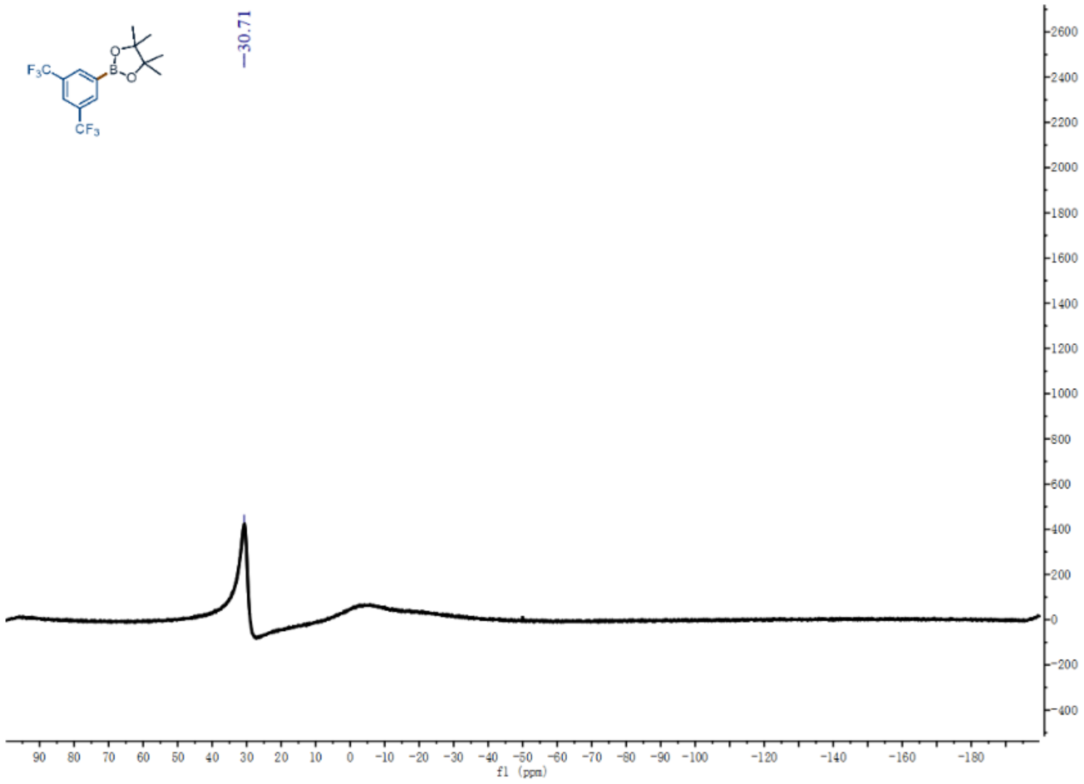

^{1}H NMR (400 MHz, $CDCl_3$) spectrum of compound **3t**

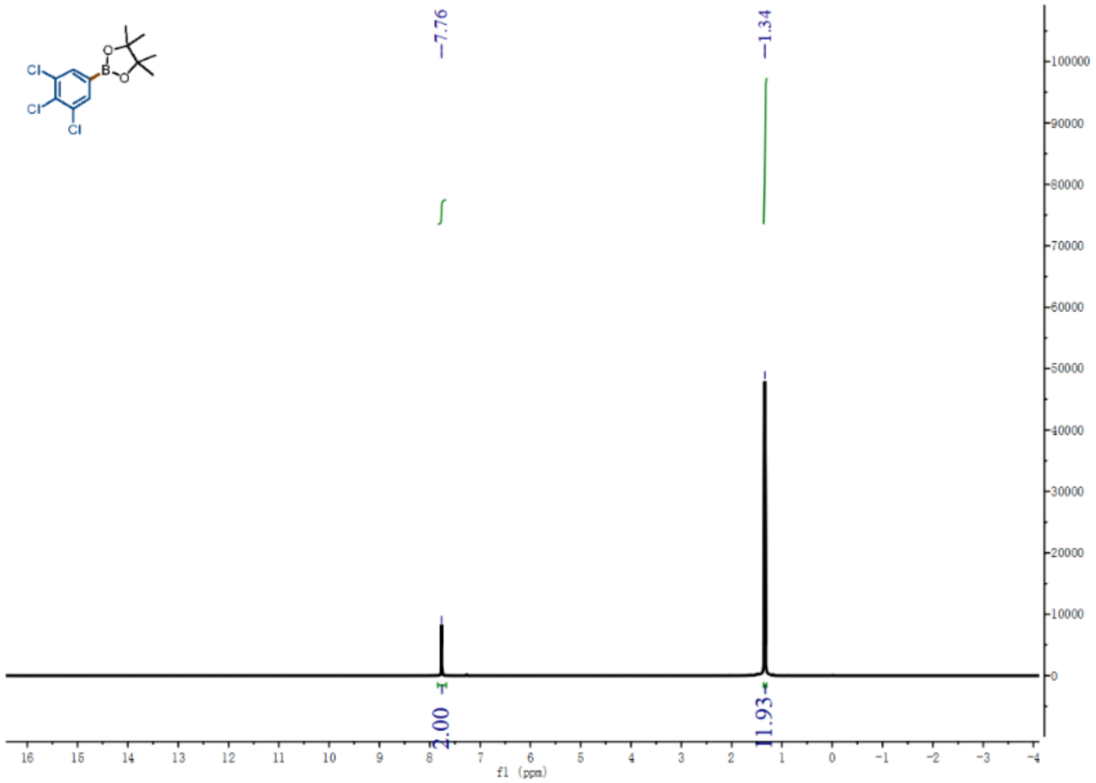

^{13}C NMR (100 MHz, $CDCl_3$) spectrum of compound **3t**

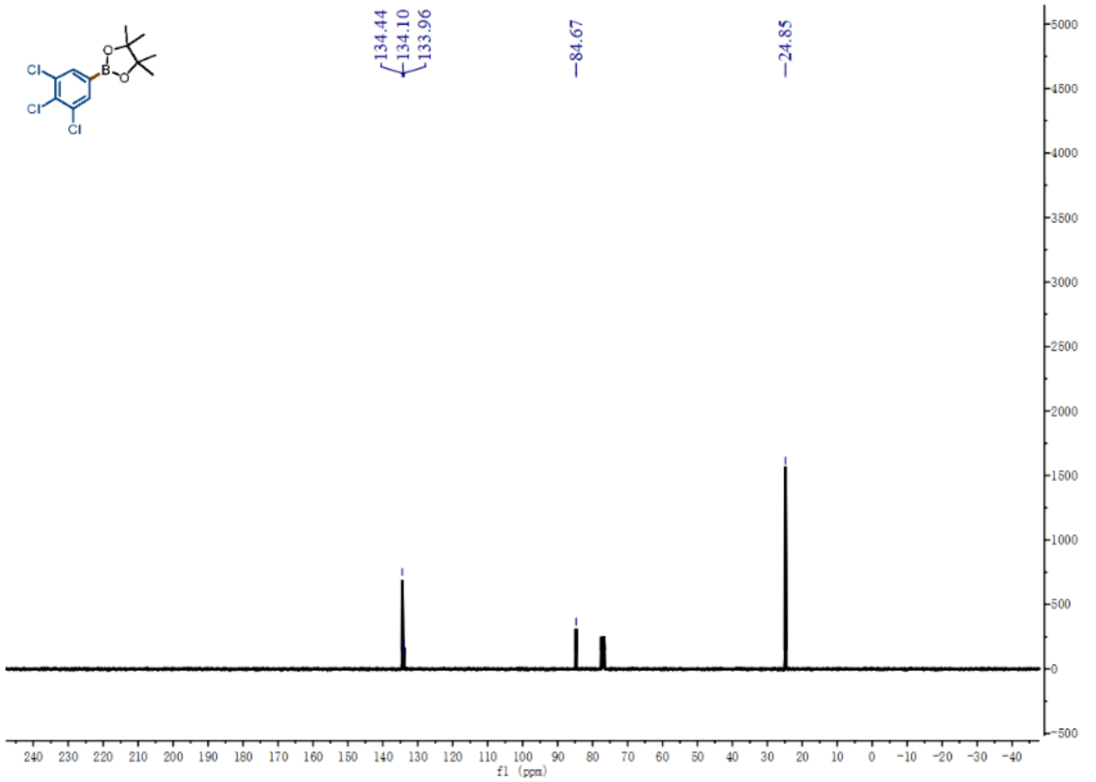

^{11}B NMR (376 MHz, $CDCl_3$) spectrum of compound **3t**

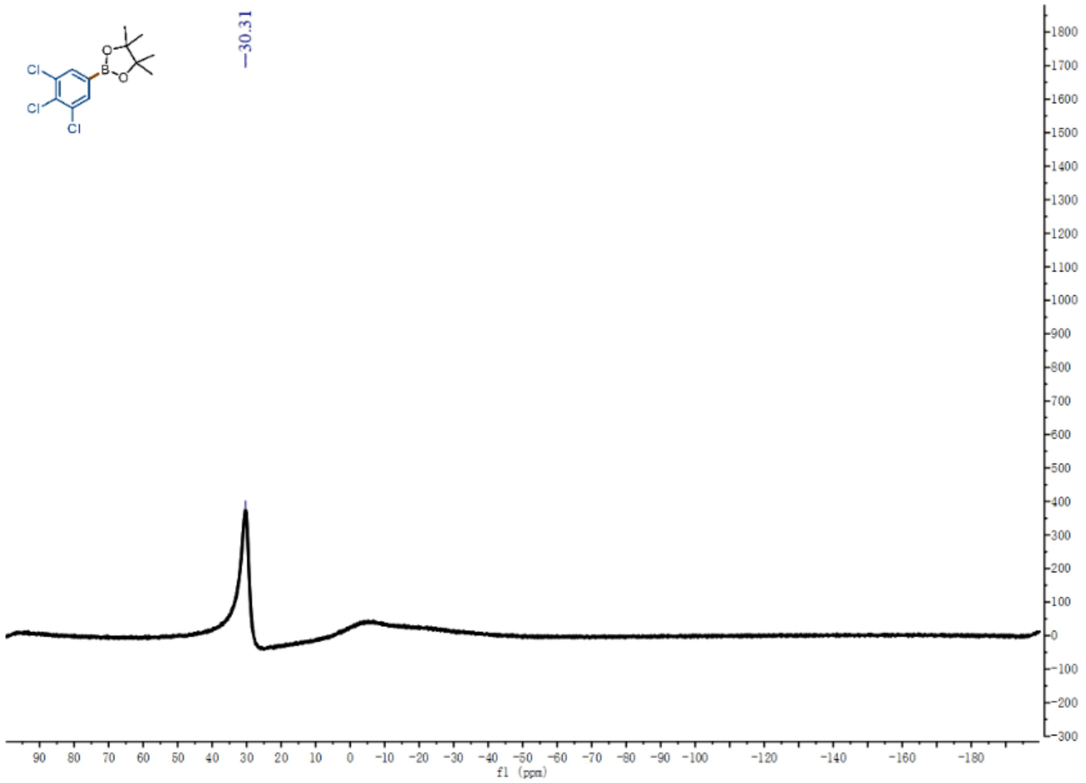

^{1}H NMR (400 MHz, $CDCl_3$) spectrum of compound **3u**

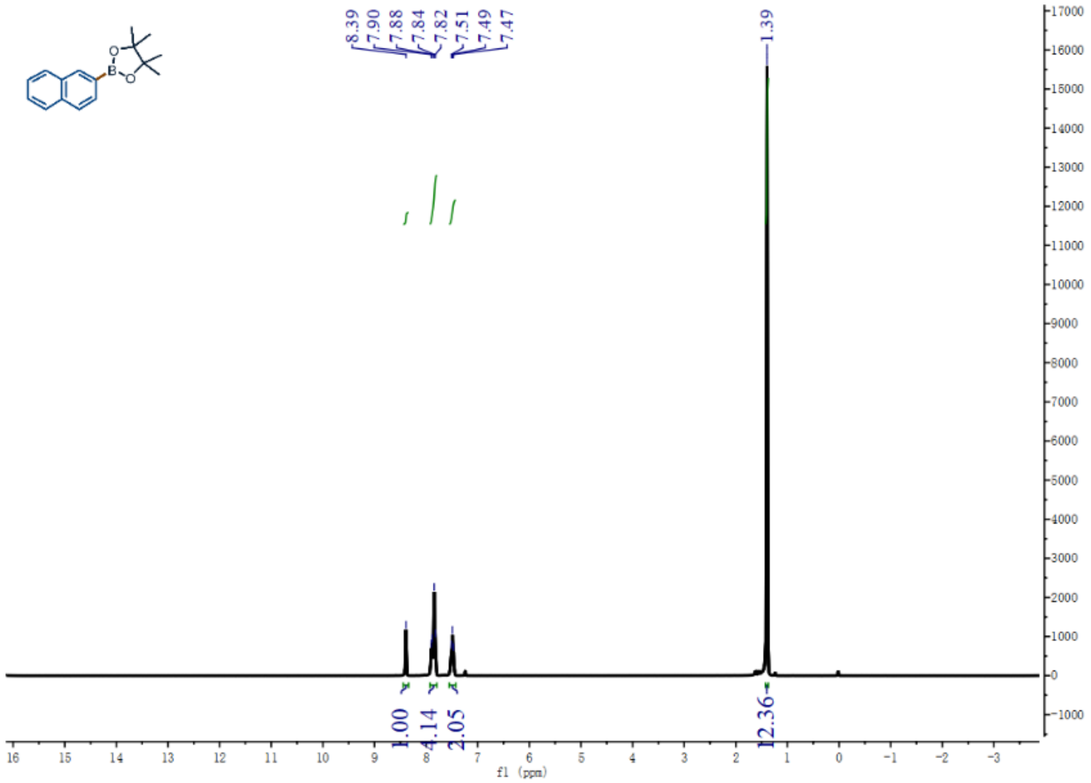

^{13}C NMR (100 MHz, $CDCl_3$) spectrum of compound **3u**

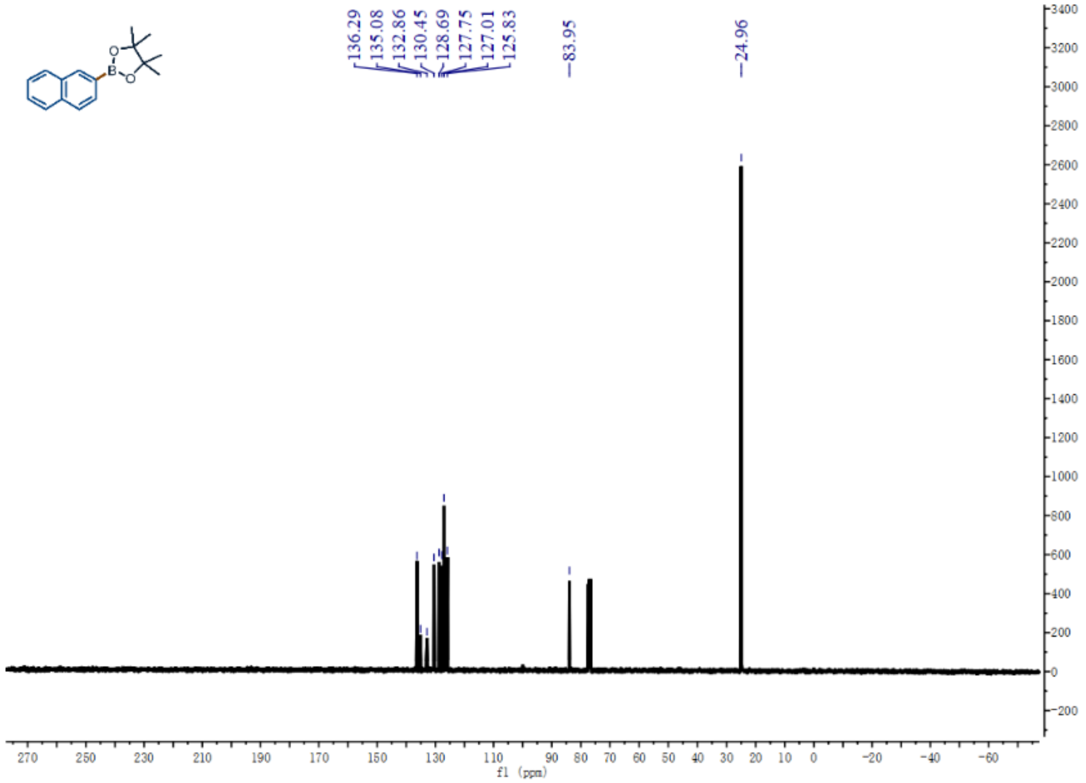

^{11}B NMR (376 MHz, $CDCl_3$) spectrum of compound **3u**

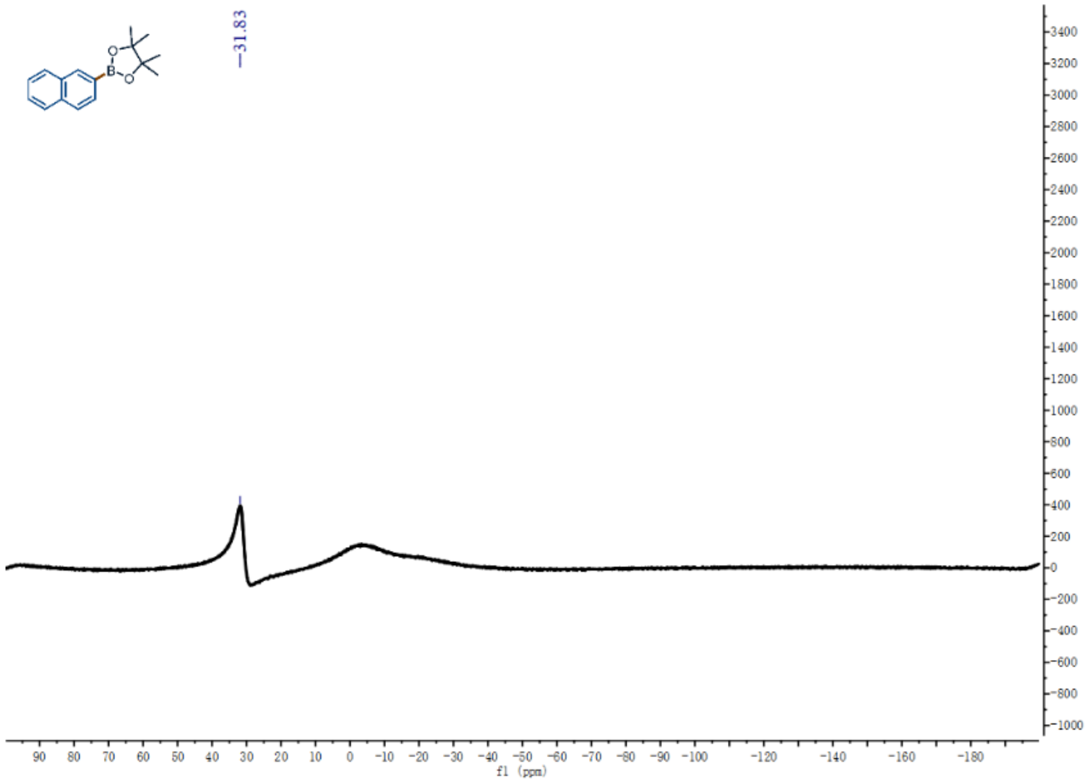

^{1}H NMR (400 MHz, $CDCl_3$) spectrum of compound **5a**

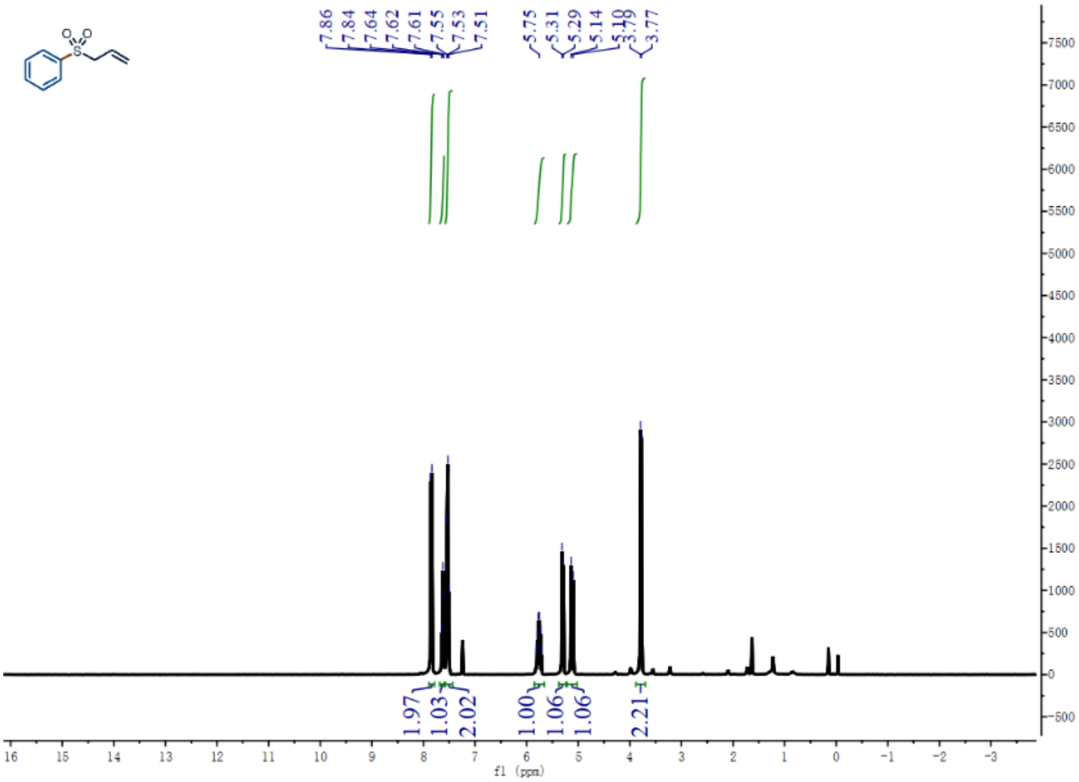

^{13}C NMR (100 MHz, $CDCl_3$) spectrum of compound **5a**

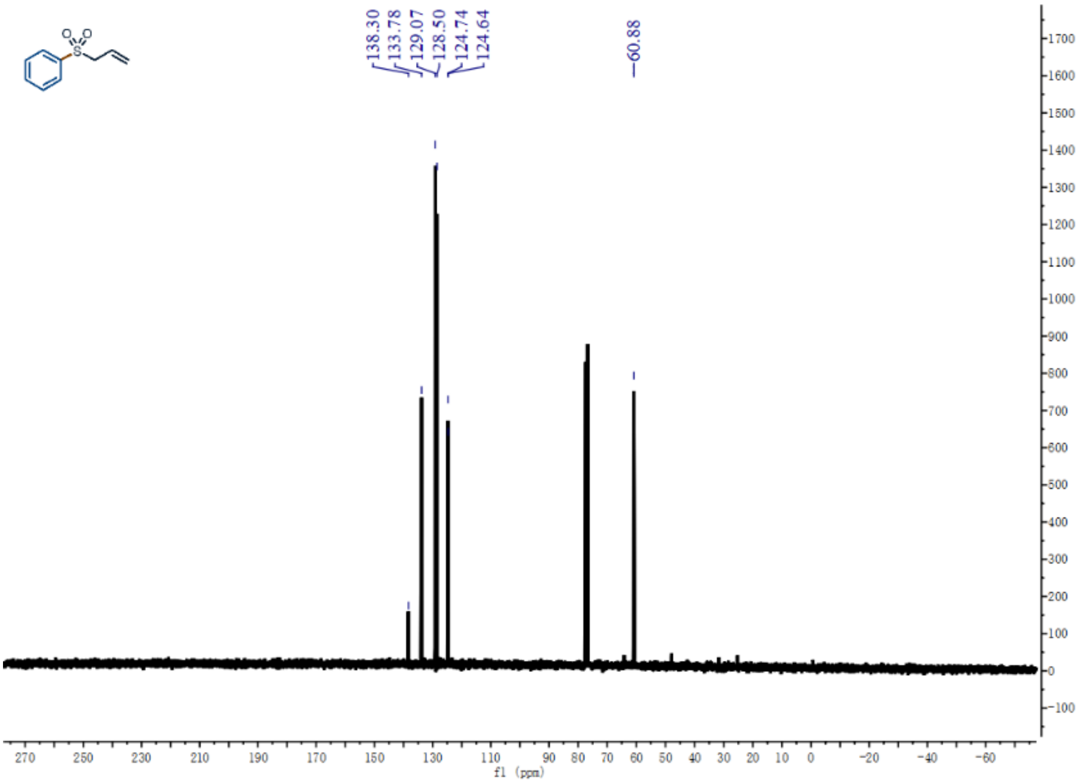

^{1}H NMR (400 MHz, $CDCl_3$) spectrum of compound **5b**

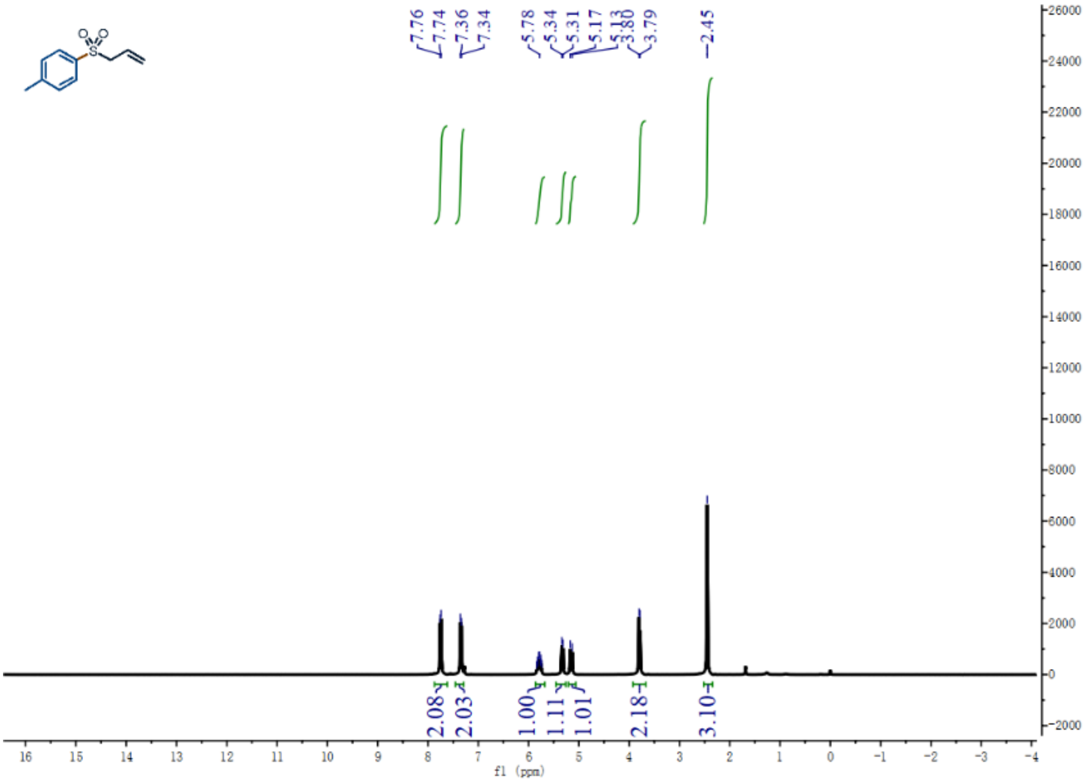

^{13}C NMR (100 MHz, $CDCl_3$) spectrum of compound **5b**

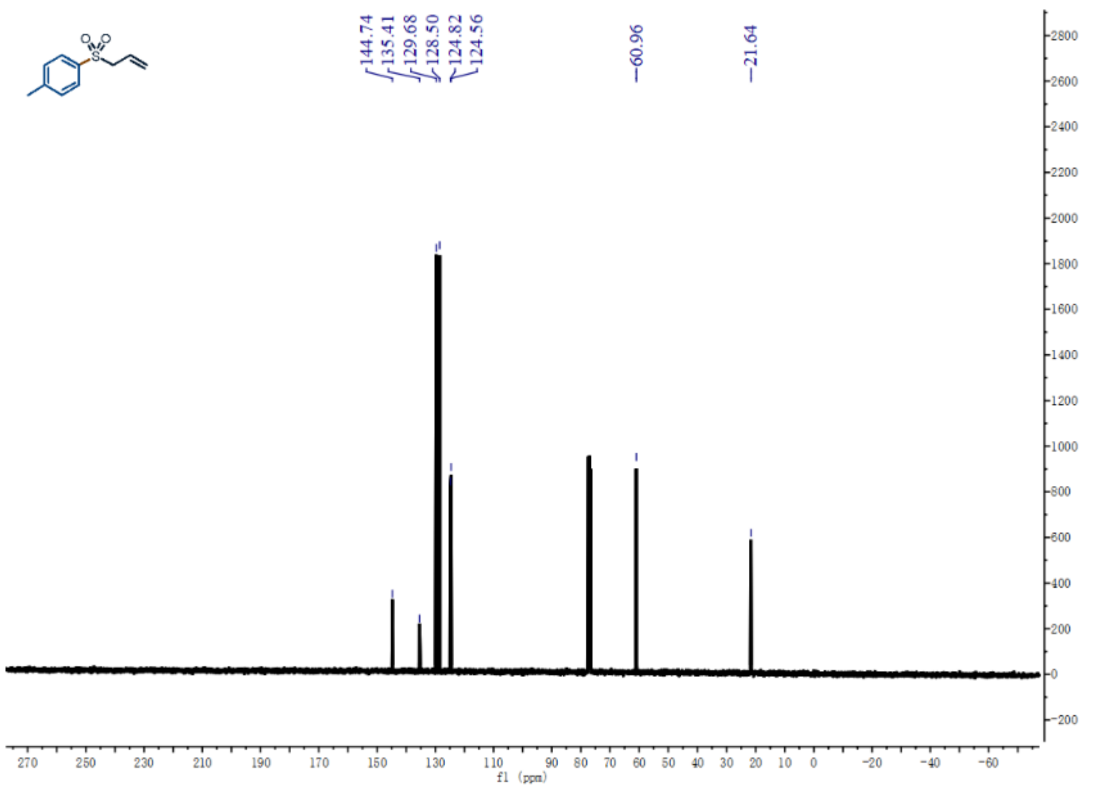

^{1}H NMR (400 MHz, $CDCl_3$) spectrum of compound **5c**

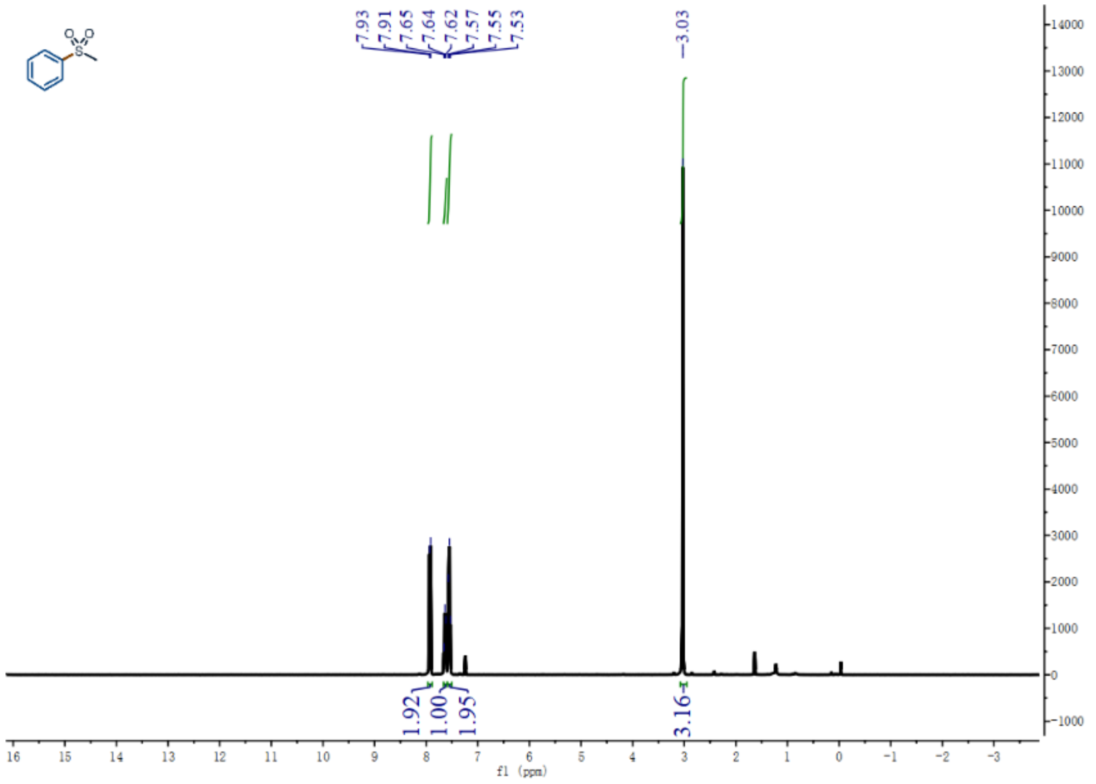

^{13}C NMR (100 MHz, $CDCl_3$) spectrum of compound **5c**

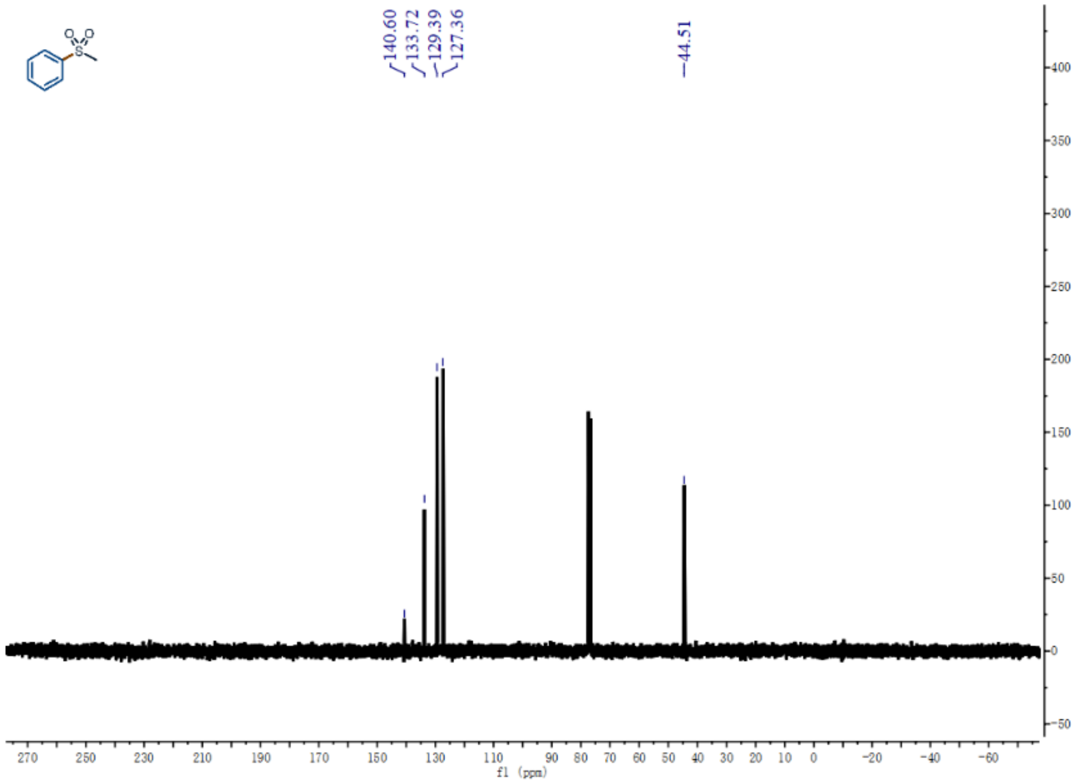

^{1}H NMR (400 MHz, $CDCl_3$) spectrum of compound **5d**

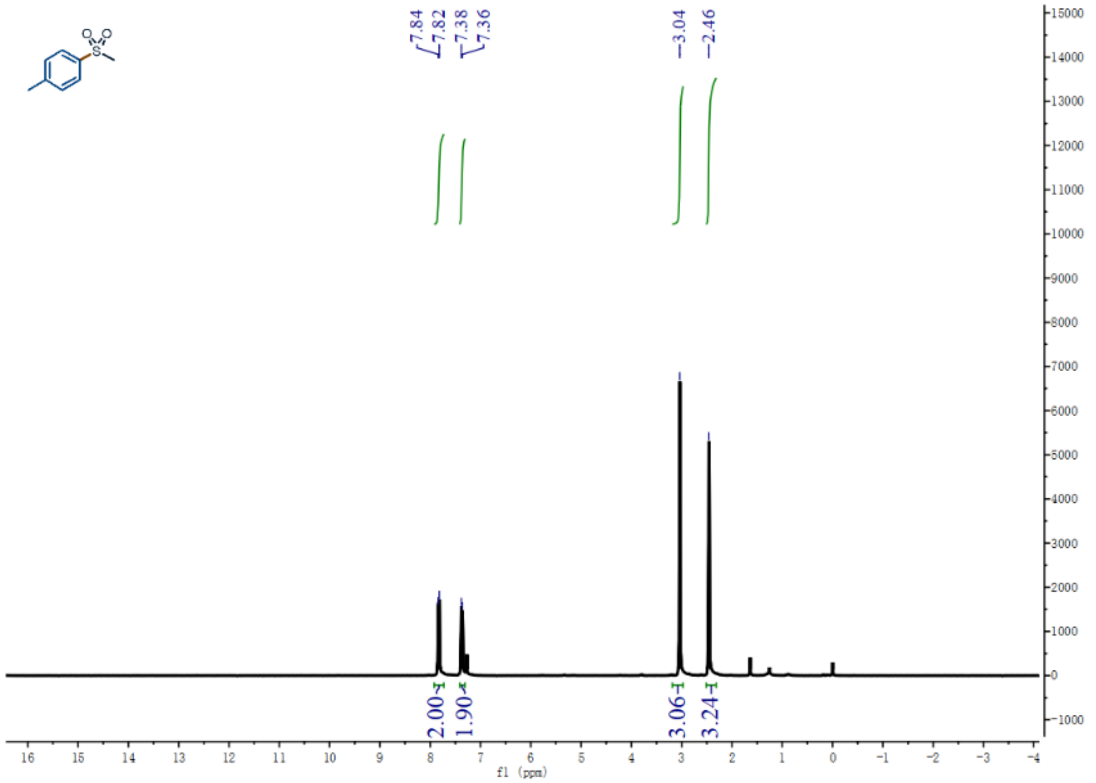

^{13}C NMR (100 MHz, $CDCl_3$) spectrum of compound **5d**

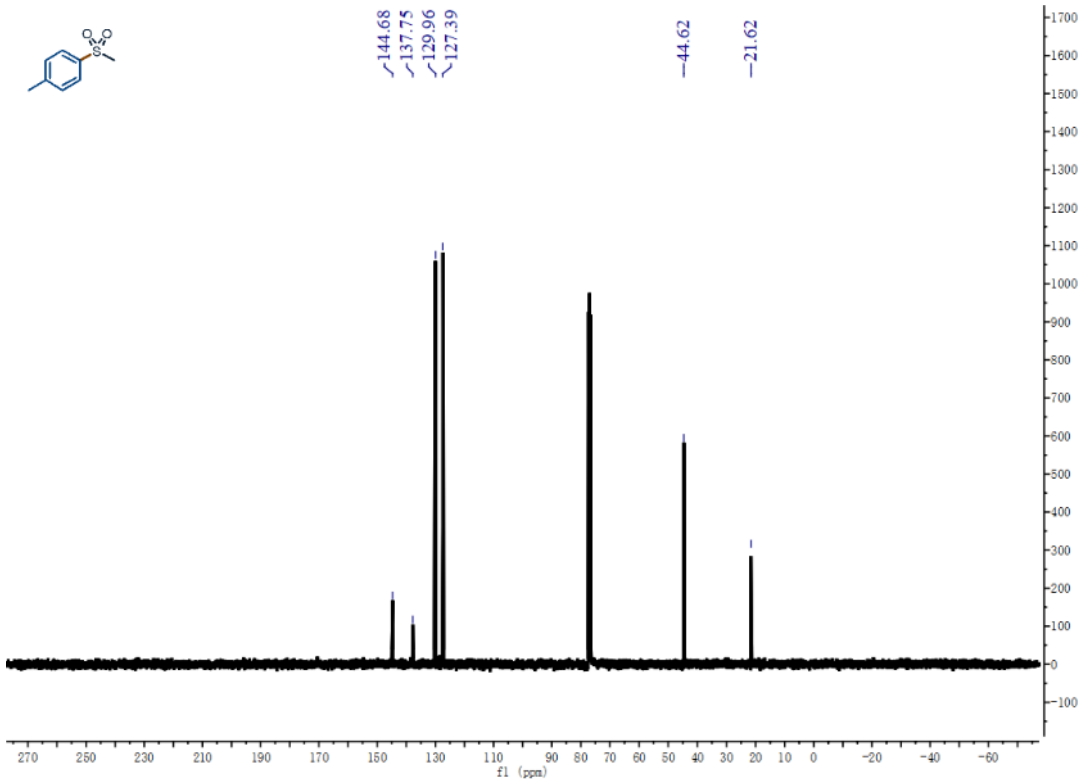

^{1}H NMR (400 MHz, $CDCl_3$) spectrum of compound **5e**

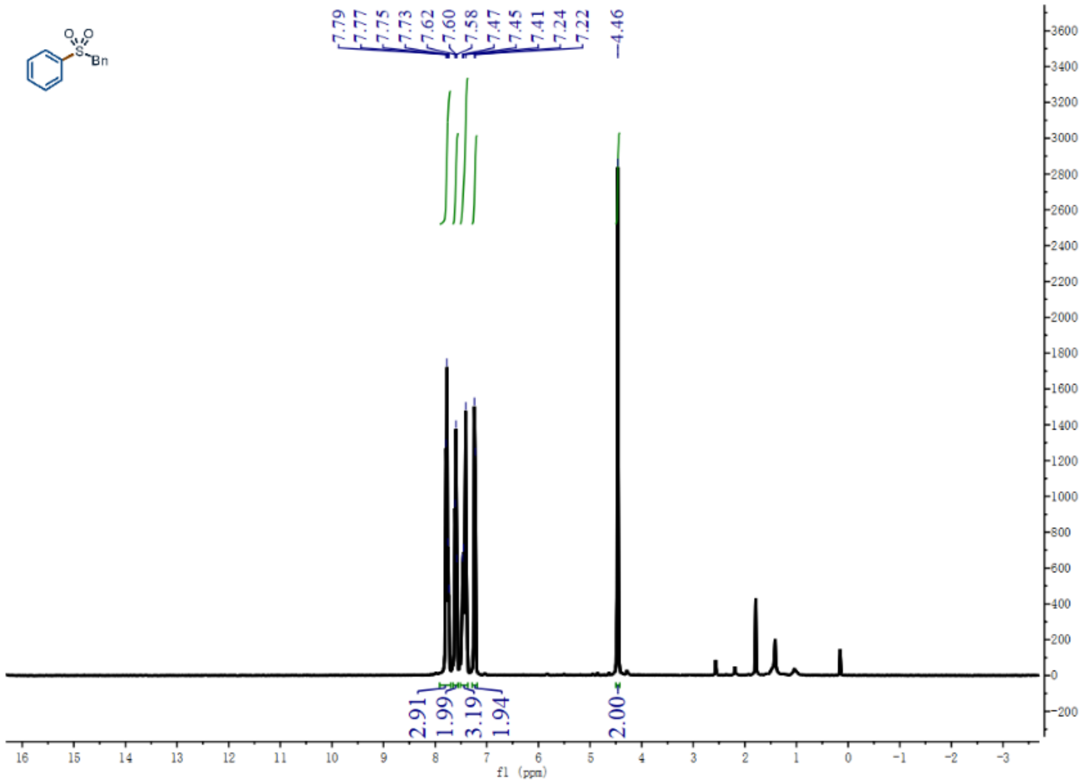

^{13}C NMR (100 MHz, $CDCl_3$) spectrum of compound **5e**

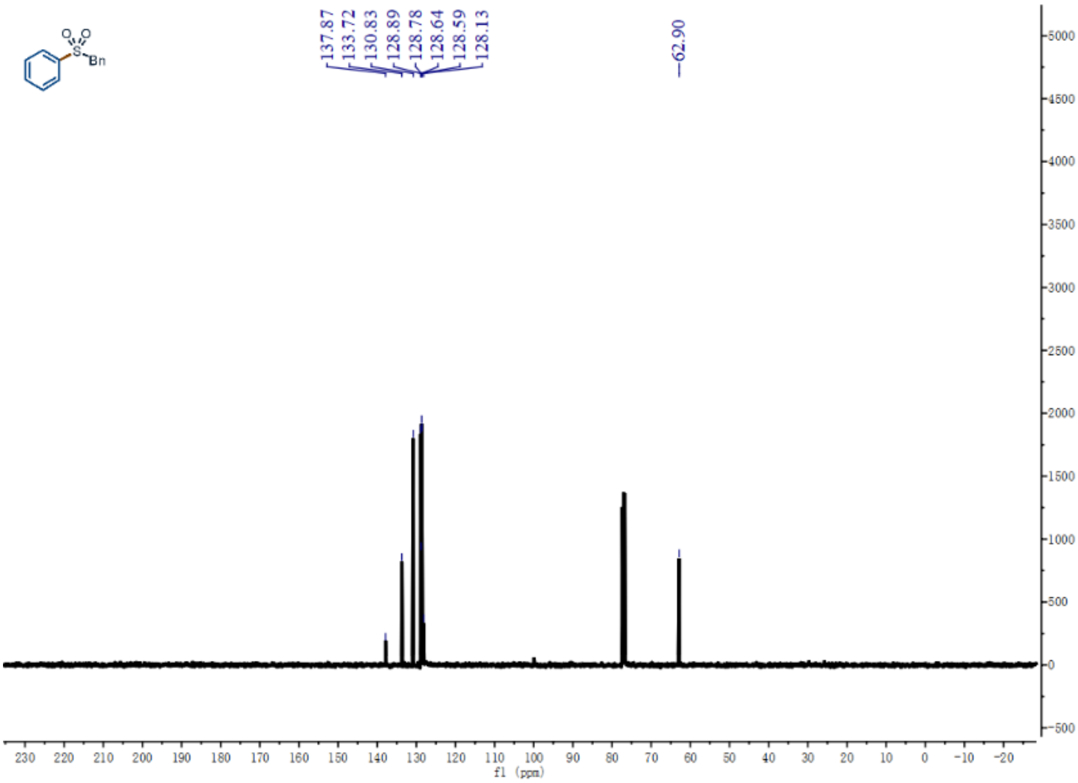

^{1}H NMR (400 MHz, $CDCl_3$) spectrum of compound **5f**

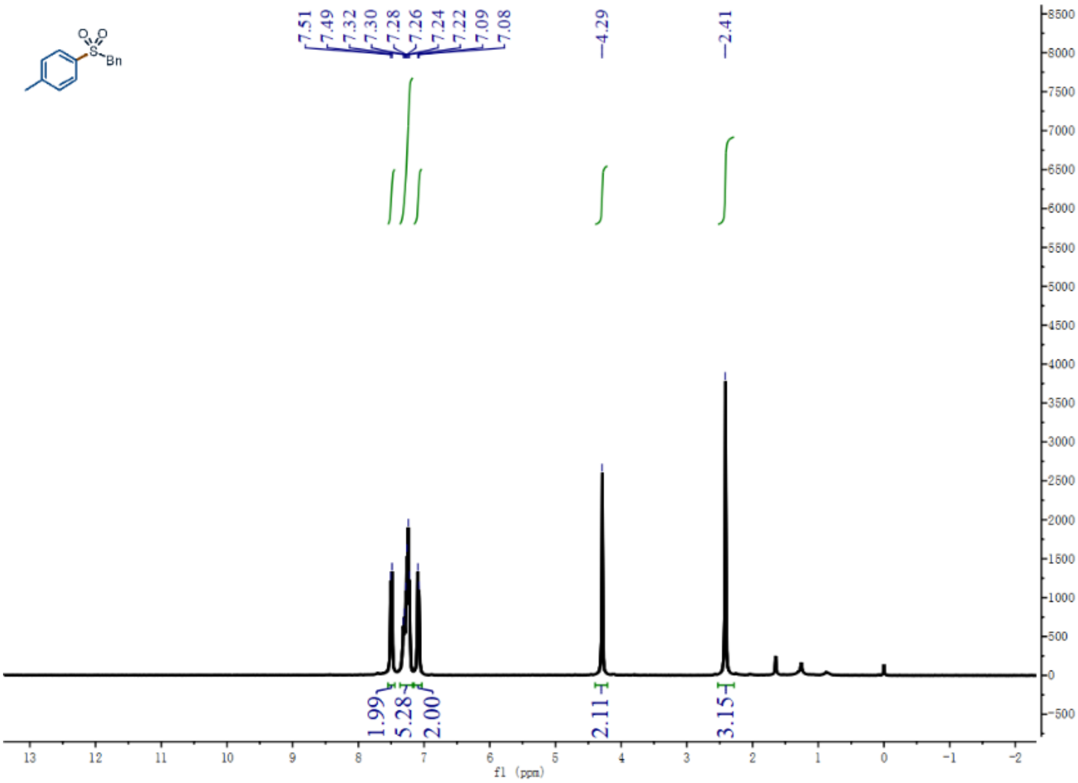

^{13}C NMR (100 MHz, $CDCl_3$) spectrum of compound **5f**

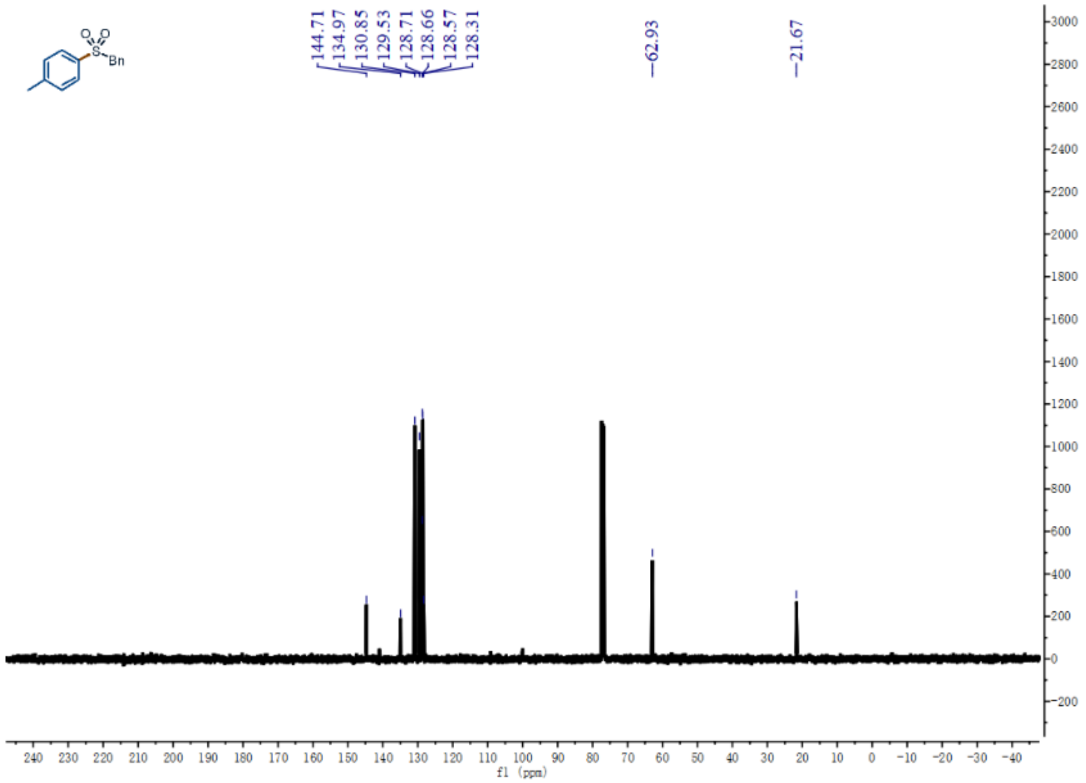

^{1}H NMR (400 MHz, $CDCl_3$) spectrum of compound **5g**

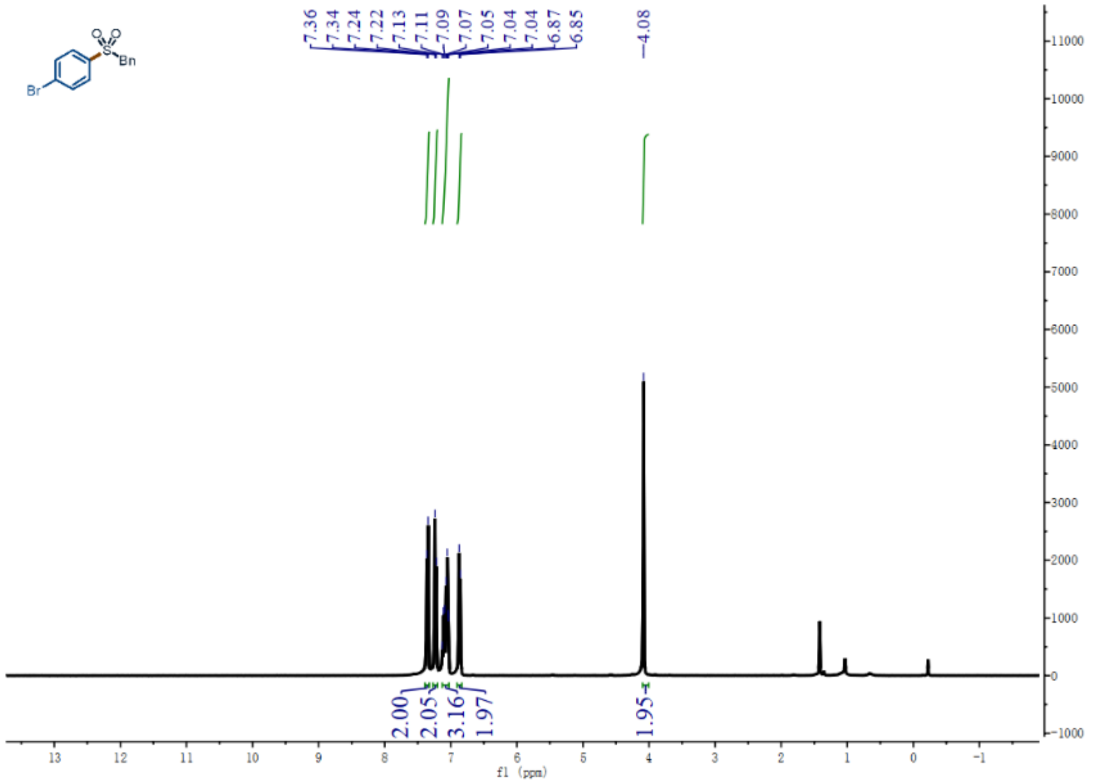

^{13}C NMR (100 MHz, $CDCl_3$) spectrum of compound **5g**

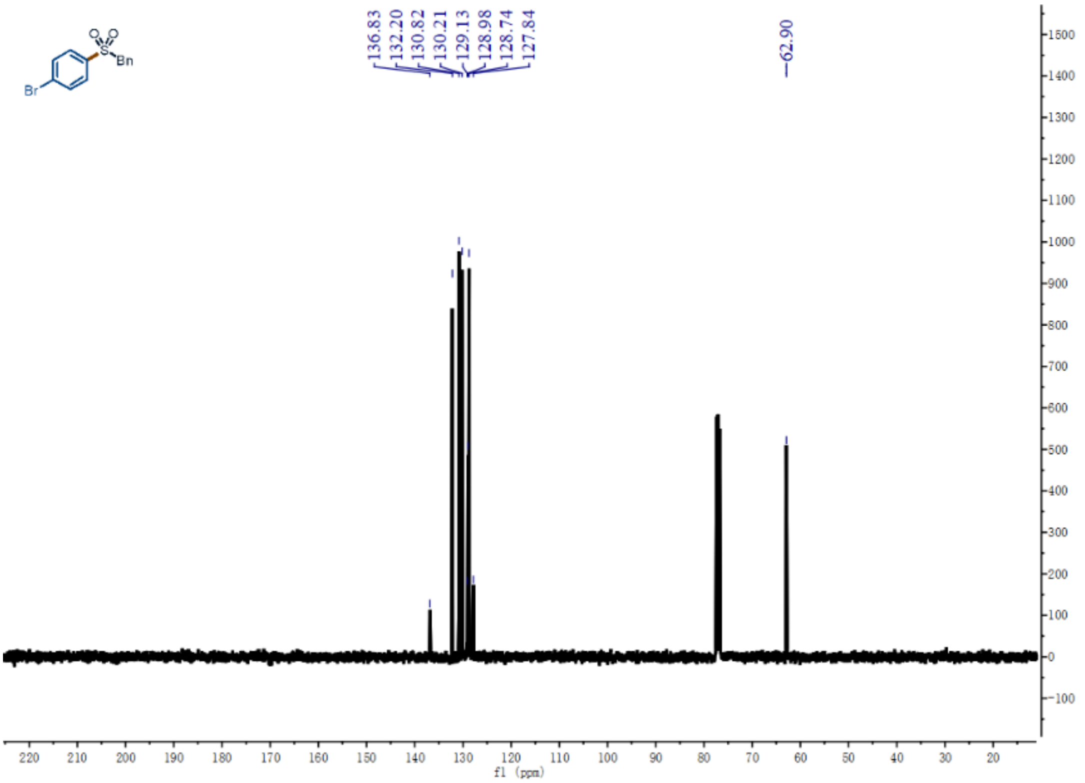

^{1}H NMR (400 MHz, $CDCl_3$) spectrum of compound **5h**

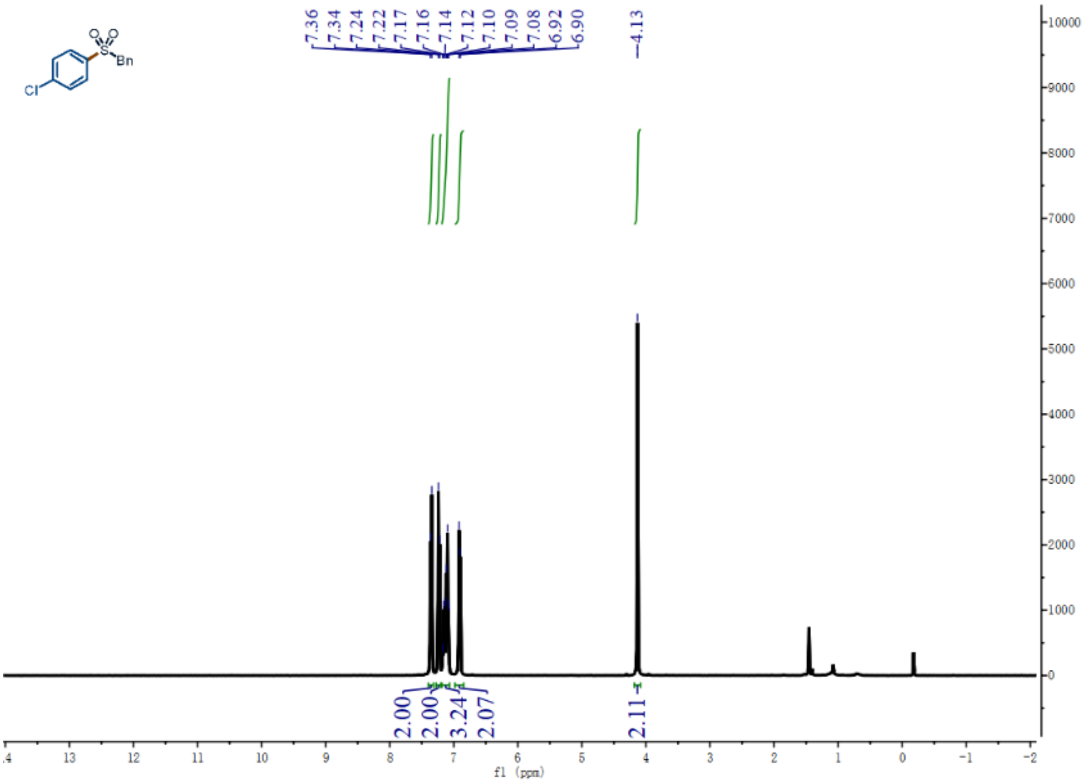

^{13}C NMR (100 MHz, $CDCl_3$) spectrum of compound **5h**

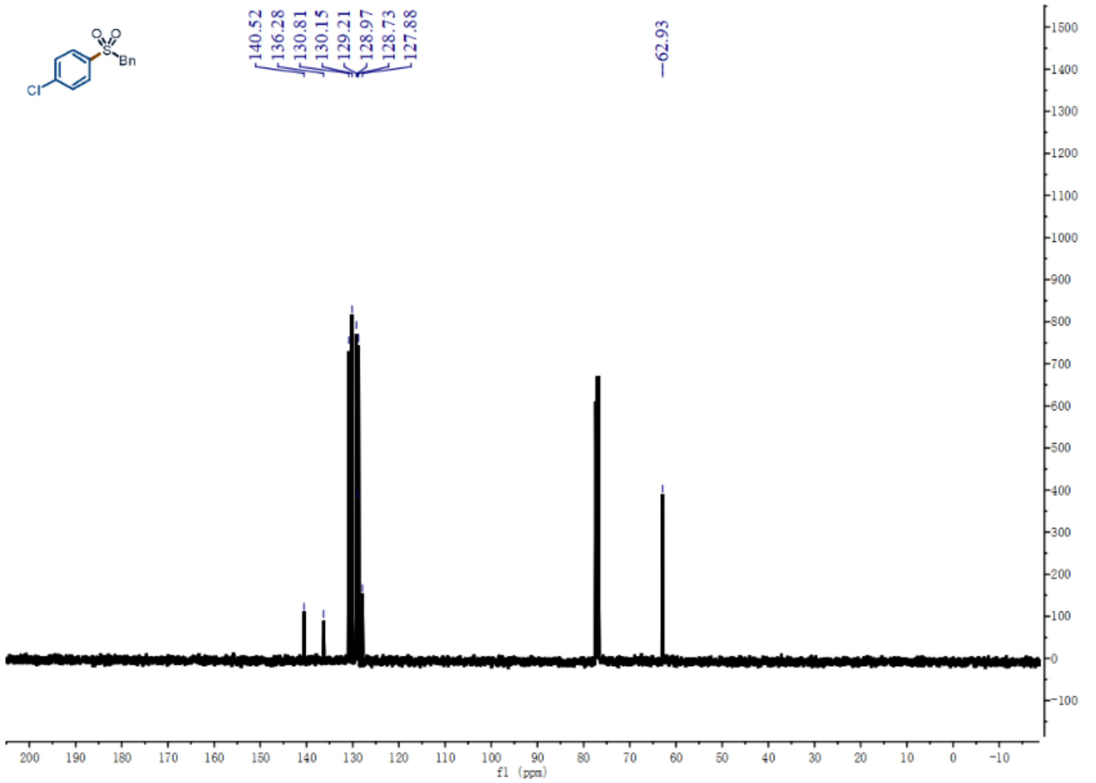

^{1}H NMR (400 MHz, $CDCl_3$) spectrum of compound **5i**

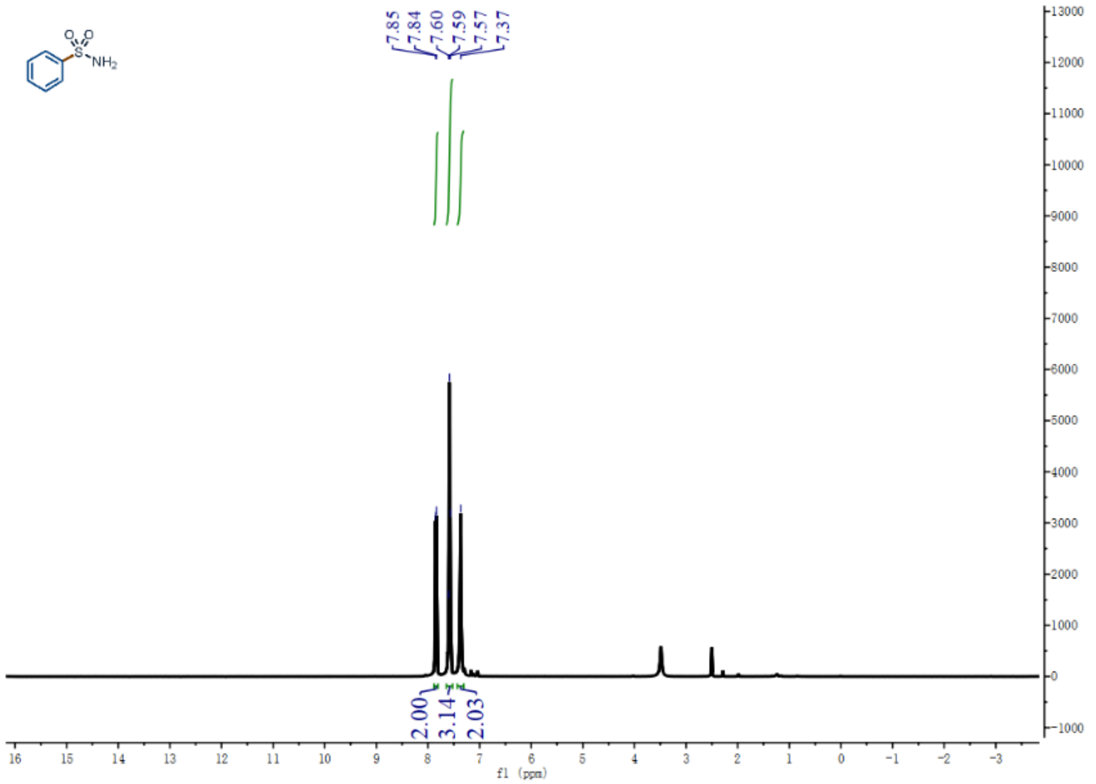

^{13}C NMR (100 MHz, $CDCl_3$) spectrum of compound **5i**

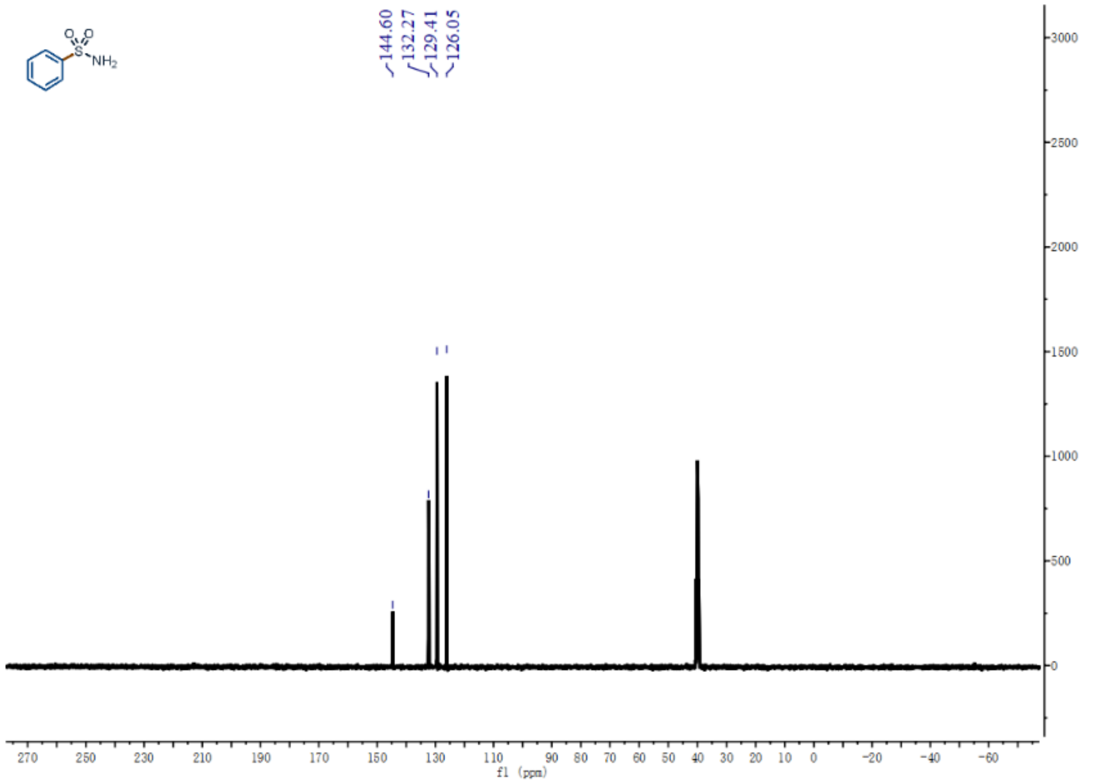

^{1}H NMR (400 MHz, $CDCl_3$) spectrum of compound **5j**

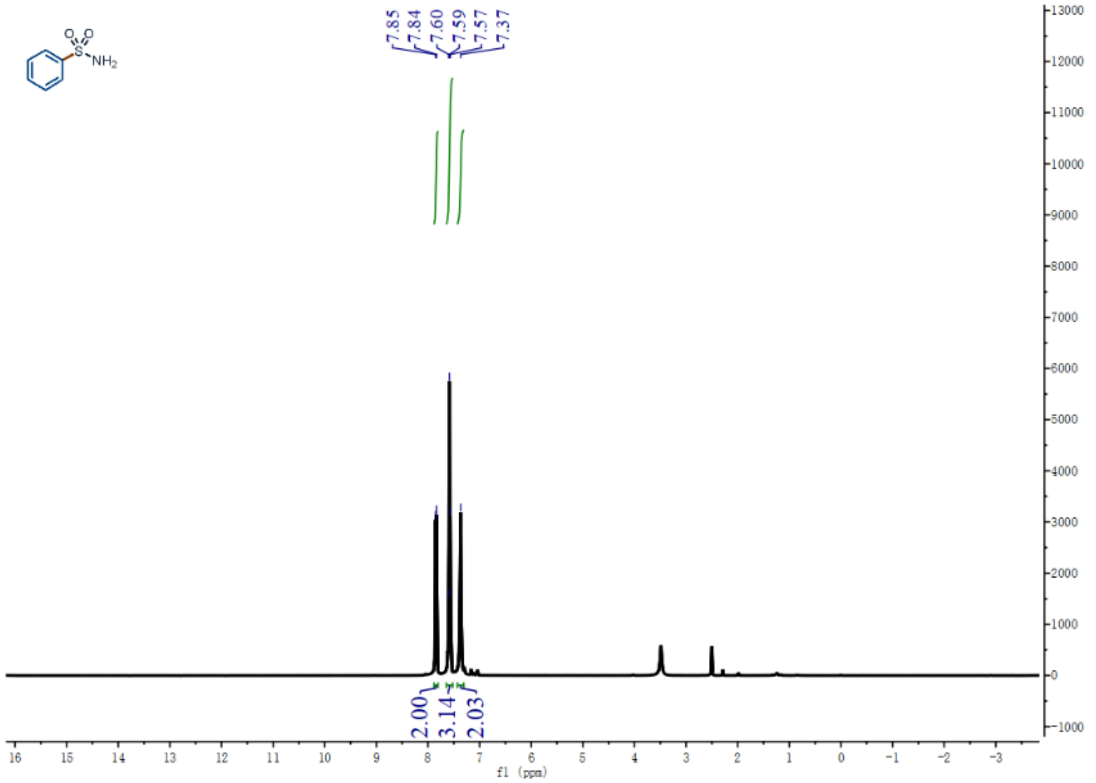

^{13}C NMR (100 MHz, $CDCl_3$) spectrum of compound **5j**

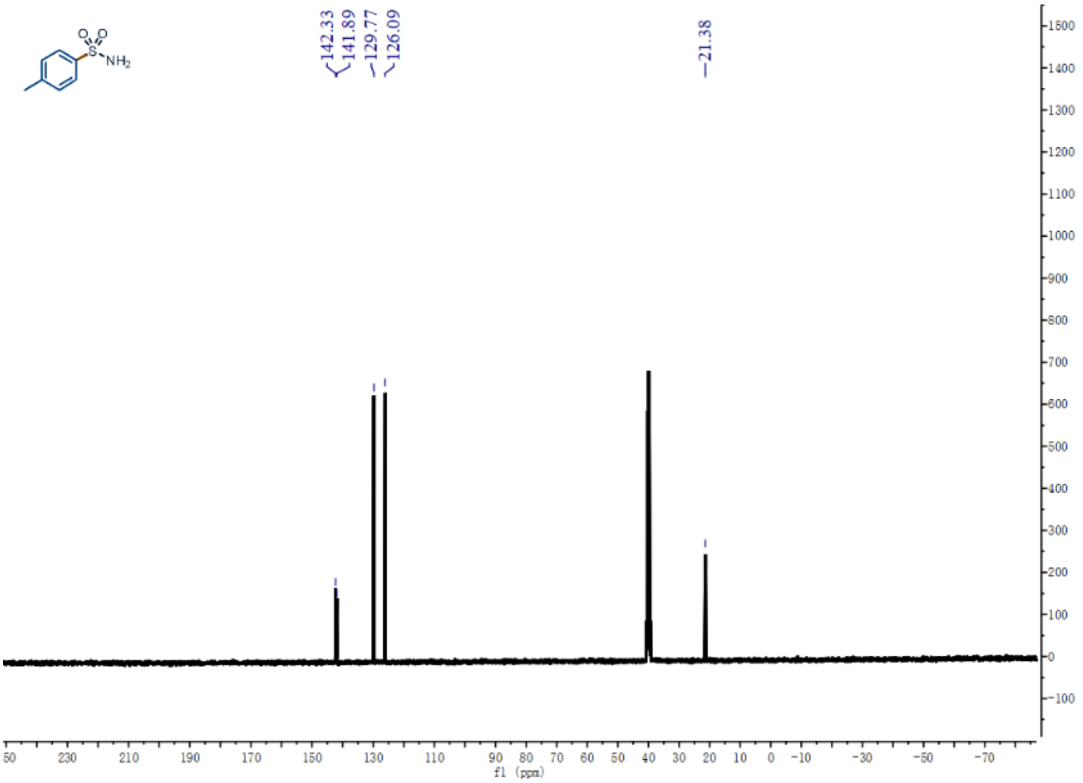

^{1}H NMR (400 MHz, $CDCl_3$) spectrum of compound **5k**

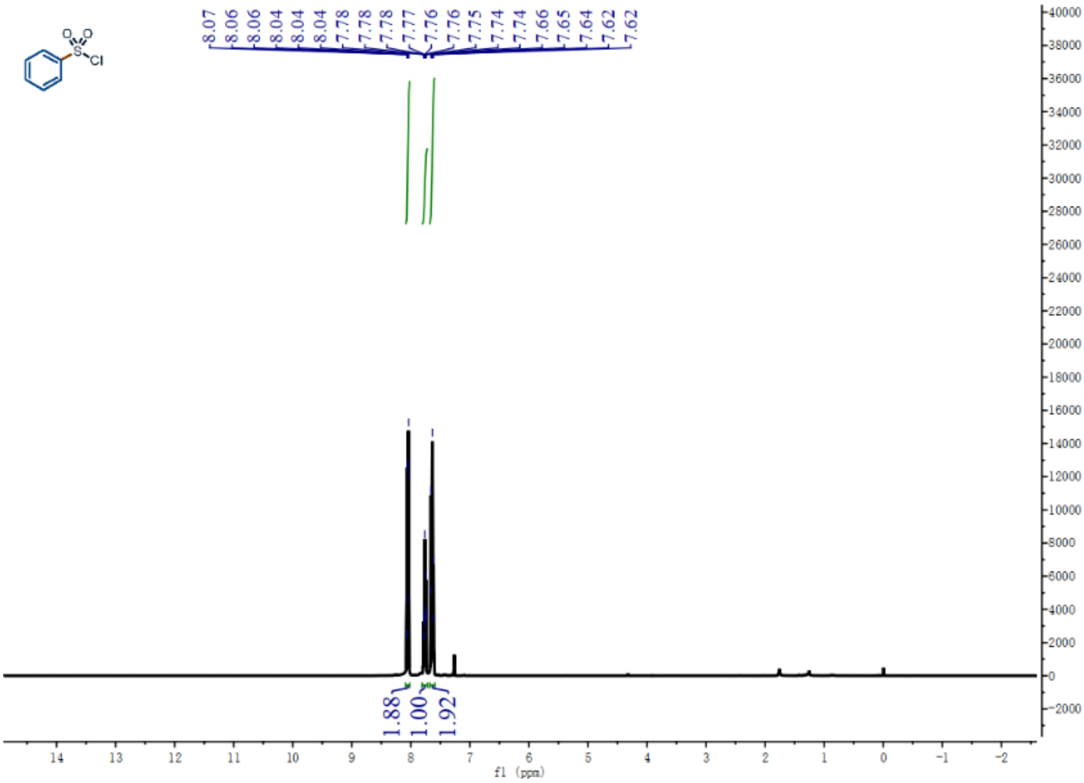

^{13}C NMR (100 MHz, $CDCl_3$) spectrum of compound **5k**

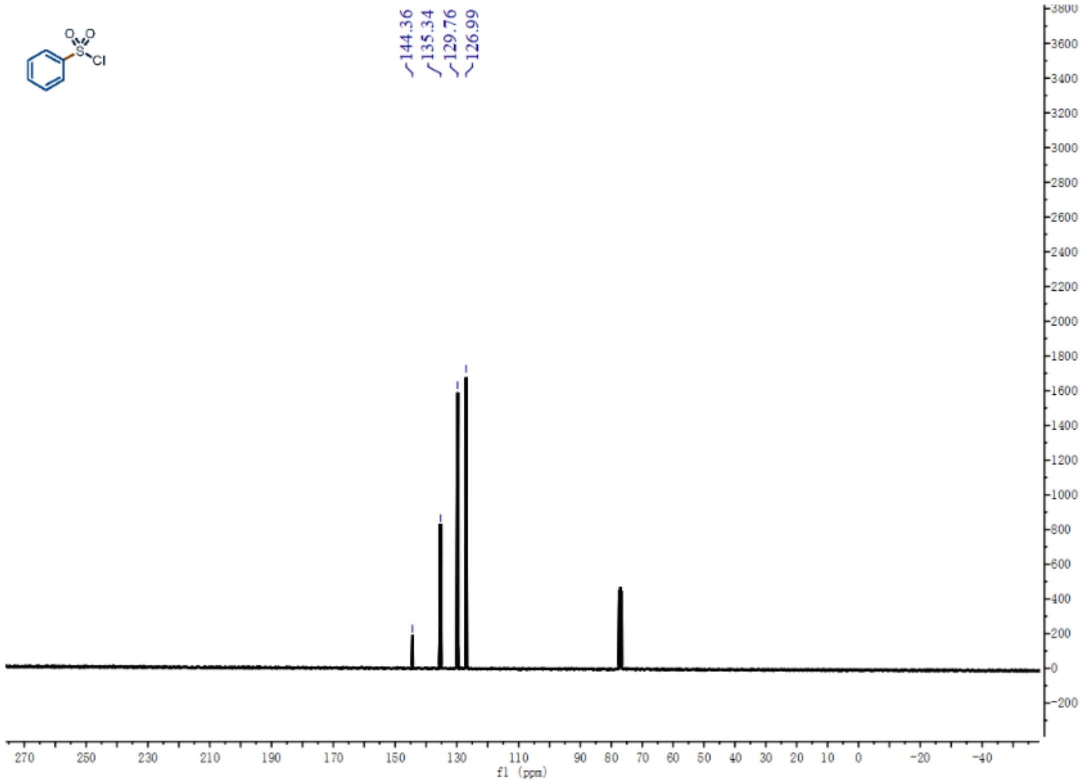

^{1}H NMR (400 MHz, $CDCl_3$) spectrum of compound **5l**

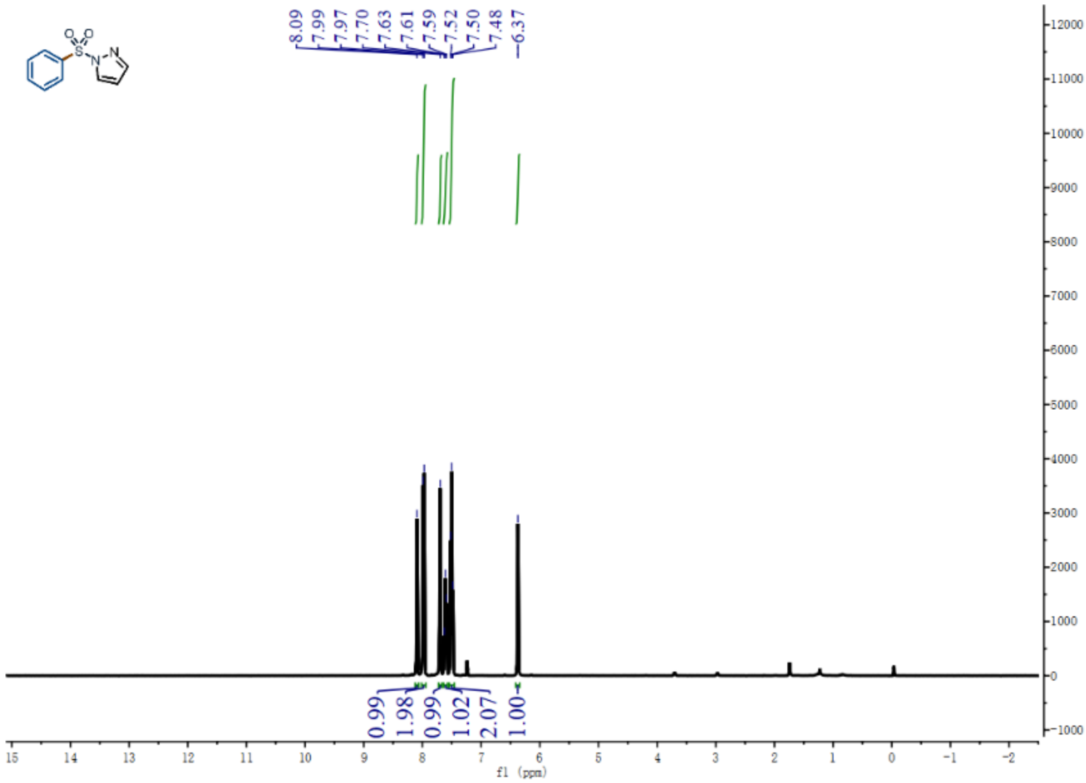

^{13}C NMR (100 MHz, $CDCl_3$) spectrum of compound **5l**

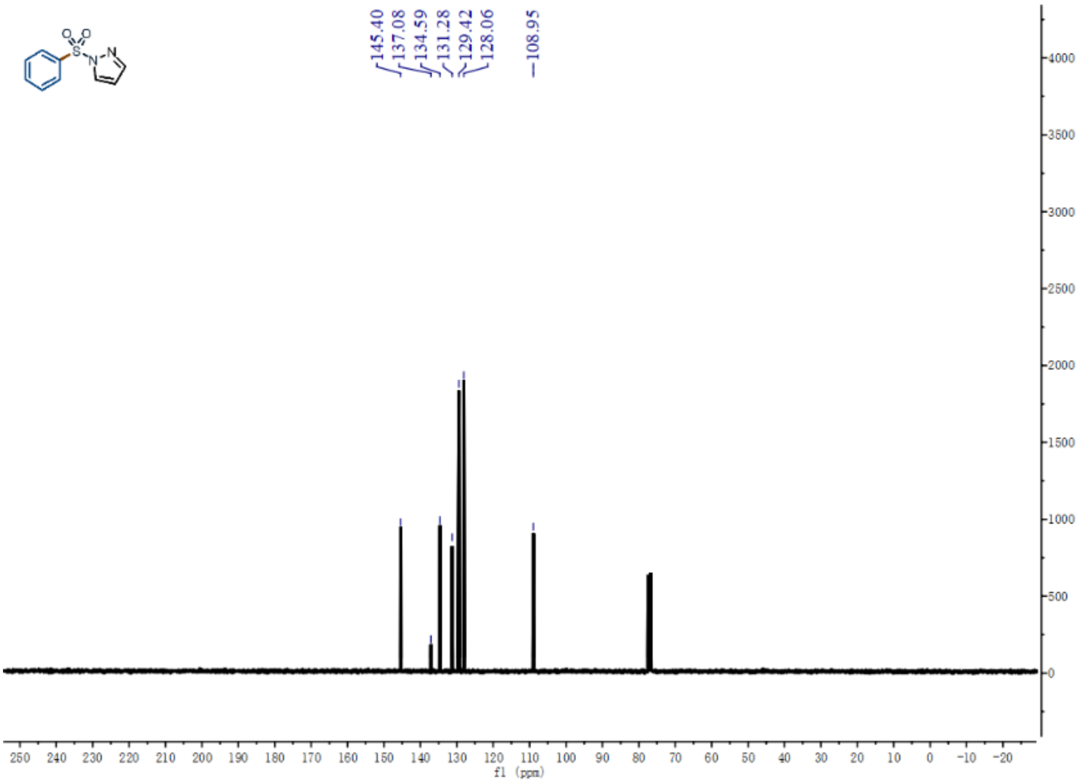

^{1}H NMR (400 MHz, $CDCl_3$) spectrum of compound **5m**

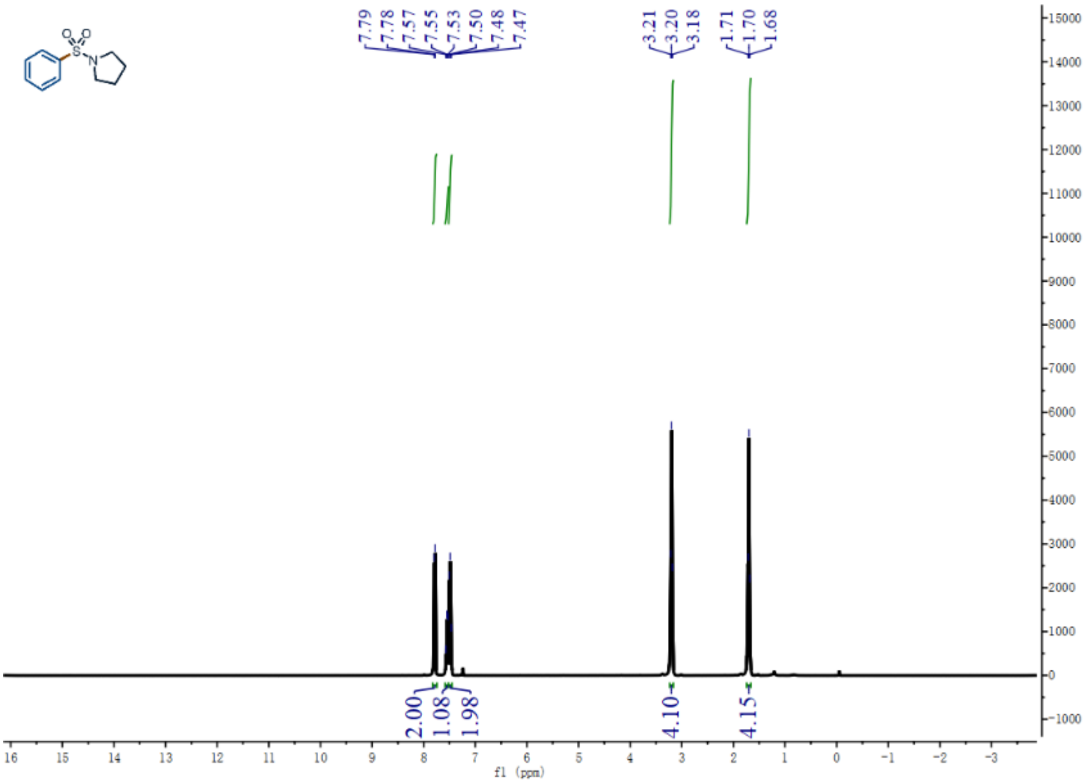

^{13}C NMR (100 MHz, $CDCl_3$) spectrum of compound **5m**

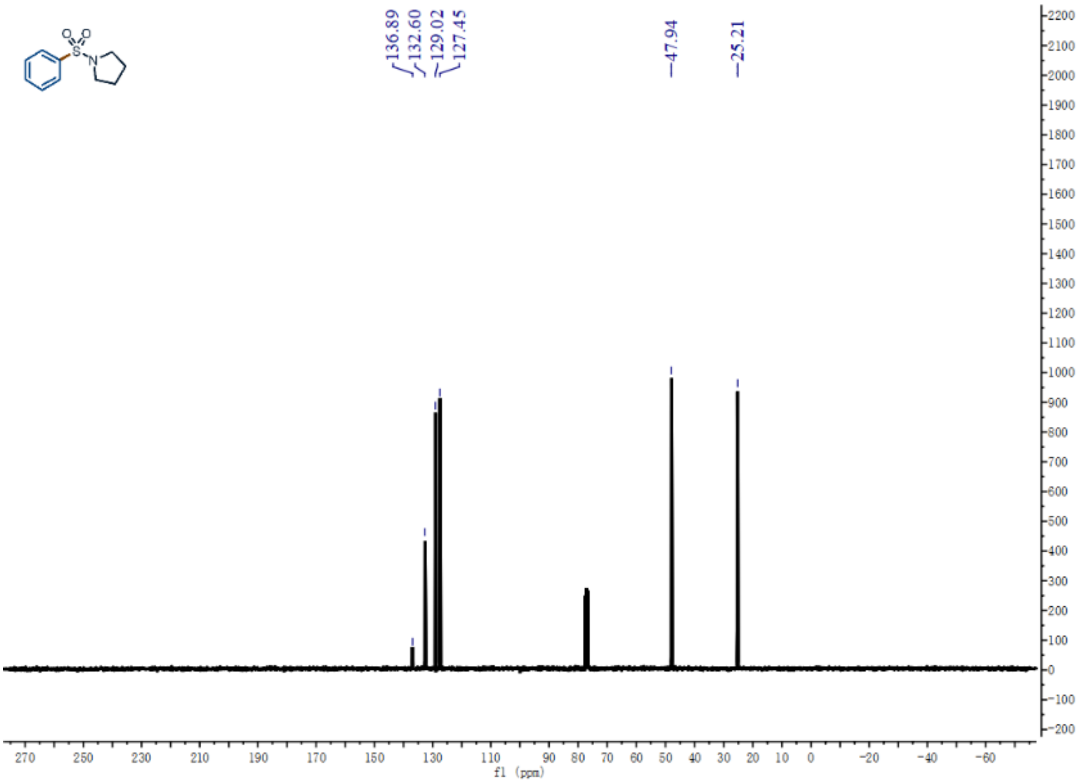

^{1}H NMR (400 MHz, $CDCl_3$) spectrum of compound **5n**

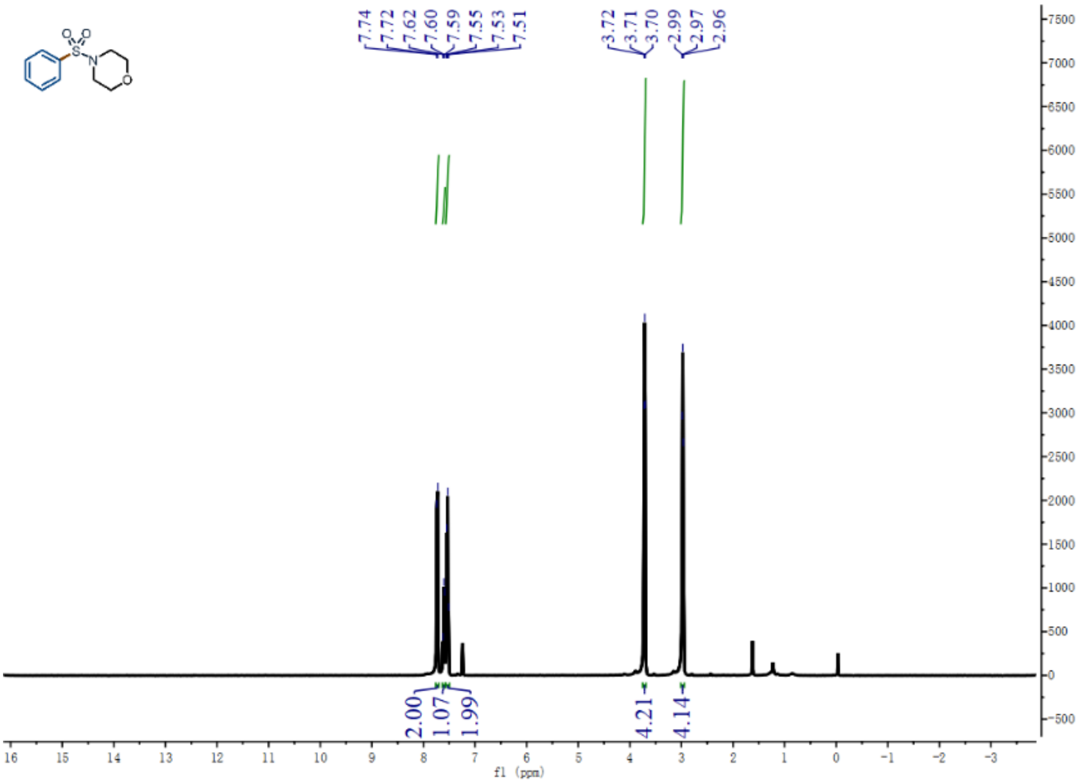

^{13}C NMR (100 MHz, $CDCl_3$) spectrum of compound **5n**

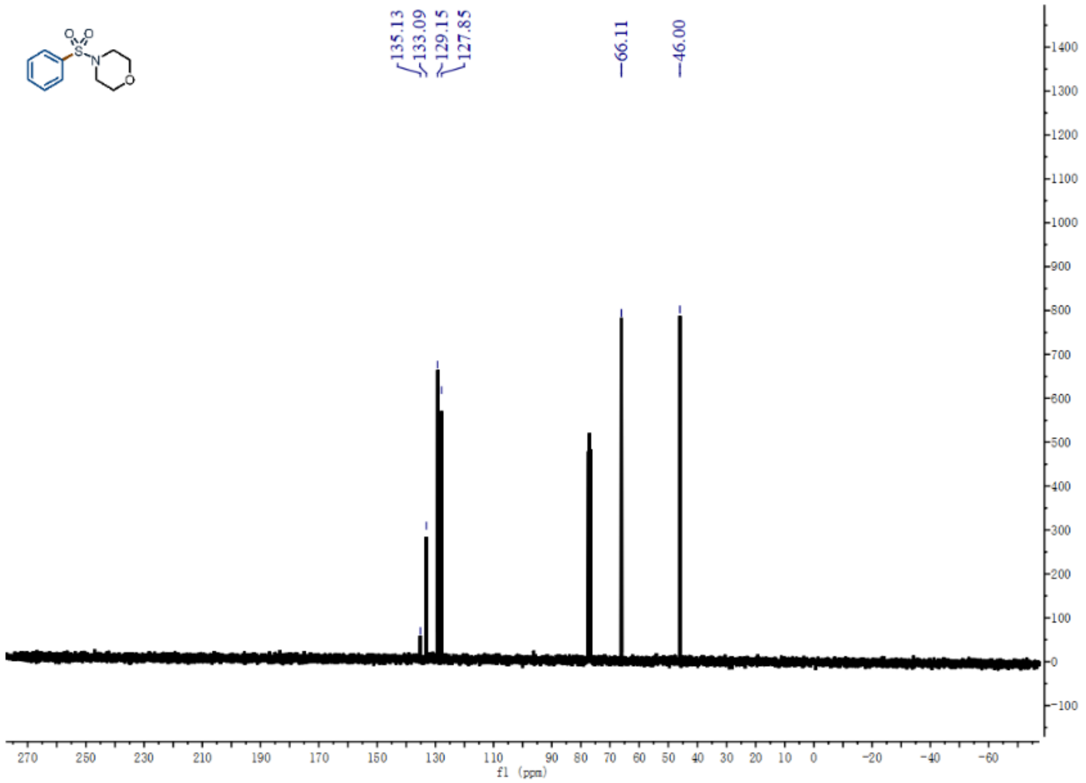

^{1}H NMR (400 MHz, $CDCl_3$) spectrum of compound **5o**

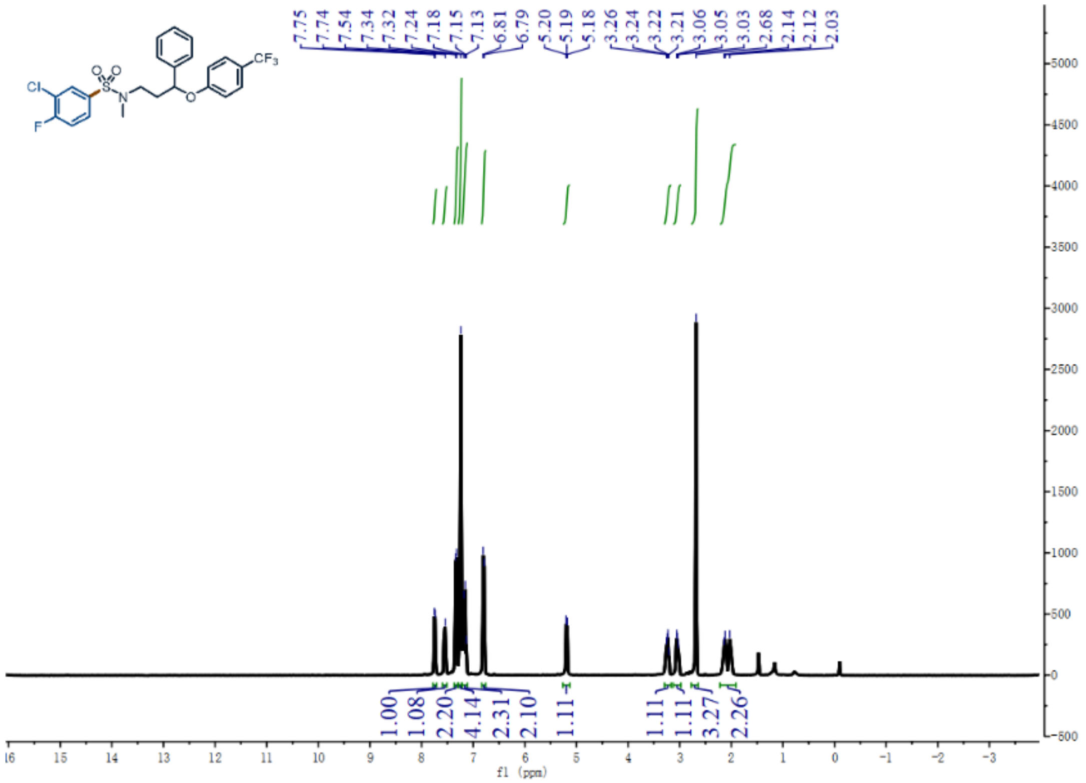

^{13}C NMR (100 MHz, $CDCl_3$) spectrum of compound **5o**

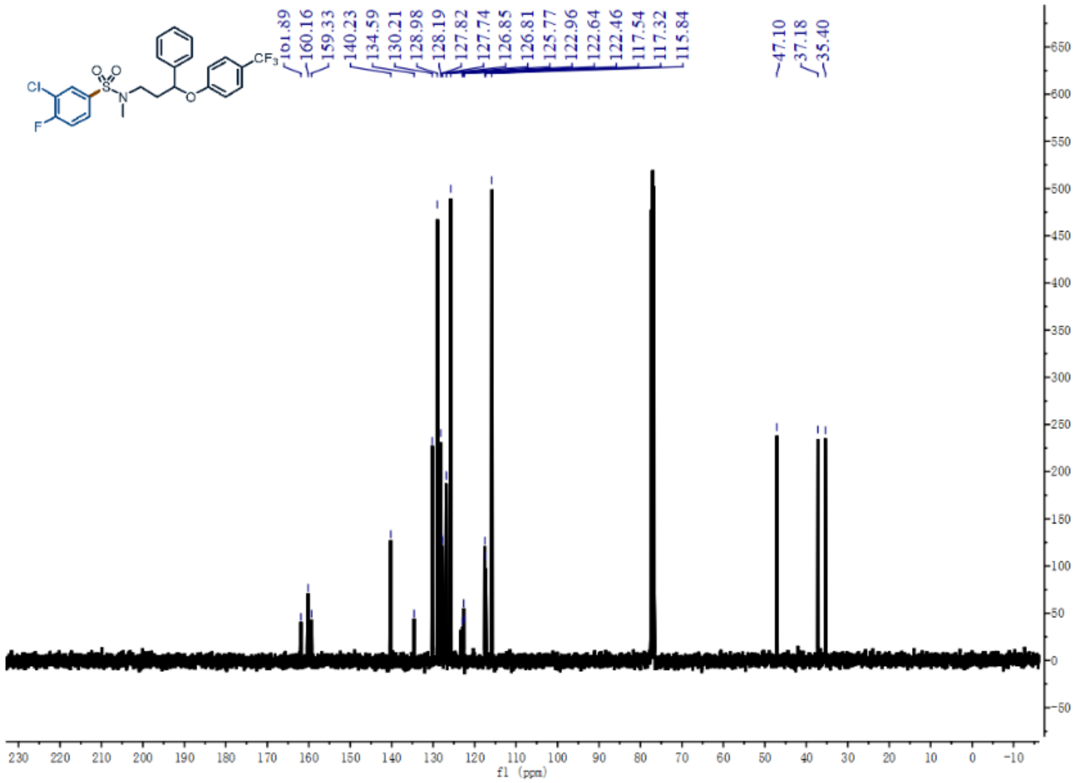

^1H NMR (400 MHz, $CDCl_3$) spectrum of compound **5p**

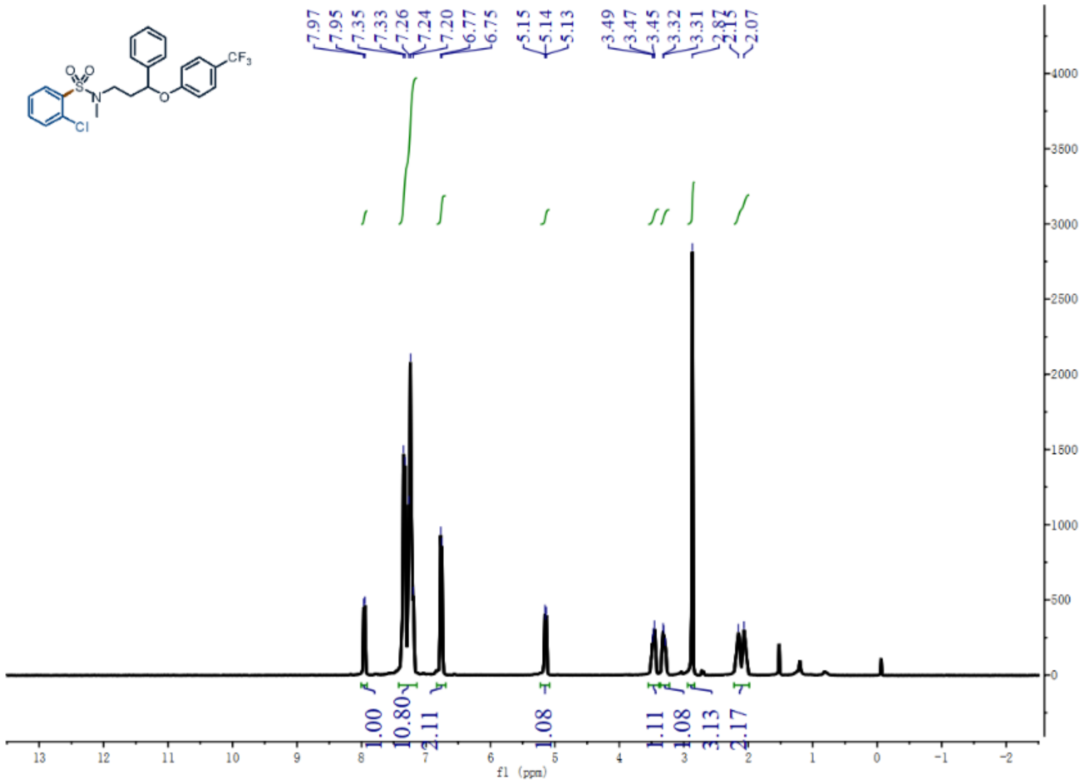

^{13}C NMR (100 MHz, $CDCl_3$) spectrum of compound **5p**

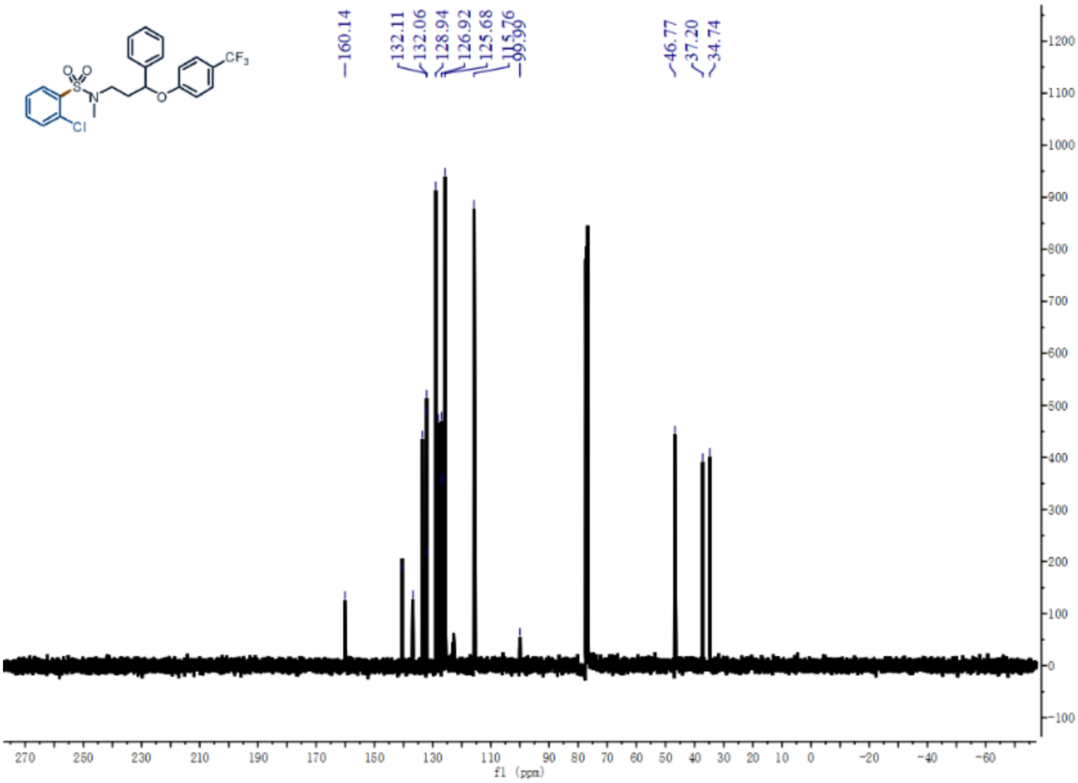

^{1}H NMR (400 MHz, $CDCl_3$) spectrum of compound **5q**

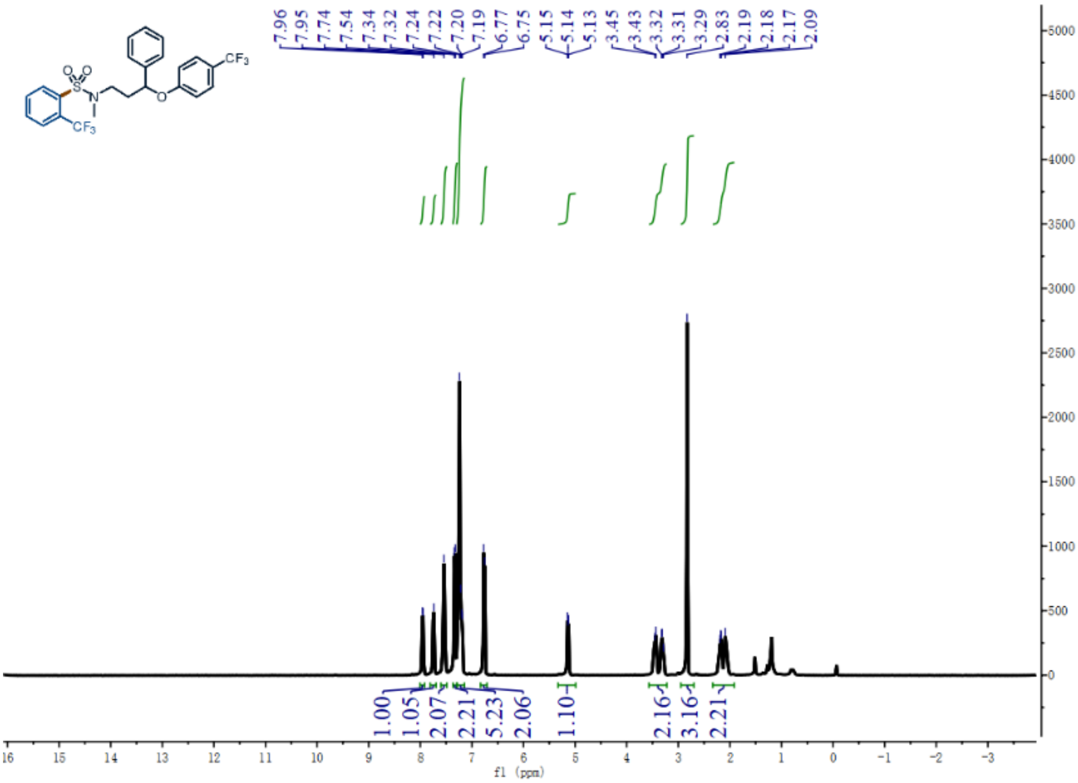

^{13}C NMR (100 MHz, $CDCl_3$) spectrum of compound **5q**

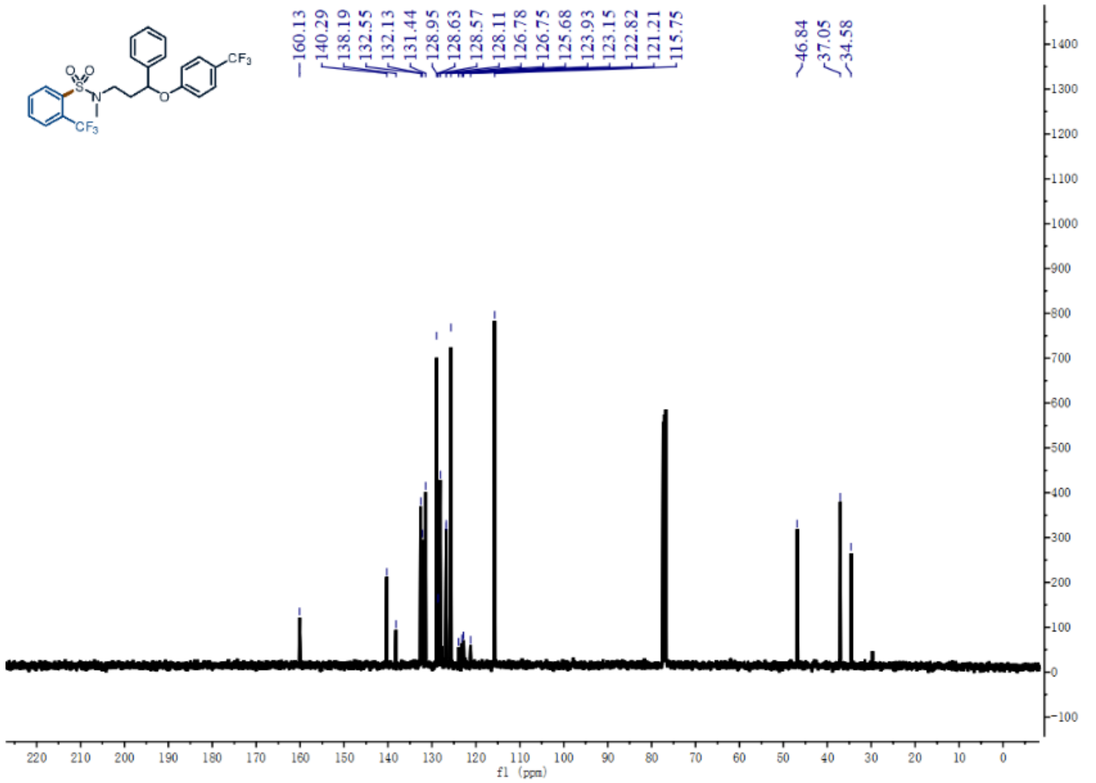

^{1}H NMR (400 MHz, $CDCl_3$) spectrum of compound **5r**

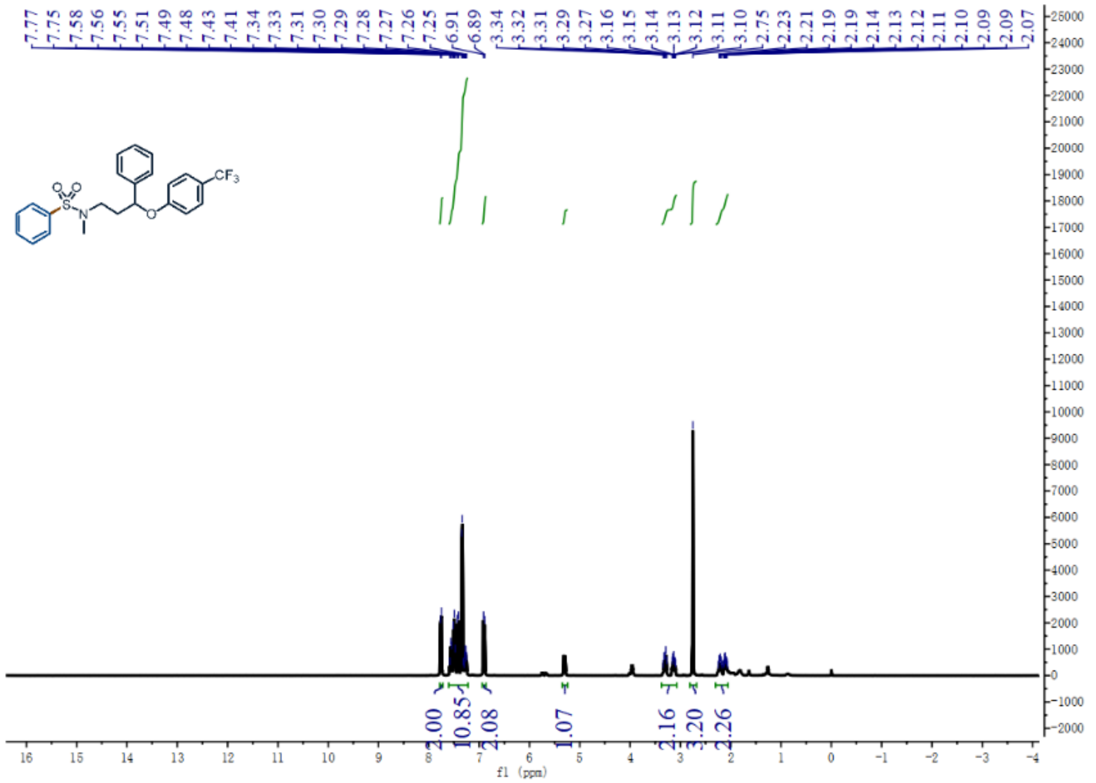

^{13}C NMR (100 MHz, $CDCl_3$) spectrum of compound **5r**

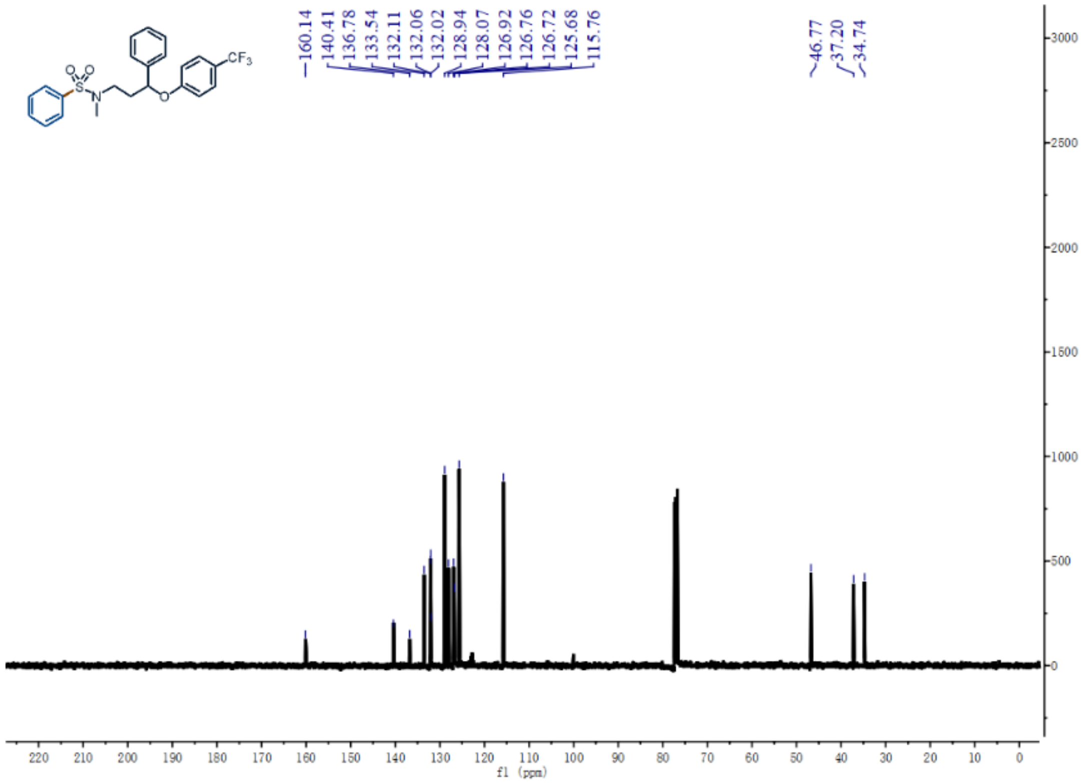

^{1}H NMR (400 MHz, $CDCl_3$) spectrum of compound **7a**

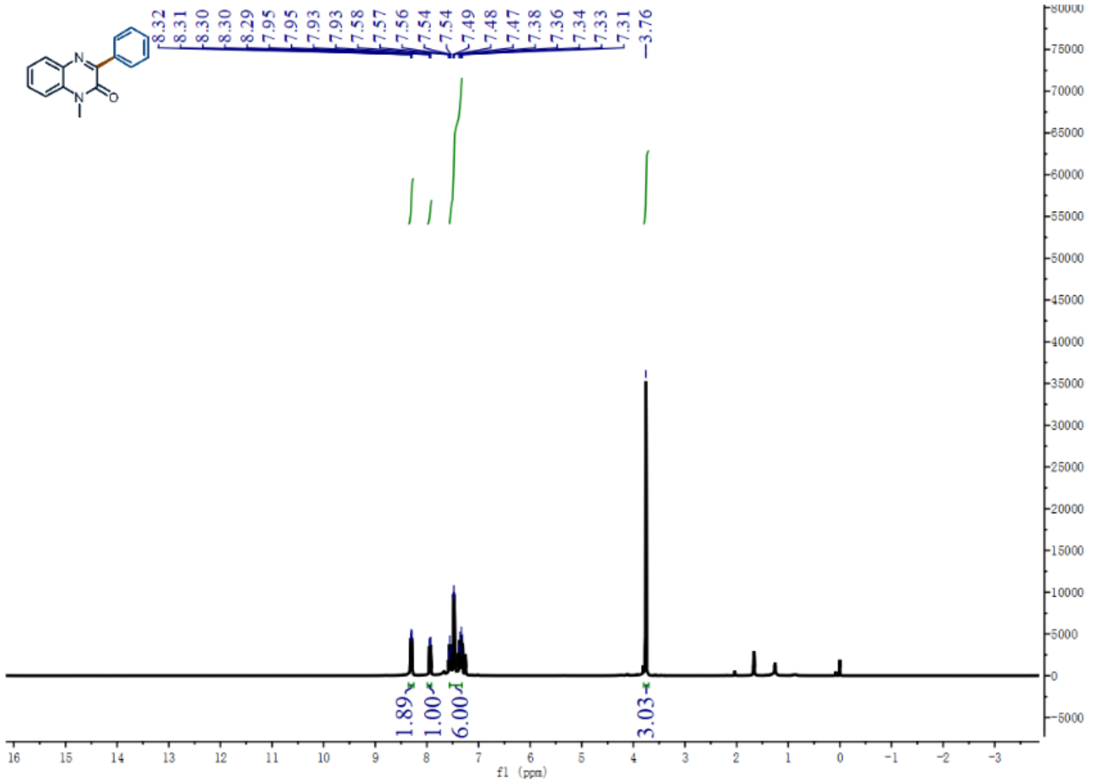

^{13}C NMR (100 MHz, $CDCl_3$) spectrum of compound **7a**

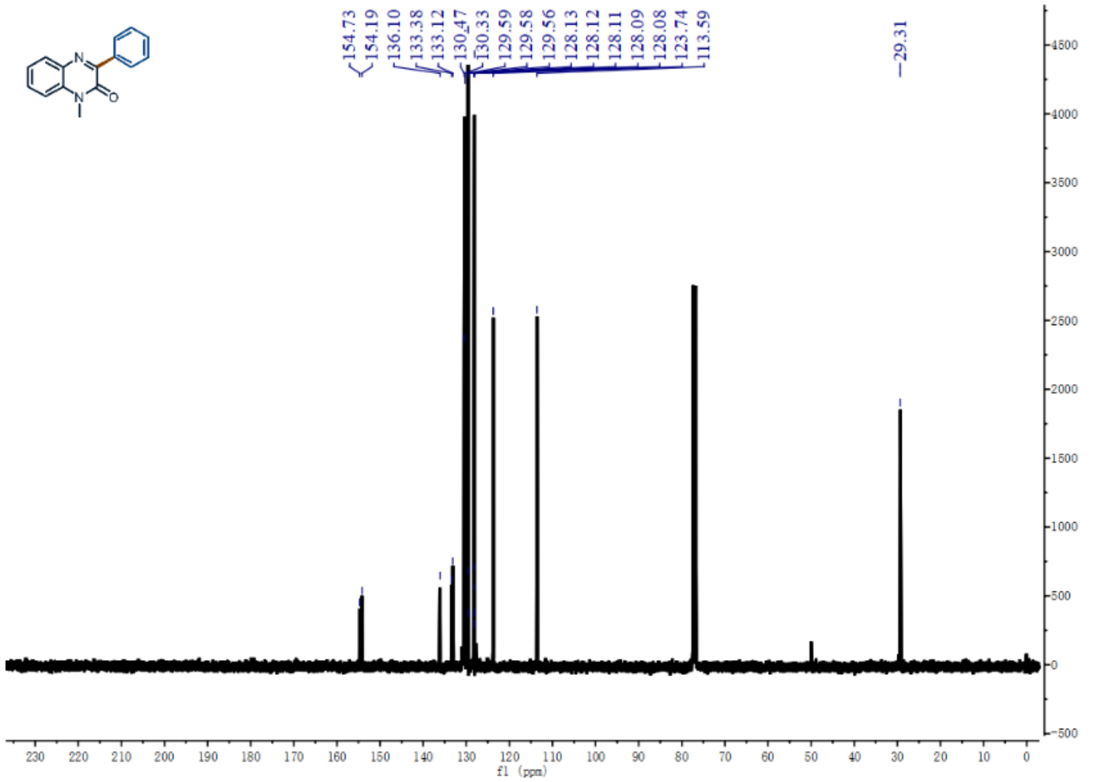

^{1}H NMR (400 MHz, $CDCl_3$) spectrum of compound **7b**

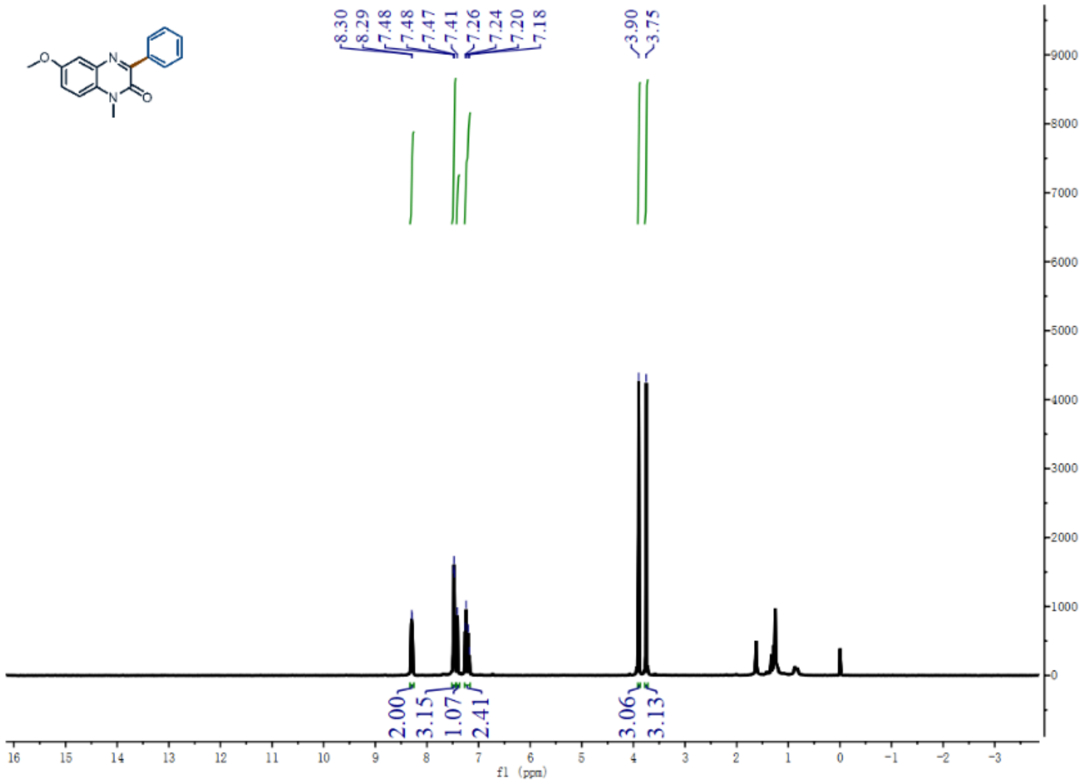

^{13}C NMR (100 MHz, $CDCl_3$) spectrum of compound **7b**

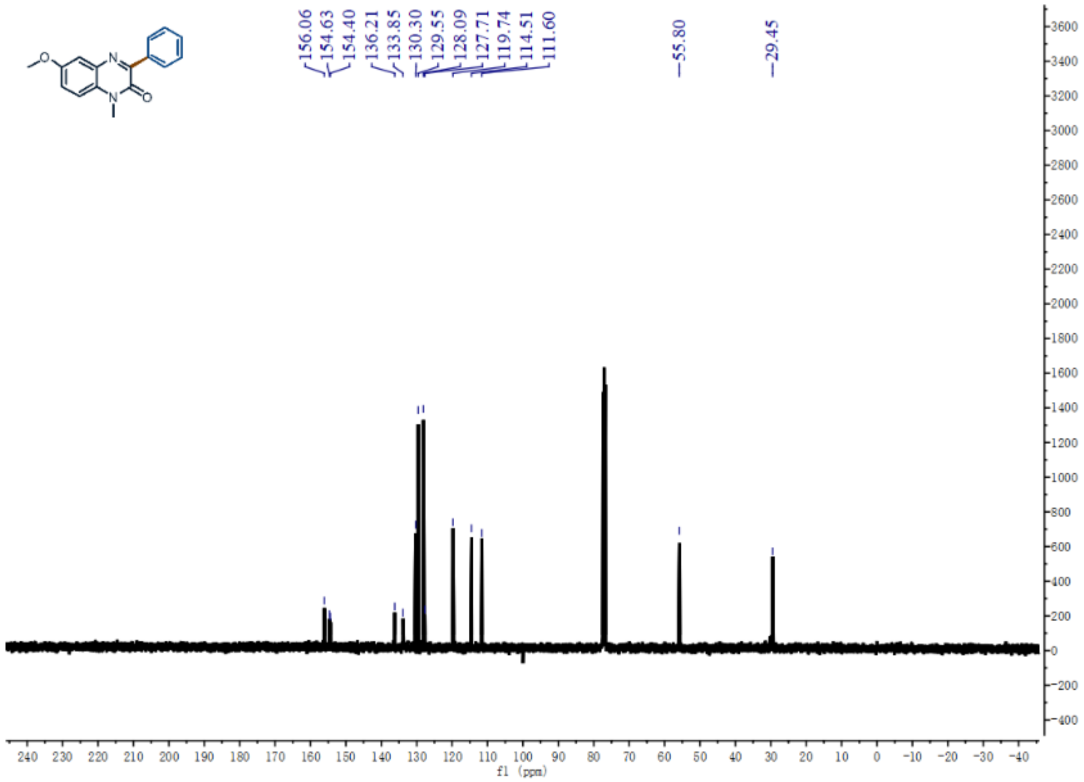

^{1}H NMR (400 MHz, $CDCl_3$) spectrum of compound **7c**

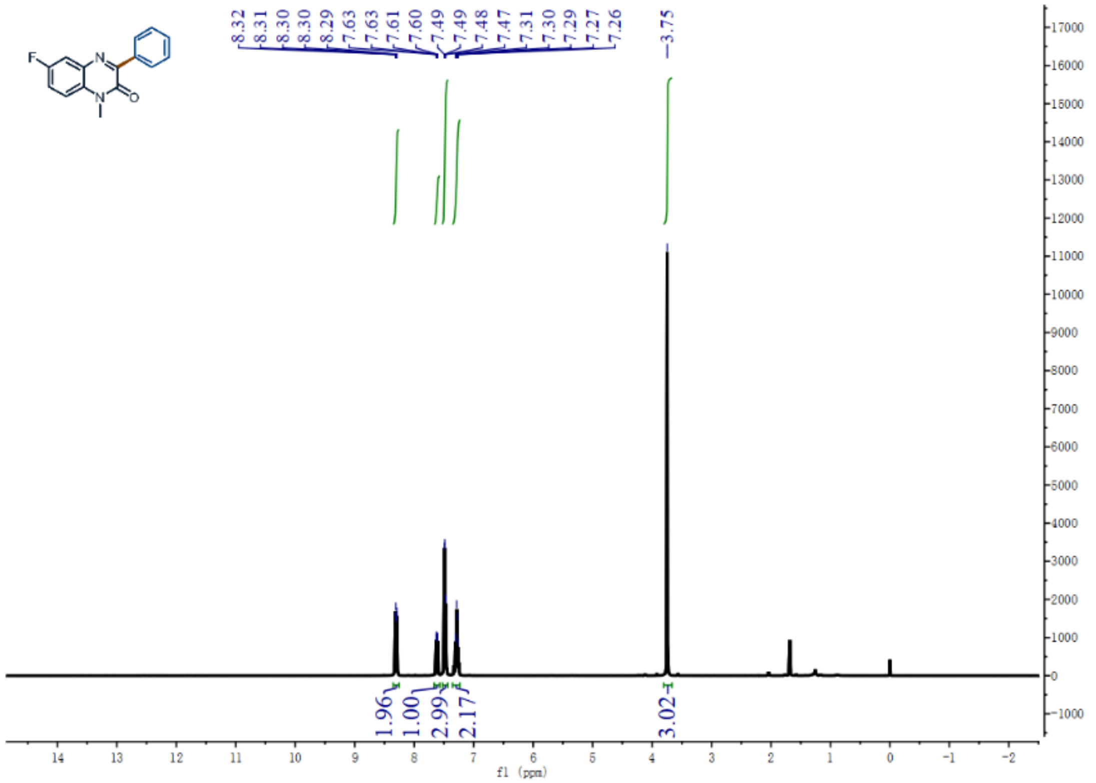

^{13}C NMR (100 MHz, $CDCl_3$) spectrum of compound **7c**

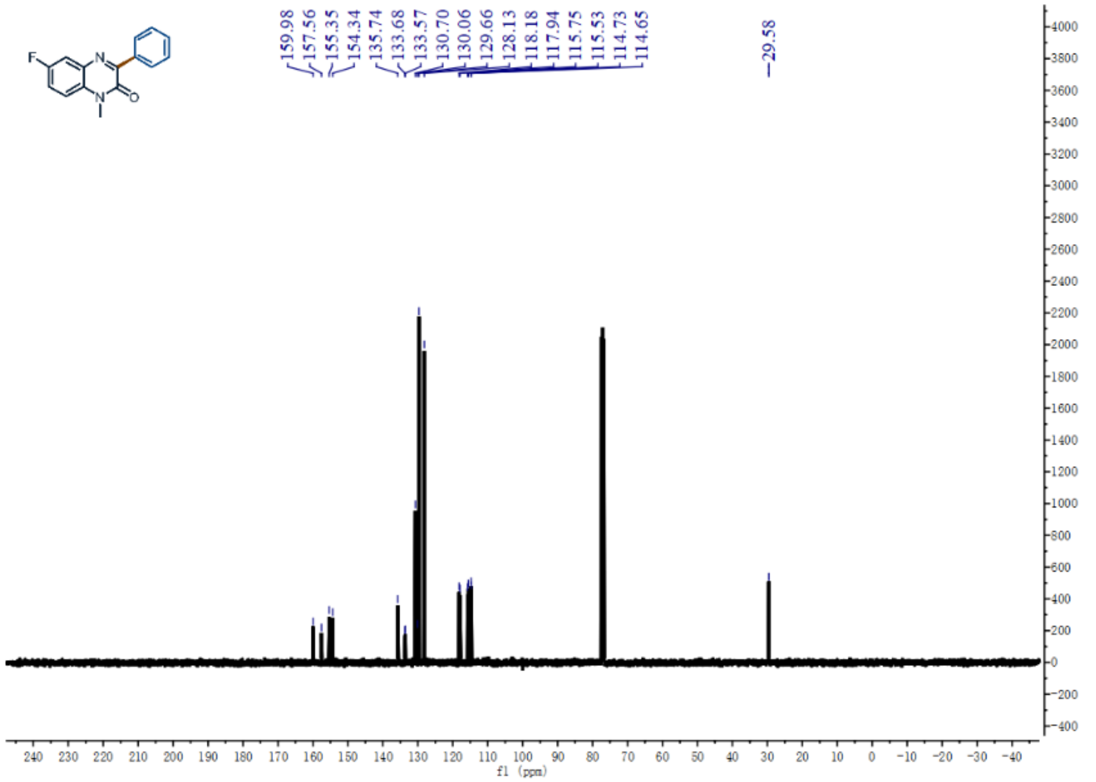

^{1}H NMR (400 MHz, $CDCl_3$) spectrum of compound **7d**

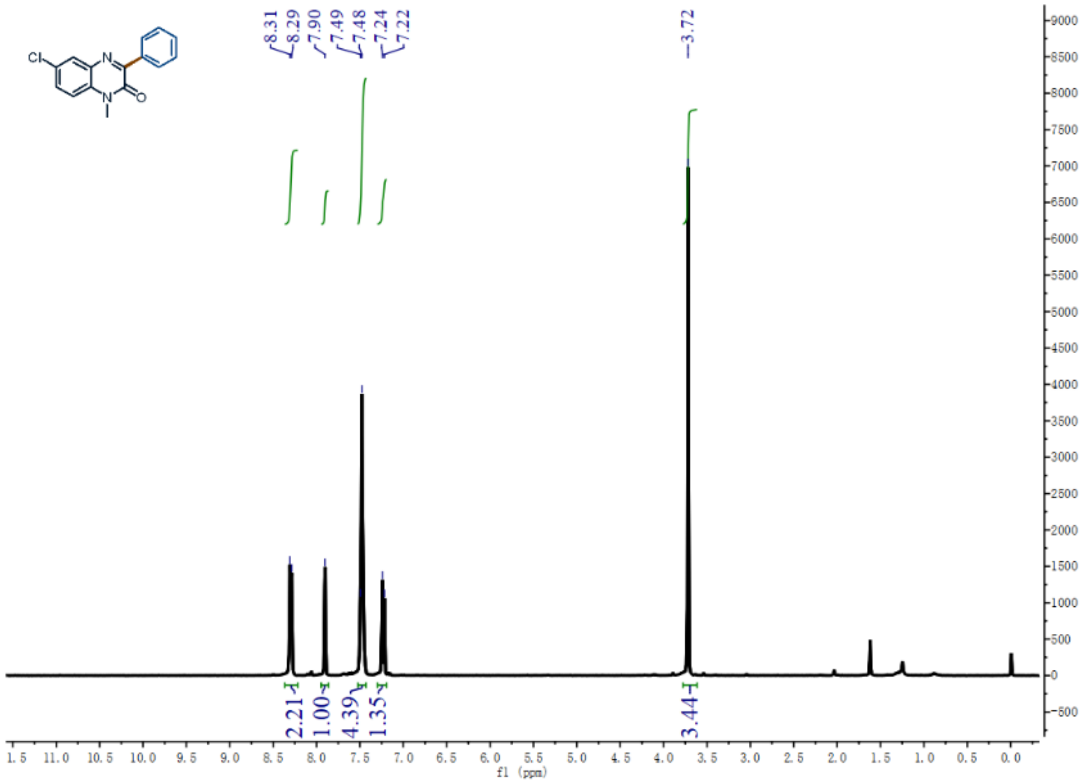

^{13}C NMR (100 MHz, $CDCl_3$) spectrum of compound **7d**

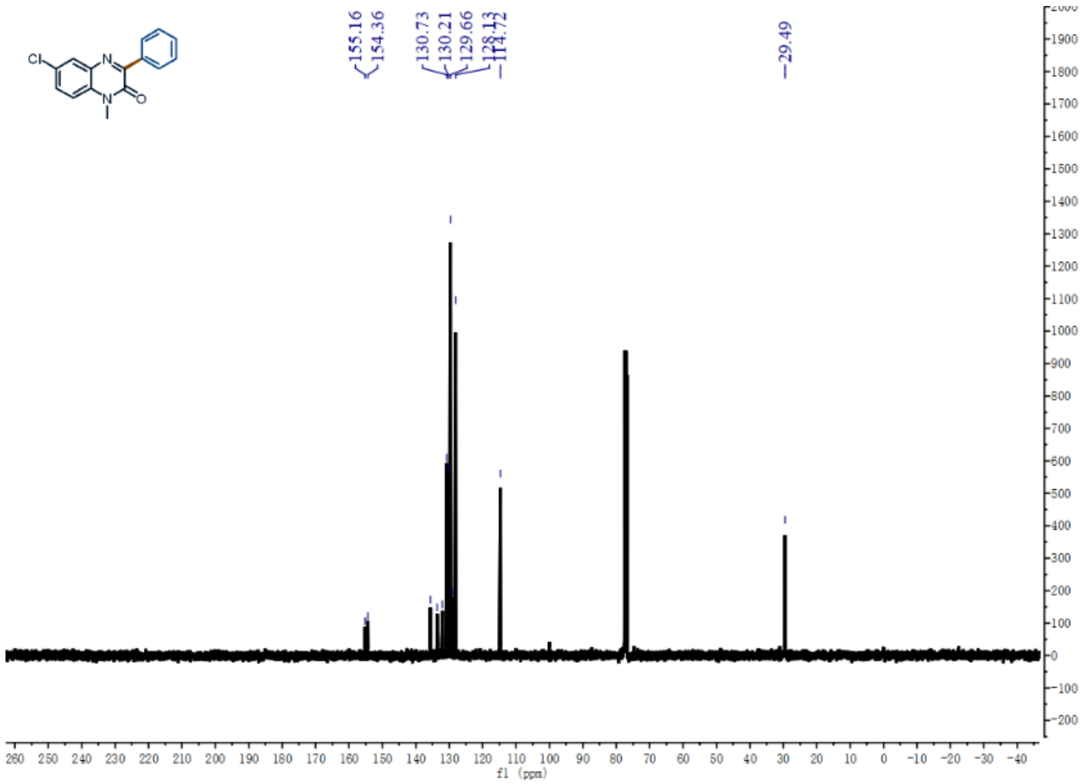

^{1}H NMR (400 MHz, $CDCl_3$) spectrum of compound **7e**

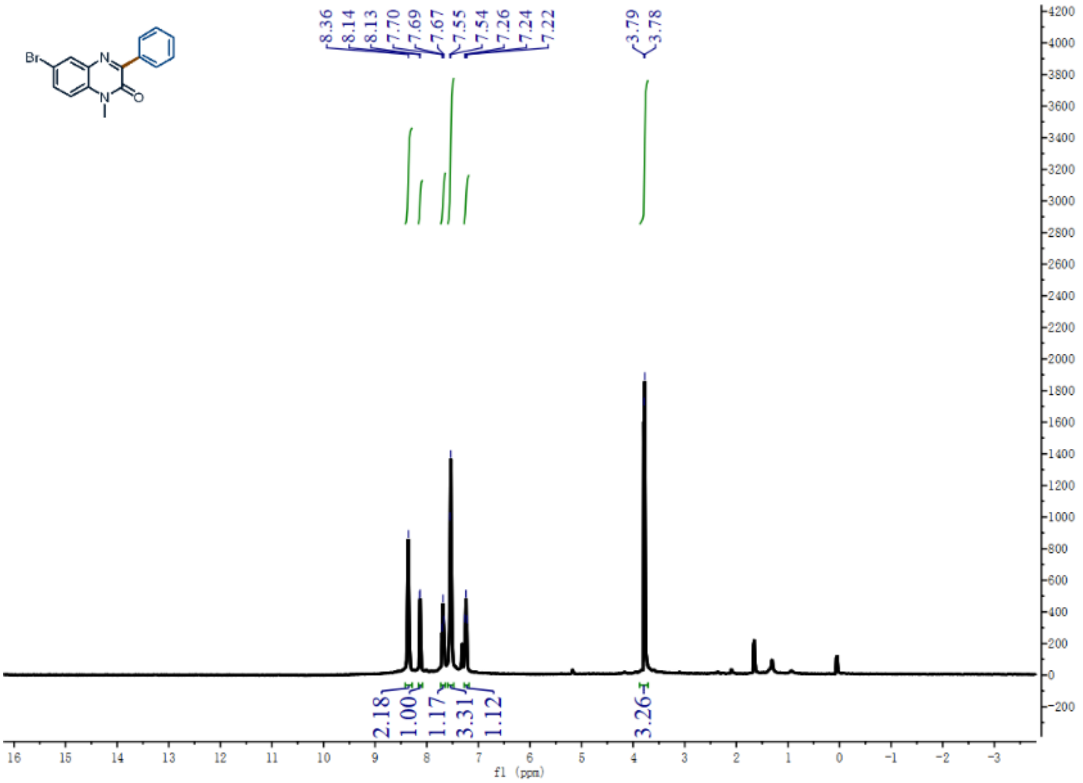

^{13}C NMR (100 MHz, $CDCl_3$) spectrum of compound **7e**

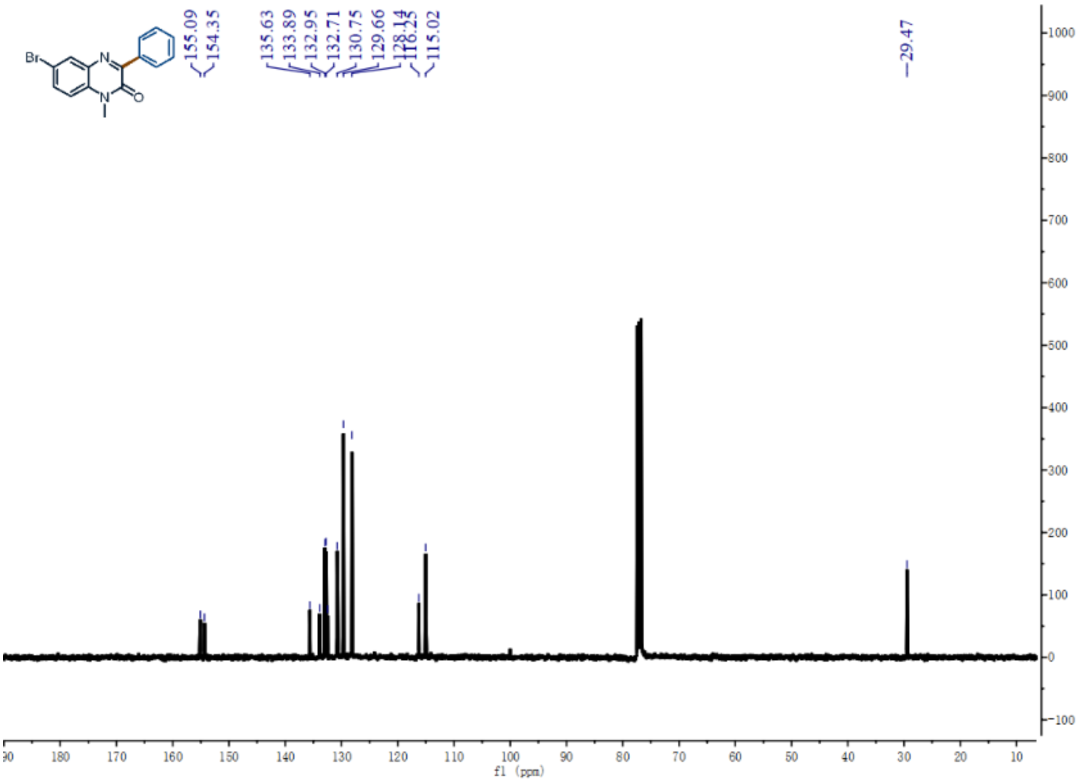

^{1}H NMR (400 MHz, $CDCl_3$) spectrum of compound **7f**

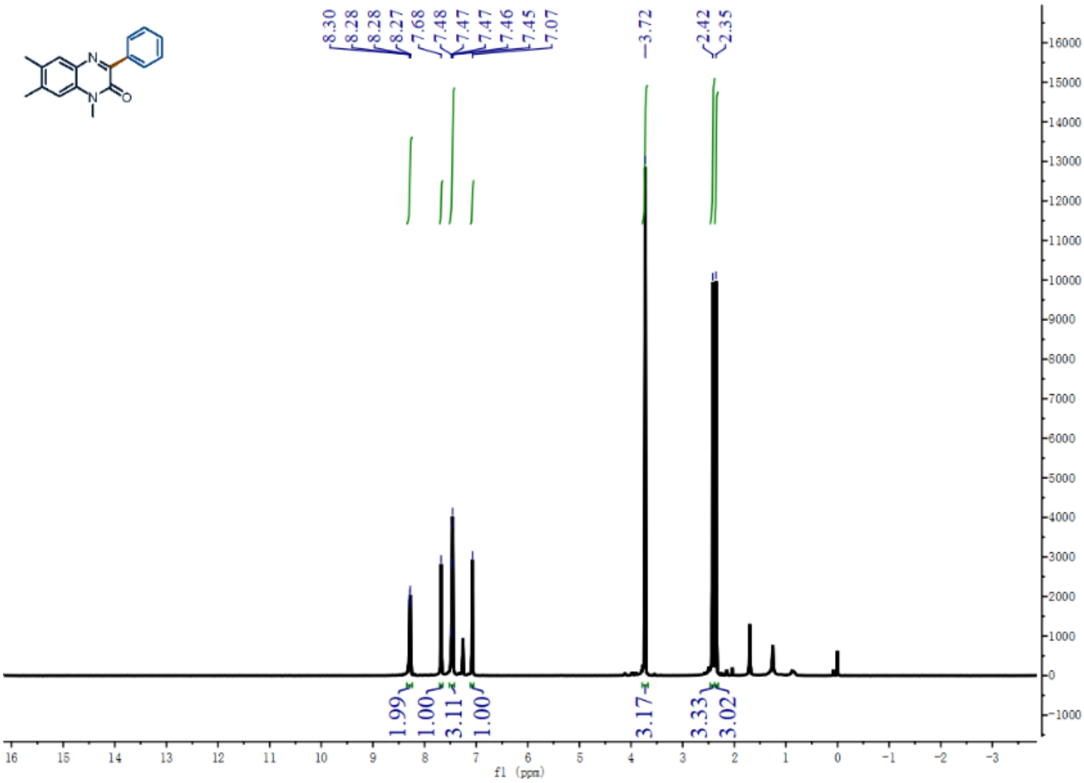

^{13}C NMR (100 MHz, $CDCl_3$) spectrum of compound **7f**

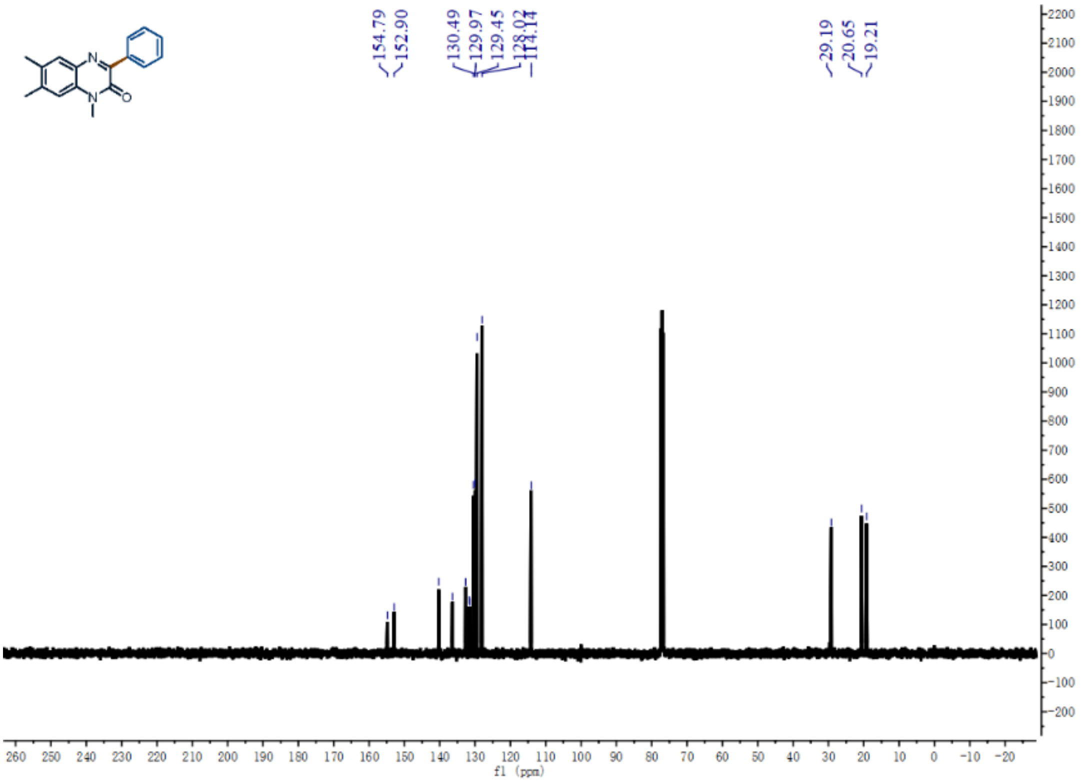

^{1}H NMR (400 MHz, $CDCl_3$) spectrum of compound **7g**

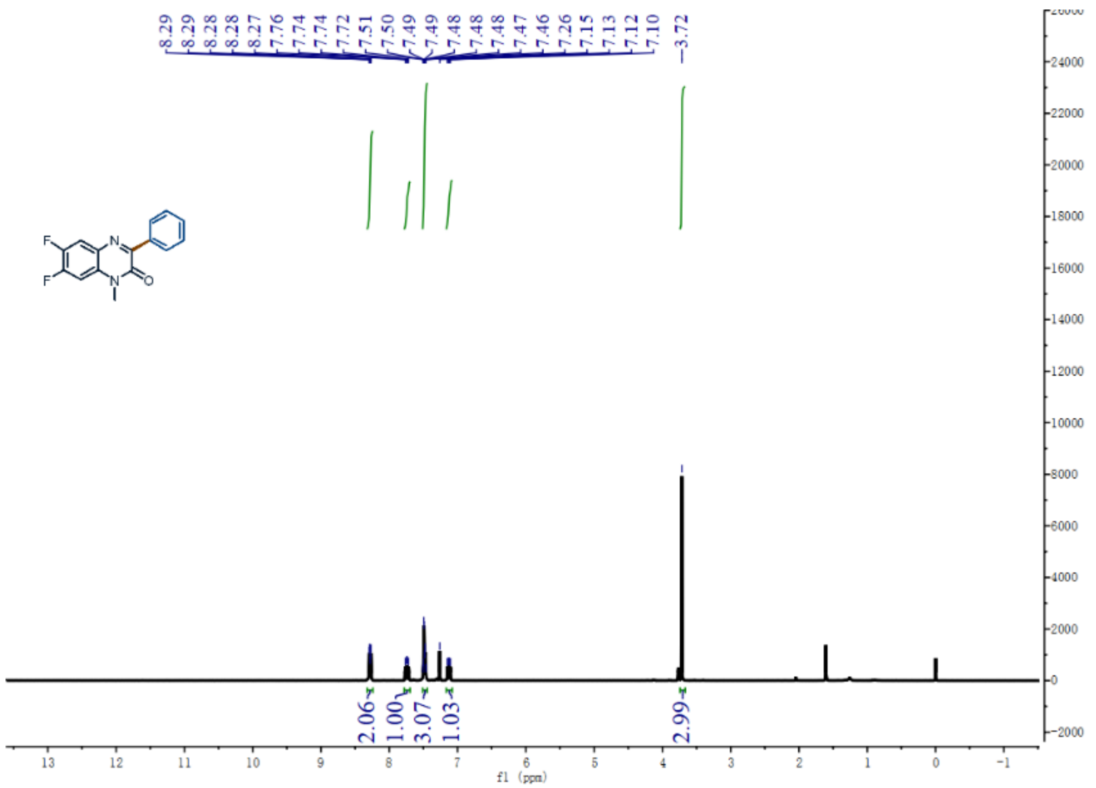

^{13}C NMR (100 MHz, $CDCl_3$) spectrum of compound **7g**

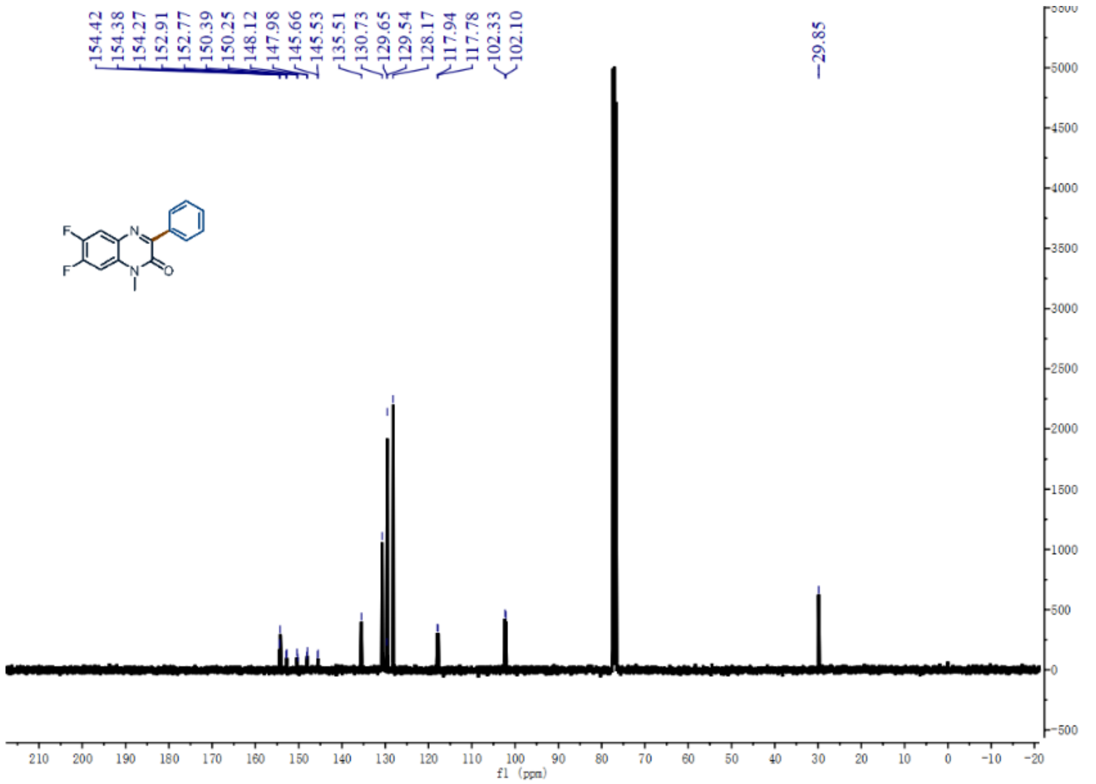

^{1}H NMR (400 MHz, $CDCl_3$) spectrum of compound **7h**

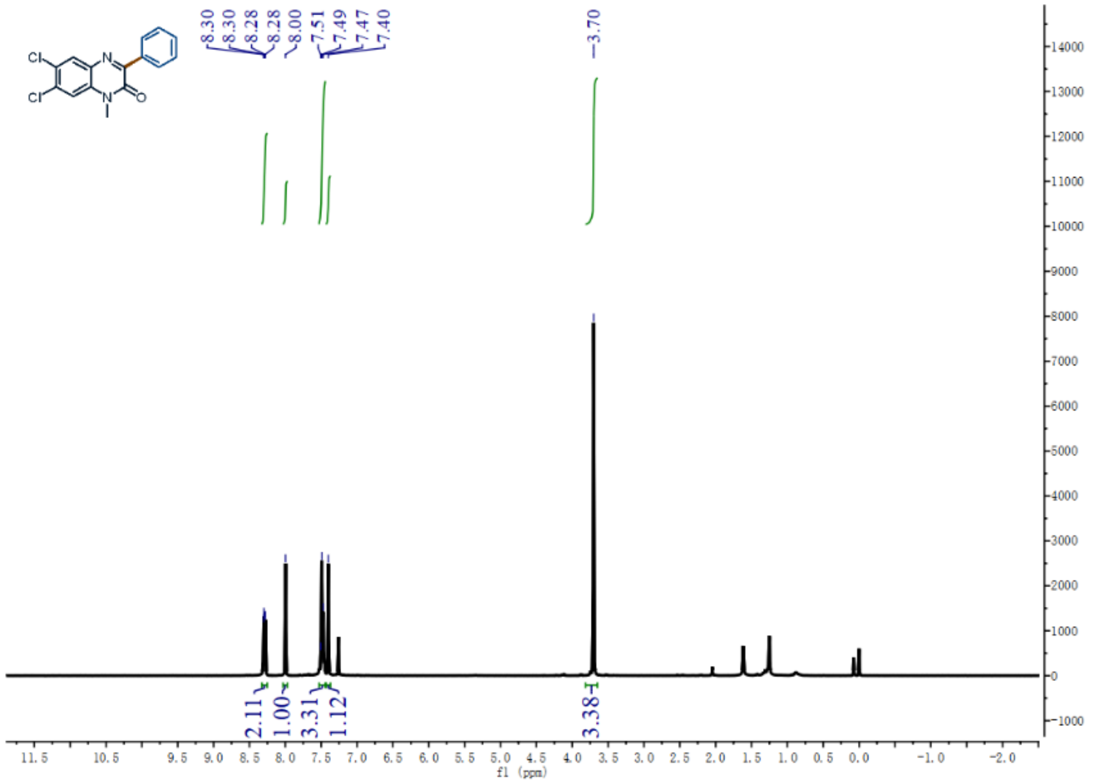

^{13}C NMR (100 MHz, $CDCl_3$) spectrum of compound **7h**

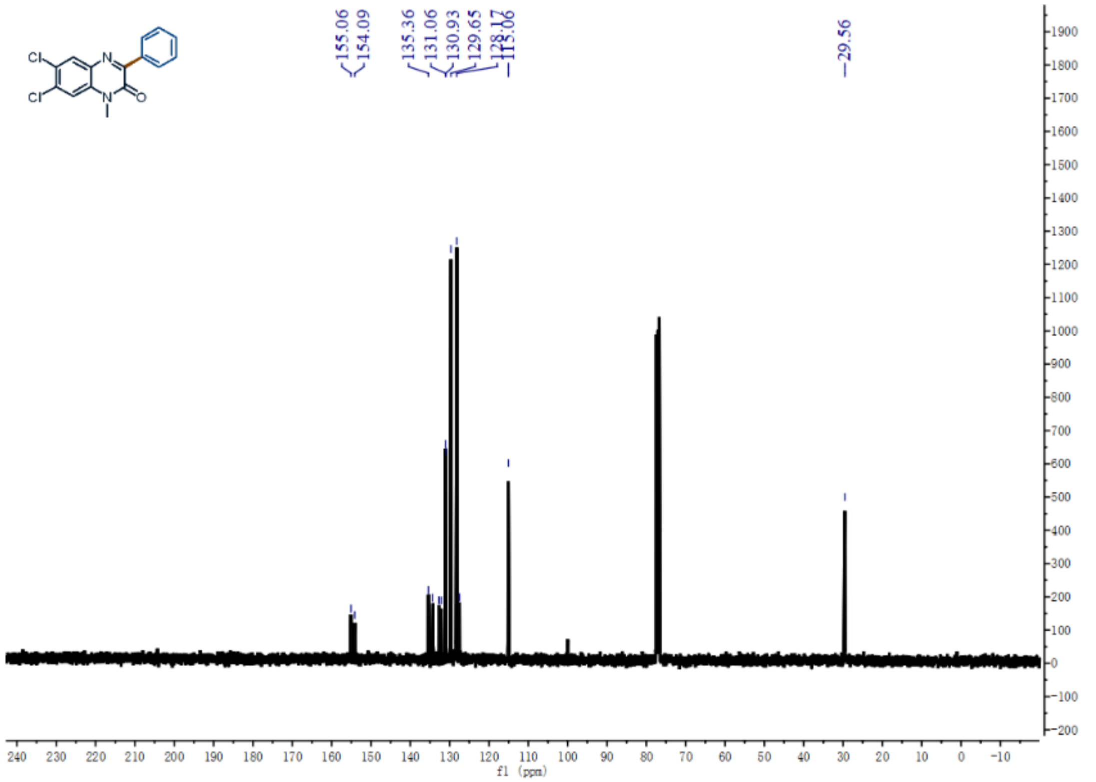

^{1}H NMR (400 MHz, $CDCl_3$) spectrum of compound **7i**

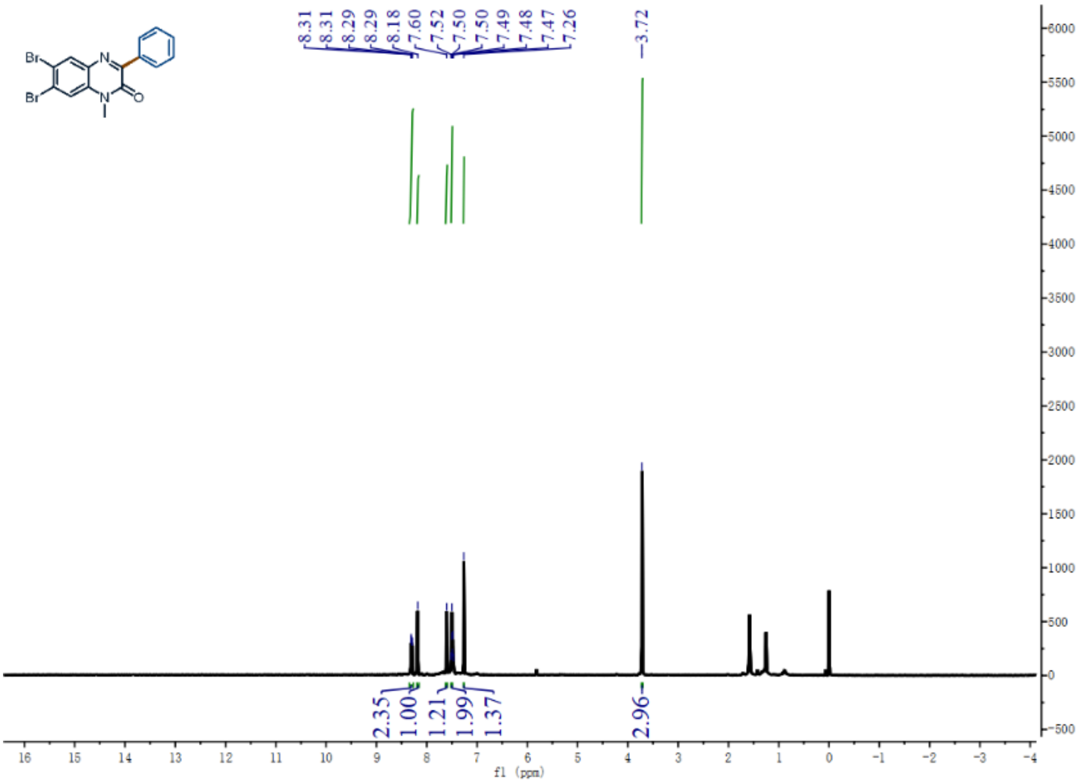

^{13}C NMR (100 MHz, $CDCl_3$) spectrum of compound **7i**

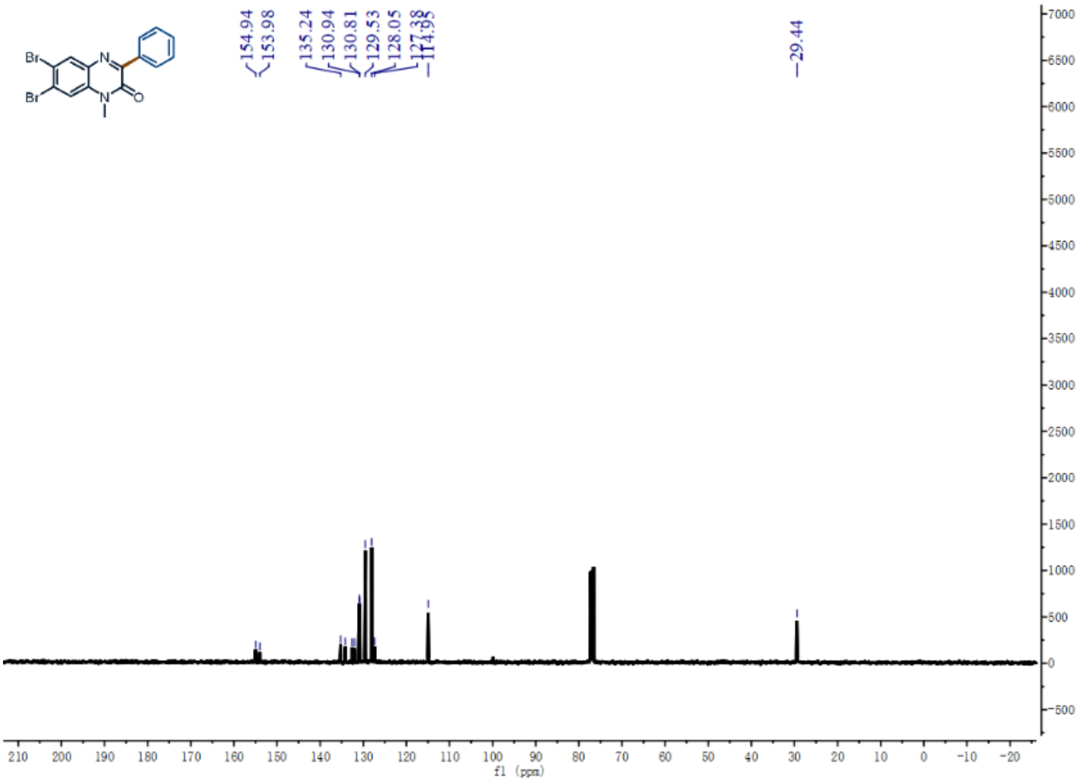

^{1}H NMR (400 MHz, $CDCl_3$) spectrum of compound **7j**

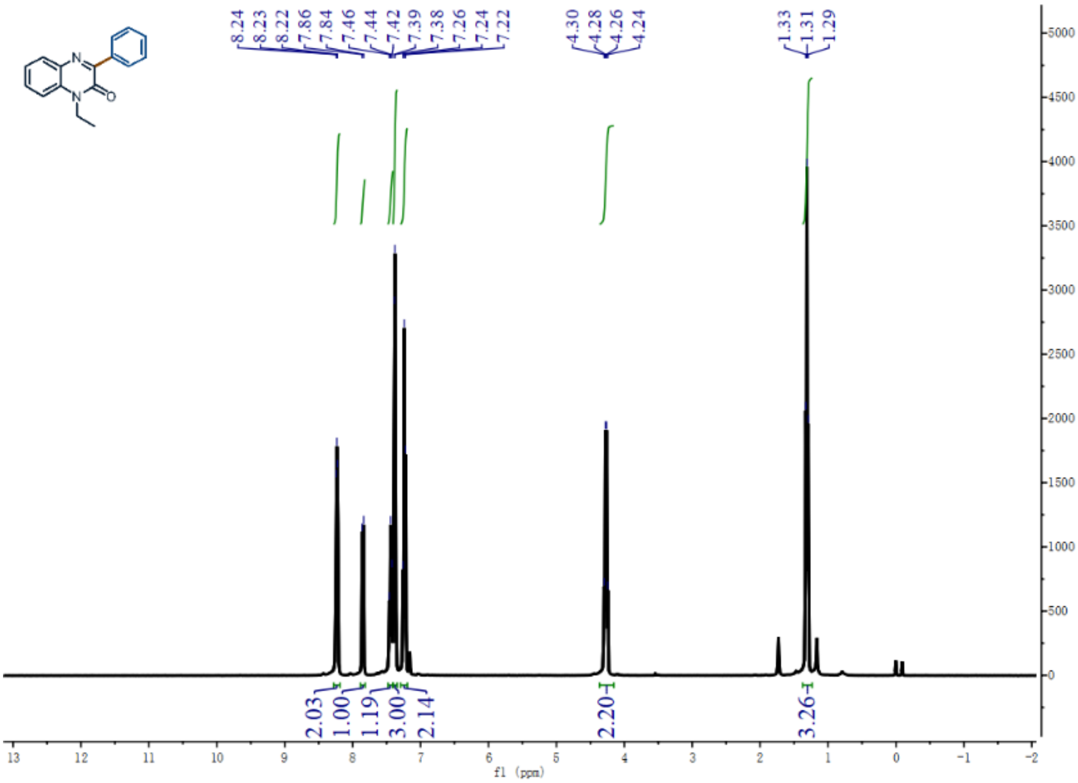

^{13}C NMR (100 MHz, $CDCl_3$) spectrum of compound **7j**

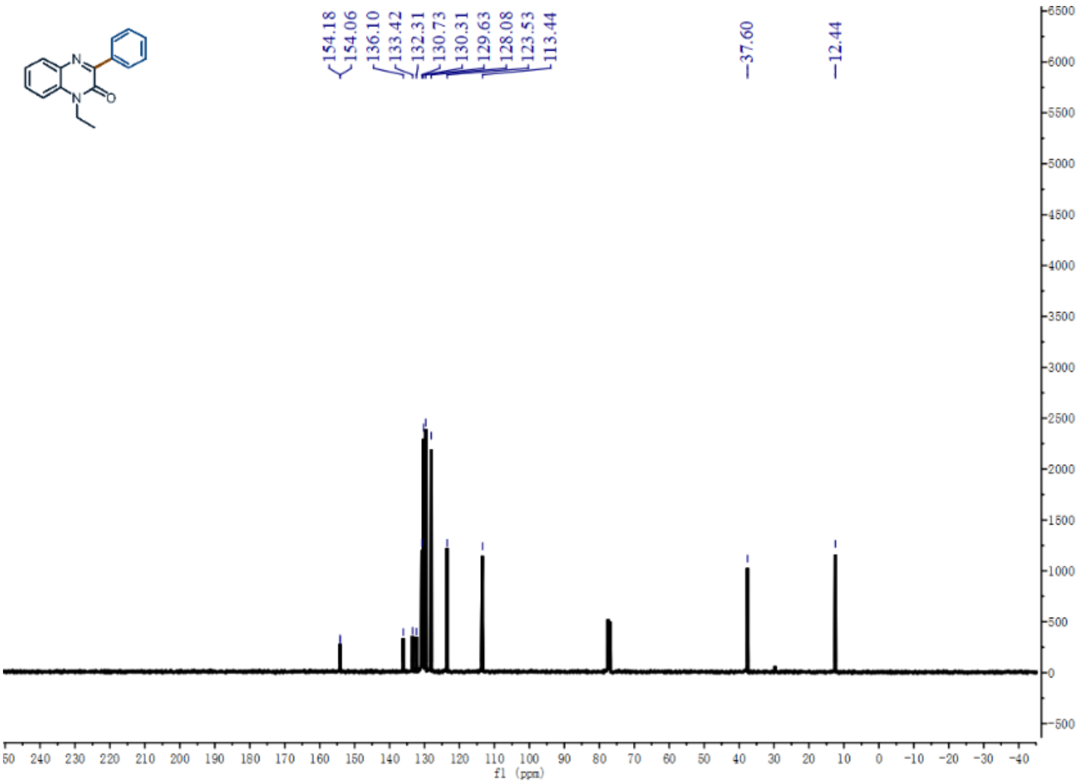

^{1}H NMR (400 MHz, $CDCl_3$) spectrum of compound **7k**

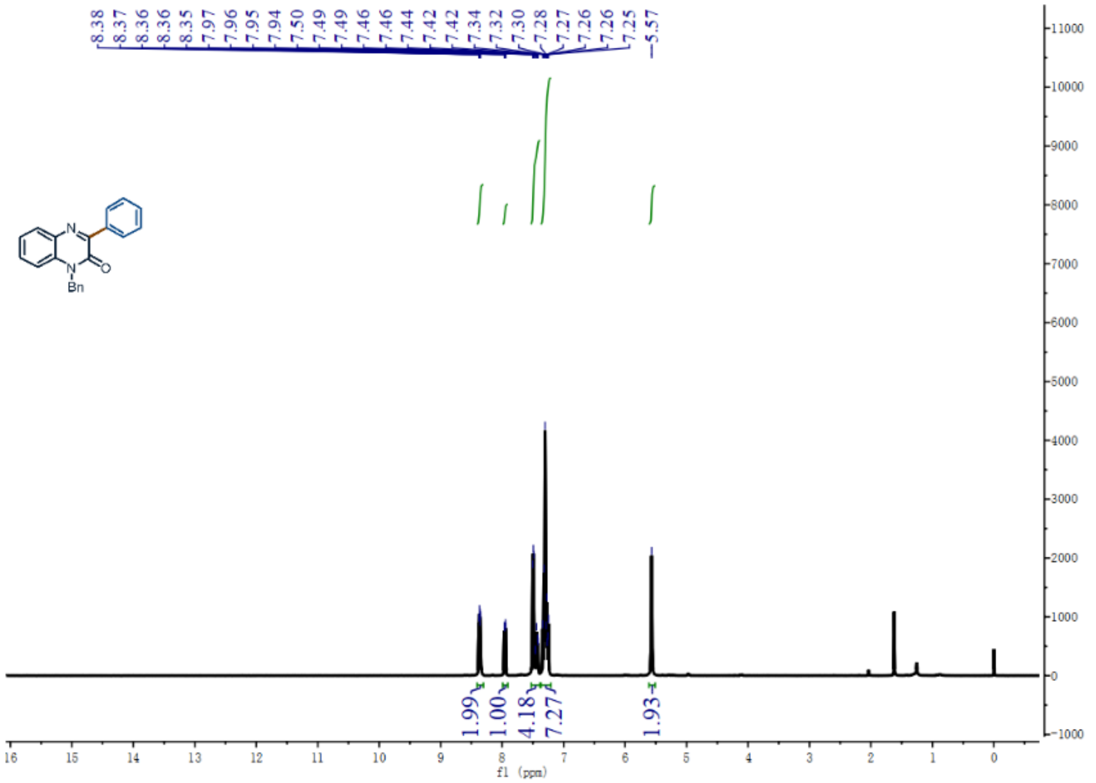

^{13}C NMR (100 MHz, CDCl$_3$) spectrum of compound **7k**

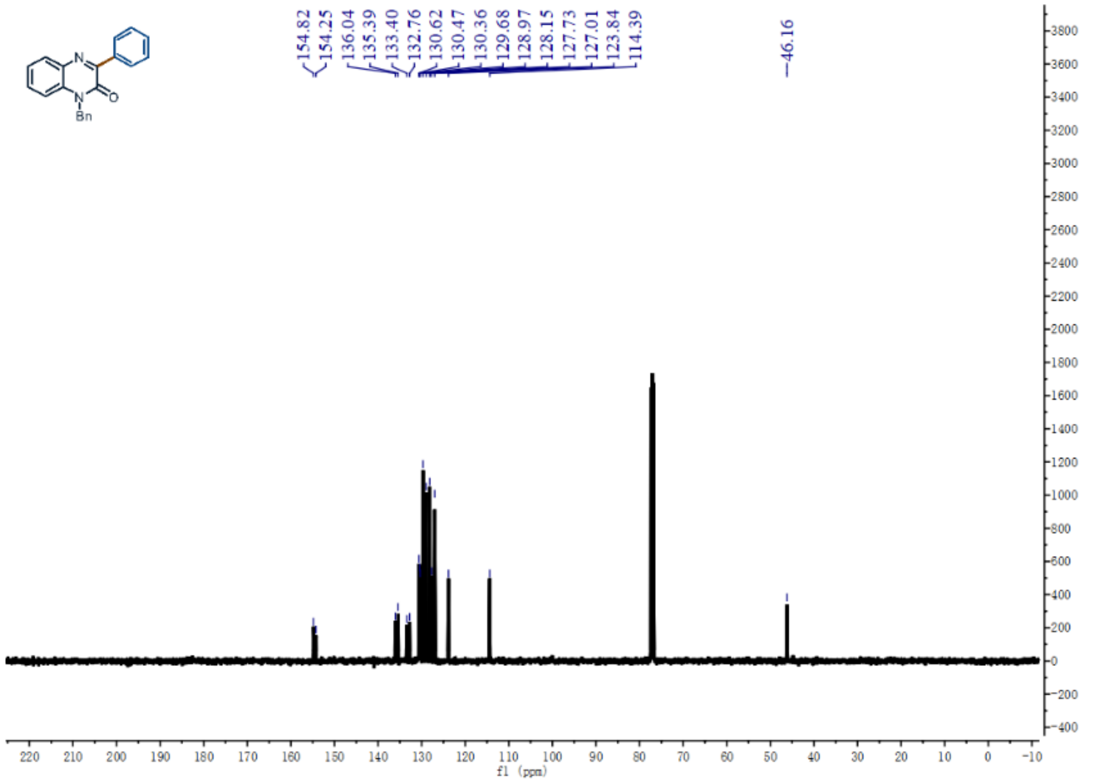

^{1}H NMR (400 MHz, CDCl$_3$) spectrum of compound **7l**

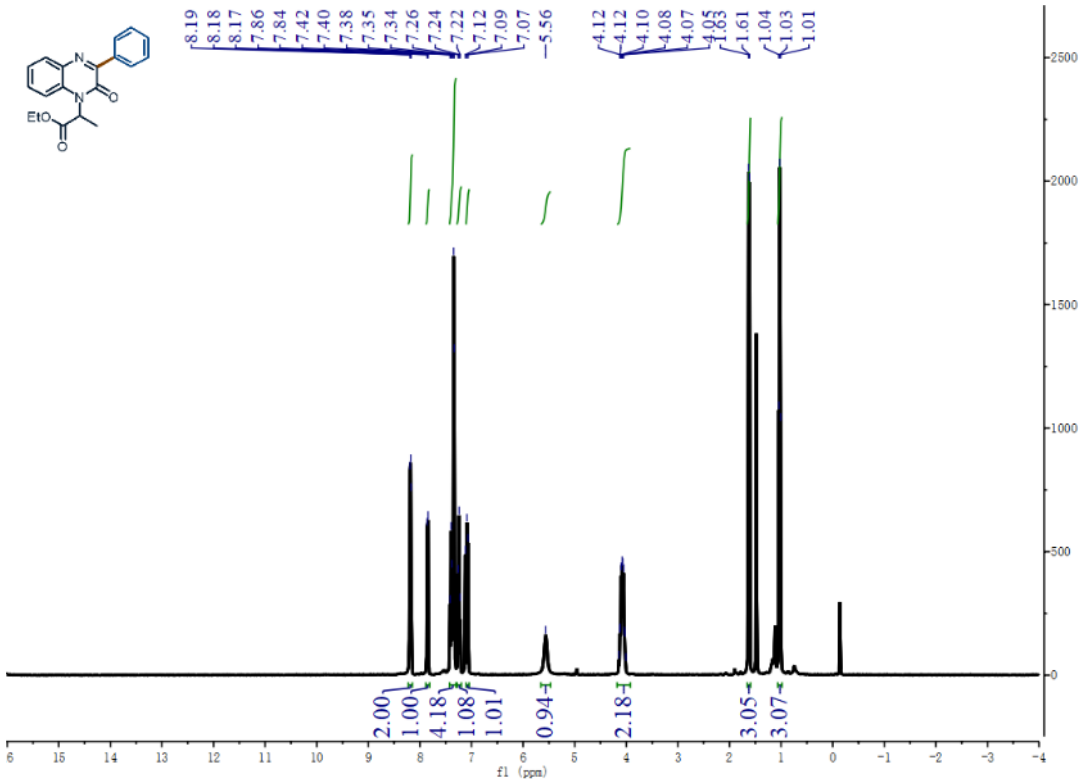

^{13}C NMR (100 MHz, $CDCl_3$) spectrum of compound **7l**

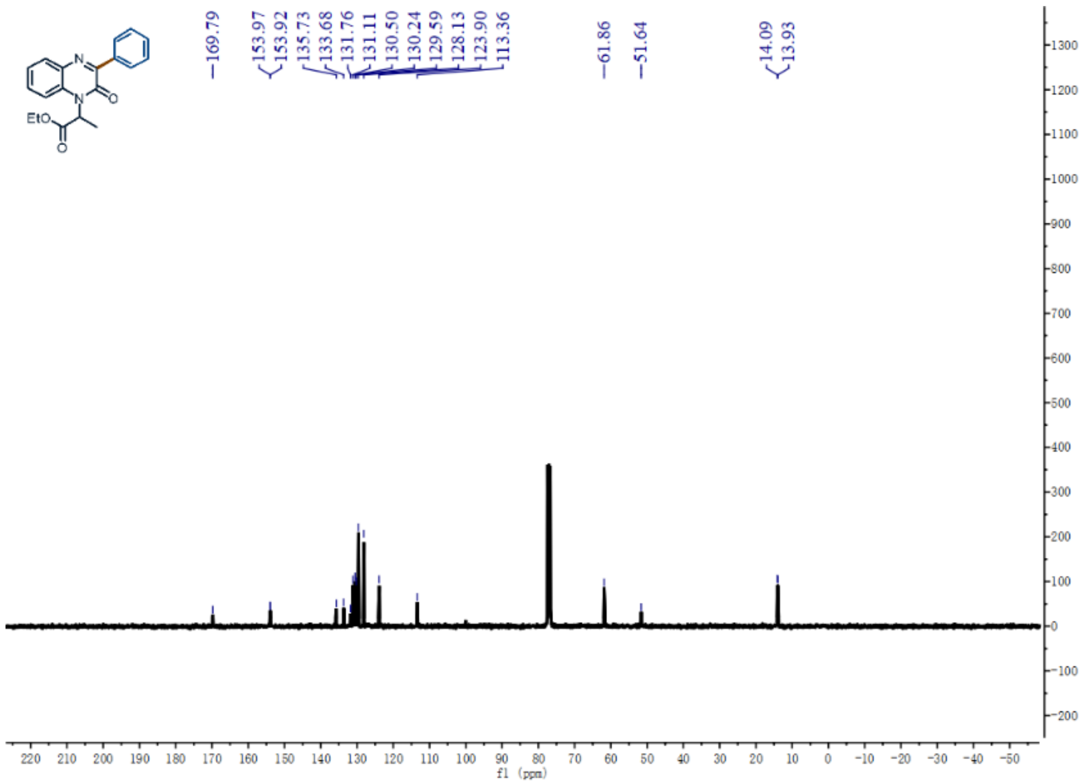

^{1}H NMR (400 MHz, $CDCl_3$) spectrum of compound **7m**

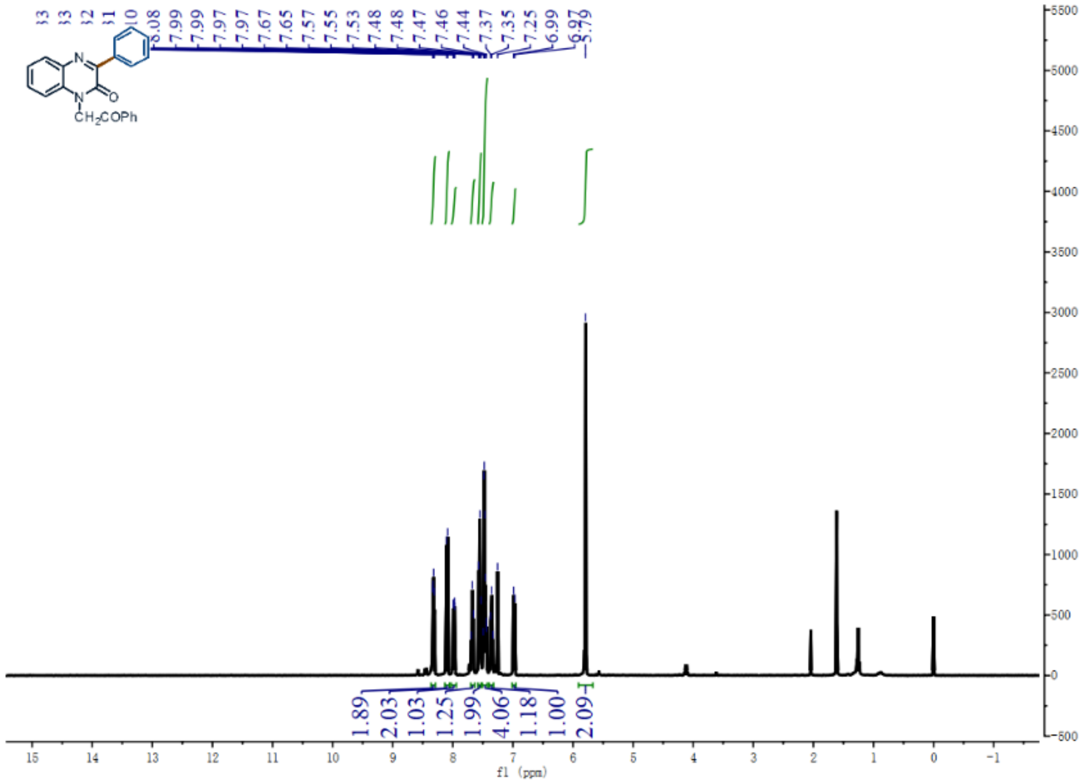

^{13}C NMR (100 MHz, $CDCl_3$) spectrum of compound **7m**

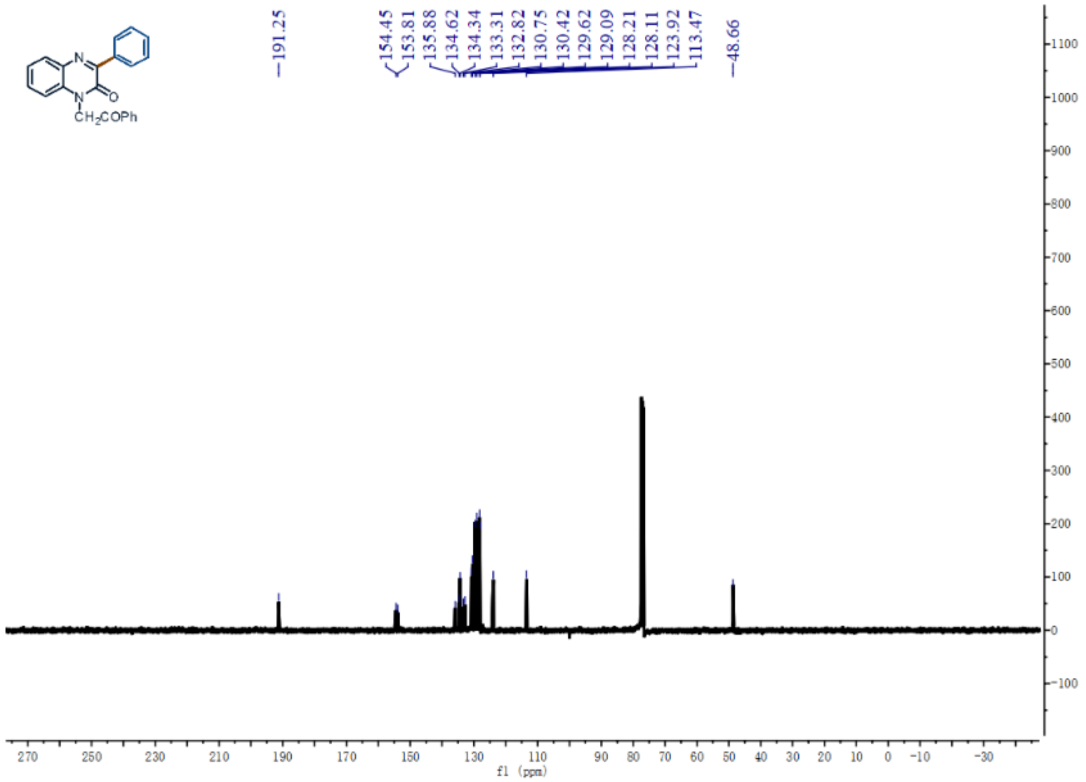

^{1}H NMR (400 MHz, $CDCl_3$) spectrum of compound **7n**

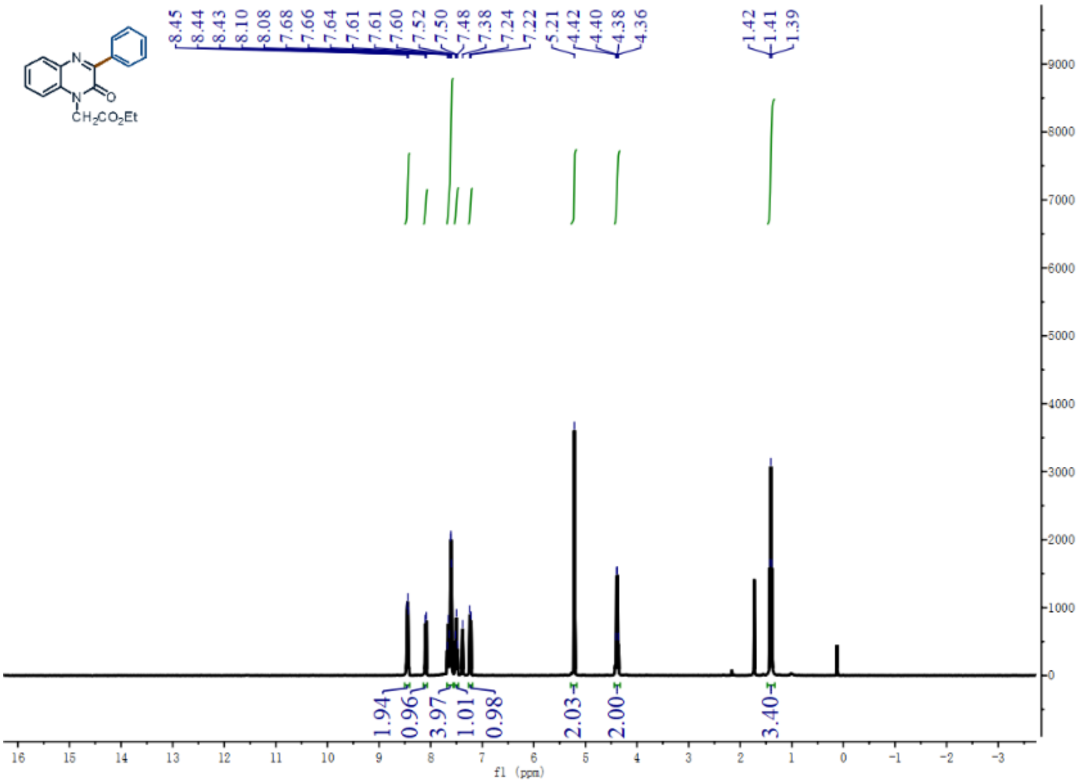

^{13}C NMR (100 MHz, $CDCl_3$) spectrum of compound **7n**

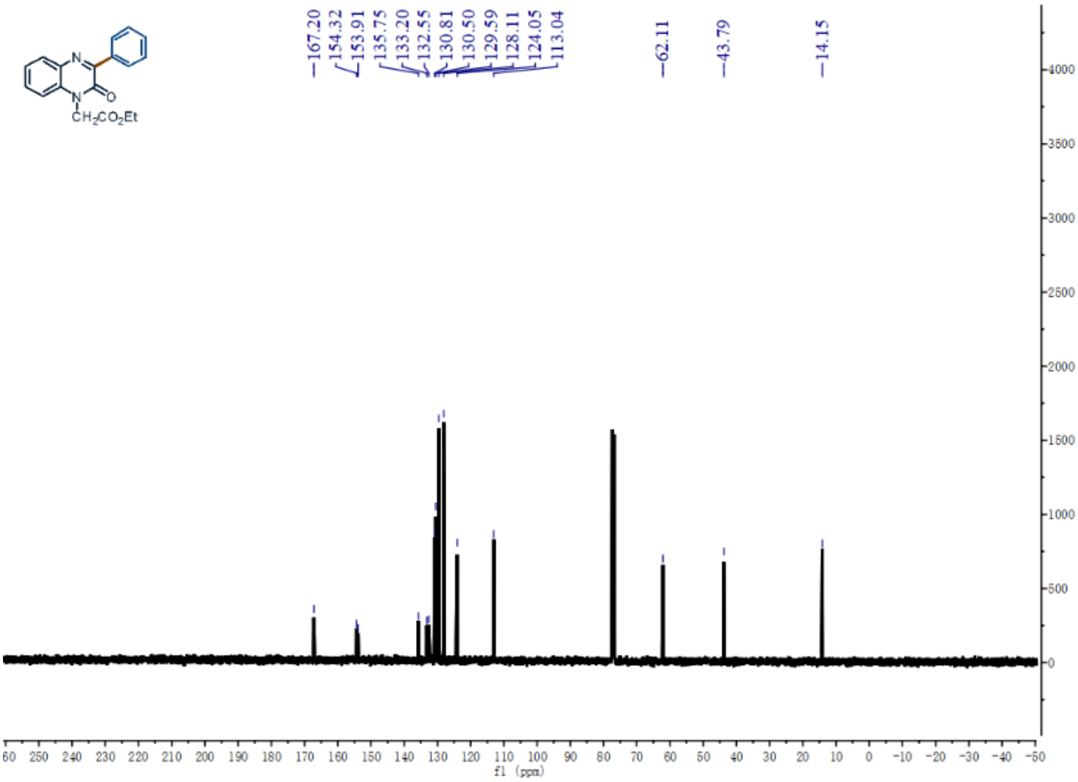

^{1}H NMR (400 MHz, $CDCl_3$) spectrum of compound **7o**

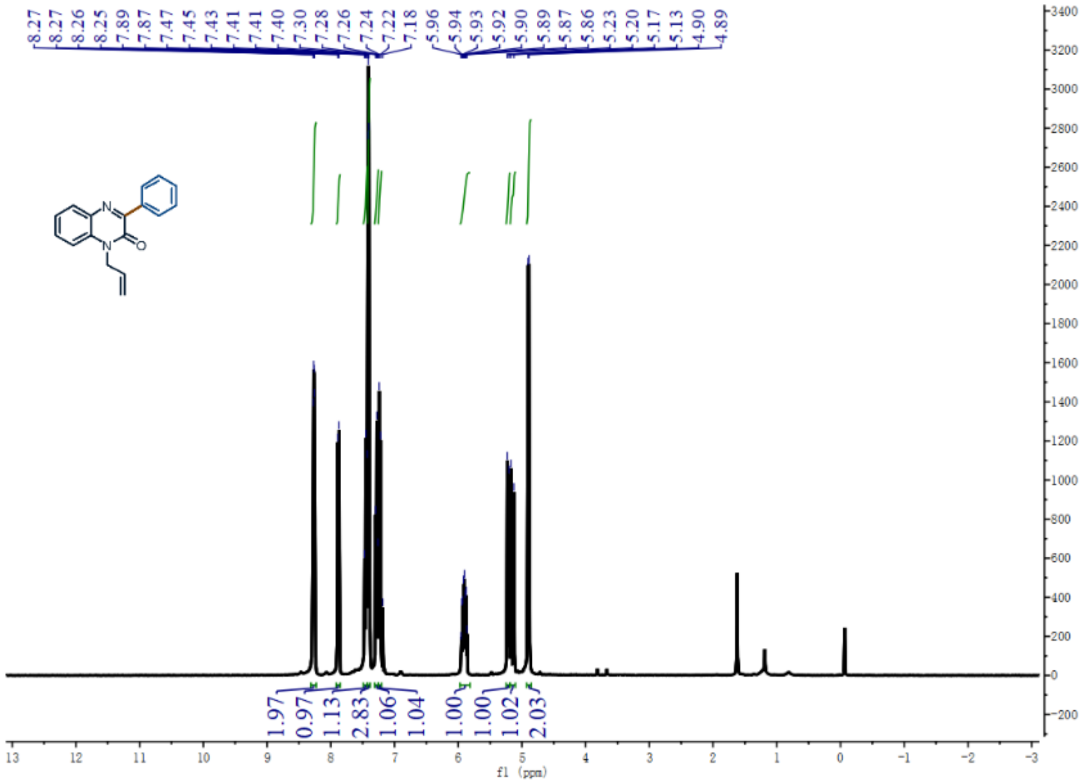

^{13}C NMR (100 MHz, $CDCl_3$) spectrum of compound **7o**

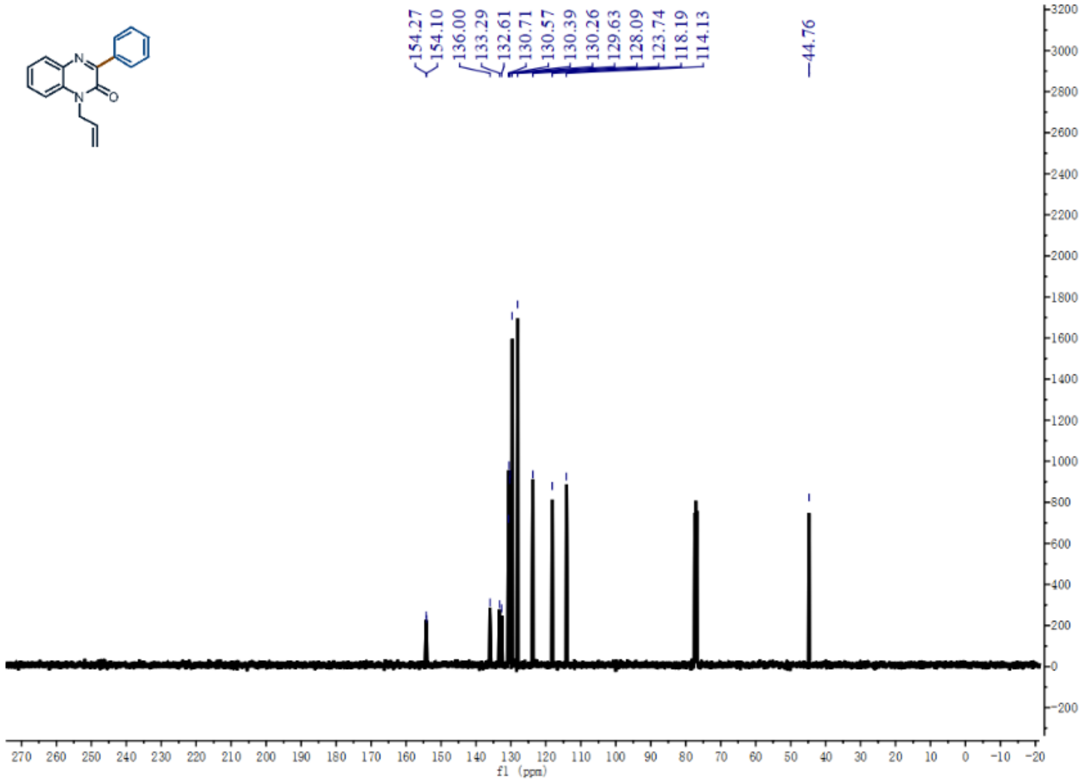

^{1}H NMR (400 MHz, $CDCl_3$) spectrum of compound **7p**

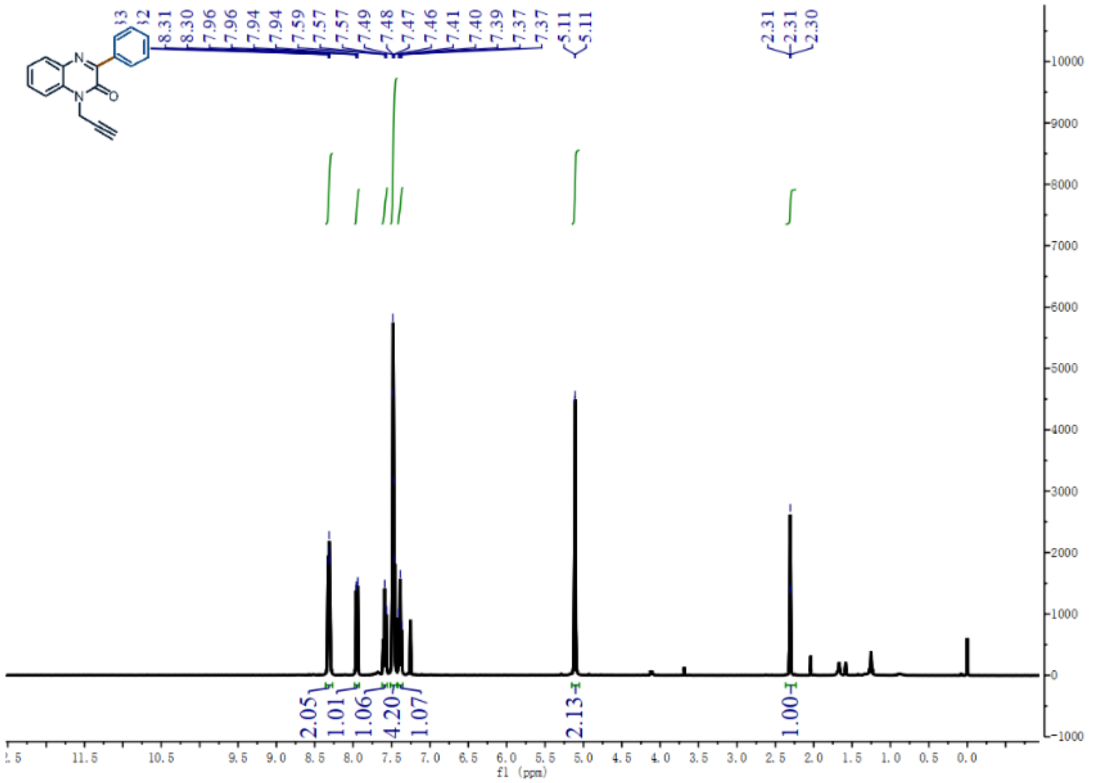

^{13}C NMR (100 MHz, $CDCl_3$) spectrum of compound **7p**

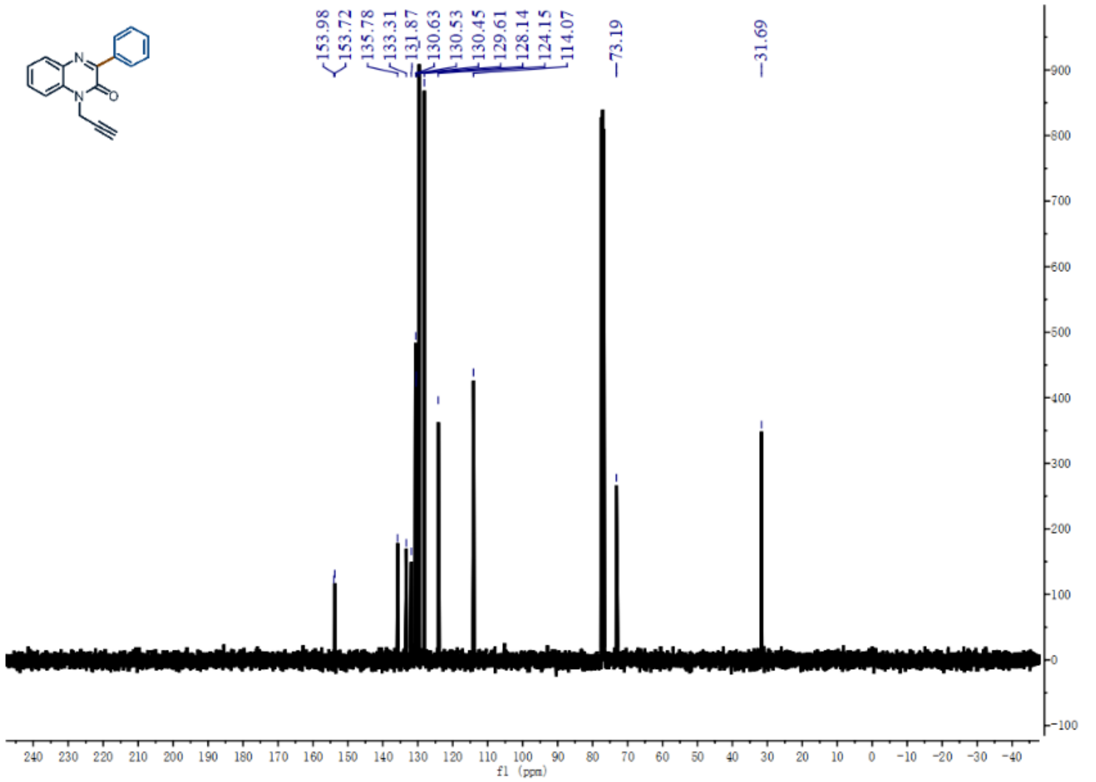

^{1}H NMR (400 MHz, $CDCl_3$) spectrum of compound **7q**

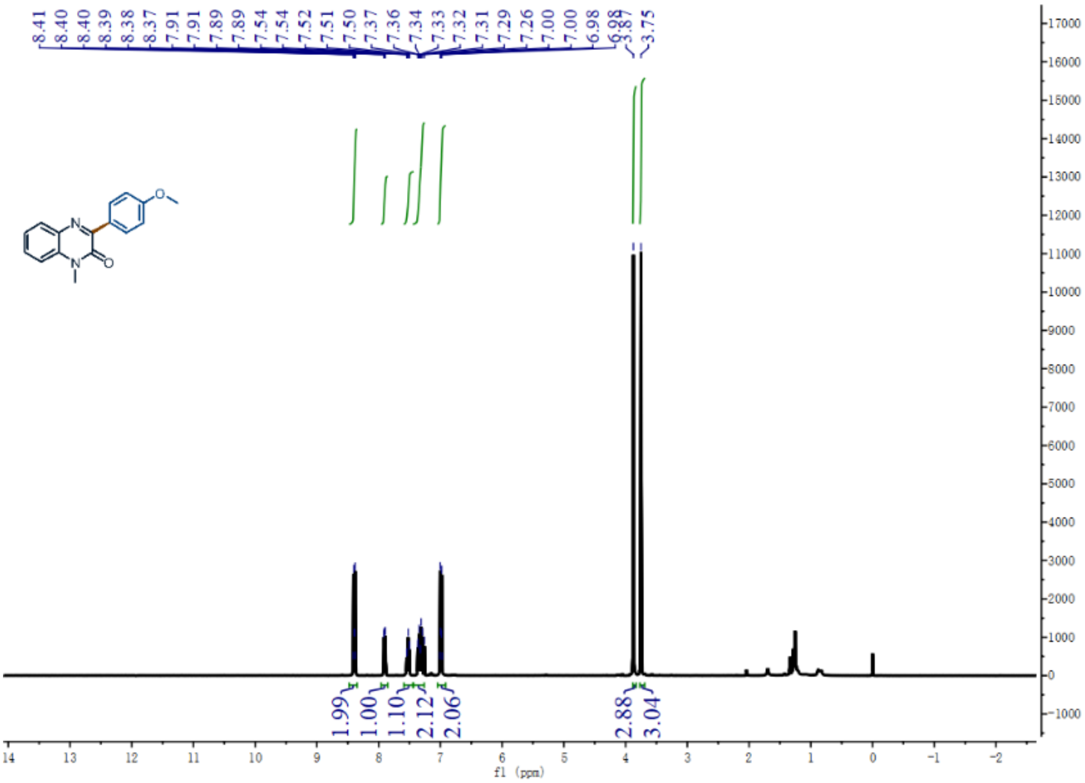

^{13}C NMR (100 MHz, $CDCl_3$) spectrum of compound **7q**

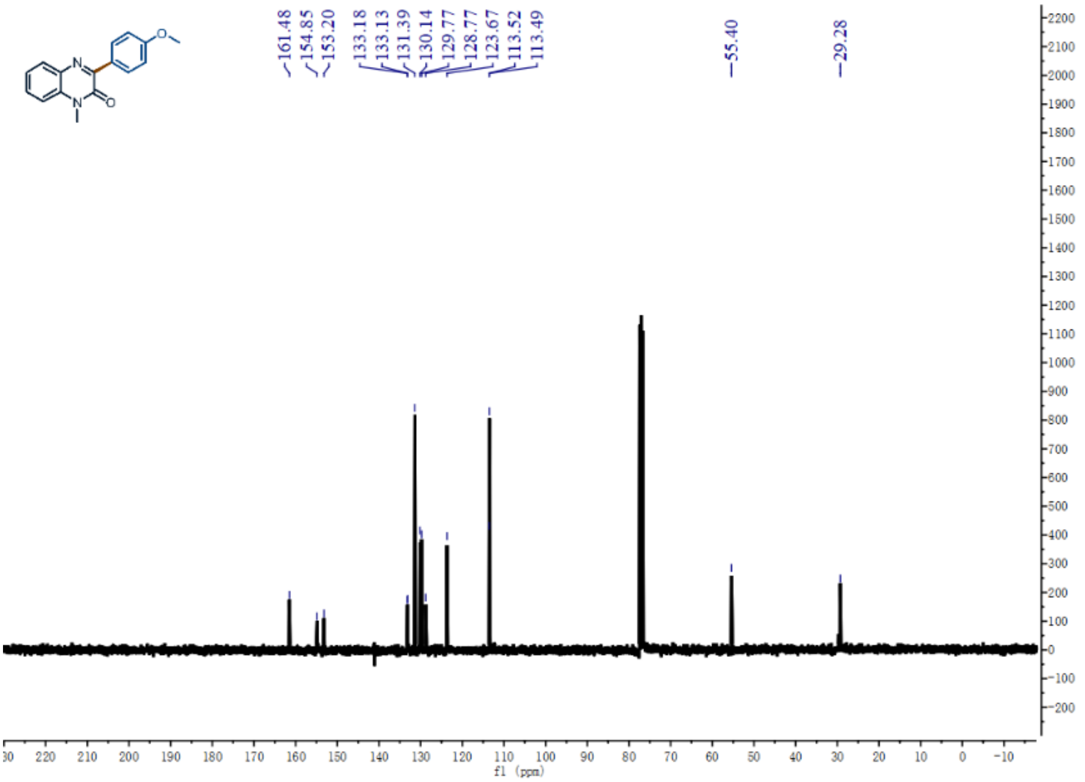

^{1}H NMR (400 MHz, $CDCl_3$) spectrum of compound **7r**

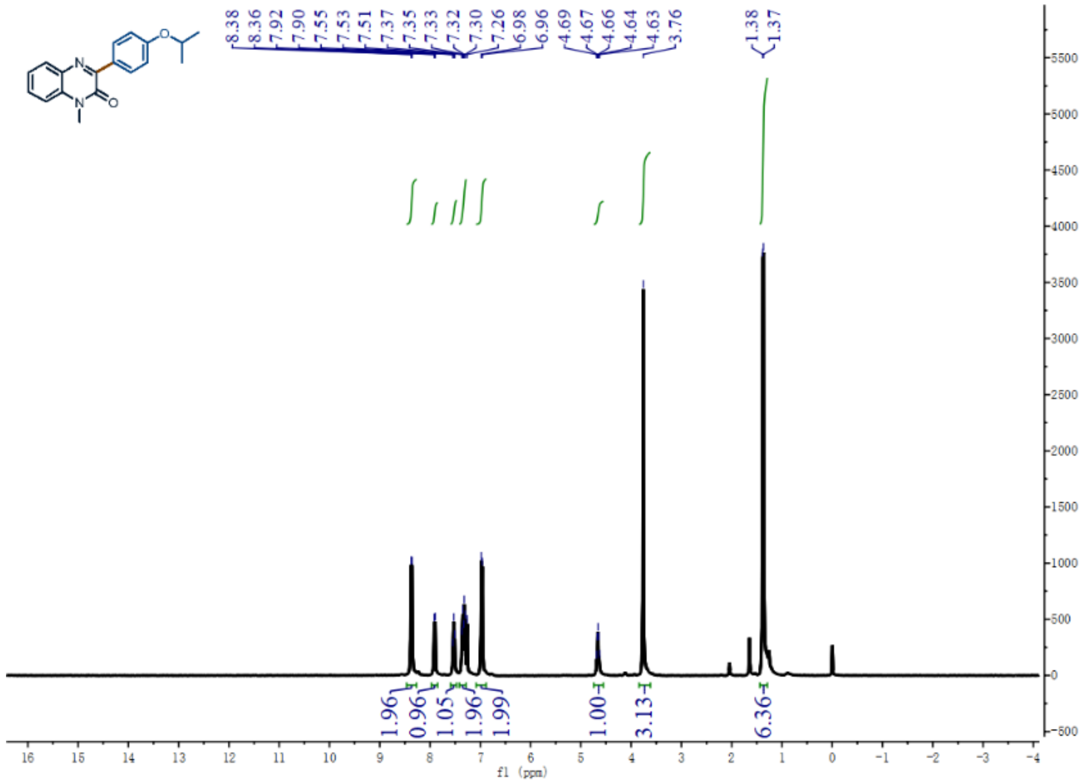

^{13}C NMR (100 MHz, $CDCl_3$) spectrum of compound **7r**

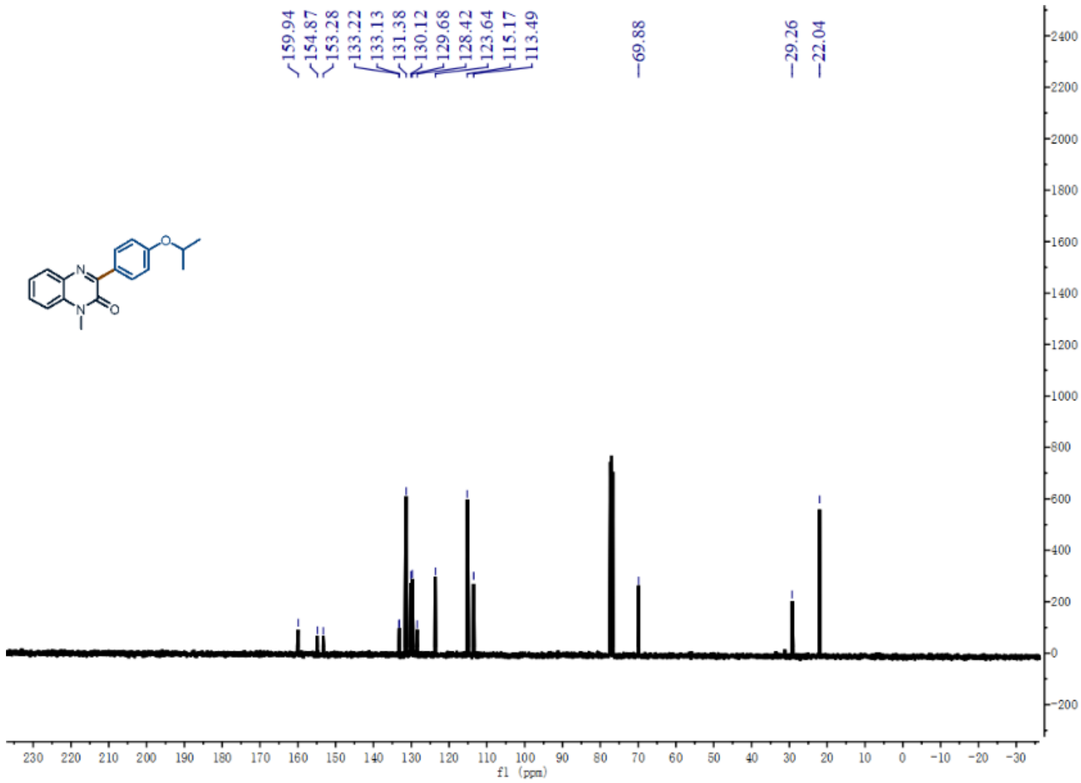

^{1}H NMR (400 MHz, $CDCl_3$) spectrum of compound **7s**

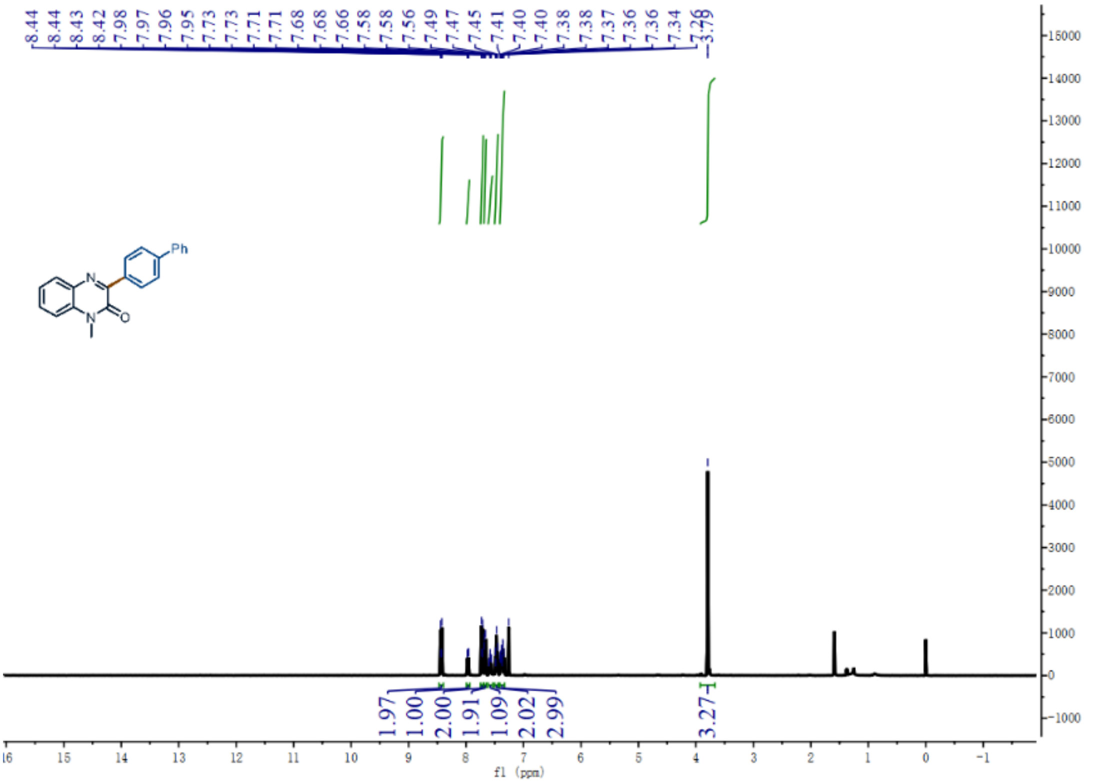

^{13}C NMR (100 MHz, $CDCl_3$) spectrum of compound **7s**

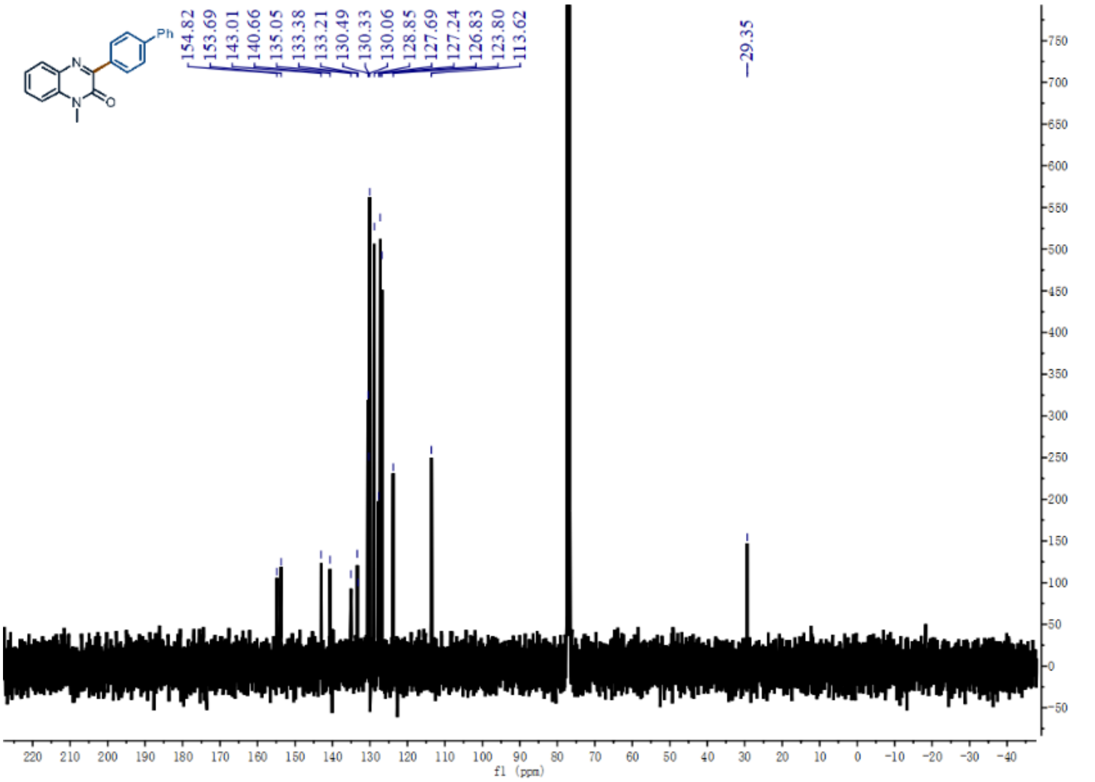

^{1}H NMR (400 MHz, $CDCl_3$) spectrum of compound **7t**

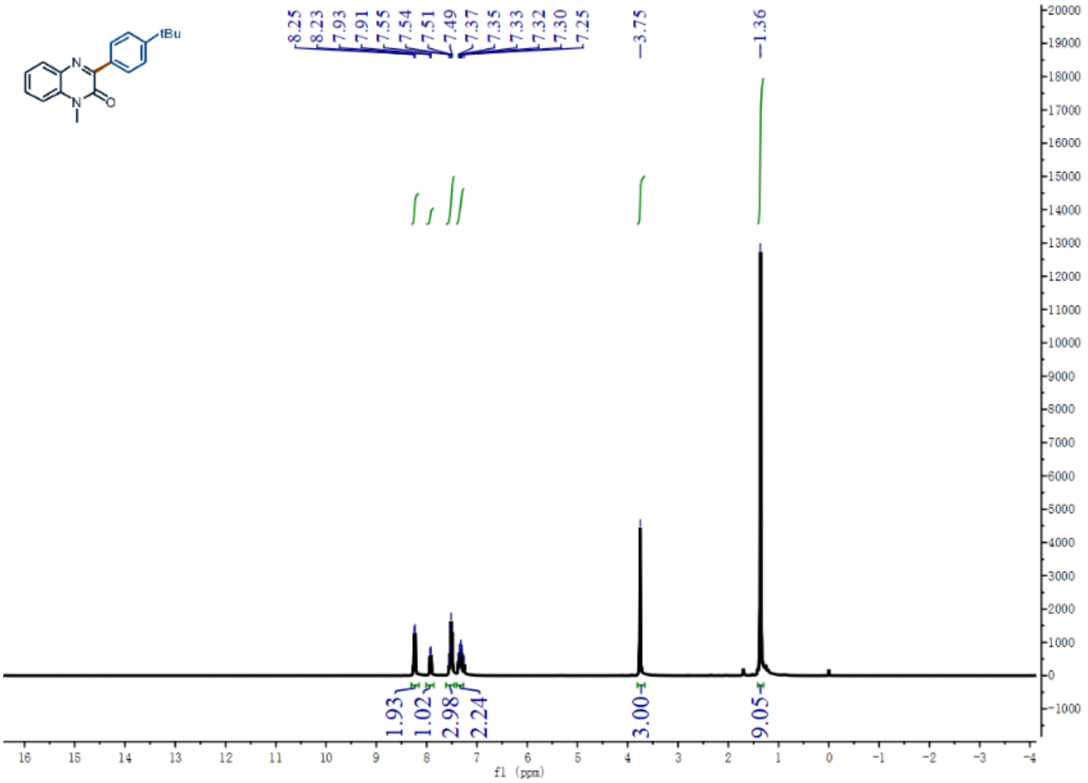

^{13}C NMR (100 MHz, $CDCl_3$) spectrum of compound **7t**

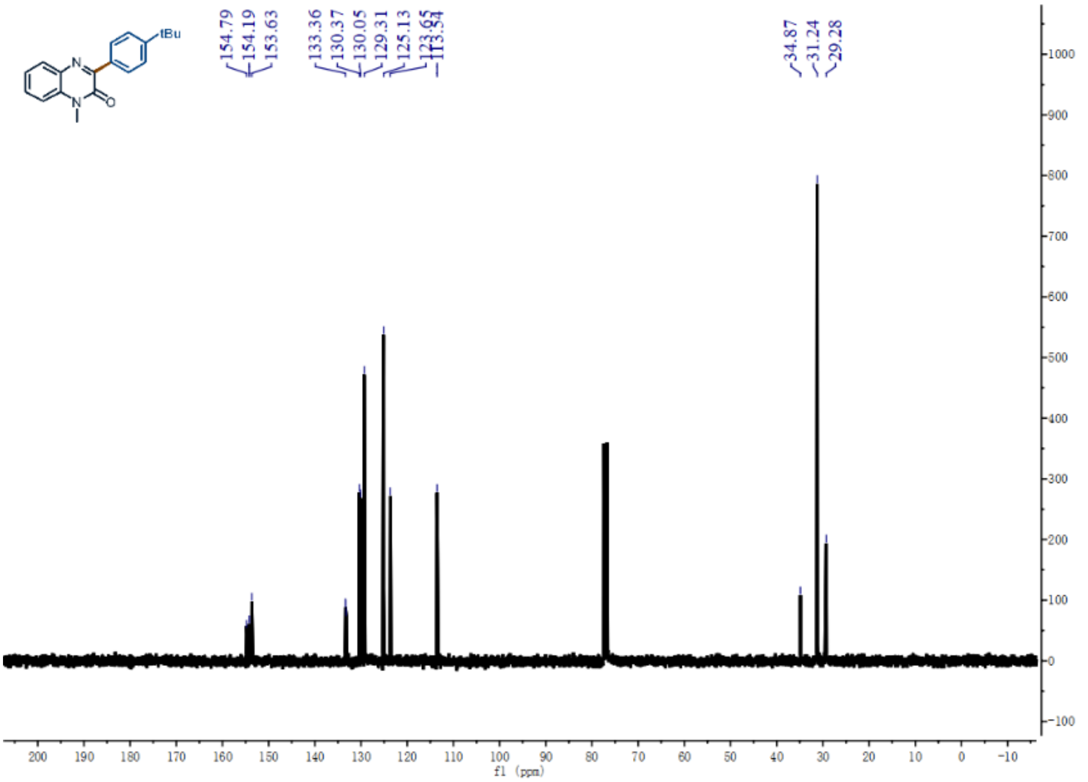

^{1}H NMR (400 MHz, $CDCl_3$) spectrum of compound **7u**

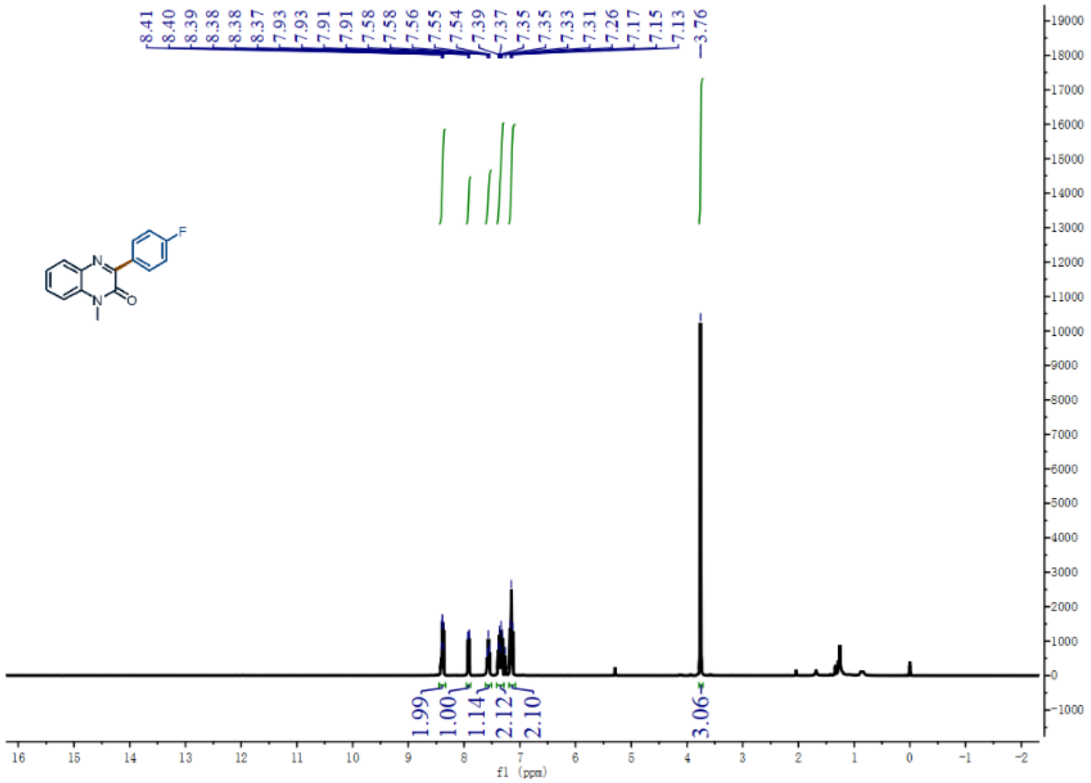

^{13}C NMR (100 MHz, CDCl3) spectrum of compound **7u**

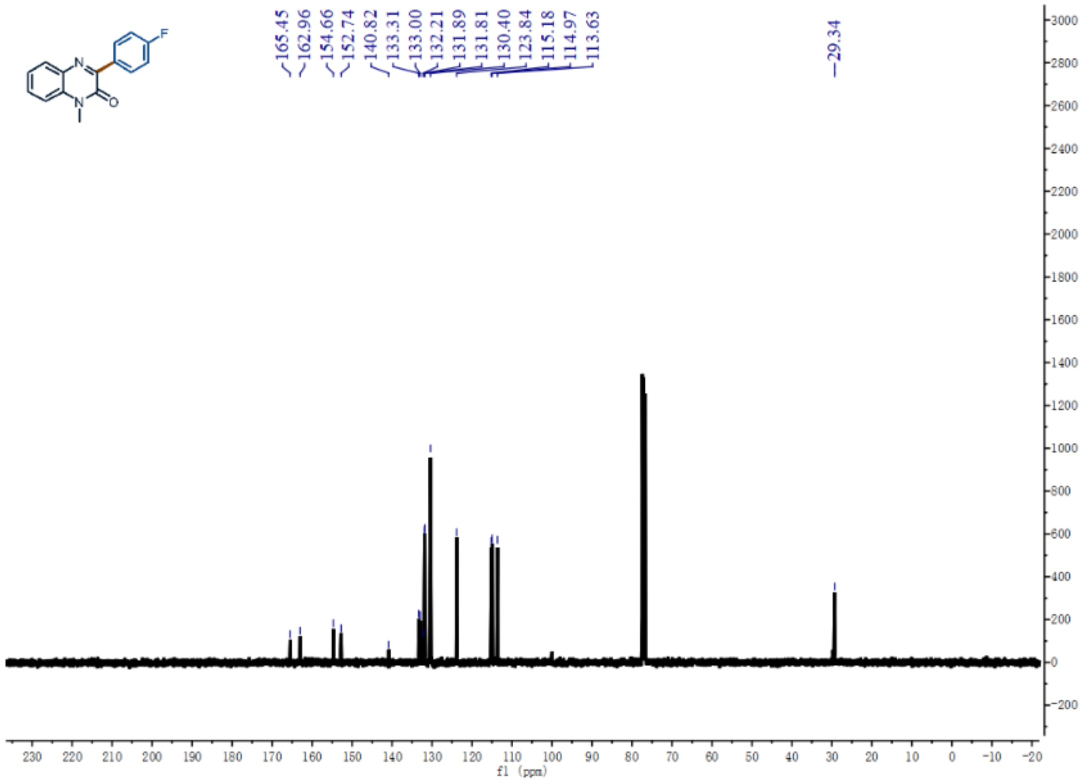

^{1}H NMR (400 MHz, CDCl3) spectrum of compound **7v**

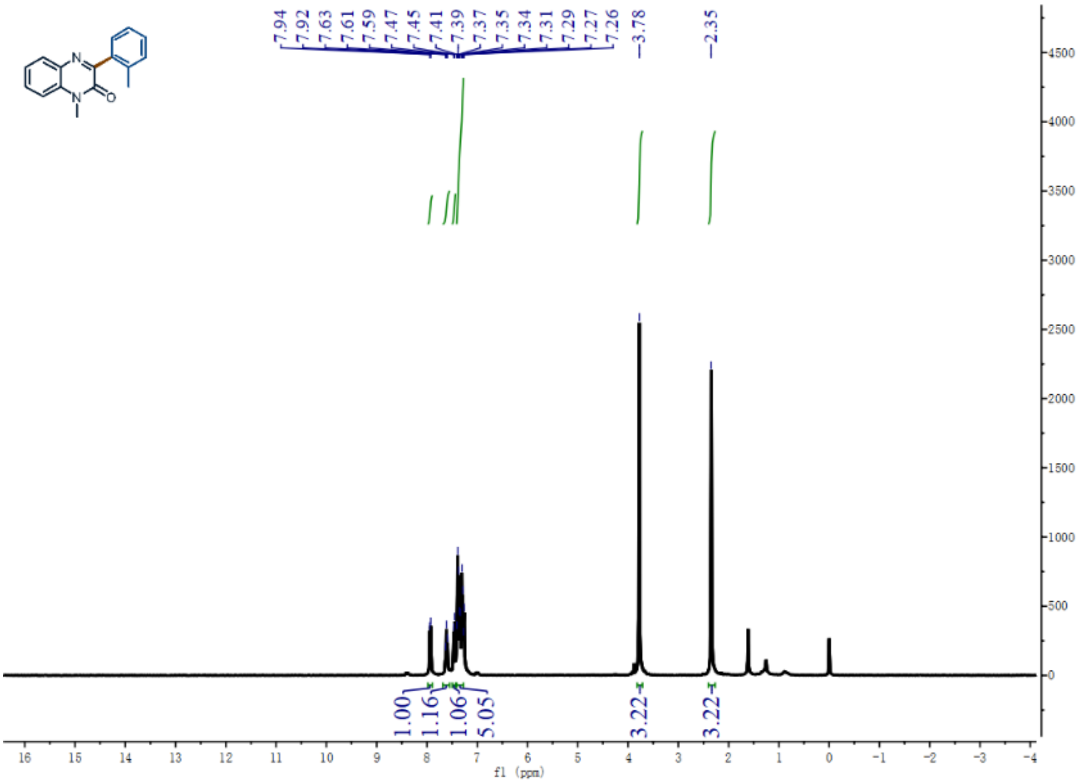

^{13}C NMR (100 MHz, $CDCl_3$) spectrum of compound **7v**

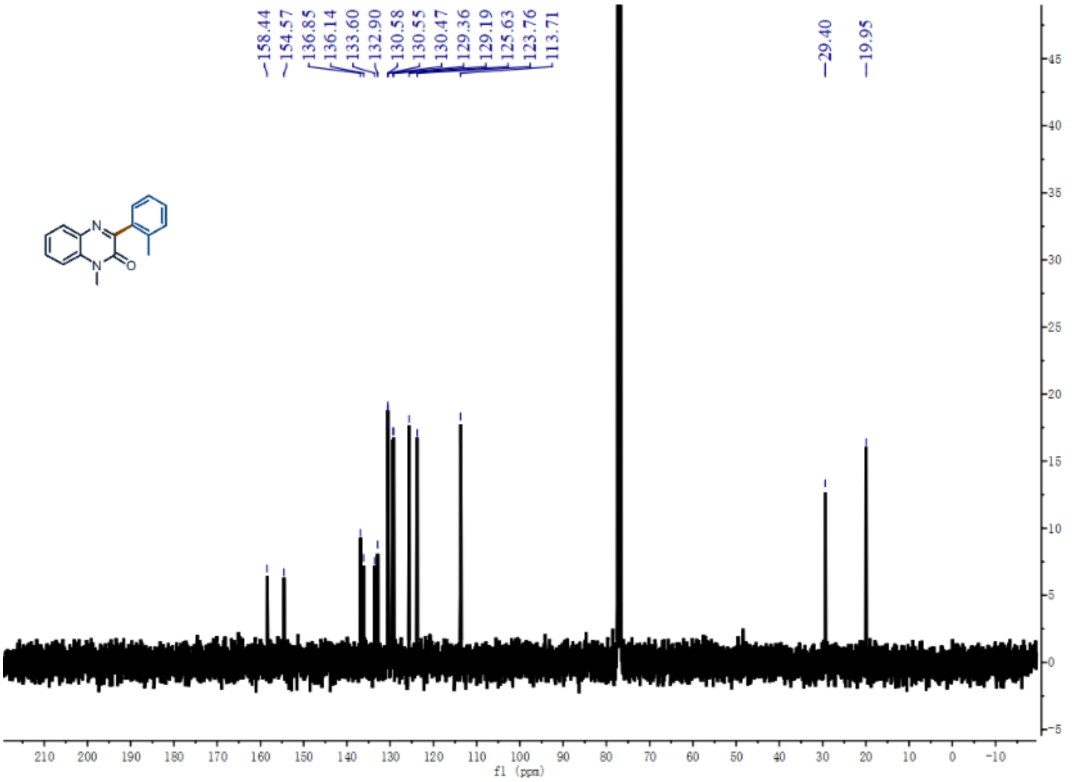

^{1}H NMR (400 MHz, CDCl$_3$) spectrum of compound **9a**

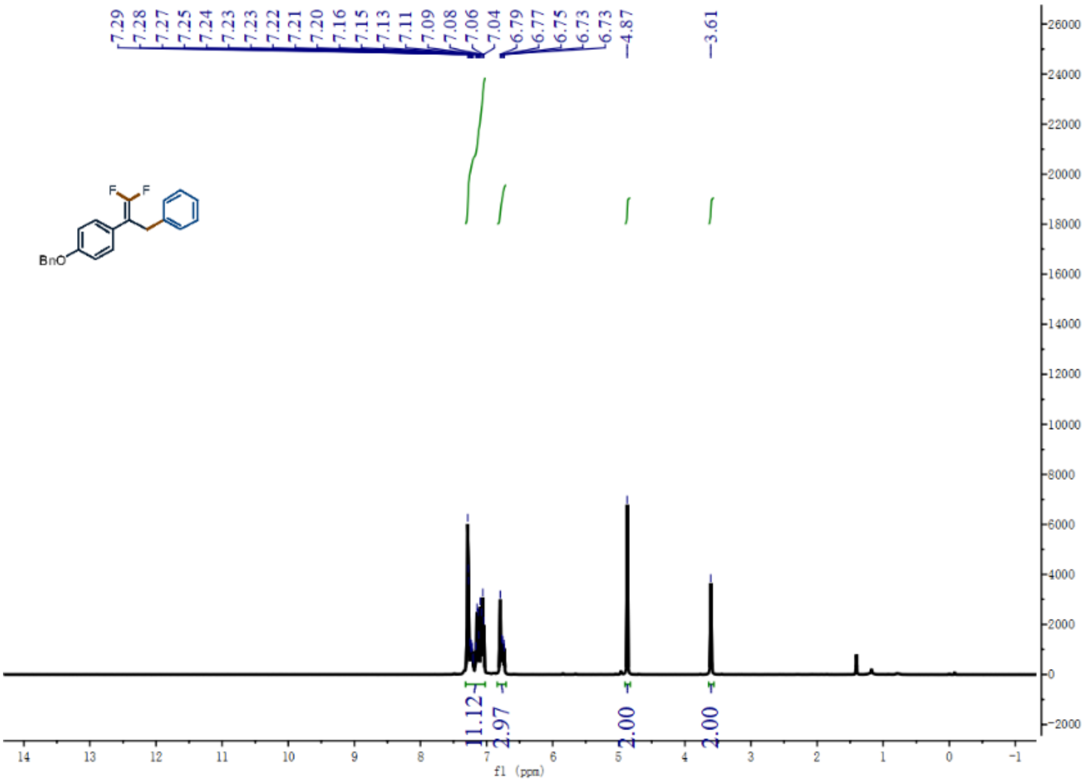

^{13}C NMR (100 MHz, CDCl$_3$) spectrum of compound **9a**

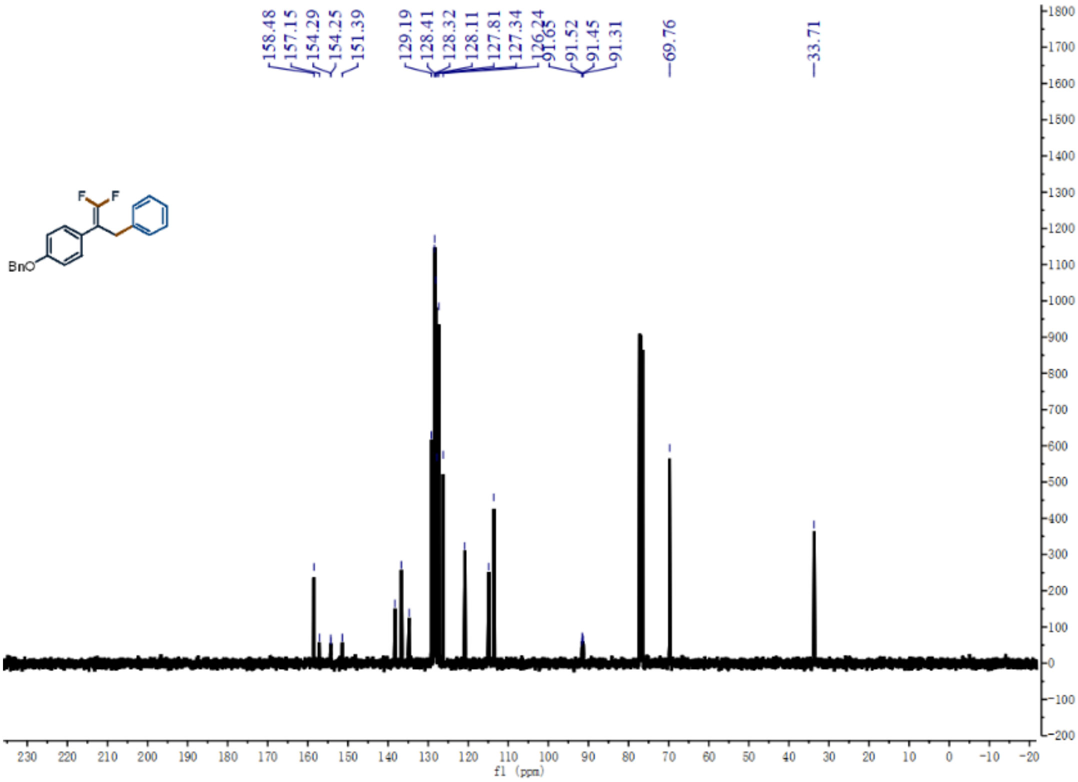

^{19}F NMR (376 MHz, CDCl$_3$) spectrum of compound **9a**

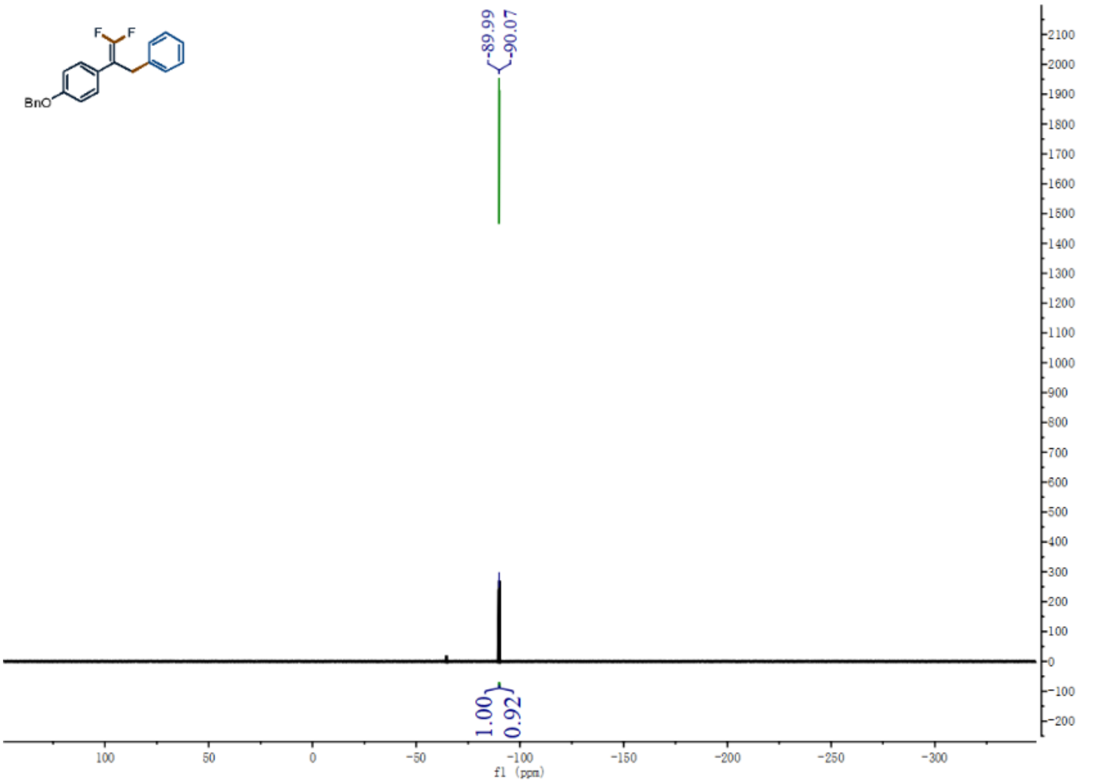

^{1}H NMR (400 MHz, $CDCl_3$) spectrum of compound **9b**

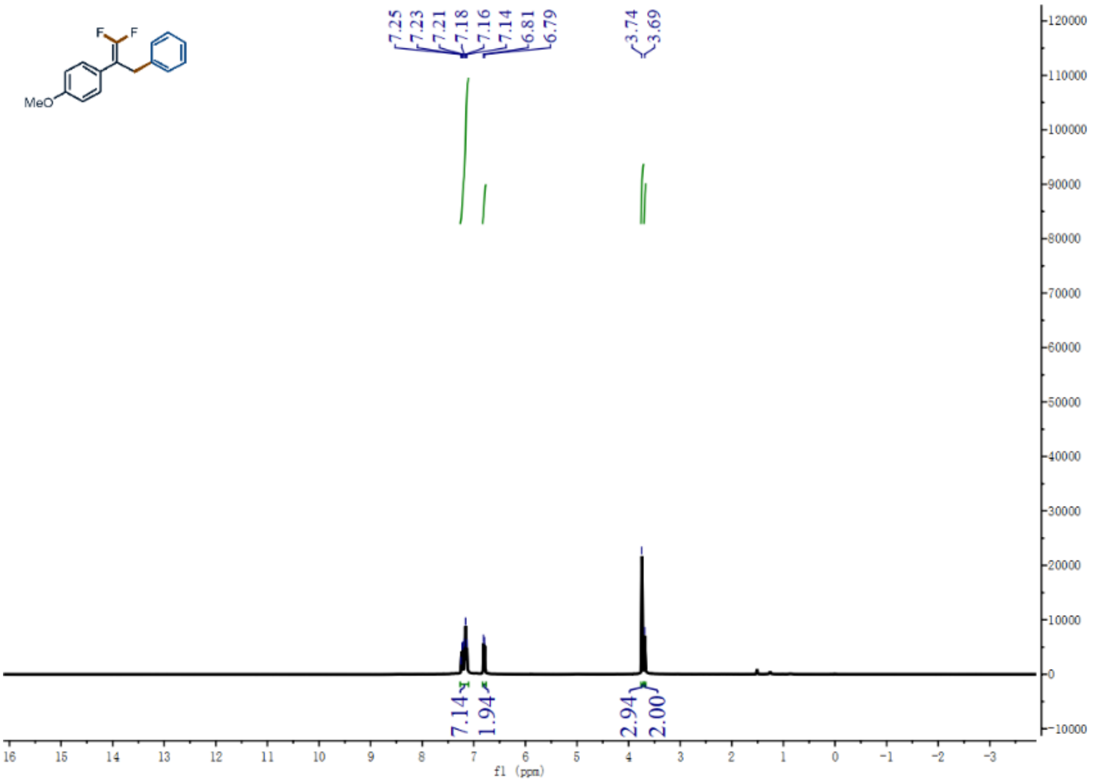

^{13}C NMR (100 MHz, $CDCl_3$) spectrum of compound **9b**

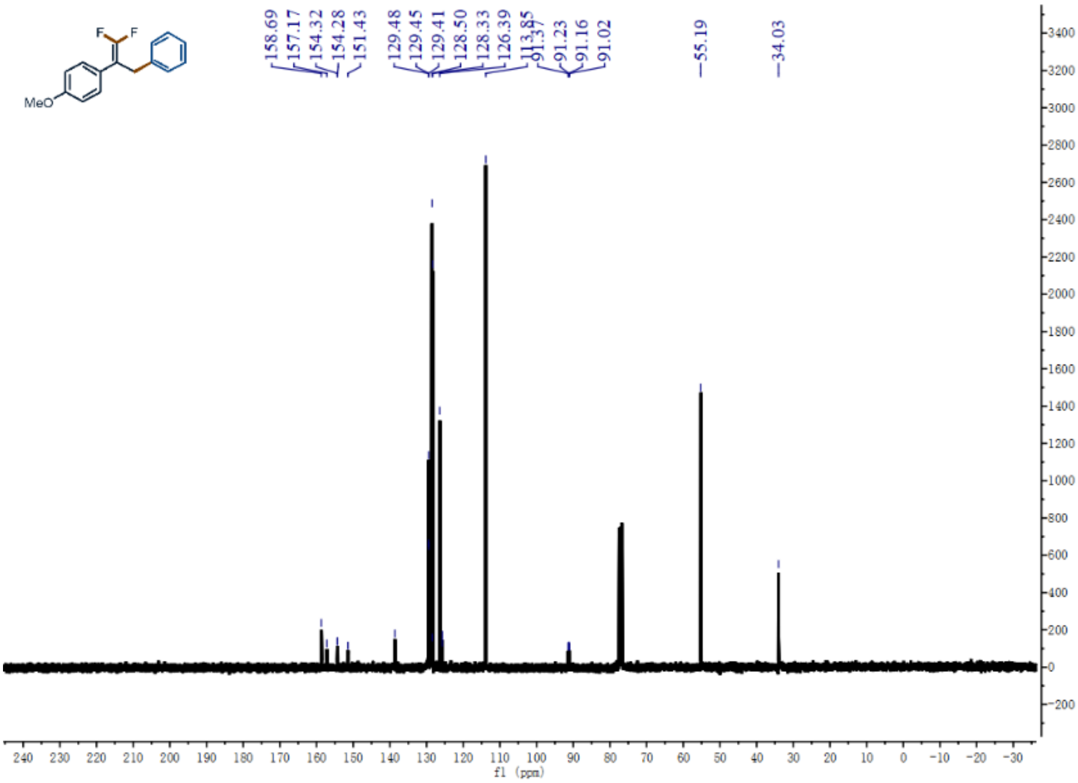

^{19}F NMR (376 MHz, $CDCl_3$) spectrum of compound **9b**

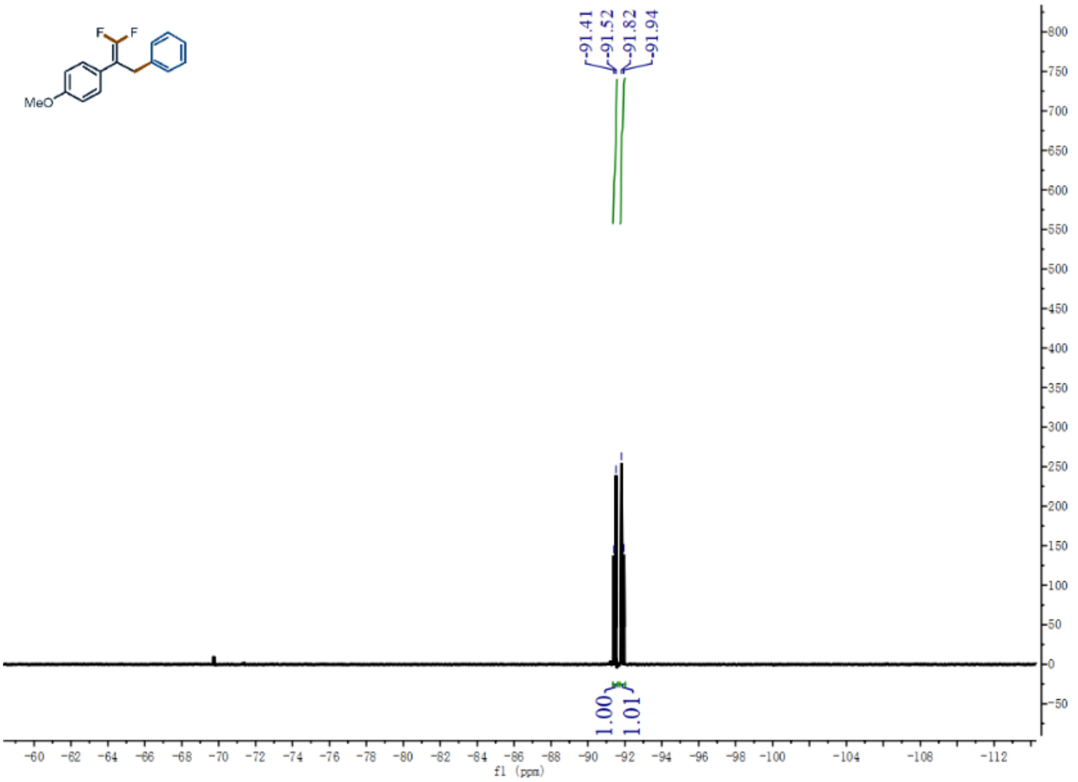

^{1}H NMR (400 MHz, $CDCl_3$) spectrum of compound **9c**

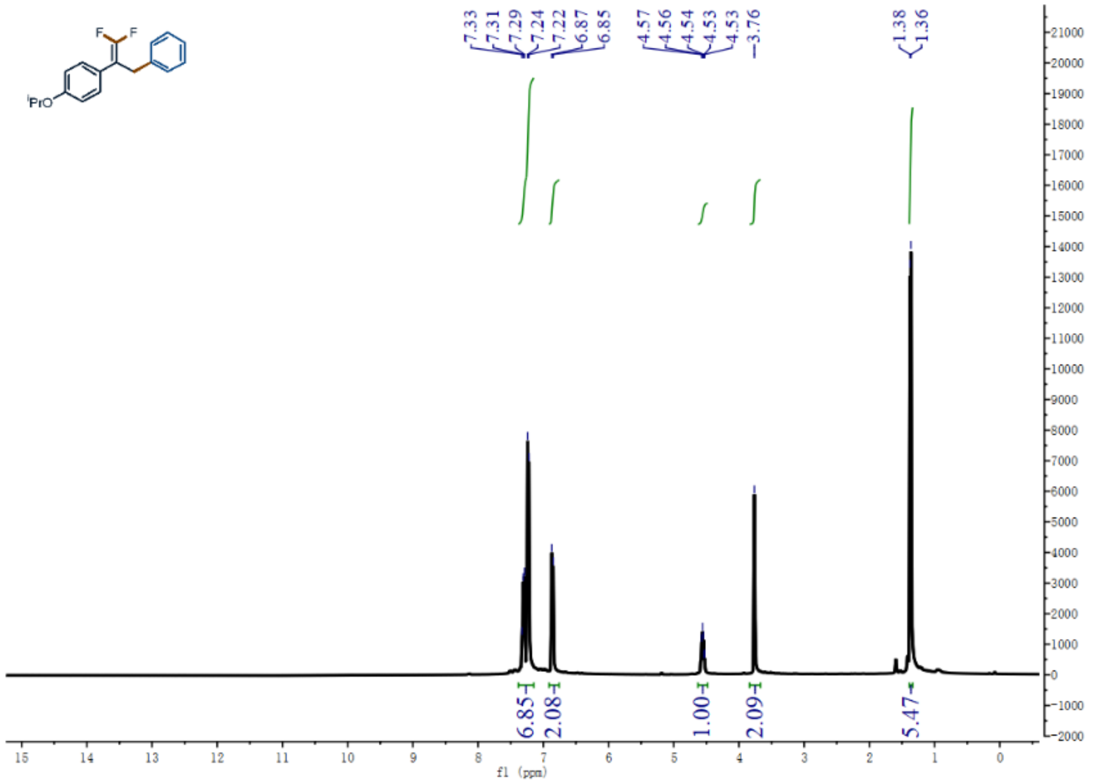

^{13}C NMR (100 MHz, $CDCl_3$) spectrum of compound **9c**

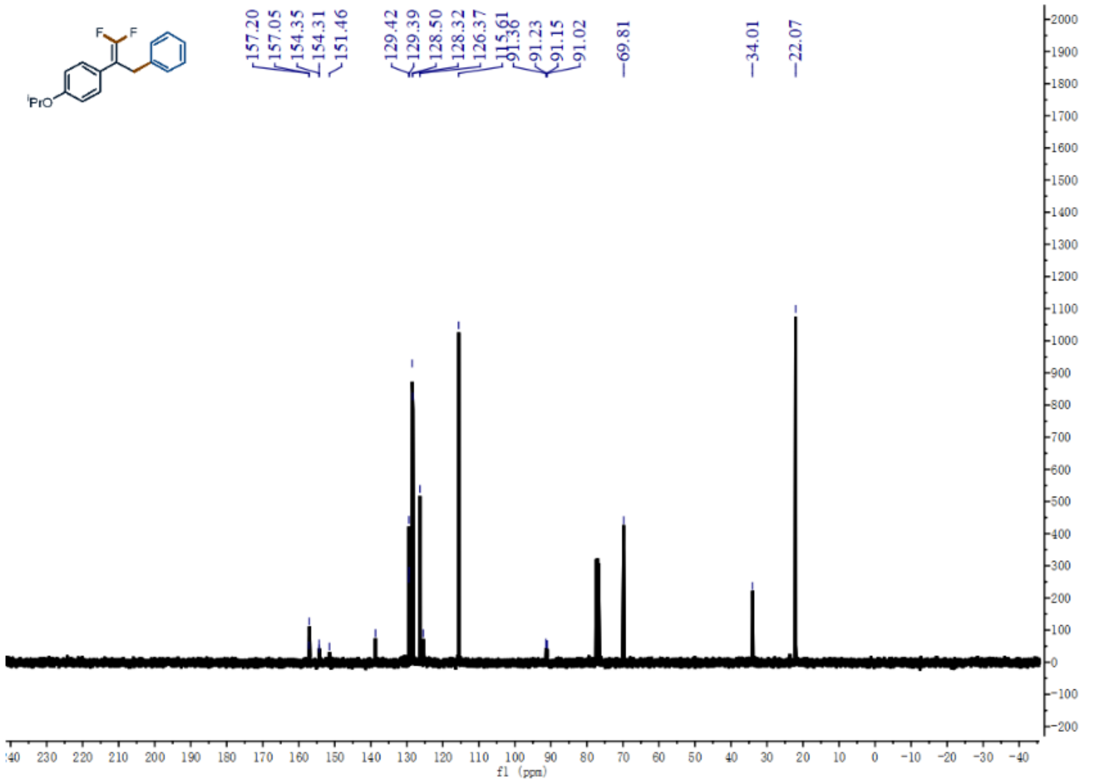

^{19}F NMR (376 MHz, $CDCl_3$) spectrum of compound **9c**

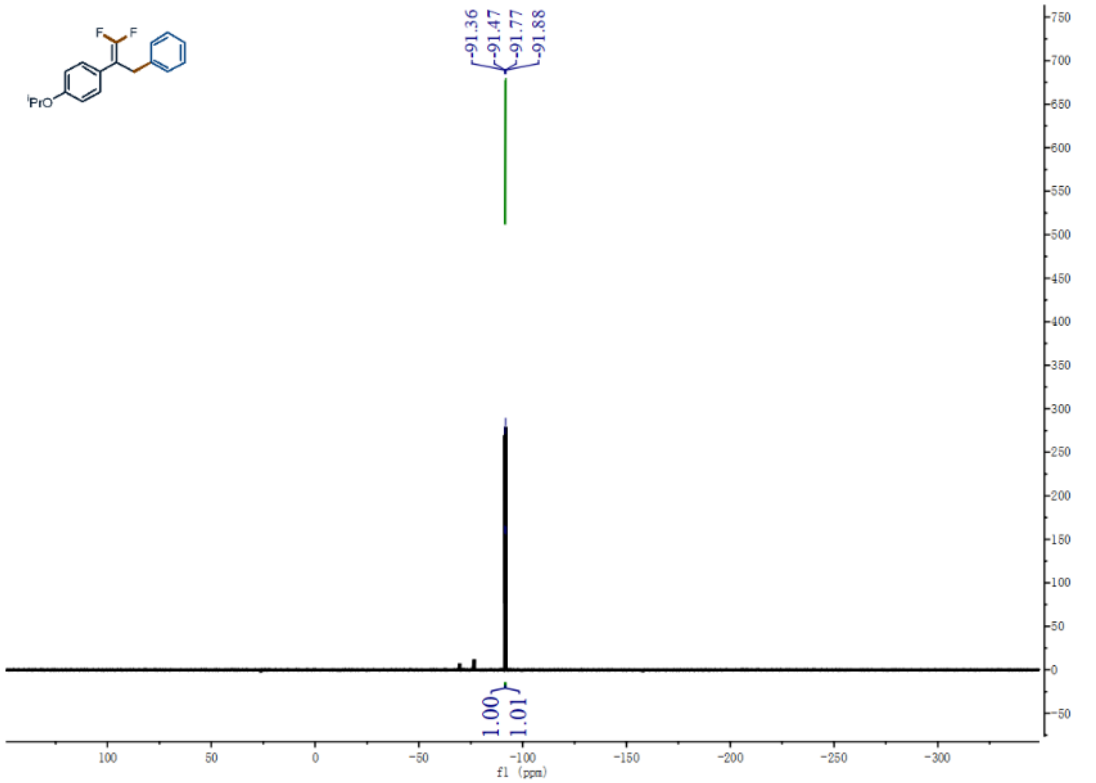

^{1}H NMR (400 MHz, $CDCl_3$) spectrum of compound **9d**

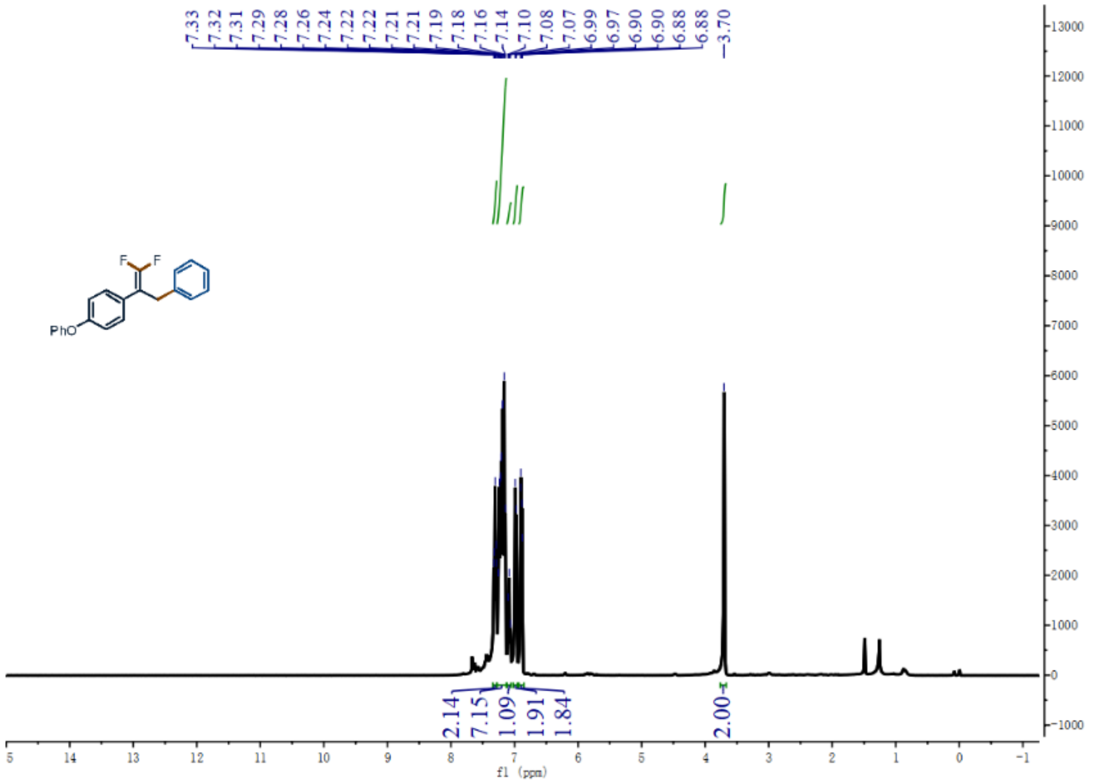

^{13}C NMR (100 MHz, $CDCl_3$) spectrum of compound **9d**

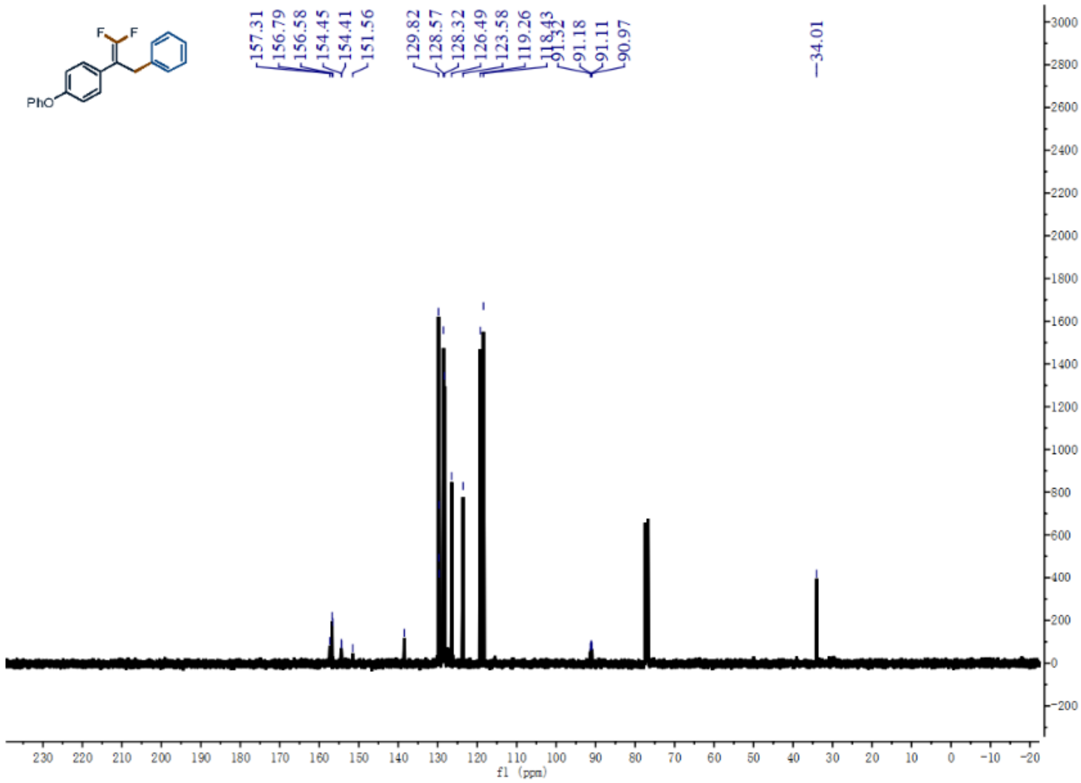

^{19}F NMR (376 MHz, $CDCl_3$) spectrum of compound **9d**

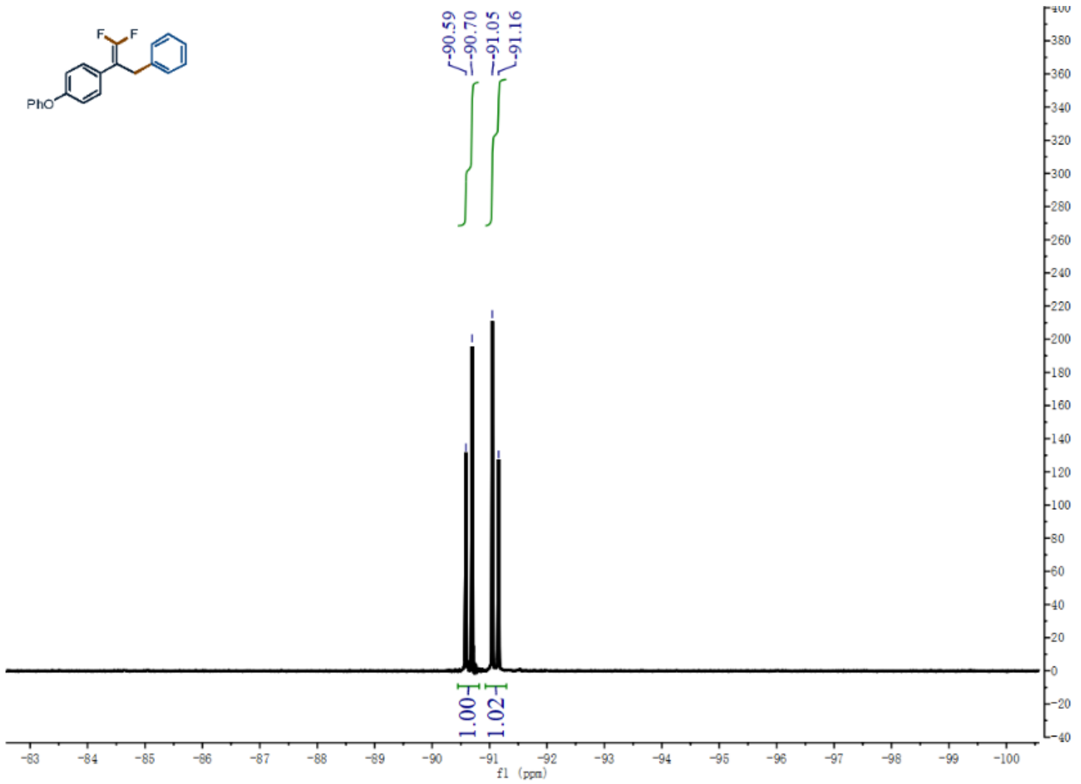

^{1}H NMR (400 MHz, $CDCl_3$) spectrum of compound **9e**

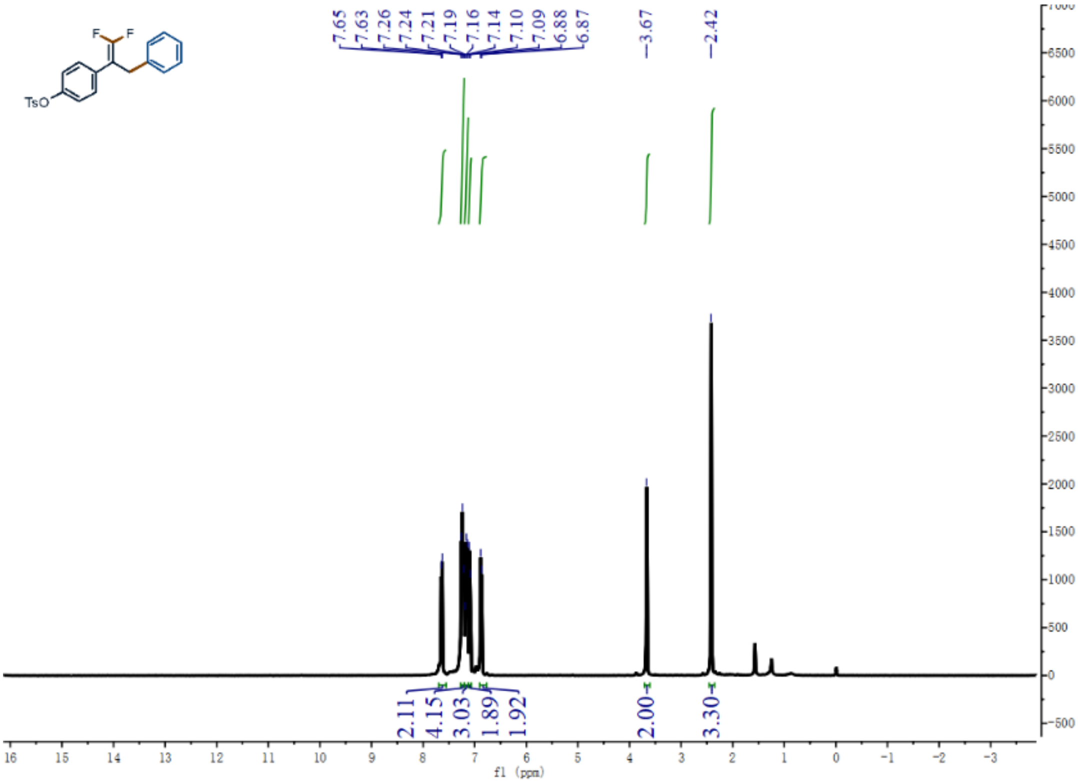

^{13}C NMR (100 MHz, $CDCl_3$) spectrum of compound **9e**

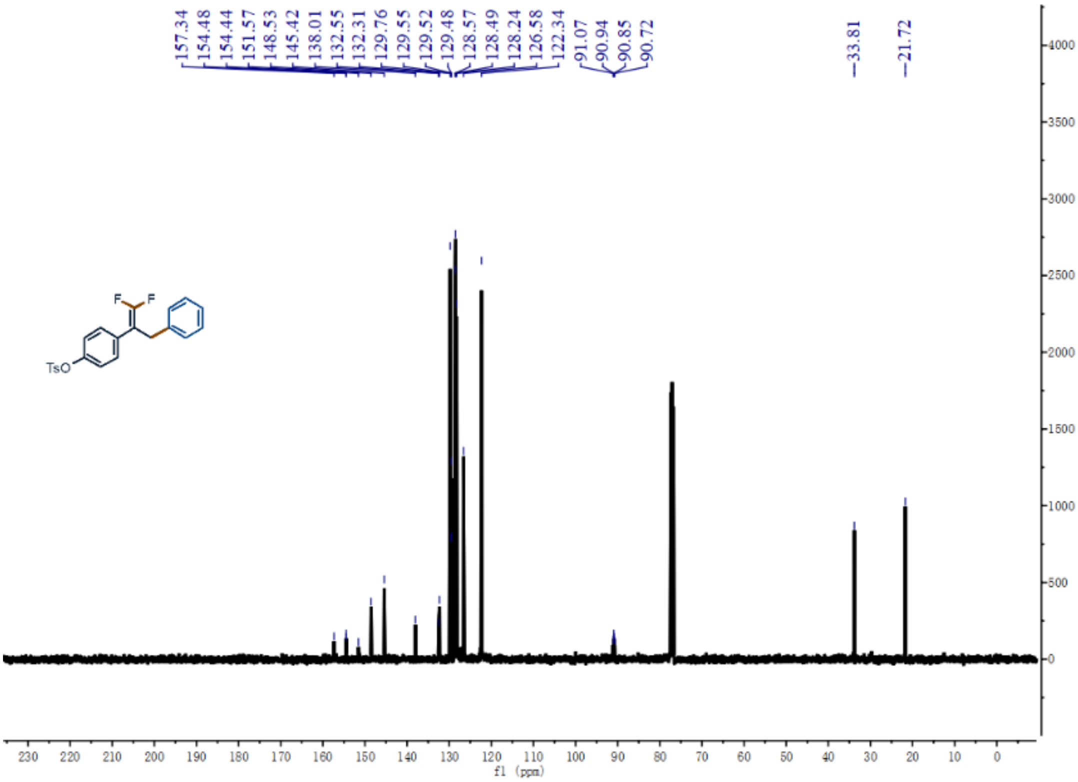

^{19}F NMR (376 MHz, $CDCl_3$) spectrum of compound **9e**

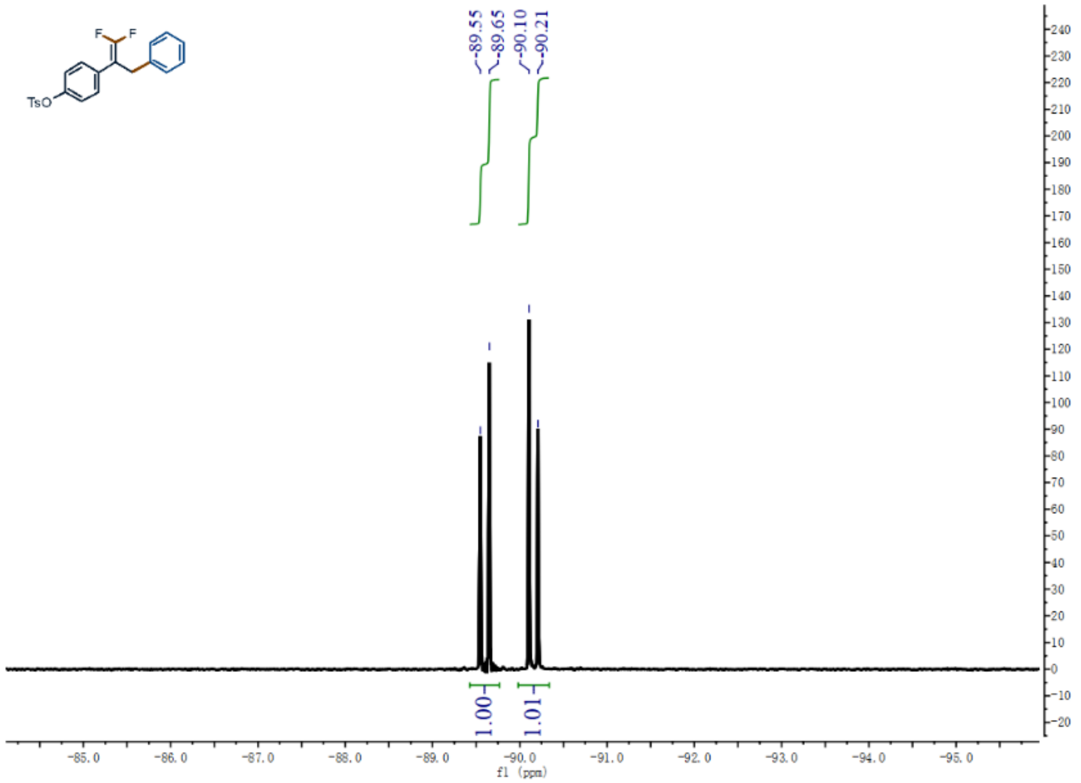

^{1}H NMR (400 MHz, $CDCl_3$) spectrum of compound **9f**

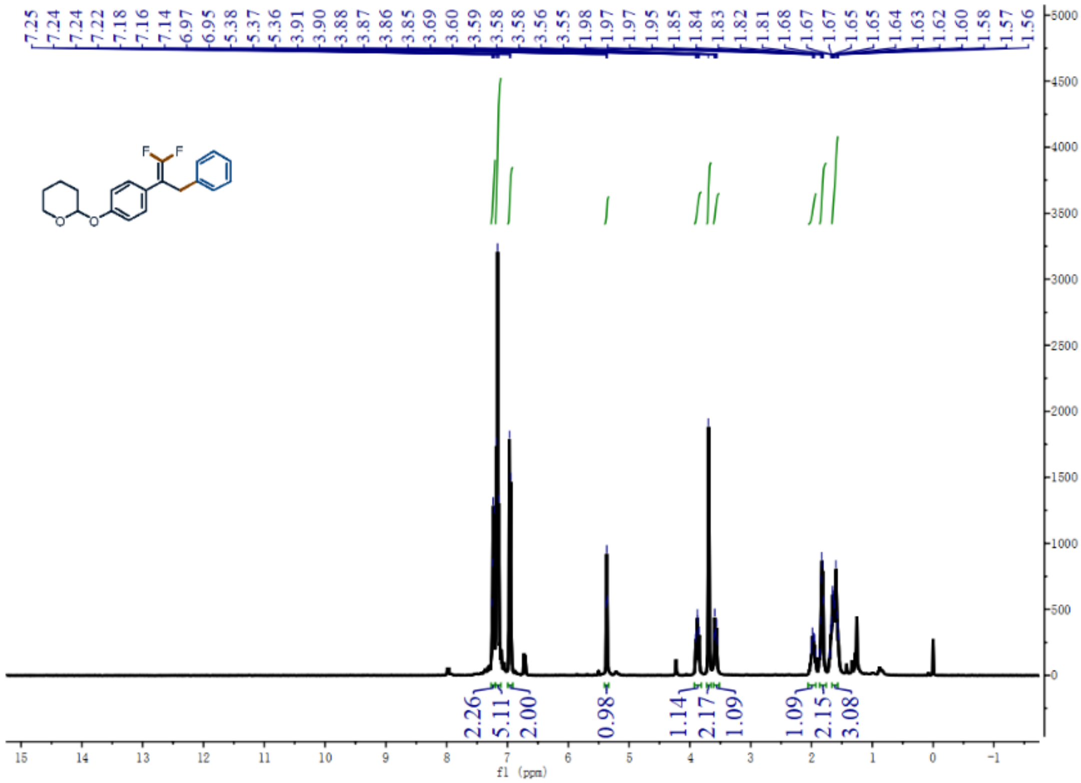

^{13}C NMR (100 MHz, $CDCl_3$) spectrum of compound **9f**

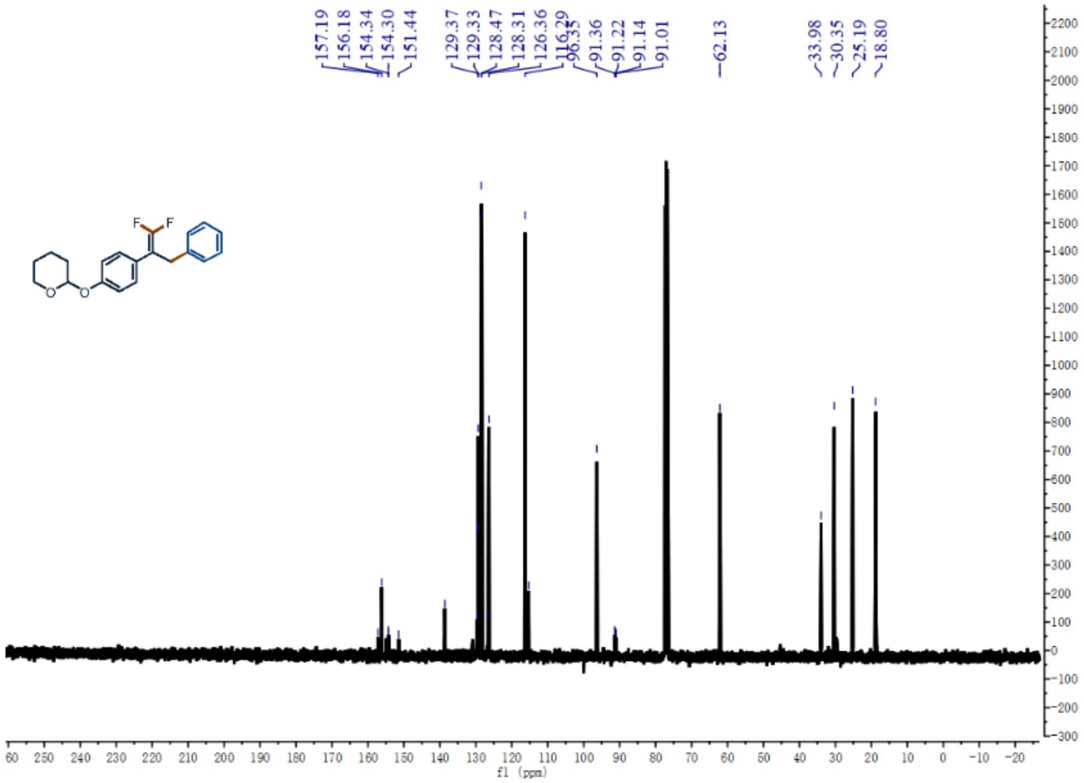

^{19}F NMR (376 MHz, $CDCl_3$) spectrum of compound **9f**

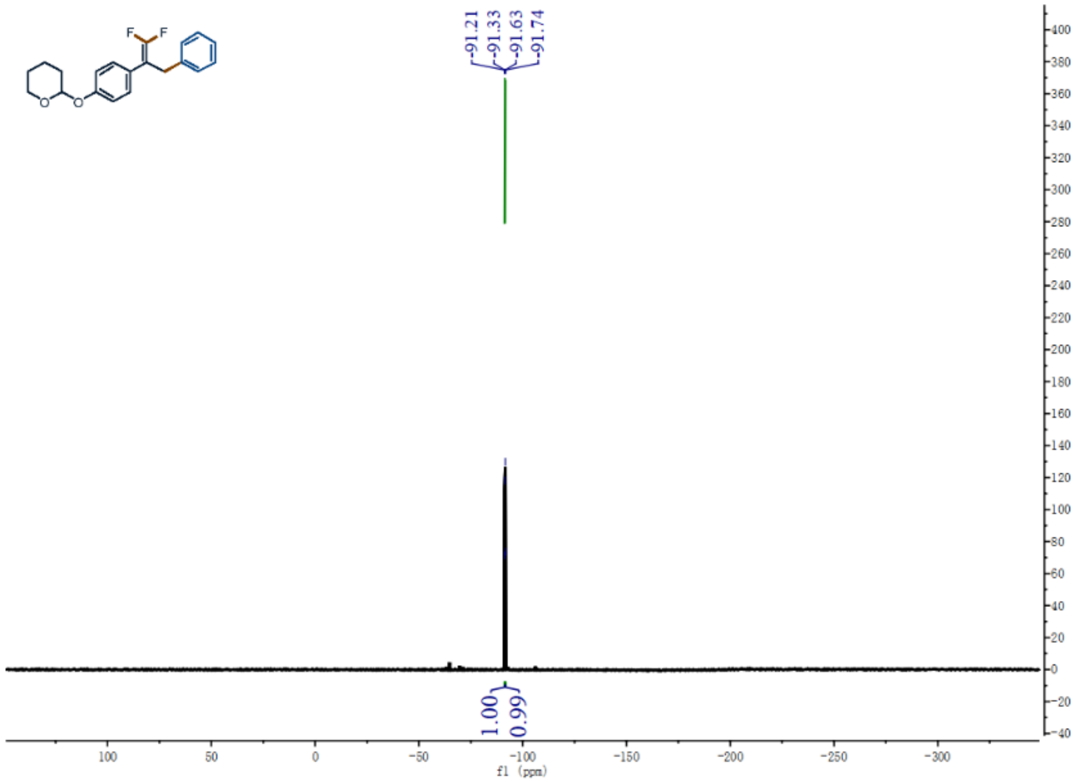

^{1}H NMR (400 MHz, $CDCl_3$) spectrum of compound **9g**

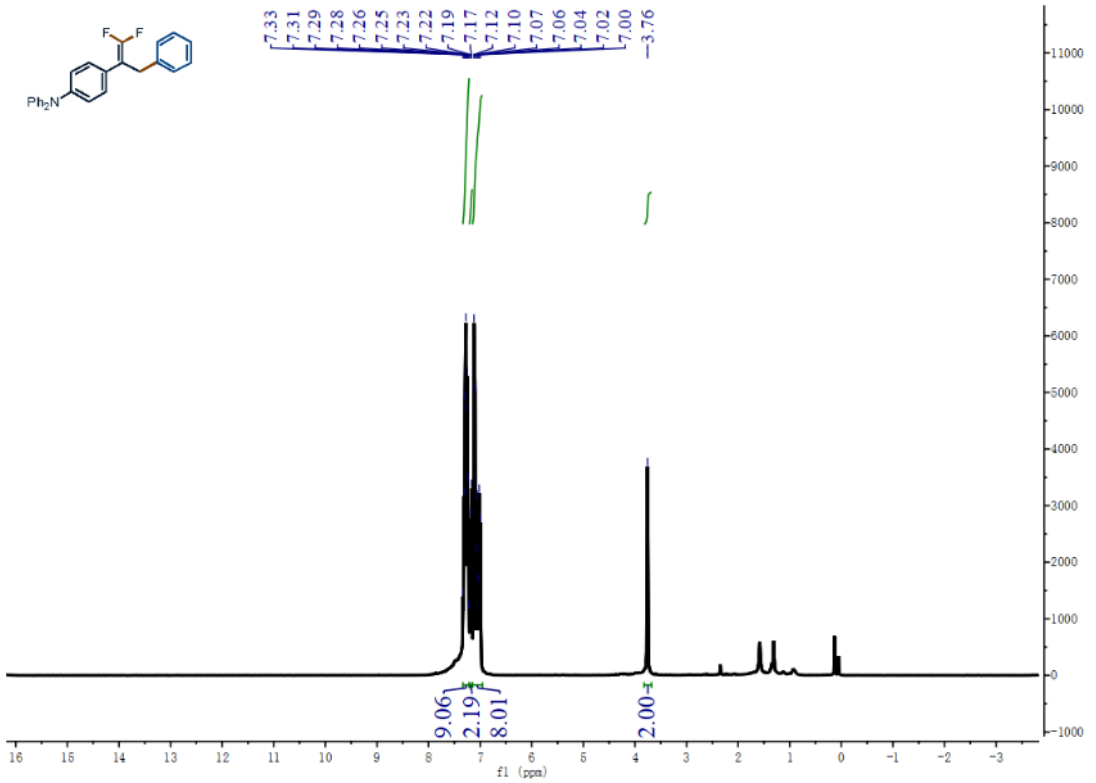

^{13}C NMR (100 MHz, $CDCl_3$) spectrum of compound **9g**

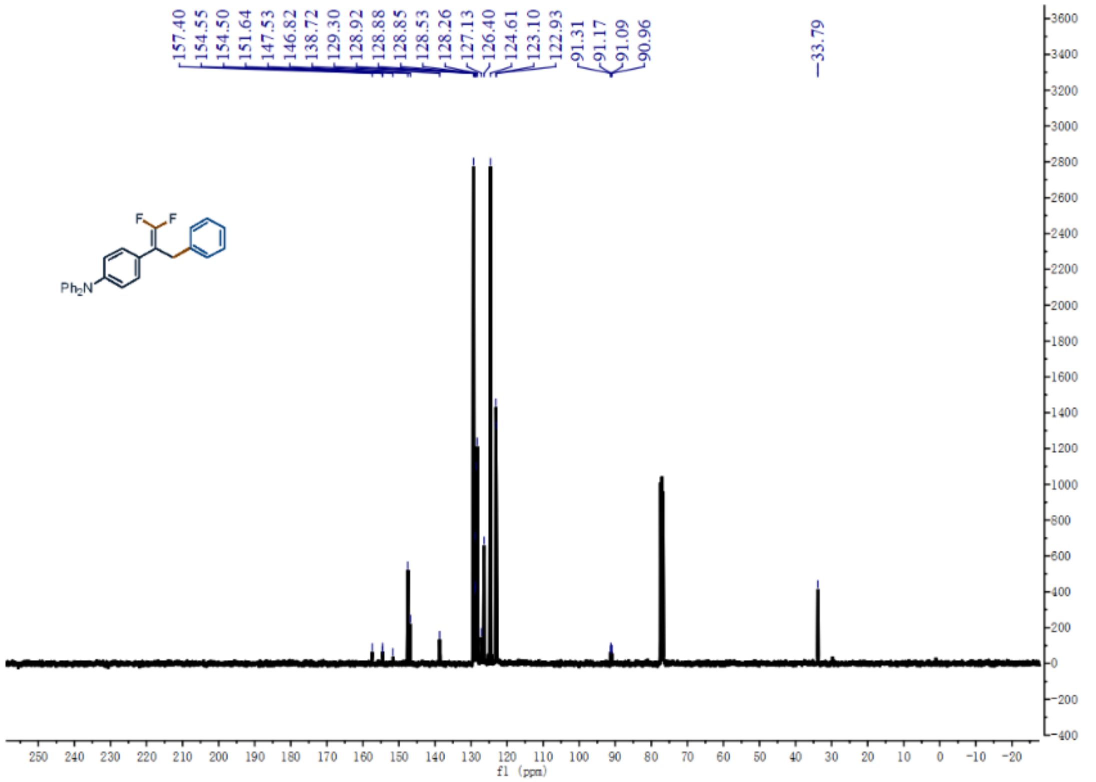

^{19}F NMR (376 MHz, $CDCl_3$) spectrum of compound **9g**

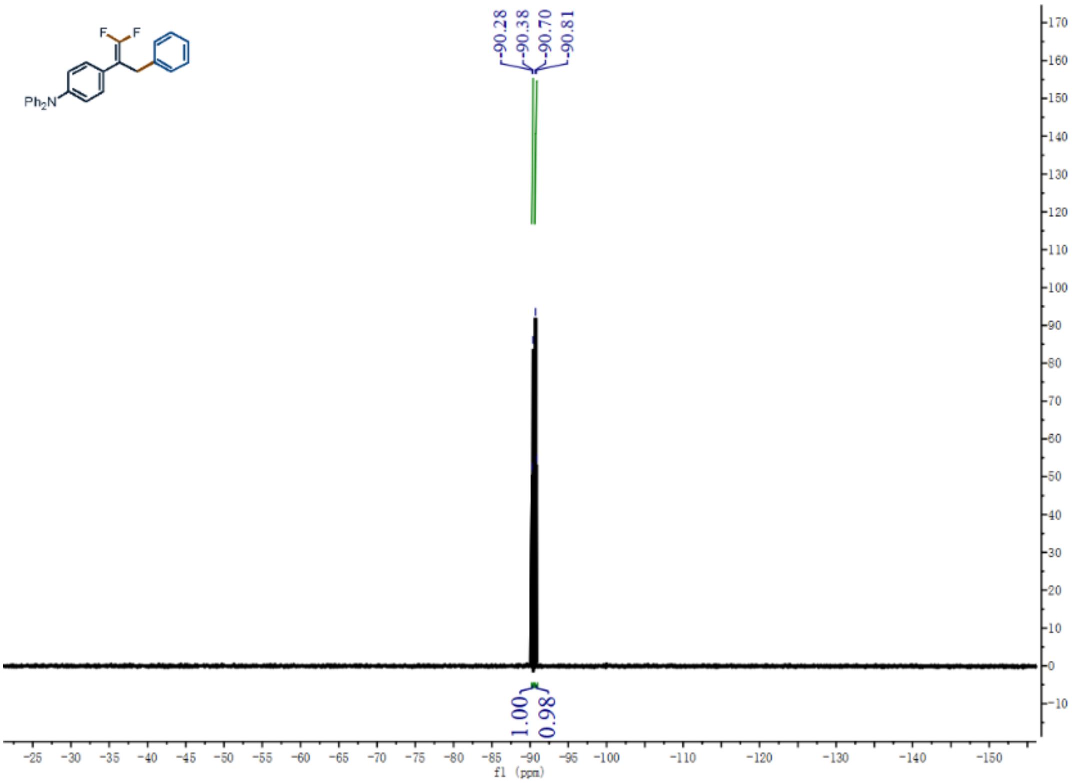

^{1}H NMR (400 MHz, $CDCl_3$) spectrum of compound **9h**

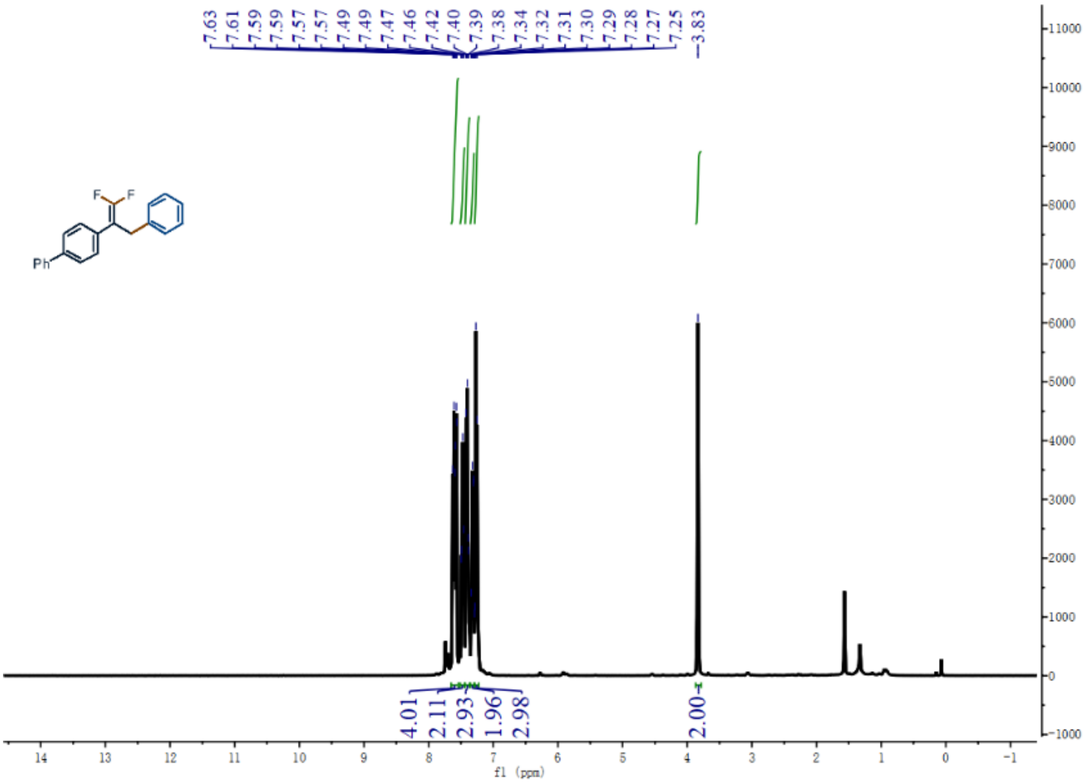

^{13}C NMR (100 MHz, $CDCl_3$) spectrum of compound **9h**

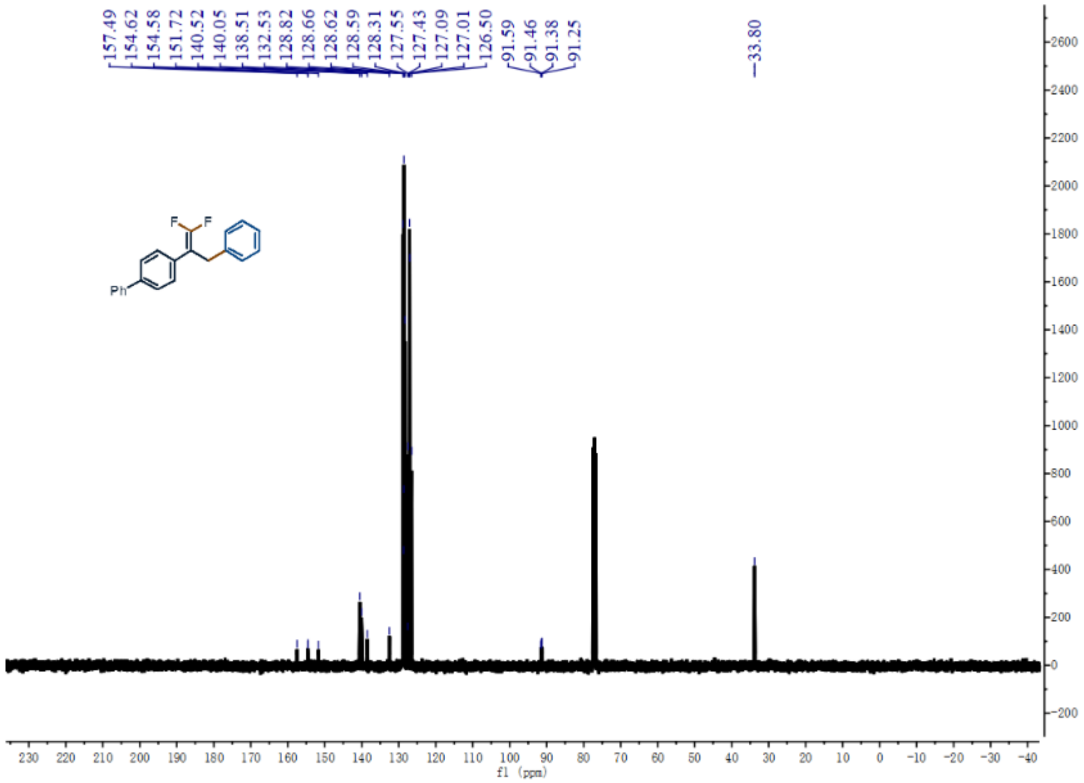

^{19}F NMR (376 MHz, CDCl$_3$) spectrum of compound **9h**

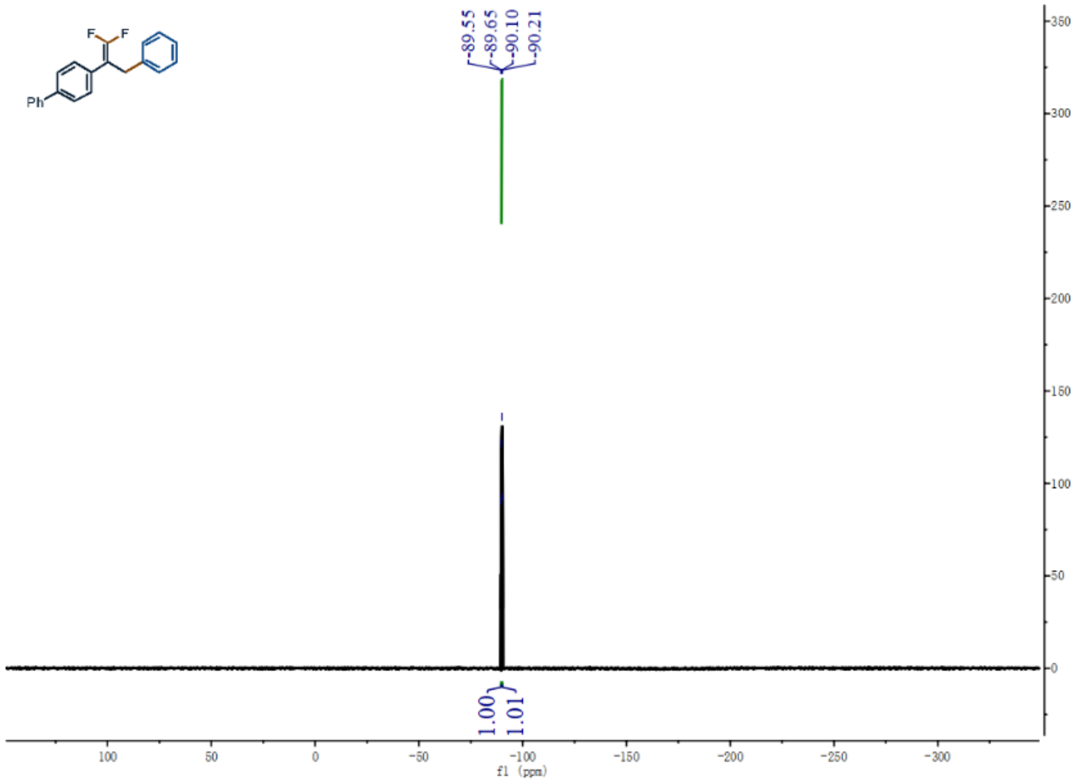

^{1}H NMR (400 MHz, CDCl$_3$) spectrum of compound **9i**

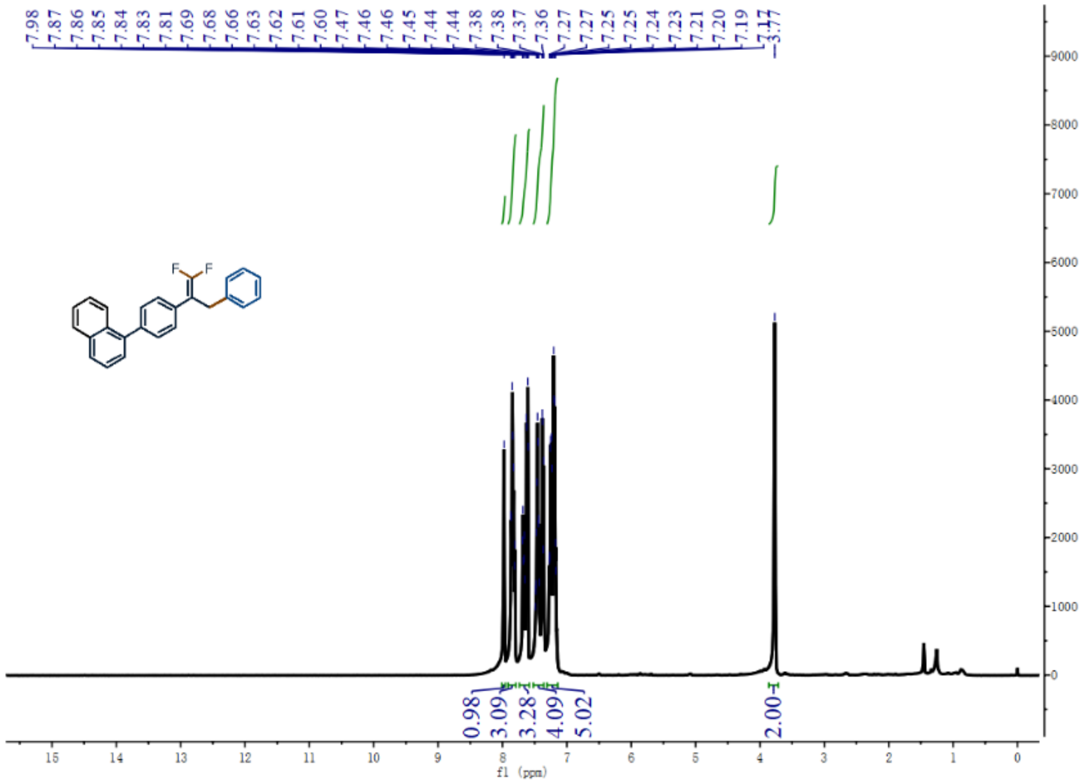

^{13}C NMR (100 MHz, $CDCl_3$) spectrum of compound **9i**

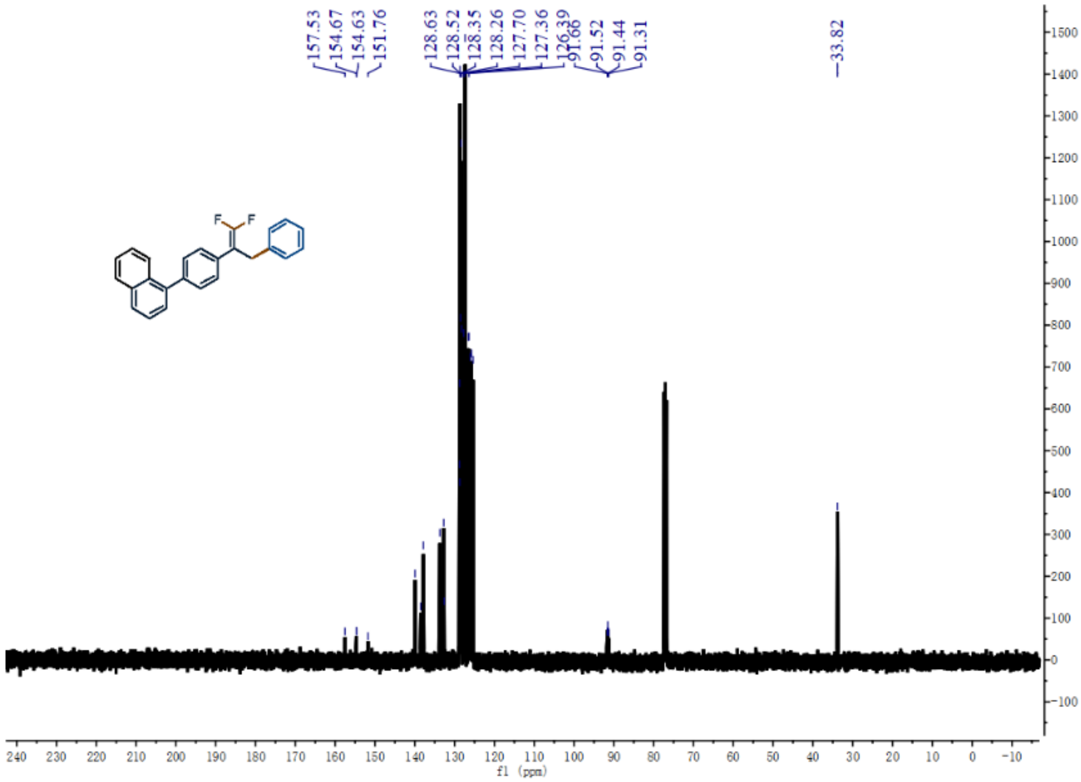

^{19}F NMR (376 MHz, $CDCl_3$) spectrum of compound **9i**

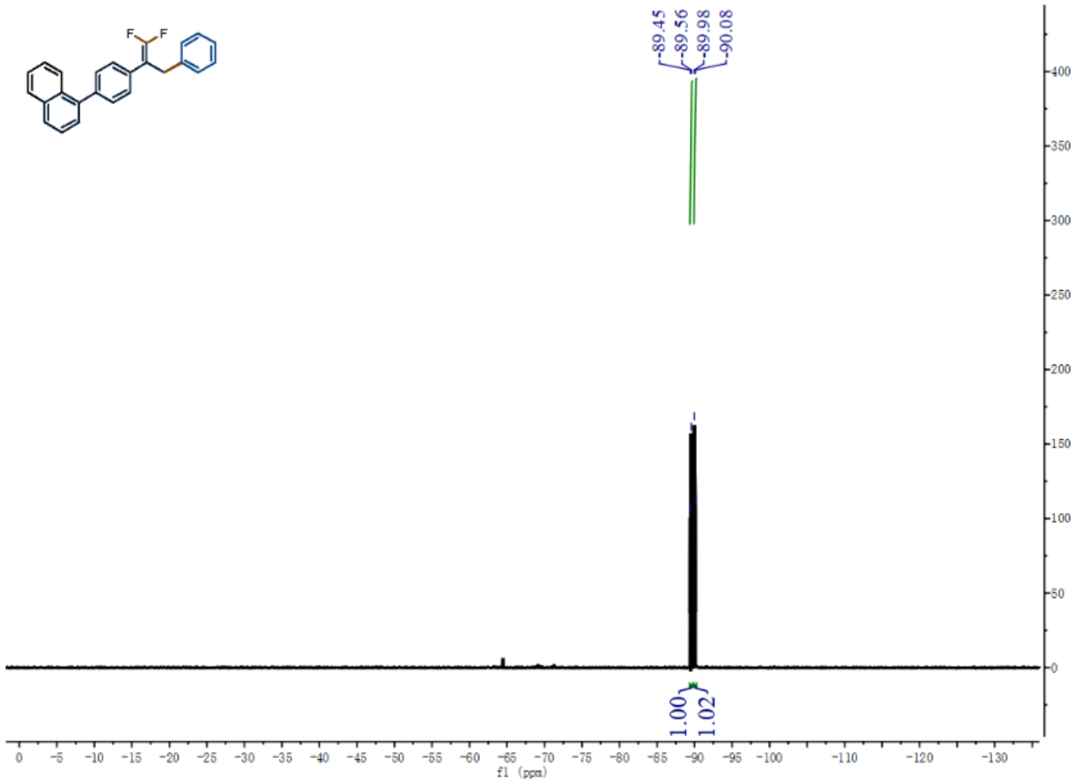

^{1}H NMR (400 MHz, CDCl$_3$) spectrum of compound **9j**

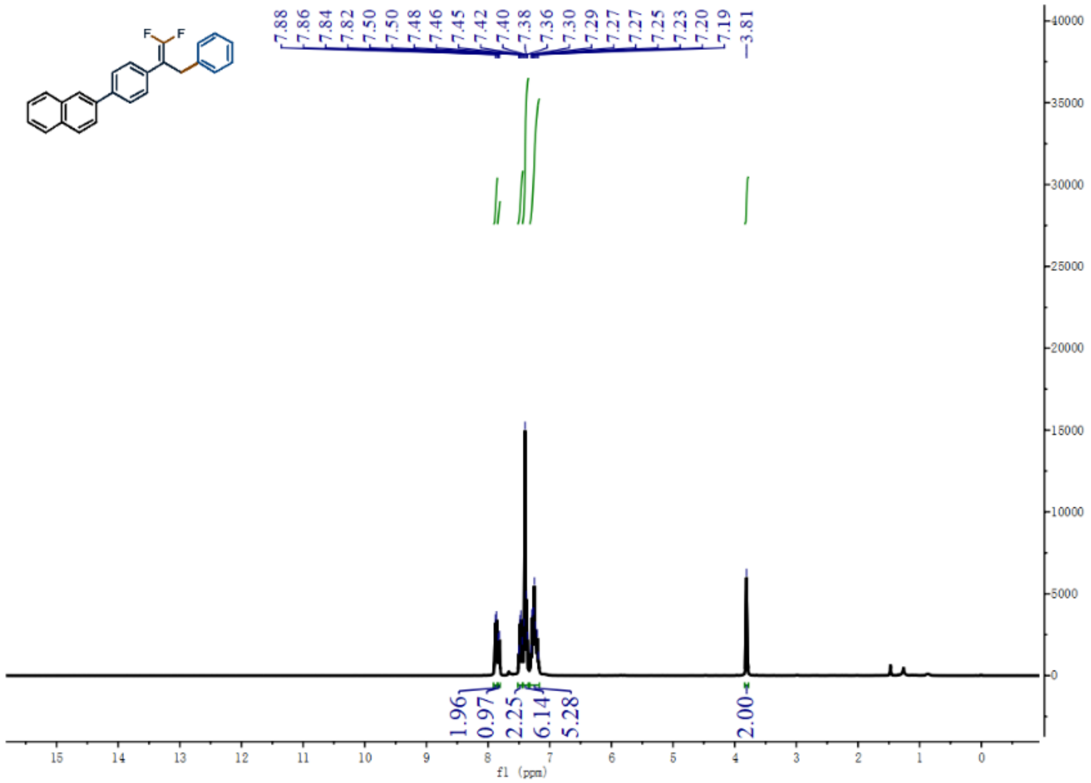

^{13}C NMR (100 MHz, CDCl$_3$) spectrum of compound **9j**

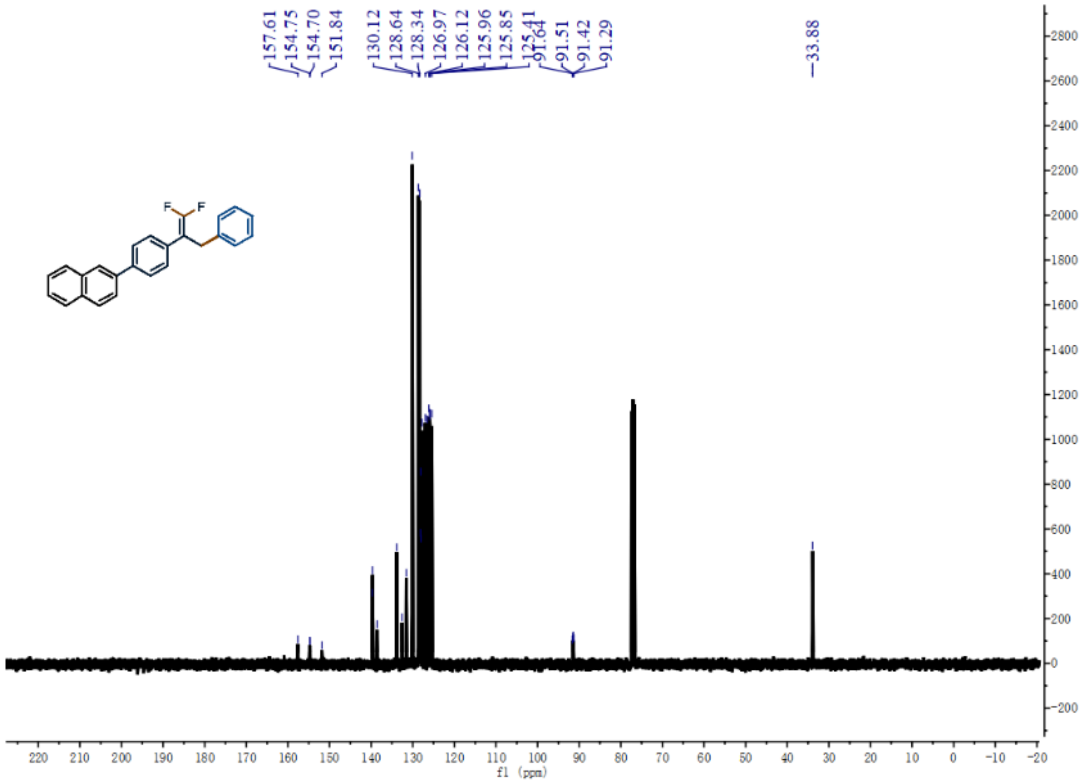

^{19}F NMR (376 MHz, $CDCl_3$) spectrum of compound **9j**

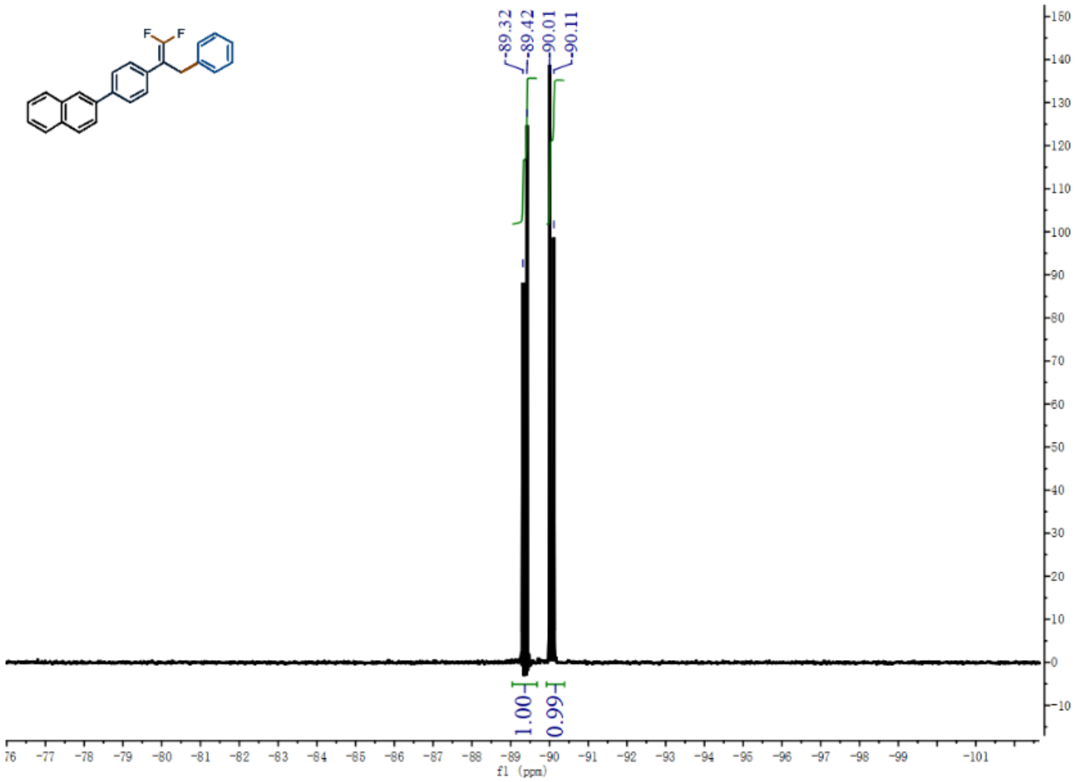

^{1}H NMR (400 MHz, $CDCl_3$) spectrum of compound **9k**

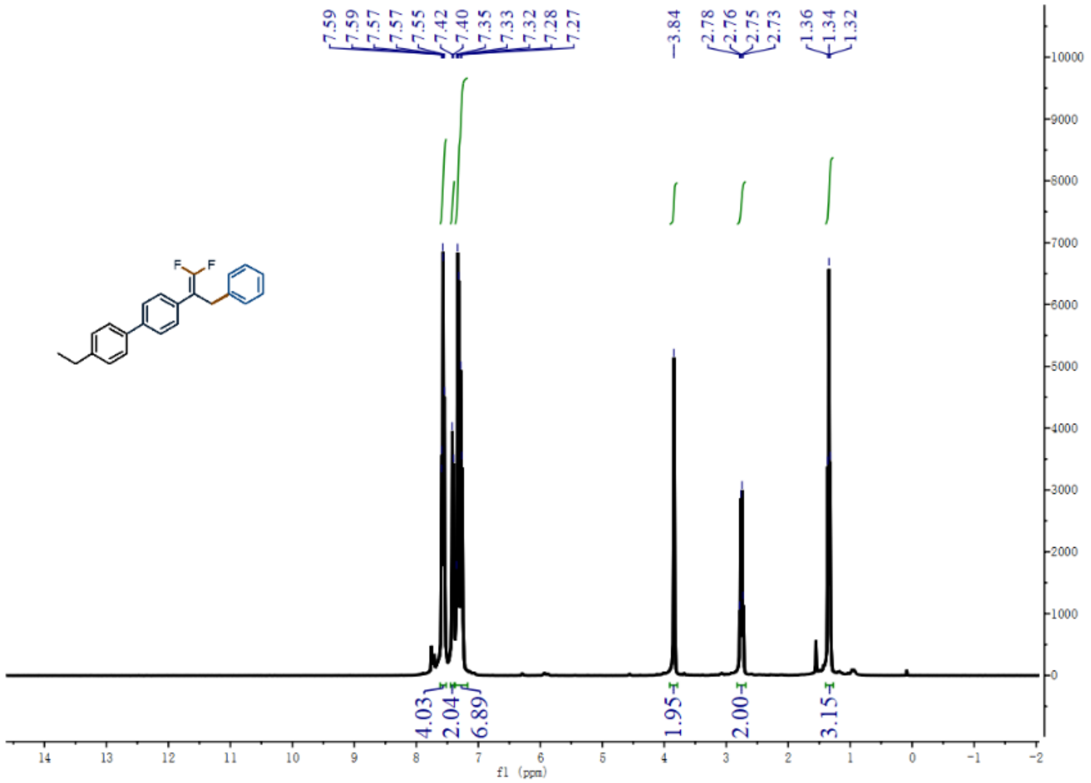

^{13}C NMR (100 MHz, $CDCl_3$) spectrum of compound **9k**

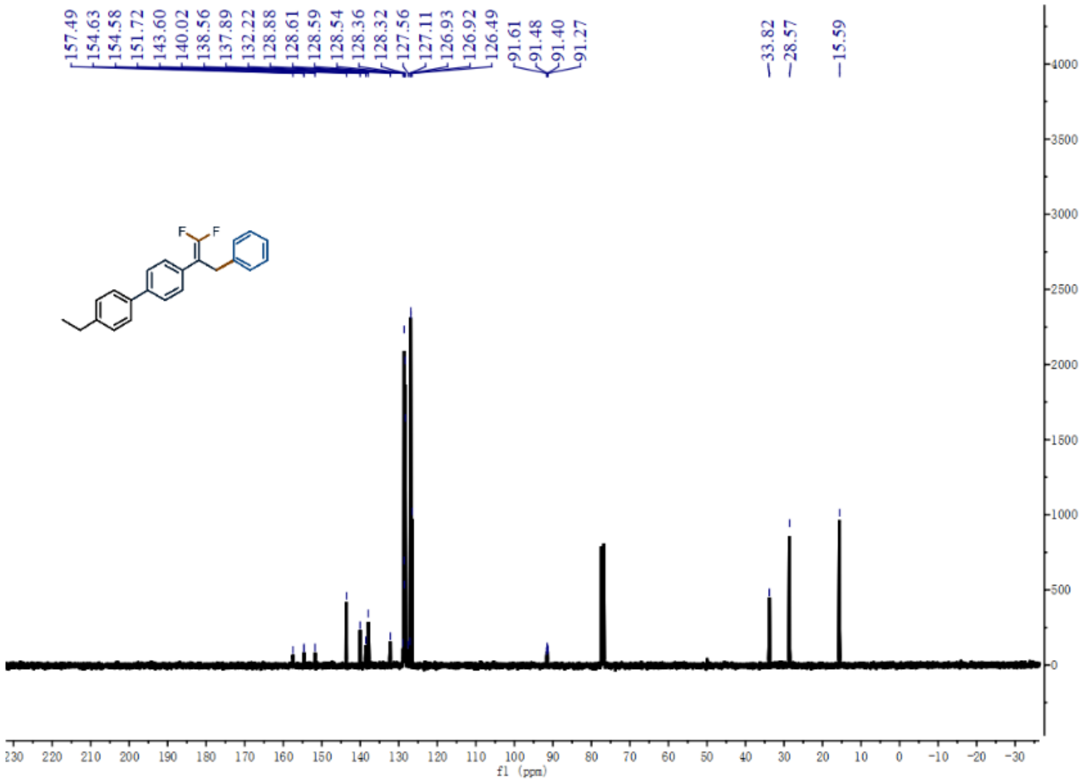

^{19}F NMR (376 MHz, $CDCl_3$) spectrum of compound **9k**

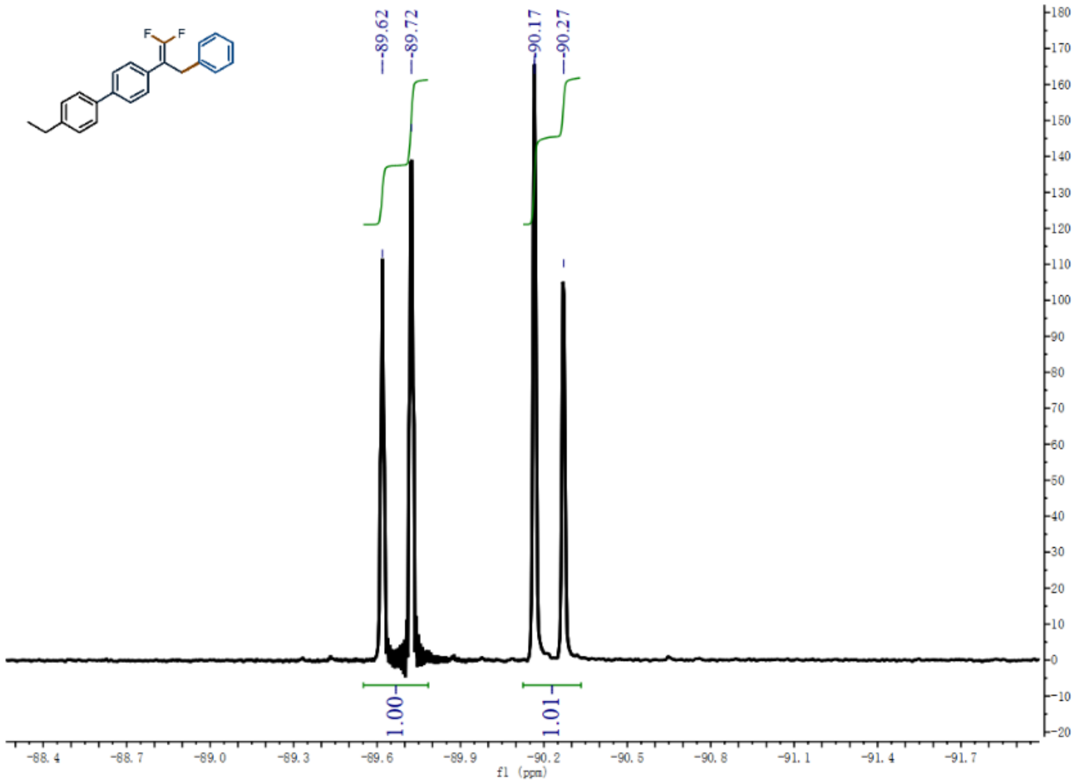

^{1}H NMR (400 MHz, $CDCl_3$) spectrum of compound **9l**

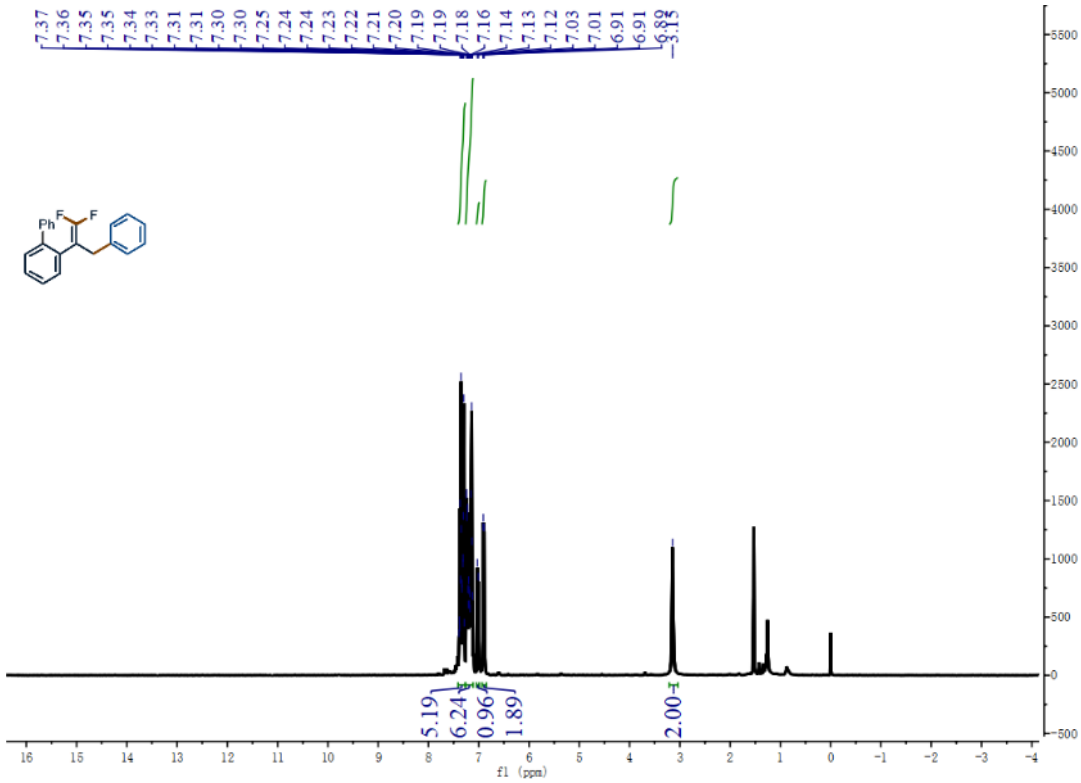

^{13}C NMR (100 MHz, $CDCl_3$) spectrum of compound **9l**

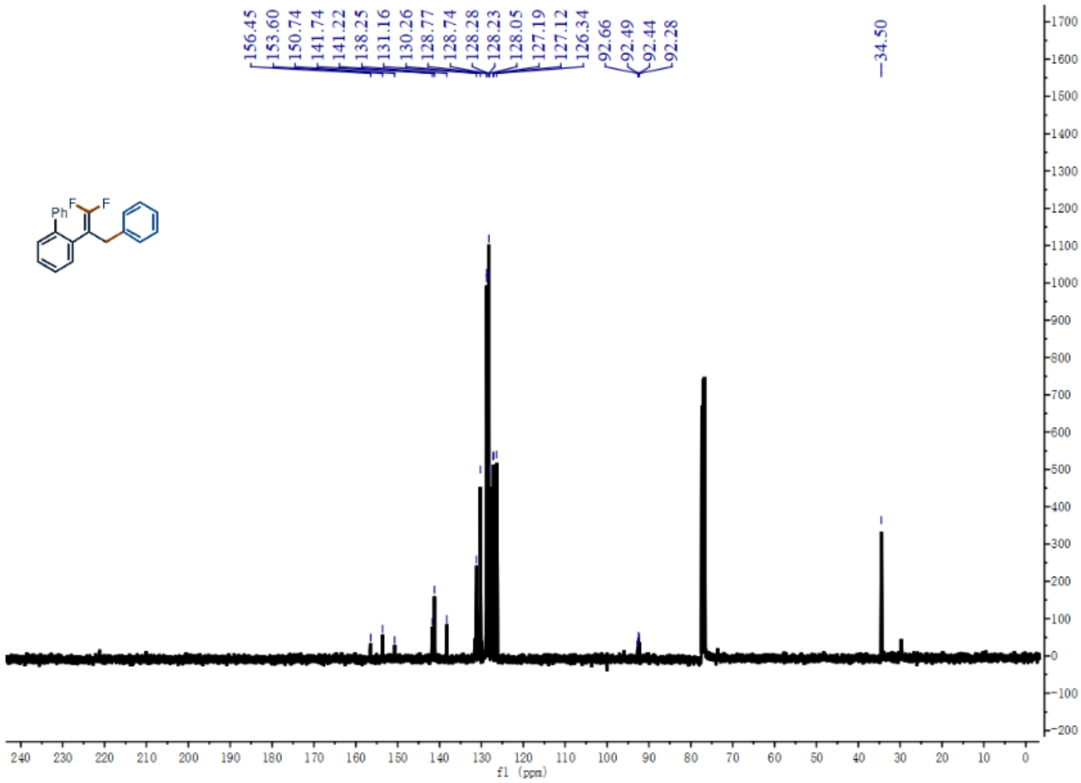

^{19}F NMR (376 MHz, $CDCl_3$) spectrum of compound **9l**

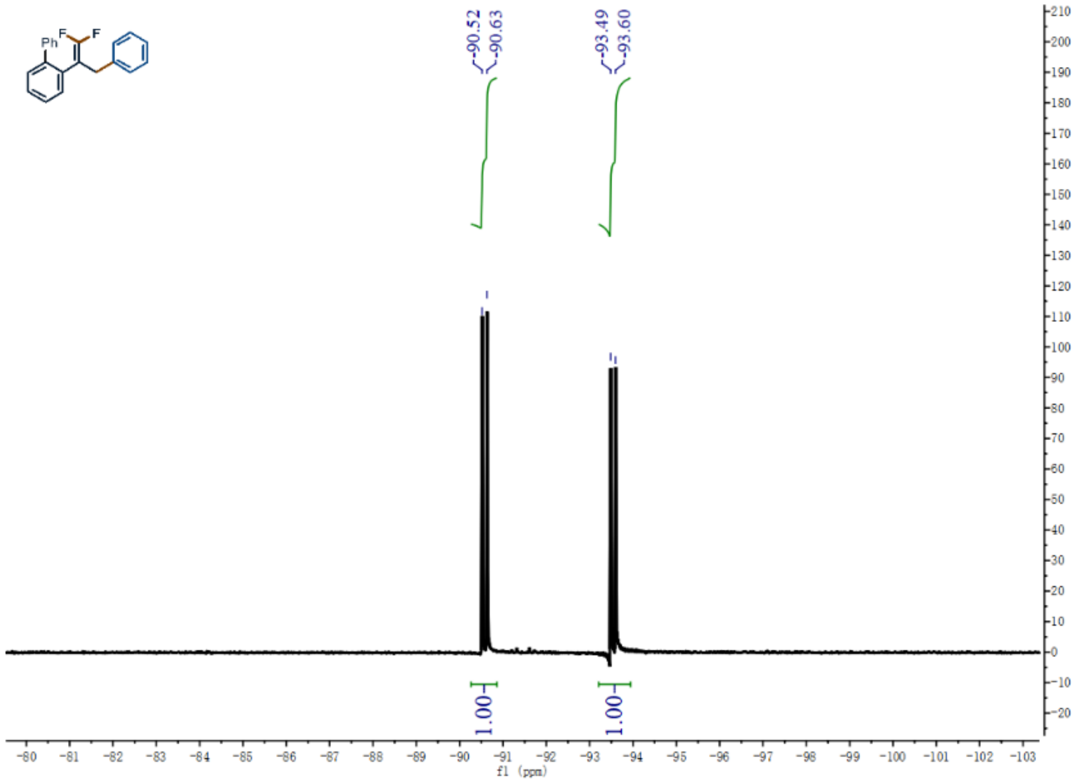

^{1}H NMR (400 MHz, $CDCl_3$) spectrum of compound **9m**

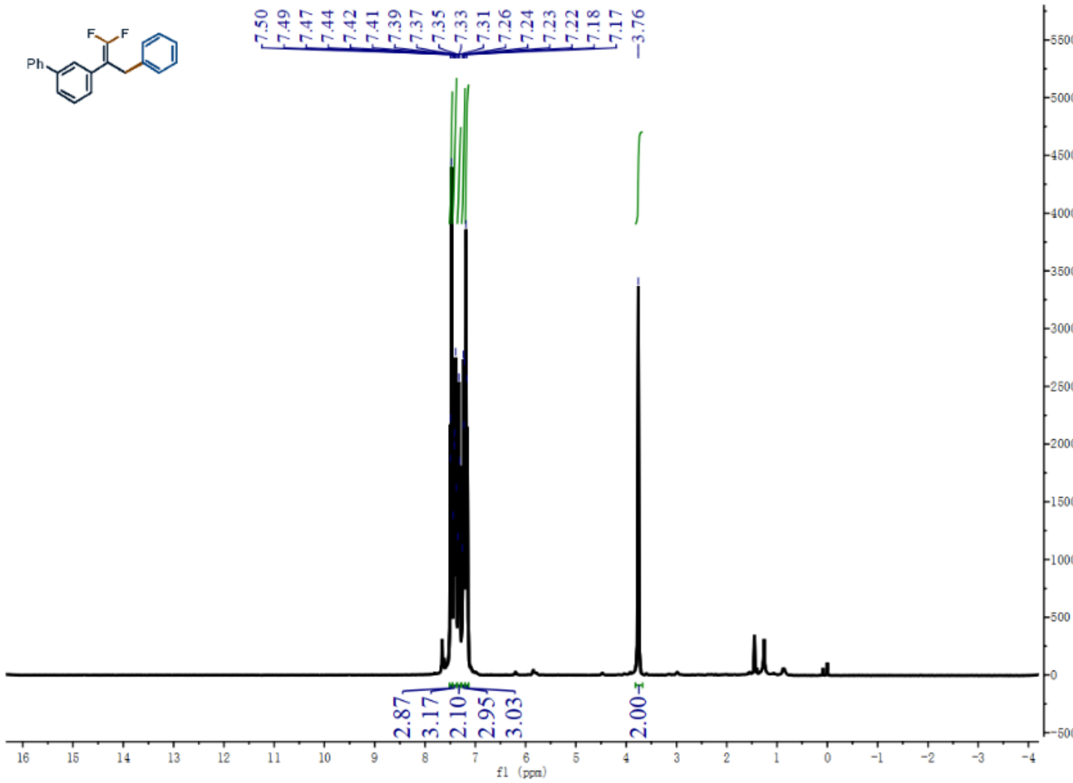

^{13}C NMR (100 MHz, $CDCl_3$) spectrum of compound **9m**

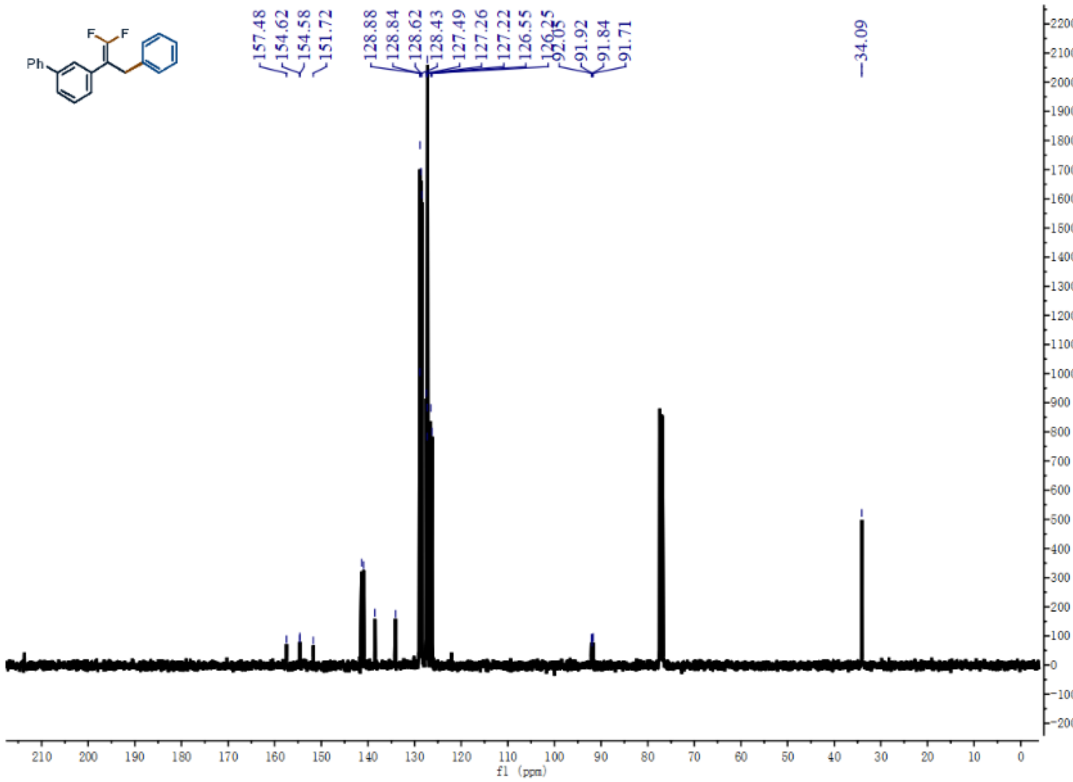

^{19}F NMR (376 MHz, $CDCl_3$) spectrum of compound **9m**

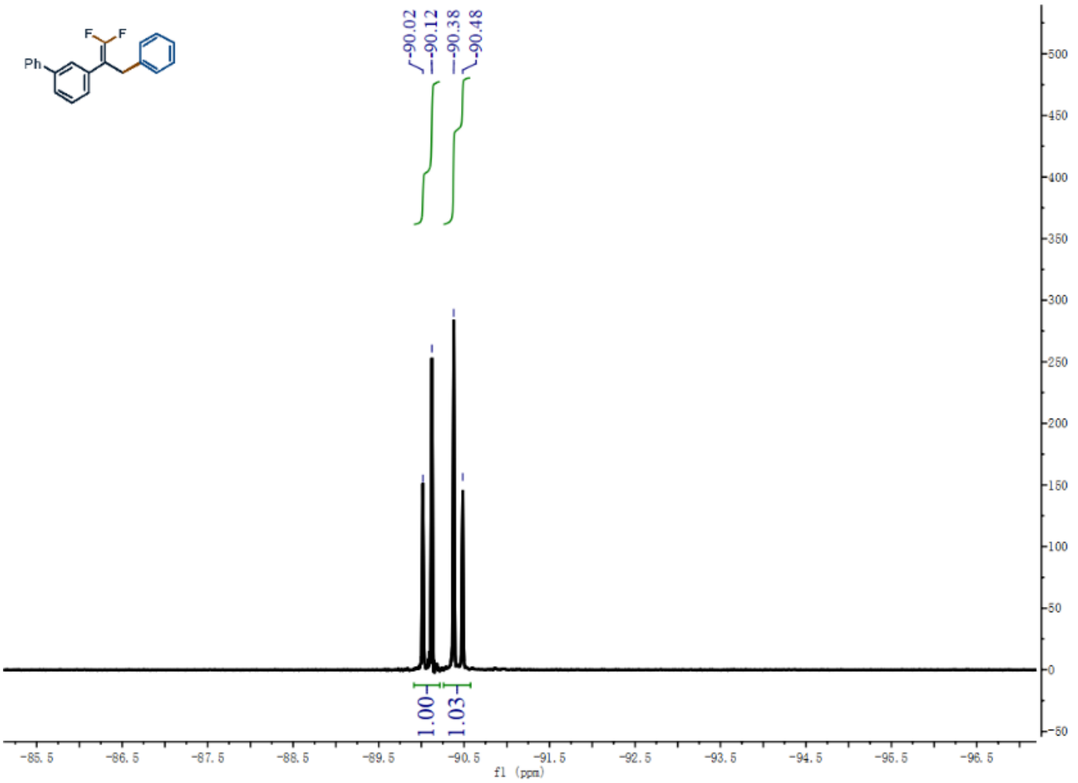

^{1}H NMR (400 MHz, $CDCl_3$) spectrum of compound **9n**

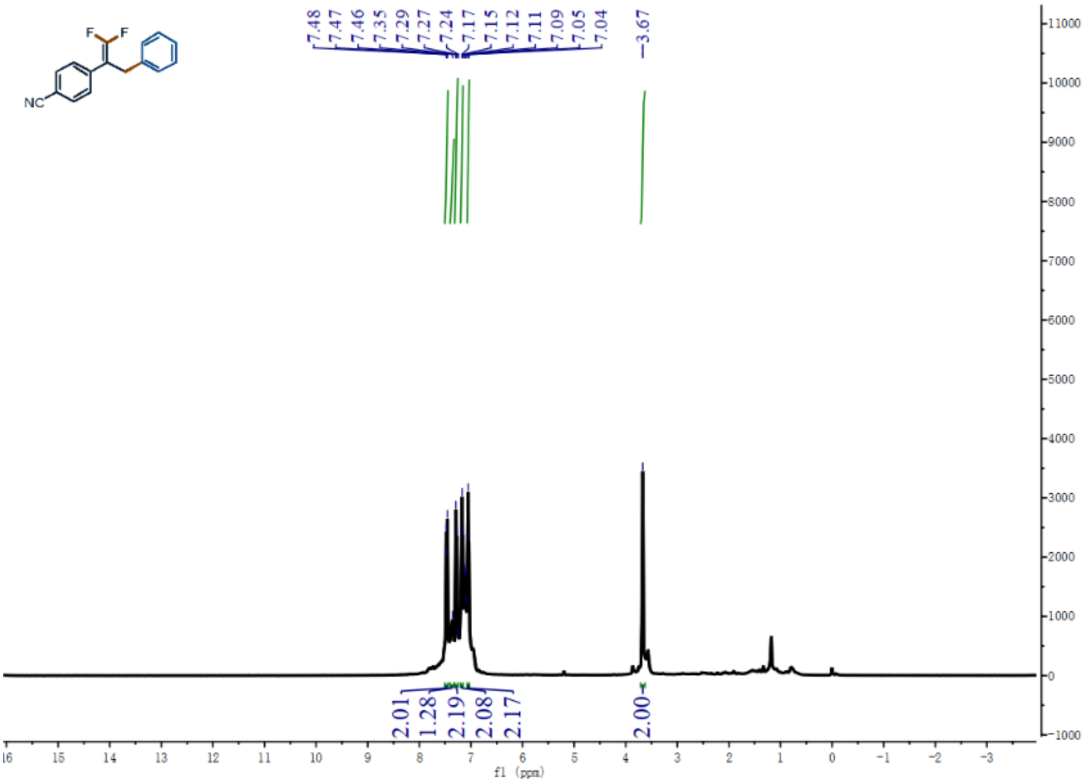

^{13}C NMR (100 MHz, $CDCl_3$) spectrum of compound **9n**

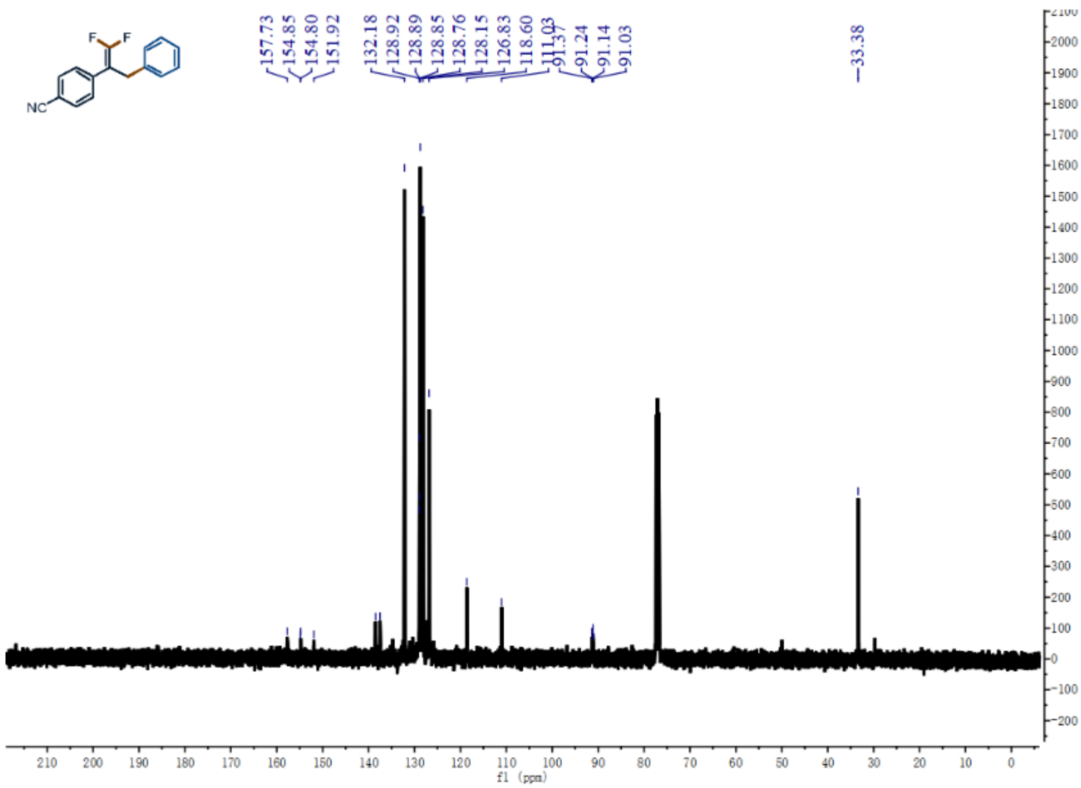

^{19}F NMR (376 MHz, $CDCl_3$) spectrum of compound **9n**

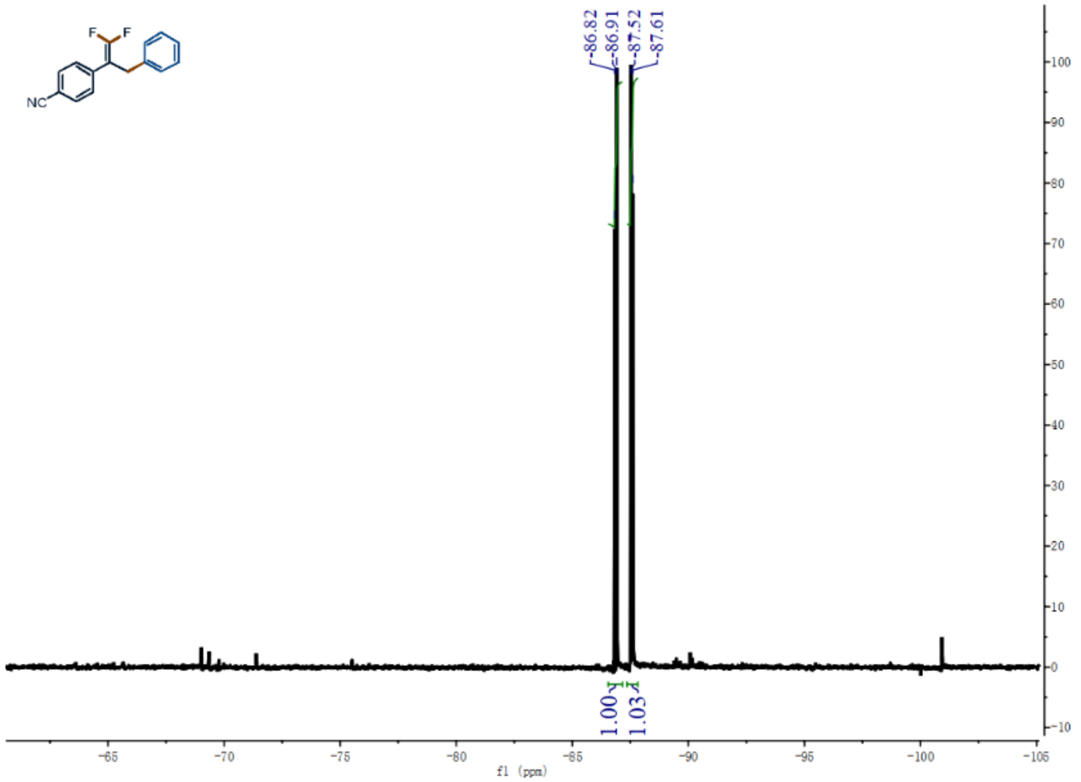

^{1}H NMR (400 MHz, $CDCl_3$) spectrum of compound **9o**

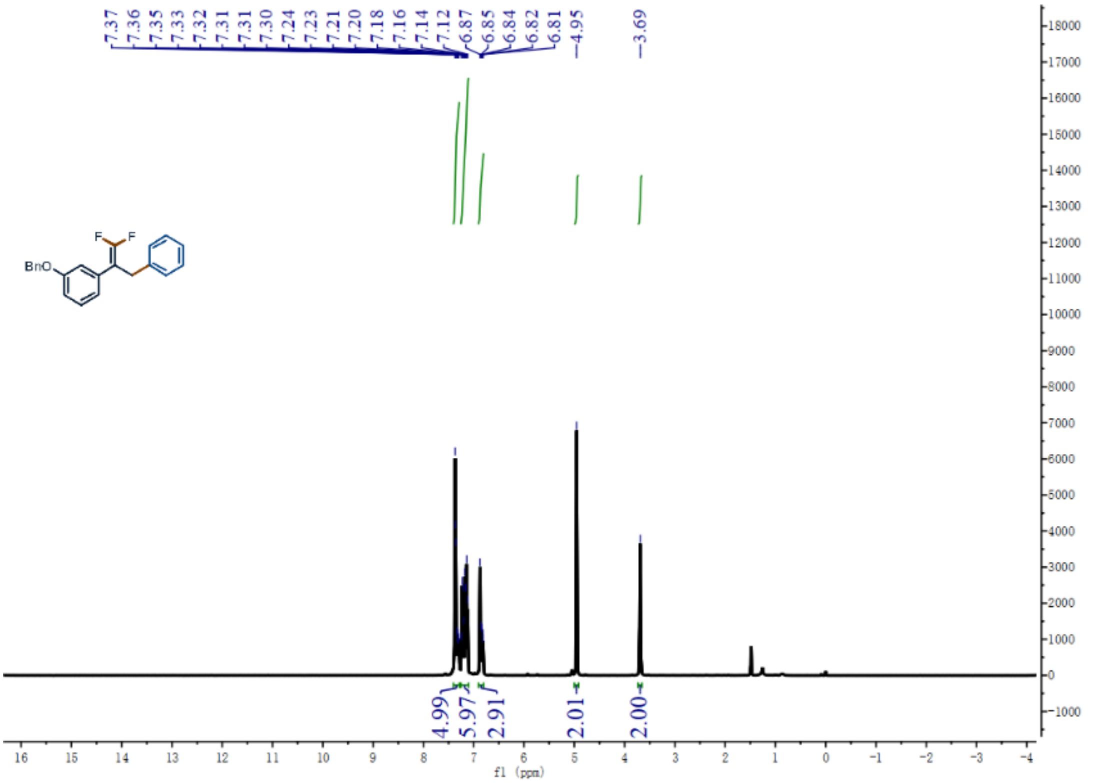

^{13}C NMR (100 MHz, $CDCl_3$) spectrum of compound **9o**

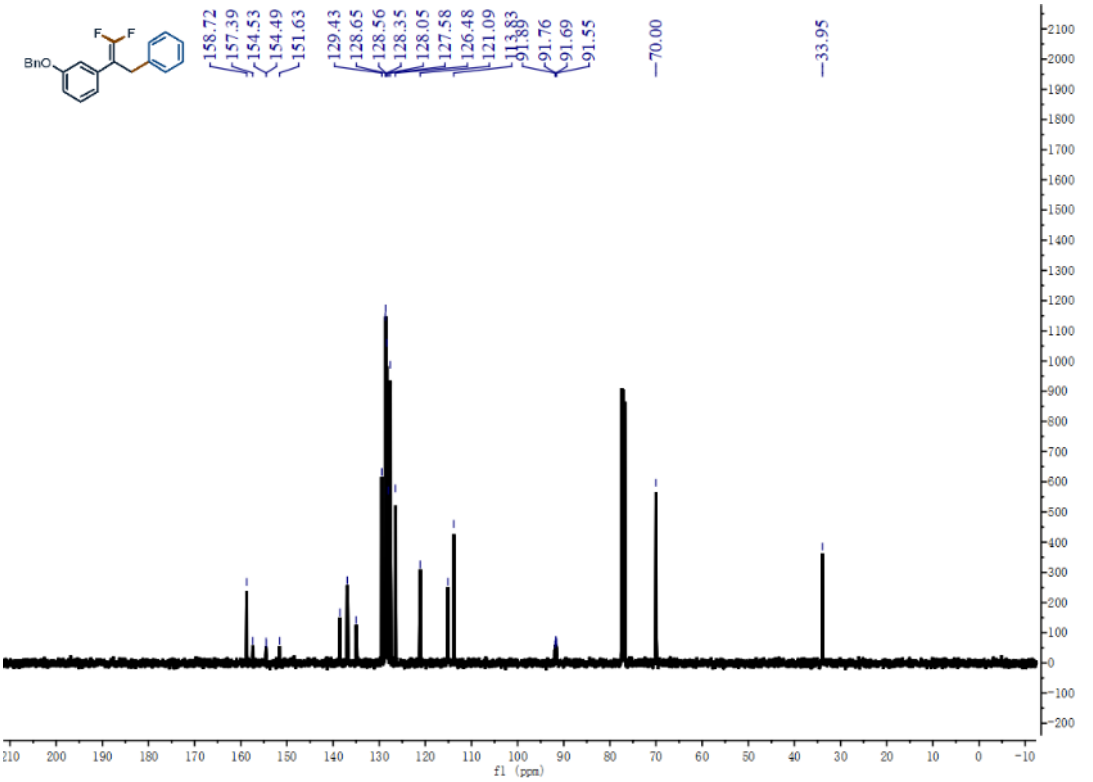

^{19}F NMR (376 MHz, $CDCl_3$) spectrum of compound **9o**

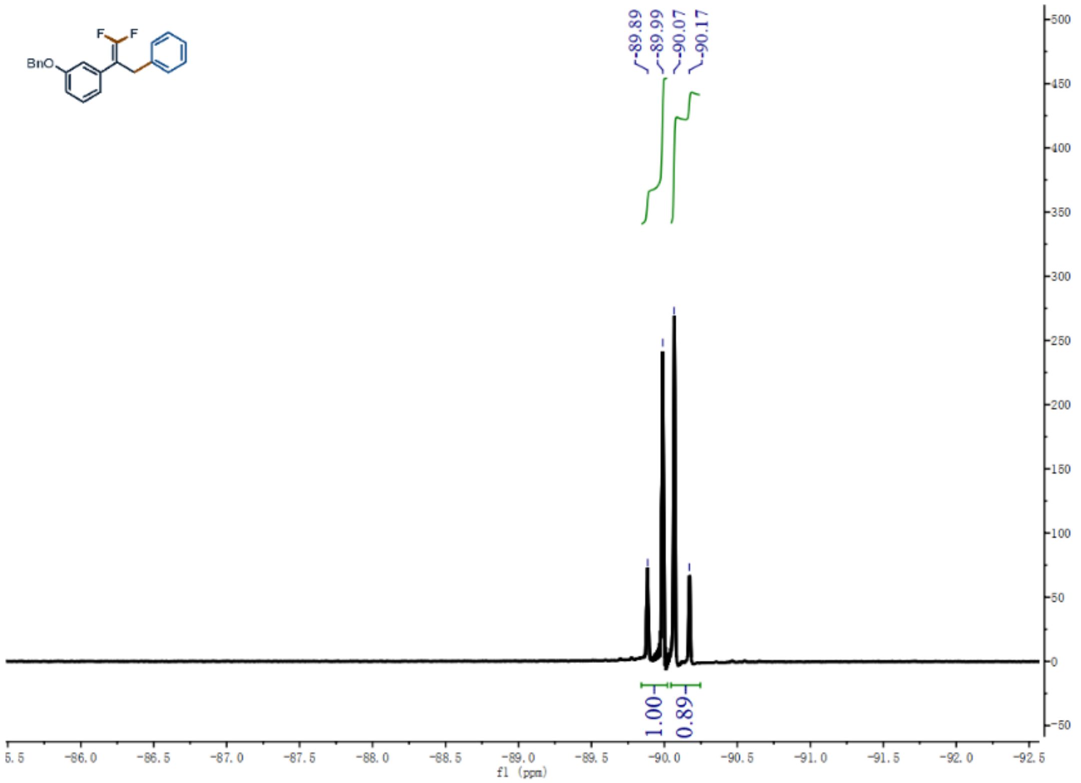

^{1}H NMR (400 MHz, $CDCl_3$) spectrum of compound **9p**

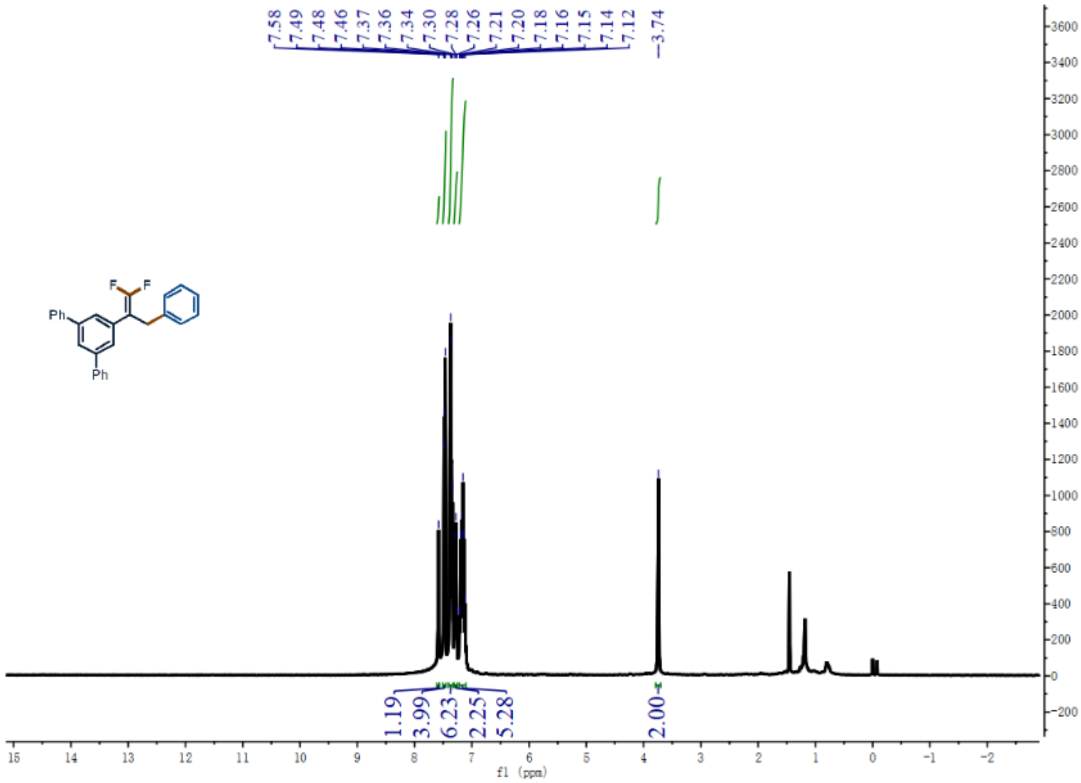

^{13}C NMR (100 MHz, $CDCl_3$) spectrum of compound **9p**

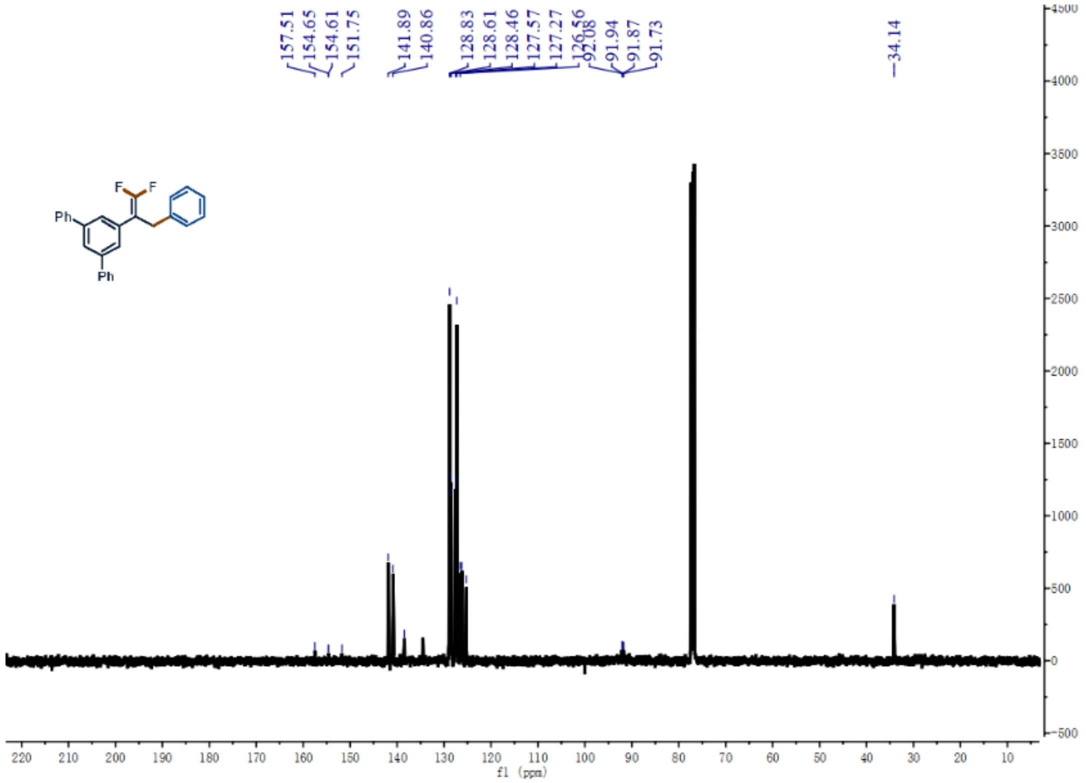

^{19}F NMR (376 MHz, $CDCl_3$) spectrum of compound **9p**

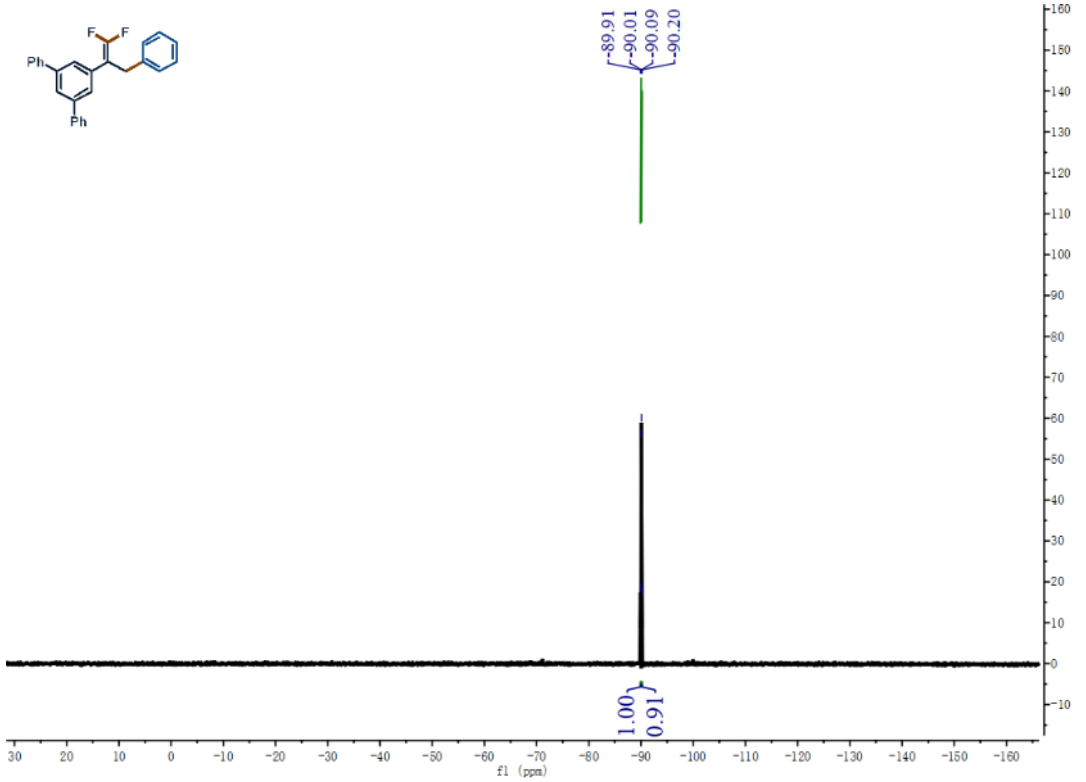

^{1}H NMR (400 MHz, $CDCl_3$) spectrum of compound **9q**

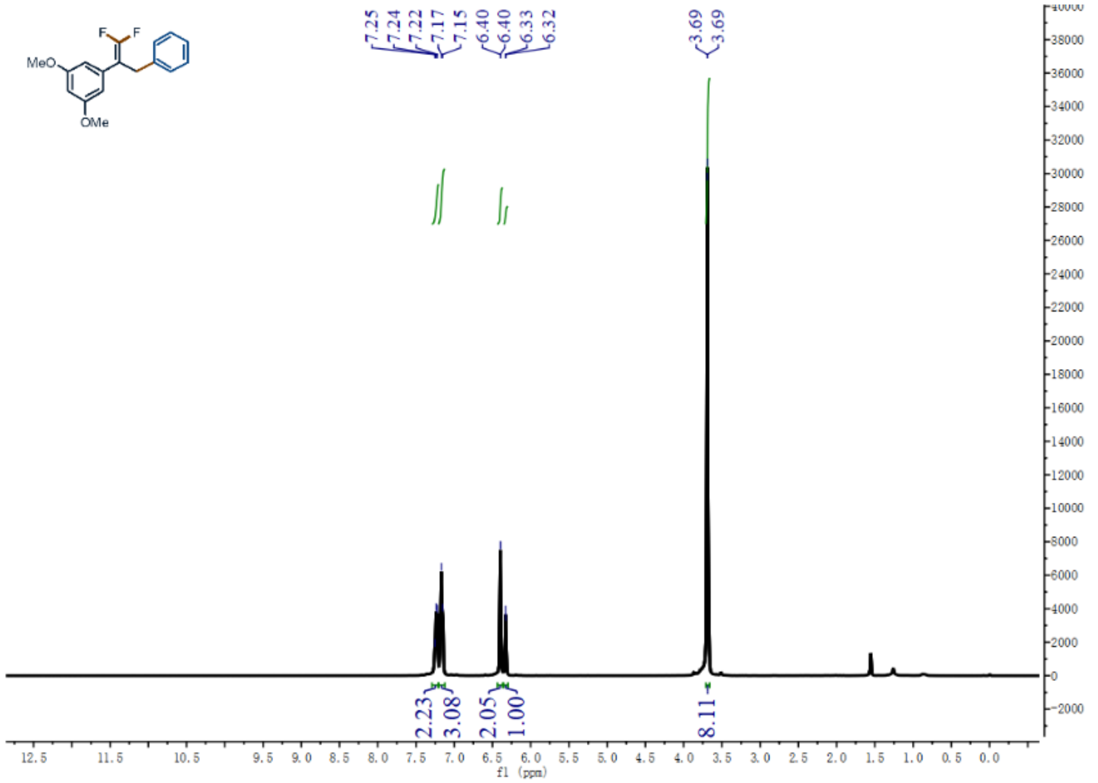

^{13}C NMR (100 MHz, $CDCl_3$) spectrum of compound **9q**

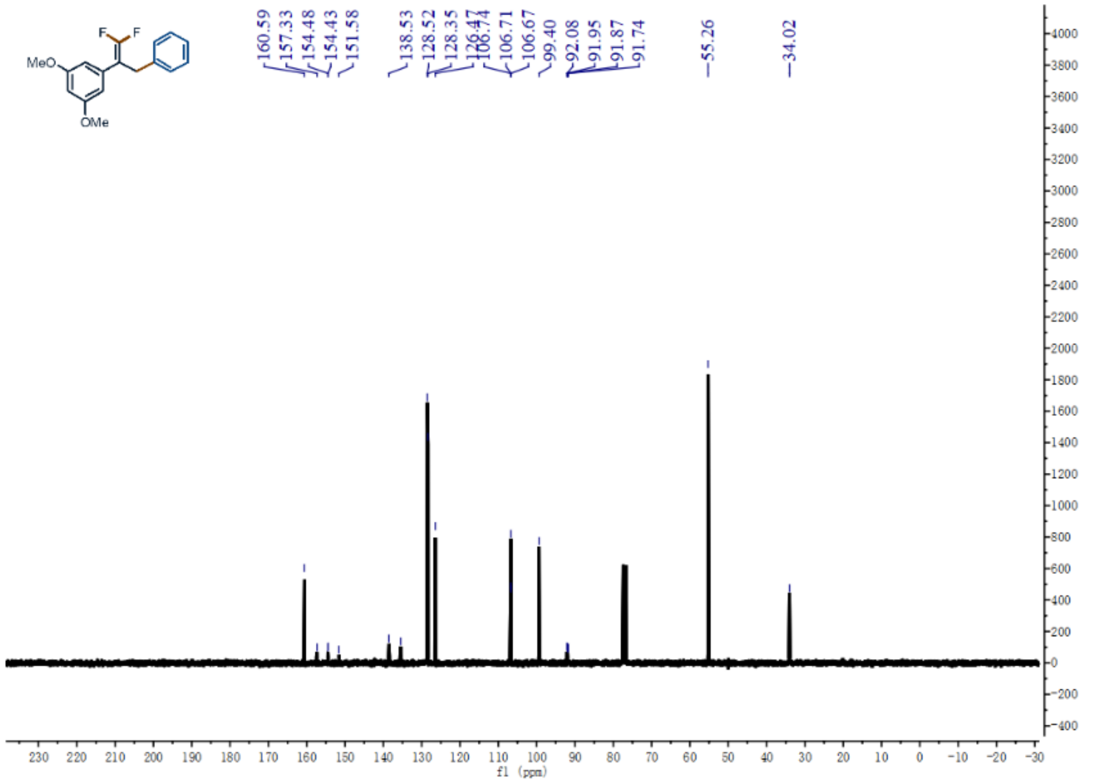

^{19}F NMR (376 MHz, $CDCl_3$) spectrum of compound **9q**

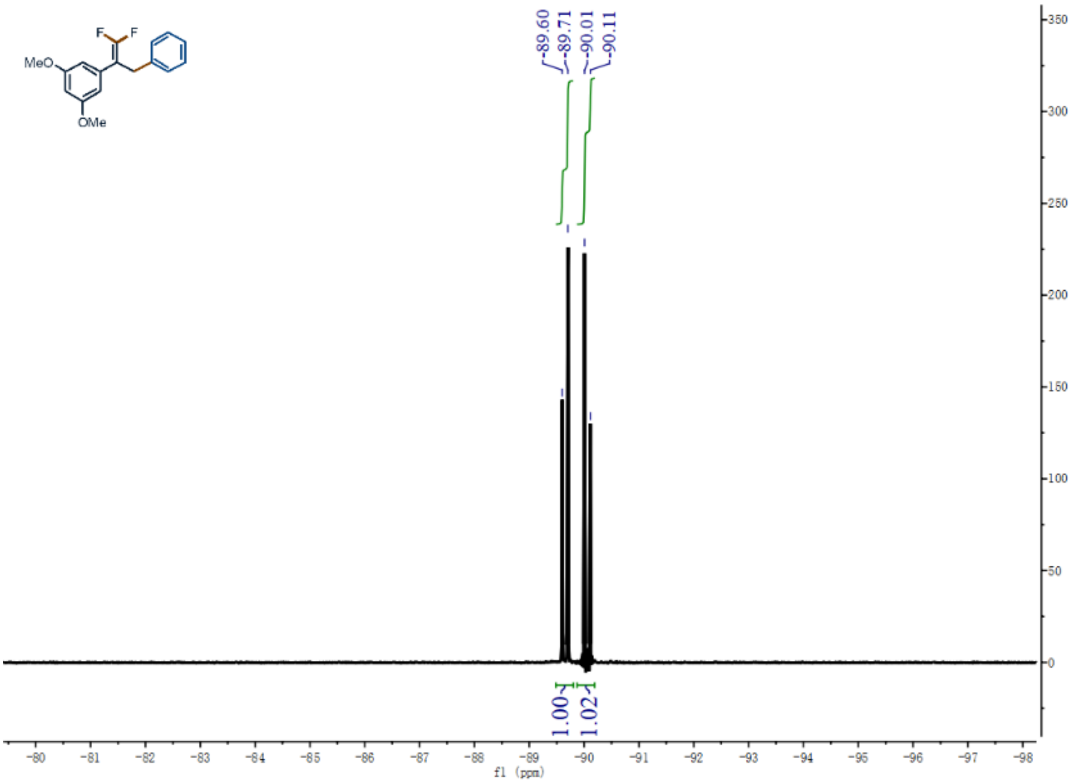

^{1}H NMR (400 MHz, $CDCl_3$) spectrum of compound **9r**

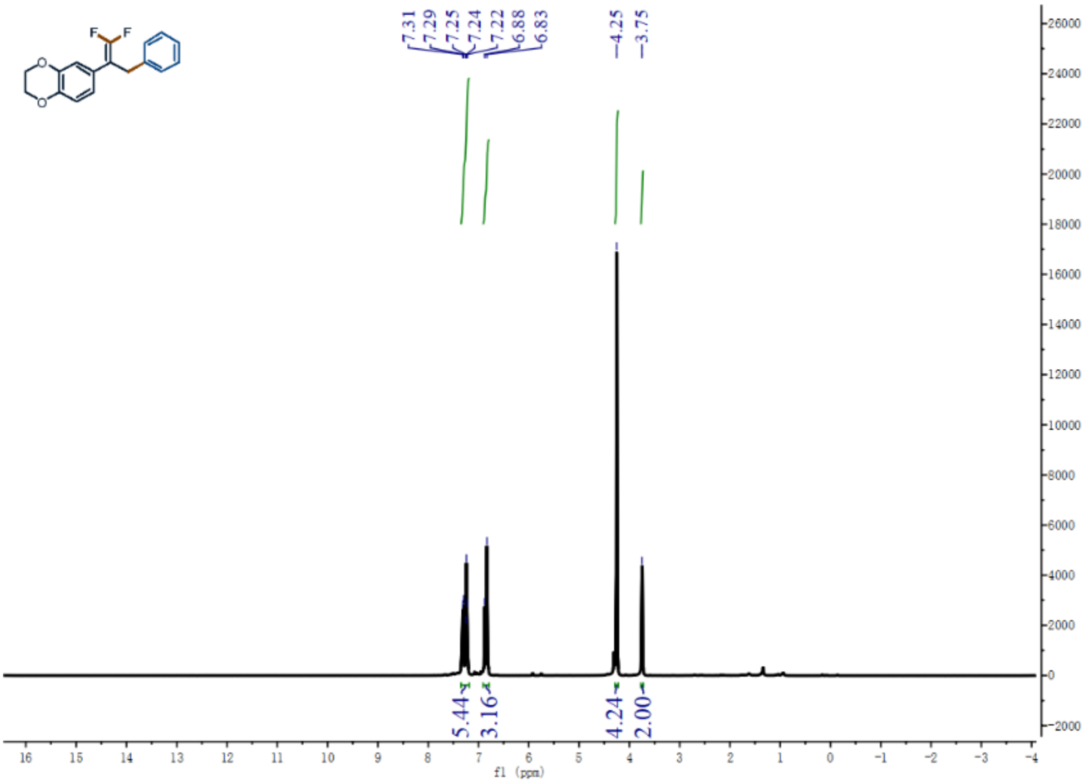

^{13}C NMR (100 MHz, $CDCl_3$) spectrum of compound **9r**

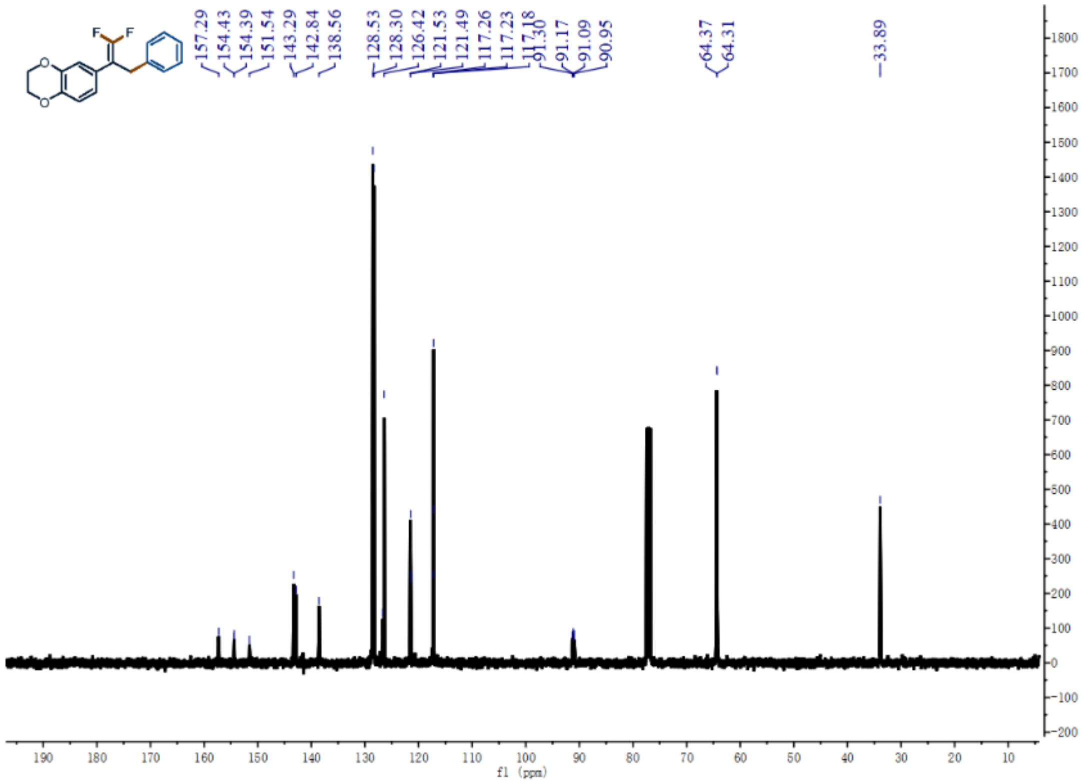

^{19}F NMR (376 MHz, $CDCl_3$) spectrum of compound **9r**

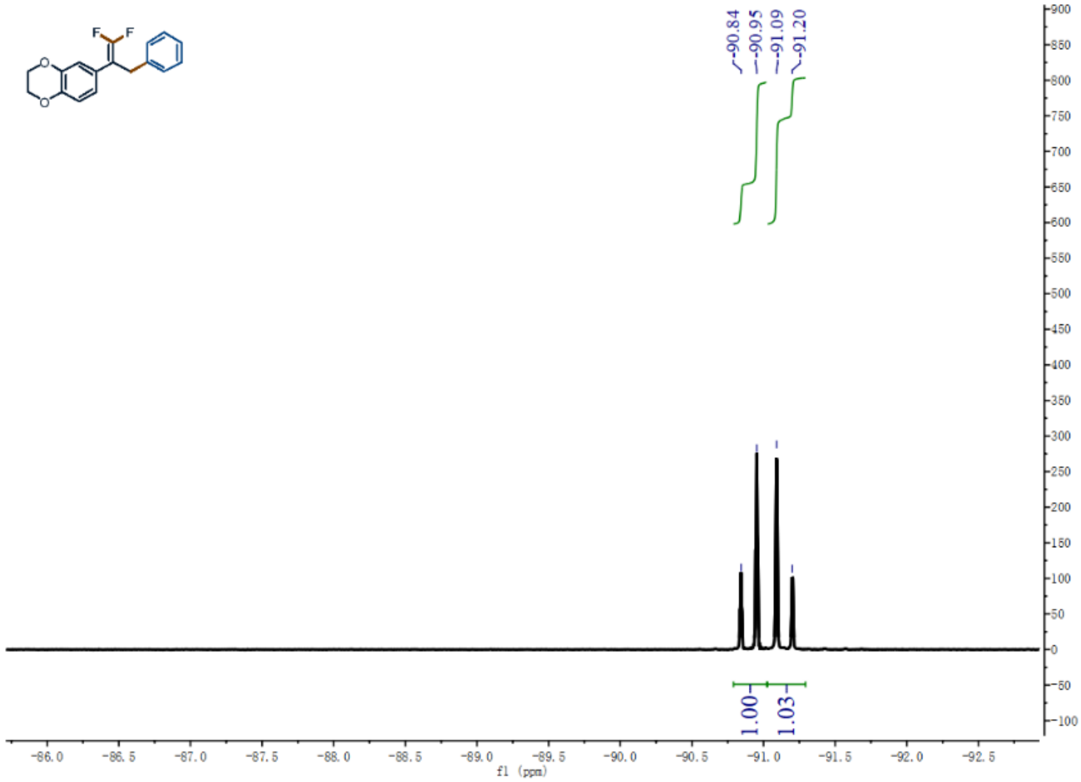

^{1}H NMR (400 MHz, $CDCl_3$) spectrum of compound **9s**

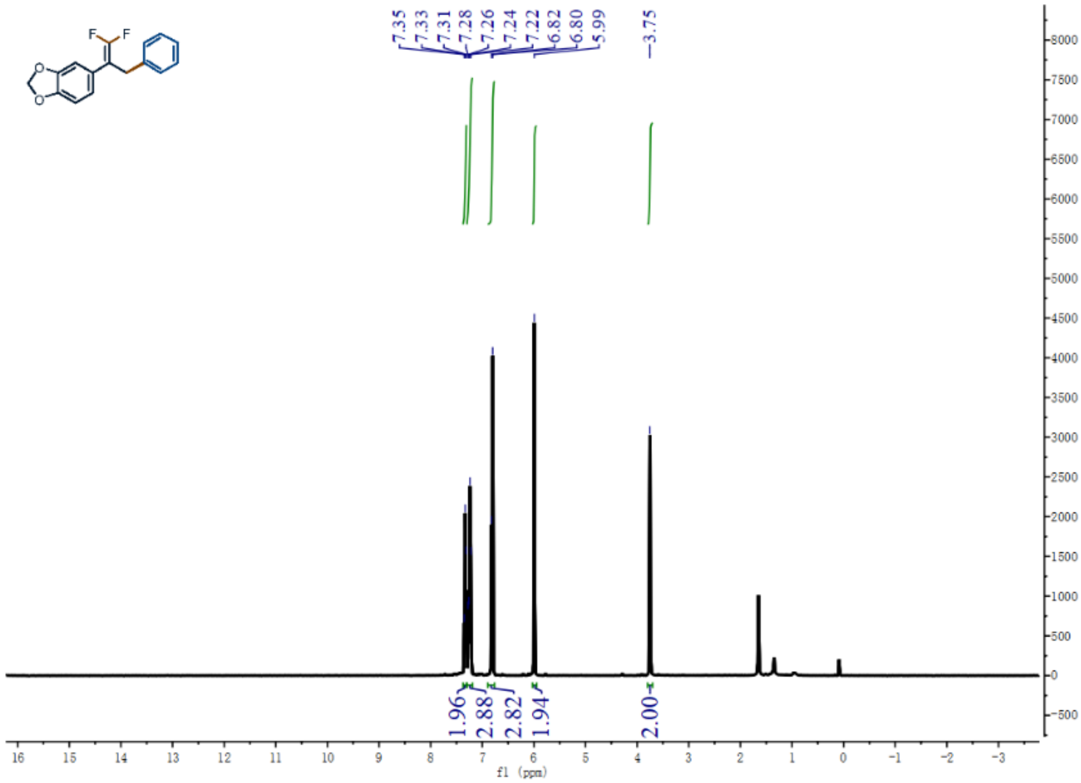

^{13}C NMR (100 MHz, $CDCl_3$) spectrum of compound **9s**

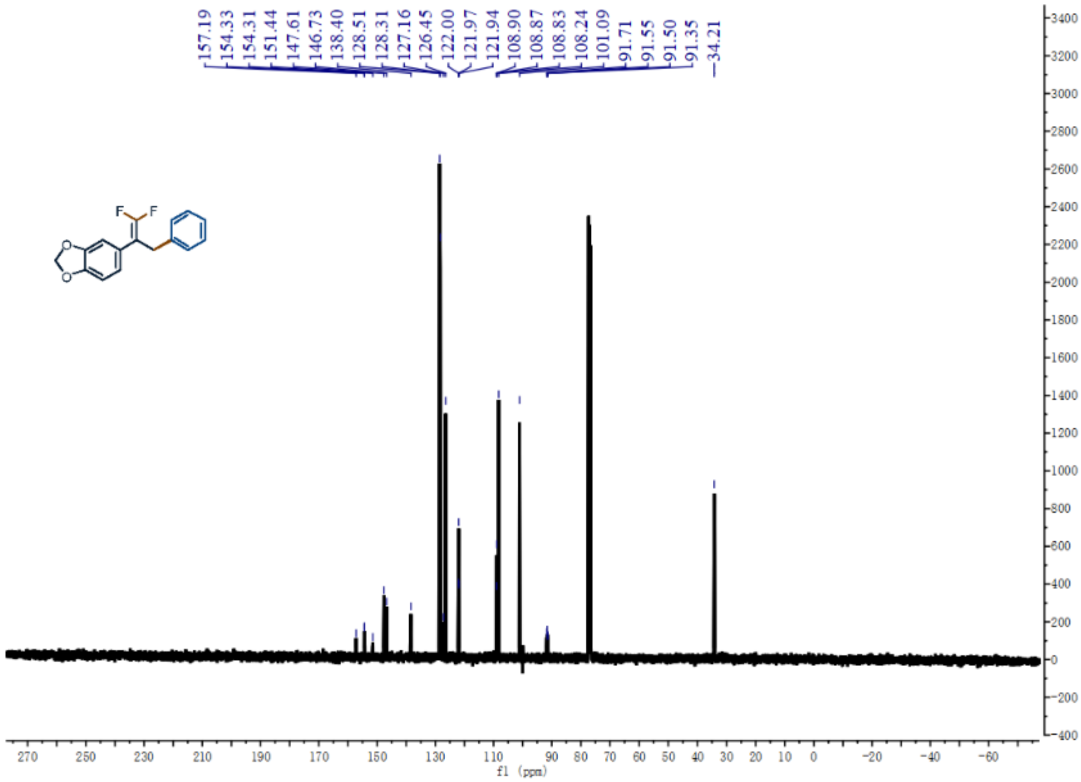

^{19}F NMR (376 MHz, $CDCl_3$) spectrum of compound **9s**

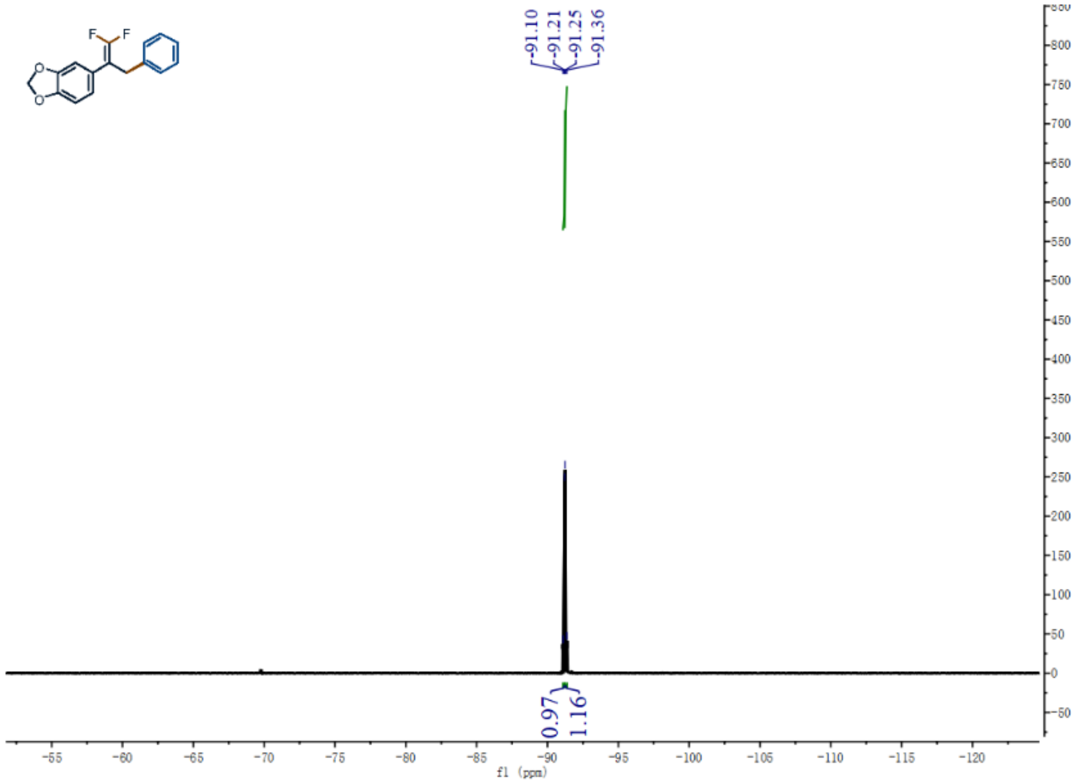

^{1}H NMR (400 MHz, CDCl$_3$) spectrum of compound **9t**

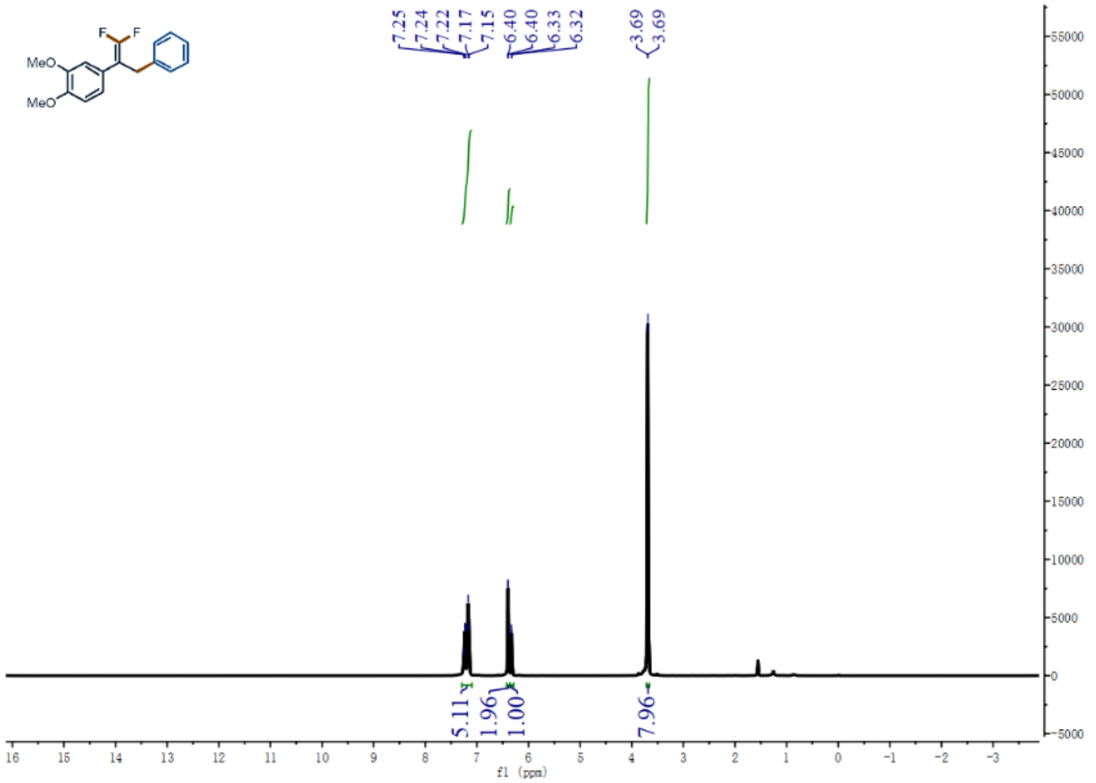

^{13}C NMR (100 MHz, CDCl$_3$) spectrum of compound **9t**

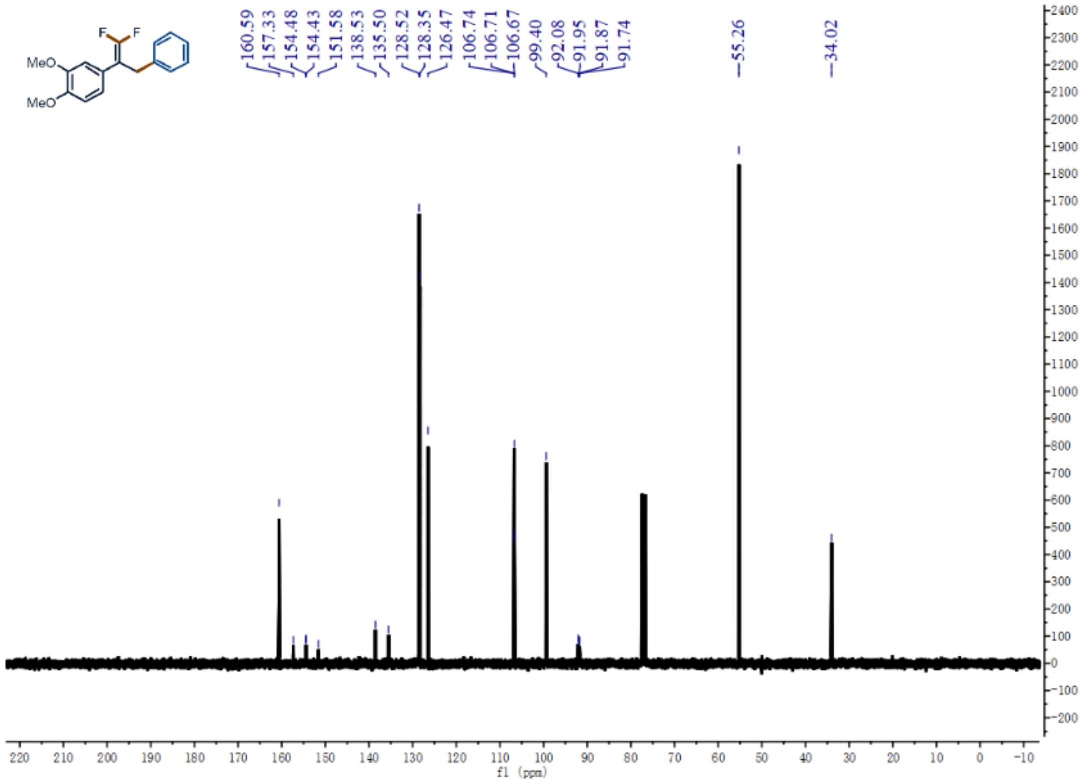

^{19}F NMR (376 MHz, $CDCl_3$) spectrum of compound **9t**

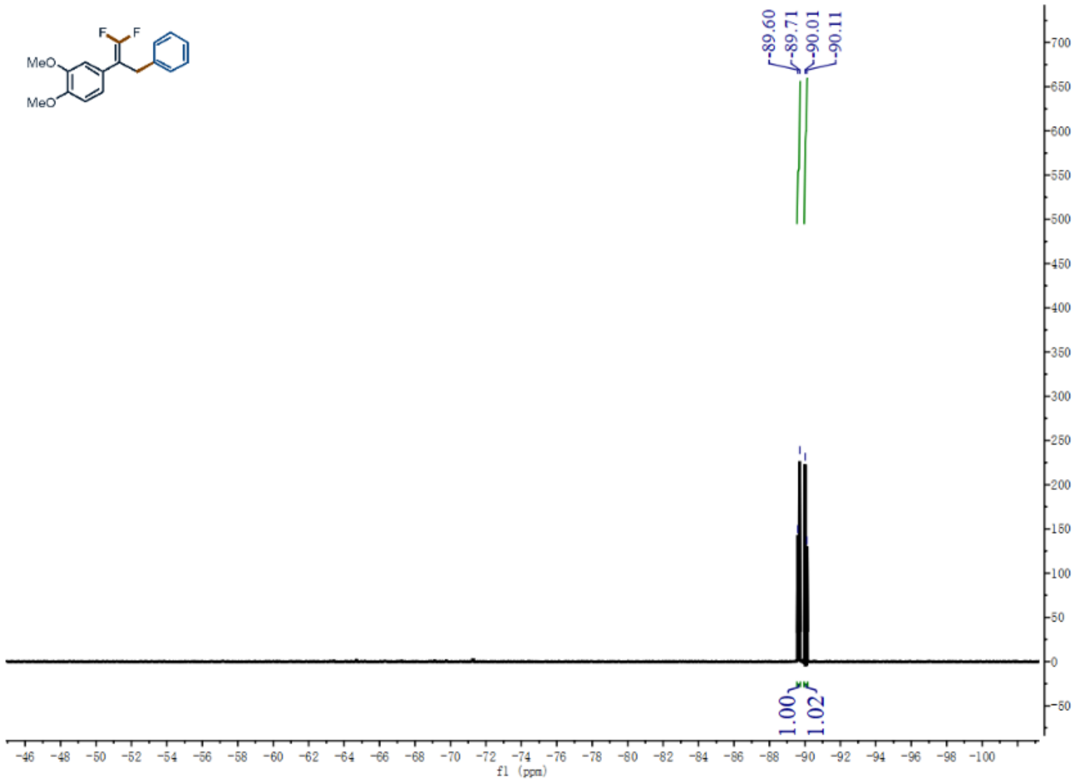

^{1}H NMR (400 MHz, $CDCl_3$) spectrum of compound **9u**

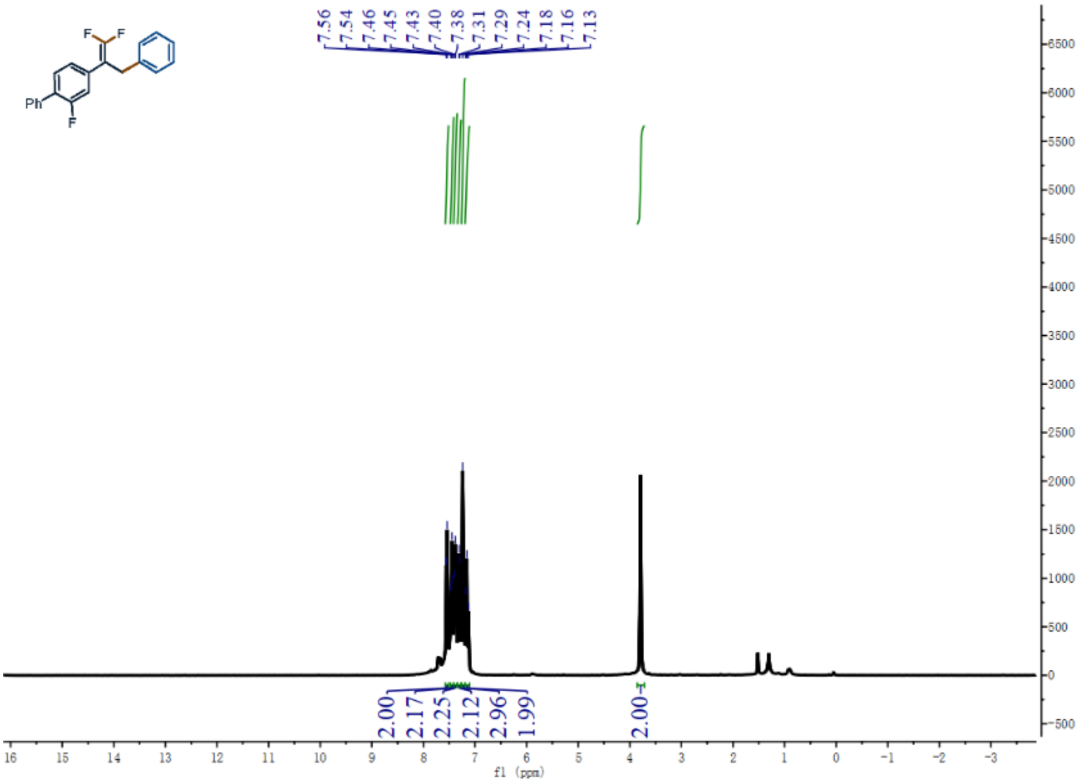

^{13}C NMR (100 MHz, $CDCl_3$) spectrum of compound **9u**

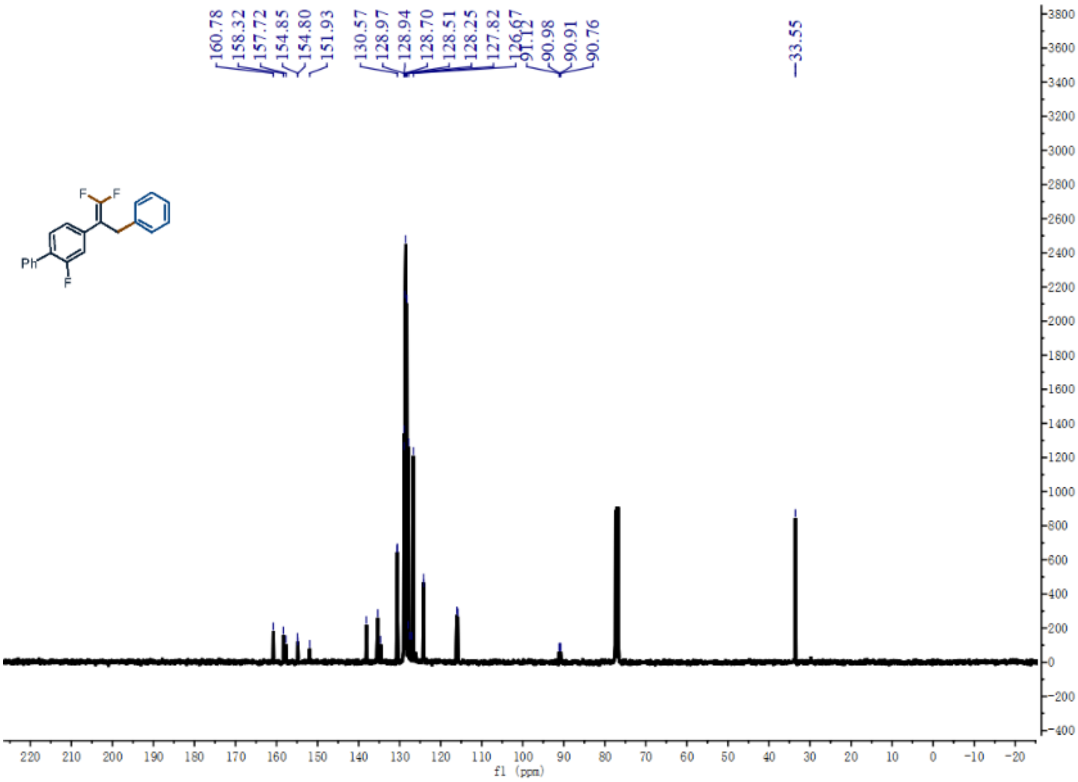

^{19}F NMR (376 MHz, $CDCl_3$) spectrum of compound **9u**

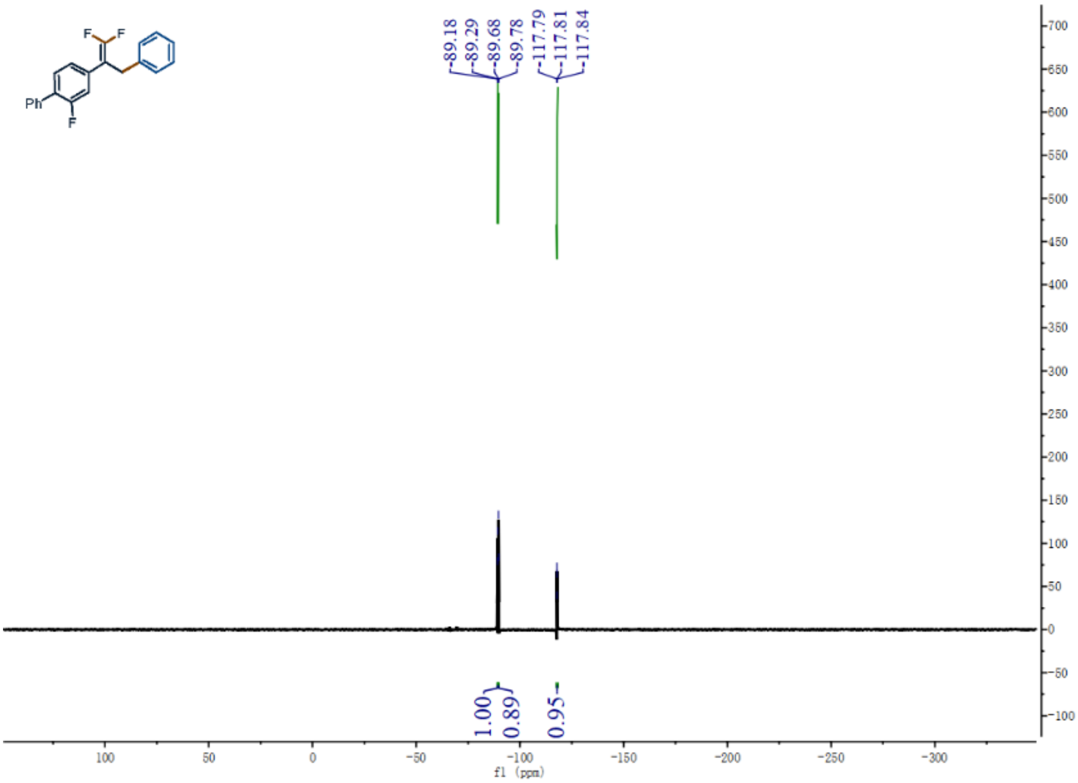

^{1}H NMR (400 MHz, $CDCl_3$) spectrum of compound **9v**

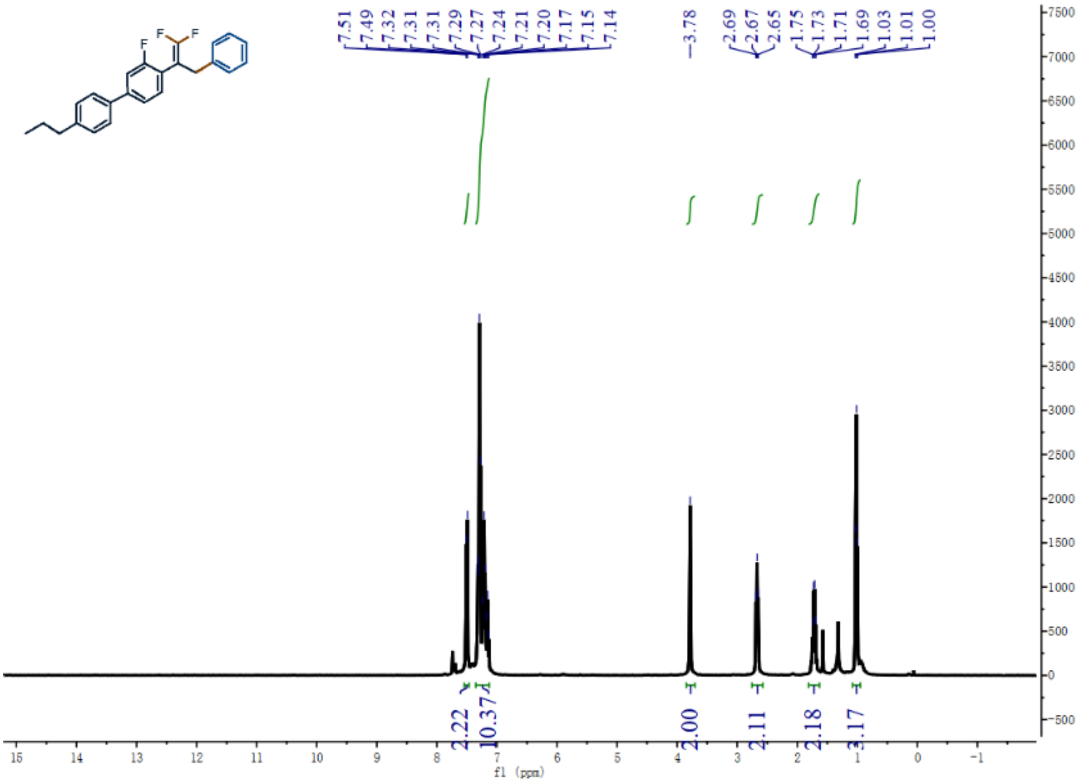

^{13}C NMR (100 MHz, $CDCl_3$) spectrum of compound **9v**

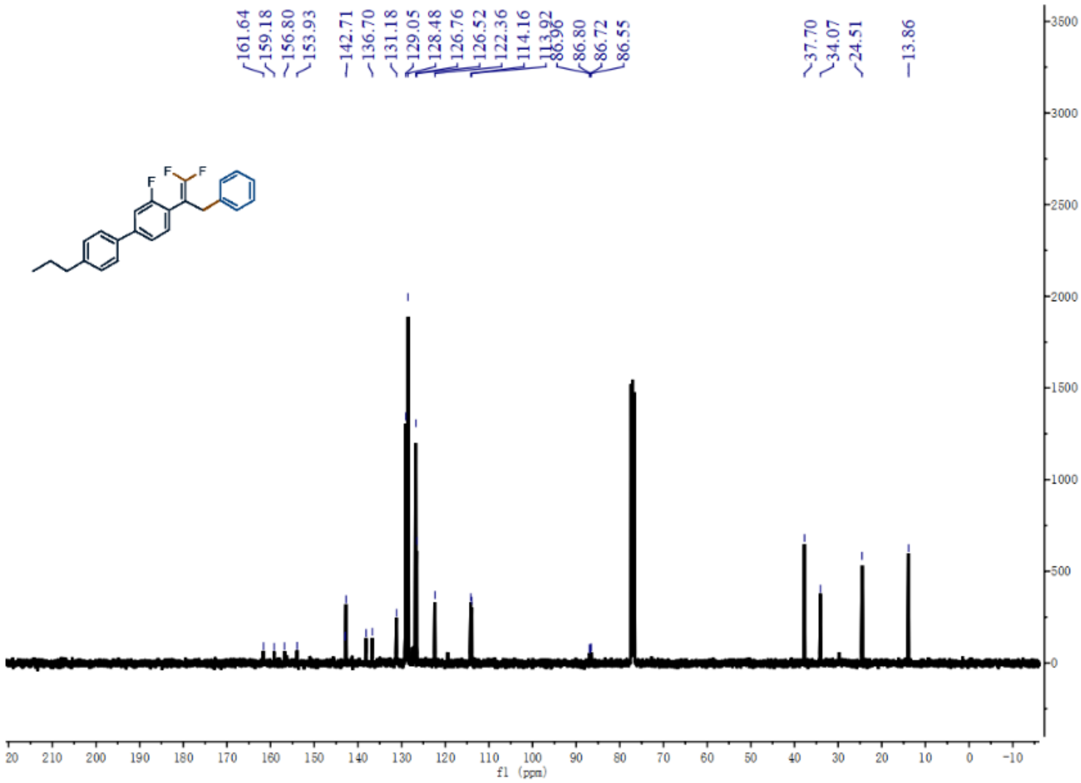

^{19}F NMR (376 MHz, $CDCl_3$) spectrum of compound **9v**

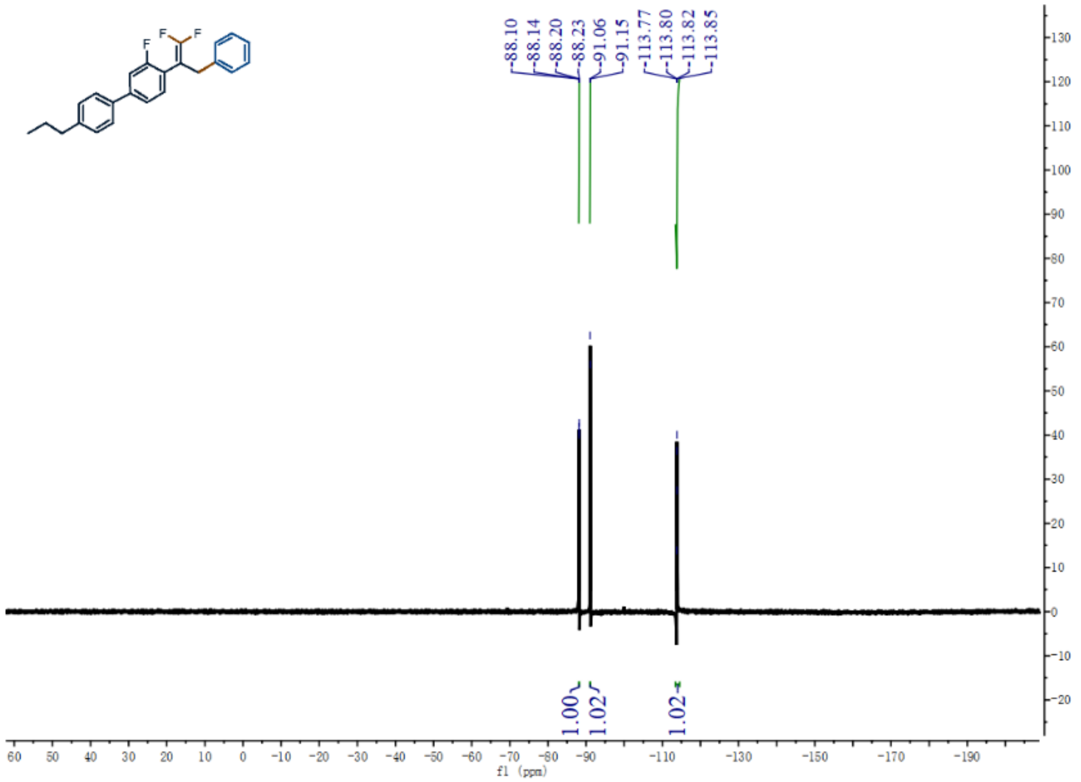

^{1}H NMR (400 MHz, $CDCl_3$) spectrum of compound **9w**

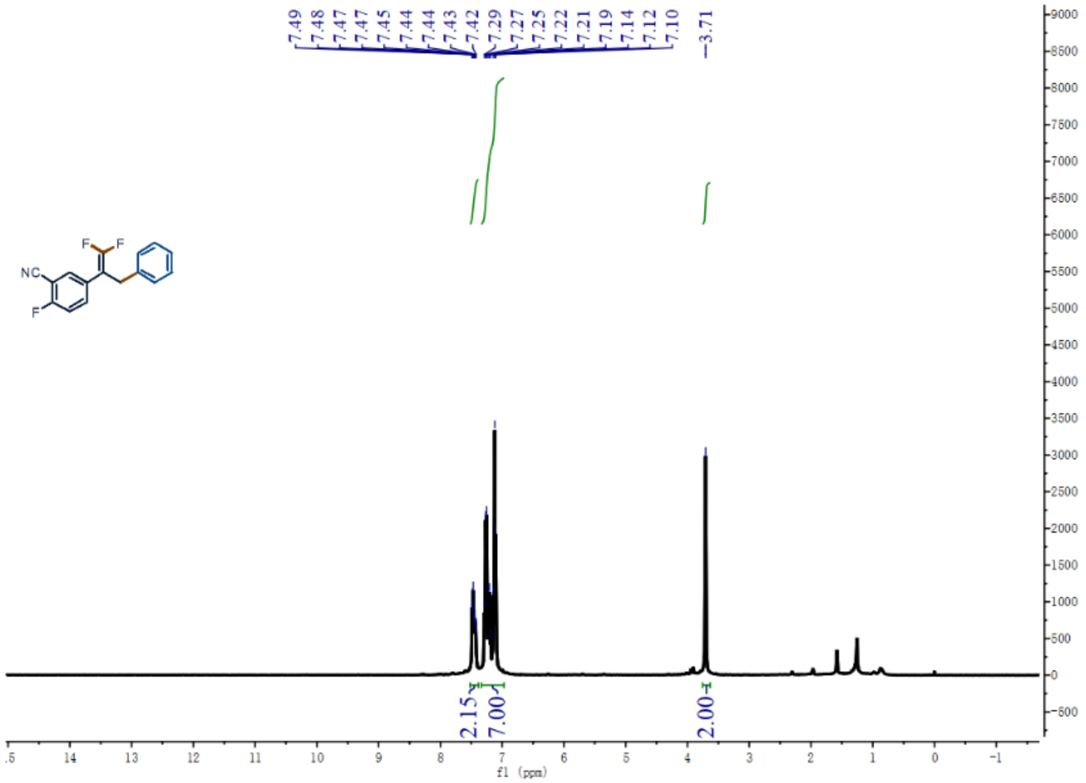

^{13}C NMR (100 MHz, $CDCl_3$) spectrum of compound **9w**

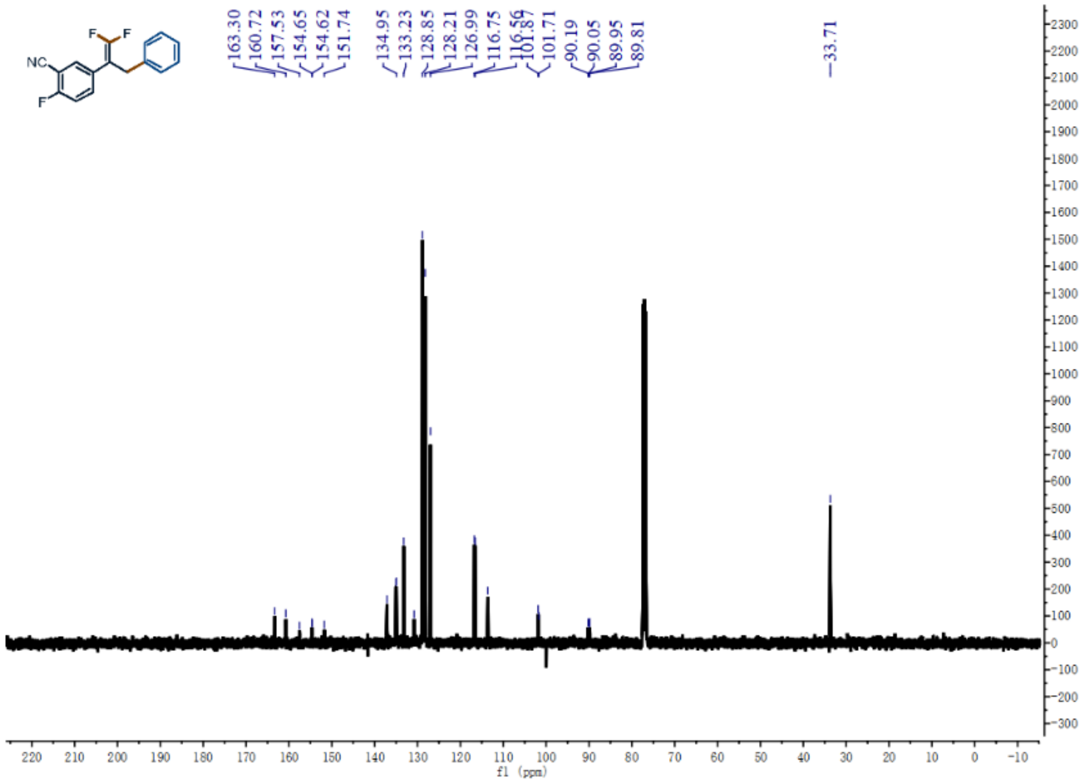

^{19}F NMR (376 MHz, $CDCl_3$) spectrum of compound **9w**

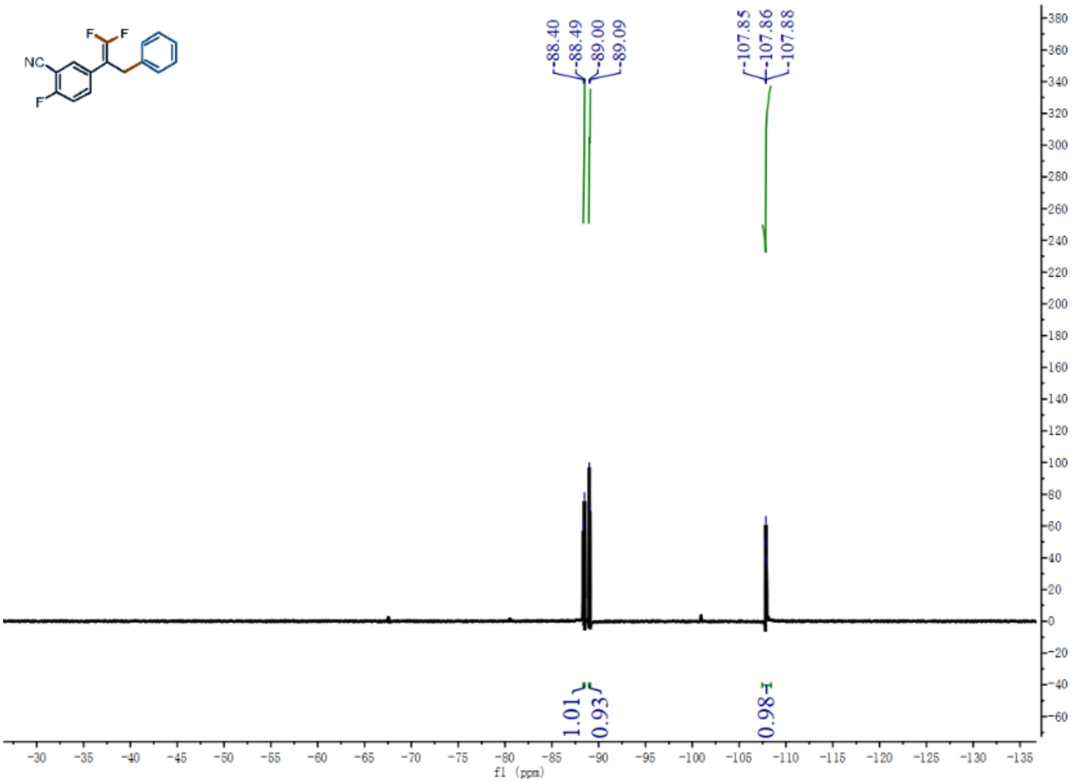

^{1}H NMR (400 MHz, $CDCl_3$) spectrum of compound **9x**

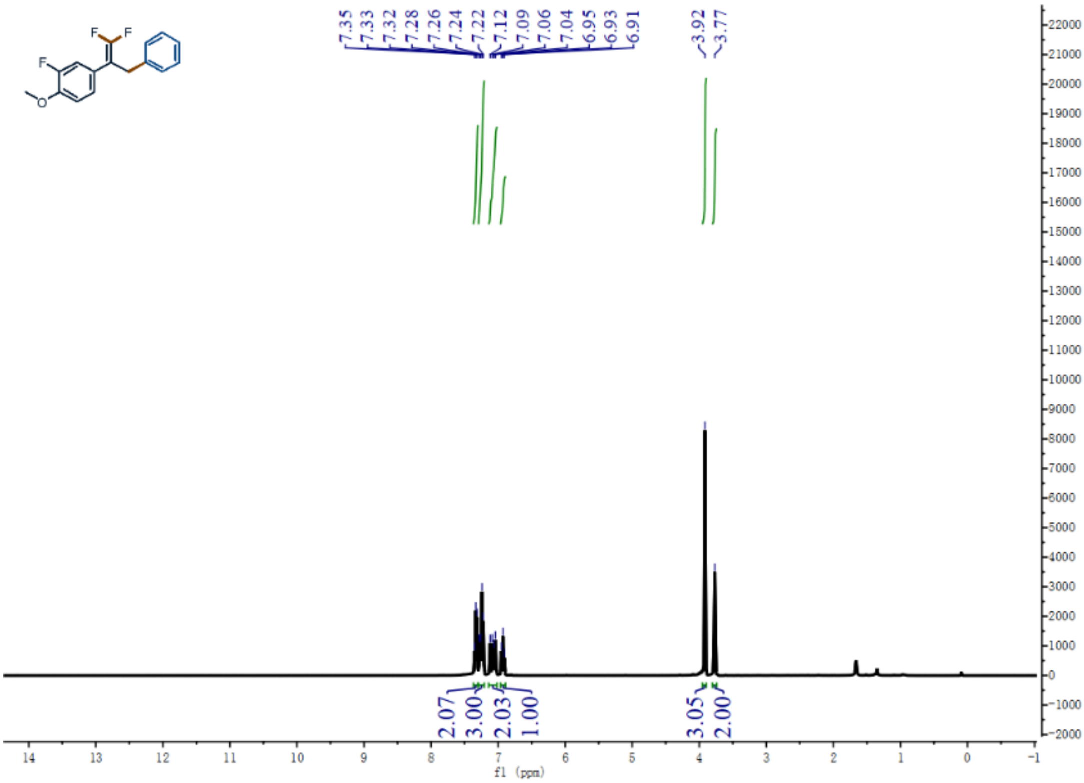

^{13}C NMR (100 MHz, $CDCl_3$) spectrum of compound **9x**

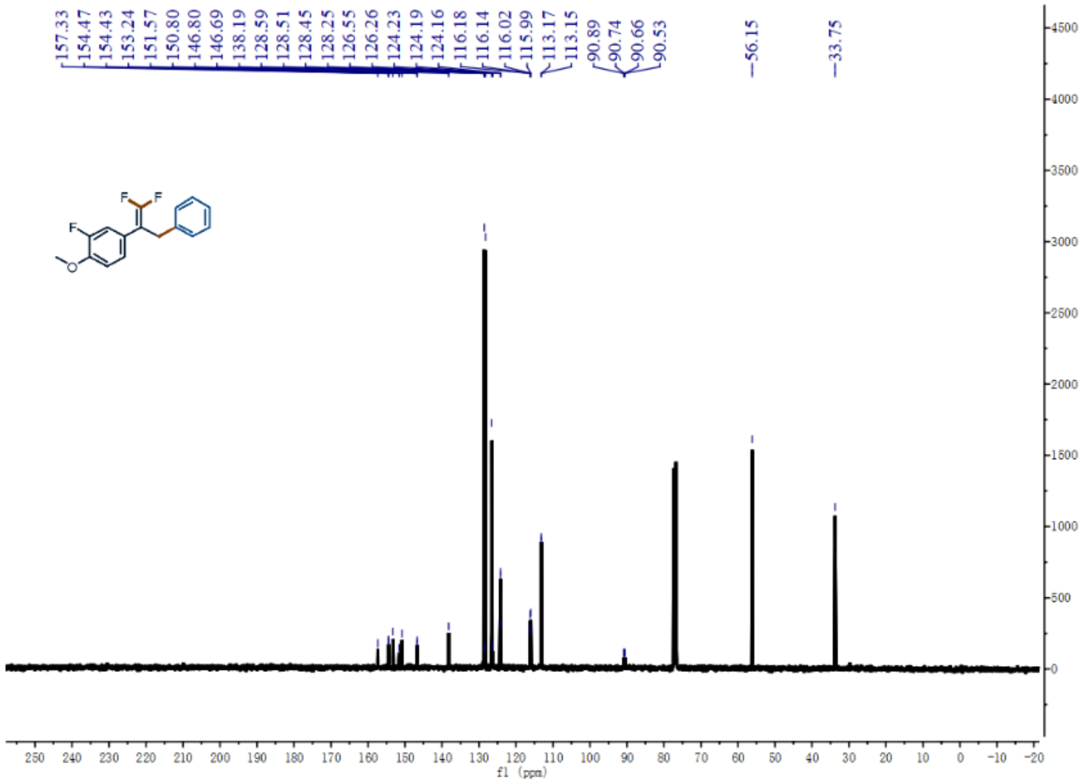

^{19}F NMR (376 MHz, CDCl$_3$) spectrum of compound **9x**

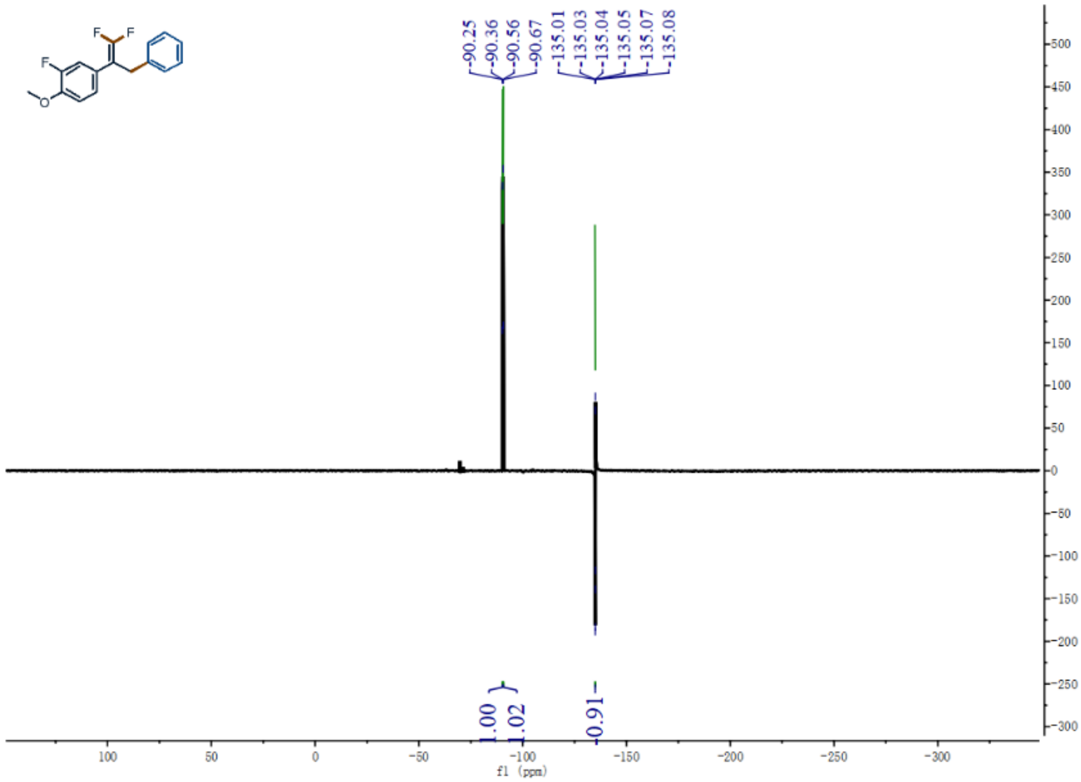

^{1}H NMR (400 MHz, CDCl$_3$) spectrum of compound **9y**

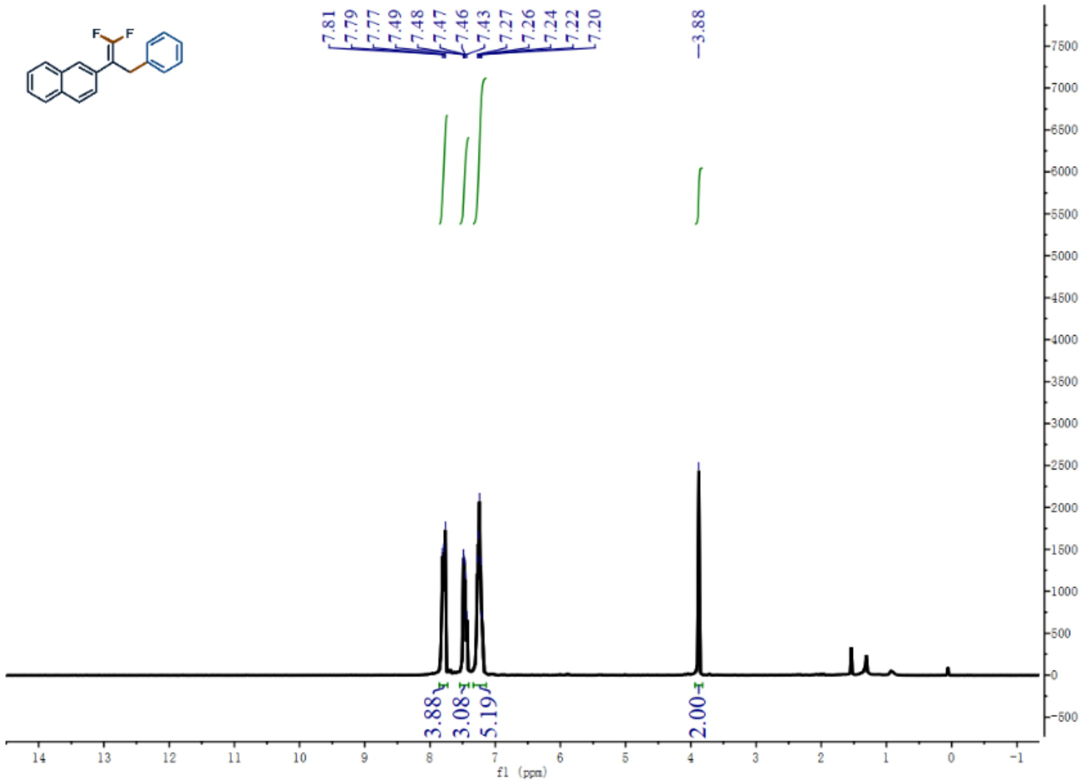

^{13}C NMR (100 MHz, $CDCl_3$) spectrum of compound **9y**

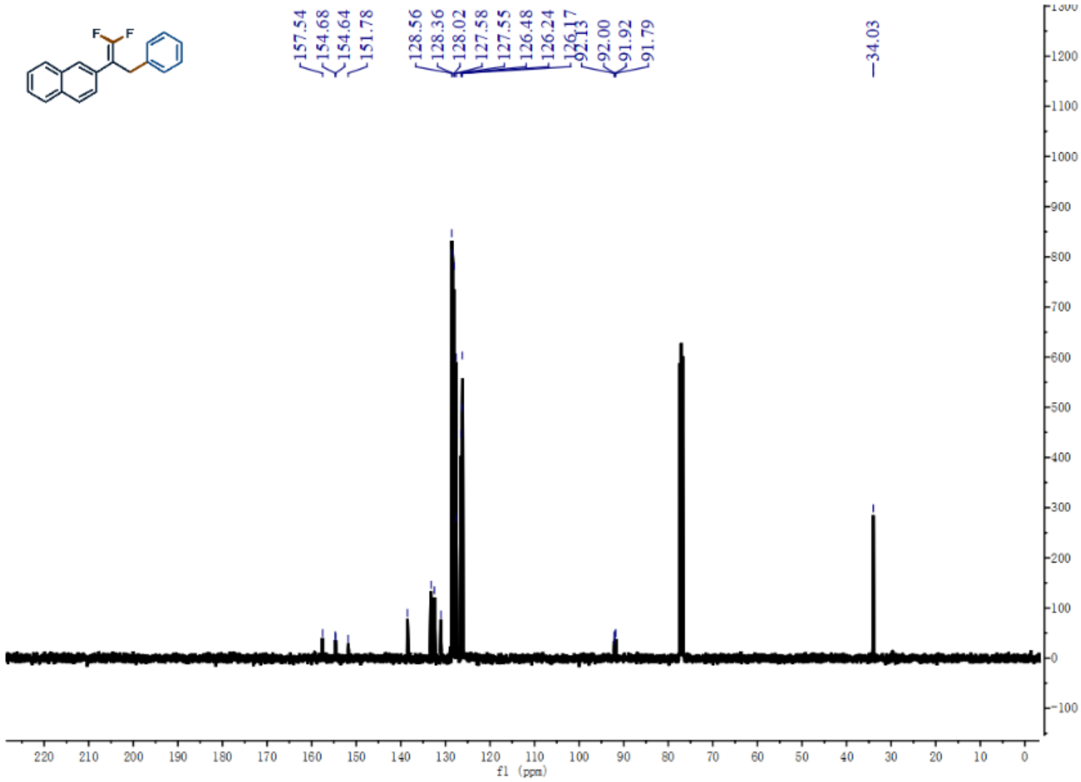

^{19}F NMR (376 MHz, $CDCl_3$) spectrum of compound **9y**

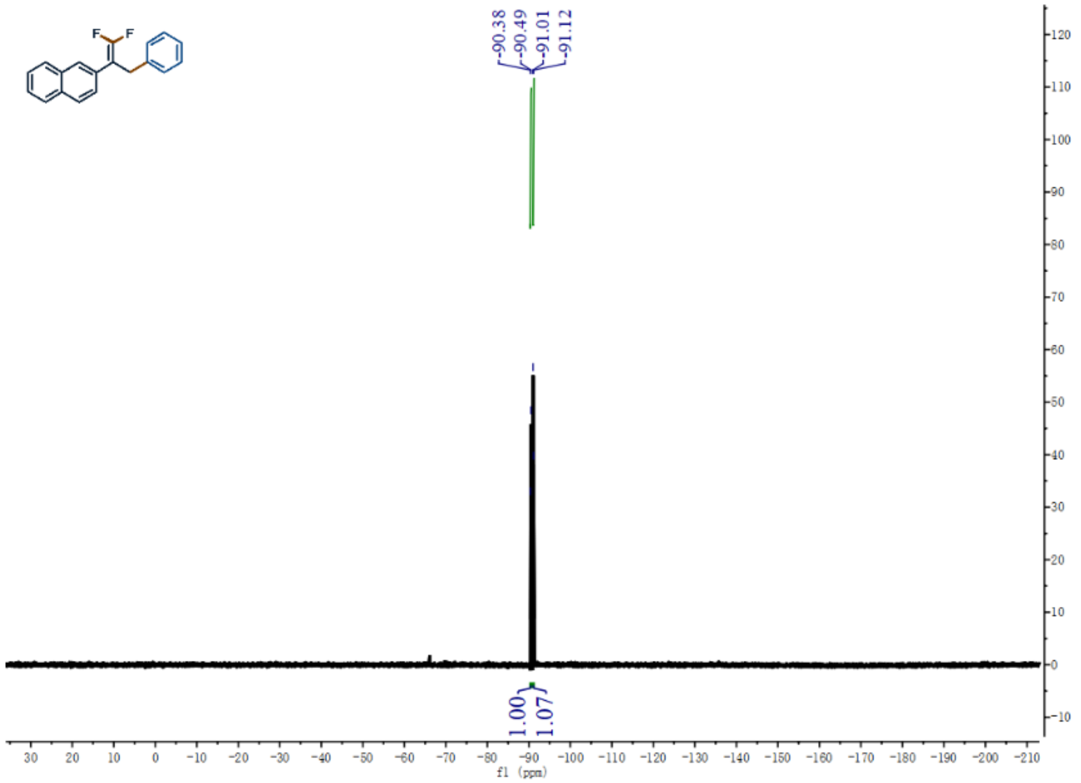

^{1}H NMR (400 MHz, $CDCl_3$) spectrum of compound **9z**

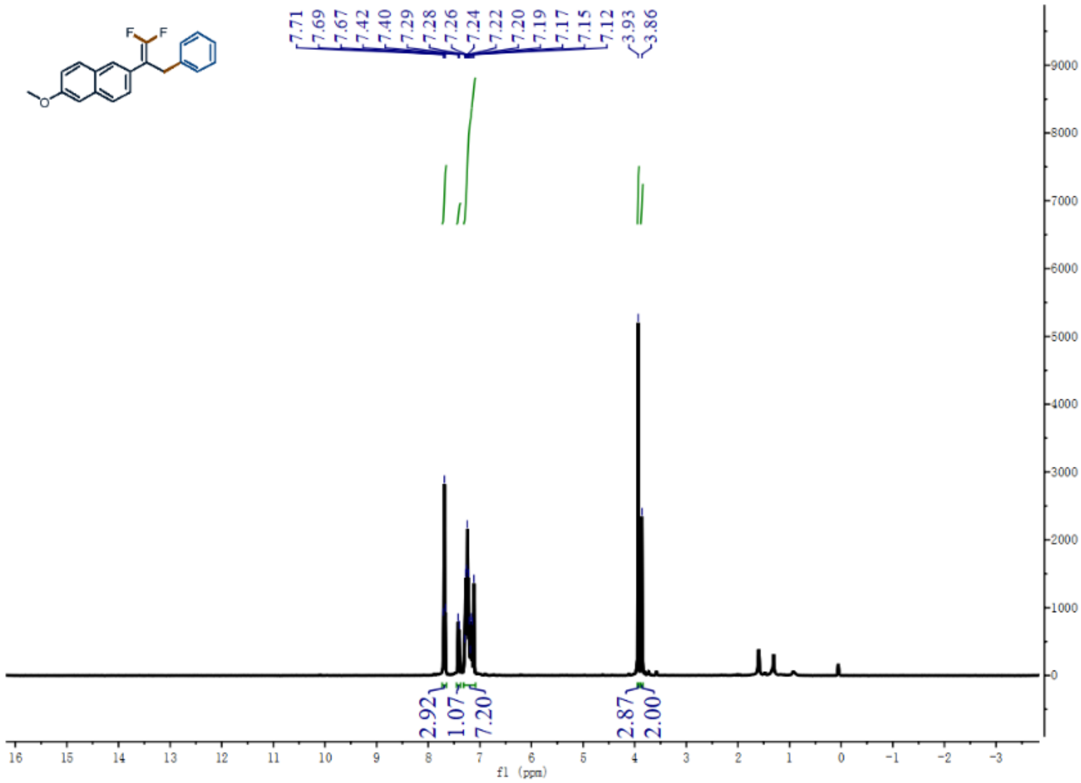

^{13}C NMR (100 MHz, $CDCl_3$) spectrum of compound **9z**

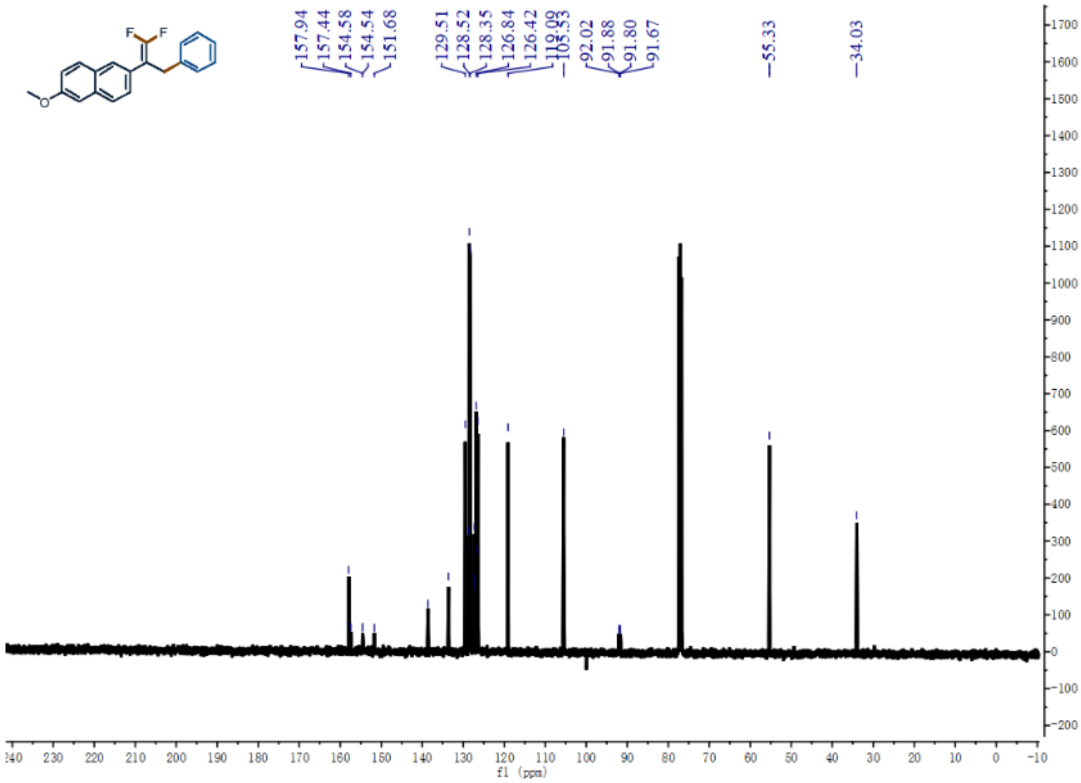

^{19}F NMR (376 MHz, $CDCl_3$) spectrum of compound **9z**

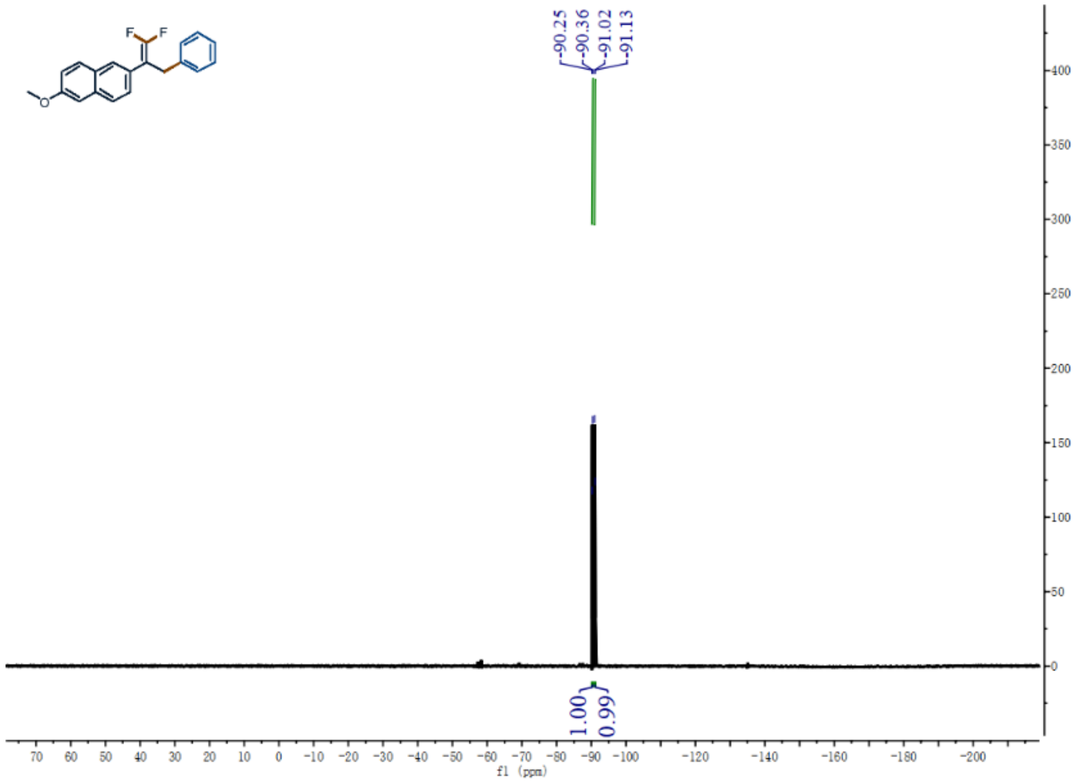

^{1}H NMR (400 MHz, $CDCl_3$) spectrum of compound **9aa**

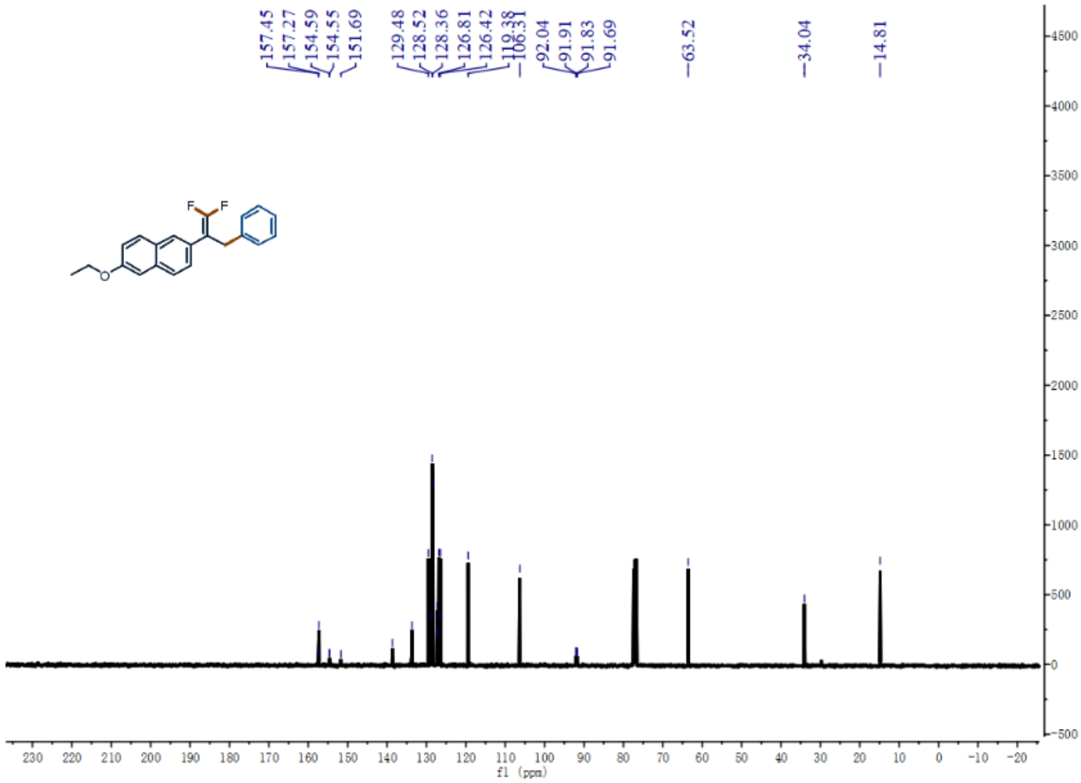

^{13}C NMR (100 MHz, $CDCl_3$) spectrum of compound **9aa**

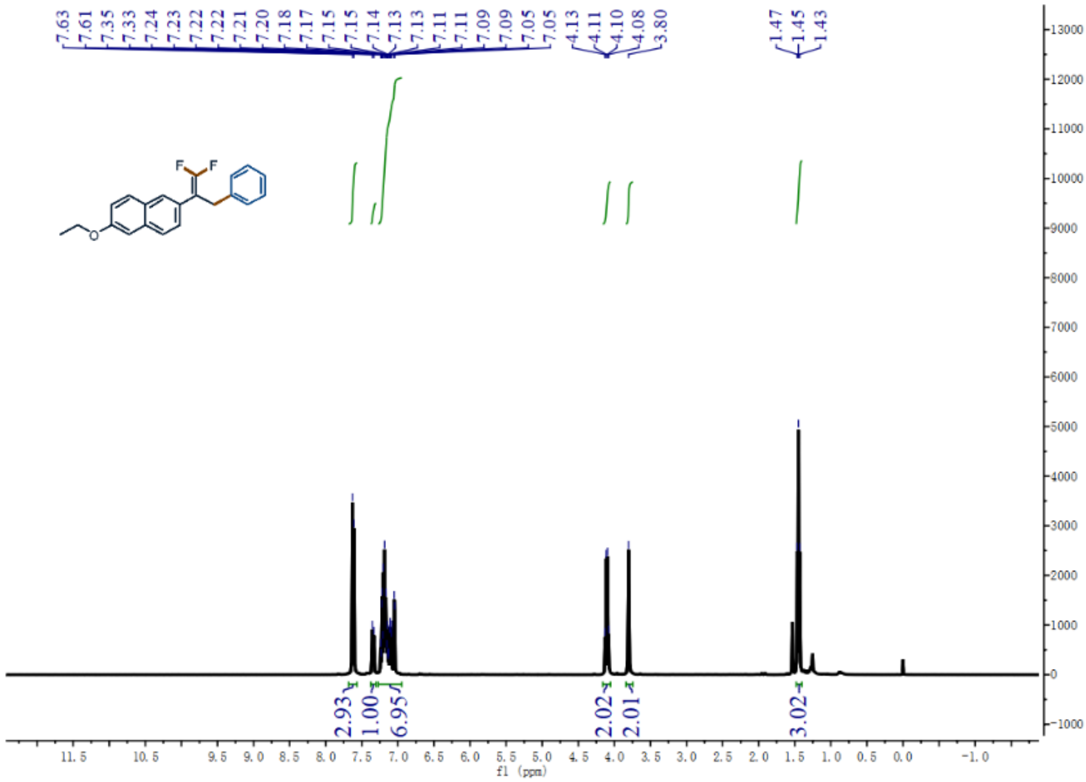

^{19}F NMR (376 MHz, $CDCl_3$) spectrum of compound **9aa**

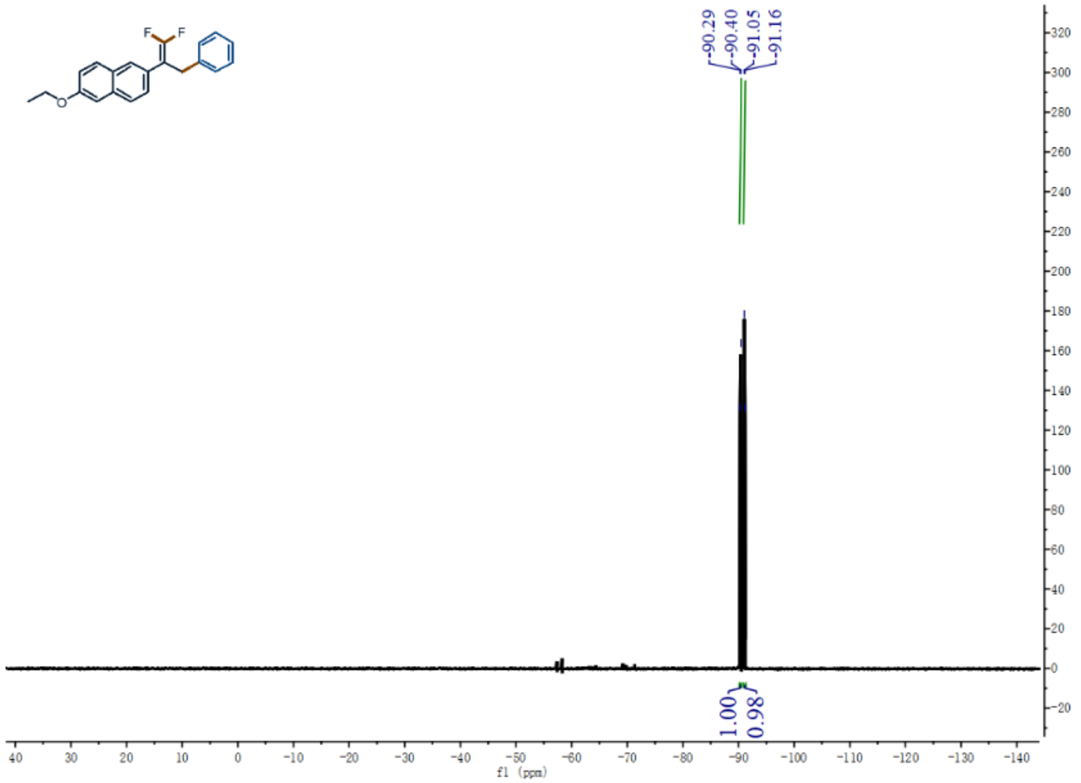

^{1}H NMR (400 MHz, $CDCl_3$) spectrum of compound **9ab**

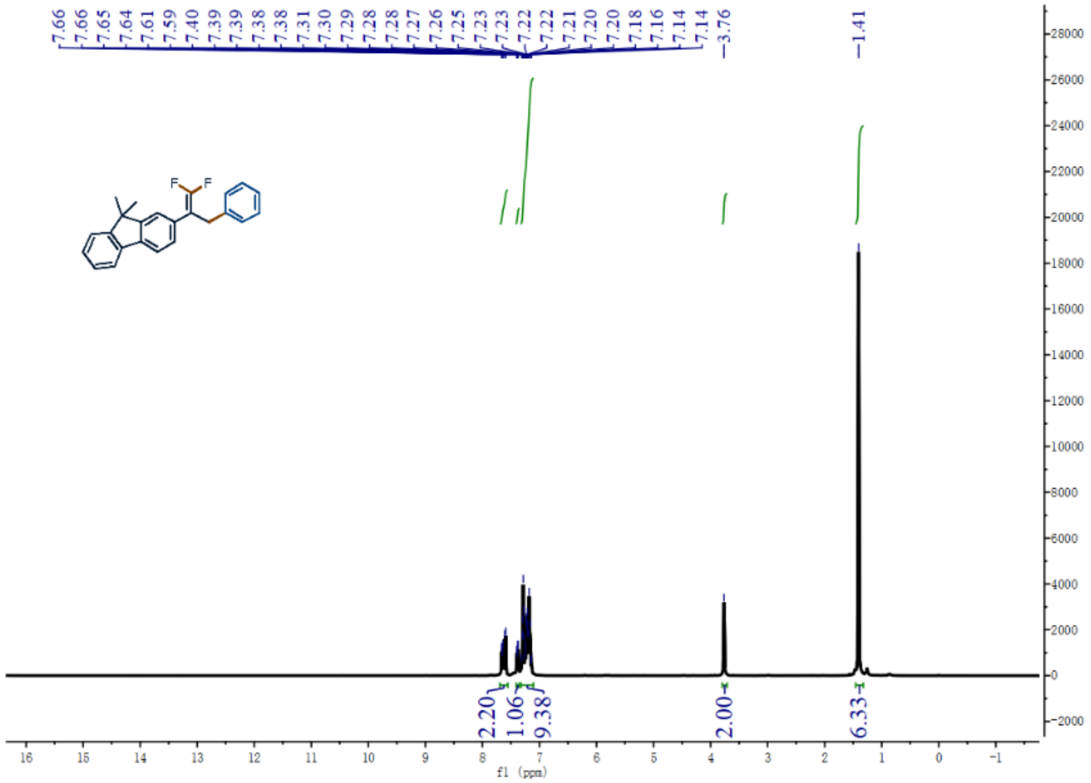

^{13}C NMR (100 MHz, $CDCl_3$) spectrum of compound **9ab**

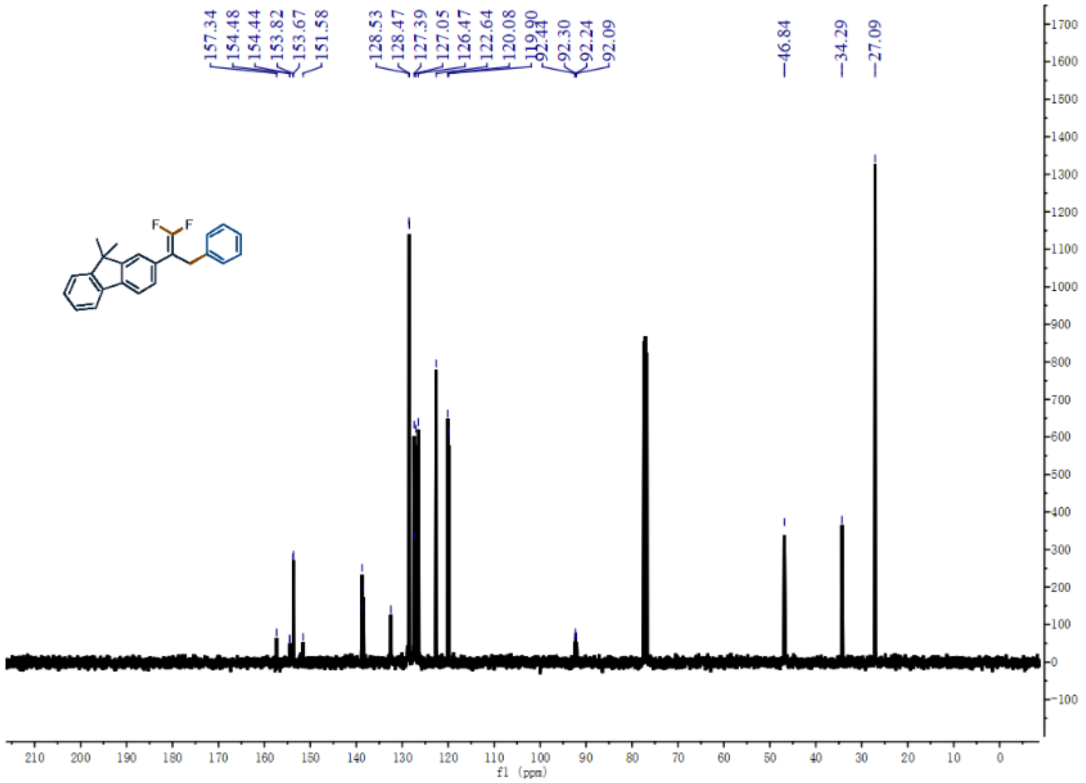

^{19}F NMR (376 MHz, $CDCl_3$) spectrum of compound **9ab**

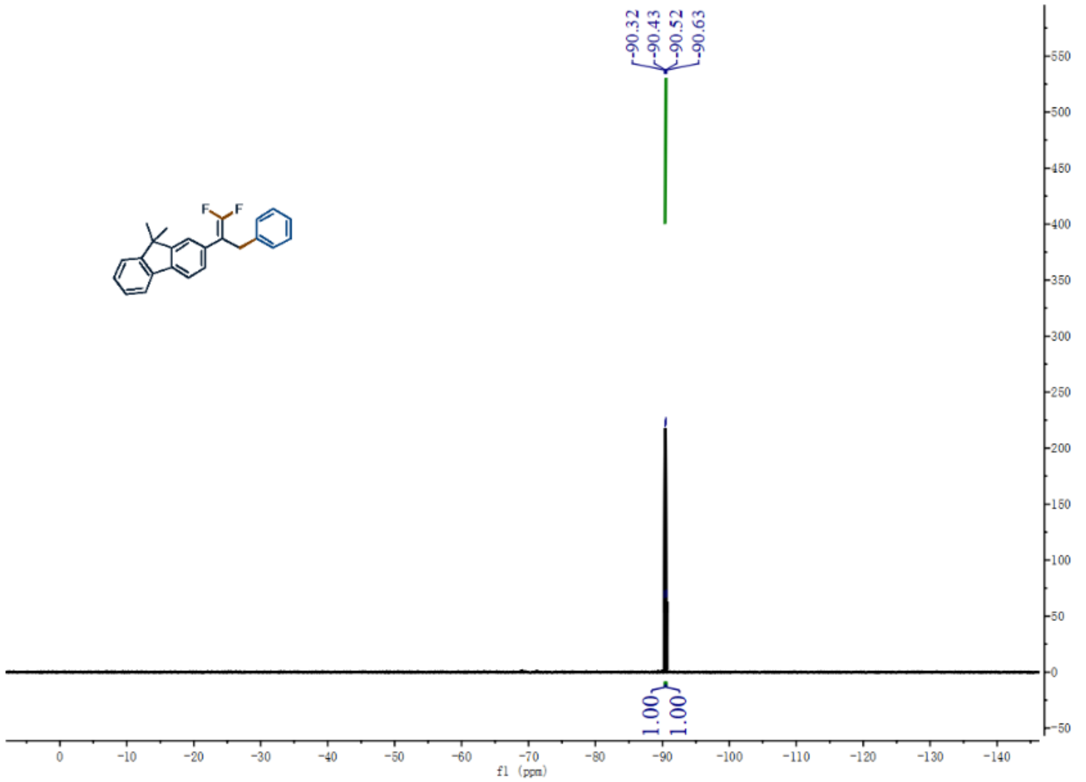

^{1}H NMR (400 MHz, $CDCl_3$) spectrum of compound **9ac**

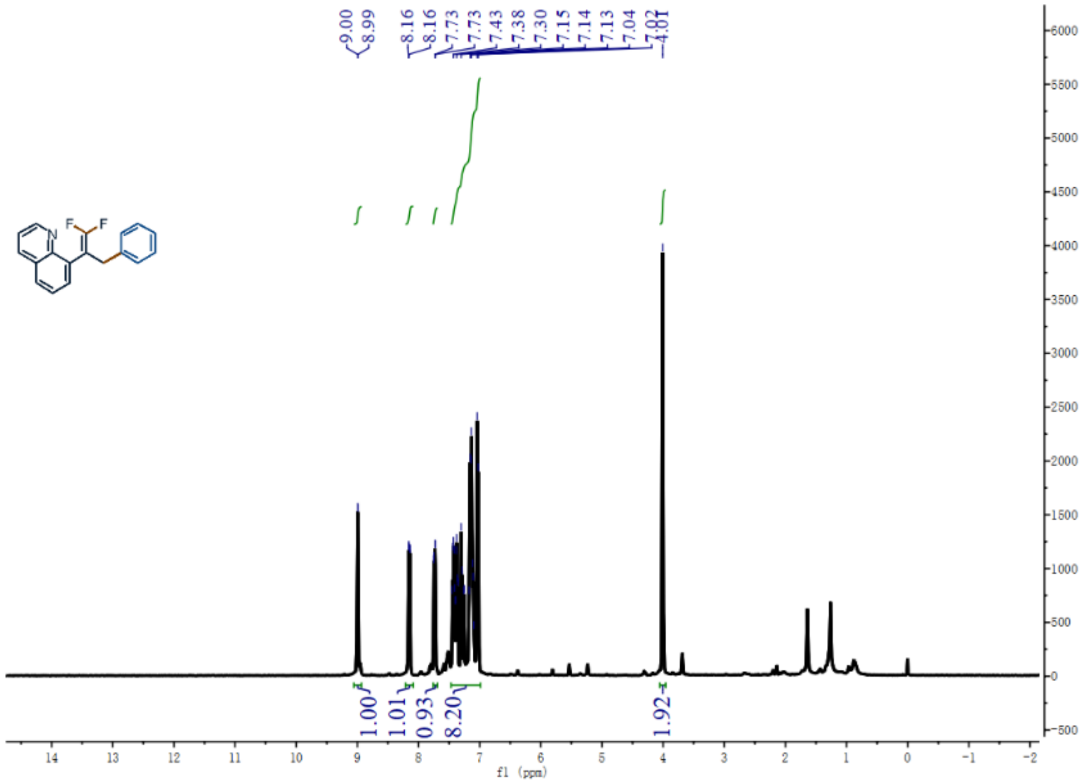

^{13}C NMR (100 MHz, $CDCl_3$) spectrum of compound **9ac**

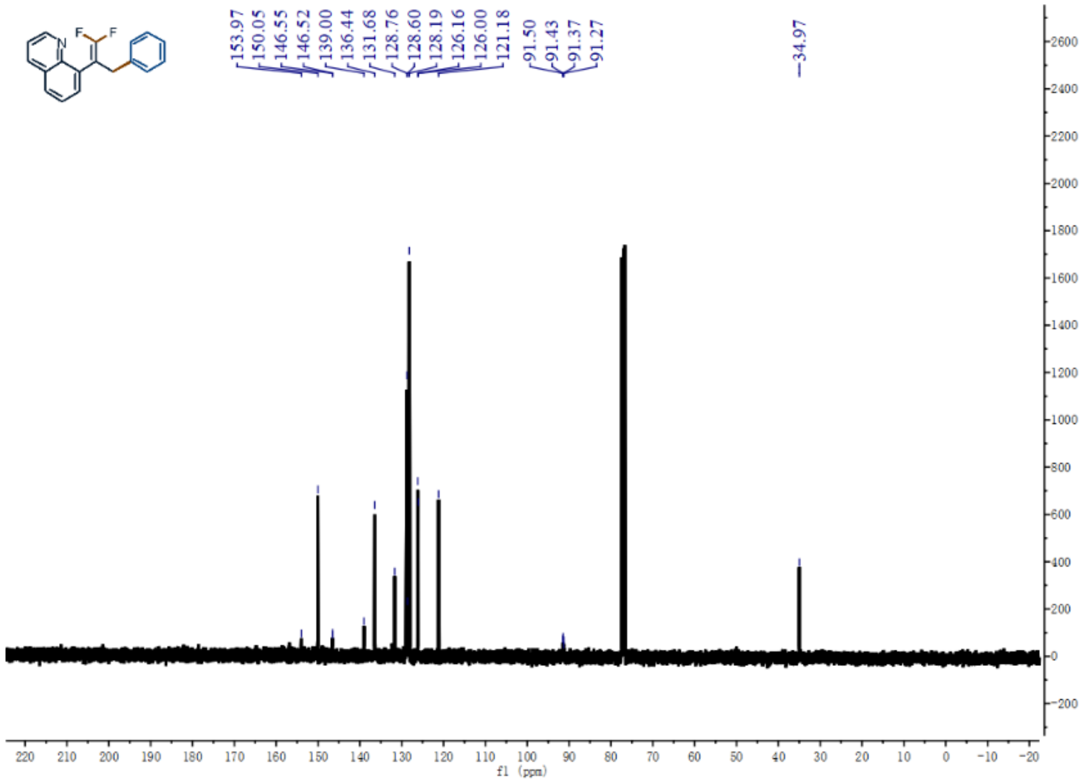

^{19}F NMR (376 MHz, $CDCl_3$) spectrum of compound **9ac**

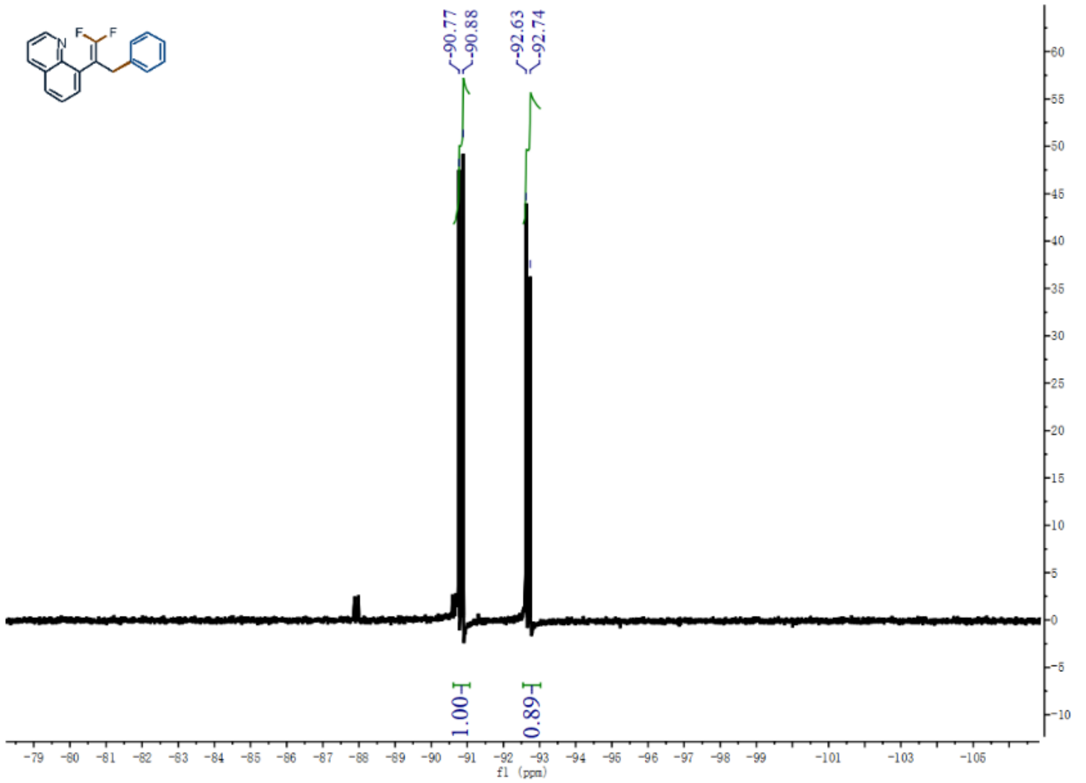

^{1}H NMR (400 MHz, $CDCl_3$) spectrum of compound **9ad**

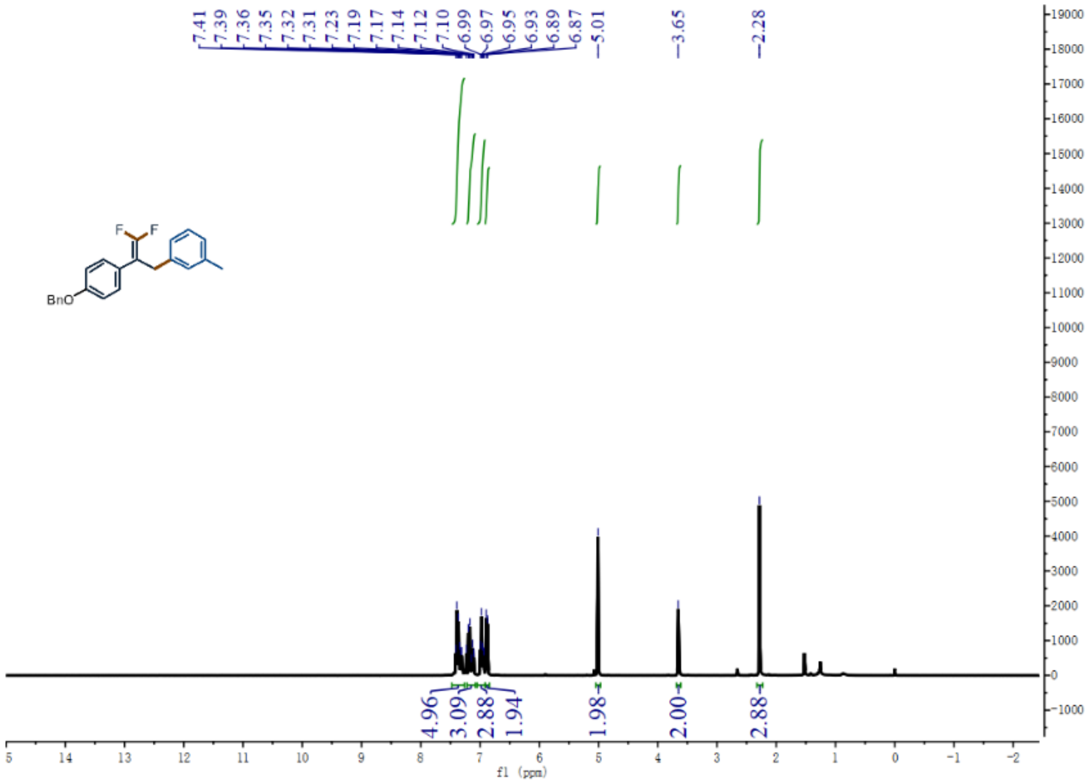

^{13}C NMR (100 MHz, $CDCl_3$) spectrum of compound **9ad**

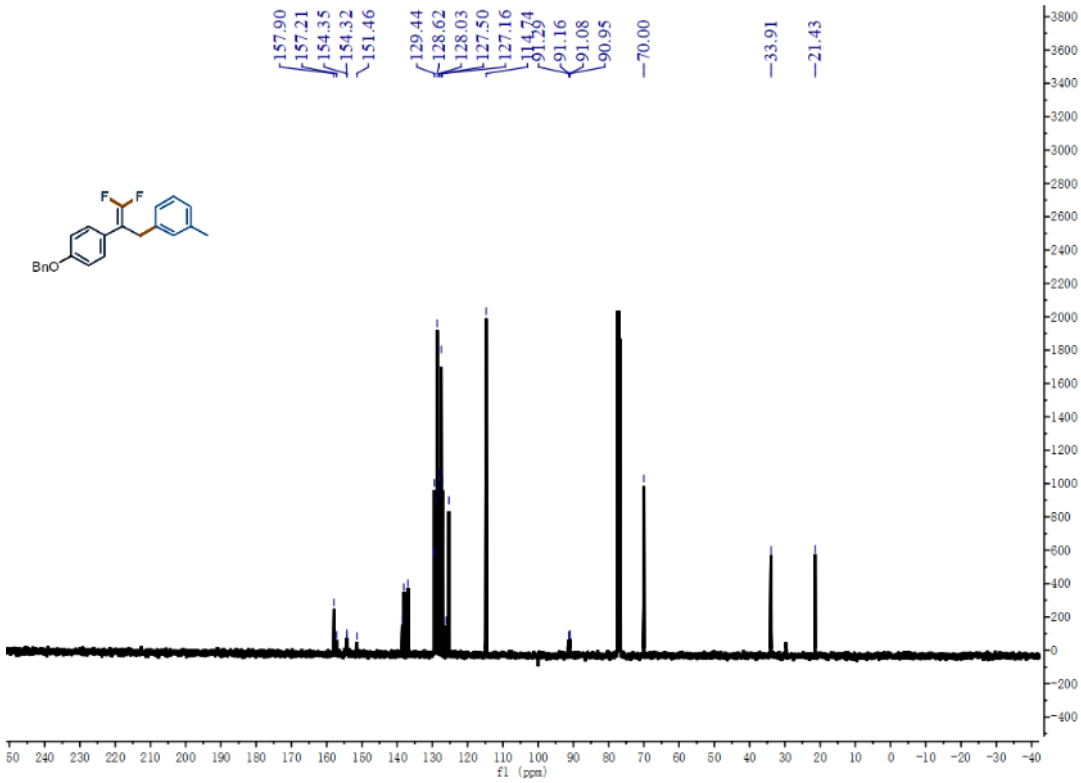

^{19}F NMR (376 MHz, $CDCl_3$) spectrum of compound **9ad**

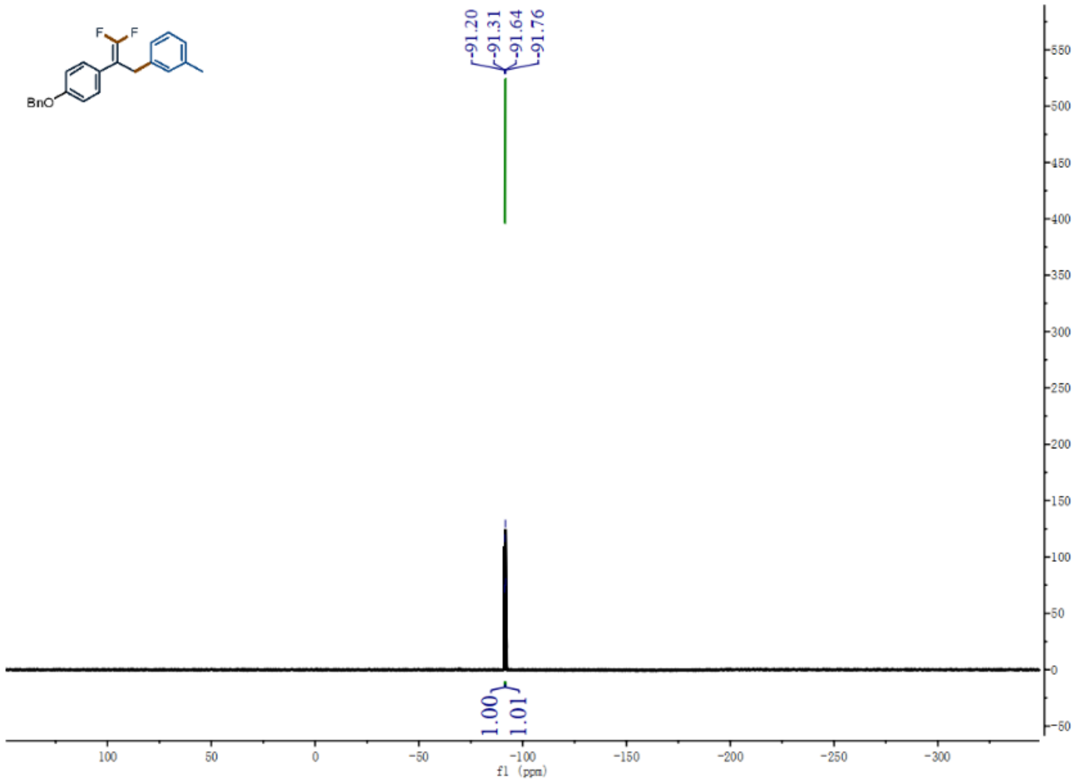

^{1}H NMR (400 MHz, $CDCl_3$) spectrum of compound **11**

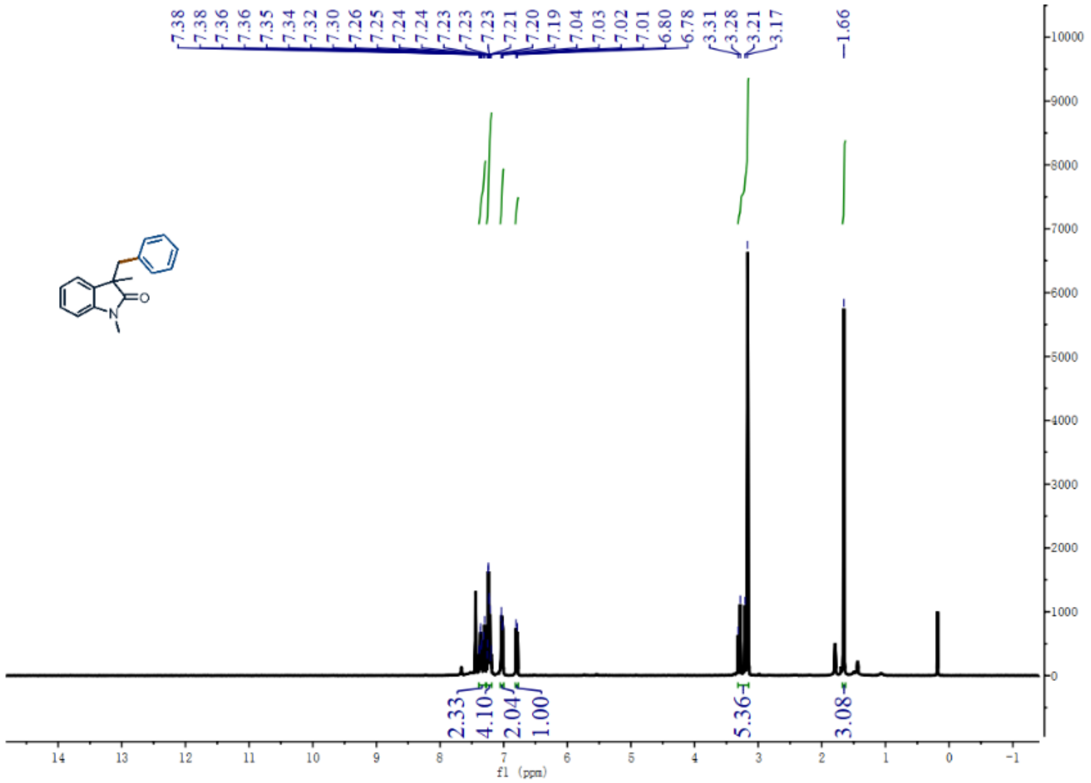

^{13}C NMR (100 MHz, CDCl$_3$) spectrum of compound **11**

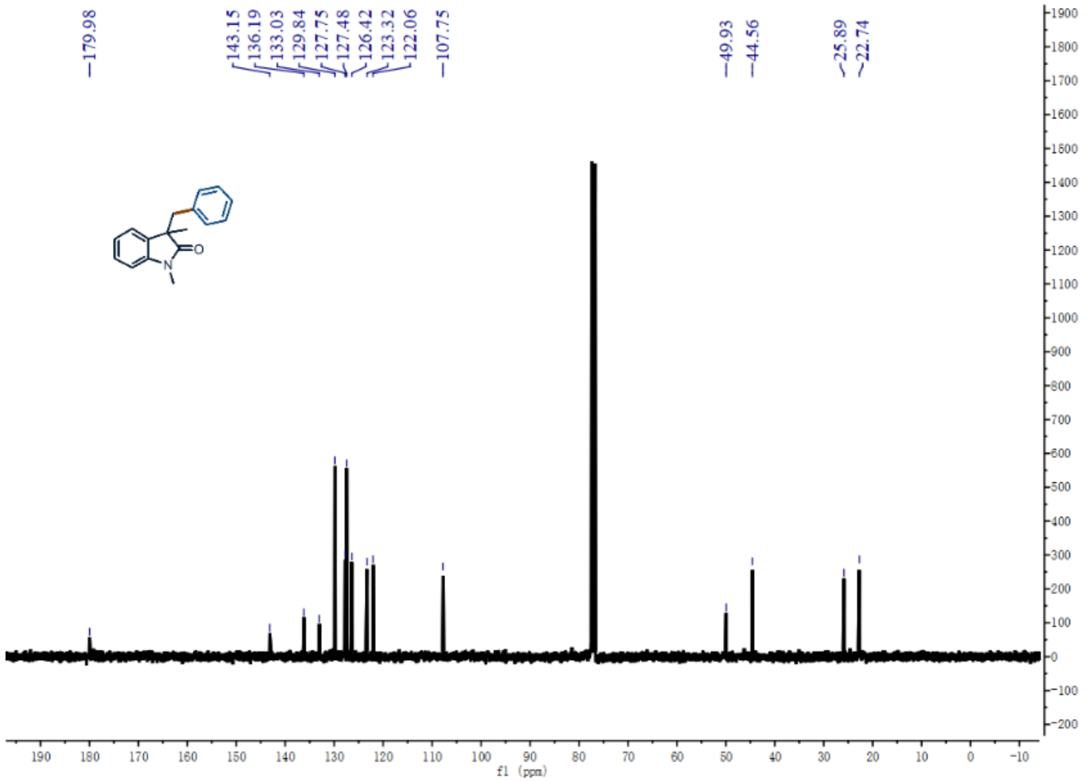

^{1}H NMR (400 MHz, CDCl$_3$) spectrum of compound **12a**

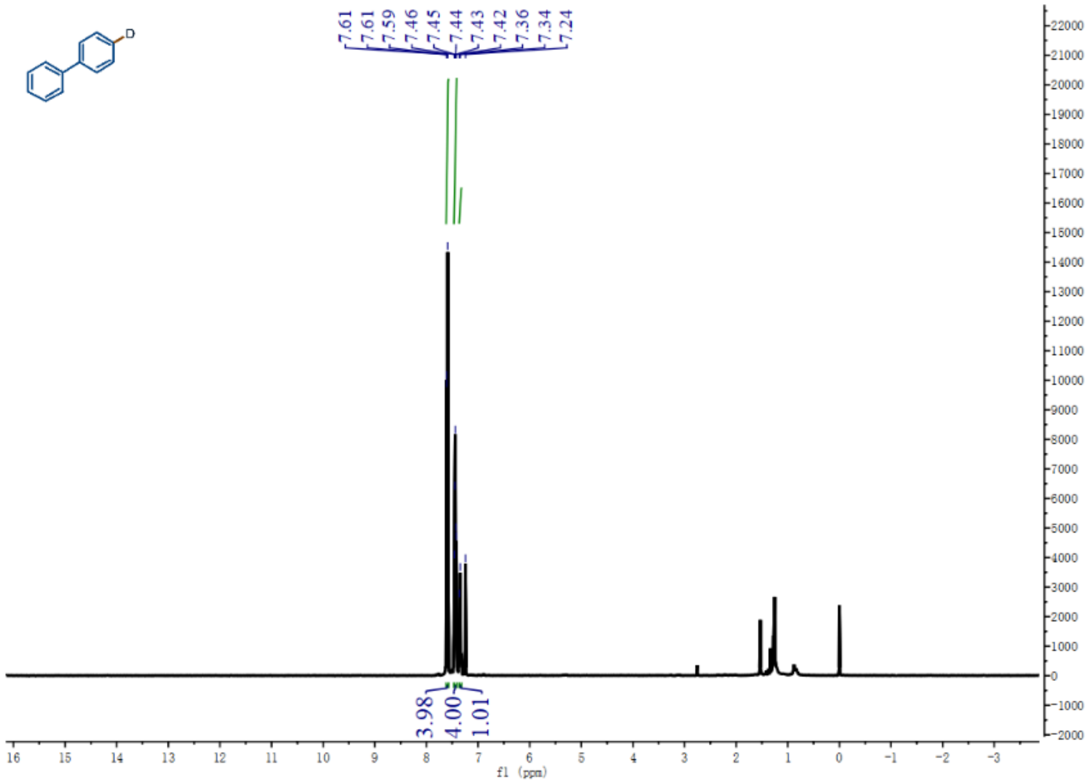

^{13}C NMR (100 MHz, $CDCl_3$) spectrum of compound **12a**

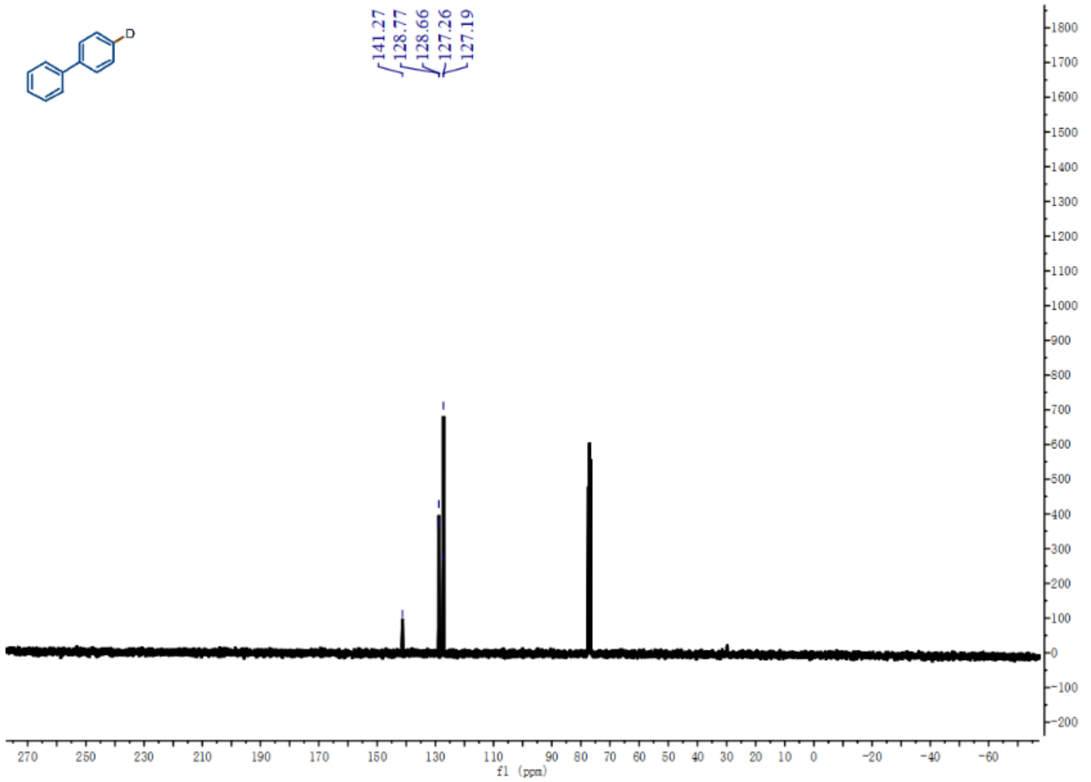

^{1}H NMR (400 MHz, $CDCl_3$) spectrum of compound **12b**

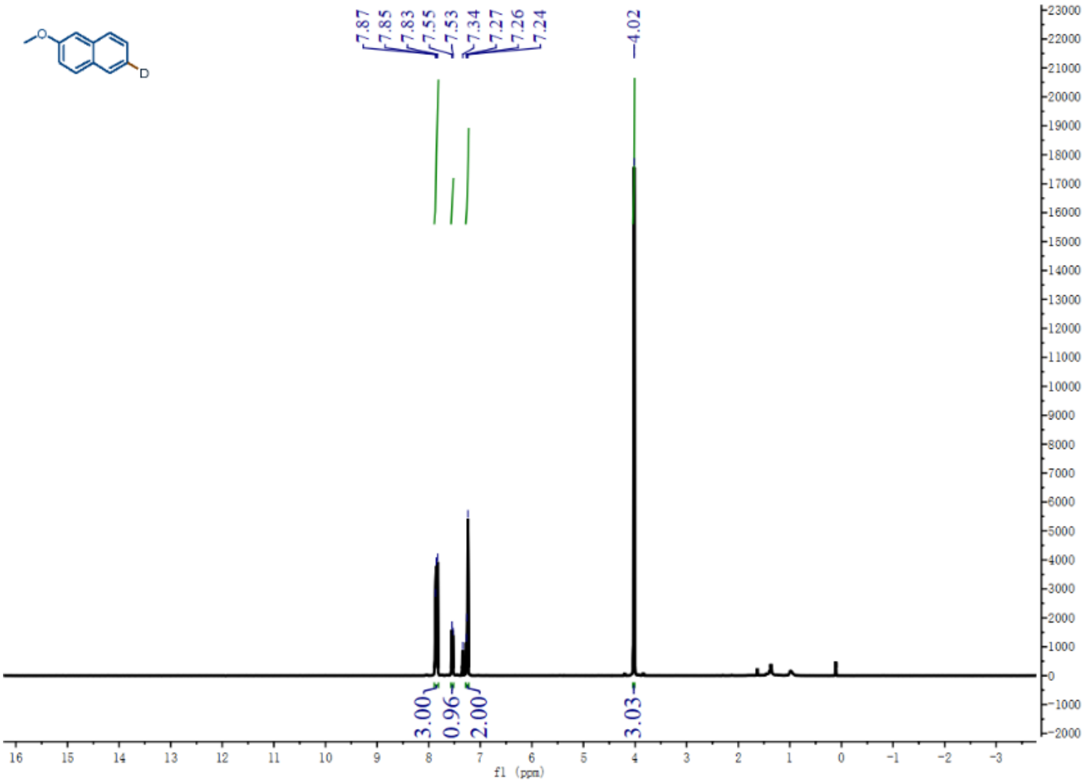

^{13}C NMR (100 MHz, $CDCl_3$) spectrum of compound **12b**

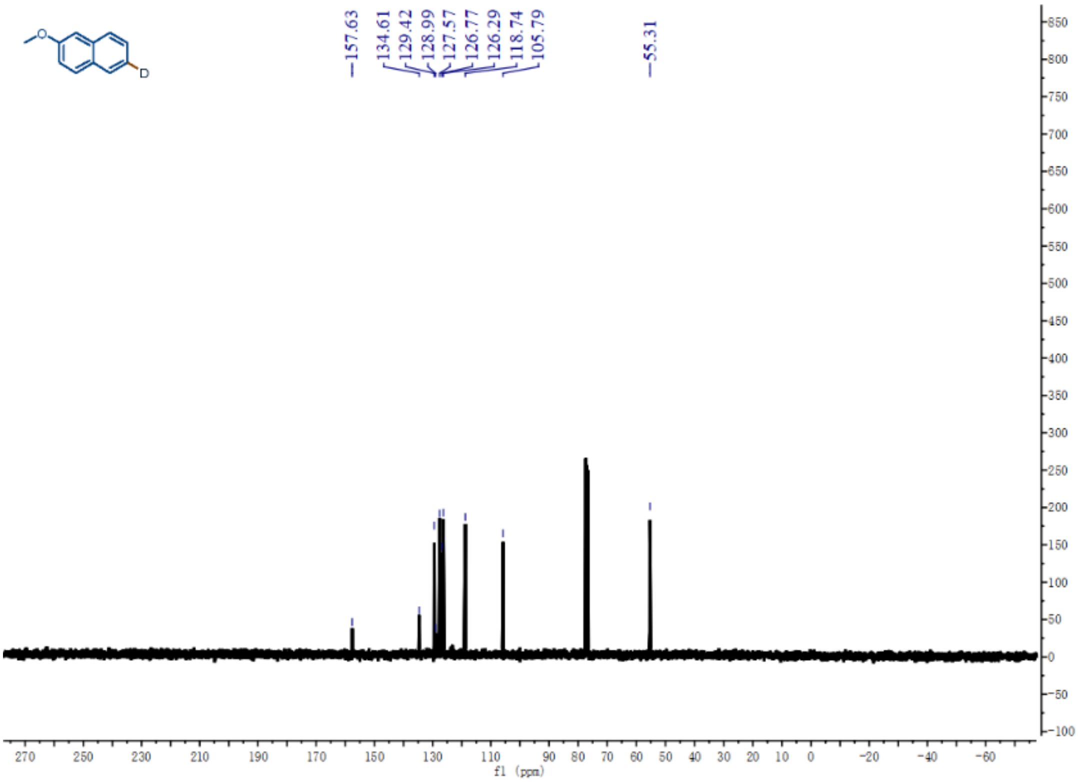

^{1}H NMR (400 MHz, $CDCl_3$) spectrum of compound **14**

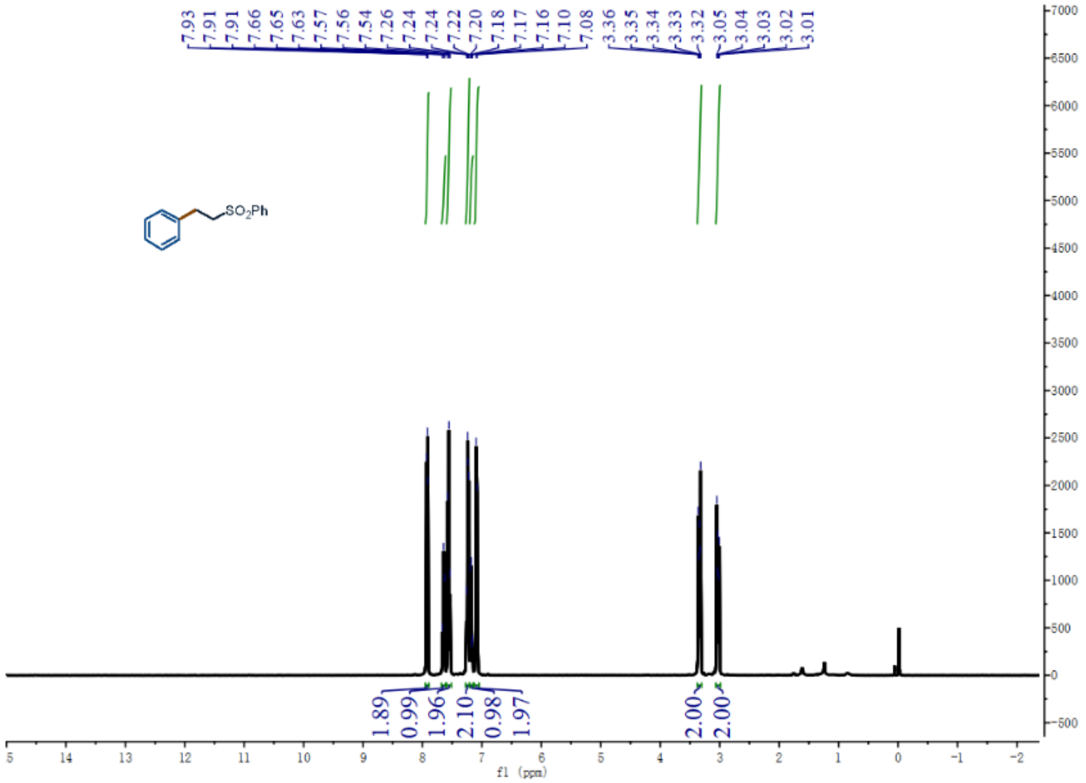

^{13}C NMR (100 MHz, $CDCl_3$) spectrum of compound **14**

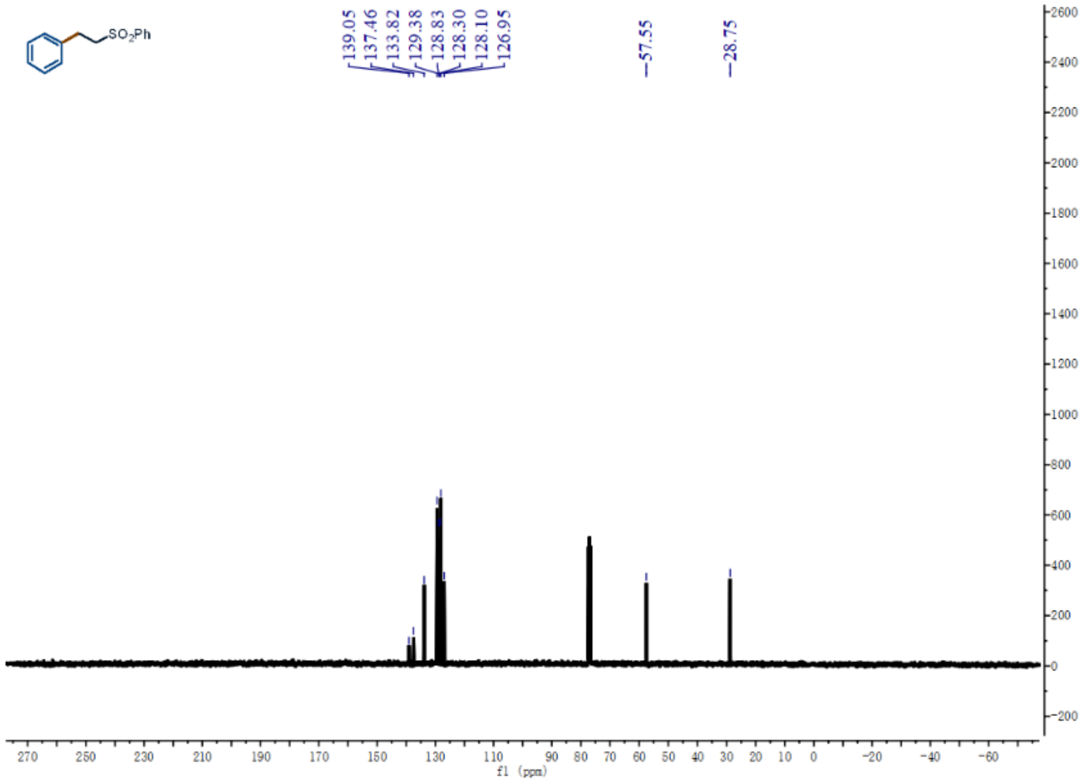

^{1}H NMR (400 MHz, $CDCl_3$) spectrum of compound **16**

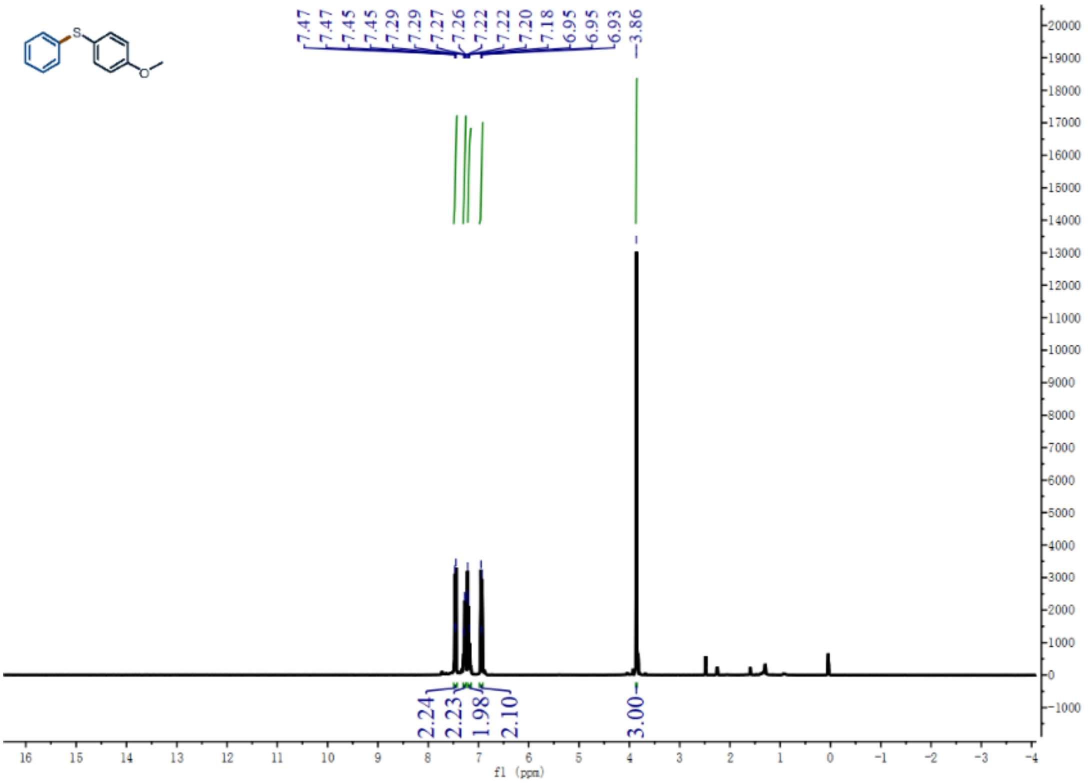

^{13}C NMR (100 MHz, $CDCl_3$) spectrum of compound **16**

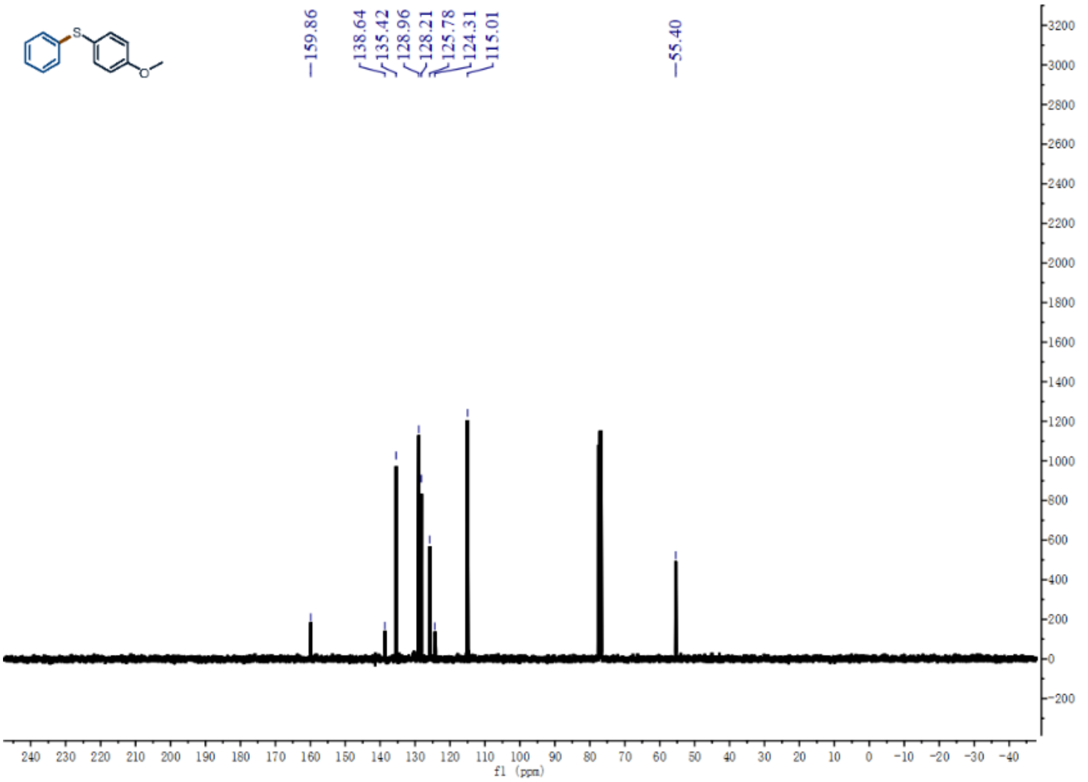

^{1}H NMR (400 MHz, $CDCl_3$) spectrum of compound **17**

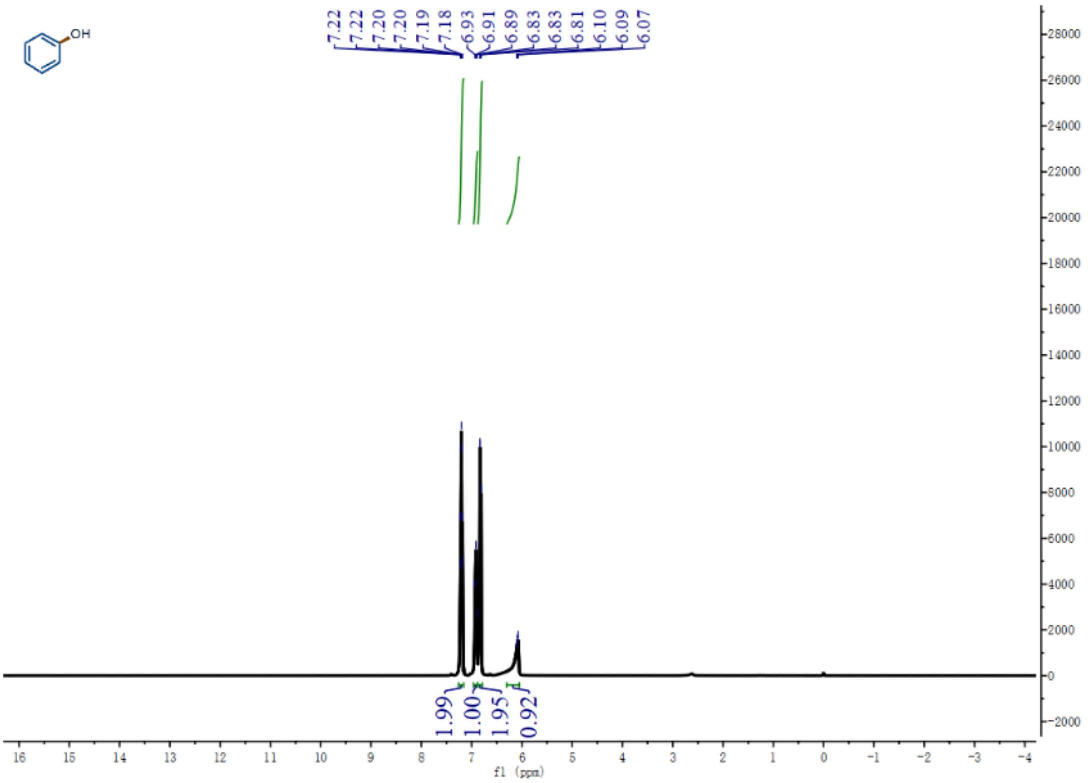

^{13}C NMR (100 MHz, $CDCl_3$) spectrum of compound **17**

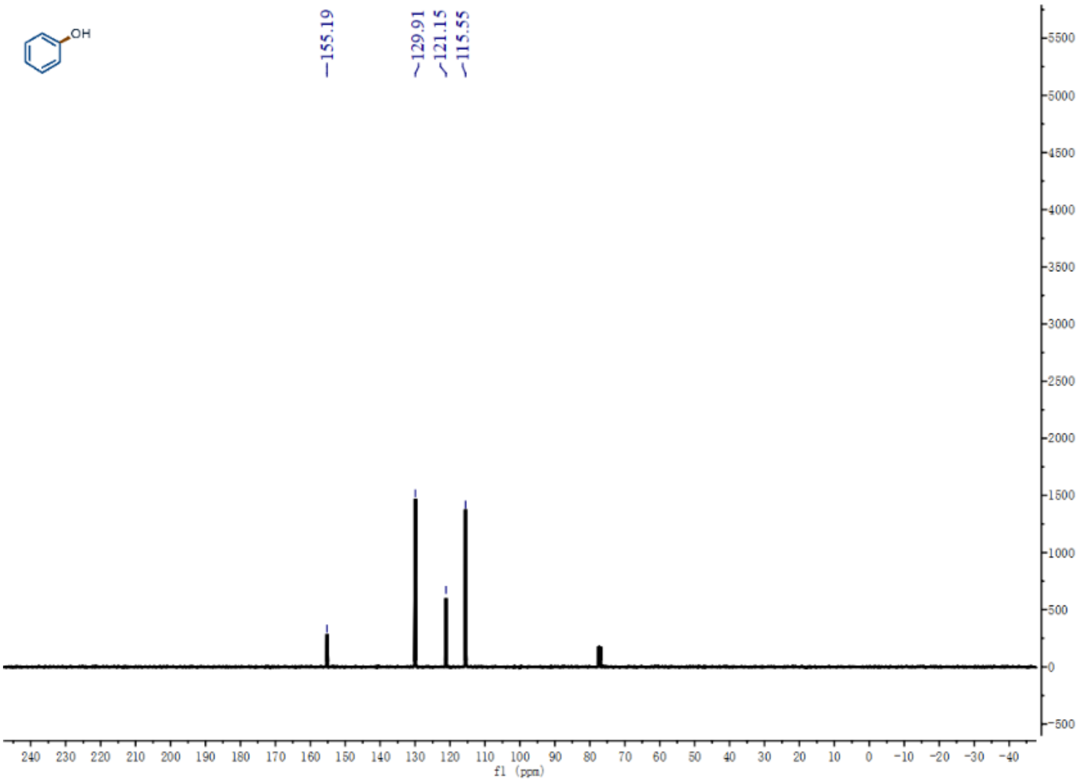

^{1}H NMR (400 MHz, $CDCl_3$) spectrum of compound **18**

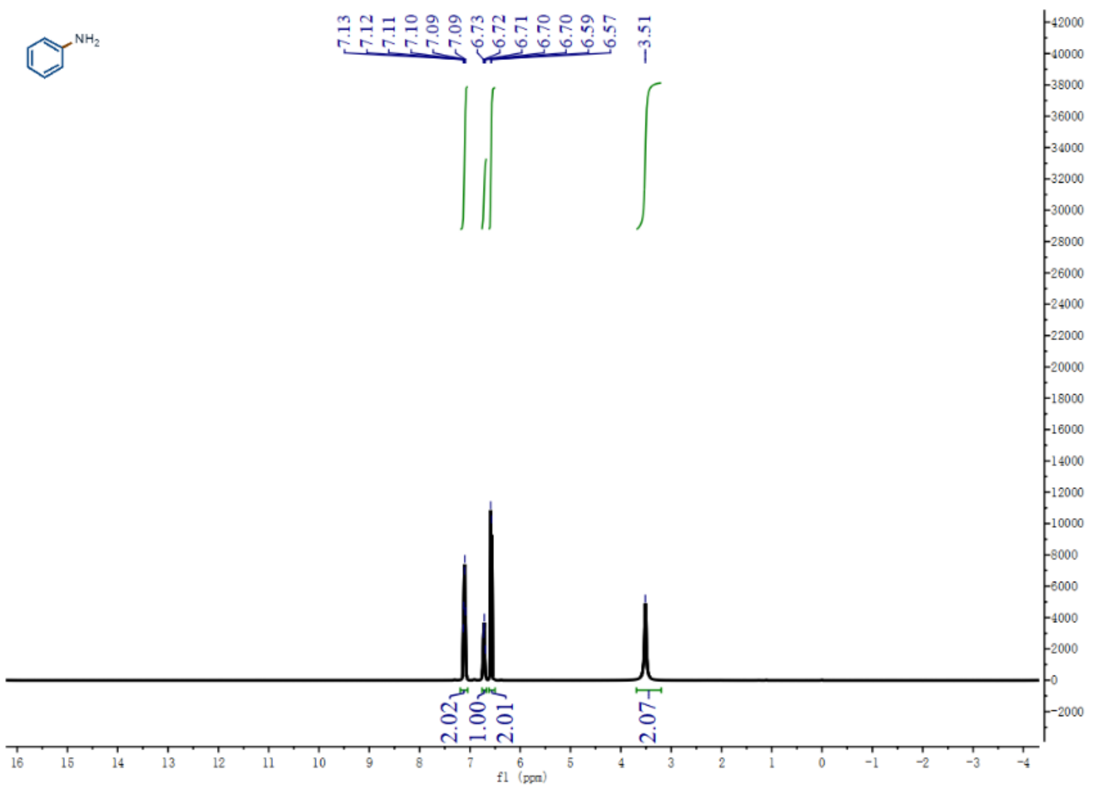

^{13}C NMR (100 MHz, CDCl3) spectrum of compound **18**

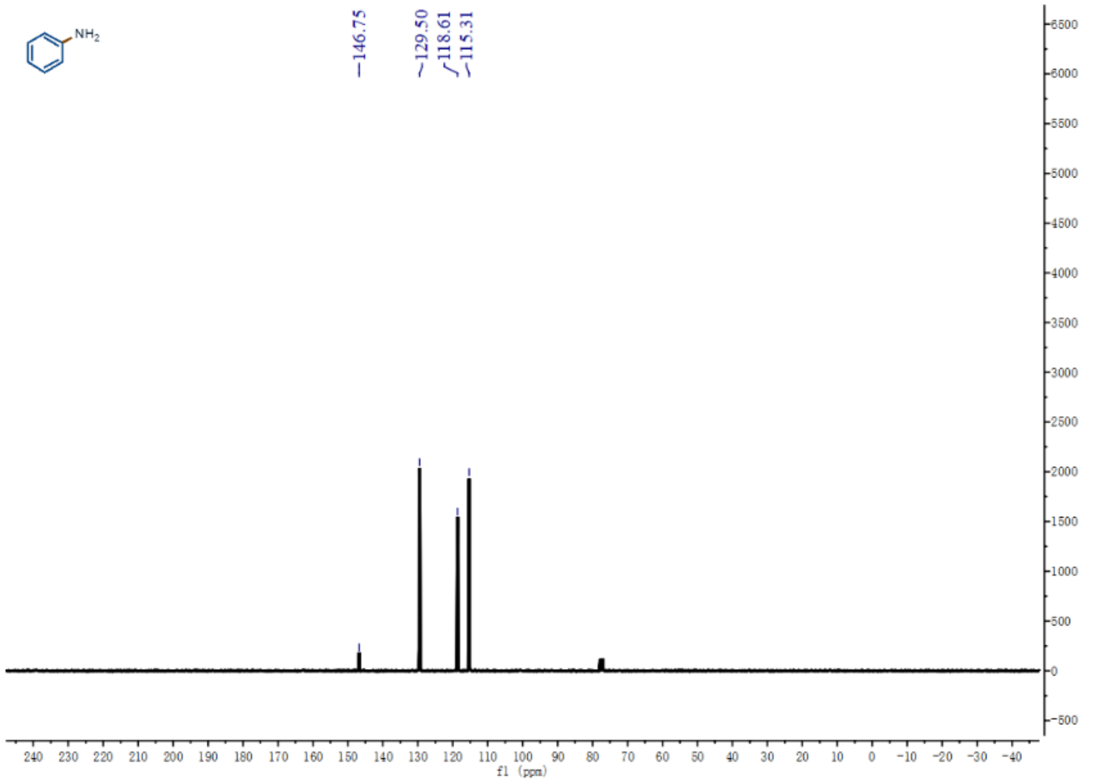

^{1}H NMR (400 MHz, CDCl3) spectrum of compound **19**

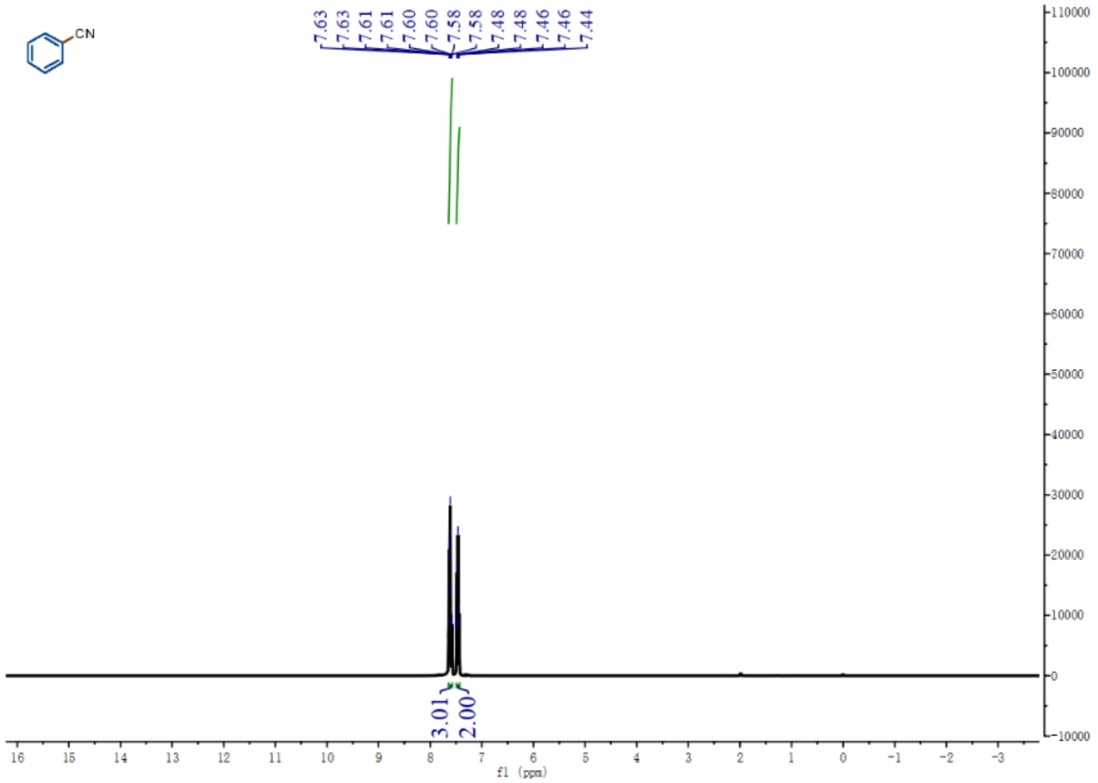

^{13}C NMR (100 MHz, $CDCl_3$) spectrum of compound **19**

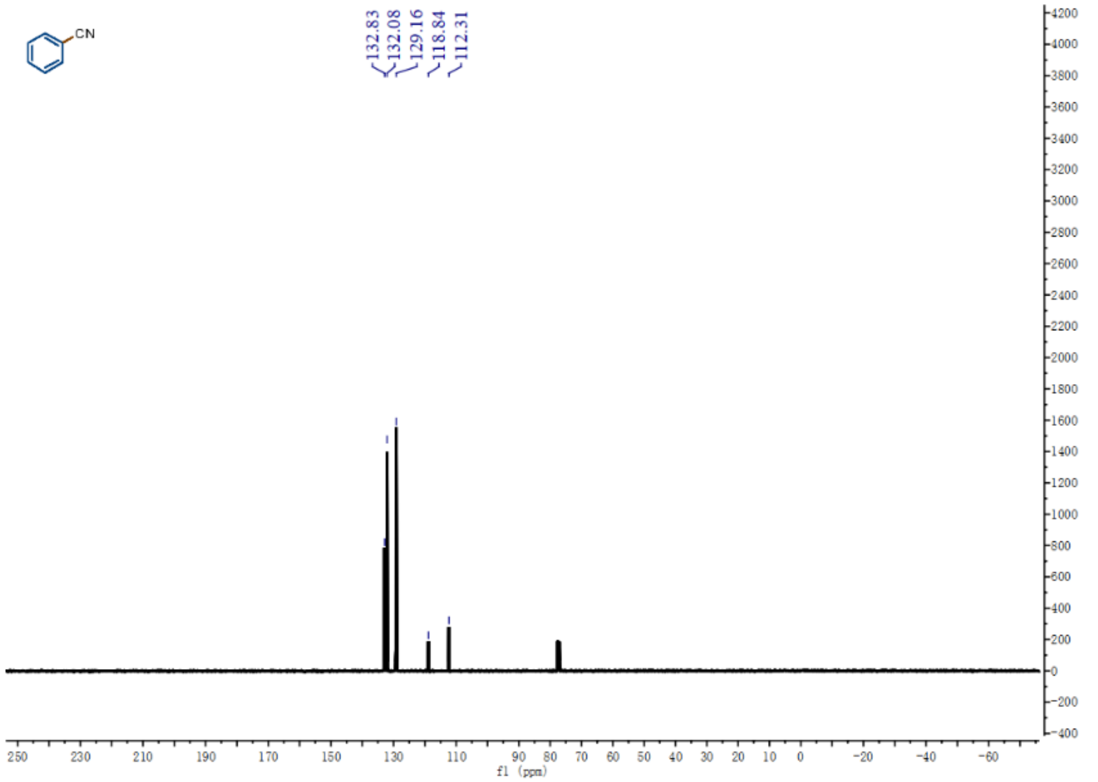

^{1}H NMR (400 MHz, $CDCl_3$) spectrum of compound **20**

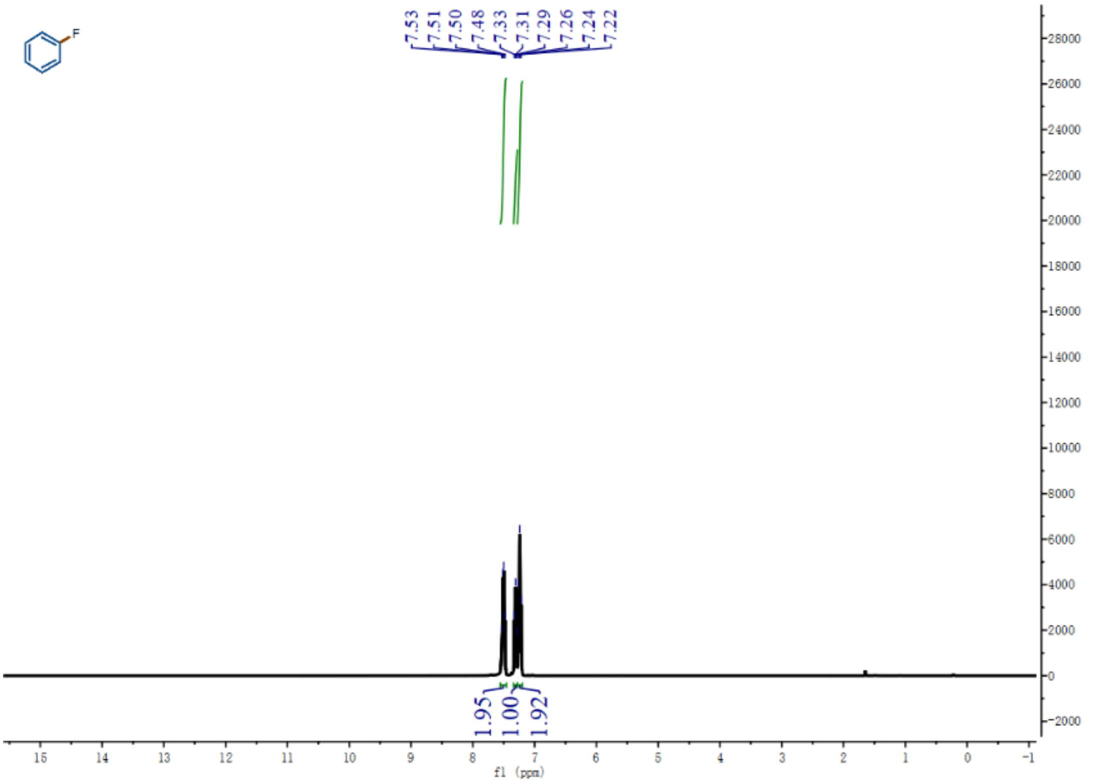

^{13}C NMR (100 MHz, $CDCl_3$) spectrum of compound **20**

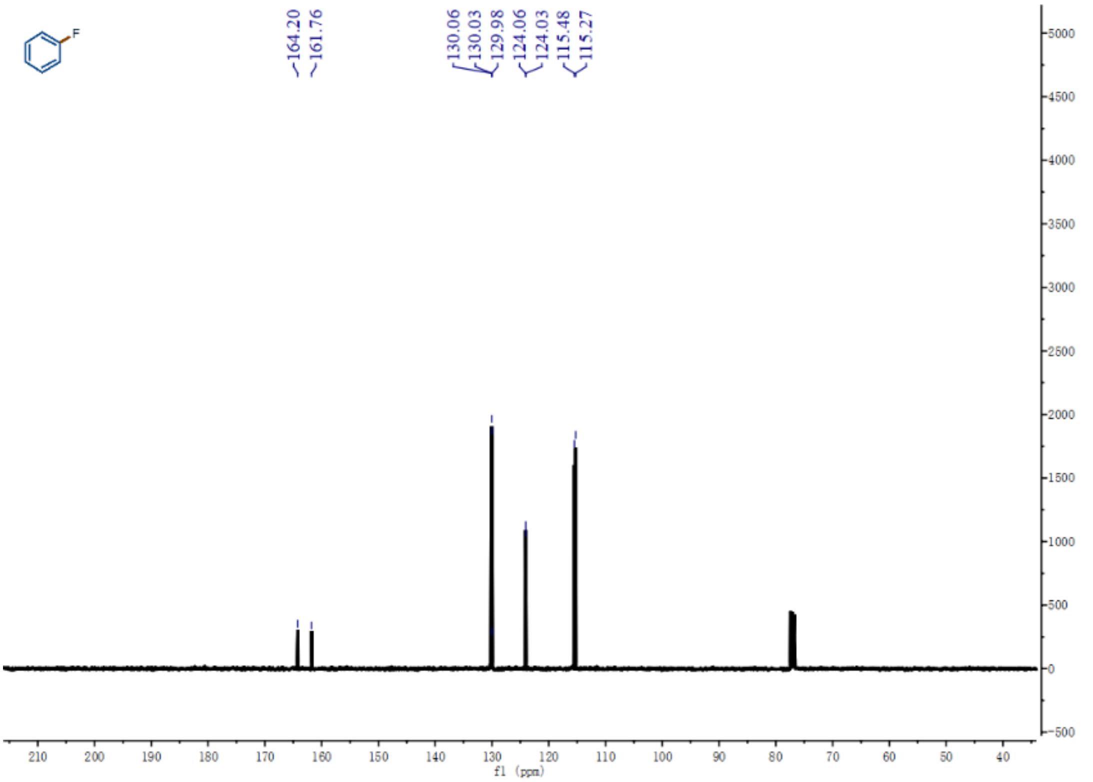

^{1}H NMR (400 MHz, $CDCl_3$) spectrum of compound **21**

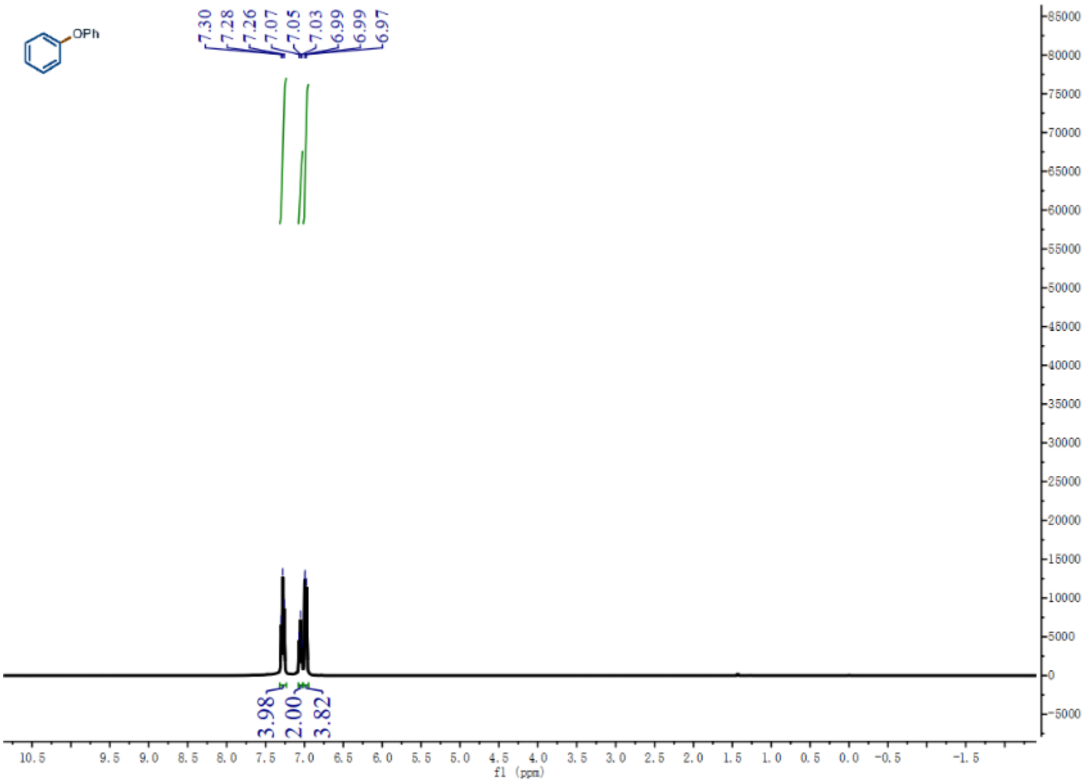

^{13}C NMR (100 MHz, $CDCl_3$) spectrum of compound **21**

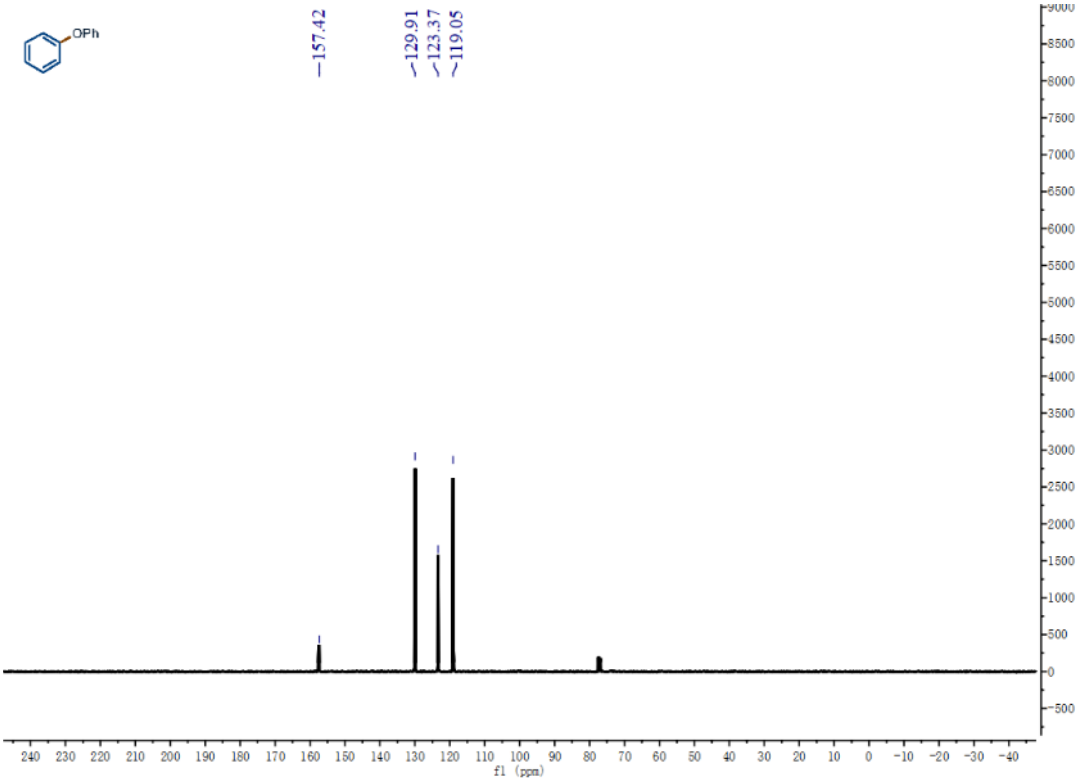

^{1}H NMR (400 MHz, $CDCl_3$) spectrum of compound **22**

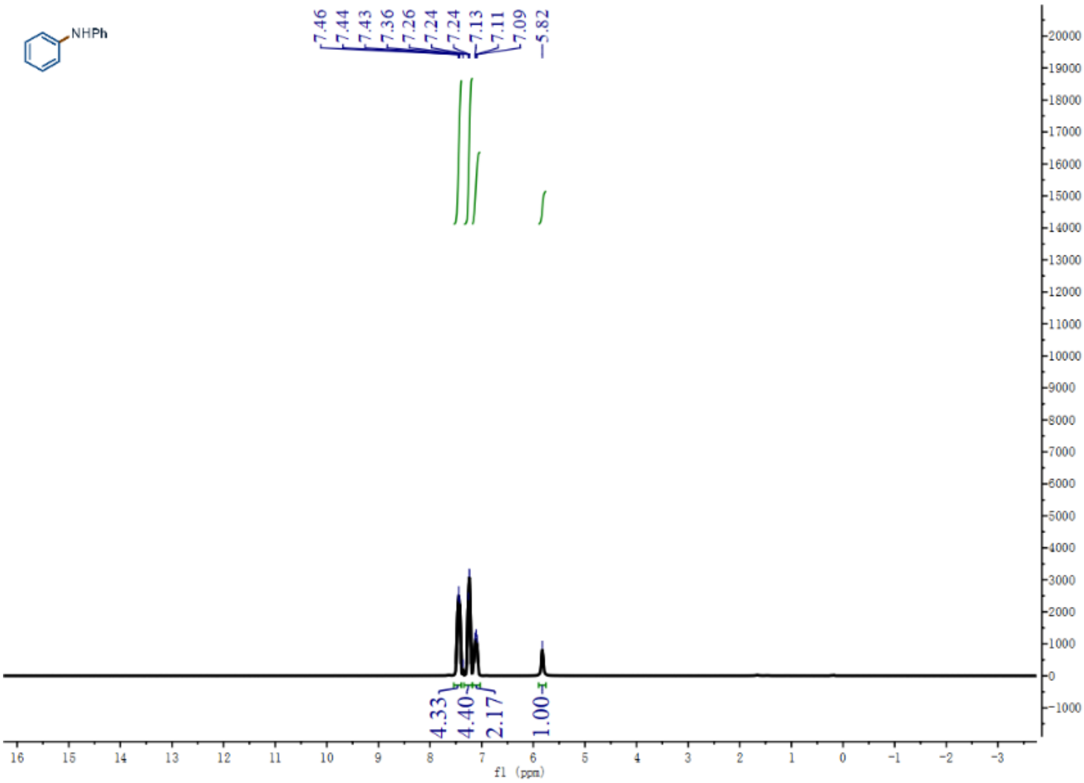

^{13}C NMR (100 MHz, $CDCl_3$) spectrum of compound **22**

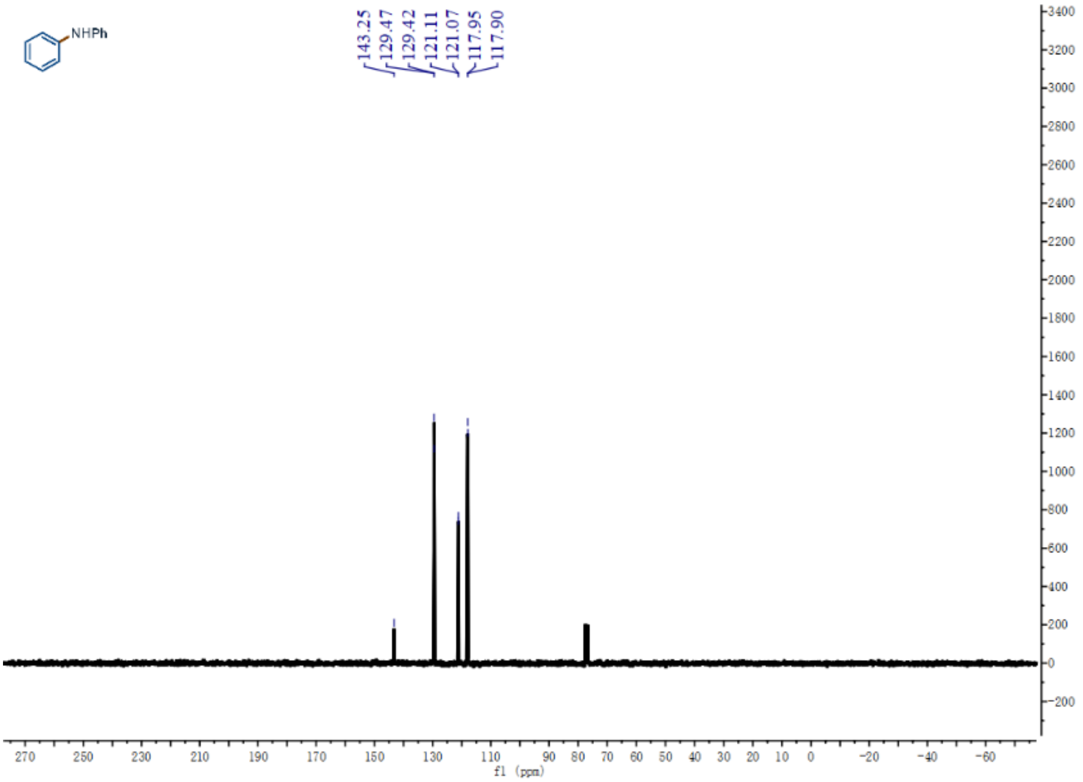

Supplement: Supplementary file 1 — oc3c00993_si_001.pdf [file oc3c00993_si_001.pdf]
